# Large Stokes shift fluorophores from *meta*-substituted zwitterions

**DOI:** 10.1039/d6sc03405e

**Published:** 2026-06-17

**Authors:** David T. Hogan, Alexander R. Krappe, Ralf Feyerherm, Manuela Weber, Ute Resch-Genger, Siegfried Eigler

**Affiliations:** a Institute of Chemistry and Biochemistry (SupraFAB), Freie Universität Berlin Altensteinstraße 23a 14195 Berlin Germany siegfried.eigler@fu-berlin.de; b Institute for Quantum Phenomena in New Materials, Helmholtz-Zentrum Berlin für Materialien und Energie Hahn-Meitner Platz 1 14109 Berlin Germany; c Institute of Chemistry and Biochemistry, Freie Universität Berlin Fabeckstraße 34/36 14195 Berlin Germany; d Department 1, Division Biophotonics, Bundesanstalt für Materialforschung und -prüfung (BAM) Richard-Willstätter-Straße 11 12489 Berlin Germany

## Abstract

Many commonly used dye classes suffer from strong overlap of their absorption and emission spectra, favoring reabsorption and hampering the combination of several dyes for multi-analyte sensing with single wavelength excitation. This can be overcome by increasing the energy difference between absorption and emission using donor–acceptor dyes with charge-transfer processes. A neglected concept to finetune the Stokes shift is *meta*-substitution, which we exploited to design a single-benzene fluorophore exhibiting the largest Stokes shift of a zwitterionic compound. The *meta*-substitution of permanently-charged donor and acceptor groups provides a Stokes shift of >10 000 cm^−1^ (1.24 eV), absorbing light in the UV-region at 375 nm and emitting yellow-orange light at 605 nm. Relative to *para*-substitution, the orbitals are primed for more effective intramolecular charge-transfer and more energy is dissipated by structural reorganisation upon excitation, stemming from greater excited-state antiaromaticity. The large Stokes shift is retained by π-extended derivatives, thus *meta*-substitution of zwitterionic groups is a general way to design organic fluorophores with small spectral overlap.

## Abstract 2

Charged donor and acceptor groups in *meta*-orientation confer longer wavelength emission and greater Stokes shifts to fluorescent dyes than *para*-substitution.

## Graphical abstract

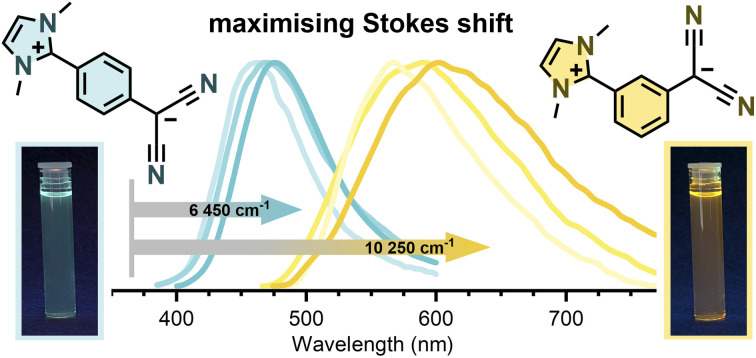

## Introduction

Organic fluorophores have a wide range of applications, comprising light-emitting diodes,^1,2^ dye lasers,^3,4^ holographics,^5,6^ and luminescent solar concentrators.^7,8^ Fluorescent dyes have also become an indispensable part of the chemical biologist's toolbox because they allow sensitive and selective visualisation of biological structures by fluorescence microscopy.^9,10^ The dominant fluorescent dyes for microscopy are based upon xanthene and cyanine core structures known for their high molar absorption coefficients and fluorescence quantum yields.^10–13^ However, these dyes generally suffer from a small energy difference between the exciting and emitted photons, called the Stokes shift.^14^ This leads to overlap in the absorption and fluorescence spectra, which causes self-quenching of emitted light by the inner-filter effect and excitation light backscattering, which reduce the signal-to-noise ratio of the imaging technique.^15^ Several dyes within the xanthene and cyanine structural families have been synthesised which begin to address the problem, however the Stokes shifts are still relatively small (<3000 cm^−1^).^16,17^

Common methods to induce a large Stokes shift include intramolecular charge-transfer (ICT),^18,19^ as well as the twisted and planarised variants (TICT and PLICT),^20–24^ excited-state intramolecular proton-transfer (ESIPT),^25,26^ and the formation of excimers and exciplexes.^27,28^ Additionally, fluorophores can be designed which undergo a large structural reorganisation upon excitation and dissipate energy through non-radiative pathways before emitting a lower energy photon. Such compounds may twist,^29^ or fold and un-fold,^30,31^ as a result of excited-state antiaromaticity (ESAA).^32^ Combining these effects has led to some fluorophores exhibiting staggeringly large Stokes shifts of over 15 000 cm^−1^.^33^

**Fig. 1:**
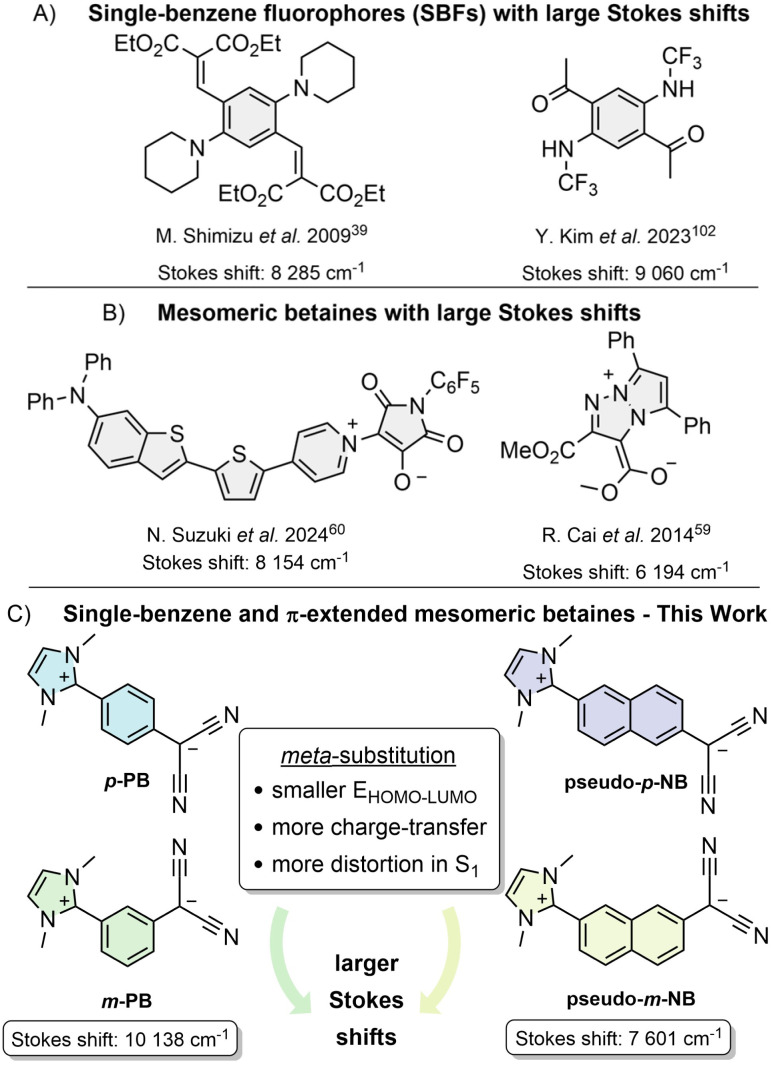
(A) Representative single-benzene fluorophores, (B) fluorescent mesomeric betaines, and (C) mesomeric betaines presented in this study with labelled Stokes shifts.

An increasing number of compounds with large Stokes shifts have come from the single-benzene fluorophore (SBF) structural family.^34,35^ These fluorophores contain a decorated benzene as the key structural component, depicted in Fig. 1A, which itself has a Stokes shift of ∼4400 cm^−1^.^36^ Substitution with strong π–donor and π–acceptor groups can provide compounds with strong absorption,^37^ and high quantum yields both in solution and in the solid state.^38,39^ The term SBF was coined within the last century, although the study of fluorescent substituted benzenes with large Stokes shifts can be identified several decades prior, if not earlier.^40^ In addition, SBFs possess low molecular masses, which is beneficial for increased membrane permeability in cells and decreased synthetic intensity.^41^ Although benzene only contains six carbon atoms, there are multiple reports of hexa-substituted benzenes with smaller Stokes shift and fluorescence quantum yields than tetra-substituted analogues.^42–45^ The basic donor–π–acceptor motif only requires two substituents.^46,47^ Work by Mandal *et al.* neatly summarises that substitution pattern of the donor and acceptor groups has a potent effect on the electronic properties of disubstituted aromatics.^48–50^ Compounds with permanent charges can concentrate positive and negative charge on separate portions of a molecule, simultaneously improving electron-donor and electron-acceptor effects. The resulting sub-class of zwitterions are known as mesomeric betaines, stemming from the term mesomerism.^51–57^ Mesomeric betaines such as those shown in Fig. 1B can even exhibit visible light absorption and fluorescence, possessing Stokes shifts exceeding 6000 cm^−1^.^58–61^

Herein we present a series of new, fluorescent betaines with Stokes shifts >4500 cm^−1^ which have molecular masses below 300 g mol^−1^. The strong electronic perturbation from positively and negatively charged groups through *meta*-substitution imbues betaine *m*-PB with a Stokes shift >10 000 cm^−1^ (1.24 eV) in solution. *m*-PB exhibits the largest Stokes shift of a zwitterionic compound, which originates from a combination of ICT and structural reorganisation upon excitation. This value is among the largest for a disubstituted benzene, as outlined in Section S2 of the SI. As a result, its emission spectrum tails into the near infra-red region^62^ and is 99.9% free from reabsorption. Through a combined spectrometric and computational analysis of *meta*- and *para*-regioisomers, we propose that *meta*-substitution causes a greater change in charge separation, dipole moment, and excited-state antiaromaticity. The large Stokes shift is retained when the substituents are positioned across a naphthylene core. Overall, our findings show that (pseudo)-*meta*-substitution with zwitterionic groups could be a general way to design large Stokes shift fluorophores from π-extended aromatic scaffolds.

## Results and discussion

### Structural characterisation

**Fig. 2:**
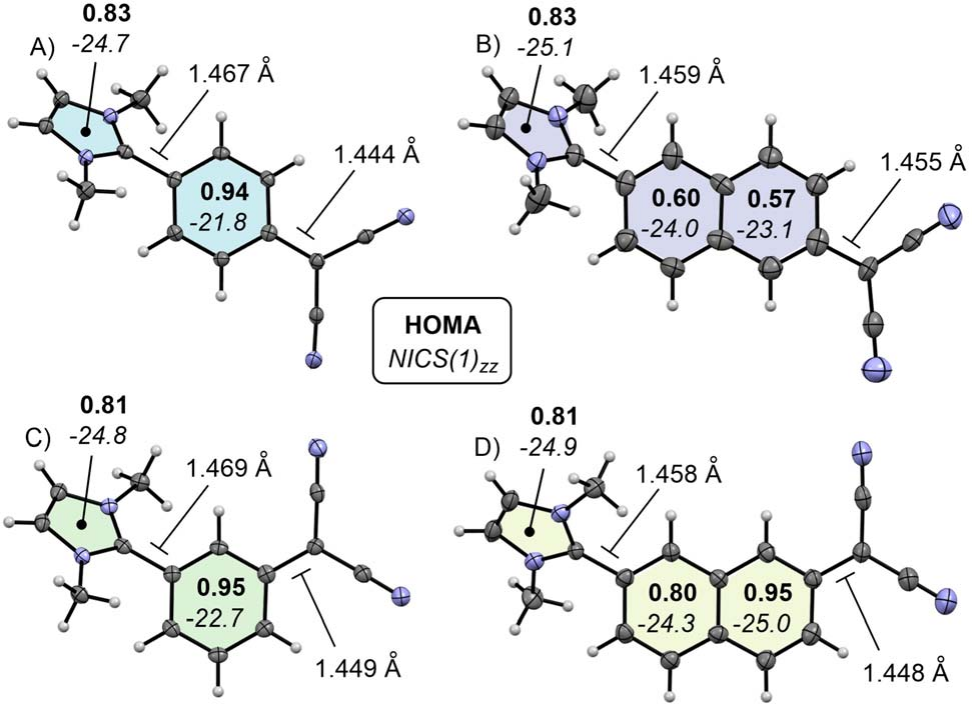
Crystal structures of (A) *p*-PB, (B) pseudo-*p*-NB, (C) *m*-PB, and (D) pseudo-*p*-NB with 50% probability ellipsoids. HOMA (bold) and NICS(1)_zz_ (italics) values are labelled inside each ring along with critical bond distances.

Full synthetic details, ^1^H and ^13^C nuclear magnetic resonance (NMR), and mass spectrometric characterisations can be found in Sections S3 and S4 of the SI. All compounds are indefinitely air and moisture-stable crystalline solids with melting points >250 °C in air. Due to the co-localisation of positive and negative charges within *m*-PB and pseudo-*m*-NB in Fig. 1C, they are classified as pseudo-cross-conjugated mesomeric betaines (PCCMBs).^63–65^ Although the term zwitterion also has general acceptance, here the synonymous name betaine (hence phenylene betaine PB and naphthylene betaine NB) will be used to obey the rigorous classification system of Ollis, Stanforth, and Ramsden in Section S5.^66^ The connectivity of the four betaines is confirmed by X-ray crystallography in Fig. 2 – further details can be found in Section S6. The imidazolium–aryl and malonide–aryl C–C bond lengths of *m*-PB and pseudo-*m*-NB are similar to their *para*-analogues *p*-PB and pseudo-*p*-NB. These bond lengths are also within 0.01 Å of the analogous bonds in phenylimidazolium nitrate^67^ and triphenylaminophosphonium phenylmalonide,^68^ which are both unequivocally described as delocalisation-shortened single bonds. The lack of shortened C=C bonds in *p*-PB and pseudo-*p*-NB indicates that the charge-separated depiction in Fig. 1C reflects their true structures, and therefore the use of betaine nomenclature is applicable.

The harmonic oscillator model of aromaticity (HOMA)^69,70^ and the nucleus-independent chemical shift (NICS)_zz_^71,72^ in Fig. 2 both indicate that the aromaticity of the benzenoid rings of *m*-PB and pseudo-*m*-NB is intact. Relative to benzene and naphthalene,^73^ the HOMA values close to 1.0 suggest little structural deformation from ideal aromatic rings, while the strongly negative NICS(1)_zz_ values are indicative of diamagnetic shielding from aromatic ring currents. The aromaticity of the imidazolium rings is also retained relative to 1,3-dimethylimidazolium iodide.^74^ Detailed results are in Section S7, along with comparison of NICS(1)_zz_ values to cationic and anionic reference compounds. The values for *p*-PB are of similar magnitude to those of *m*-PB, and even though the HOMA values for pseudo-*p*-NB indicate some bond-length alternation in the naphthylene π-system,^75^ the strongly negative NICS(1)_zz_ values are retained. Retention of local aromaticity in both *meta*- and *para*-betaines favours the charge-separated over the quinoidal depiction of their structures.

**Fig. 3:**
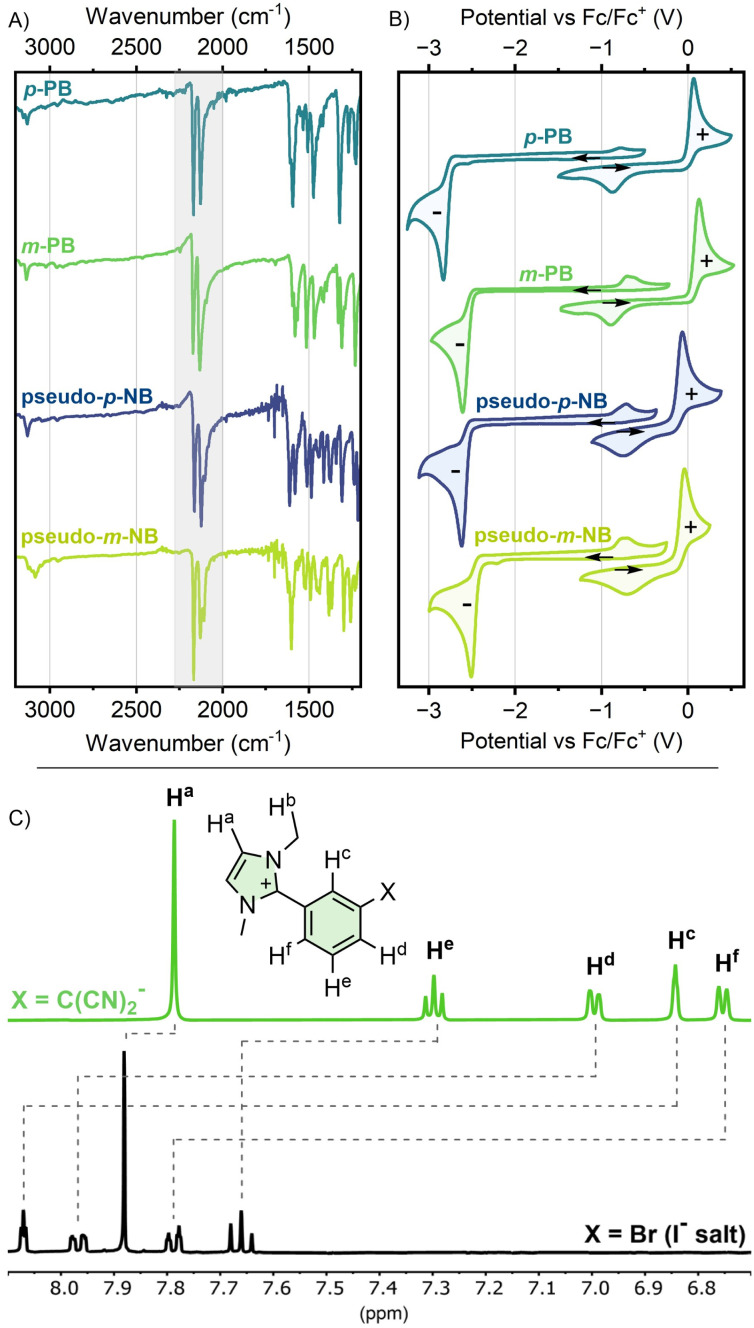
(A) Infra-red transmission spectra and (B) cathodic (−) and anodic (+) cyclic voltammograms of *p*-PB (teal), *m*-PB (green), pseudo-*p*-NB (blue), and pseudo-*m*-NB (lime) in CH_3_CN at 500 mV s^−1^. (C) Aromatic region of the 500 MHz ^1^H NMR spectrum of *m*-PB (green) and 2-(3-bromophenyl)-1,3-dimethylimidazolium iodide (black) in DMSO-*d*_6_ at 298 K.

The symmetric and antisymmetric C≡N stretching modes in the infra-red spectra of all four betaines (2164–2172 cm^−1^ and 2116–2123 cm^−1^) in Fig. 3A occur at similar energies as those of anionic reference compounds in Section S8. The frequency and splitting of those modes are contrasted with the single C≡N stretch of neutral phenyl- and 2-naphthylmalononitrile. Thus, the cyano substituents in *m*-PB, *p*-PB, pseudo-*m*-NB and pseudo-*p*-NB all bear significant negative charge. The central imidazolium carbon atoms in ^13^C NMR spectra of the *meta*- and *para*-betaines resonate at 144.9–145.5 ppm, while the central malonide carbon atoms resonate at 29.1–31.5 ppm. These values are very similar to reference compounds in Section S9 which supports the notion of intact carbocationic and carbanionic moieties.^76^ The ^1^H NMR in Fig. 3C shows that the protons *ortho*- and *para*- to the malonide group in *m*-PB experience dramatic shielding of Δ*δ* = −0.97, −1.03, −1.22. The significant shielding effect is retained in *p*-PB, pseudo-*m*-NB, and pseudo-*p*-NB (Δ*δ* = −0.34 to −1.34) which confirms that considerable negative charge is delocalised within the aromatic core of all four betaines (full details in Section S9).

Having determined that *m*-PB, *p*-PB, pseudo-*m*-NB and pseudo-*p*-NB are all charge-separated betaines, the electronic consequences of *meta*- *versus para*-substitution were explored by cyclic voltammetry (CV). Reduction and oxidation are irreversible and occur at comparable potentials to reference compounds bearing charged fragments in Section S10. The observed irreversibility is likely linked to an electrochemical–chemical–electrochemical (ECE) mechanism in the CV in Fig. 3B.^77,78^ Following reduction or oxidation of the betaines, the nascent radical anions or radical cations separately undergo a rapid chemical reaction, whose products are subsequently oxidised or reduced and give rise to the small faradaic current between −0.50 V and −1.00 V *vs.* Fc/Fc^+^. This ECE behaviour was also recorded for reference compounds bearing imidazolium and malonide fragments (Section S10), further supporting the notion that the four betaines contain oppositely charged moieties. Comparison of the potentials from differential-pulse voltammetry (DPV) in Table 1 reveals that *m*-PB and pseudo-*m*-NB have tighter HOMO–LUMO gaps than their *para*-analogues by ∼0.2 eV. This suggests that *meta*-substitution provides a distinct advantage when tuning the HOMO–LUMO gap in organic electronic materials.

**Table 1:** Optical, electrochemical, and theoretical data in CH_3_CN of all betaines

	Absorption maximum *λ*_abs_^a^ (nm)	Molar absorption coefficient *ε*^a^ (L mol^−1^ cm^−1^)	Fluorescence maximum *λ*_em_^a^ (nm)	Stokes shift^a^ (cm^−1^)	Fluorescence lifetime *τ*_F_^a^ (ns)	Quantum yield *Φ*_F_^a^	*E* ^elec^_gap_ b (eV)	*E* ^opt^_gap_ c (eV)	*E* ^theo^_gap_ d (eV)
*p*-PB	366	29 500	470	6046	<1	<0.01	2.78	3.00	3.92
*m*-PB	375	1600	605	10 138	1.36	0.01	2.56	2.96	3.73
pseudo-*p*-NB	424	13 600	544	4397	2.83	46.9	2.62	2.58	3.35
pseudo-*m*-NB	444	2800	641	7601	2.06	0.02	2.41	2.56	3.34

a Values in CH_3_CN solution.

b Determined from peak potentials in DPV.

c Determined from the 0–0 transition wavelength (Table S18).

d CAM-B3LYP/def2-TZVP.

### Spectroscopic characterisation

**Fig. 4:**
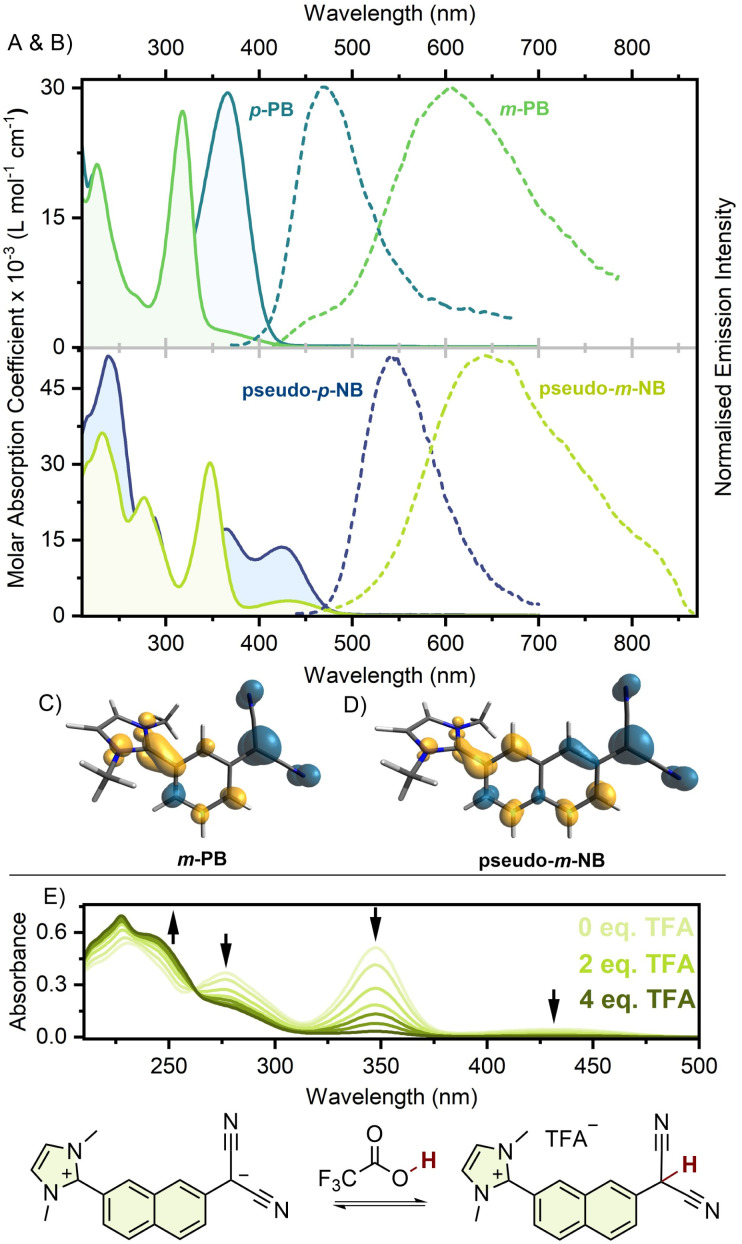
Absorption spectra (solid) with associated fluorescence spectra (dash) in CH_3_CN of (A) *m*-PB (green, *λ*_exc_ 400 nm) & *p*-PB (teal, *λ*_exc_ 370 nm) and (B) pseudo-*m*-NB (lime, *λ*_exc_ 430 nm) & pseudo-*p*-NB (blue, *λ*_exc_ 420 nm). Charge density difference of loss (teal) and gain (gold) during S_1_ excitation of (C) *m*-PB and (D) pseudo-*m*-NB. (E) Arrows mark the changes in absorption spectra of pseudo-*m*-NB in CH_3_CN as 4 equiv. of TFA are titrated.

The dominant bands at 226 nm and 318 nm in the UV-vis absorption spectrum of *m*-PB in acetonitrile, shown in Fig. 4A, originate from electronic transitions within the individual arylimidazolium and arylmalonide moieties. Those transitions are also responsible for the bands at 277 nm and 347 nm in the absorption spectrum of pseudo-*m*-NB in Fig. 4B. The assignments are made by comparison to reference compounds in Section S11. The electronic transition between the two oppositely charged moieties of *m*-PB emerges as a low-intensity shoulder band at ∼375 nm and 431 nm for pseudo-*m*-NB. The concentration-independence of these features confirms that they do not originate from aggregation effects (Section S12), which is consistent with related dyes.^79^ The redistribution of electrons between the singlet ground (S_0_) and excited (S_1_) states is confirmed by charge density difference maps^80,81^ from time-dependent density functional theory (TD-DFT) with the CAM-B3LYP^82^ functional in Section S13 (Fig. S62–S69).^83^ S_0_–S_1_ excitation from the electron-rich malonide moiety onto the electron-poor imidazolium moiety in Fig. 4C and D closely follows the HOMO–LUMO distributions. The high degree of spatial separation of these orbitals (*t* index, Table S19) confirms the donor–acceptor character of *m*-PB and pseudo-*m*-NB. Thus, the lowest energy transition is best described as an intramolecular charge-transfer (ICT). The corresponding HOMO–LUMO transition in *p*-PB and pseudo-*p*-NB occurs at slightly shorter wavelengths of 366 & 424 nm (Table S31) and with greater oscillator strengths (1.10 & 1.09) relative to *m*-PB and pseudo-*m*-NB (0.05 & 0.09). This effect is also explained by TD-DFT, which predicts greater transition electric dipole moment (*µ*_elec_) and greater overlap of the involved orbitals (*S*_r_) for *p*-PB and pseudo-*p*-NB, shown in Table S19. The HOMO–LUMO energy gaps determined from experiment and calculation are listed in Table S18, which are all in agreement that the *meta*-betaines have smaller energy gaps than their *para*-analogues.

Spectrometric titration with trifluoroacetic acid (TFA) in Section S14 offers further insight on the location and function of the separated charges to the electronic makeup of the betaines. As represented by pseudo-*m*-NB in Fig. 4E, the ICT and malonide absorption bands at 431 nm 347 nm are diminished upon addition of TFA while the imidazolium spectral features are retained. After full titration, the absorption and emission profiles closely match a 2-naphthylimidazolium reference compound. Such behaviour suggests that the donor–acceptor character has been interrupted by protonation of the malonide moiety. This reactivity is exhibited by *m*-PB, *p*-PB, pseudo-*m*-NB and pseudo-*p*-NB and strongly supports the ICT character of their HOMO–LUMO transition.

**Fig. 5:**
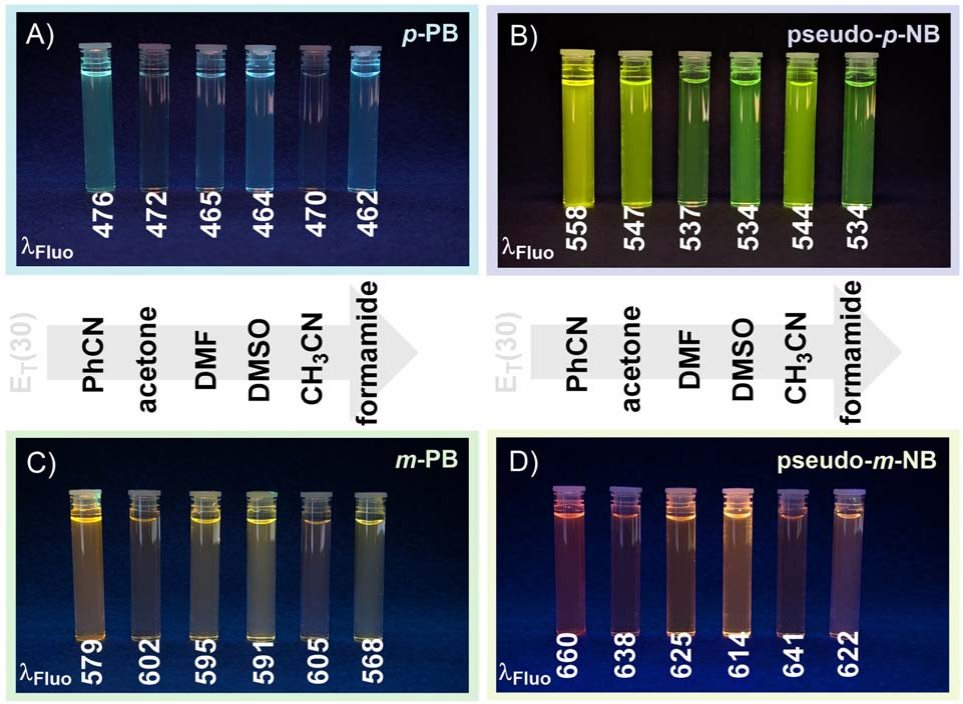
Photographs of (A) *p*-PB, (B) pseudo-*p*-NB, (C) *m*-PB, and (D) pseudo-*m*-NB under 365 nm irradiation in solvents arranged according to relative *E*_T_(30) value. Emission maxima are labelled in wavelengths below each solution.

Photoexcitation of *m*-PB and pseudo-*m*-NB leads to orange fluorescence at 605 nm and 641 nm in acetonitrile (Fig. 4A and B).^84^ The slight shoulder at ∼475 nm could be emission from a locally-excited (LE) state, although it does not exhibit the expected behaviour in solvents of differing polarity (Fig. S126). Considering that the absorption peak of *m*-PB is in the UV region, it has a large Stokes shift of 10 138 cm^−1^, which to our knowledge is the largest of any zwitterion/mesomeric betaine (Section S2). The Stokes shift of π-extended pseudo-*m*-NB in acetonitrile is slightly smaller (7601 cm^−1^), and no evidence for excimerism is observable in solutions of higher concentrations (Section S12). Although neither of these *meta*-betaines qualify as zero-overlap fluorophores,^33^ the emission spectra are 99.9% and 99.7% free from reabsorption effects, which is an important metric for evaluation of dyes for fluorescence microscopy.^9^ The *para*-betaines *p*-PB and pseudo-*p*-NB fluoresce at 470 nm and 544 nm in acetonitrile (Fig. 5A and B), which result in diminished Stokes shifts (Table S32) and greater spectral overlap (Table S30).

### Dipole contribution to Stokes shift

To derive general design criteria for large Stokes shift dyes, we calculated the fluorescence spectra using the excited state dynamics (ESD) function of ORCA.^85–88^ Comparison of the spectra in Fig. S72–S87 predicted the Stokes shifts for *m*-PB and pseudo-*m*-NB to be larger than *p*-PB and pseudo-*p*-NB, which is in line with experimental observation (Table S19). The charge density difference between the malonide and imidazolium moieties from S_0_ to S_1_ in Fig. 4C and D indicate that ICT could be a possible explanation. The emission maxima of *m*-PB are shifted hypsochromically in solvents of higher polarity in Fig. 5C, from 605 nm in acetonitrile to 568 nm in formamide. Likewise, the emission of pseudo-*m*-NB exhibits a hypsochromic shift from 660 nm to 614 nm. A smaller effect is observed for *p*-PB (476 nm to 462 nm) and pseudo-*p*-NB (558 nm to 534 nm) in Fig. 5A and B. Such negative solvatochromism is an indication of a diminished molecular dipole moment in the excited state. Furthermore, the larger solvatochromic shift of the *meta*-betaines indicates that their dipoles decrease more than their *para*-analogues. Attempts to quantify this change revealed a poor correlation to Lippert and Mataga's orientation polarizability,^89–91^ and Reichardt's *E*_T_(30) parameter (Section S15).^92,93^ The poor correlation indicates that ICT is not the only factor which governs the solvatochromism, and additional effects are responsible for the larger Stokes shifts of the *meta*-betaines.

The DFT-calculated changes in dipole moment between S_0_ and S_1_ reinforce the difference between *meta*- and *para*-betaines: *m*-PB and pseudo-*m*-NB lose more dipole moment upon excitation relative to *p*-PB and pseudo-*p*-NB (Table S19). The *meta*-betaines have a larger *t* index^80,81^ than the *para*-betaines, which suggests that the frontier orbitals of *m*-PB and pseudo-*m*-NB are more localised onto the charged malonide and imidazolium moieties. Upon excitation, the charge-transfer between them more effectively neutralises those formal charges, quenching the molecular dipole. Put in other words, the *meta*-substitution creates orbitals which are better localised to permit a greater dipole in the ground state and a lesser dipole after excitation.

**Fig. 6:**
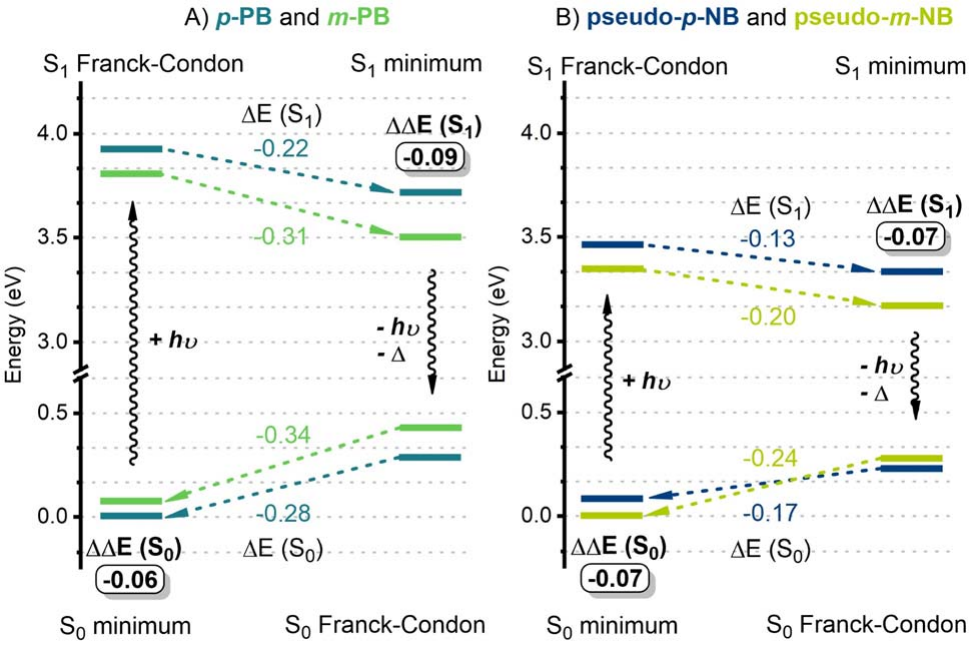
Relative energy diagrams showing relaxation energies after excitation and de-excitation of (A) *m*-PB (green) & *p*-PB (teal) and (B) pseudo-*m*-NB (lime) & pseudo-*p*-NB (blue) at the CAM-B3LYP/def2-TZVPD-D4 (SMD-DRACO) level in CH_3_CN.

The greater change in dipole moment upon de-excitation also contributes to the larger Stokes shifts of the *meta*-betaines. The solvent environment rearranges in response to changes in solute molecular dipole after (de-)excitation. Energy diagrams in Fig. 6 of the four critical structures involved in excitation and de-excitation were constructed using DFT calculations with the DRACO solvation scheme, which is particularly suited for solvating charged species.^94^ Full details are in Fig. S89 and S90. The calculations faithfully reproduce the lower energy gaps of *meta*-betaines observed from absorption and fluorescence spectroscopy. Energy differences are small but consistent: both *m*-PB (Fig. 6A) and pseudo-*m*-NB (Fig. 6B) dissipate >0.06 eV more energy in S_1_ and S_0_ after relaxing from the Franck–Condon state to the minimum-energy geometry. The result is more stable S_1_ minima and less stable S_0_ Franck–Condon states, which combine to decrease the energy of fluorescence.

### Structural contribution to Stokes shift

**Fig. 7:**
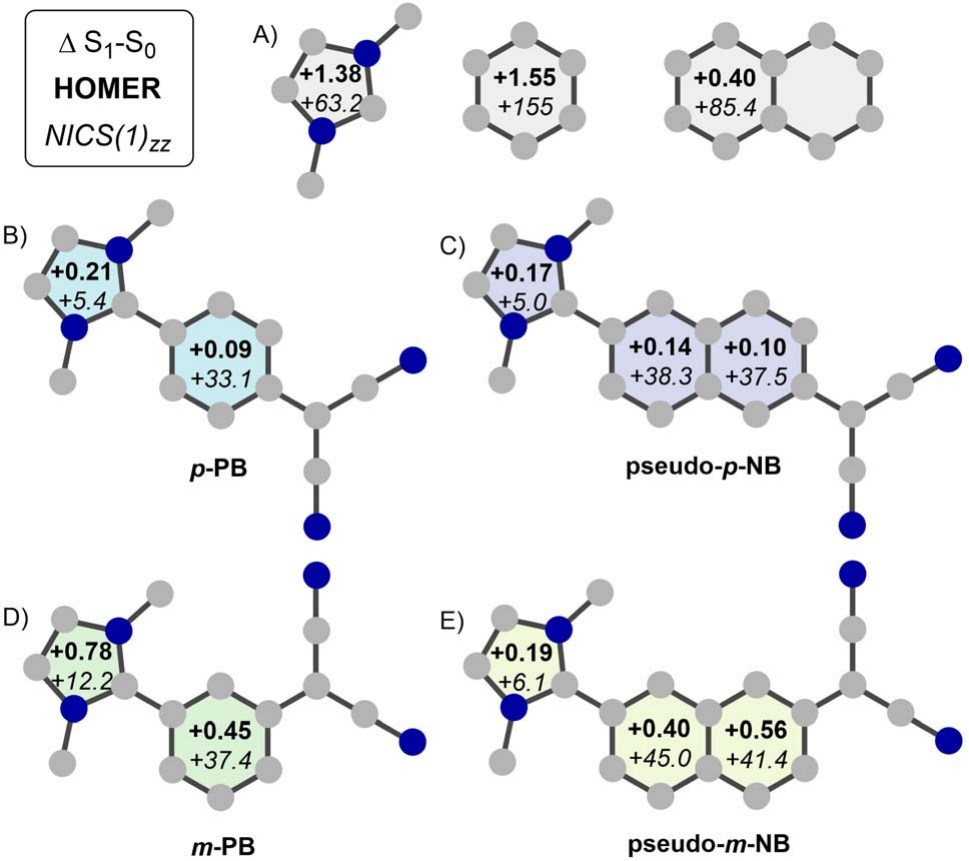
Changes in HOMER (bold) and NICS(1)_zz_ indices between S_0_ and S_1_ of (A) 1,3-dimethylimidazolium, benzene, and naphthalene (B) *p*-PB, (C) pseudo-*p*-NB, (D) *m*-PB, and (D) pseudo-*m*-NB at the CAM-B3LYP/pcSseg-2 level.

Structural distortion of the aromatic rings could also be an avenue for energy dissipation after excitation. This proposal is based upon the concept of Baird aromaticity, in which the excited triplet^95,96^ and singlet^97–99^ states of Hückel antiaromatic [4*n*]-annulenes exhibit pronounced stability from electronic delocalisation. The complement is that excited states of Hückel aromatic [4*n* + 2]-annulenes become antiaromatic.^100^ The significant positive shifts of the NICS(1)_zz_ indices of structural reference compounds from S_0_ to S_1_ in Fig. 7A reflect their excited-state antiaromaticity (ESAA). Likewise, the rings of all four betaines exhibit pronounced antiaromaticity in S_1_ in Fig. 7B–E. Full details are in Section S7. However, the NICS(1)_zz_ indices for *m*-PB and pseudo-*m*-NB increase more than for the *para*-betaines. This suggests that *m*-PB and pseudo-*m*-NB experience greater ESAA upon excitation.

The structural reorganisation which accompanies such antiaromaticity has been used to explain the anomalous Stokes shifts of other single-benzene fluorophores.^101,102^ We relied upon the harmonic oscillator model of excited-state aromaticity (HOMER)^103^ of Arpa and Durbeej to index the changes in geometry upon excitation. Positive values indicate Hückel antiaromaticity, while negative values indicate Hückel aromaticity. Calculations on structural reference compounds in Fig. 7A reveal encouraging changes upon transitioning from the S_0_ to S_1_ relaxed geometry. Upon transitioning from S_0_ to S_1_ geometries, the rings of all four betaines experience a positive shift in HOMER index in Fig. 7B–E. However, the indices for *m*-PB and pseudo-*m*-NB shift considerably more than for their *para*-analogues. Thus, *m*-PB and pseudo-*m*-NB are more Hückel antiaromatic in their S_1_ state than either *p*-PB or pseudo-*p*-NB. Full details are in Section S8. Increased ESAA has consequences on the energy diagram in Fig. 6. Since the HOMER index is based on geometry, the *meta*-betaines experience more structural reorganisation than the *para*-betaines. Therefore, more energy is dissipated after (de-)excitation from the Franck–Condon states to reach the S_1_ and S_0_ minimum geometries. Thus, ESAA contributes to the large observed Stokes shifts in fluorescence of *m*-PB and pseudo-*m*-NB. As proposed by Filatov *et al.*, here ESAA works alongside ICT and dipole moment effects.^104^

### TICT contribution to Stokes shift

**Fig. 8:**
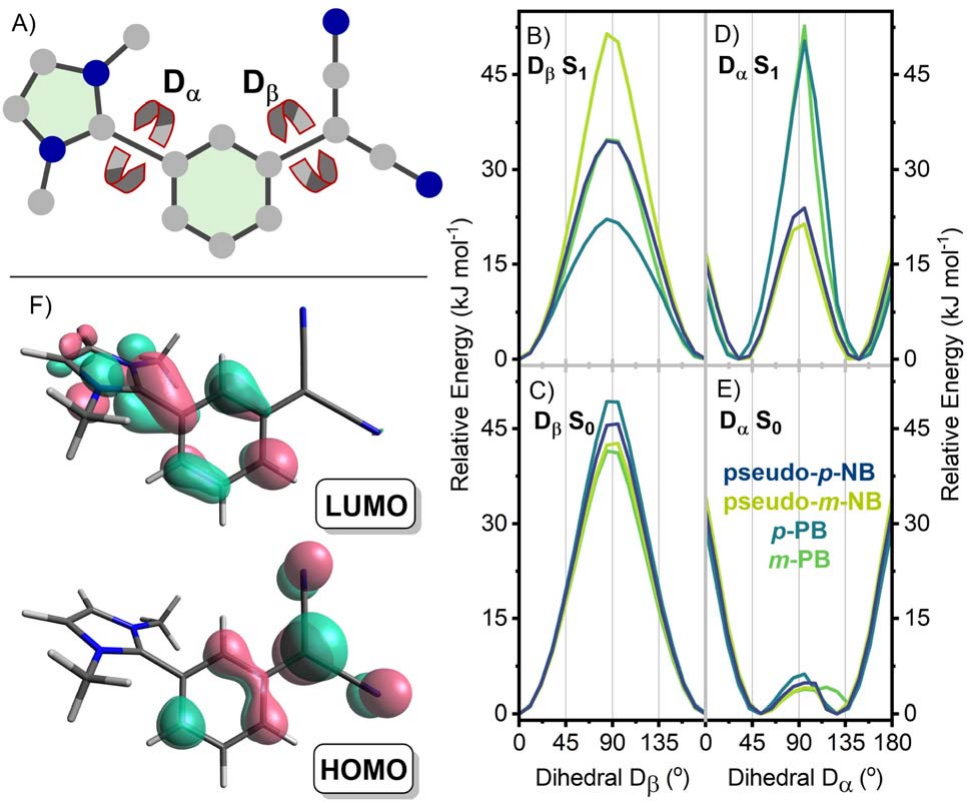
(A) Diagram of *m*-PB showing the *D*_α_ and *D*_β_ dihedral angles. Rotational barriers around (B) *D*_β_ in S_1_ and (C) *D*_β_ in S_0_; (D) *D*_α_ in S_1_ and (E) *D*_α_ in S_0_ for *p*-PB (teal), *m*-PB (green), pseudo-*p*-NB (blue), and pseudo-*m*-NB (lime) at the CAM-B3LYP/def2-SVP-D4 level in CH_3_CN. (F) Kohn–Sham frontier molecular orbitals of *m*-PB.

Rotation around aryl–aryl single bonds in donor–π–acceptor systems has been implicated in twisted intramolecular charge-transfer (TICT)^20,21^ and related phenomena.^23,24^ To determine if TICT contributes to the four betaines here, we calculated the rotational energy barriers around dihedral angles *D*_α_ and *D*_β_ in the S_0_ and S_1_ states in Fig. 8A and Section S16. The malonide and aryl moieties of all four betaines are predicted to be virtually co-planar and remain so after excitation. This corresponds to the energy minima at 0° and 180° on the potential energy surface (PES) for *D*_β_ in both S_0_ and S_1_ in Fig. 8B and C. The aryl-malonide bond is therefore excluded from participating in TICT. The S_0_ PES for *D*_α_ of all four betaines has energy maxima at 0° and 180° in Fig. 8E from steric repulsion between the imidazolium methyl groups and the *ortho*-hydrogen atoms on the aryl ring. Upon excitation into S_1_, the PES has a new global maximum at 90° which originates from increased bond order between the aryl and imidazolium moieties, based on the LUMO of *m*-PB in Fig. 8F. Full details are in Section S16. The energetically unfavourable orthogonal conformation in S_1_ confidently excludes the TICT and PLICT mechanisms from consideration.^105,106^

### Fluorescence quantum yields and lifetimes

The fluorescence quantum yields (*Φ*_F_) of *m*-PB, pseudo-*m*-NB and *p*-PB in polar organic solvents are low: ∼0.01, 0.02–0.07, and <0.01. Correspondingly, their fluorescence lifetimes (*τ*_F_) decay quickly within 1−5 ns (Table S34) and do not show a clear trend with common solvent parameters. In contrast to the other betaines, the *Φ*_F_ of pseudo-*p*-NB ranges from 43–76%, which is an astonishing 100-fold increase over *p*-PB. Thus, there is no observable *meta*-/*para*-effect in this dataset. The fluorescence lifetimes contain no delayed component from reverse intersystem crossing (RISC) (Section S17) and emission intensities are comparable whether conducted in de-oxygenated solvent or not (Section S18). Thus, there is no evidence for contribution from triplet states to photon emission unless they rapidly lead to thermal de-excitation. Calculation of the singlet and triplet potential energy surfaces (in Fig. S159–S166) reveals that intersystem crossing from S_1_ to T_*n*_ states is accessible through rotation of the aryl-imidazolium bond. It was previously shown for related compounds that easy access to triplet states was responsible for quenching fluorescence,^107^ thus we conclude that the betaines studied here follow a similar de-excitation pathway. The S_1_–T_*n*_ spin–orbit coupling constants (SOC, *ζ*) calculated by TD-DFT of pseudo-*p*-NB are consistently the lowest among the four betaines. This has two effects. It further supports the proposal that the rapid non-radiative de-excitation of *m*-PB, pseudo-*m*-NB and *p*-PB involves triplet states. Secondly, it indicates that population of triplet states would be the least efficient for pseudo-*p*-NB, potentially explaining the higher quantum yield.

### Influence of viscosity on fluorescence

**Fig. 9:**
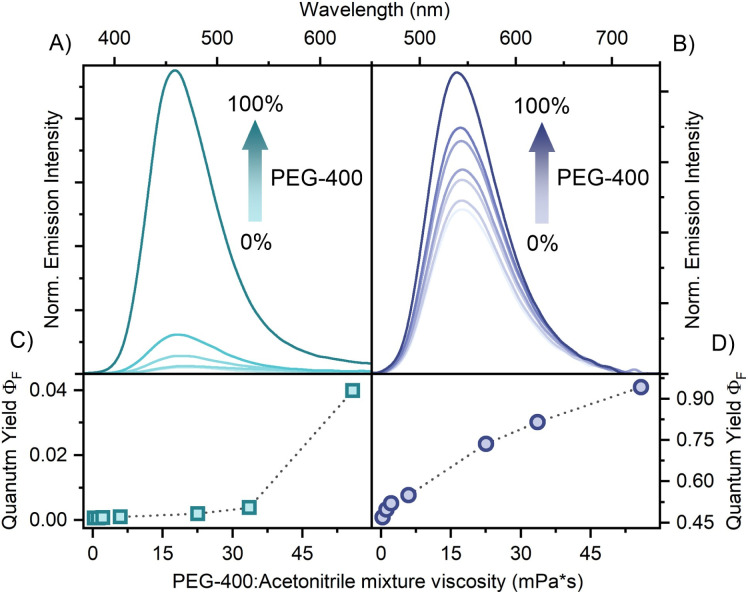
Fluorescence spectra recorded in mixed PEG-400 : CH_3_CN solvent for (A) *p*-PB (teal, *λ*_exc_ 370 nm) and (B) pseudo-*p*-NB (blue, *λ*_exc_ 420 nm). (C & D) Fluorescence quantum yields as a function of solvent viscosity.

Short-lived excited states could originate from rapid de-excitation by molecular motions, which can be influenced by solvent viscosity. As detailed in Section S19, *τ*_F_ and *Φ*_F_ increase roughly 3-fold for *m*-PB and pseudo-*m*-NB. Those of *p*-PB in Fig. 9A and C increase by 5-fold and 67-fold from 0–100% PEG-400. The emission peak maxima do not shift and no additional peaks appear in the emission spectra in pure PEG-400. The *Φ*_F_ of pseudo-*p*-NB increases to an impressive 94% in a viscous environment in Fig. 9B and D. Comparison of the calculated S_0_, S_1_, and T_1_ rotational energy barriers in Table S33 does not explain the relative quantum yields, in contrast to literature.^107^ However, the significance of molecular motions to the excited state energy profile of the betaines was supported by excited state dynamics calculations.^86,87^ The Herzberg–Teller^108,109^ components of the absorption and fluorescence spectra of *m*-PB and pseudo-*m*-NB are calculated between 30–40%, compared to <12% for their *para*-analogues (Fig. S88). Consideration of vibrational coupling not only improves the match to experimental data, but also reproduces the trend in full-width-at-half-maximum of fluorescence spectra, relating the broad profiles of *m*-PB and pseudo-*m*-NB to emission within vibronic states (Fig. S72–S87).

## Conclusions

Our findings reveal the considerable potential of *meta*-substitution around arene cores to tune key photophysical characteristics of organic dyes. We have demonstrated that 99.9% reabsorption-free emission and Stokes shifts >10 000 cm^−1^ are accessible from betaines with a single-benzene core. Extending the design to a naphthalene core yielded a betaine with similar results (99.7% and 7601 cm^−1^ respectively). The *meta*-substitution of malonide donor and imidazolium acceptor groups was critical to the realisation of these properties, relative to *para*-substitution. Weakly solvatochromic fluorescence, acidochromism, and computational modelling of the frontier molecular orbitals revealed that intramolecular charge-transfer was only partially responsible for the large Stokes shift. An in-depth structural analysis of the excited states revealed that greater change in dipole and excited-state antiaromaticity in the *meta*-betaines were the other contributors, functioning together to increase the fluorescence wavelength (by decreasing the energy gap between the involved states). We believe that fluorophores based on *meta*-substituted donor–acceptor design with improved light absorptivity, excited state lifetime, and emission quantum yield could become additions to the toolbox of large Stokes shift fluorophores.

## Author contributions

DTH: conceptualisation, investigation, formal analysis, project administration, data curation, funding acquisition, visualisation, writing – original draft, writing – review and editing. ARK: investigation, writing – review and editing. RF: investigation, formal analysis, resources. MW: investigation, formal analysis, resources. URG: funding, resources, writing – review and editing. SE: supervision, funding acquisition, resources, writing – review and editing.

## Conflicts of interest

There are no conflicts to declare.

## Supplementary Material

SC-OLF-D6SC03405E-s001

SC-OLF-D6SC03405E-s002

## Data Availability

CCDC 2494028 (*m*-PB), 2494029 (*p*-PB), 2494030 (pseudo-*m*-NB), and 2494031 (pseudo-*p*-NB) contain the supplementary crystallographic data for this paper.^110*a–d*^ The data supporting for this article have been included as part of the supplementary information (SI). Supplementary information: compound characterisation, analytical spectroscopy, computational modelling in Fig. S1–S210, and Tables S1–S43. Raw experimental data are available at Refubium at http://dx.doi.org/10.17169/refubium-50616. See DOI: https://doi.org/10.1039/d6sc03405e.
