# Supplementary material for: Large Stokes shift fluorophores from meta-substituted zwitterions
Source: Chem Sci. 2026 Jun 17. Online ahead of print. doi: 10.1039/d6sc03405e (PMC13312548; doi:10.1039/d6sc03405e)
Supplement: SC-OLF-D6SC03405E-s001 [file SC-OLF-D6SC03405E-s001.pdf]

Supplementary Information (SI) for Chemical Science.
This journal is © The Royal Society of Chemistry 2026

Supplementary Information

"Large Stokes shift fluorophores from *meta*-zwitterions"

David T. Hogan, Alexander R. Krappe, Ralf Feyerherm, Manuela Weber, Ute Resch-Genger, Siegfried Eigler

siegfried.eigler@fu-berlin.de

Table of Contents

**Section S1**	General Considerations	...2
**Section S2**	Survey of Literature Compounds	...5
**Section S3**	Synthetic Procedures	...10
**Section S4**	Analytical Spectra of Synthesised Compounds	...29
**Section S5**	Mesomeric Betaine Nomenclature	...51
**Section S6**	X-ray Crystallography	...52
**Section S7**	Aromaticity Analysis	...61
**Section S8**	Infra-red Spectroscopy	...63
**Section S9**	2-D Nuclear Magnetic Resonance Spectroscopy	...64
**Section S10**	Electrochemistry	...83
**Section S11**	Model Compounds Photophysics	...89
**Section S12**	Concentration-dependent Photophysics	...92
**Section S13**	Computational Modelling of Electronic Excitations	...95
**Section S14**	Acidochromism	...112
**Section S15**	Solvatochromism	...119
**Section S16**	Computational Modelling of Rotational Energy Barriers	...130
**Section S17**	Fluorescence Lifetimes Fitting	...135
**Section S18**	Oxygen-dependent Photophysics	...185
**Section S19**	Viscosity-dependent Photophysics	...186
**Section S20**	Electron Paramagnetic Resonance Spectroscopy	...191
**Section S21**	SQUID Magnetometry	...196
**Section S22**	Predicted Stability of Singlet Betaines versus Triplet Diradicals	...200
**Section S23**	Computational Modelling of Ground States with Point-Charges	...207
**Section S24**	References	...210

Section S1 General Considerations

Unless specified, the following chemicals were used as received from the supplier at the listed purity:

THF from an MBraun MB-SPS 800 solvent purification system packed with Al_2O_3; EtOH (Berkel, anhydrous with added petroleum ether), CH_3CN (Fisher Chemical, ≥99%); MeOH (Fisher Scientific, analytical reagent grade); DMF (Fisher Chemical, ≥99.5% analytical reagent grade); DMSO (Fisher Chemical 99.9% analytical reagent grade); formamide (Sigma-Aldrich ≥99.0% (GC) reagent plus); PhCN (ABCR 99%); CH_2Cl_2 (BDH Chemicals, ≥99.9%, stabilised with 0.002% 2-methyl-2-butene); CyH (Fisher Scientific laboratory reagent grade); EtOAc (Fisher Chemical ≥99.8% analytical reagent grade); acetone (Th. Geyer GmbH & co. KG minimum 99.5% puriss); PEG-400 (Roth Rotipuran).

Benzaldehyde (Acros Organics, 98+% pure); glyoxal (Sigma-Aldrich 40% solution in water); ammonia (Roth 25% solution in water); CH_3I (Sigma-Aldrich copper stabilised 99% reagent plus); K_2CO_3 (Roth ≥99% pure); $MeNH_2$ (40% solution in water); ammonium carbonate (Fisher Chemical analytical reagent grade); NaOH (Fisher Chemical analytical reagent grade 99.22% pure); malononitrile (Merck KGaA ≥98% for synthesis); $Pd(PPh_3)_4$ (Roth ≥98% pure); NaH (TCI 60% dispersion in paraffin oil); *n*-BuLi (Sigmal-Aldrich 2.5 M in hexanes); bromine (Thermo Fisher 99.6% for synthesis); PPh_3 (Thermo Fisher 99% pure); HCl (Fisher Chemical 37% solution in water reagent grade); 2,7-dihdroxynaphthalene (Thermo Scientific 97%).

Thin-layer chromatography was performed using ALUGRAM® Xtra SIL G/UV_{254} plates from Macherey-Nagel. Visualisation was performed by irradiation with UV light of wavelengths λ = 254 or 365 nm. Column chromatography was performed using silica gel Silica 60 M (0.04 - 0.063 mm) from Macherey-Nagel.

Melting points were uncorrected and obtained using a Büchi 510 melting point apparatus.

^1H NMR and ^{13}C {^1H} NMR were collected at the specified frequencies on the spectra (see below) using the following instruments: Bruker Biospin AVANCE700 (^1H at 700 MHz, ^{13}C at 176 MHz), JEOL ECZ600 (^1H at 600 MHz, ^{13}C at 151 MHz), Bruker Biospin AVANCE500 (^1H at 500 MHz, ^{13}C at 126 MHz), JEOL ECX400 (^1H at 400 MHz, ^{13}C at 101 MHz). All experiments were conducted at 20 °C. ^1H NMR and $^{13}C\{^1H\}$ NMR spectra were referenced against the residual solvent signals from $CHCl_3$ in $CDCl_3$ (^1H δ = 7.26, ^{13}C δ = 77.2), or DMSO-d_5H in DMSO-d_6 (^1H δ = 2.50, ^{13}C δ = 39.5).[1]

High-resolution mass spectrometric (HR MS) measurements were collected using a Waters Autospec Premier with electron impact ionisation. All chemical formula confirmations were made with less than 5 ppm difference between calculated and observed masses.

Elemental analysis for C, H, N, and S determination was carried out on a VARIO EL from Elementar.

Suitable crystals were selected and mounted on a glass loop using Paratone. Diffraction experiments were performed on a Bruker Smart APEX-II CCD diffractometer equipped with rotating anode (Mo Kα, λ = 0.71069 Å) and an APEX-II CCD detector. Diffraction spots were integrated and scaled with SAINT and the space group was determined with XPREP. Using Olex2,[2] the structure was solved with the ShelXT[3] structure solution program using Intrinsic Phasing and refined with the ShelXL[4] refinement package using Least Squares minimisation.

Infra-red spectroscopy was performed using the attenuated total reflectance module of an FT-IR Spectrum Two with $LiTaO_3$ detector from Perkin Elmer, with a 0.5 cm^{-1} resolution.

UV-visible absorbance spectroscopy was performed at 20 °C on a Lambda 365 spectrometer from Perkin Elmer in 10 mm pathlength quartz cuvettes from Helma Analytics.

UV-visible fluorescence spectroscopy was performed at 20 °C on a FL 6500 spectrometer from Perkin Elmer in 10 × 10 mm pathlength quartz cuvettes from Hellma Analytics GmbH.

Fluorescence quantum yields were determined at using a relative method[5] against the standards tryptophan in water (Φ=0.15)[6] for compound **17**, tyrosine in water (Φ=0.13)[7] for compound **3**, quinine sulfate in 0.1 M sulfuric acid (Φ=0.52)[6] for compound ***p*-PB**, or cresyl violet in methanol (Φ=0.57)[8] for compounds ***m*-PB** and **pseudo-*m*-NB**. The fluorescence quantum yield of **pseudo-*p*-NB** was measured using a Hamamatsu Quantaurus-QY C11347-11 integrating sphere. Fluorescence quantum yields for PEG-400/CH_3CN at different viscosities at 20 °C were measured with the relative method, using the individual compounds dissolved in pure CH_3CN as standards.

Refractive indices for CH_3CN/PEG-400 mixtures were acquired at 20 °C using an analog Abbe refractometer.

Fluorescence lifetimes were determined on an Edinburgh Instruments FLS 920 equipped with a Hamamatsu R3809U-50 Multi-Channel Plate (MCP) detector, Czerny-Turner double monochromators and a supercontinuum laser excitation source. All the measurements were performed at 20 °C using 10 × 10 mm quartz cuvettes from Hellma Analytics GmbH. Following each measurement, the instrument response function (IRF) was measured using a Ludox solution. The lifetime measurements were analyzed with Edinburgh Instruments FAST Software and fitted with a mono-, bi-, or tri-exponential reconvolution fit.

Electrochemistry was performed with a Gamry Instruments Interface 1010B potentiostat in a single compartment glass cell with a three-electrode setup: a 3 mm glassy carbon planar button working electrode, a Pt wire counter electrode, and a Ag/Ag^+ non-aqueous reference electrode containing a silver wire immersed in 0.01 M $AgNO_3$ + 0.1 M $TBAPF_6$ filling solution separated by a thirsty Vycor frit. Solutions contained ~0.1 M $TBAPF_6$ supporting electrolyte, were bubbled with nitrogen before measurement and retained under nitrogen during measurement, and were internally referenced to ferrocene/ferrocenium (Fc/Fc^+) couple immediately post-scan. Cyclic voltammetry experiments were performed at 20 °C, scanned at a rate of 500 $mV \cdot s^{-1}$, with 3 mV step size, 5 sec conditioning time at 0.0 V vs the reference electrode potential. Differential pulse voltammetry experiments were performed at 20 °C, scanned with 5 mV step size, 0.25 sec sample period, 0.1 sec pulse time, 50 mV pulse size, 0.02 sec integration time, and 5 sec equilibration time before measurement.

Continuous wave X-band EPR was performed in 4 mm quartz tubes at 20 °C using a continuous wave Magnettech miniscope MS-5000 from Freiberg Instruments. Solutions were purged and sealed with nitrogen prior to measurement.

Superconducting quantum interference device (SQUID) magnetometry with a Quantum Design MPMS vibrating sample magnetometer (VSM) under an applied field of 5 kOe was used to determine the molar magnetic susceptibility of powder samples held in plastic capsules between 5 K and 400 K.

Gaussian 16 rev C.02[9] was used in calculations regarding the stability of singlet and triplet states. Geometry optimizations were carried out using the specified density functional and basis set (refer to tables in Sections S8 as needed), either in gas phase or with acetonitrile implicitly modelled with the CPCM[10–12] with D3 dispersion.[13] Optimised tructures were checked for imaginary frequencies to ensure that they had converged to minima. Calculations were performed on a high-performance computing cluster named CURTA at the Freie Universität Berlin[14]

Orca 6.1.0[15–23] was used for all other calculations. Geometry optimizations were generally carried out in the gas phase using the desired functional with the def2-SVP basis set, then the desired property was calculated with the def2-TZVP[24,25] basis set in acetonitrile using the CPCM. These calculations were performed on a local server using the WebMO graphical user interface[26] or via high-performance computing cluster.

Rotational energy barriers were calculated using the relaxed surface potential scan of ORCA, using previously optimized geometries at the lowest singlet, first excited singlet, and lowest triplet states.

Differences in solvated S_0 and S_1 geometries at relaxed and Frank-Condon geometries were determined at the CAM-B3LYP/def2-TZVPD level using SMD[27] solvation, D4 dispersion,[28] and with the DRACO[29] scheme. This scheme is currently only available for single-point calculations.

Time-dependent DFT employed the Tamm-Dancoff approximation. Geometry optimization of singlet states used the rijcosx approximation.[30] Vibrational coupling from the Herzberg-Teller effect was added into excited state dynamics (ESD) calculations of absorption and fluorescence spectra using the *doht true* keywords with a Lorentzian lineshape and linewidth of 600. Spin-orbit coupling (SOC) constants were calculated in a TD-DFT input for both singlets and triplets using the *dosoc true* keyword.[31,32]

NMR calculations were done with the pcSseg-2[33] basis set in acetonitrile modelled with the CPCM, using the GIAO function[34–39].

Multiwfn 3.8 [40,41] was used for many applications: to calculate, NICS(1)$_{zz}$, HOMA[42,43] / HOMAc[44] / HOMER[45] / FLU[46] indices, Laplacian bond orders,[47] to place ghost atoms for the NICS(1) calculations; generating cube files for triplet spin density, biorthogonalization of α and β SOMOs, electron-hole (medium grid), overlap function S_r (medium grid), Coulomb integrals (low grid), charge density difference maps (medium grid), transition electric dipole moment, D index, and *t* index.[48] The *Tprint 1E-8* keyword was used in the Orca TD-DFT input files to print more orbital contributions, which was necessary for acquiring the above data from Multiwfn.

Avogadro 1.2.0 [49] was used for visualisation of all molecules and surfaces.

Coordinates and output files for all calculations can be found in the data repository, referenced at the end of the manuscript

Section S2 Survey of Literature Compounds

Table S1. Some small fluorophores with large Stokes shifts.

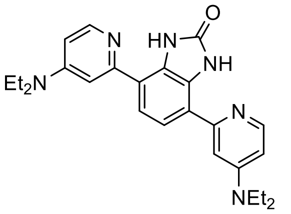<br>$\lambda_{(abs,\ CH2Cl2)}$ (nm) = 312<br>$\lambda_{(em,\ CH2Cl2)}$ (nm) = 509<br>Stokes shift (cm^{-1}) = **12 405**<br>10.1039/D4CC03389B	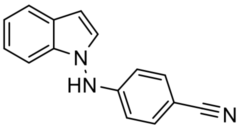<br>$\lambda_{(abs,\ dioxane)}$ (nm) = 268<br>$\lambda_{(em,\ dioxane)}$ (nm) = 515<br>Stokes shift (cm^{-1}) = **18 100**<br>10.1002/chem.201700566	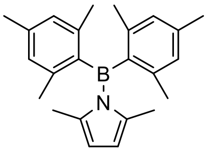<br>$\lambda_{(abs,\ CH2Cl2)}$ (nm) = 286<br>$\lambda_{(em,\ CH2Cl2)}$ (nm) = 662<br>Stokes shift (cm^{-1}) = **19 860**<br>10.1002/poc.610020109

Table S2. Some single-benzene fluorophores (SBFs) with large Stokes shifts.

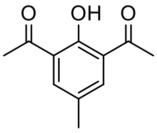<br>$\lambda_{(abs,\ MeOH)}$ (nm) = 350<br>$\lambda_{(em,\ MeOH)}$ (nm) = 525<br>Stokes shift (cm^{-1}) = **9 730**<br>10.1002/chem.202501178	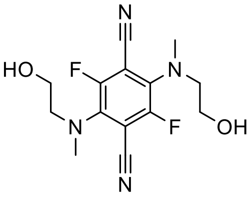<br>$\lambda_{(abs,\ CH2Cl2)}$ (nm) = 414<br>$\lambda_{(em,\ CH2Cl2)}$ (nm) = 546<br>Stokes shift (cm^{-1}) = **5 840**<br>10.1016/j.molstruc.2024.139340	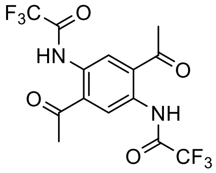<br>$\lambda_{(abs,\ CHCl3)}$ (nm) = 381<br>$\lambda_{(em,\ CHCl3)}$ (nm) = 584<br>Stokes shift (cm^{-1}) = **9 060**<br>10.1002/anie.202302107
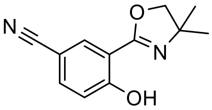<br>$\lambda_{(abs,\ CyH)}$ (nm) = 306<br>$\lambda_{(em,\ CyH)}$ (nm) = 468<br>Stokes shift (cm^{-1}) = **11 310**<br>10.1039/D0TC00776E	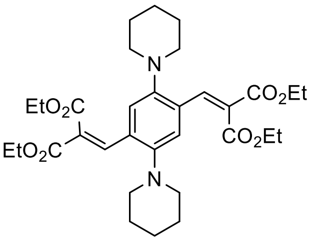<br>$\lambda_{(abs,\ CyH)}$ (nm) = 425<br>$\lambda_{(em,\ CyH)}$ (nm) = 656<br>Stokes shift (cm^{-1}) = **8 285**<br>10.1002/anie.200900963	

Table S3. Some disubstituted single-benzene fluorophores (SBFs) with large Stokes shifts.

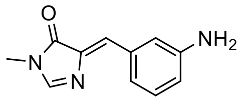

$\lambda_{(abs,\ CyH)}$ (nm) = 378
$\lambda_{(em,\ CyH)}$ (nm) = 578
Stokes shift (cm^{-1}) = **9 154**
10.1039/B717714C

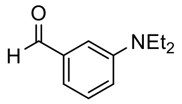

$\lambda_{(abs,\ MeOH)}$ (nm) = 382
$\lambda_{(em,\ MeOH)}$ (nm) = 600
Stokes shift (cm^{-1}) = **9 511**
10.1021/acs.jpcc.9b08524

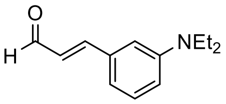

$\lambda_{(abs,\ CH3CN)}$ (nm) = 400
$\lambda_{(em,\ CH3CN)}$ (nm) = 600
Stokes shift (cm^{-1}) = **8 333**
10.1021/acs.jpcc.0c09106

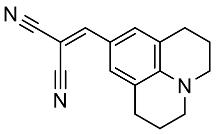

$\lambda_{(abs,\ CH3CN)}$ (nm) = 460
$\lambda_{(em,\ CH3CN)}$ (nm) = 505
Stokes shift (cm^{-1}) = **1 937**
10.1021/acs.jpca.5b12612

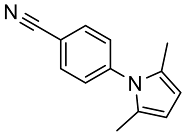

$\lambda_{(abs,\ CH2Cl2)}$ (nm) = 275
$\lambda_{(em,\ CH2Cl2)}$ (nm) = 530
Stokes shift (cm^{-1}) = **17 500**
10.1021/jp981306j

Table S4. Some non-fluorescent zwitterions/betaines/mesoionic compounds.
(zwitterions formed by attachment of pendant ionic groups are not considered here)

Generally: D-A Stenhouse adducts

and derivatives of Reichardt's Betaine-30.

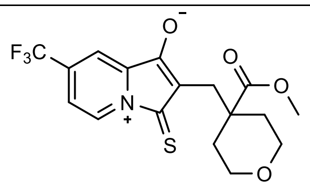

$\lambda_{(abs,\ DMSO)}$ (nm) = 602
$\lambda_{(em)}$ (nm) = ---
Stokes shift (cm^{-1}) = ---
10.1021/acs.joc.4c00812

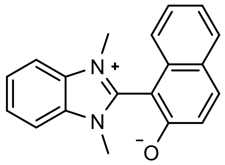

$\lambda_{(abs,\ DMSO)}$ (nm) = 415
$\lambda_{(em)}$ (nm) = ---
Stokes shift (cm^{-1}) = ---
10.1021/acs.joc.8b00282

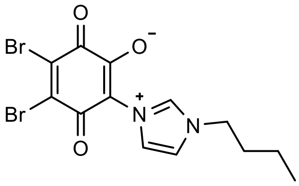

$\lambda_{(abs)}$ (nm) = yellow
$\lambda_{(em)}$ (nm) = ---
Stokes shift (cm^{-1}) = ---
10.3762/bjoc.8.42

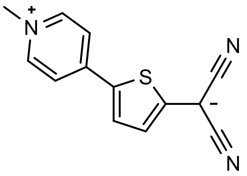

$\lambda_{(abs,\ CH3CN)}$ (nm) = 545
$\lambda_{(em)}$ (nm) = ---
Stokes shift (cm^{-1}) = ---
10.1021/jacs.5b12457

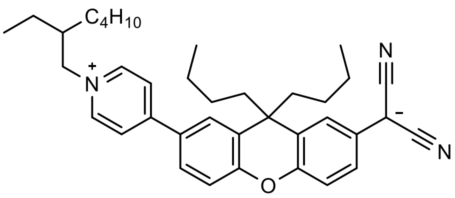

$\lambda_{(abs,\ DMF)}$ (nm) = 500
$\lambda_{(em)}$ (nm) = ---
Stokes shift (cm^{-1}) = ---
10.1039/D2MA00721E

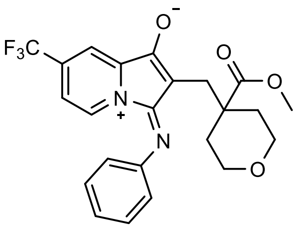

$\lambda_{(abs,\ MeOH)}$ (nm) = 590
$\lambda_{(em)}$ (nm) = ---
Stokes shift (cm^{-1}) = ---
10.1021/acs.joc.1c02630

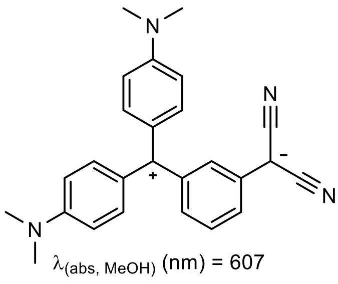

$\lambda_{(abs,\ MeOH)}$ (nm) = 607
$\lambda_{(em)}$ (nm) = ---
Stokes shift (cm^{-1}) = ---
10.1246/cl.2003.322

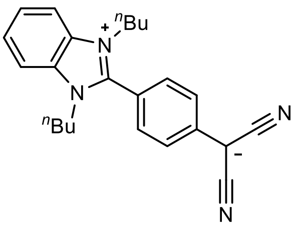

$\lambda_{(abs,\ CH3CN)}$ (nm) = 434
$\lambda_{(em)}$ (nm) = ---
Stokes shift (cm^{-1}) = ---
10.1021/jacs.8b04320

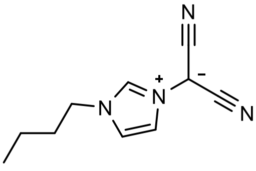

$\lambda_{(abs,solid)}$ (nm) = yellow
$\lambda_{(em)}$ (nm) = ---
Stokes shift (cm^{-1}) = ---
10.1002/ejoc.202400163

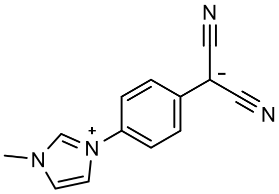

$\lambda_{(abs,\ solid)}$ (nm) = yellow
$\lambda_{(em)}$ (nm) = ---
Stokes shift (cm^{-1}) = ---
Guo, Lijuan; Hu, Xiaojian; Zeng, Ming; Cui, Xiaoying
Jingxi Huagong Zhongjianti 2012, *42*, 64.

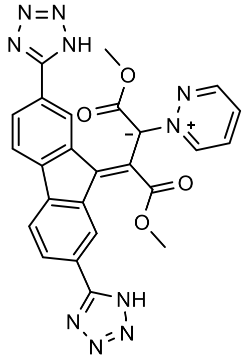

$\lambda_{(abs,\ CH2Cl2)}$ (nm) = 521
$\lambda_{(em)}$ (nm) = --
Stokes shift (cm^{-1}) =--
10.1016/j.jphotochem.2018.04.040

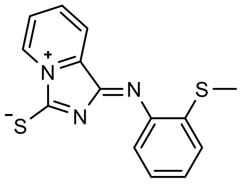

$\lambda_{(abs,\ CH3CN)}$ (nm) = 510
$\lambda_{(em)}$ (nm) = --
Stokes shift (cm^{-1}) = --
10.1039/C1DT11435B

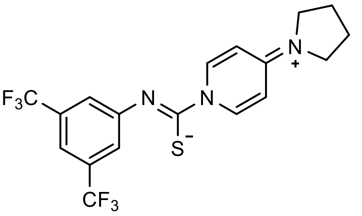

$\lambda_{(abs,\ DMSO)}$ (nm) = yellow
$\lambda_{(em)}$ (nm) = --
Stokes shift (cm^{-1}) = --
10.1039/D4QO01390E

Table S5. Some fluorescent zwitterions/betaines/mesoionic compounds.
(zwitterions formed by attachment of pendant ionic groups are not considered here)

Generally: merocyanines from photochromic spiropyrans, rhodamines, and squaraine dyes are characterised by small Stokes shifts.

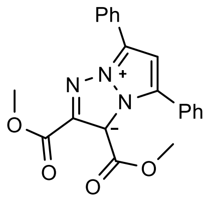

$\lambda_{(abs,\ CHCl3)}$ (nm) = 370
$\lambda_{(em,\ CHCl3)}$ (nm) = 480
Stokes shift (cm^{-1}) = **6 194**
10.1039/C4CC03175J

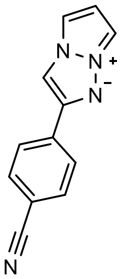

$\lambda_{(abs,\ CH2Cl2)}$ (nm) = 381
$\lambda_{(em,\ CH2Cl2)}$ (nm) = 509
Stokes shift (cm^{-1}) = **6 600**
10.1021/ja203917r

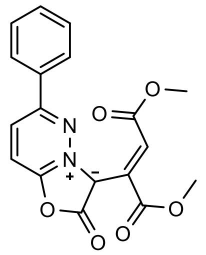

$\lambda_{(abs,\ EtOH)}$ (nm) = 447
$\lambda_{(em,\ EtOH)}$ (nm) = 511
Stokes shift (cm^{-1}) = **2 802**
Revue Roumaine de Chimie, 2004, 49(11), 905–910

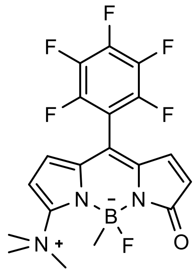

$\lambda_{(abs,\ EtOH)}$ (nm) = 405
$\lambda_{(em,\ EtOH)}$ (nm) = 461
Stokes shift (cm^{-1}) = **3 000**
10.1039/C6CC09325F

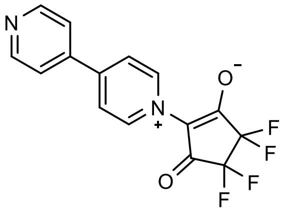

$\lambda_{(abs,\ PhH)}$ (nm) = 386
$\lambda_{(em,\ PhH)}$ (nm) = 480
Stokes shift (cm^{-1}) = **5 073**
10.1002/anie.201704832

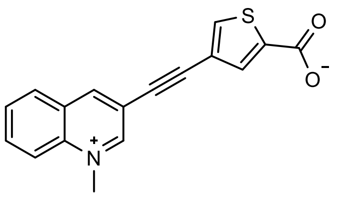

$\lambda_{(abs)}$ (nm) = --
$\lambda_{(em,\ THF)}$ (nm) = 480
Stokes shift (cm^{-1}) = --
10.1016/j.tet.2017.04.031

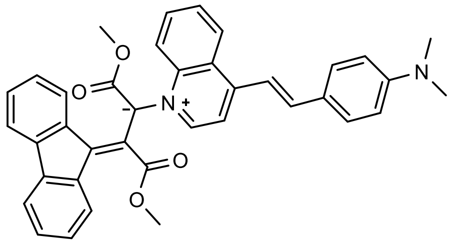

$\lambda_{(abs,\ PMMA)}$ (nm) = 587
$\lambda_{(em,\ PMMA)}$ (nm) = 645
Stokes shift (cm^{-1}) = **1 532**
10.1039/b905956c

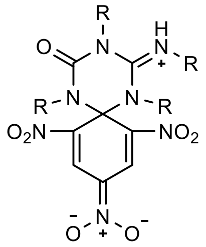

$\lambda_{(abs,\ CH2Cl2)}$ (nm) = 526
$\lambda_{(em,\ CH2Cl2)}$ (nm) = 561
Stokes shift (cm^{-1}) = **1 186**
10.1002/ejoc.200400260

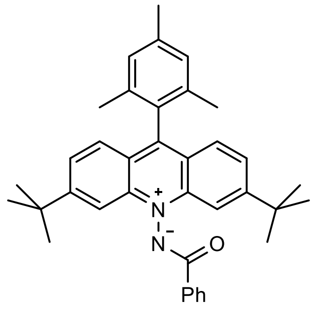

$\lambda_{(abs,\ CH3CN)}$ (nm) = 420
$\lambda_{(em,\ CH3CN)}$ (nm) = 490
Stokes shift (cm^{-1}) = **3 401**
10.1002/anie.202404890

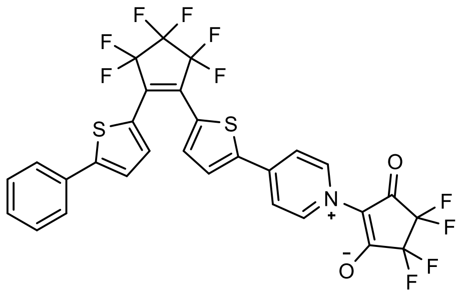

$\lambda_{(abs,\ EtOAc)}$ (nm) = 395
$\lambda_{(em,\ EtOAc)}$ (nm) = 490
Stokes shift (cm^{-1}) = **4 908**
10.1002/chem.202403076

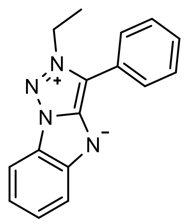

$\lambda_{(abs,\ MeOH)}$ (nm) = 358
$\lambda_{(em,\ MeOH)}$ (nm) = 483
Stokes shift (cm^{-1}) = **7 229**
10.1039/C8NJ04070B

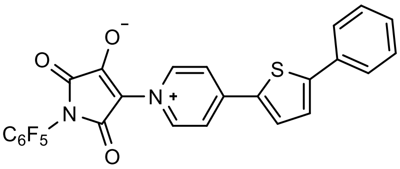

$\lambda_{(abs,\ CH2Cl2)}$ (nm) = 443
$\lambda_{(em,\ CH2Cl2)}$ (nm) = 524
Stokes shift (cm^{-1}) = **3 490**
10.1016/j.dyepig.2023.111291

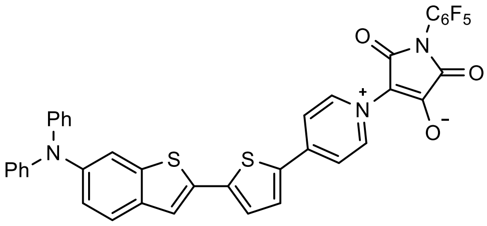

$\lambda_{(abs,\ CH2Cl2)}$ (nm) = 490
$\lambda_{(em,\ CH2Cl2)}$ (nm) = 816
Stokes shift (cm^{-1}) = **8 154**
10.1002/cptc.202400086

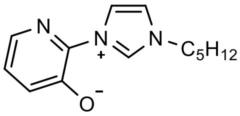

$\lambda_{(abs,\ solid)}$ (nm) = 397
$\lambda_{(em,\ solid)}$ (nm) = 424
Stokes shift (cm^{-1}) = **1 604**
10.1002/ejoc.201700943

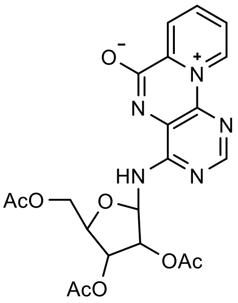

$\lambda_{(abs,\ H20)}$ (nm) = 425
$\lambda_{(em,\ H20)}$ (nm) = 528
Stokes shift (cm^{-1}) = **4 590**
10.1030/P29890001691

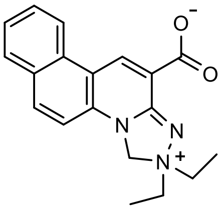

$\lambda_{(abs,\ DMF)}$ (nm) = 361
$\lambda_{(em,\ DMF)}$ (nm) = 463
Stokes shift (cm^{-1}) = **6 102**
10.1016/j.dyepig.2018.09.005

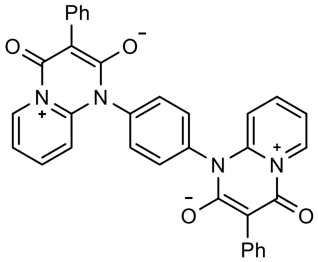

$\lambda_{(abs,\ solid)}$ (nm) = yellow
$\lambda_{(em,\ solid)}$ (nm) = yellow
Stokes shift (cm^{-1}) = --
10.1107/S0108270109018952

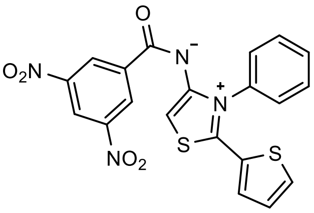

$\lambda_{(abs,\ CH3CN)}$ (nm) = 420
$\lambda_{(em,\ CH3CN)}$ (nm) = 534
Stokes shift (cm^{-1}) = **5 083**
10.1002/ejoc.2021007944803

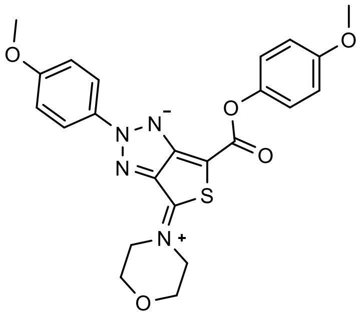

$\lambda_{(abs,\ CHCl3)}$ (nm) = 506
$\lambda_{(em,\ CHCl3)}$ (nm) = 591
Stokes shift (cm^{-1}) = **2 842**
10.1016/j.dyepig.2021.109777

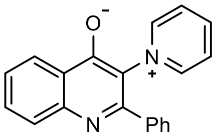

$\lambda_{(abs,\ solid)}$ (nm) = yellow
$\lambda_{(em,\ solid)}$ (nm) = 563
Stokes shift (cm^{-1}) = --
10.1055/a-2556-3115

Section S3 Synthetic Procedures

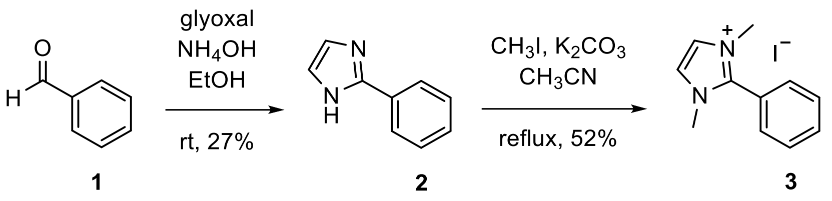

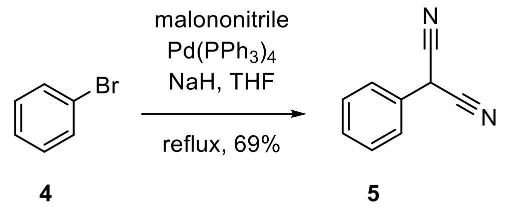

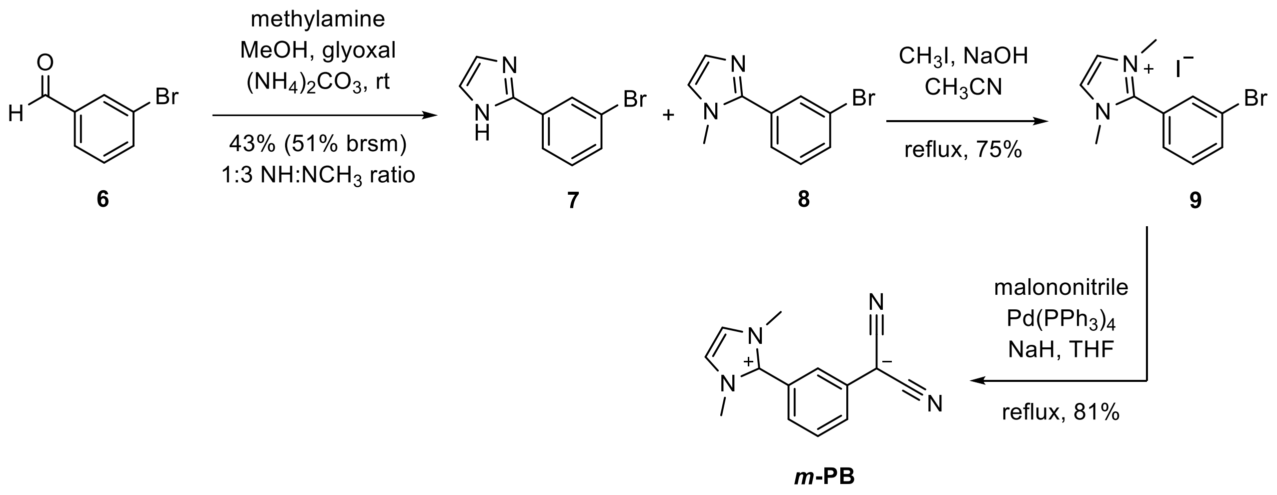

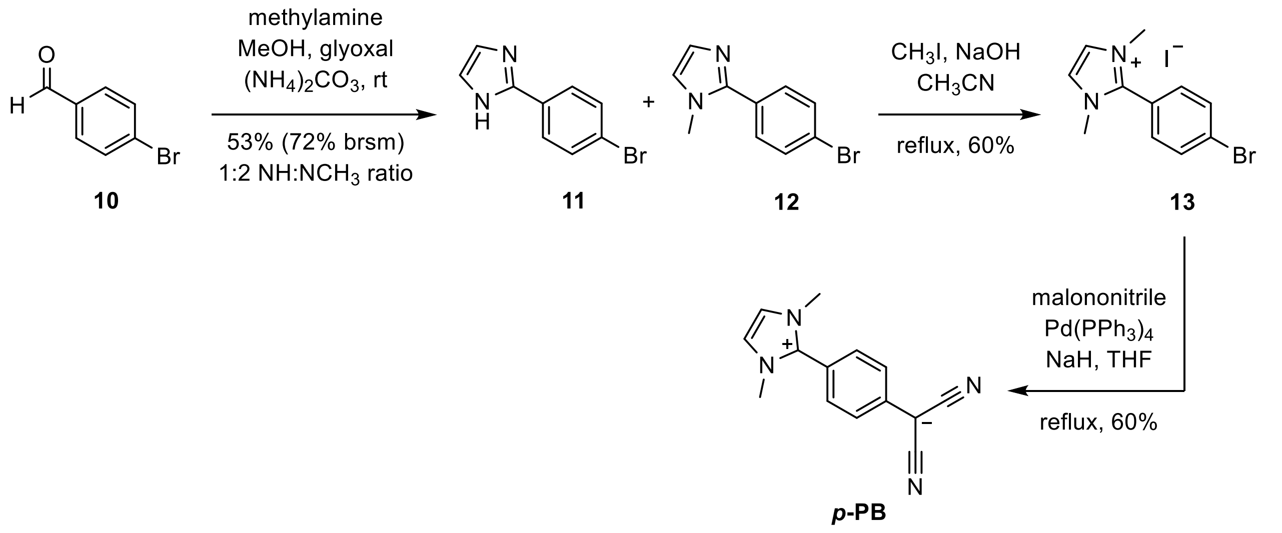

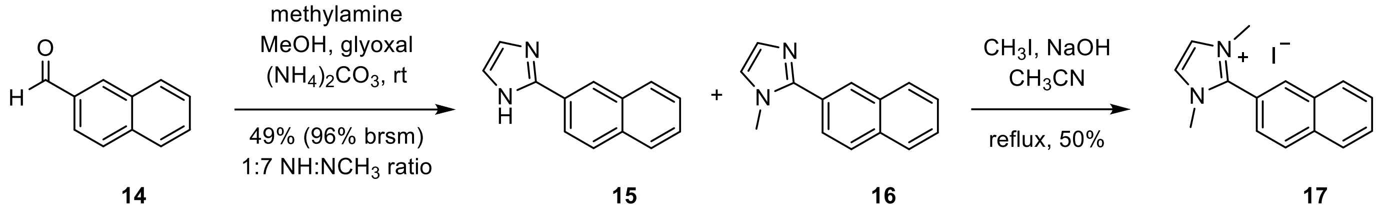
methylamine
MeOH, glyoxal
$(NH_4)_2CO_3$, rt
49% (96% brsm)
1:7 NH:NCH_3 ratio
CH_3I, NaOH
CH_3CN
reflux, 50%
14
15
16
17

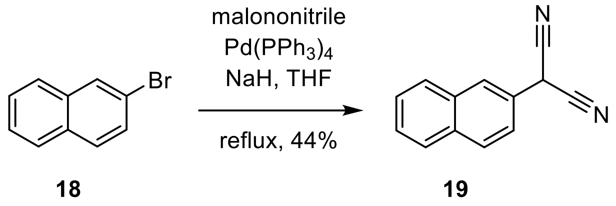
malononitrile
$Pd(PPh_3)_4$
NaH, THF
reflux, 44%
18
19

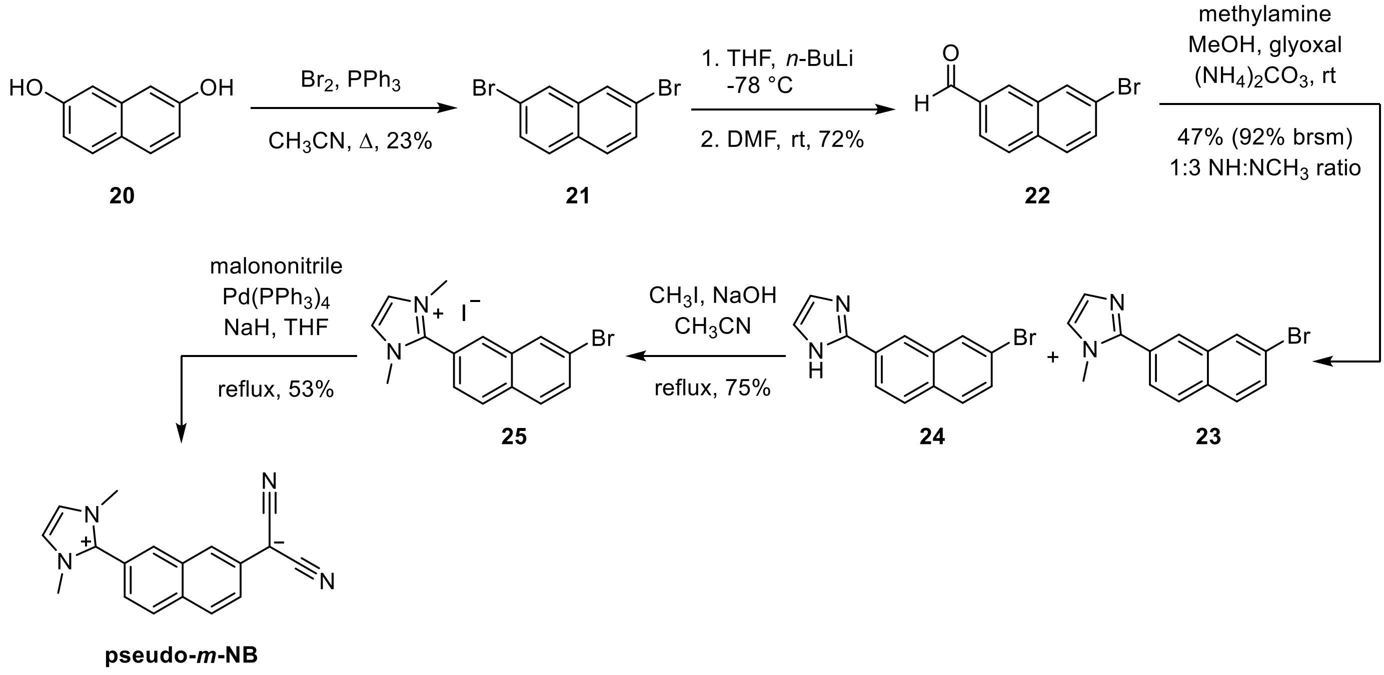
Br_2, PPh_3
CH_3CN, Δ, 23%
1. THF, *n*-BuLi -78 °C
2. DMF, rt, 72%
methylamine
MeOH, glyoxal
$(NH_4)_2CO_3$, rt
47% (92% brsm)
1:3 NH:NCH_3 ratio
CH_3I, NaOH
CH_3CN
reflux, 75%
malononitrile
$Pd(PPh_3)_4$
NaH, THF
reflux, 53%
20
21
22
23
24
25
pseudo-*m*-NB

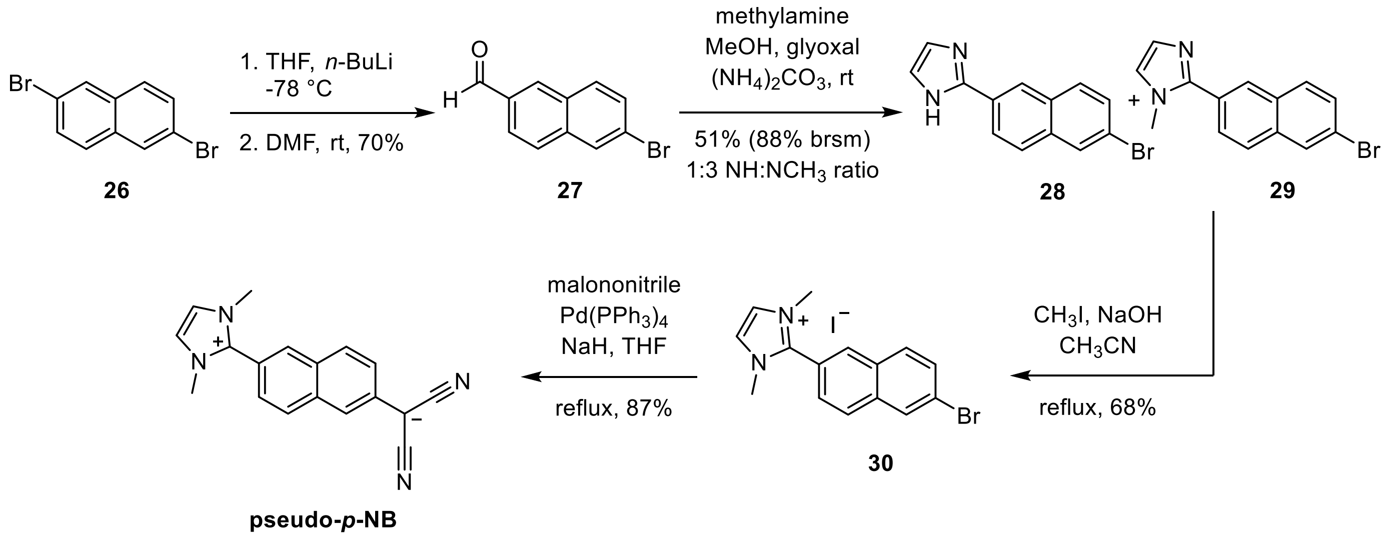
1. THF, *n*-BuLi -78 °C
2. DMF, rt, 70%
methylamine
MeOH, glyoxal
$(NH_4)_2CO_3$, rt
51% (88% brsm)
1:3 NH:NCH_3 ratio
CH_3I, NaOH
CH_3CN
reflux, 68%
malononitrile
$Pd(PPh_3)_4$
NaH, THF
reflux, 87%
26
27
28
29
30
pseudo-*p*-NB

2-Phenyl-1-*H*-imidazole **2**

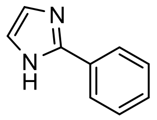

2

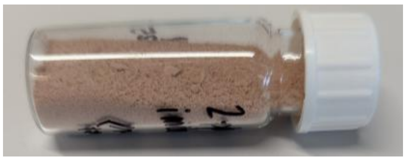

Into a 500 mL RBF were added a large magnetic stirbar benzaldehyde (5.1 mL, 50 mmol, 1 eq.), glyoxal (6.3 mL of 40% solution in water, 61 mmol, 1.2 eq), and EtOH (175 mL). Ammonia solution (80 mL of 25% solution in water, 510 mmol, 10 eq.) was added and the solution was capped and stirred at room temperature for 3 days. The volatiles were removed under reduced pressures, the molasses-like syrup was dissolved in CH_2Cl_2, adsorbed to silica, and loaded onto a 11 cm tall × 5.5 cm wide silica column packed in CH_2Cl_2. Gradient elution: CH_2Cl_2 (~500 mL), 1% MeOH/CH_2Cl_2 (~750 mL), 2% MeOH/CH_2Cl_2 (~750 mL), 3% MeOH/CH_2Cl_2 (~750 mL). The desired imidazole co-eluted with many impurities, so all eluate which contained desired imidazole by TLC was collected and concentrated under reduced pressures into a brown solid. This was recrystallised twice from boiling 50% $CHCl_3$/CyH (~50 mL), allowed to cool to room temperature for 30 minutes then in the fridge for 30 minutes, followed by isolation of the solids by suction filtration and washing with some chilled 50% $CHCl_3$/CyH to yield 2-phenyl-1-*H*-imidazole **2** as blush-coloured microcrystalline needles (0.1933 g, 27% yield): characterisation data conforms with literature.[50]

1,3-Dimethyl-2-phenylimidazolium iodide **3**

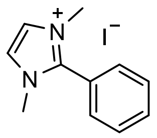

3

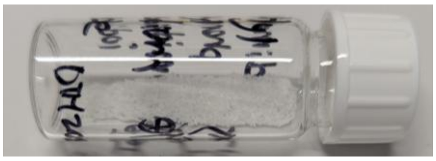

Into a 25 mL RBF with magnetic stirbar were added **2** (0.501 g, 3.47 mmol, 1 eq.), K_2CO_3 (0.960 g, 6.94 mmol, 2 eq.), CH_3CN (10 mL) followed by CH_3I (1.08 mL, 17.3 mmol, 5 eq.). The flask was equipped with a water condenser and the peachy-tan suspension was refluxed and stirred on a 85-90 °C oil bath. Within 1 hour the colour of the precipitates had lightened and after 4 hours TLC A indicated complete reaction. The mixture was cooled to room temperature and filtered by gravity and the filter cake was washed with CH_3CN (10 mL). The solvent was removed under reduced pressures, the white solid was boiled with CH_2Cl_2 (15 mL) and hot gravity filtered. This process was repeated 5 × total. The combined filtrate was concentrated under reduced pressures into a yellow solid, which was recrystallised with minimal boiling CH_3CN, allowed to sit at room temperature for 1 hour then in the freezer for 2 hours. Suction filtration and washing the solid with CH_3CN yielded white crystals of 1,3-dimethyl-2-phenylimidazolium iodide **3** (0.549 g, 53% yield): characterisation data conforms with literature.[51]

IR (solid, cm^{-1}): $\tilde{\nu}$= 3050, 1520, 1250, 778, 690; UV/Vis (CH_3CN, nm): λ_{max} (ε)= 244 (22 000); fluorescence (CH_3CN, nm): λ_{ex}= 345; λ_{em} (Φ)= 315 (0.02 ± 0.01).

2-Phenylmalononitrile **5**

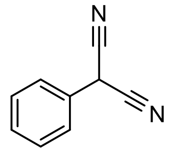

5

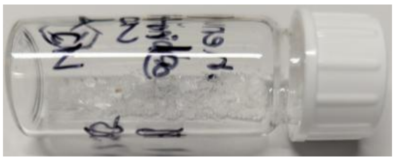

Adapted from literature.[52] A 50 mL Schlenk flask and small magnetic stirbar were heated with a heatgun under vacuum to drive off water, cooled to room temp and backfilled with nitrogen. Dried THF (9 mL) were added to the flask, bubbled with nitrogen and stirred for 10 mins, malononitrile (0.441 g, 6.66 mmol, 2 eq.), then $Pd(PPh_3)_4$ (0.115 g, 99.9 µmol, 0.03 eq.) were added. To the orange-yellow solution was added portion-wise NaH (0.401 g of 60% dispersion in paraffin oil, 16.6 mmol, 3 eq.) to minimise frothing. The cloudy yellow mixture was stirred at room temperature under nitrogen for 5 mins after the frothing subsided, then bromobenzene (0.35 mL, 3.33 mmol, 1 eq.) were added by syringe. The resulting custard-yellow mixture was equipped with a pre nitrogen-flushed water condenser and refluxed and stirred on a 90 °C oil bath. It quickly generated cream-coloured precipitates. After 20 hours the pale orange turbid mixture was removed from the oil bath, allowed to cool to room temperature, and the solvent was removed under reduced pressures. The mixture was diluted with water (10 mL) and CH_2Cl_2 (10 mL), then the aqueous layer was acidified to pH ~2 (by paper) using concentrated HCl. The layers were separated, the aqueous layer was extracted using CH_2Cl_2 (3 x 10 mL), the organics were combined and brine washed, dried over sodium sulfate then concentrated under reduced pressures into an orange oil. It was dissolved in CH_2Cl_2, adsorbed to silica, and loaded onto a 10 cm tall × 2 cm wide silica column in CyH. Gradient elution: CyH (~50 mL) to elute paraffin oil, 5% EtOAc/CyH ~150 mL), 10% EtOAc (~100 mL). Collection of the 5% and 10 % EtOAc/CyH eluate and concentration under reduced pressures into a dark solid. The solid was twice recrystallised from boiling minimal diethyl ether, allowed to sit at room temperature for 15 minutes then in the fridge for 1 hour. Suction filtration yielded snowflake-like white crystals of 2-phenylmalononitrile **5** (0.328 g, 69%): characterisation data conforms with literature.[53]

Mixture of 2-(3-bromophenyl)-1-*H*-imidazole **7**
and
2-(3-bromophenyl)-1-methylimidazole **8**

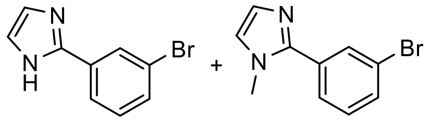

7 **8**

Adapted from literature.[54] Into a 50 mL RBF with magnetic stirbar were added melted 3-bromobenzaldeyhde (3.011 g, 16.3 mmol, 1 eq.) and MeOH (15 mL), then to the solution was added methylamine (1.69 mL of 40% solution in water, 17.1 mmol, 1.05 eq.) and the solution was stirred for 15 minutes at room temperature. Then glyoxal (1.86 mL of 40% solution in water, 16.3 mmol, 1 eq.) and freshly ground ammonium carbonate (1.170 g, 12.2 mmol, 0.75 eq.) were added and mixture slowly became golden yellow, released gases, and all solids slowly dissolved. The mixture was stirred at room temperature for 20 hours, after which time the mixture was concentrated under reduced pressures into a biphasic mixture. Most of the colourless water was carefully pipetted away, then EtOAc (~10 mL) was added to the mixture which was then dried over sodium sulfate. The solution was adsorbed to silica, loaded onto a 10 cm tall × 3.2 cm wide silica column packed in 50% CH_2Cl_2/CyH. Gradient elution: 50% CH_2Cl_2/CyH (~350 mL), CH_2Cl_2 (~100 mL), 3% MeOH/CH_2Cl_2 (~400 mL). Unreacted 3-bromobenzaldeyhde **6** (0.507 g, 17% recovery) eluted with 50% CH_2Cl_2/CyH. The mixed imidazoles eluted with 3% MeOH/CH_2Cl_2 following a yellow-coloured band. Concentration of the eluate under reduced pressures yielded a ~1:3 mixture of 2-(3-bromophenyl)-1-*H*-imidazole **7** and 2-(3-bromophenyl)-1-methylimidazole **8** as an amber liquid which partially solidified into a beige residue (1.633 g, 51% yield based on recovered starting material). The substance was used without further purification.

R_f=0.43 (10% MeOH/CH_2Cl_2), 0.47 (10%MeOH/CH_2Cl_2);^{1}H NMR (400 MHz, DMSO-d_6, ppm): diagnostic signals for **7** δ= 8.13 (t, J = 1.8 Hz, rel. int. 1.00), 7.93 (m, rel. int. 1.44), 7.52 (m, rel. int. 1.69); diagnostic signals for **8** δ= 7.87 (t, J= 1.7 Hz, rel. int. 3.77), 7.70 (m, rel. int. 4.08), 7.61 (m, rel. int. 4.11).

2-(3-Bromophenyl)-1,3-dimethylimidazolium iodide **9**

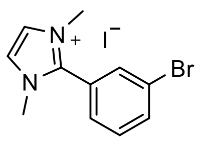

9

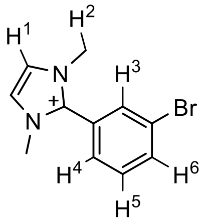

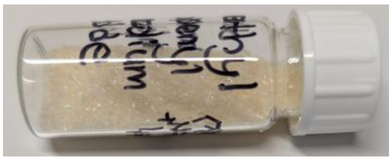

Into a 100 mL RBF with mixed **7** and **8** (1.65 g, ~6.95 mmol, 1 eq.) were added a magnetic stirbar, CH_3CN (16 mL) then NaOH pieces (0.278 g, 6.95 mmol, 1 eq.). The mixture was stirred at room temperature for 5 minutes then CH_3I (2.16 mL) were added, the flask was equipped with a water condenser and the mixture was stirred and refluxed on an 84 °C oil bath for 2 hours. After this time, the reaction was cooled to room temperature, remaining NaOH chunks were removed with tweezers, and the solvent was removed under reduced pressures. The beige residue was recrystallised in minimal boiling EtOH, allowed to cool at room temperature for 15 minutes, then in the freezer for 30 minutes and dried in air to yield 2-(3-bromophenyl)-1,3-dimethylimidazolium iodide **9** as a crystalline beige solid (1.970 g, 75% yield). The solid can also be recrystallised from minimal boiling CH_3CN, although EtOH more efficiently removes coloured impurities.

R_f=0.11 (10% MeOH/CH_2Cl_2);^{1}H NMR (400 MHz, DMSO-d_6, ppm): δ= 8.07 (t, *J* = 1.8 Hz, 1H^3), 7.99 – 7.95 (m, 1H^6), 7.88 (s, 2H^1), 7.80 – 7.77 (m, 1H^4), 7.66 (t, *J* = 7.9 Hz, 1H^5), 3.69 (s, 6H^2); ^{13}C NMR (151 MHz, DMSO-d_6) δ = 142.5, 135.2, 133.1, 131.4, 130.0, 123.4, 123.33, 122.3, 35.7; HRMS (ESI, positive mode): *m/z* calcd for $[C_{11}H_{12}BrN_2]^+$: 251.0179 $[M^{79}Br]^+$, 253.1058 $[M^{81}Br]^+$; found: 251.0190, 253.0171; elemental analysis calcd (%) for $C_{11}H_{12}BrN_2I$: C 34.86, H 3.19, N 7.39; found: C 36.19, H 3.23, N 7.29.

meta-Phenylene Betaine ***m*-PB**

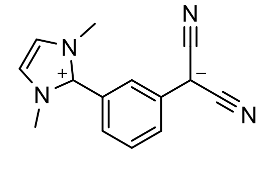

***m*-PB**

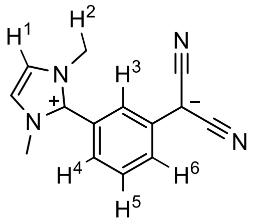

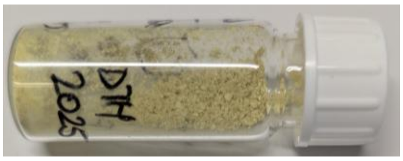

A 10 mL RBF and magnetic stirbar were heated with a heat gun under vacuum and cooled to room temperature under a stream of nitrogen. Dried THF (5 mL) were added to the flask and bubbled with nitrogen for 10 minutes with stirring. $Pd(PPh_3)_4$ (0.018 g, 15 µmol, 0.03 eq.) and malononitrile (0.068 g, 1.1 mmol, 2 eq.) were added. The headspace was flushed with nitrogen, then NaH (0.064 g of 60% dispersion in paraffin oil, 1.6 mmol, 3 eq.) were slowly added to minimise frothing. The murky yellow mixture was stirred for 5 minutes at room temperature under nitrogen. Then (0.199 g, 0.527 mmol, 1 eq.) **9** were added, the flask was equipped with a pre nitrogen-flushed water condenser and the mixture was stirred and heated to reflux on an 87 °C oil bath. Within 5 minutes slightly orange precipitate formed, then after 1.5 hours the mixture was clear yellow with orange precipitate clinging to the walls. After this time, the mixture was cooled to room temperature and remaining NaH was quenched with MeOH (1 mL) and all solvent was removed under reduced pressures. To the caramel-like residue CyH (2 × 3 mL) was added followed by vigorous sonication, the solvent was pipetted away to remove paraffin oil. THF (3 mL) was added, the mixture was vigorously sonicated and allowed to settle, then the solvent was pipetted away. Water (3 mL) was added followed by vigorous sonication, warming in a 50 °C water bath, vigorous sonication again, then finally suction filtration. Washing the yellow cake with excess water, THF (10 mL), and EtOAc (10 mL) removed dark yellow colouration from the fluffy pale-yellow powder *meta*-phenylene betaine ***m*-PB** (0.105 g, 81% yield).

R_f=0.43 (20% MeOH/CH_2Cl_2); m.p. (THF) ~210 °C (darkening), 252-257 °C (decomposition); ^{1}H NMR (500 MHz, DMSO-d_6, ppm): δ = 7.79 (s, 2H^1), 7.31 (t, J = 7.8 Hz, 1H^5), 7.02 – 6.99 (m, 1H^6), 6.85 (t, J = 1.9 Hz, 1H^3), 6.78 – 6.74 (m, 1H^4), 3.66 (s, 6H^2); ^{13}C NMR (176 MHz, DMSO-d_6, ppm): δ= 145.2, 143.5, 129.3, 124.8, 122.8, 121.3, 121.2, 118.4, 118.0, 35.6, 29.1.; IR (solid, cm^{-1}): $\tilde{\nu}$= 3137, 2171, 2133, 1515, 1232, 805, 700; UV/Vis (CH_3CN, nm): λ_{max} (ε)= 227 (21 100), 318 (27 300), ~375 (1 500); fluorescence (CH_3CN, nm): λ_{ex}= 400; λ_{em} (Φ)= 606 (0.01(4) ± 0.01); HRMS (ESI, positive mode): m/z calcd for $[C_{14}H_{12}N_4Na]^+$: 259.0960 $[M+Na]^+$; found: 259.0979; elemental analysis calcd (%) for $C_{14}H_{12}N_4$: C 71.17, H 5.12, N 23.71; found: C 72.64, H 5.85, N 22.52.

Mixture of 2-(4-bromophenyl)-1-*H*-imidazole **11**
and
2-(4-bromophenyl)-1-methylimidazole **12**

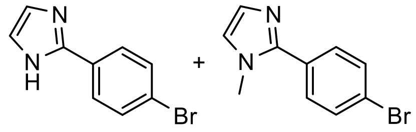

11 **12**

Adapted from literature.[54] Into a 50 mL RBF were added a magnetic stirbar, 4-bromophenylbenzaldehyde (2.002 g, 10.81 mmol, 1 eq.) which was dissolved with sonication in MeOH (10 mL). Methylamine (1.12 mL of 40% solution in water, 11,4 mmol, 1.05 eq.) was added, then the solution was stirred at room temperature for 15 minutes. Glyoxal (1.24 mL of 40% solution in water, 10.8 mmol, 1 eq.) was added followed by powdered ammonium carbonate (0.782 g, 8.11 mmol, 0.75 eq.). Some fizzing was accompanied by the solution turning golden yellow. The mixture was stirred at room temperature for 20 hours, after which time it was concentrated under reduced pressures into a biphasic mixture. Most of the colourless water was carefully pipetted away, then EtOAc (~10 mL) was added to the mixture which was then dried over sodium sulfate. The solution was adsorbed to silica and loaded onto a 10 cm tall × 3.2 cm wide silica column packed in 50% CH_2Cl_2/CyH. Gradient elution: 50% CH_2Cl_2/MeOH (~300 mL), CH_2Cl_2 (~200 mL), 3% MeOH/CH_2Cl_2 (~450 mL). Unreacted 4-bromobenzaldeyhde **10** (0.837 g, 42% recovery) eluted with 50% CH_2Cl_2/CyH and the CH_2Cl_2. The mixed imidazoles eluted with 3% MeOH/CH_2Cl_2 following a yellow-coloured band. Concentration of the eluate under reduced pressures yielded a ~1:2 mixture of 2-(4-bromophenyl)-1-*H*-imidazole **11** and 2-(3-bromophenyl)-1-methylimidazole **12** as a waxy yellow solid (1.071 g, 72% yield based on recovered starting material). The substance was used without further purification.

R_f=0.64 (10% MeOH/CH_2Cl_2), 0.69 (10% MeOH/CH_2Cl_2);^{1}H NMR (400 MHz, DMSO-d_6, ppm): diagnostic signal for **11** δ= 7.04 (broad s, rel. int. 0.36); diagnostic signal for **12** δ= 6.99 (d, J =1.3 Hz, rel. int. 1.00).

2-(4-Bromophenyl)-1,3-dimethylimidazolium iodide **13**

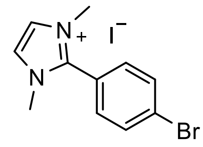

13

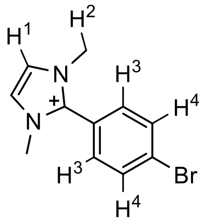

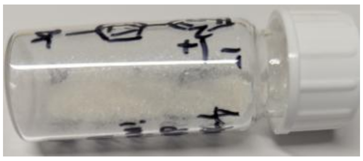

Into a 50 mL RBF with magnetic stirbar were added mixed imidazoles **11** and **12** (1.311 g, ~5.53 mmol, 1 eq.) and CH_3CN (15 mL) and then small chunks of NaOH (0.223 g, 5.53 mmol, 1 eq.) and the mixture was stirred at room temperature for 5 minutes. Then CH_3I (1.72 mL, 27.7 mmol, 5 eq.) was added, a water condenser was equipped to the flask and the mixture was stirred and refluxed on an 84 °C oil bath. The solids dissolved as the mixture was heated. After 2 hours the mixture was cooled to room temperature and gravity filtered because the NaOH had become crushed by the stirbar, although care should be exercised to avoid this*. The filter paper was washed with CH_2Cl_2 (5 mL) and CH_3CN (5 mL), and the solution was concentrated under reduced pressures into a beige solid. The solid was recrystallised from minimal boiling CH_3CN, allowed to cool for 30 minutes at room temperature then for 30 minutes in the freezer. Suction filtration and washing the filter cake with minimal ice cold CH_3CN yielded large flakey crystals of 2-(4-bromophenyl)-1,3-dimethylimidazolium iodide **13** (1.254 g, 60% yield). The solid can also be recrystallised from EtOH, which more efficiently removes coloured impurities.

R_f=0.22 (10% MeOH/CH_2Cl_2);^{1}H NMR (400 MHz, DMSO-d_6, ppm): δ= 7.94 (d, J = 8.5 Hz, 2H^4), 7.87 (s, 2H^1), 7.72 (d, J = 8.5 Hz, 2H^3), 3.68 (s, 6H^2); ^{13}C NMR (151 MHz, DMSO-d_6, ppm): δ= 143.2, 132.7, 132.5, 126.4, 123.3, 120.4, 35.7.; HRMS (ESI, positive mode): m/z calcd for $[C_{11}H_{12}BrN_2]^+$: 251.0179 $[M^{79}Br]^+$, 253.1058 $[M^{81}Br]^+$; found: 251.0181, 253.0161; elemental analysis calcd (%) for $C_{11}H_{12}BrN_2I$: C 34.86, H 3.19, N 7.39; found: C 36.16, H 3.28, N 7.25.

para-Phenylene Betaine ***p*-PB**

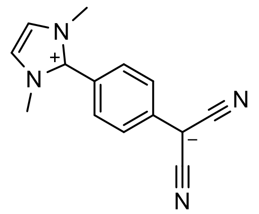

***p*-PB**

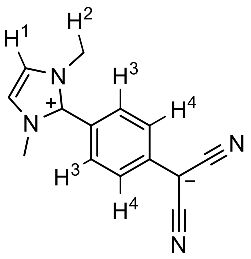

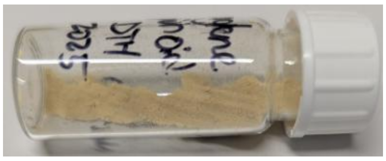

A 10 mL RBF with a magnetic stirbar were heated with a heat gun under vacuum and cooled to room temperature under a stream of nitrogen. Dried THF (5 mL) was added then bubbled with nitrogen for 10 minutes with stirring. $Pd(PPh_3)_4$ (0,018 g, 15 µmol, 0,03 eq.) and malononitrile (0,068 g, 1.1 mmol, 2 eq.) then added NaH (0,065 g of 60% dispersion in paraffin oil, 1.6 mmol, 3 eq.) slowly in portions to minimise frothing. The murky yellow mixture was stirred for 5 minutes at room temperature under nitrogen. Then **13** (0,201 g, 0.528 mmol, 1 eq.) was added and the flask was equipped with a pre nitrogen-flushed water condenser, stirred and heated to reflux on a 87 °C oil bath. Within 5 minutes slightly orange precipitates formed, then after 3 hours the mixture was cloudy with beige precipitates. The mixture was cooled to room temperature, quenched with MeOH (1 mL) and the solvent was removed under reduced pressures. To the beige residue CyH (2 × 3 mL) was added followed by vigorous sonication, the solvent was pipetted away to remove paraffin oil. THF (3 mL) was added, the mixture was vigorously sonicated and allowed to settle, then the solvent was pipetted away. Water (3 mL) was added followed by vigorous sonication, warming in a 50 °C water bath, vigorous sonication again, then finally suction filtration. Washing the solids with excess water, THF (10 mL), EtOAc (10 mL), and CH_2Cl_2 (3 mL) removed brown colouration. Drying in air yielded beige powder *para*-phenylene betaine ***p*-PB** (0,075 g, 60 % yield).

R_f=0.34 (10% MeOH/CH_2Cl_2); m.p. (THF) ~210 °C (darkening), 274-279 °C (decomposition); ^{1}H NMR (400 MHz, DMSO-d_6, ppm): δ= 7.73 (s, 2H^1), 7.32 (d, J = 8.6 Hz, 2H^4), 6.90 (d, J = 8.6 Hz, 2H^3), 3.69 (s, 6H^2); ^{13}C NMR (176 MHz, DMSO-d_6, ppm): δ= 146.7, 145.5, 130.3, 124.0, 122.6, 117.8, 107.3, 35.7, 31.5.; IR (solid, cm^{-1}): $\tilde{\nu}$= 3132, 2169, 2128, 1593, 1474, 13223, 1187, 832; UV/Vis (CH_3CN, nm): λ_{max} (ε)= 366 (29 400), 222 (19 900); fluorescence (CH_3CN, nm): λ_{ex}= 330; λ_{em} (Φ)= 470 (<0.01); HRMS (ESI, positive mode): m/z calcd for $[C_{14}H_{13}N_4]^+$: 237.1140 $[M+H]^+$, 473.2202 $[2M+H]^+$; found: 237.1130, 473.2191; elemental analysis calcd (%) for $C_{14}H_{12}N_4$: C 71.17, H 5.12, N 23.71; found: C 73.16, H 5.36, N 22.63.

Mixture of 2-(2-naphthyl)-1-*H*-imidazole **15**
and
2-(2-naphthyl)-1-methylimidazole **16**

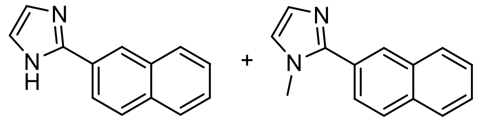

15 **16**

Adapted from literature.[54] Into a 100 mL RBF were added a magnetic stirbar and 2-naphthaldehyde (1.701 g, 10.9 mmol, 1 eq.), with MeOH (20 mL), then it was gently heated until it dissolved. The solution was returned to room temperature, then methylamine (1.14 mL of a 40% solution in water, 11.4 mmol, 1.05 eq) was added and the yellow solution was stirred at room temp for 15 minutes. Powdered ammonium carbonate (0.786 g, 8.16 mmol, 0,75 eq.) were added followed by glyoxal (1.24 mL of 40% solution in water, 10.9 mmol, 1 eq.). The solids fizzed slightly and slowly dissolved as the solution was stirred at room temperature for 20 hours. After that time, it was concentrated under reduced pressures into a biphasic mixture, most water was pipetted away. EtOAc (15 mL) was added, the solution was dried over sodium sulfate, adsorbed to silica, and loaded onto a 10 cm tall × 3.2 cm wide silica column packed in 50% CH_2Cl_2/CyH. Gradient elution: 50% CH_2Cl_2/CyH (~300 mL), CH_2Cl_2 (~200 mL), 5% MeOH/CH_2Cl_2 (~300 mL). The 50% CH_2Cl_2/CyH was collected until a strongly yellow band eluted, recovering unreacted 2-naphthaldehyde (0,829 g, 49% recovery). Using 5% MeOH/CH_2Cl_2, the eluate was collected just before a brown band eluted. All eluent was collected and concentrated into a ~1:7 mixture of 2-(2-naphthyl)-1-*H*-imidazole **15** and 2-(2-naphthyl)-1-methylimidazole **16** as a caramel coloured solid (1.109 g, 96% brsm).

R_f=0.33 (5% MeOH/CH_2Cl_2), 0.41 (5%MeOH/CH_2Cl_2);^{1}H NMR (400 MHz, DMSO-d_6, ppm): diagnostic signals for **15** δ= 8.44 (d, J = 1.2 Hz, rel. int. 0.14), 8.12 (dd, J = 1.7 Hz, 8.6 Hz, rel. int. 0.17); diagnostic signals for **16** δ= 8.23 (d, J = 1.2 Hz, rel. int. 1.00), 7.87 (dd, J = 1.8 Hz, 8.6 Hz, rel. int. 1.06).

1,3-Dimethyl-2-(2-naphthyl)imidazolium iodide **17**

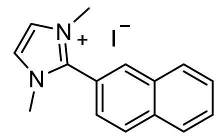

17

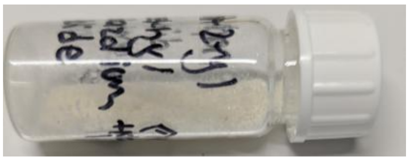

Into a 50 mL RBF with mixed imidazoles **15** and **16** (1.109 g, ~4.85 mmol, 1 eq.) were added a magnetic stirbar and CH_3CN (12 mL), then NaOH (0.202 g, 0.485 mmol, 1 eq.) in chunks and the mixture was stirred at room temperature for 5 minutes. CH_3I (1.51 mL, 24.3 mmol, 5 eq.) was added, the flask was equipped with a water condenser and the mixture was refluxed and stirred on a 87 °C oil bath. The solids were dissolved on heating and the solution turned medium brown and clear. After 1 hour of heating, the reaction was cooled to room temperature, the remaining NaOH chunk was removed and the solvent was removed under reduced pressures. The resulting beige-brown solid was recrystallised from boiling EtOH, cooled at room temperature for 30 minutes, then suction filtered and washed with minimal ice cold EtOH to yield beige flakey crystals. The solid was then recrystallised from boiling CH_3CN, allowed to cool at room temperature for 30 minutes then suction filtered and washed with ice cold CH_3CN. Drying yielded large rice-like off-white crystals of 1,3-dimethyl-2-(2-naphthyl)imidazolium iodide **17** (0.857 g, 50% yield).

R_f=0.11 (10% MeOH/CH_2Cl_2);^{1}H NMR (500 MHz, DMSO-d_6, ppm): δ= 8.46 – 8.43 (m, 1H), 8.24 (d, J = 8.5 Hz, 1H), 8.14 – 8.08 (m, 2H), 7.93 (s, 2H), 7.81 (dd, J = 8.5, 1.8 Hz, 1H), 7.76 (ddd, J = 8.2, 6.8, 1.5 Hz, 1H), 7.71 (ddd, J = 8.1, 6.9, 1.4 Hz, 1H), 3.77 (s, 6H); ^{13}C NMR (151 MHz, DMSO-d_6, ppm): δ= 144.2, 134.0, 132.1, 131.8, 129.1, 128.8, 128.7, 128.0, 127.6, 125.9, 123.3, 118.4, 35.9; IR (solid, cm^{-1}): $\tilde{\nu}$= 3052, 1252, 819, 764, 482; UV/Vis (CH_3CN, nm): λ_{max} (ε)= 243 (45 800), 282 (7 900), 324 (1 200); fluorescence (CH_3CN, nm): λ_{ex}= 250; λ_{em} (Φ)= 357 (0.13 ± 0.01); HRMS (ESI, positive mode): m/z calcd for $[C_{15}H_{15}N_2]^+$: 223.1230 $[M]^+$; found: 223.1229; elemental analysis calcd (%) for $C_{15}H_{15}N_2I$: C 51.45, H 4.32, N 8.00; found: C 51.38, H 4.57, N 8.09.

2-(2-Naphthyl)malononitrile **19**

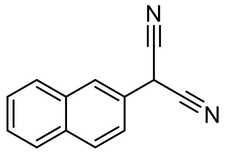

19

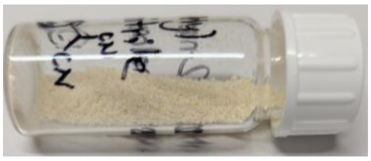

A 50 mL RBF and magnetic stirbar were heated with a heat gun under vacuum and cooled to room temperature under a stream of nitrogen. Dried THF (8 mL) was bubbled with nitrogen for 10 minutes while stirring. $Pd(PPh_3)_4$ (0.082 g, 72 μmol, 0.03 eq.) and malononitrile (0.319 g, 4.83 mmol, 2 eq.) were added, followed by NaH (0.288 g 60% dispersion in paraffin oil, 7.24 mmol, 3 eq.) slowly to minimise frothing. The murky orange mixture was stirred for 5 minutes at room temperature under nitrogen. Then 2-bromonaphthalene (0.501 g, 2.41 mmol, 1 eq.) was added and the flask was equipped with a pre nitrogen-flushed water condenser. The reaction was stirred and heated to reflux on an 87 °C oil bath for 20 hours. The mixture was cooled to room temperature, then MeOH (1 mL) was added to quench remaining NaH and the solvent was removed by under reduced pressures. The resulting caramel residue was diluted with CH_2Cl_2 (15 mL) and water (10 mL), then concentrated HCl was added to bring the pH to around 2 (by paper). The layers were shaken and separated, then the aqueous layer was extracted with CH_2Cl_2 (3 x 5 mL) *caution emulsion*. The CH_2Cl_2 layer was dried over sodium sulfate, and concentrated under reduced pressures into a brown oil. The oil was dissolved in CH_2Cl_2, adsorbed to silica then loaded onto a 7.5 cm tall × 2.2 cm wide silica column packed in CyH. Gradient: CyH (~100 mL), 5% EtOAc/CyH (~100 mL), 20% EtOAc (~150 mL). Paraffin oil eluted in the CyH, and 5% EtOAc/CyH eluted unreacted bromonaphthalene. A dark purple band completely co-eluted with the desired product, which was collected together and concentrated under reduced pressures into a dark purple solid. The solid was recrystallised in boiling 50% CH_2Cl_2/CyH, cooled at room temperature for several hours to produce slightly purple blocky prisms. The solid was sublimated under a high vacuum with gentle heating from a heat gun, to yield 2-(2-naphthyl)malononitrile **19** (0.188 g, 44% yield) as an air-sensitive white powder: characterisation data conforms with literature.[55]

2,7-Dibromonaphthalene **21**

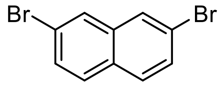

21

This compound was prepared according to a literature procedure, and characterisation conformed to the reported data.[56]

7-Bromo-2-naphthaldehyde **22**

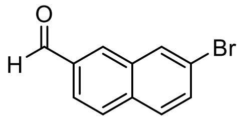

22

This compound was prepared according to a literature procedure, and characterisation conformed to the reported data.[57]

Mixture of 2-(7-bromo-2-naphthyl)-1-*H*-imidazole **24**
and
2-(7-bromo-2-naphthyl)-1-methylimidazole **23**

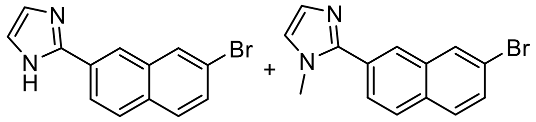

24 **23**

Into a 100 mL RBF with 7-bromo-2-naphthaldehyde **22** (1.291 g, 5.49 mmol, 1 eq.) was added a magnetic stirbar, MeOH (20 mL) and the suspension was stirred on a 50 °C oil bath. At room temperature the solubility in MeOH is too low for full dissolution. Methylamine (0.57 mL of 40% solution in water, 5,76 mmol, 1.05 eq.) was added causing the solids to slowly dissolve and a yellow solution to develop. The mixture was stirred at 50 °C for 15 minutes, then it was brought to room temperature and ammonium carbonate (0.394 g, 4.12 mmol, 0.75 eq.) was added followed by glyoxal (0.62 mL of a 40% solution in water, 5.49 mmol, 1 eq.). The solids fizzed slightly, and the mixture was stirred at room temp for 20 hours, the solids slowly dissolving. The resulting solution was concentrated under reduced pressures into a biphasic mixture, most of the water was pipetted away, then the mixture was diluted with EtOAc (15 mL), dried over sodium sulfate and concentrated under reduced pressures into a brown oil. The oil was dissolved in 50% CH_2Cl_2/CyH and loaded onto a 10 cm tall × 2.3 cm wide silica column packed in 50% CH_2Cl_2/CyH. Gradient elution; 50% CH_2Cl_2/CyH (~400 mL), CH_2Cl_2(~200 mL), 5% MeOH/CH_2Cl_2 (~200 mL). The 50% CH_2Cl_2/CyH and some 100% CH_2Cl_2 were concentrated under reduced pressures to recover unreacted 7-bromo-2-naphthaldehyde **22** (0.610 g, 47% recovery) as a pale yellow solid. The 5% MeOH was collected, but stopped just before a significant brown band eluted. Concentration yielded mixed 2-(7-bromo-2-naphthyl)-1-*H*-imidazole **24** and 2-(7-bromo-2-naphthyl)-1-methylimidazole **23** (0.773g, 92% yield brsm) as a brown solid:

R_f=0.33 (5% MeOH/CH_2Cl_2), 0.44 (5%MeOH/CH_2Cl_2);^{1}H NMR (400 MHz, DMSO-d_6, ppm): diagnostic signals for **24** δ= 8.19 (d, J= 2.0 Hz, rel. int. 0.26), 7.62 (dd, J= 8.8 Hz, 2.0 Hz, rel. int. 0.29); diagnostic signals for **23** δ= 8.24 (d, J= 1.7 Hz, rel. int. 1.00), 7.68 (dd, J = 1.7 Hz, 8.7 Hz, rel. int. 1.00).

2-(7-Bromo-2-naphthyl)-1,3-dimethylimidazolium iodide **25**

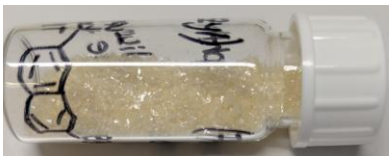

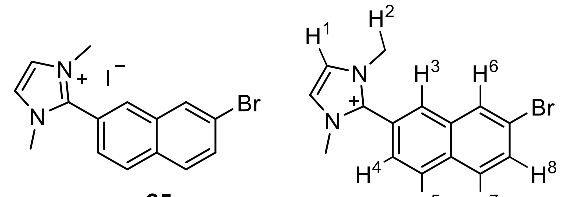

25

To a 50 mL RBF with mixed imidazoles **23** and **24** (0.687 g, ~2.39 mmol, 1 eq.) and a magnetic stirbar was added CH_3CN (10 mL) then NaOH chunks (0.096 g, 2.39 mmol, 1 eq.), and the mixture was stirred at room temperature for 5 minutes. CH_3I (0.75 mL, 11,96 mmol, 5 eq.) was added, the flask was equipped with a water condenser and the mixture was refluxed and stirred on an 87 °C oil bath for 1 hour. With heating the solids dissolved, forming a clear orange solution, then within 10 minutes pale beige solids began to form. After 1 hour the mixture was cooled to room temperature, remaining NaOH was removed with tweezers, then the solvents removed under reduced pressures. The resulting beige solid was recrystallised from boiling 40% DMF/EtOH, cooled at room temperature for 30 minutes, then in the freezer for 3 hours. The solids were suction filtered, washed with ice cold EtOH to yield flakey beige crystals of 2-(7-bromo-2-naphthyl)-1,3-dimethylimidazolium iodide **25** (0.744 g, 75% yield).

R_f=0.10 (10% MeOH/CH_2Cl_2);^{1}H NMR (500 MHz, DMSO-d_6, ppm): δ= 8.45 – 8.36 (m, 1H^3 1H^6), 8.27 (d, J = 8.5 Hz, 1H^5), 8.10 (d, J = 8.8 Hz, 1H^7), 7.93 (s, 2H^1), 7.87 (m, 1H^4 1H^8), 3.76 (s, 6H^2); ^{13}C NMR (151 MHz, DMSO-d_6, ppm): δ= 143.7, 133.3, 132.5, 131.7, 131.1, 130.5, 130.3, 129.3, 126.6, 123.5, 120.8, 119.6, 35.9; HRMS (ESI, positive mode): m/z calcd for $[C_{15}H_{12}BrN_2]^+$: 301.0335 $[M^{79}Br]^+$, 303.0315 $[M^{81}Br]^+$; found: 301.0334, 303.0315; elemental analysis calcd (%) for $C_{15}H_{12}BrN_2I$: C 41.99, H 3.29, N 6.53; found: C 41.91, H 3.05, N 6.52.

pseudo-*meta*-Naphthylene Betaine **pseudo-*m*-NB**

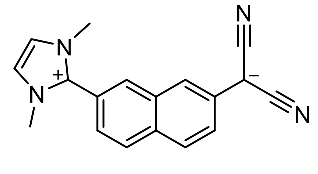

pseudo-*m*-NB

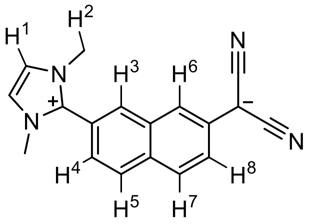

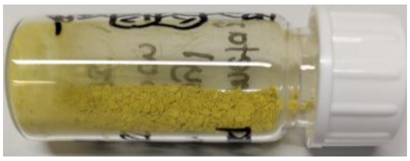

A 25 mL RBF with a magnetic stirbar was heated with a heat gun under vacuum and cooled to room temperature under a stream of nitrogen, then dried THF (10 mL) was added and bubbled with nitrogen for 10 minutes. $Pd(PPh_3)_4$ (0.036 g, 32 μmol, 0.03 eq.) and malononitrile (0.210 g, 3.17 mmol, 3 eq.) were added were added and the solution was stirred at room temperature, then NaH (0.210 g of a 60% dispersion in paraffin oil, 4.22 mmol, 4 eq.) were added slowly to minimise frothing. The murky pineapple-coloured solution was stirred at room temperature for 5 mins, then imidazolium salt **25** (0.453 g, 1.06 mmol, 1 eq.) was added, the flask was equipped with a pre nitrogen-flushed water condenser and the mixture was stirred and refluxed on a 87 °C oil bath for 24 hours, becoming mango coloured and precipitating golden solids slowly on heating. Mango yellow precipitates appeared after 10 minutes. After 24 hours, the mixture was cooled to room temperature, quenched with MeOH (1 mL), and the solvent was removed under reduced pressures. The resulting yellow sludge was vigorously sonicated with CyH (2 × 5 mL), pipetting away the solvent to remove paraffin oil, then vigorously sonicated with water (5 mL). This released fluffy yellow solids which were suction filtered, washed with excess room temperature water, THF, EtOAc, and a small volume of CH_2Cl_2 to yield a canary yellow solid. To remove unreacted imidazolium salt, the solid was boiled with water (~20 mL), washed with boiling water (3 × 10 mL) and dried by suction. This process was conducted a total of three times, then finally it was washed with THF to yield pseudo-*meta*-naphthylene betaine **pseudo-*m*-NB** (0.159 g, 53% yield) as a canary yellow powder.

R_f=0.15 (10% MeOH/CH_2Cl_2); m.p. (THF) ~240 °C (darkening), 276-280 °C (decomposition); ^{1}H NMR (700 MHz, DMSO-d_6, ppm): δ= 7.94 (dd, J = 1.7, 0.8 Hz, 1H^3), 7.91 (m, 1H^5), 7.85 (s, 2H^1), 7.72 – 7.70 (m, 1H^7), 7.27 (dd, J = 8.3, 1.8 Hz, 1H^4), 7.25 (dd, J = 8.7, 2.0 Hz, 1H^8), 7.16 – 7.14 (m, 1H^6), 3.75 (s, 6H^2); ^{13}C NMR (151 MHz, DMSO-d_6, ppm): δ= 144.9, 141.64, 133.9, 128.8, 128.7, 128.3, 127.7, 125.3, 123.6, 123.1, 120.6, 118.3, 112.0, 35.8, 29.9.; IR (solid, cm^{-1}): $\tilde{\nu}$= 3086, 2168, 2129, 2107, 1602, 843, 467; UV/Vis (CH_3CN, nm): λ_{max} (ε)= 434 (3 000), 348 (30 100), 278 (23 300), 233 (36 100); fluorescence (CH_3CN, nm): λ_{ex}= 450; λ_{em} (Φ)= 644 (0.02(3) ± 0.01); HRMS (ESI, positive mode): m/z calcd for $[C_{18}H_{15}N_4]^+$: 287.1297 $[M+H]^+$, 309.1116 $[M+Na]^+$; found: 287.1288, 309.1115; elemental analysis calcd (%) for $C_{18}H_{14}N_4$: C 75.70, H 4.93, N 19.57; found: C 73.46, H 5.42, N 17.90.

6-Bromo-2-naphthaldehyde **27**

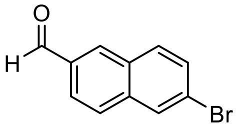

27

This compound was prepared according to a literature procedure, and characterisation conformed to the reported data.[58]

Mixture of 2-(6-bromo-2-naphthyl)-1-*H*-imidazole **28** and 2-(6-bromo-2-naphthyl)-1-methylimidazole **29**

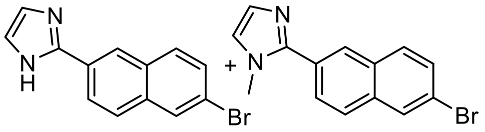

28 **29**

Into a 100 mL RBF were added 6-bromo-2-naphthaldehyde **27** (2.311 g, 9.83 mmol, 1 eq.) and a magnetic stirbar, followed by MeOH (40 mL). The mixture was stirred on a 50 °C oil bath, not dissolving the solids, then methylamine (1.02 mL of a 40% solution in water, 10.3 mmol, 1.05 eq.) was added. The solids slowly dissolved, and the mixture was stirred at 50 °C for 15 minutes, then ammonium carbonate (0.781 g, 7.37 mmol, 0.75 eq.) and glyoxal (1.12 mL of a 40% solution in water, 9.83 mmol, 1 eq.) were added. The carbonate solids fizzed and slowly dissolved, causing the solution to turn yellow, then the solution was stirred at room temperature for 20 hours. The solvent was removed under reduced pressures yielding a solid residue, most remaining water was pipetted away and then the solid was dissolved in EtOAc, dried with sodium sulfate, and concentrated again while adsorbing to silica. The mixture was then loaded onto a 10 cm tall ×3.2 cm wide silica column packed in 50% CH_2Cl_2/CyH. Gradient elution: 50% CH_2Cl_2/CyH (~600 mL), CH_2Cl_2 (~200 mL), 5% MeOH/ CH_2Cl_2 (~400 mL). The 50% CH_2Cl_2/CyH and CH_2Cl_2were collected and concentrated under reduced pressures to recover unreacted 6-bromo-2-naphthaldehyde **27** (1.171 g, 51% recovery) as a pale-yellow solid. The 5% MeOH was collected and stopped just before a brown band eluted. Concentration yielded mixed 2-(6-bromo-2-naphthyl)-1-*H*-imidazole **28** and 2-(6-bromo-2-naphthyl)-1-methylimidazole **29** (1.220 g, 88% brsm) as a dark yellow granular solid.

R_f=0.37 (10% MeOH/CH_2Cl_2), 0.50 (10% MeOH/CH_2Cl_2).

2-(6-Bromo-2-naphthyl)-1,3-dimethylimidazolium iodide **30**

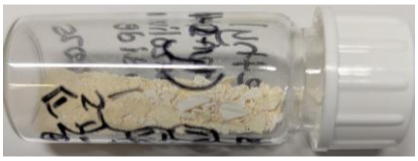

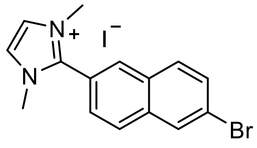

30

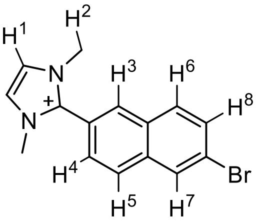

Into a 100 mL RBF containing of mixed imidazoles **28** and **29** (1.220 g, 4.25 mmol, 1 eq.) was added a magnetic stirbar, CH_3CN (20 mL) and one chunk of NaOH (0.181 g, 4.25 mmol, 1 eq.). The mixture was stirred at room temperature for 5 minutes, the CH_3I (1.32 mL, 21.2 mmol, 5 eq.) were added, the flask was equipped with a water condenser and the mixture was refluxed and stirred on a 87 °C oil bath. The suspended solids slowly dissolved with heating, forming a pale brown solution. After 3 hours the reaction was cooled to room temperature, the NaOH chunk was removed and the solvent removed under reduced pressures to yield a dark brown solid. The solid was recrystallised from minimal boiling 50% CH_3CN/THF, cooled to room temperature for 30 minutes, in the freezer for 2 hours, then suction filtered and washed with excess ice cold THF to yield a dark mustard-coloured solid. The solid was again recrystallised from boiling 33% CH_3CN/THF, cooled to room temp for 30 minutes, then in the freezer for 2 hours. Suction filtration and washing with excess ice cold THF providing a cream coloured solid which yellowed on drying in air. After drying, the solid was sonicated in room temperature THF (5 mL) and suction filtered again, washed with excess THF and dried. This process was repeated three times total, yielding 2-(6-bromo-2-naphthyl)-1,3-dimethylimidazolium iodide **30** (1.096 g, 68% yield) as a cream coloured solid.

R_f=0.20 (10% MeOH/CH_2Cl_2);^{1}H NMR (500 MHz, DMSO-d_6, ppm): δ= 8.45 (s, 1H^3), 8.44 (s, 1H^7), 8.22 (d, J = 8.5 Hz, 1H^6), 8.06 (d, J = 8.8 Hz, 1H^5), 7.91 (s, 2H^1), 7.86 (dd, J = 8.7, 1.9 Hz, 1H^8), 7.84 (dd, J = 8.5, 1.9 Hz, 1H^4) 3.75 (s, 6H^2); ^{13}C NMR (151 MHz, DMSO-d_6, ppm): δ= 143.9, 135.1, 132.0, 130.9, 130.7, 130.62, 130.0, 128.4, 127.2, 123.4, 122.2, 119.0, 35.9.; HRMS (ESI, positive mode): m/z calcd for $[C_{15}H_{12}BrN_2]^+$: 301.0335 $[M^{79}Br]^+$, 303.0315 $[M^{81}Br]^+$; found: 301.0394, 303.0378; elemental analysis calcd (%) for $C_{15}H_{12}BrN_2I$: C 41.99, H 3.29, N 6.53; found: C 41.01, H 3.15, N 6.59.

pseudo-*para*-Naphthylene Betaine **pseudo-*p*-NB**

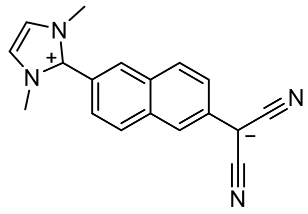

pseudo-*p*-NB

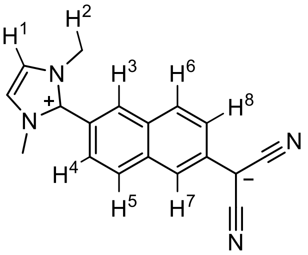

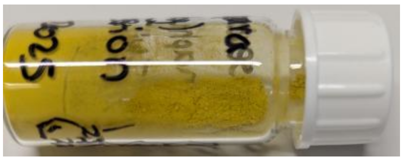

A 10 mL RBF with magnetic stirbar was heated with a heat gun under vacuum and cooled to room temperature under a stream of nitrogen, then to it was added dried THF (5 mL) which was bubbled for 10 minutes with nitrogen. $Pd(PPh_3)_4$ (0.018 g, 16 µmol, 0.03 eq.) and malononitrile (0.104 g, 1.57 mmol, 3 eq.) were added and stirred at room temperature, flushed with nitrogen, then NaH (0.085 g of a 60% dispersion in paraffin oil, 2.10 mmol, 4 eq.) was added slowly to minimise frothing. The resulting pineapple yellow solution was stirred for 5 mins, then imidazolium salt **30** (0.226 g, 0.524 mmol, 1 eq.) was added. The flask was equipped with a pre nitrogen-flushed water condenser, the mixture was stirred and refluxed on a 87 °C oil bath, turning dark yellow-orange on heating, and after 30 minutes precipitates separated out of the solution and clung to the walls of the flask. After 3 hours, the mixture was allowed to cool to room temperature and then it was quenched with MeOH (1 mL) and the solvent was removed under reduced pressures. The resulting yellow sludge was vigorously sonicated with CyH (2 × 5 mL) and the solvent was pipetted away to remove paraffin oil. The solid was vigorously sonicated with water (5 mL) which formed fluffy yellow solids, which were suction filtered and washed with excess water, THF to remove dark yellow colouration, EtOAc, and some CH_2Cl_2. Drying in air yielded pseudo-*para*-naphthylene betaine **pseudo-*p*-NB** (0.130 g, 87% yield) as a golden yellow solid.

R_f=0.29 (10% MeOH/CH_2Cl_2); m.p. (THF) ~240 °C (darkening), 282-286 °C (decomposition); ^{1}H NMR (500 MHz, DMSO-d_6, ppm): δ= 8.00 (d, J = 2.1 Hz, 1H^3), 7.83 (s, 2H^1), 7.73 (d, J = 8.6 Hz, 1H^5), 7.69 (d, J = 8.8 Hz, 1H^6), 7.47 (dd, J = 8.5, 1.9 Hz, 1H^4), 7.20 (dd, J = 8.7, 2.1 Hz, 1H^8), 7.10 (d, J = 2.0 Hz, 1H^7), 3.74 (s, 6H^2); ^{13}C NMR (176 MHz, DMSO-d_6, ppm): δ= 145.0, 143.3, 135.7, 131.1, 128.5, 126.3, 125.9, 125.73, 124.8, 122.9, 122.2, 112.3, 111.0, 35.8, 31.3; IR (solid, cm^{-1}): $\tilde{\nu}$=3130, 2164, 2124, 2102, 1612, 1216, 710, 467; UV/Vis (CH_3CN, nm): λ_{max} (ε)= 427 (13 600), 367 (17 000), 289 (18 900), 241 (50 900); fluorescence (CH_3CN, nm): λ_{ex}= 430; λ_{em} (Φ)= 545 (0.469 ± 0.023); HRMS (ESI, positive mode): m/z calcd for $[C_{18}H_{15}N_4]^+$: 287.1297 $[M+H]^+$, 309.1116 $[M+Na]^+$; found: 287.1288, 309.1103; elemental analysis calcd (%) for $C_{18}H_{14}N_4$: C 75.70, H 4.93, N 19.57; found: C 71.83, H 4.63, N 17.70.

Section S4 Analytical Spectra of Synthesised Compounds

^{1}H NMR (400 MHz, DMSO-d_6, 298 K) and for mixture of 2-(3-bromophenyl)-1-*H*-imidazole **7** and 2-(3-bromophenyl)-1-methylimidazole **8**.

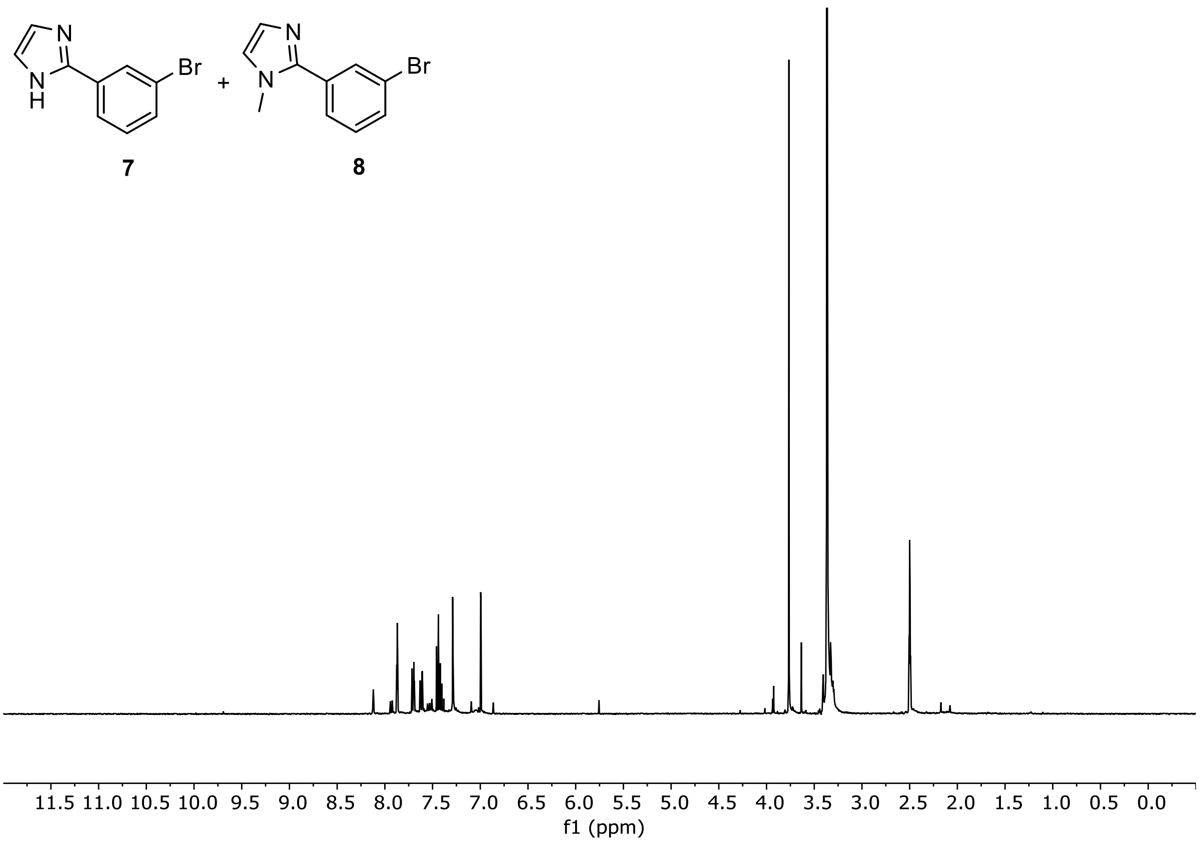

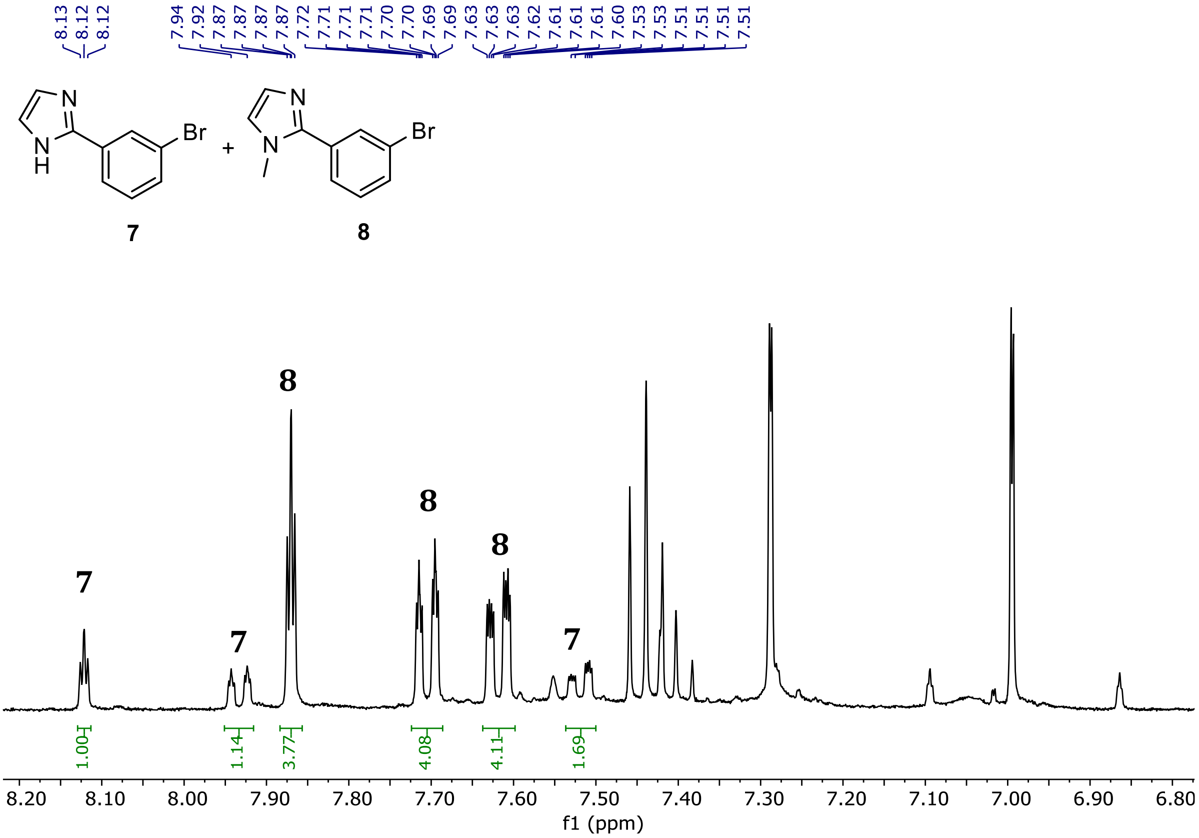

^{1}H NMR (400 MHz, DMSO-d_6, 298 K, top) and $^{13}C\{^{1}H\}$ NMR (151 MHz, DMSO-d_6, 298 K, bottom) for 2-(3-bromophenyl)-1,3-dimethylimidazolium iodide **9**.

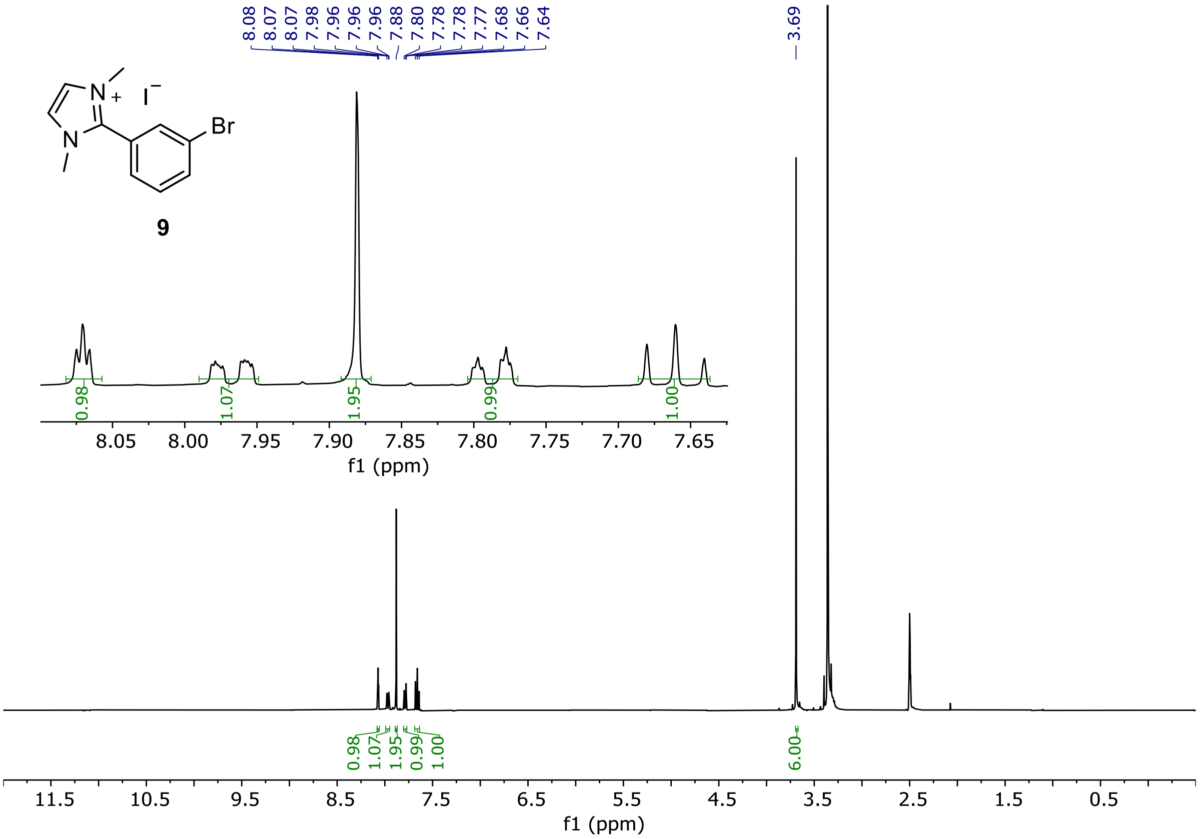

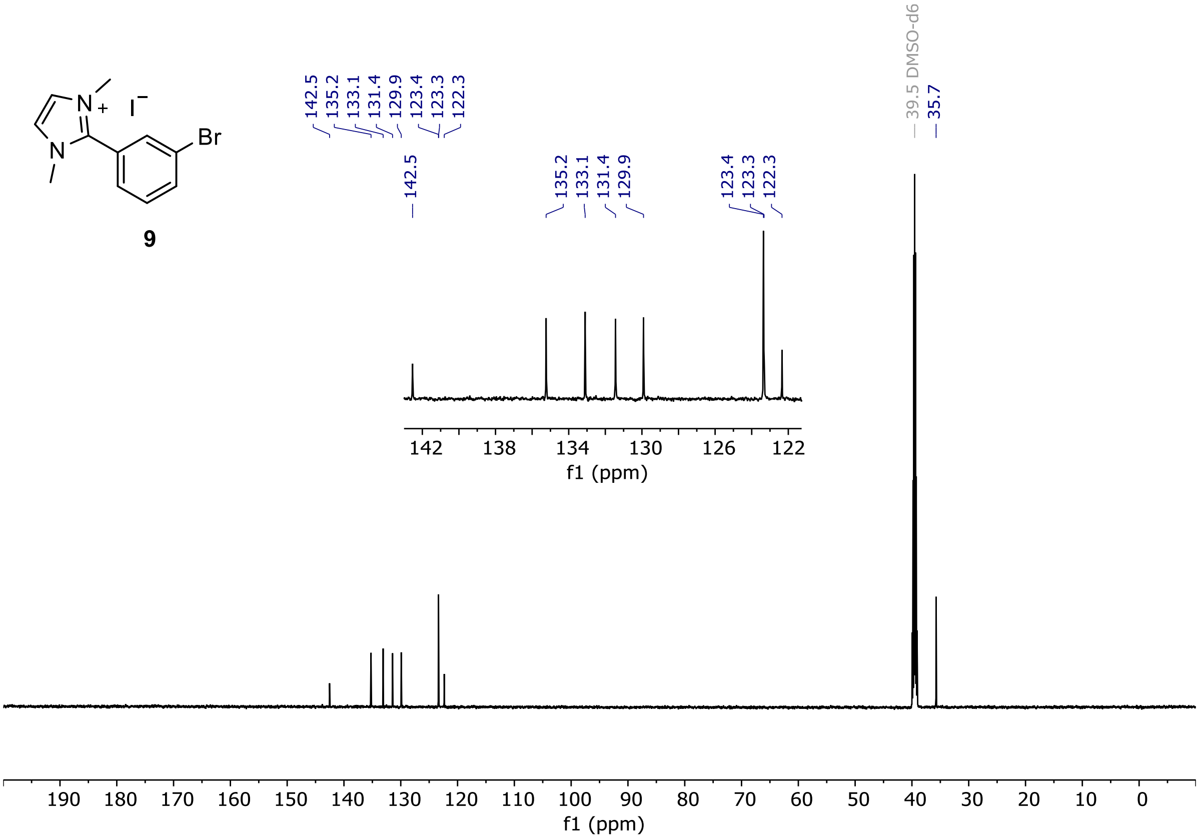

ESI-MS spectrum (positive mode) of 2-(3-bromophenyl)-1,3-dimethylimidazolium iodide **9** as the $[M]^+$ complex at 251.0190 and 253.0171 m/z.

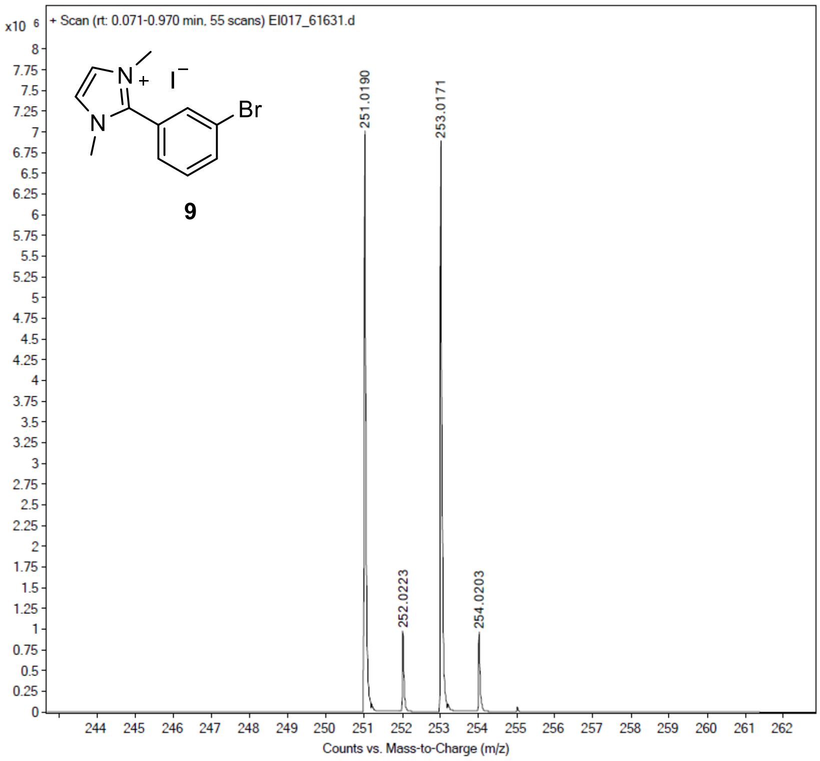

^{1}H NMR (500 MHz, DMSO-d_6, 298 K, top) and $^{13}C\{^{1}H\}$ NMR (176 MHz, DMSO-d_6, 298 K, bottom) for *meta*-phenylene betaine ***m*-PB**.

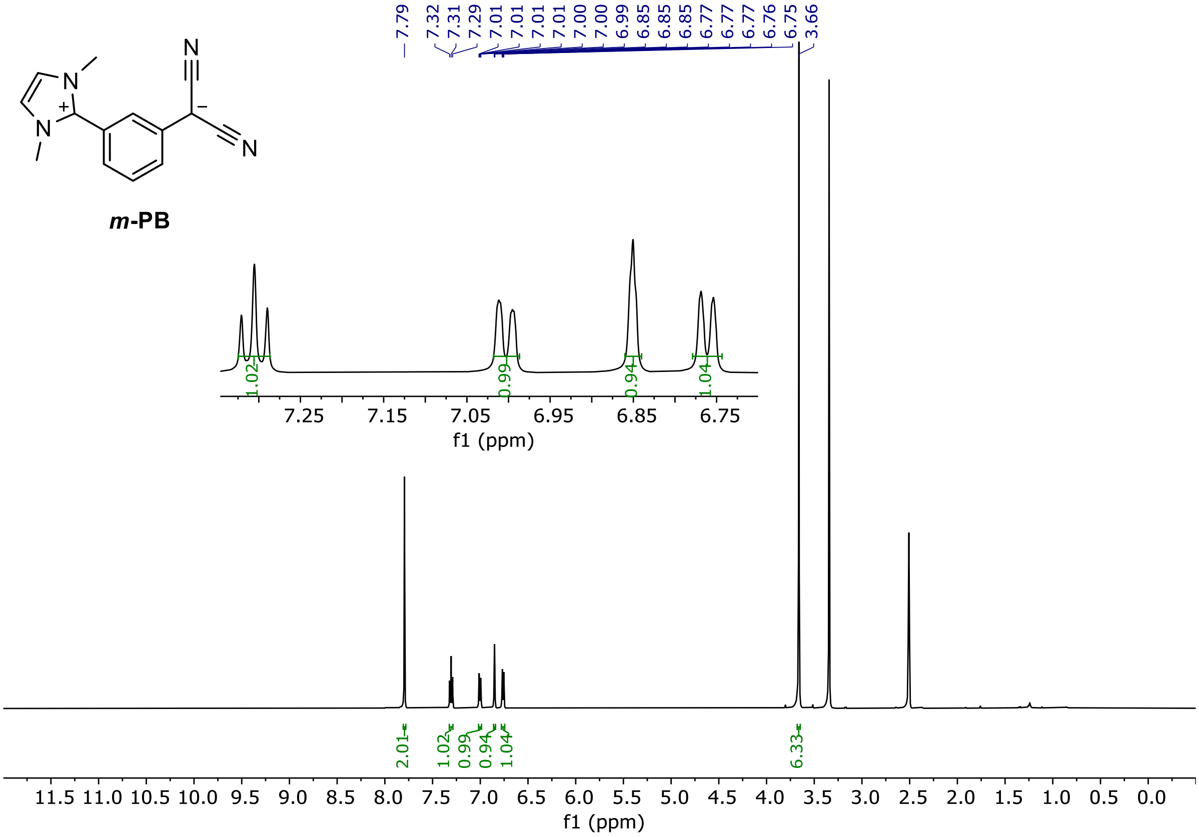

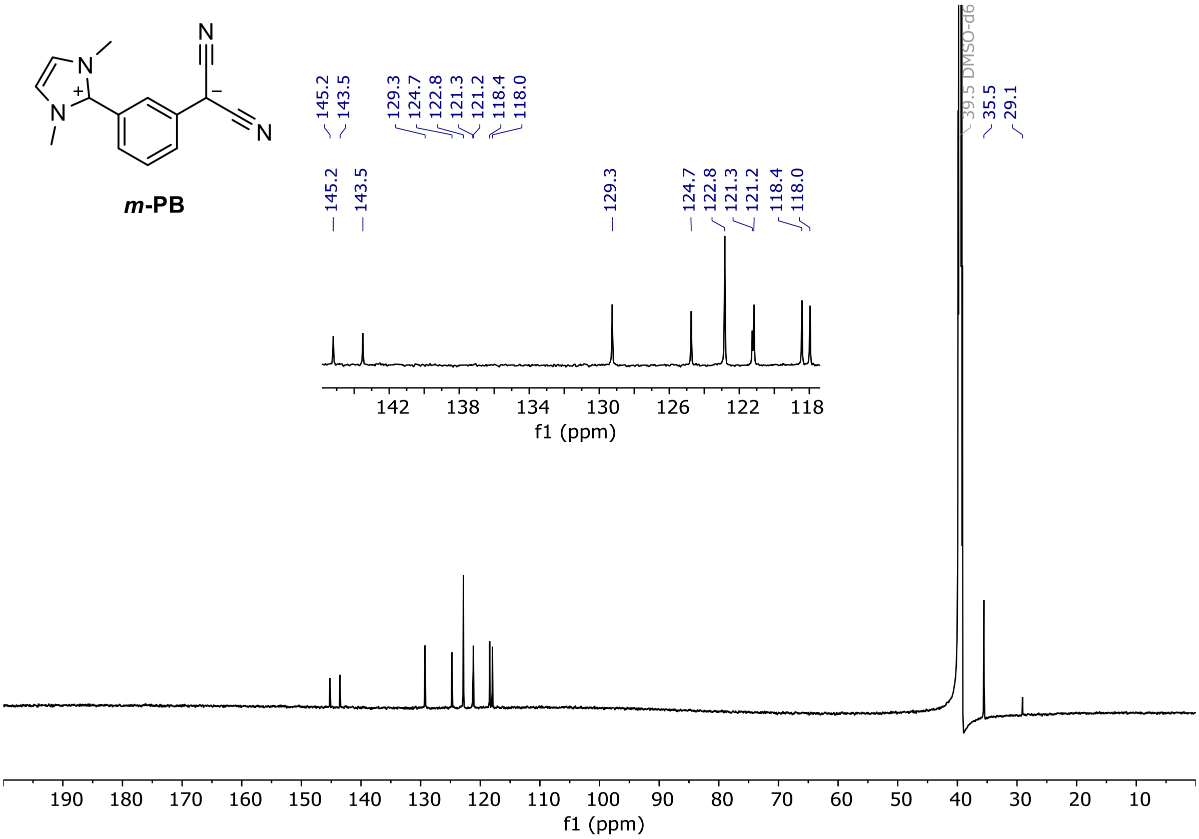

ESI-MS spectrum (positive mode) of *meta*-phenylene betaine ***m*-PB** as the $[M+Na]^+$ complex at 259.0979 m/z.

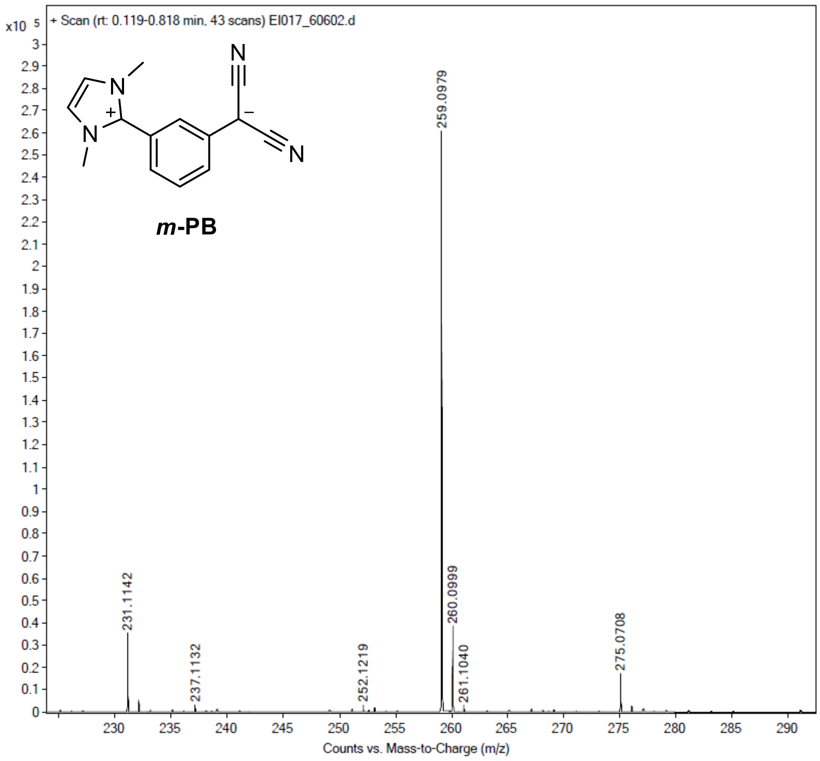

^{1}H NMR (400 MHz, DMSO-d_6, 298 K) and for mixture of 2-(4-bromophenyl)-1-*H*-imidazole **11** and 2-(4-bromophenyl)-1-methylimidazole **12.**

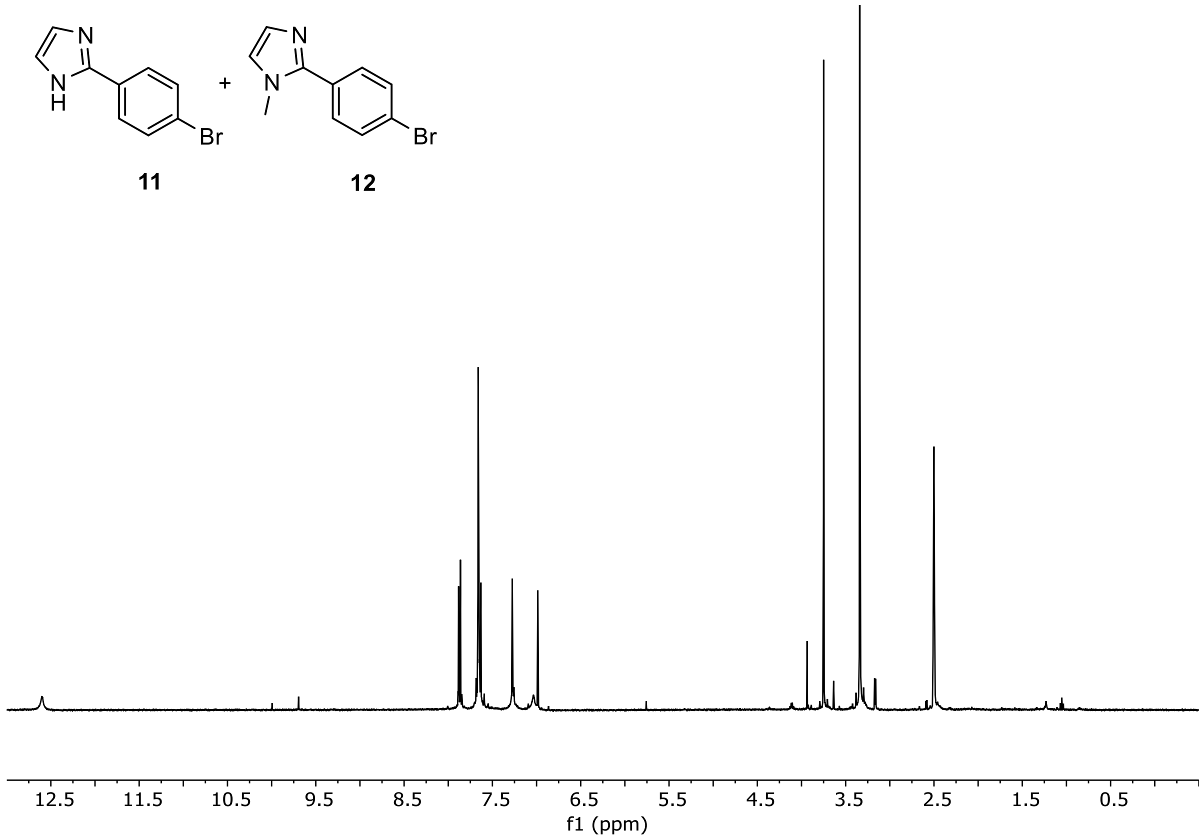

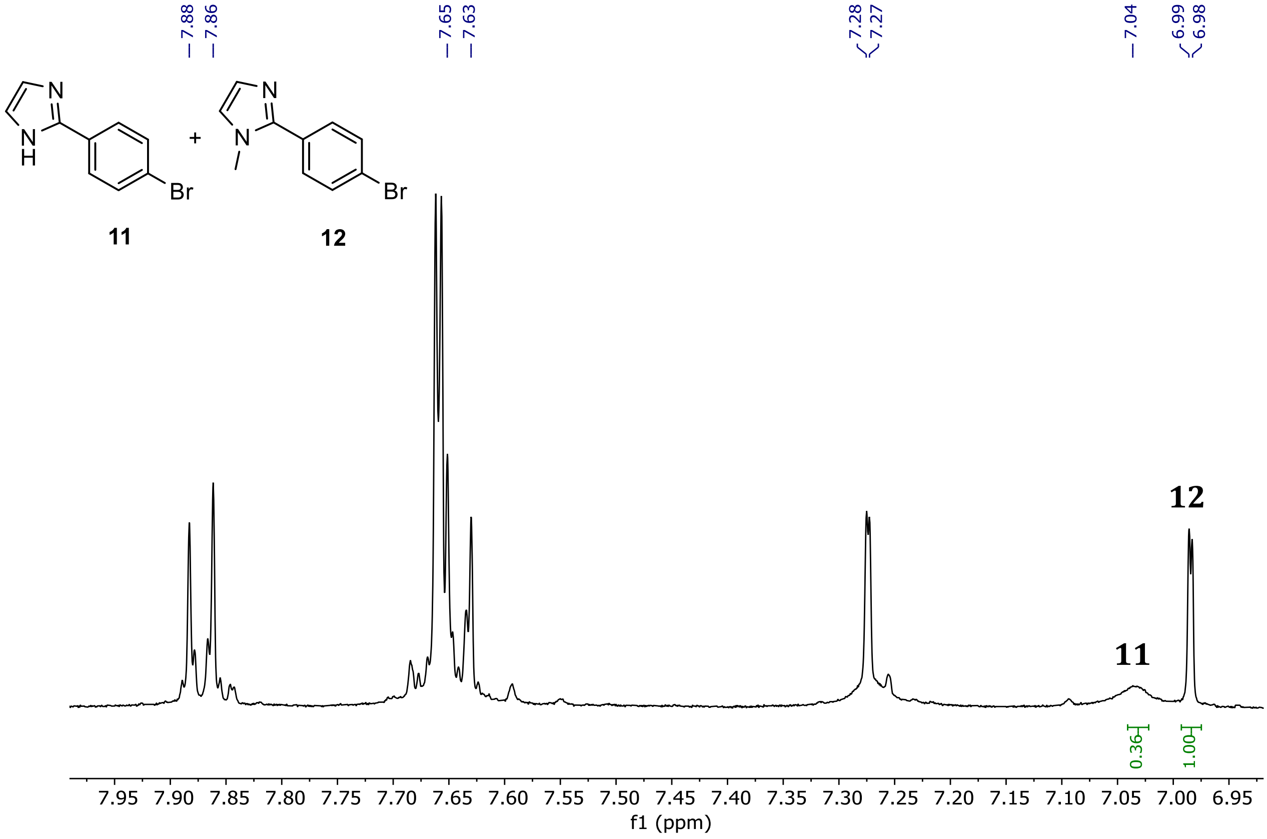

^{1}H NMR (400 MHz, DMSO-d_6, 298 K, top) and ^{13}C{^{1}H} NMR (151 MHz, DMSO-d_6, 298 K, bottom) for 2-(4-bromophenyl)-1,3-dimethylimidazolium iodide **13.**

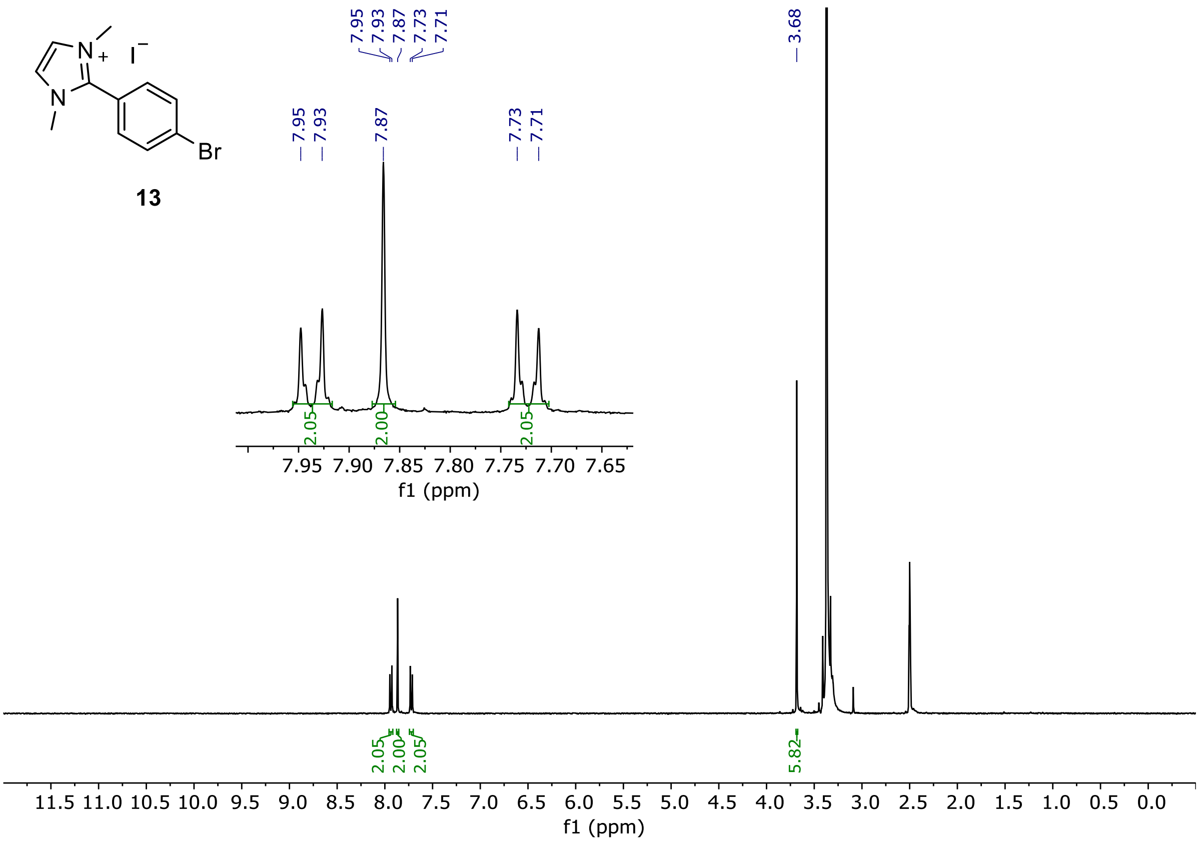

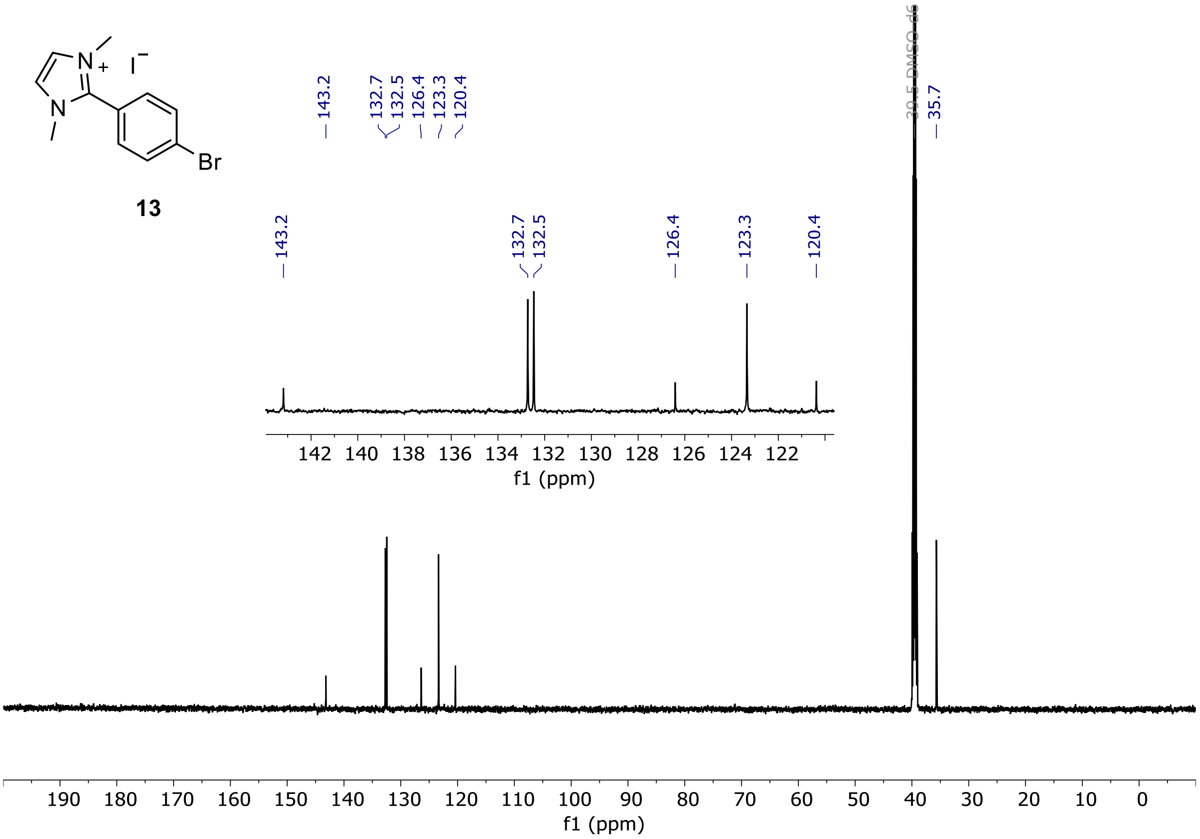

ESI-MS spectrum (positive mode) of 2-(4-bromophenyl)-1,3-dimethylimidazolium iodide **13** as the $[M]^+$ complex at 251.0181 and 253.0161 m/z.

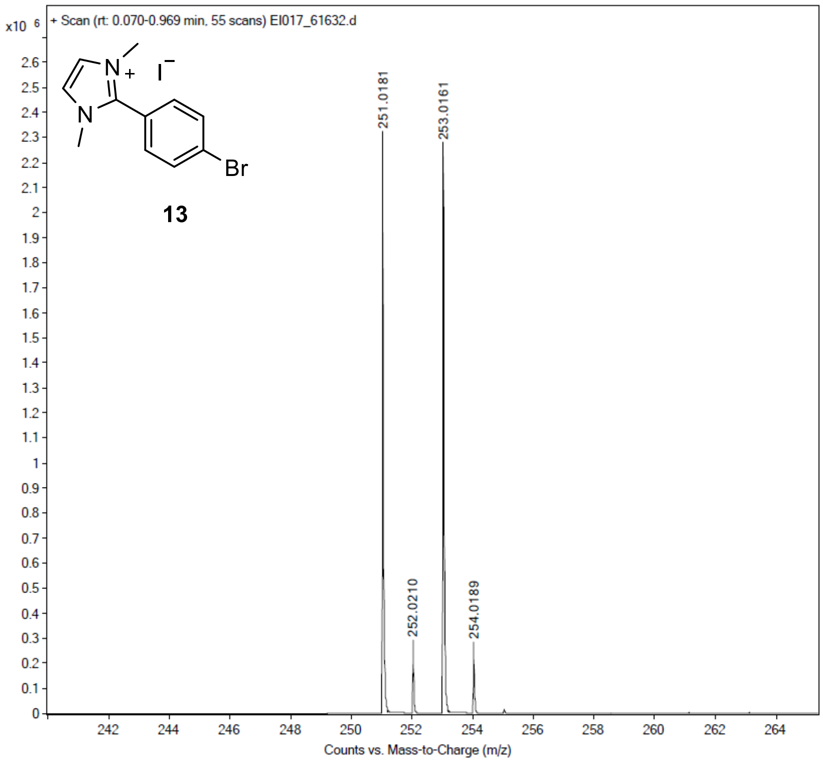

^{1}H NMR (400 MHz, DMSO-d_6, 298 K, top) and $^{13}C\{^{1}H\}$ NMR (176 MHz, DMSO-d_6, 298 K, bottom) for *para*-phenylene betaine ***p*-PB**.

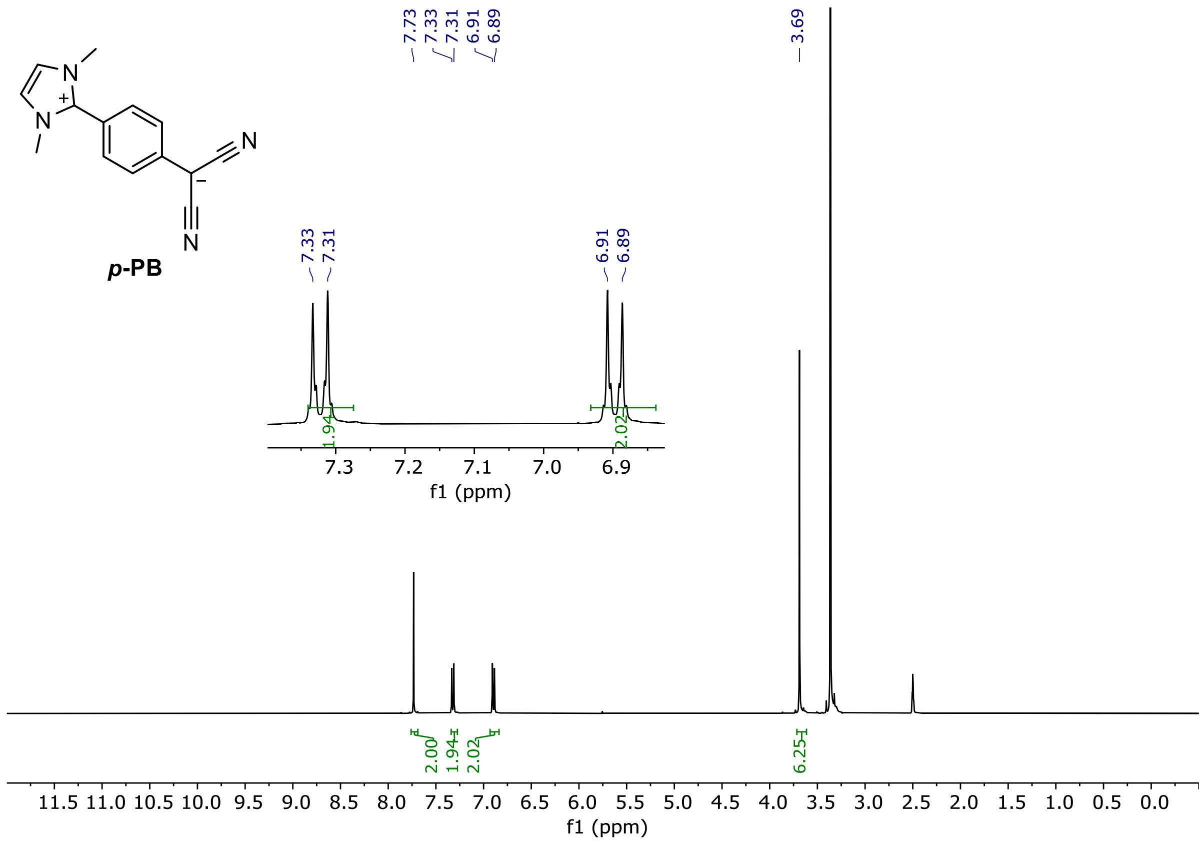

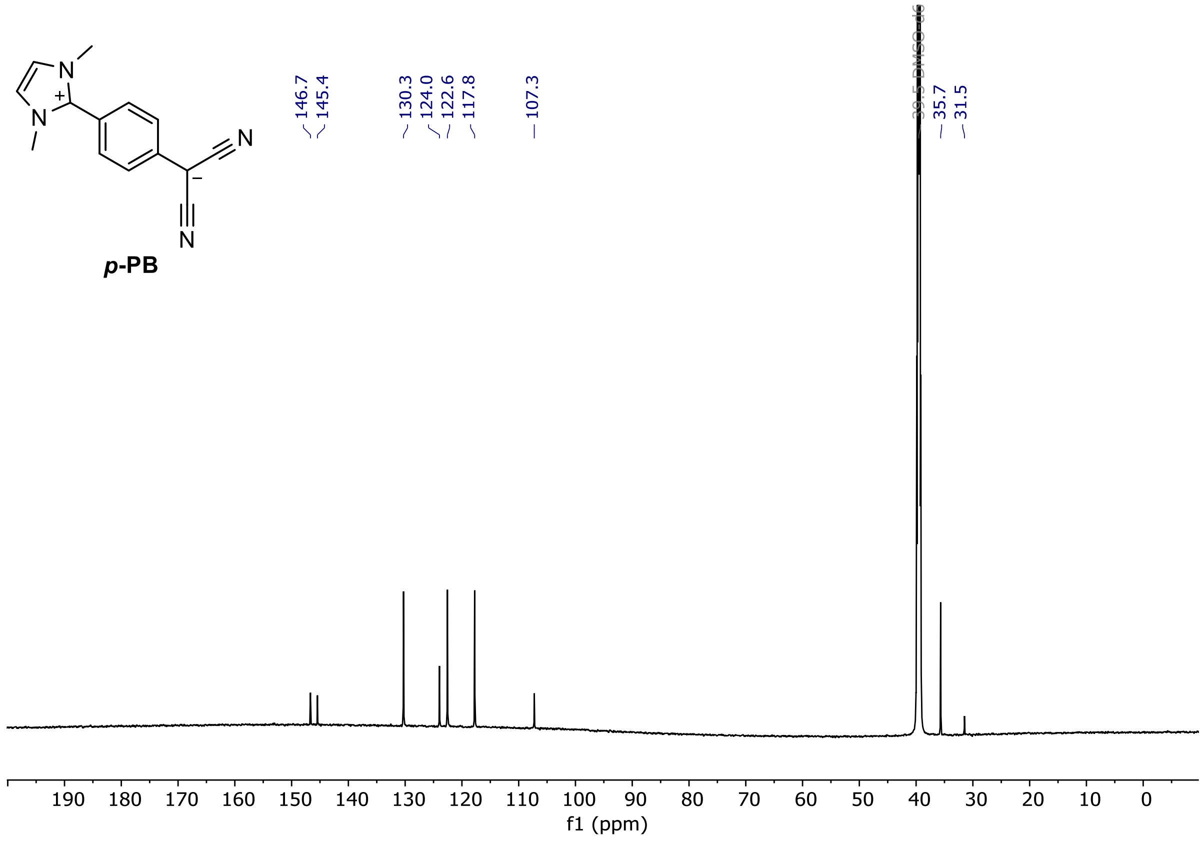

ESI-MS spectrum (positive mode) of *para*-phenylene betaine ***p*-PB** as the $[M+H]^+$ and $[2M+H]^+$ complexes at 237.1130 and 473.2191 m/z.

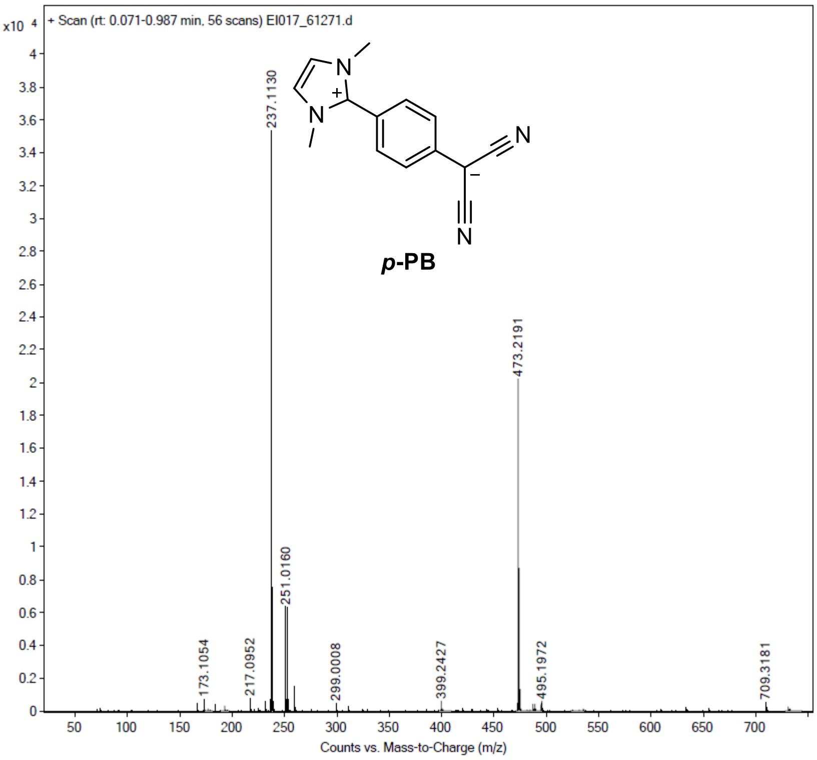

^{1}H NMR (400 MHz, DMSO-d_6, 298 K) and for mixture of 2-(2-naphthyl)-1-*H*-imidazole **15** and 2-(2-naphthyl)-1-methylimidazole **16.**

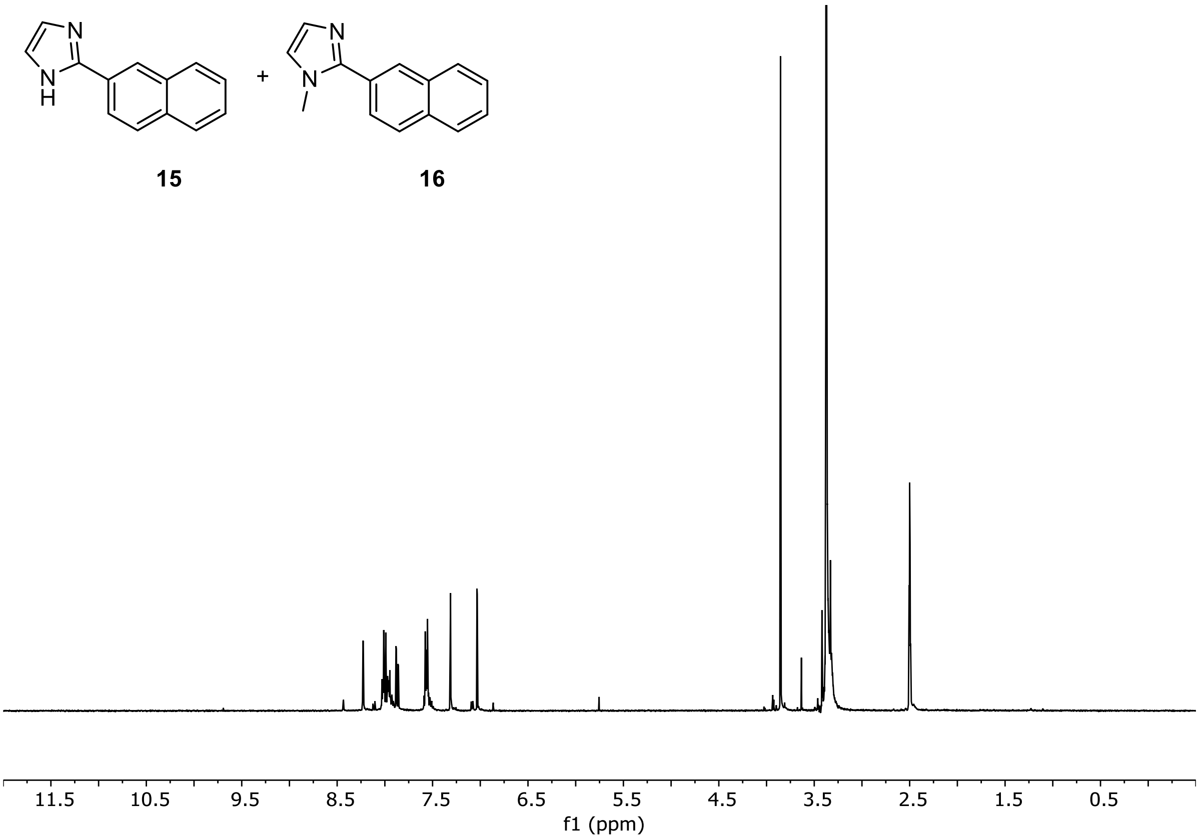

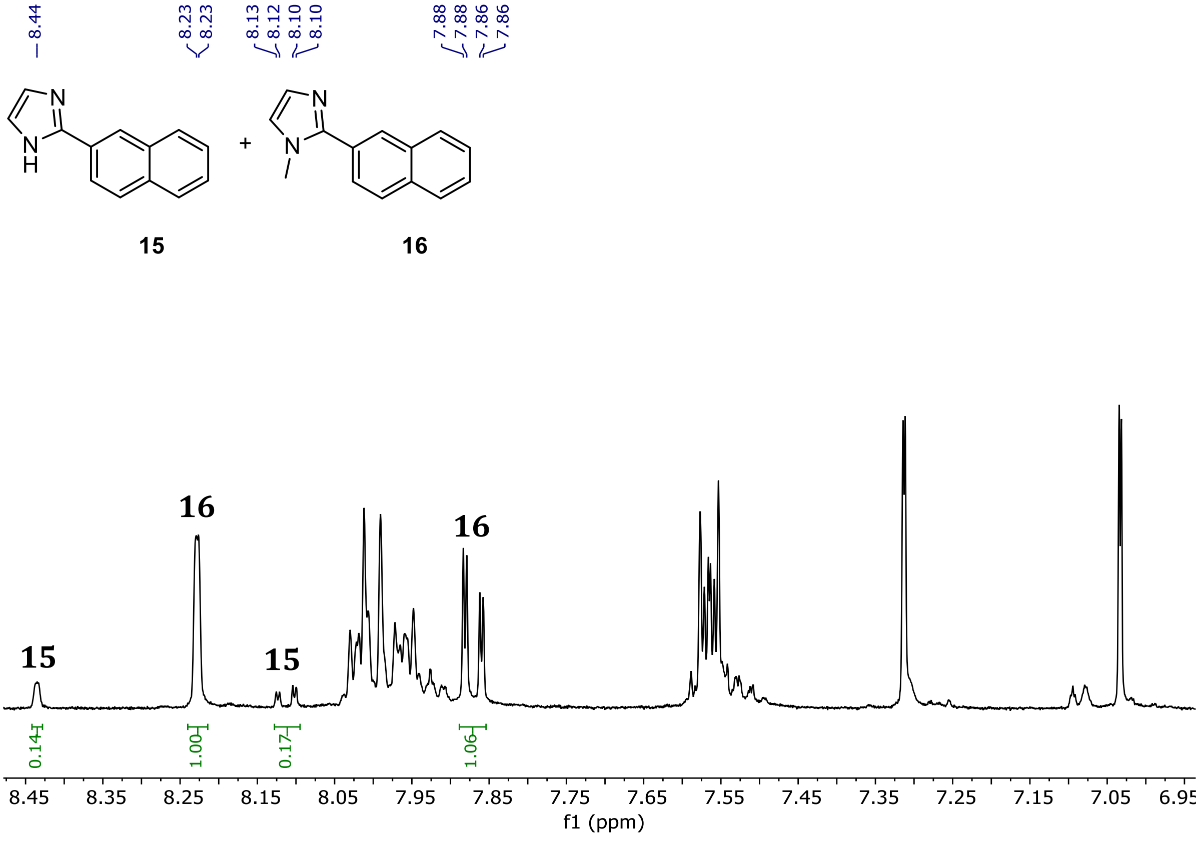

^{1}H NMR (500 MHz, DMSO-d_6, 298 K, top) and $^{13}C\{^{1}H\}$ NMR (151 MHz, DMSO-d_6, 298 K, bottom) for 1,3-dimethyl-2-(2-naphthyl)imidazolium iodide **17**.

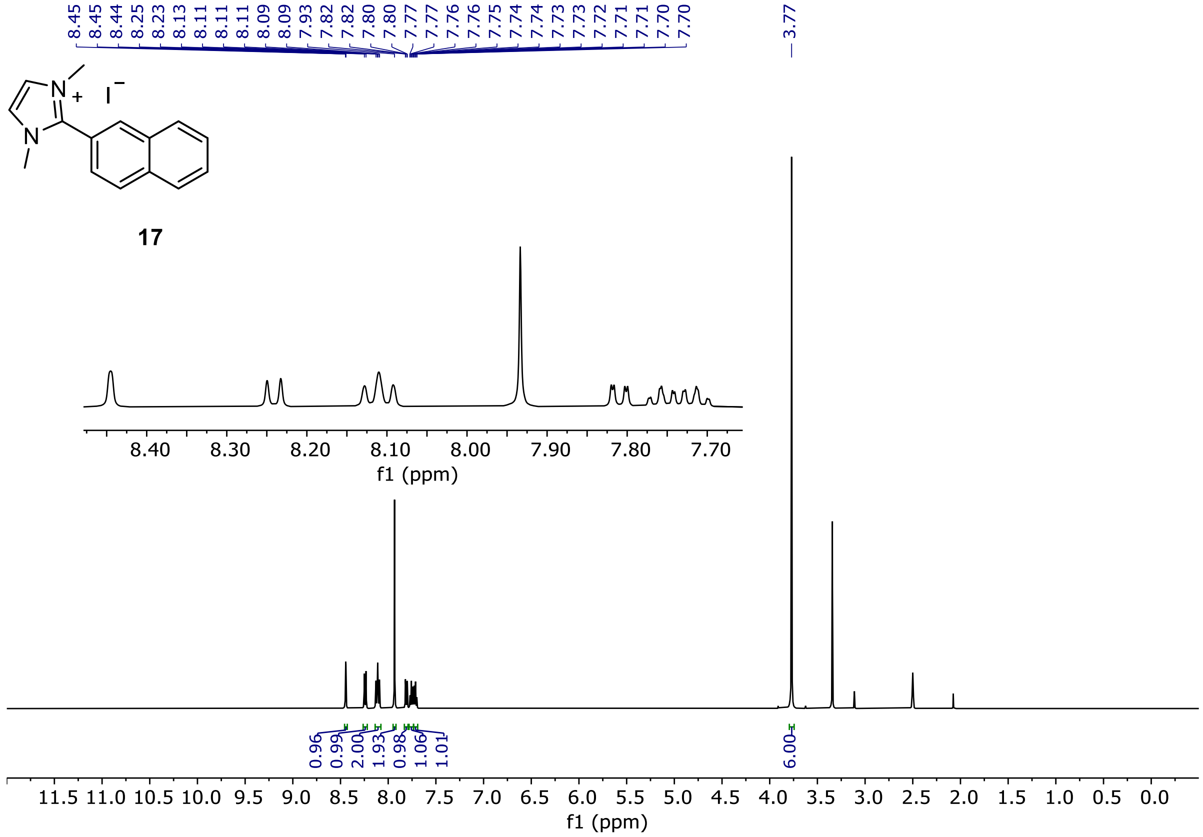

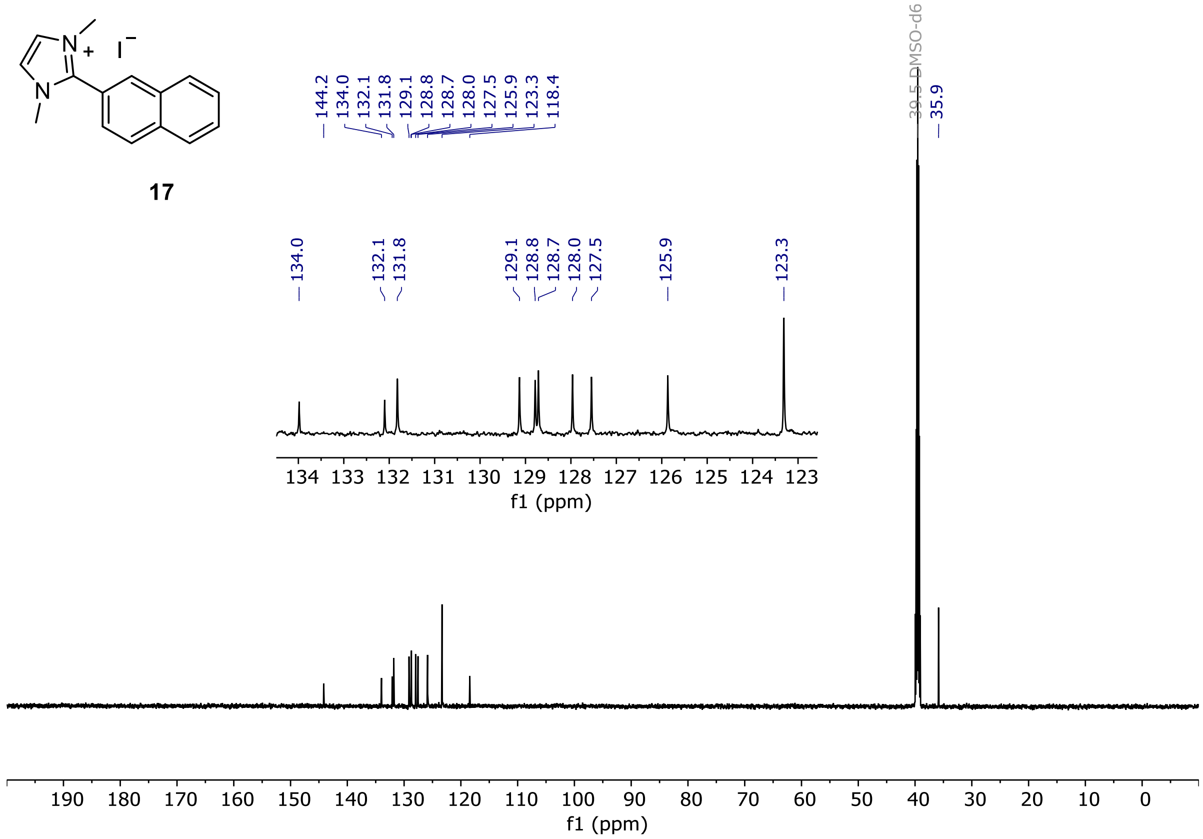

ESI-MS spectrum (positive mode) of 1,3-dimethyl-2-(2-naphthyl)imidazolium iodide **17** as the [M]+ complex at 223.1229 m/z.

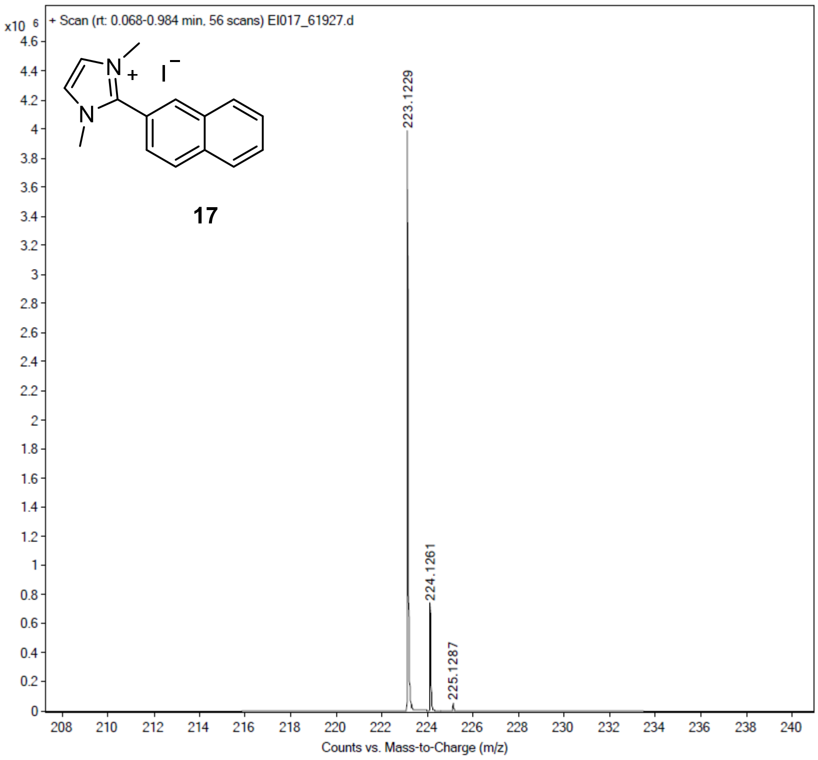

^{1}H NMR (400 MHz, DMSO-d_6, 298 K) and for mixture of 2-(7-bromo-2-naphthyl)-1-*H*-imidazole **24** and 2-(7-bromo-2-naphthyl)-1-methylimidazole **23**.

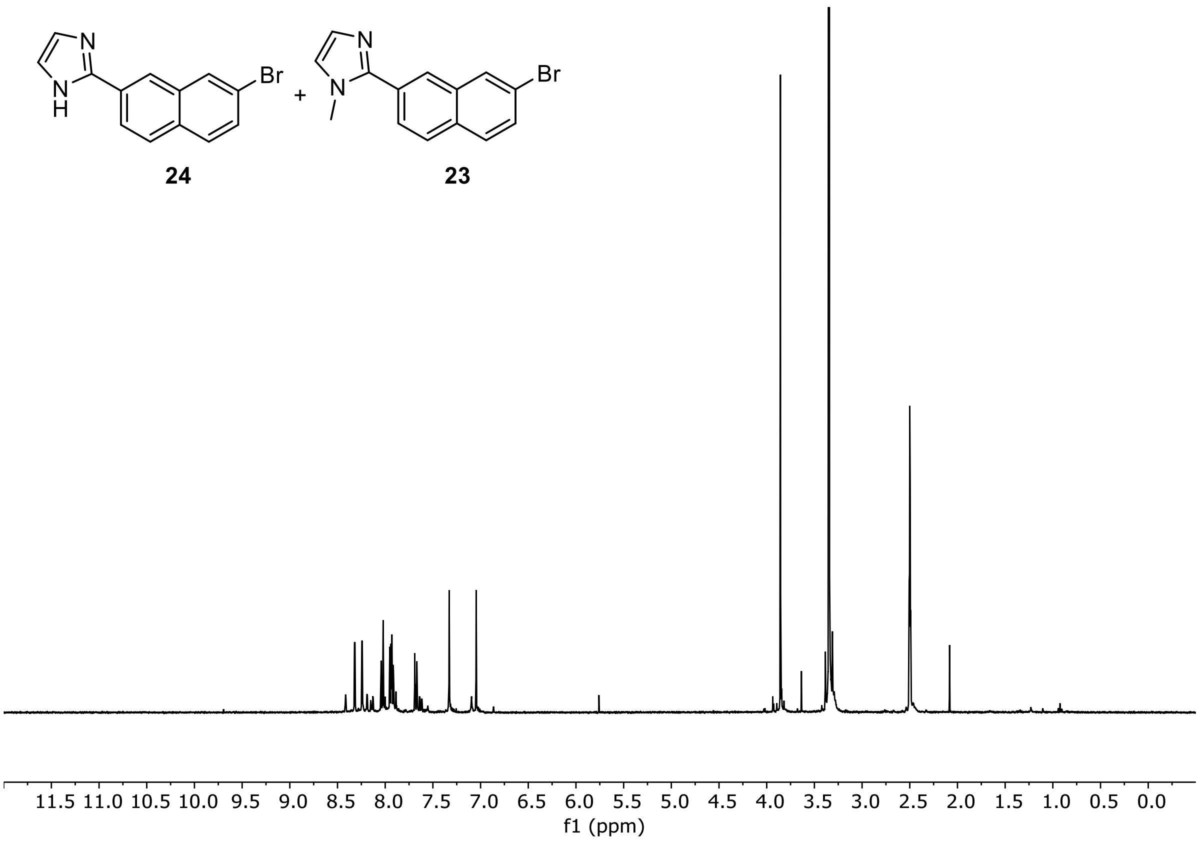

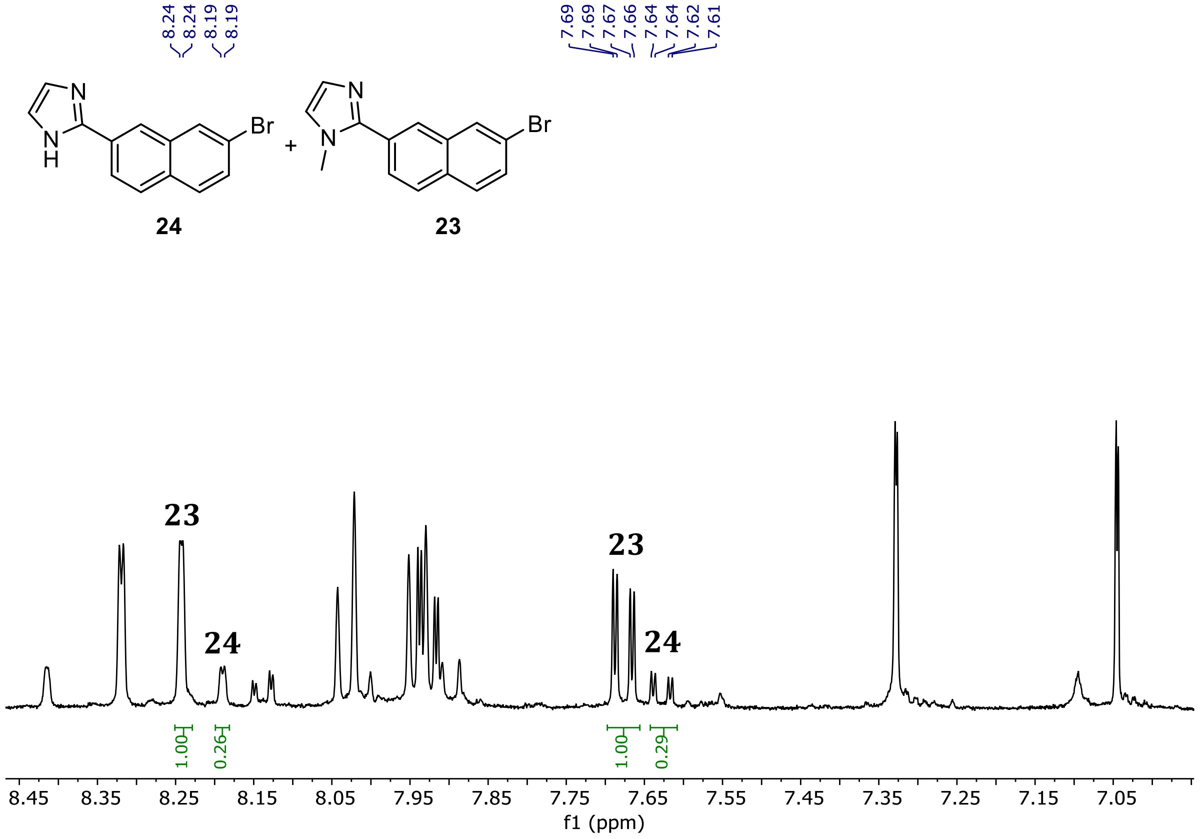

^{1}H NMR (500 MHz, DMSO-d_6, 298 K, top) and $^{13}C\{^{1}H\}$ NMR (151 MHz, DMSO-d_6, 298 K, bottom) for 2-(7-bromo-2-naphthyl)-1,3-dimethylimidazolium iodide **25.**

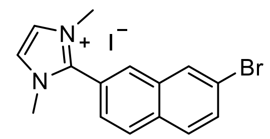

25

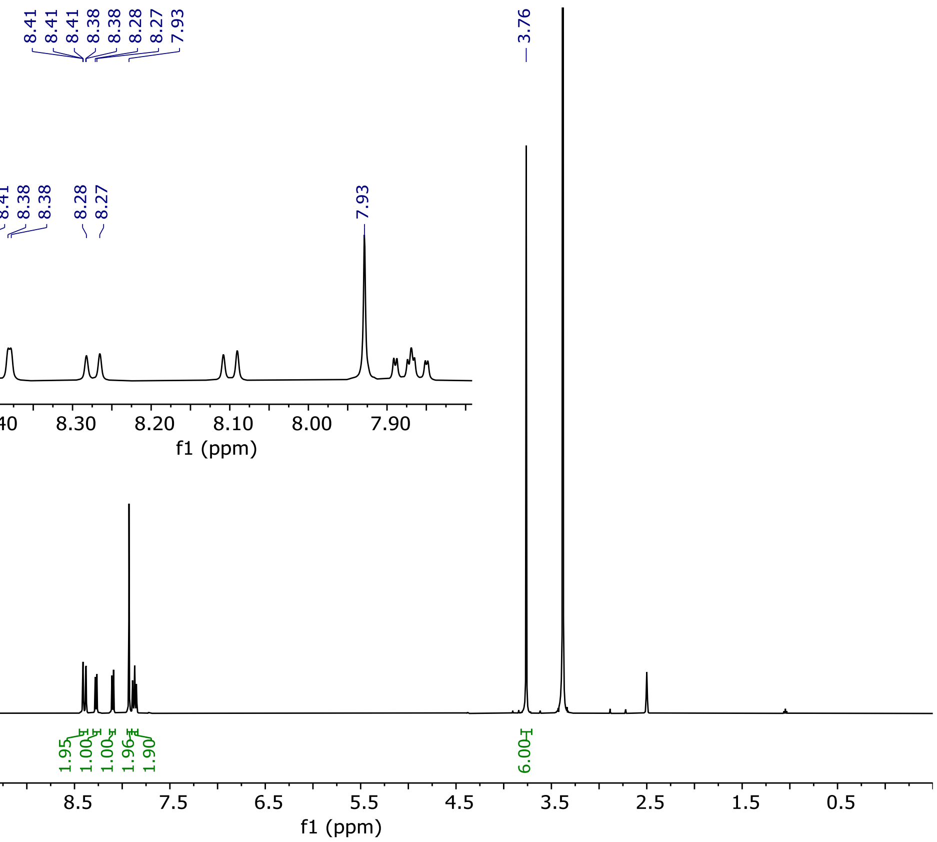

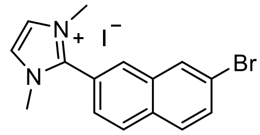

25

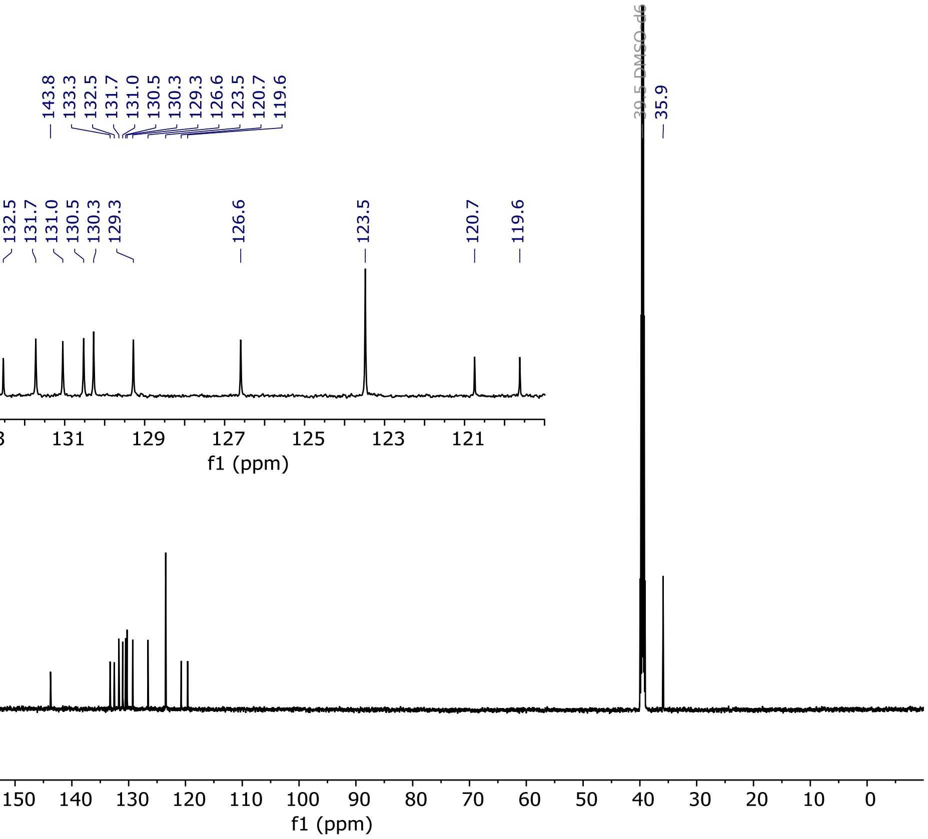

ESI-MS spectrum (positive mode) of 2-(7-bromo-2-naphthyl)-1,3-dimethylimidazolium iodide **25** as the [M]+ complex at 301.0334 and 303.0315 m/z.

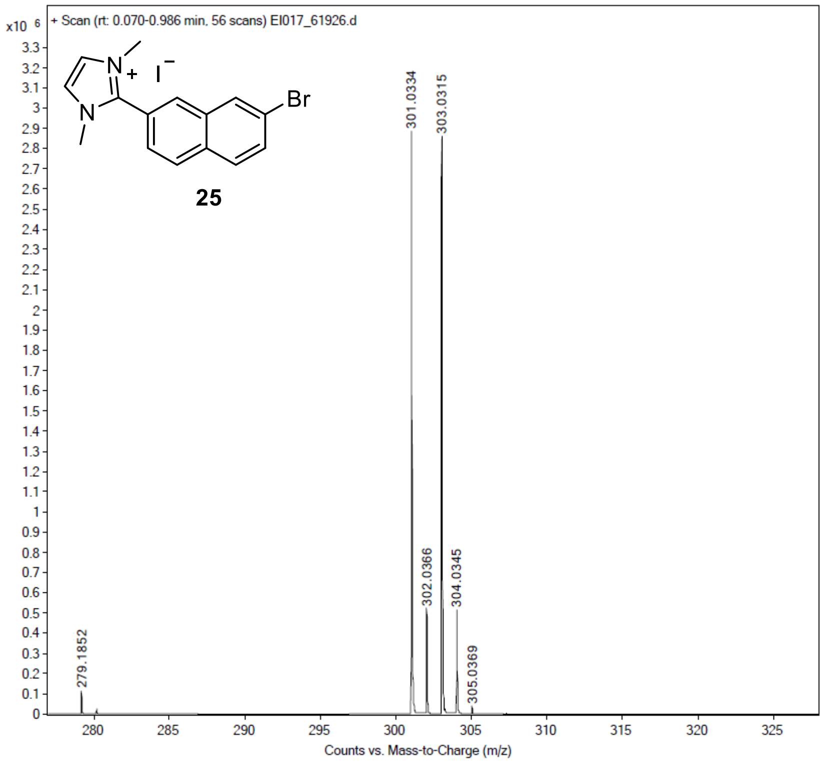

^{1}H NMR (700 MHz, DMSO-d_6, 298 K, top) and ^{13}C{^{1}H} NMR (151 MHz, DMSO-d_6, 298 K, bottom) for pseudo-*meta*-naphthylene betaine **pseudo-*m*-NB.**

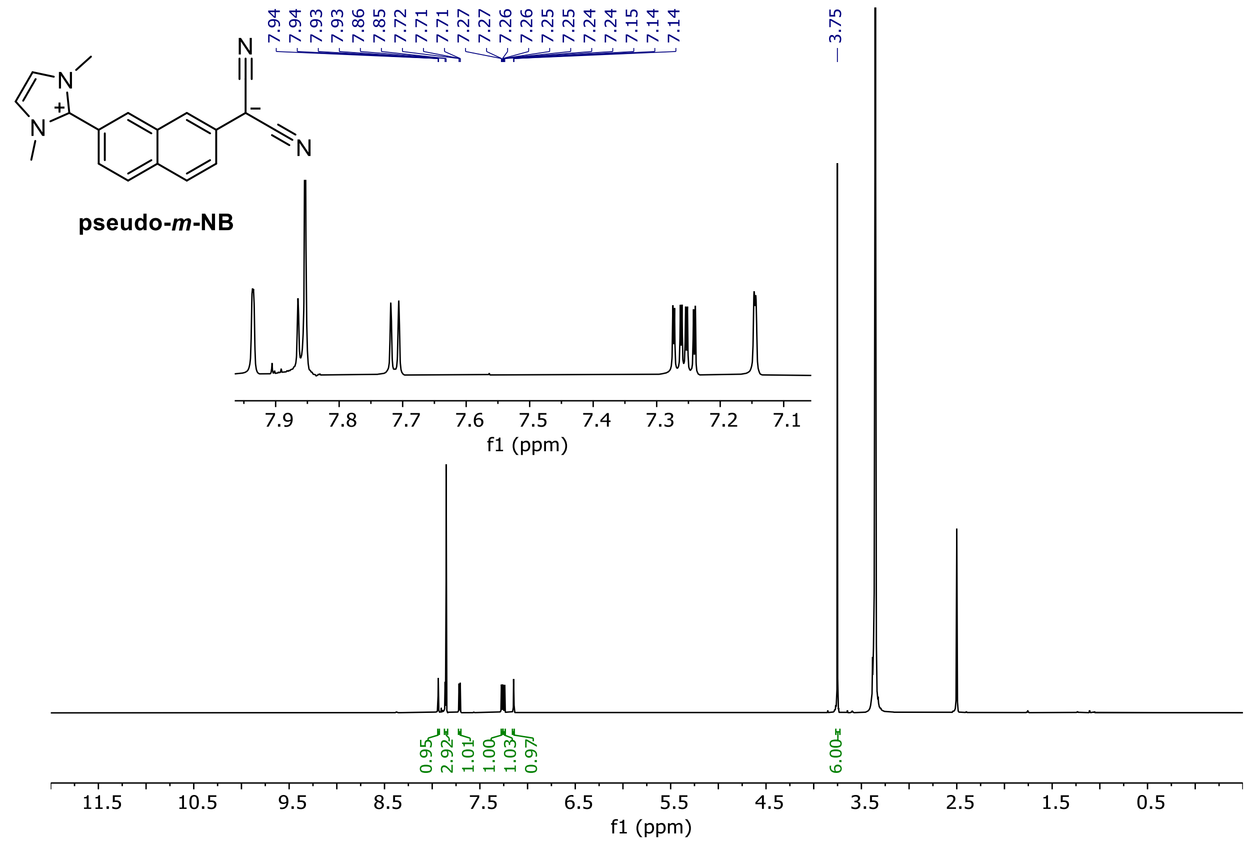

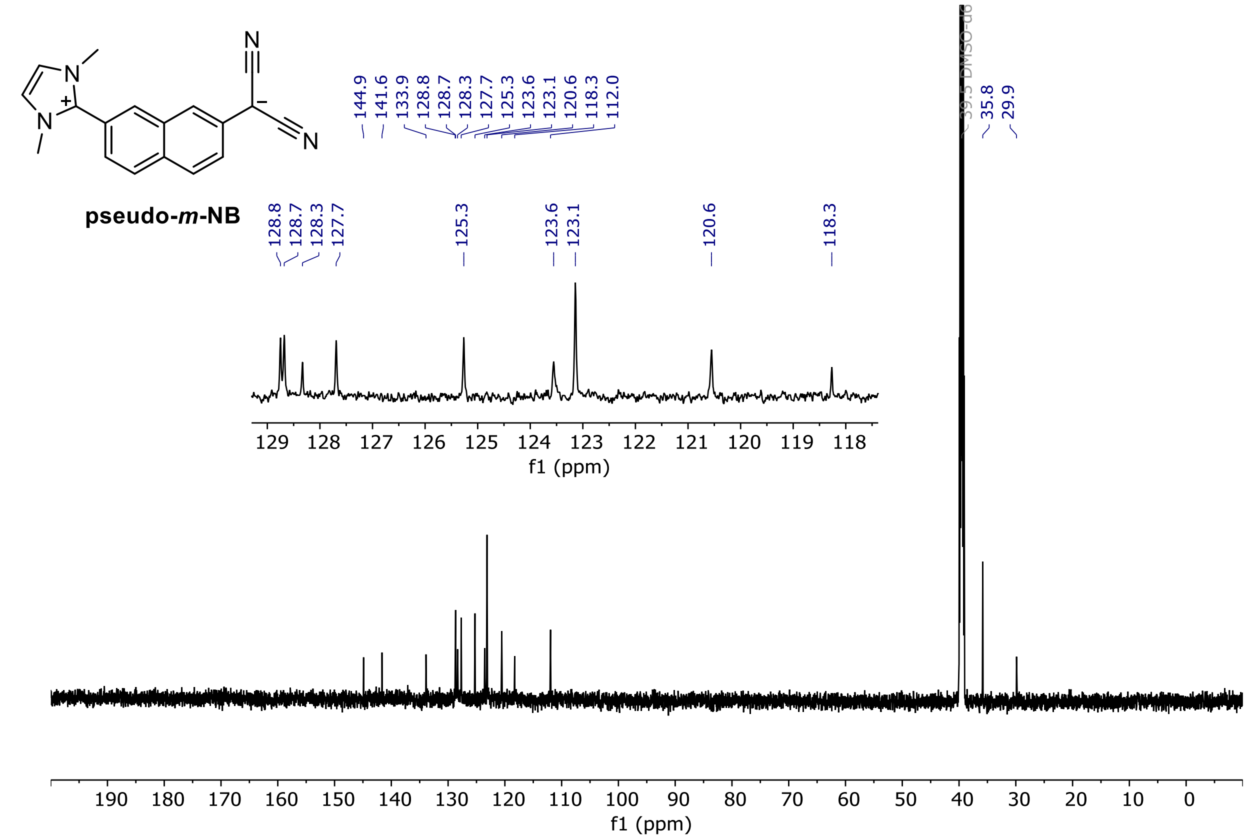

ESI-MS spectrum (positive mode) of pseudo-*meta*-naphthylene betaine **pseudo-*m*-NB** as the $[M+H]^+$ and $[M+Na]^+$ complexes at 287.1288 and 309.1115 m/z.

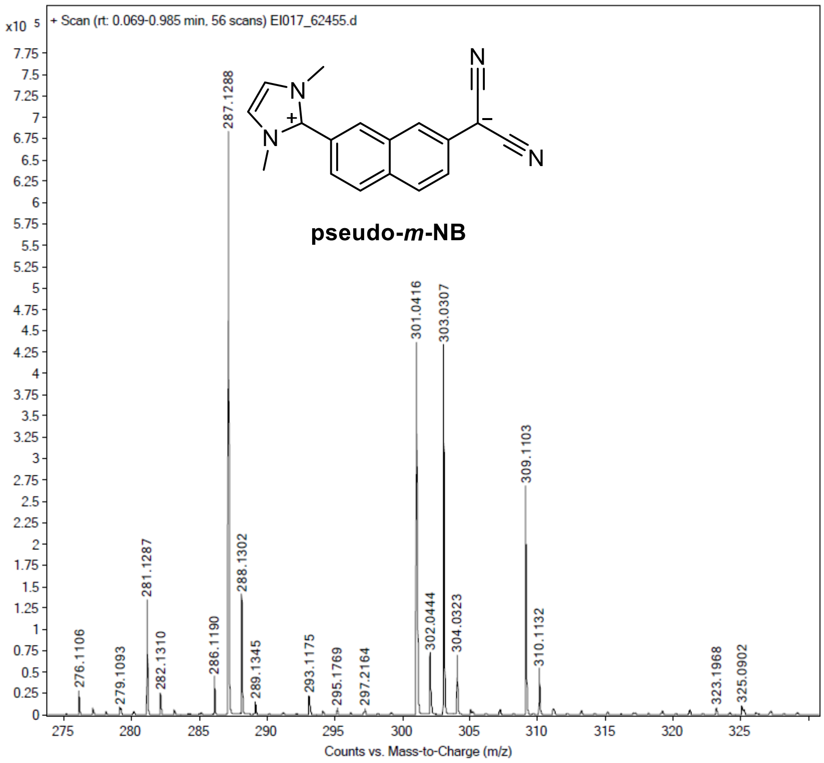

^{1}H NMR (500 MHz, DMSO-d_6, 298 K, top) and ^{13}C{^{1}H} NMR (151 MHz, DMSO-d_6, 298 K, bottom) for 2-(6-bromo-2-naphthyl)-1,3-dimethylimidazolium iodide **30**.

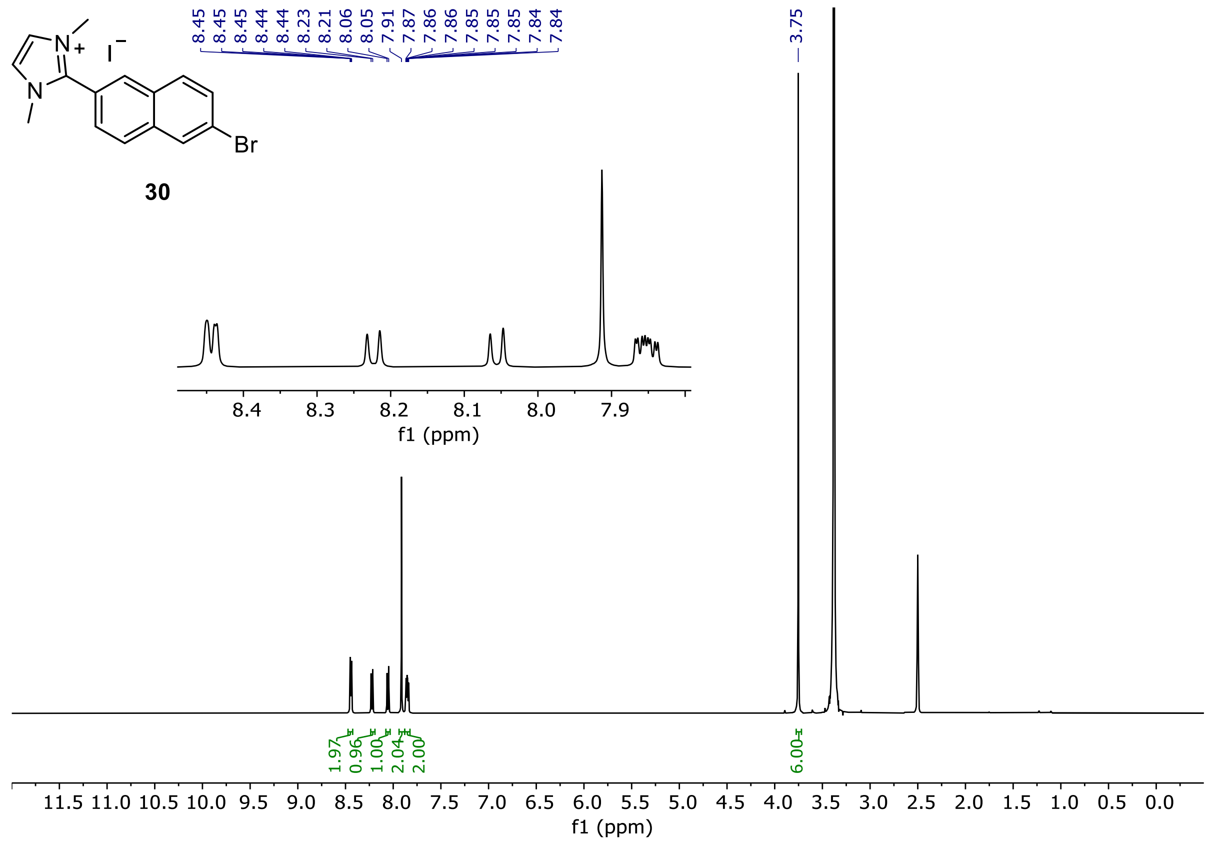

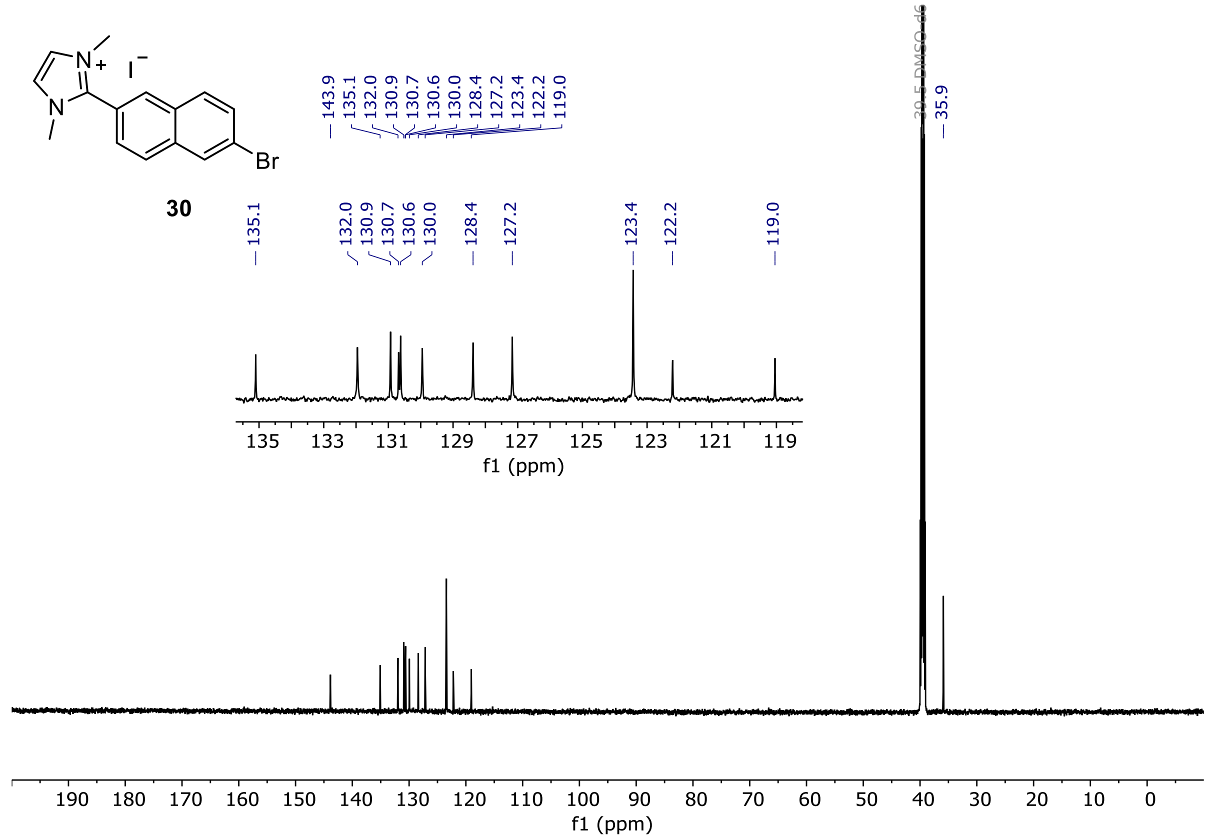

ESI-MS spectrum (positive mode) of 2-(6-bromo-2-naphthyl)-1,3-dimethylimidazolium iodide **30** as the $[M]^+$ complex at 301.0394 and 303.0378 m/z.

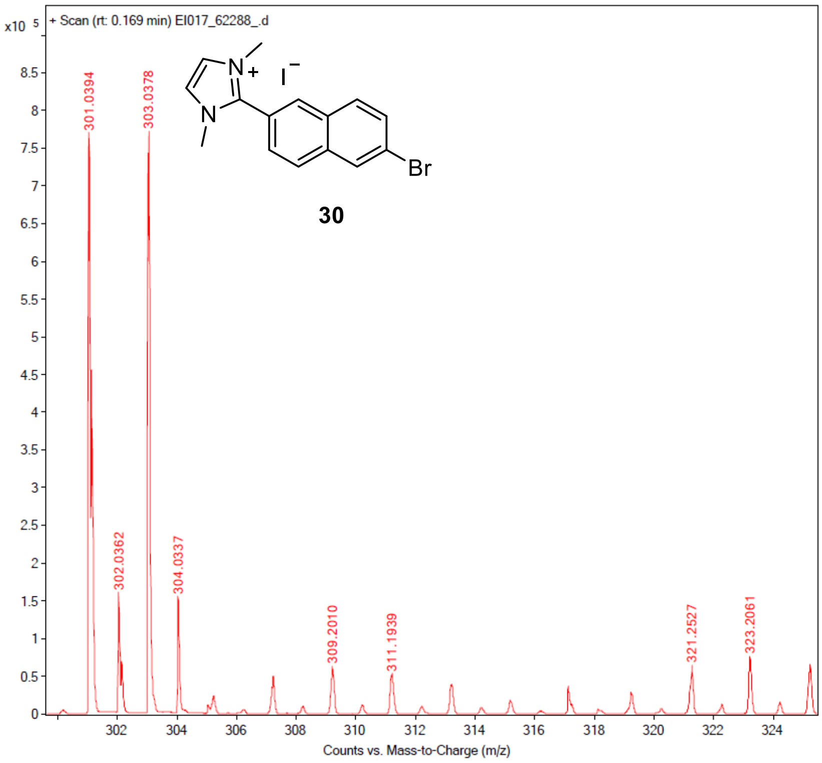

^{1}H NMR (500 MHz, DMSO-d_6, 298 K, top) and ^{13}C{^{1}H} NMR (176 MHz, DMSO-d_6, 298 K, bottom) for pseudo-*para*-naphthylene betaine **pseudo-*p*-NB**.

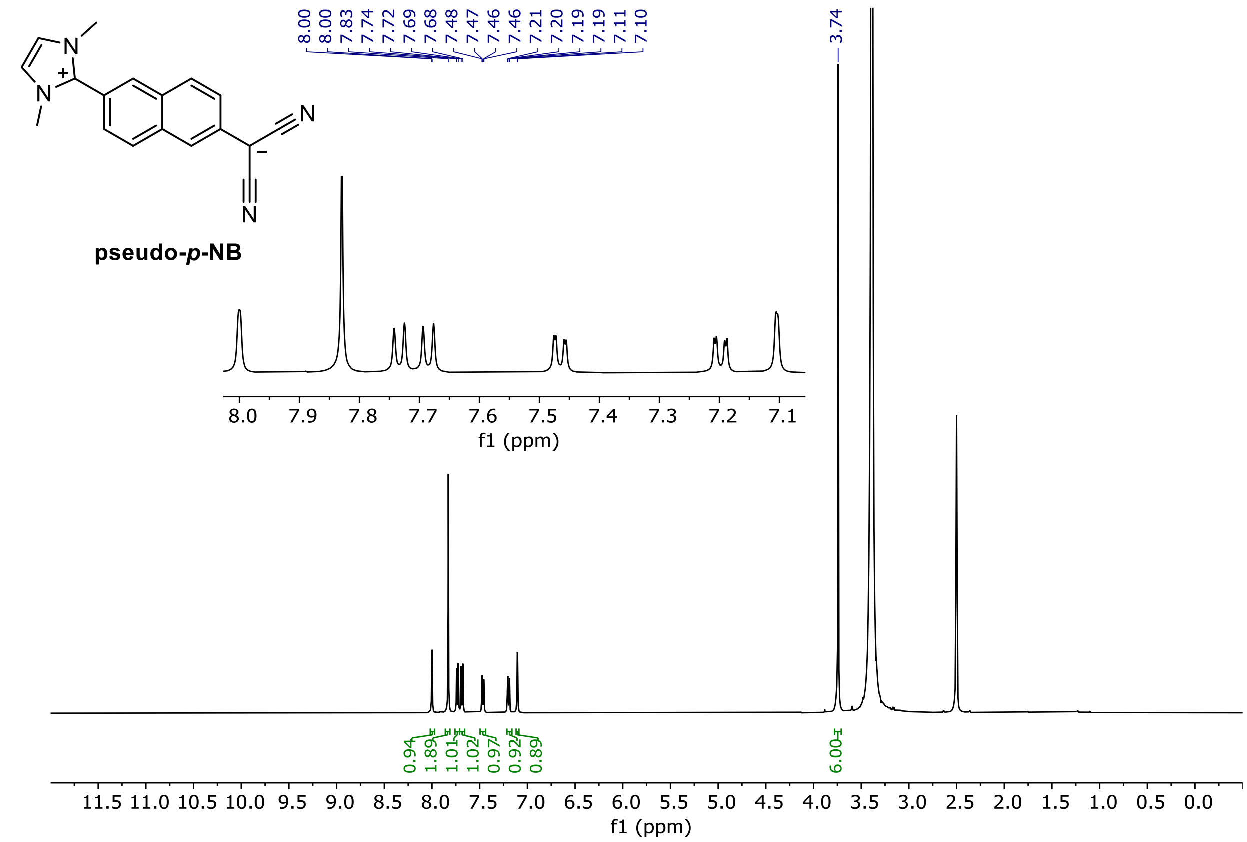

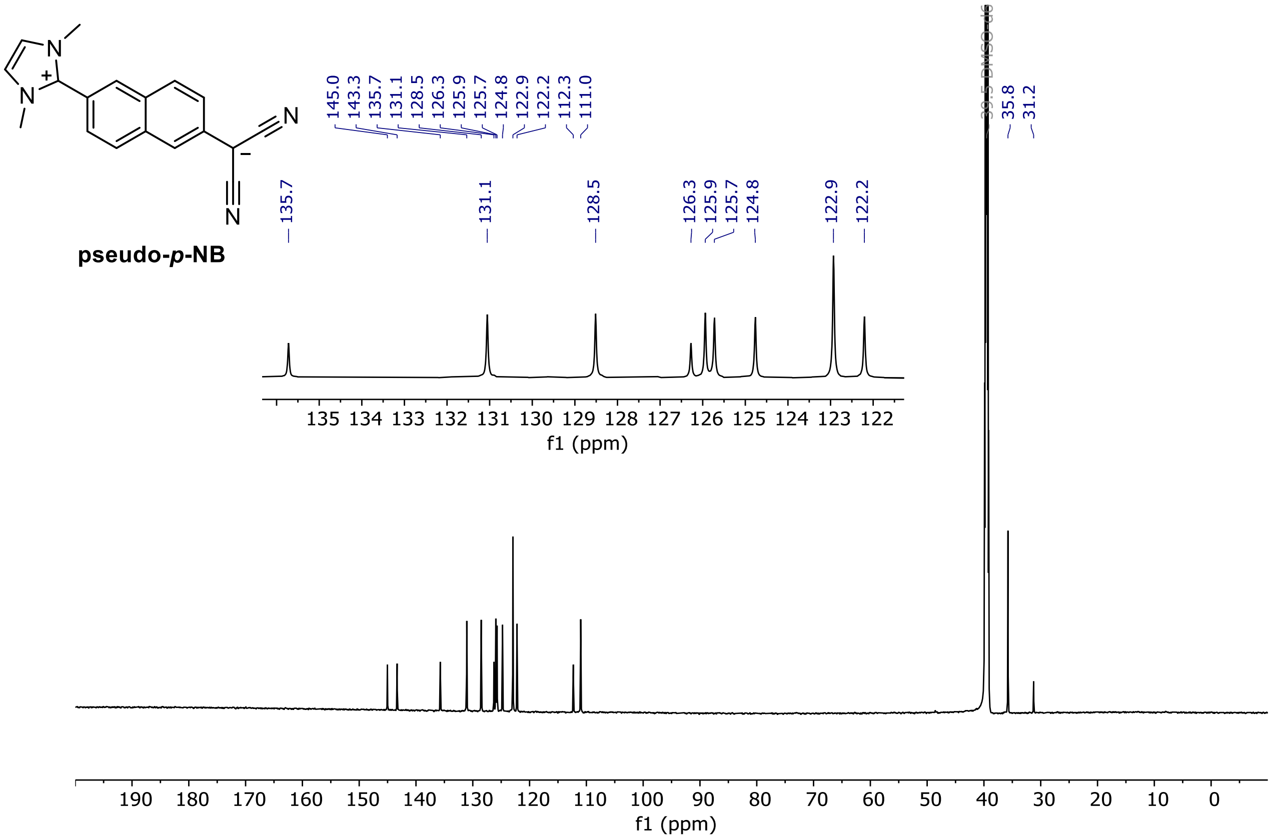

ESI-MS spectrum (positive mode) of pseudo-*para*-naphthylene betaine **pseudo-*p*-NB** as the $[M+H]^+$ and $[M+Na]^+$ complexes at 287.1288 and 309.1103 m/z.

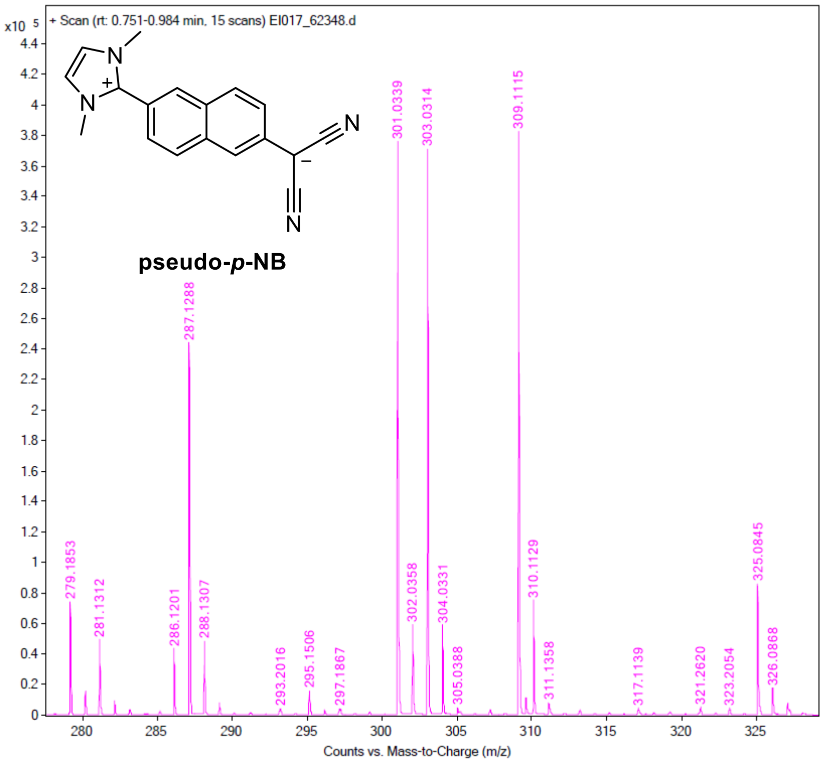

Section S5 Mesomeric Betaine Nomenclature

The following rules are applied from Ollis, Stanforth, and Ramsden.[59]

RULE 3. Pseudo-cross-conjugated mesomeric betaines are associated with dipolar canonical forms which include (i) electron octet structures, (ii) electron sextet structures with internal octet stabilisation and (iii) electron sextet structures without internal octet stabilisation. The dipolar canonical forms do provide common sites for formal positive and negative charges.

RULE 5. If (i) the delocalised negative charge of the heterocyclic mesomeric betaine is associated with a fragment which is isoconjugate with an odd alternant hydrocarbon anion, (ii) this fragment is connected through its unstarred positions to the remainder of the x-electron system of the molecule, and (iii) this fragment does not contain the heteroatom accommodating the positive charge, then the heterocyclic mesomeric betaine is either cross-conjugated or pseudo-cross-conjugated.

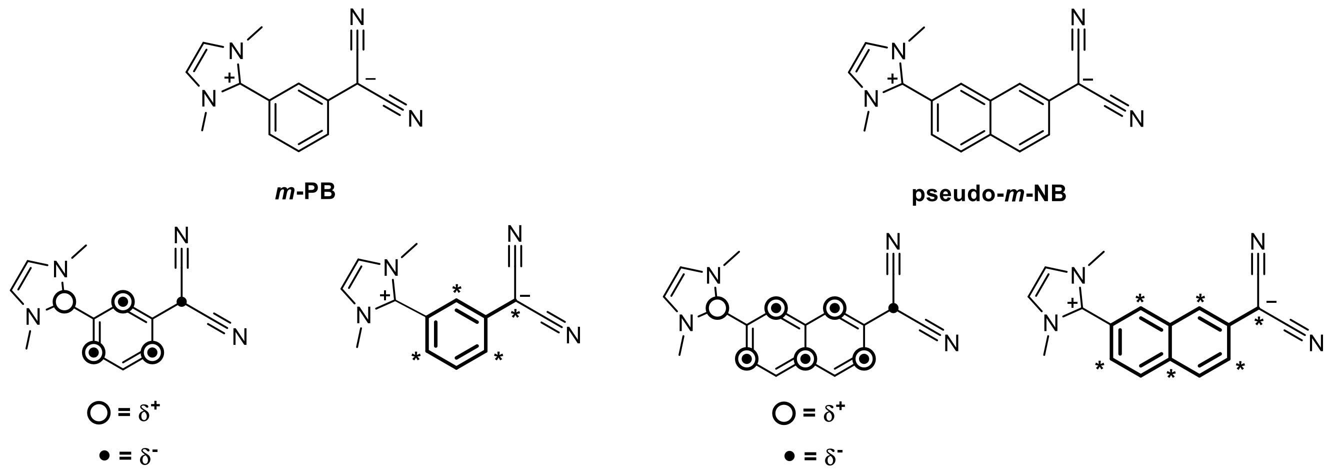

Figure S1. Demonstration that ***m*-PB** and **pseudo-*m*-NB** obey the rules for pseudo-cross-conjugated mesomeric betaines. They possess shared sites for formal positive and negative charge delocalisation (Rule 3) and that the negatively charged fragments come from odd-alternant hydrocarbon fragments which are attached by unstarred atoms to the positively charged fragments (Rule 5).

Compounds ***p*-PB** and **pseudo-*p*-NB** can be drawn as neutral Kekulé structures by virtue of their *para*-substitution, which technically excludes them from mesomeric betaine nomenclature, although they are still named as betaines because literature, experimental, and computational evidence supports the significant charge-separation which is characteristic of betaines.

Section S6 X-ray Crystallography

- Crystals of ***m*-PB** for diffraction were grown by allowing a solution containing ~1 mg/mL in MeOH to slowly evaporate over 24 hours.
- Crystals of ***p*-PB** for diffraction were grown by slow vapour diffusion of either diethyl ether or dichloromethane into a solution by dissolving ~1 mg in just enough acetonitrile or dimethylformamide for full dissolution.
- Crystals of **pseudo-*m*-NB** for diffraction were grown by slow vapour diffusion of dichloromethane into a solution made from ~1 mg in 10 drops of dimethylformamide or dimethylsulfoxide.
- Crystals of **pseudo-*p*-NB** for diffraction were grown (as twins) by slow vapour diffusion of diethyl ether into a nearly saturated solution of dimethylformamide.

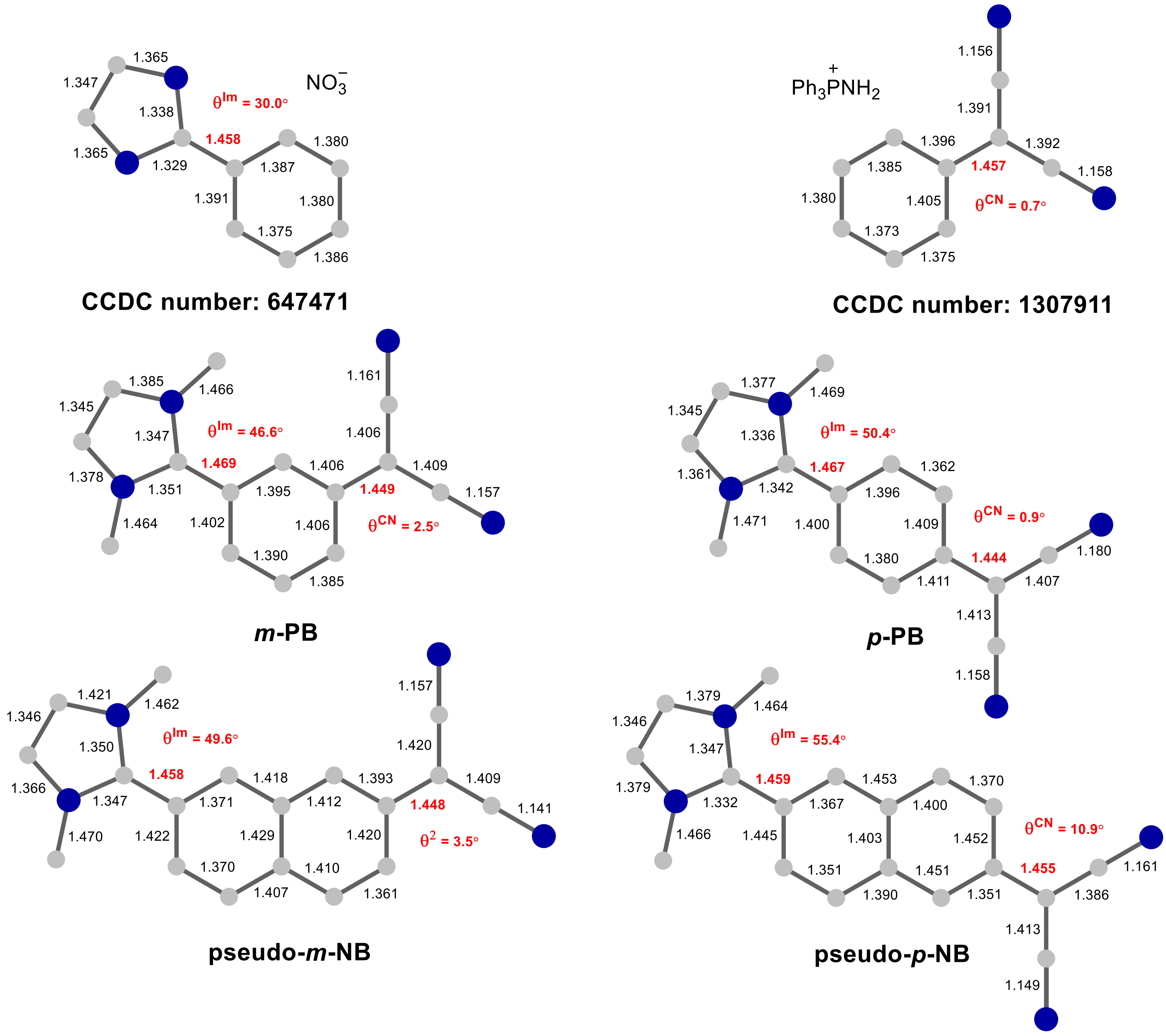

Figure S2. Crystal structures of reference and synthesised compounds with labelled bond lengths.

Table S6. Diagnostic bond lengths and dihedral angles for reference and synthesised compounds highlighted above in red.

Compound	Im^+ — Aryl bond (Å)	CN_2^- — Aryl bond (Å)	Im^+ — Aryl dihedral (°)	CN_2^- — Aryl dihedral (°)
**647471**[60]	1.458	-	30.0	-
**1307911**[61]	-	1.457	-	0.7
***m*-PB**	1.469	1.449	46.6	2.5
***p*-PB**	1.467	1.444	50.4	0.9
**pseudo-*m*-NB**	1.458	1.448	49.6	3.5
**pseudo-*p*-NB**	1.459	1.455	55.4	10.9

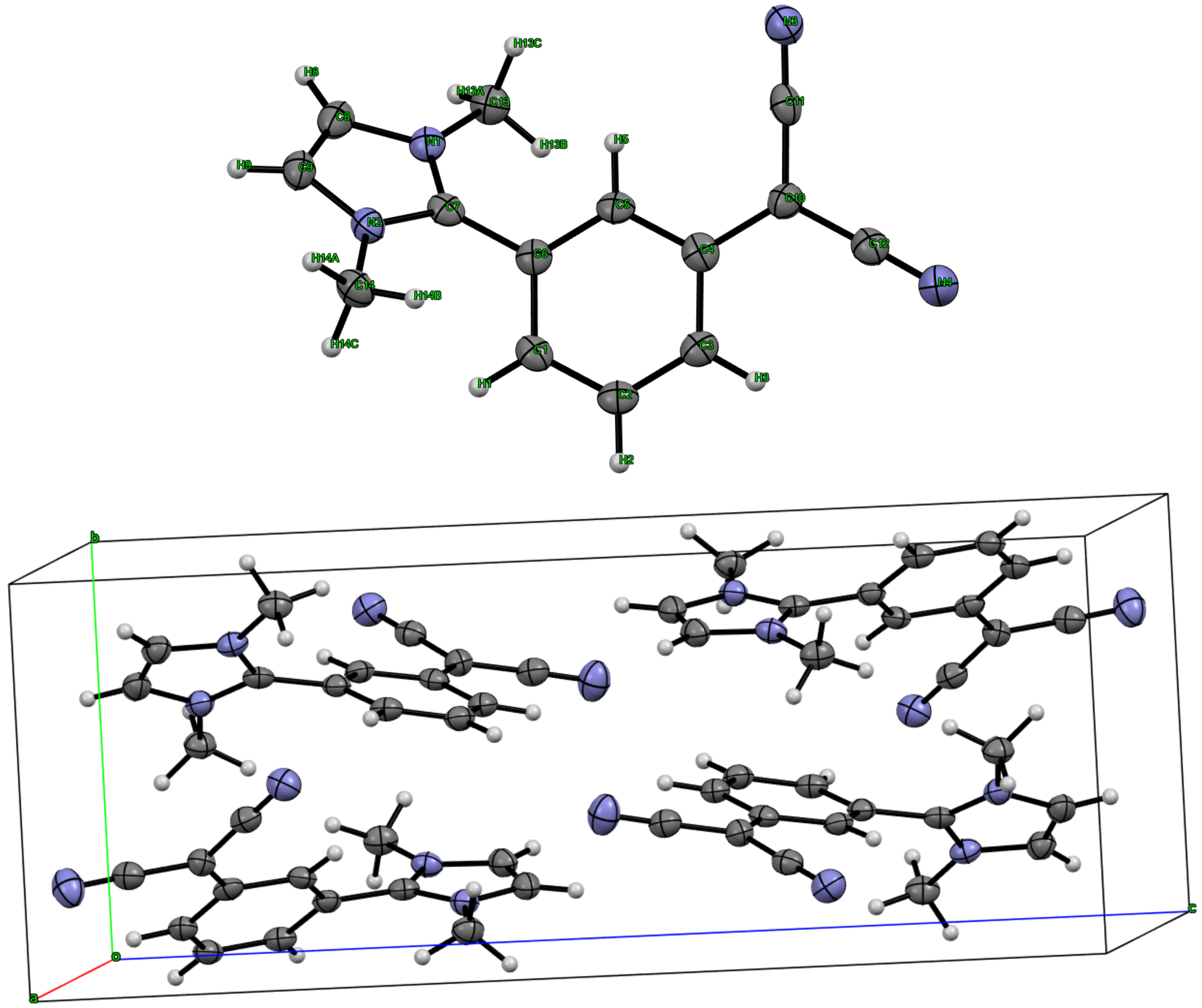

Figure S3. Ellipsoid representation of ***m*-PB** with 66% thermal probability ellipsoids, and the packing in one unit cell along the principal crystallographic axes. CCDC Number: 2494028

Table S7. Crystal data and structural refinement for ***m*-PB**

Bond precision: C-C = 0.0030 A Wavelength=1.54178

Cell: a=8.0059(3) b=7.6326(3) c=19.6494(8)
alpha=90 beta=96.704(3) gamma=90

Temperature: 100 K

	Calculated	Reported
Volume	1192.48(8)	1192.48(8)
Space group	P 21/c	P 21/c
Hall group	-P 2ybc	-P 2ybc
Moiety formula	C14 H12 N4	?
Sum formula	C14 H12 N4	C14 H12 N4
Mr	236.28	236.28
Dx,g cm-3	1.316	1.316
Z	4	4
Mu (mm-1)	0.657	0.657
F000	496.0	496.0
F000’	497.38	
h,k,lmax	9,9,23	9,9,23
Nref	2128	2076
Tmin,Tmax	0.881, 0.955	0.411, 0.889
Tmin’	0.706	

Correction method= # Reported T Limits: Tmin=0.411 Tmax=0.889
AbsCorr = MULTI-SCAN
Data completeness= 0.976 Theta(max)= 66.978
R(reflections)= 0.0593(1809)
wR2(reflections)= 0.1425(2076)
S = 1.079 Npar= 165

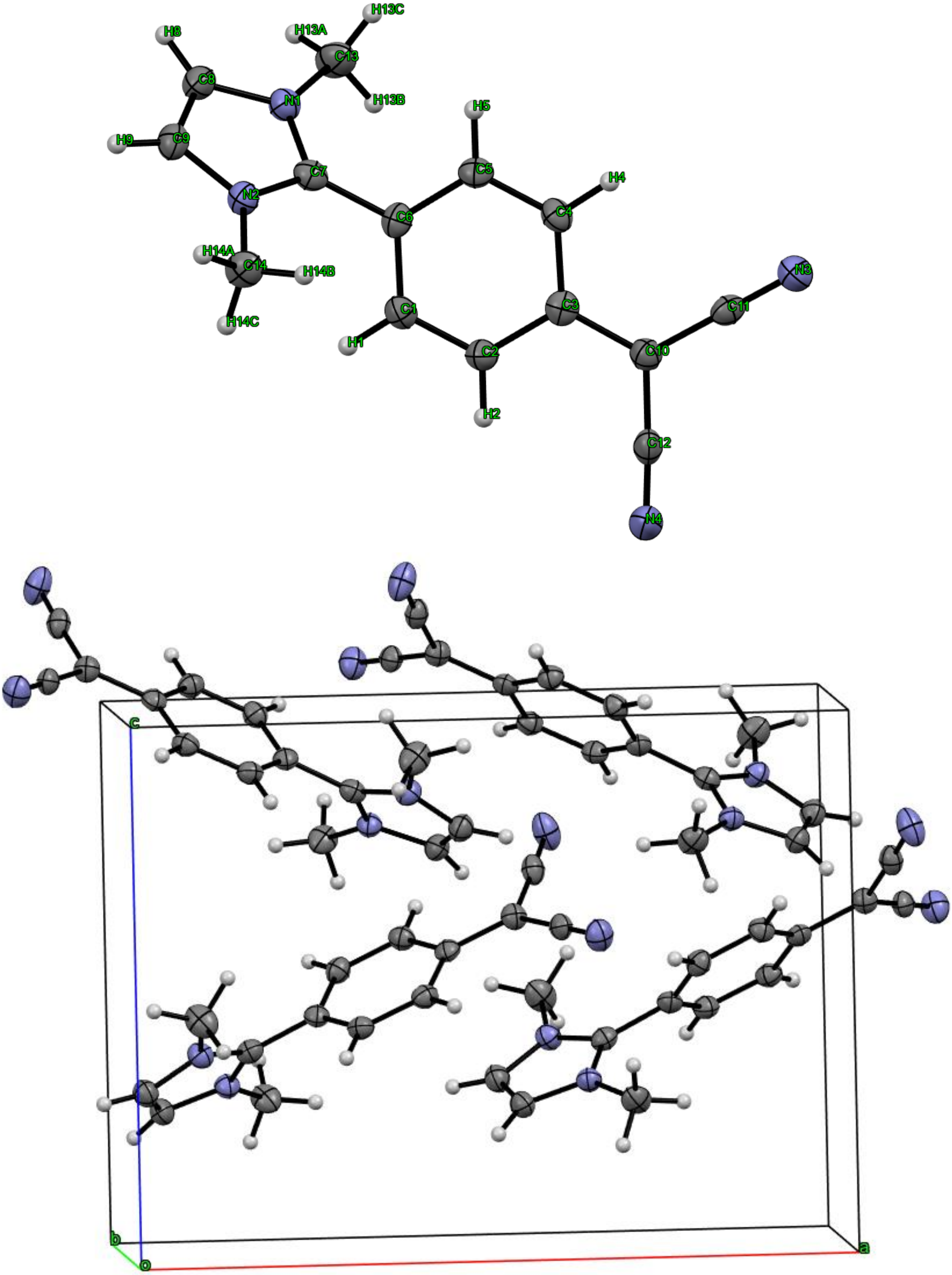

Figure S4. Ellipsoid representation of ***p*-PB** with 66% thermal probability ellipsoids, and the packing in one unit cell along the principal crystallographic axes. CCDC Number: 2494029

Table S8. Crystal data and structural refinement for ***p*-PB**

Bond precision: C-C = 0.0030 A Wavelength=1.54178

Cell:	a=13.90861(9)	b=8.59323(6)	c=10.50182(7)
	alpha=90	beta=90	gamma=90

Temperature: 100 K

	Calculated	Reported
Volume	1255.176(15)	1255.176(15)
Space group	P n a 21	P n a 21
Hall group	P 2c -2n	P 2c -2n
Moiety formula	C14 H12 N4	?
Sum formula	C14 H12 N4	C14 H12 N4
Mr	236.28	236.28
Dx,g cm-3	1.250	1.250
Z	4	4
Mu (mm-1)	0.624	0.624
F000	496.0	496.0
F000’	497.38	
h,k,lmax	16,10,12	16,10,12
Nref	2315[1226]	2219
Tmin,Tmax	0.900,0.940	0.687,0.902
Tmin’	0.769	

Correction method= # Reported T Limits: Tmin=0.687 Tmax=0.902

AbsCorr = MULTI-SCAN

Data completeness= 1.81/0.96 Theta(max)= 68.105

R(reflections)= 0.0291(2176)

wR2(reflections)= 0.0761(2219)

S = 1.043 Npar= 166

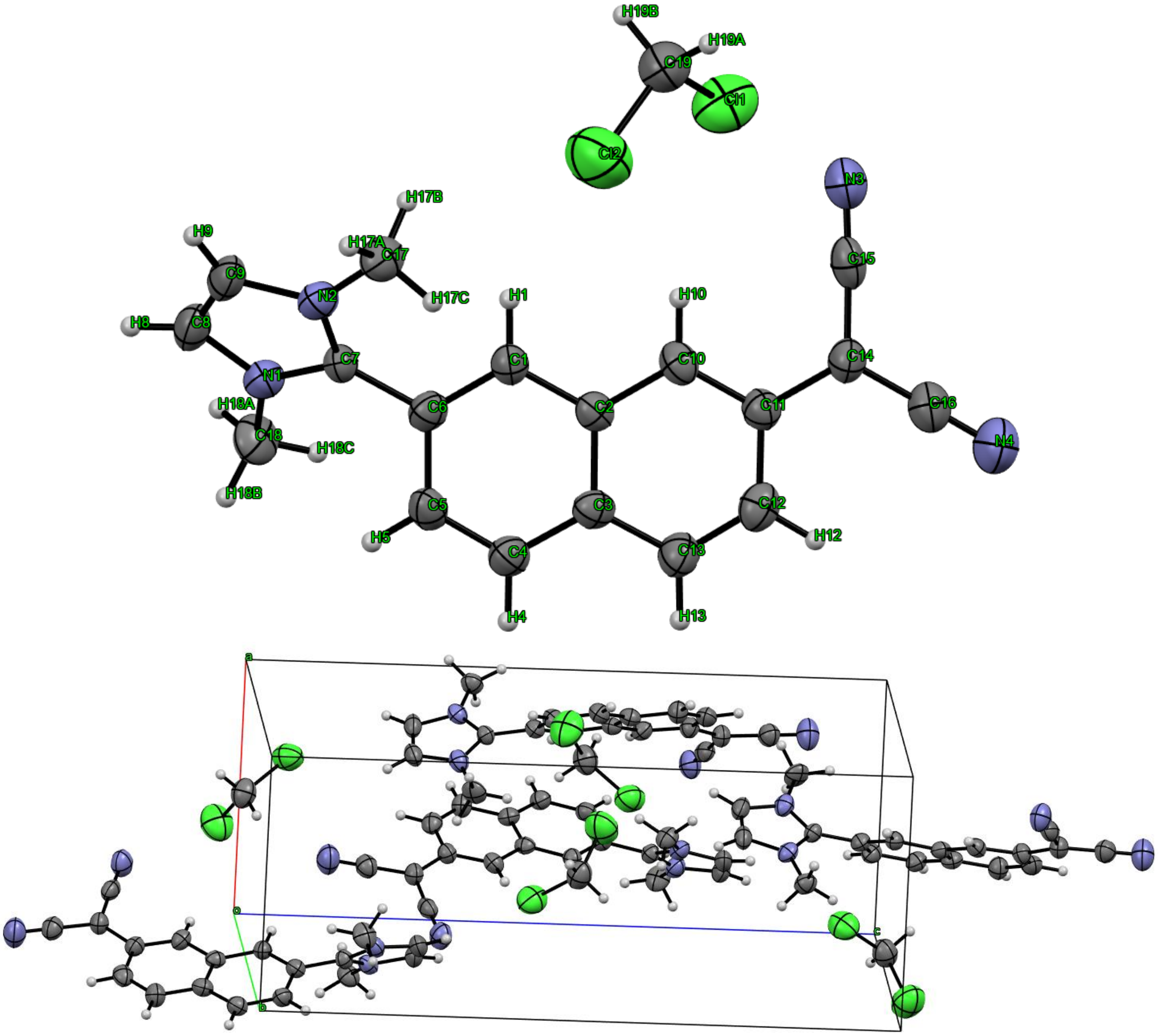

Figure S5. Ellipsoid representation of **pseudo-*m*-NB** with 66% thermal probability ellipsoids, including CH_2Cl_2, and the packing in one unit cell along the principal crystallographic axes. CCDC Number: 2494030

Table S9. Crystal data and structural refinement for **pseudo-*m*-NB**

Bond precision: C-C = 0.0076 A Wavelength=1.54178

Cell: a=7.6087(4) b=12.6810(8) c=18.7415(11)
alpha=90 beta=90 gamma=90

Temperature: 149 K

	Calculated	Reported
Volume	1808.29(18)	1808.29(18)
Space group	P 21 21 21	P 21 21 21
Hall group	P 2ac 2ab	P 2ac 2ab
Moiety formula	C18 H14 N4, C H2 Cl2	?
Sum formula	C19 H16 Cl2 N4	C19 H16 Cl2 N4
Mr	371.26	371.26
Dx,g cm-3	1.364	1.364
Z	4	4
Mu (mm-1)	3.294	3.294
F000	768.0	768.0
F000’	772.67	
h,k,lmax	9,15,22	8,15,22
Nref	3208[1858]	3052
Tmin,Tmax	0.821,0.906	0.286,0.591
Tmin’	0.130	

Correction method= # Reported T Limits: Tmin=0.286 Tmax=0.591
AbsCorr = MULTI-SCAN
Data completeness= 1.64/0.95 Theta(max)= 66.730
R(reflections)= 0.0515(2177)
wR2(reflections)= 0.1234(3052)
S = 1.037 Npar= 229

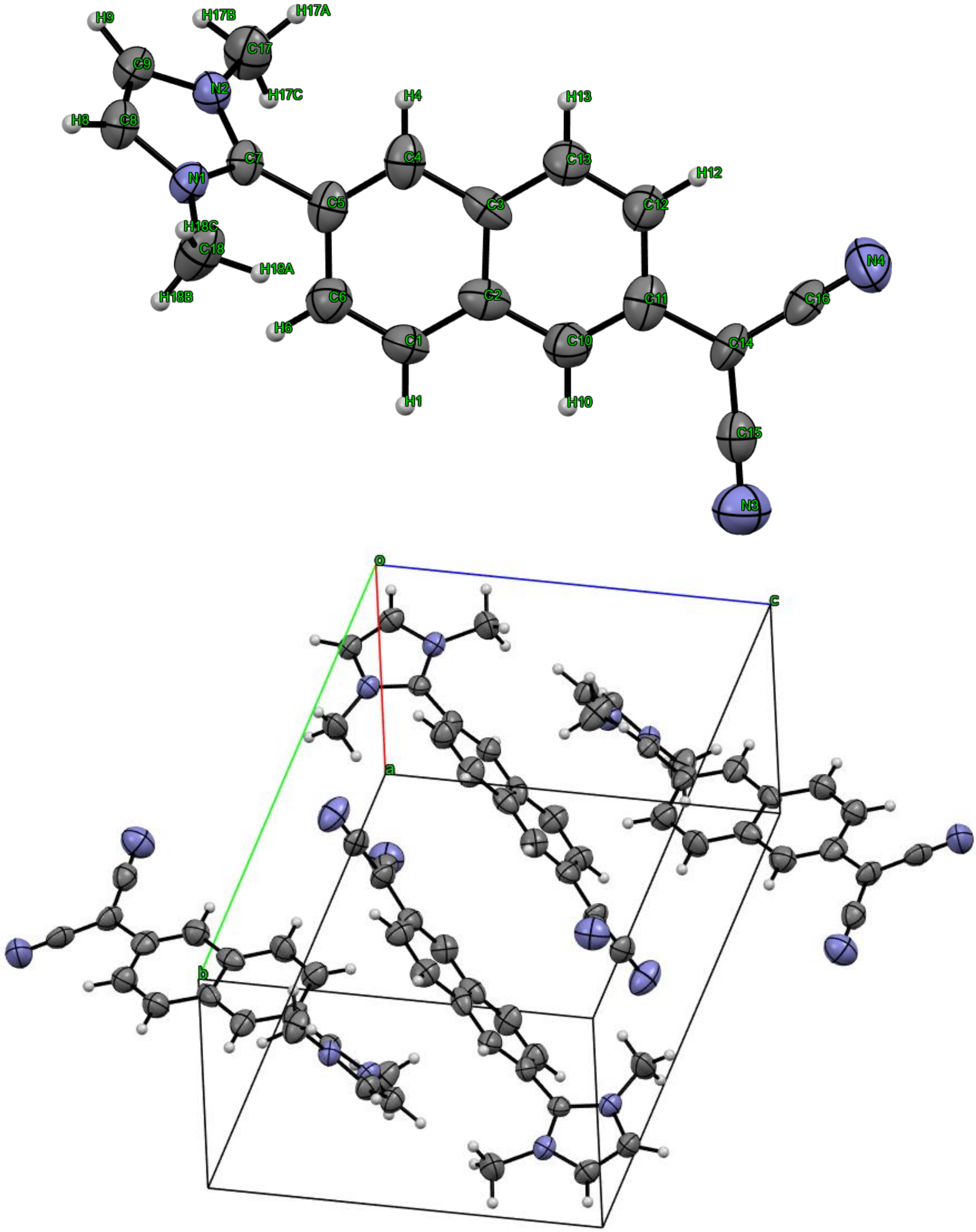

Figure S6. Ellipsoid representation of **pseudo-*p*-NB** with 66% thermal probability ellipsoids, and the packing in one unit cell along the principal crystallographic axes. CCDC Number: 2494031

Table S10. Crystal data and structural refinement for **pseudo-*p*-NB**

Bond precision: C-C = 0.0066 A Wavelength=1.54178

Cell: a=9.319(1) b=13.967(2) c=11.368(1)

alpha=90 beta=105.30(1) gamma=90

Temperature: 150 K

	Calculated	Reported
Volume	1427.2(3)	1427.2(3)
Space group	P 21/n	P 21/n
Hall group	-P 2yn	-P 2yn
Moiety formula	C18 H14 N4	?
Sum formula	C18 H14 N4	C18 H14 N4
Mr	286.33	286.33
Dx,g cm-3	1.333	1.333
Z	4	4
Mu (mm-1)	0.650	0.650
F000	600.0	600.0
F000’	601.65	
h,k,lmax	10,16,13	10,16,13
Nref	2435	2353
Tmin,Tmax	0.925,0.974	0.603,0.864
Tmin’	0.901	

Correction method= # Reported T Limits: Tmin=0.603 Tmax=0.864

AbsCorr = MULTI-SCAN

Data completeness= 0.966 Theta(max)= 65.082

R(reflections)= 0.0948(1738)

wR2(reflections)= 0.2616(2353)

S = 1.052 Npar= 202

Section S7 Aromaticity Analysis

Structures of ***m*-PB**, ***p*-PB**, **pseudo-*m*-NB**, and **pseudo-*p*-NB** were calculated using the coordinates of heavy atoms from crystal structures, allowing only the hydrogens to move, with the *optimizehydrogens true* keyword in ORCA.

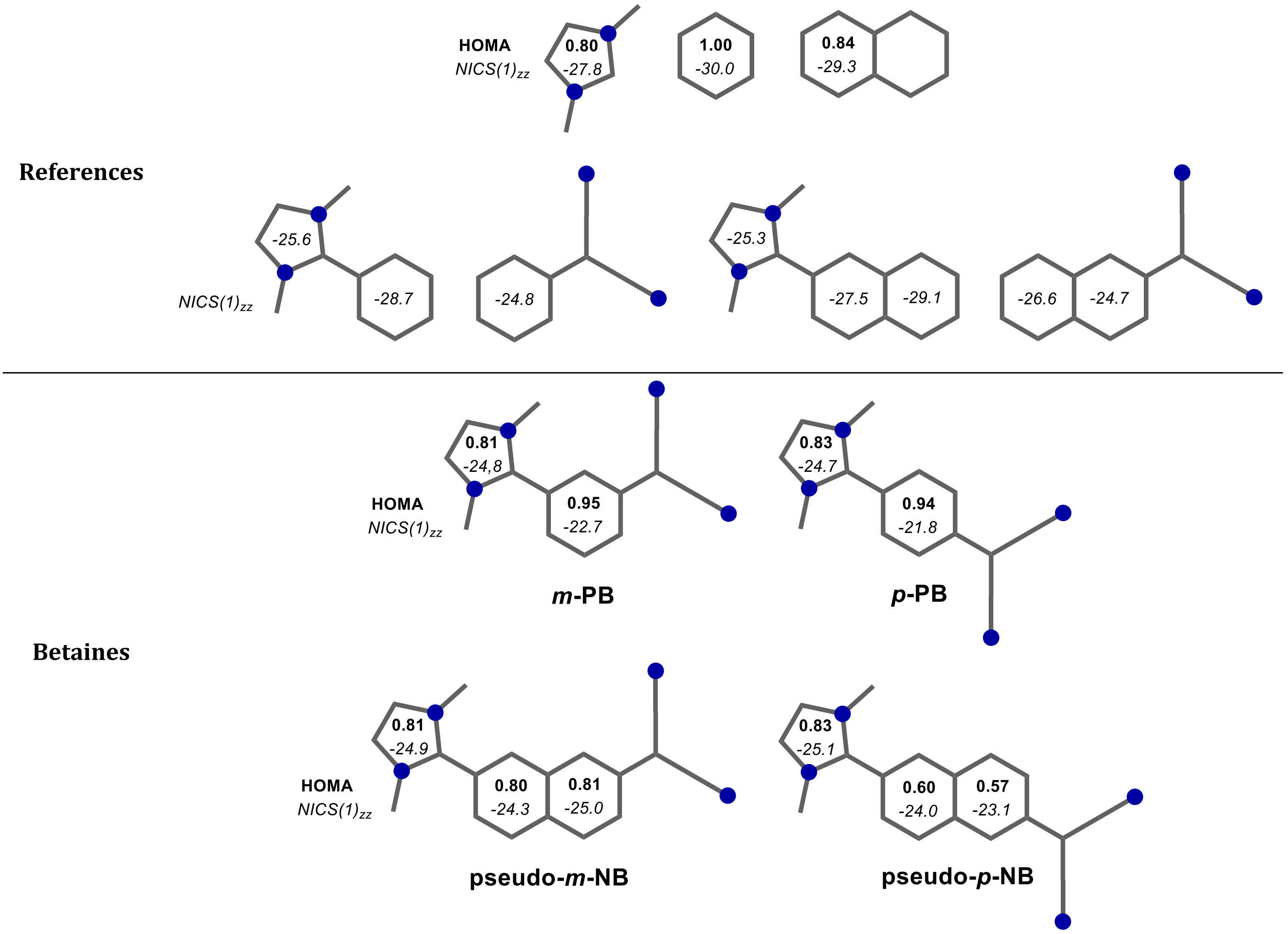

Figure S7. Ground state harmonic oscillator model of aromaticity **HOMA** and average nucleus-independent chemical shift 1 Ångström above & below the ring plane *NICS(1)*$_{zz}$ of reference compounds and compounds of study. Bond lengths for naphthalene[62] and dimethylimidazolium iodide[63] come from published crystal structures.

To determine changes in the indices of aromaticity upon excitation into the first singlet excited state (S_1), structures in both ground state and first excited singlet were optimised *without* constraints on the positions of heavy atoms. The structures were then used as inputs to calculate the aromaticity indices with Multiwfn.

The new HOMAc was chosen over the HOMA because it was parametrised to describe Hückel anti-aromatic compounds, the HOMER was chosen because it was parametrised for antiaromatic compounds in excited states. For NICS(1)$_{zz}$ indices, neither ORCA nor Gaussian are capable of performing NMR calculations in a time-dependent manner (*i.e.* for excited states). Thus, the NMR calculations were performed on open-shell triplets using the S_1 optimised geometries. This method was taken from literature.[64]

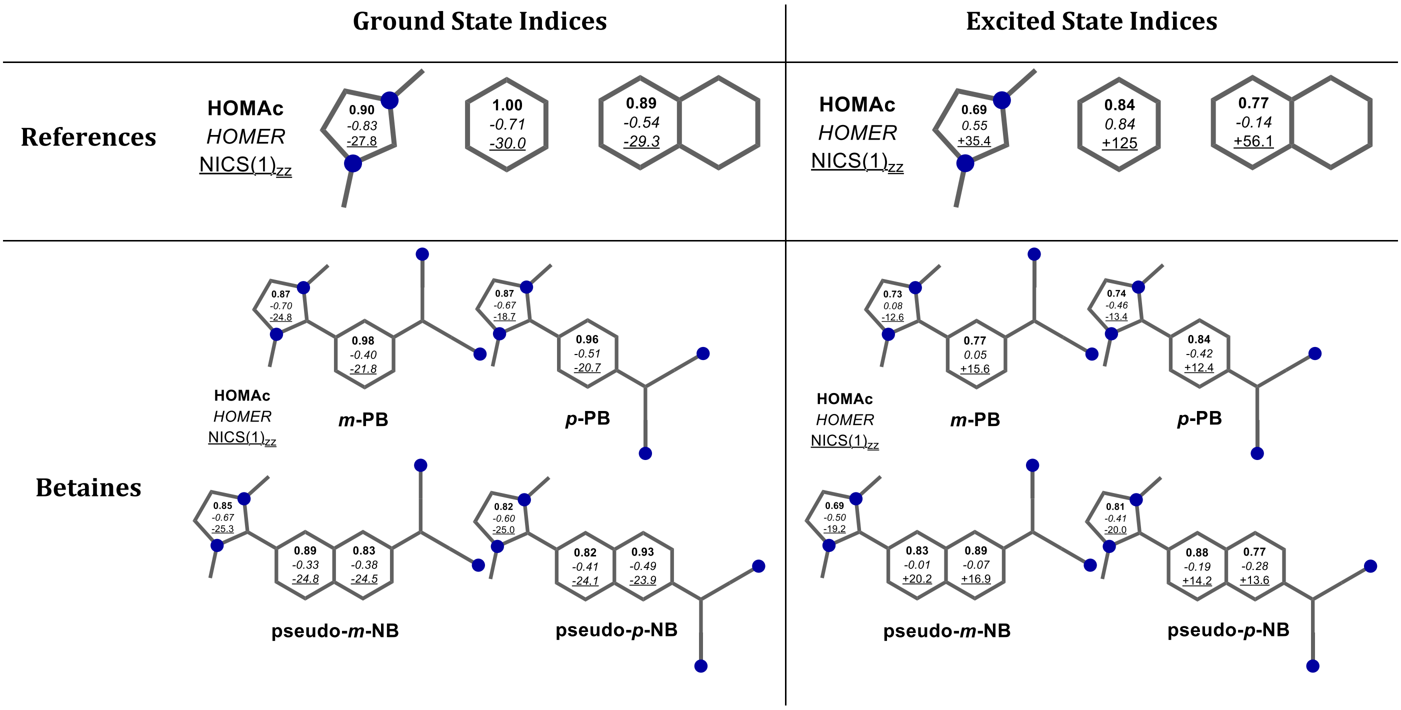

Figure S8. Ground and excited state HOMAc, HOMER, and NICS(1)$_{zz}$ indices of reference compounds and compounds of study.

Section S8 Infra-red Spectroscopy

Table S11. Signature dicyanomalonide stretching frequencies of model compounds and compounds of study.

Compound	Symmetric stretching frequency (cm^{-1})	Anti-symmetric stretching frequency (cm^{-1})
sodium phenylmalonide	2173	2110
sodium naphthylmalonide	2173	2116
***p*-PB**	2172	2131
***m*-PB**	2168	2127
**pseudo-*m*-NB**	2169	2128
**pseudo-*p*-NB**	2164	2123

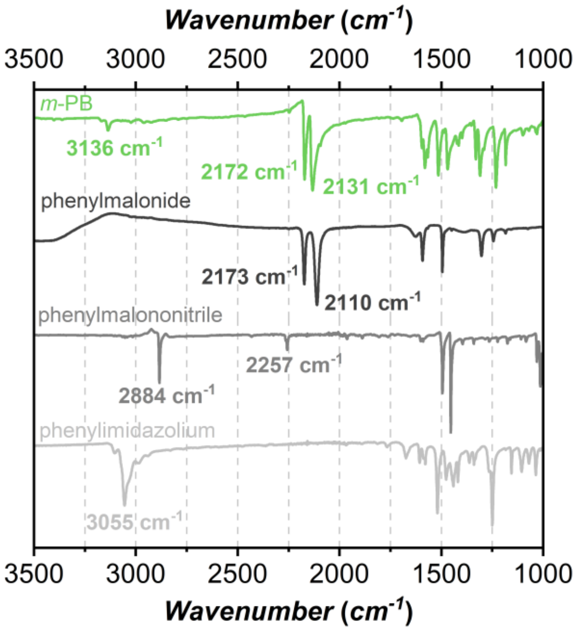

Figure S9. Infra-red spectra of ***m*-PB**, sodium phenylmalonide (in water), phenylmalononitrile **5**, and phenylimidazolium iodide **3**.

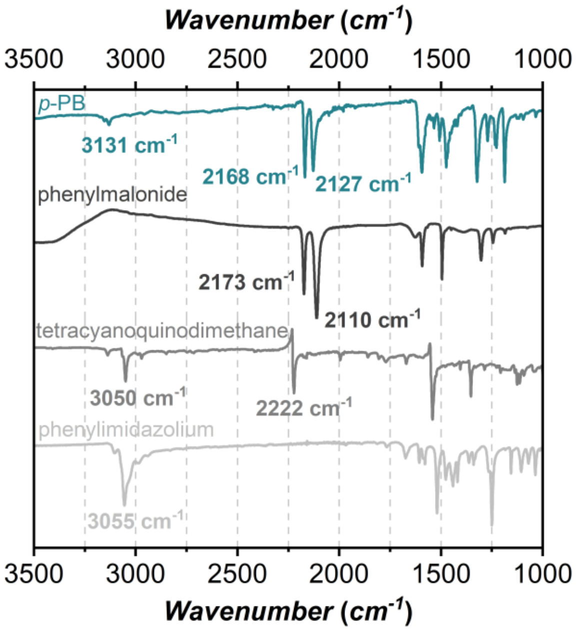

Figure S10. Infra-red spectra of ***p*-PB**, sodium phenylmalonide (in water), tetracyanoquinodimethane, and phenylimidazolium iodide **3**.

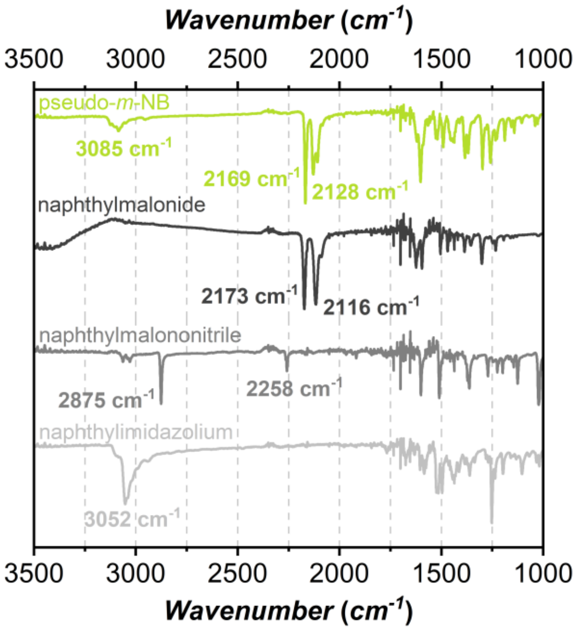

Figure S11. Infra-red spectra of **pseudo-*m*-NB**, sodium naphthylmalonide (in water), naphthylmalononitrile **19**, and naphthylimidazolium iodide **17**.

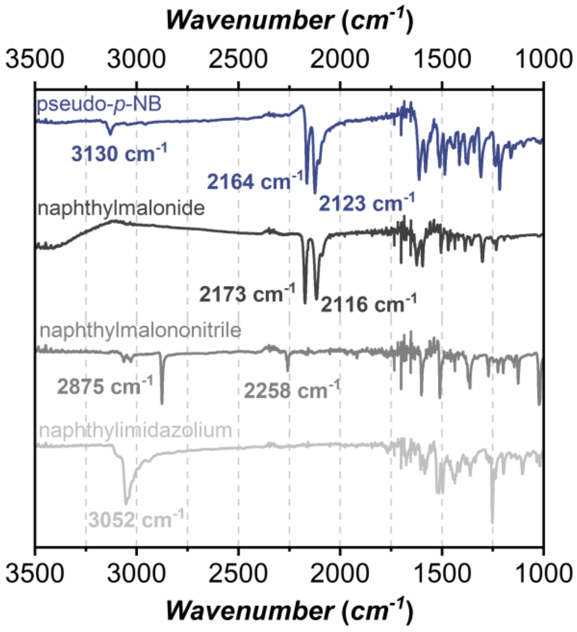

Figure S12. Infra-red spectra of **pseudo-*p*-NB**, sodium naphthylmalonide (in water), naphthylmalononitrile **19**, and naphthylimidazolium iodide **17**.

Section S9 2-D Nuclear Magnetic Resonance Spectroscopy

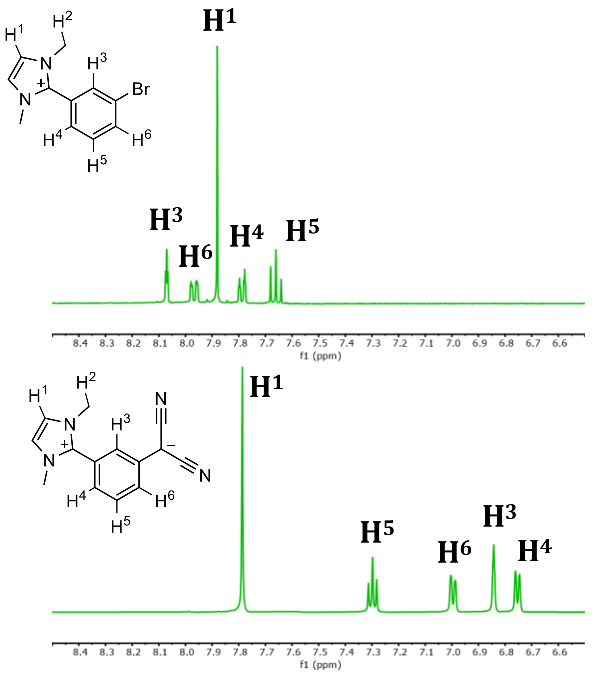

Figure S13. ^{1}H NMR spectrum (500 MHz, DMSO-d_6) of imidazolium iodide **9** and ***m*-PB**.

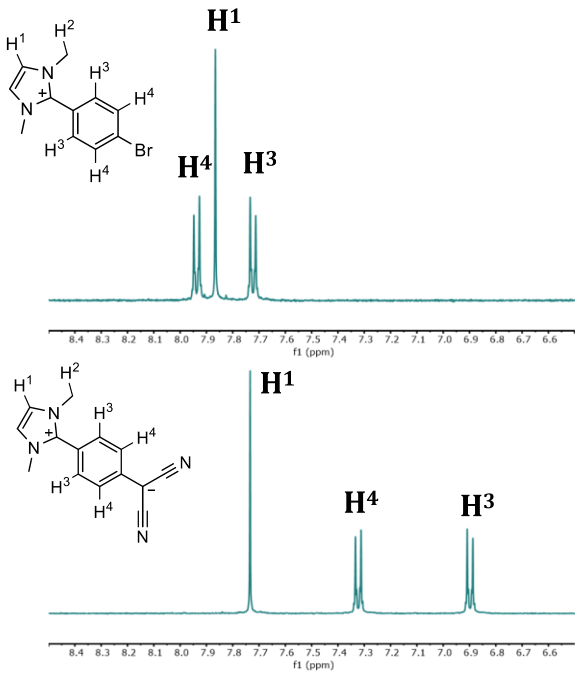

Figure S14. ^{1}H NMR spectrum (500 MHz, DMSO-d_6) of imidazolium iodide **13** and ***p*-PB**.

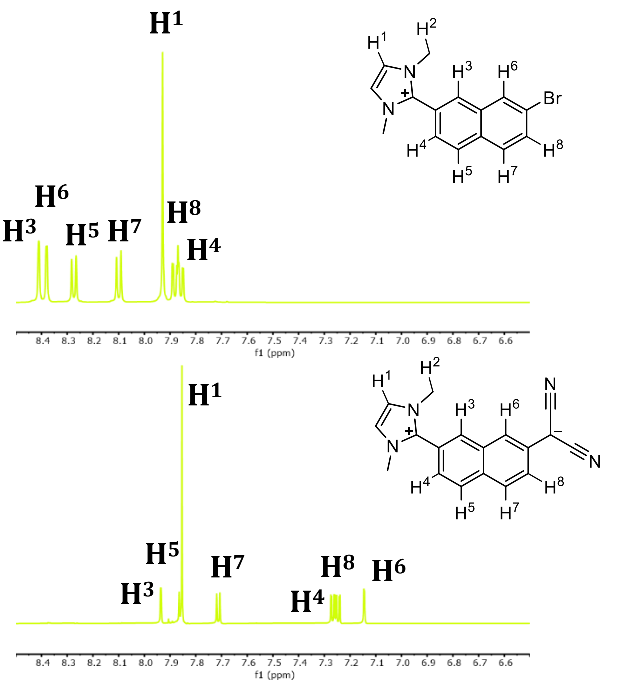

Figure S15. ^{1}H NMR spectrum (500 MHz, DMSO-d_6) of imidazolium iodide **25** and **pseudo-*m*-NB**.

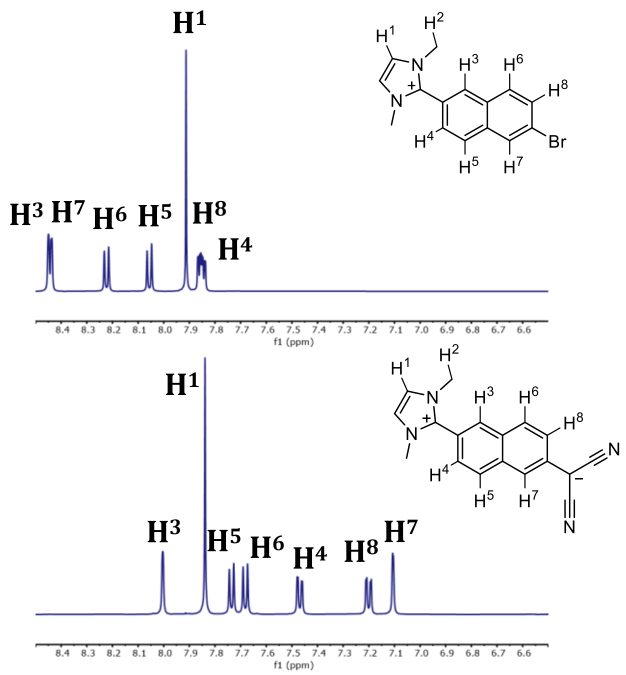

Figure S16. ^{1}H NMR spectrum (500 MHz, DMSO-d_6) of imidazolium cation **30** and **pseudo-*p*-PB**.

Table S12. ^{13}C Chemical shift changes between bromoimidazolium salts and analogous betaines.

**compound class**	Imidazolium N-C-N chemical shift (ppm)	Betaine N-C-N chemical shift (ppm)	**Change** in N-C-N chemical shift (ppm)	**Change** in (NC)-C-(CN) chemical shift (ppm)*
meta-phenylene	142.5	145.2	+2.7	+2.0
para-phenylene	143.7	145.5	+1.8	+4.4
meta-naphthylene	143.8	144.9	+1.1	+2.8
para-naphthylene	143.9	145.0	+1.1	+4.2

*change in chemical shift relative to sodium phenylmalonide central (NC)-C-(CN) at 27.1 ppm in DMSO-d_6.[65]

Table S13. ^{1}H Chemical shift changes between phenylene bromoimidazolium salts and betaines.

	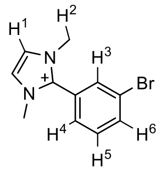	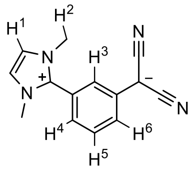		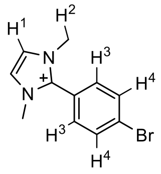	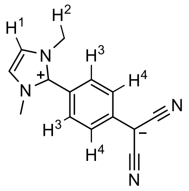	
**H**	Imidazolium (ppm)	Betaine (ppm)	**Change** (ppm)	Imidazolium (ppm)	Betaine (ppm)	**Change** (ppm)
1	7.88	7.79	−0.09	7.89	7.73	−0.14
2	3.69	3.66	−0.03	3.68	3.69	−0.01
3	8.07	6.85	−1.22	7.72	6.90	−0.82
4	7.79	6.76	−1.03	7.94	7.32	−0.62
5	7.66	7.31	−0.35	/	/	/
6	7.97	7.00	−0.97	/	/	/

Table S14. ^{1}H Chemical shift changes between naphthylene bromoimidazolium salts and betaines.

	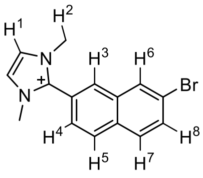	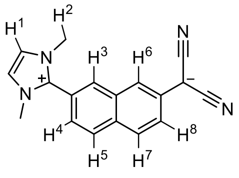		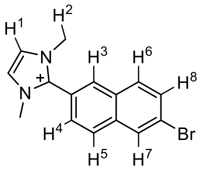	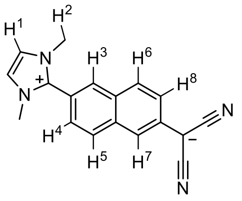	
**H**	Imidazolium (ppm)	Betaine (ppm)	**Change** (ppm)	Imidazolium (ppm)	Betaine (ppm)	**Change** (ppm)
1	7.93	7.85	−0.08	7.93	7.84	−0.09
2	3.77	3.76	−0.01	3.75	3.75	0.00
3	8.41	7.94	−0.47	8.47	8.00	−0.47
4	7.87	7.29	−0.58	7.85	7.48	−0.37
5	8.28	7.87	−0.41	8.08	7.74	−0.34
6	8.39	7.16	−1.23	8.23	7.68	−0.55
7	8.11	7.71	−0.40	8.45	7.11	−1.34
8	7.88	7.25	−0.63	7.87	7.22	−0.65

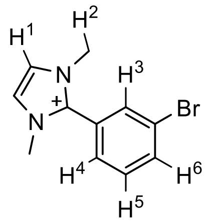

9 Using the known positions of H^2 at 3.68 ppm and H^1 at 7.87 ppm, these protons correlate through HMBC to the imidazolium N-C-N at 142.5 ppm which had no attached protons. Triplets H^3 and H^5 were assigned by coupling constants. The H^4 at 7.79 ppm correlates through HMBC to the N-C-N at 142.5 ppm, while remaining signal for H^6 at 7.97 ppm does not. Therefore, H^4 is closer to the imidazolium group.

HMQC and expansion for compound **9** in DMSO-d_6 (peak shifting in x- and y-directions is an instrument artifact).

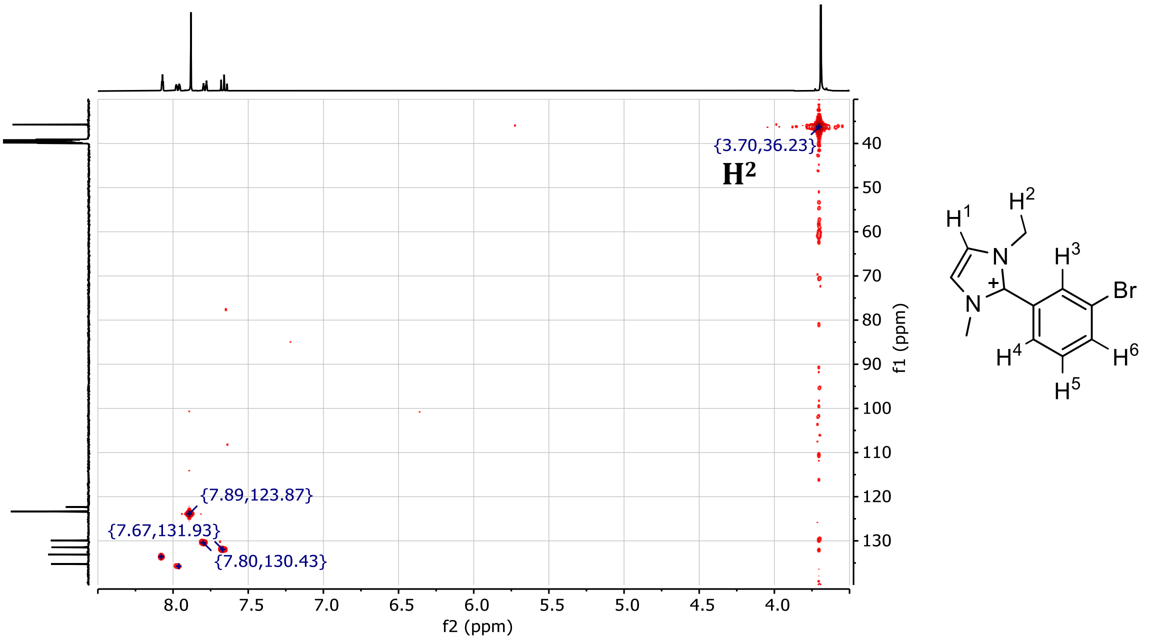

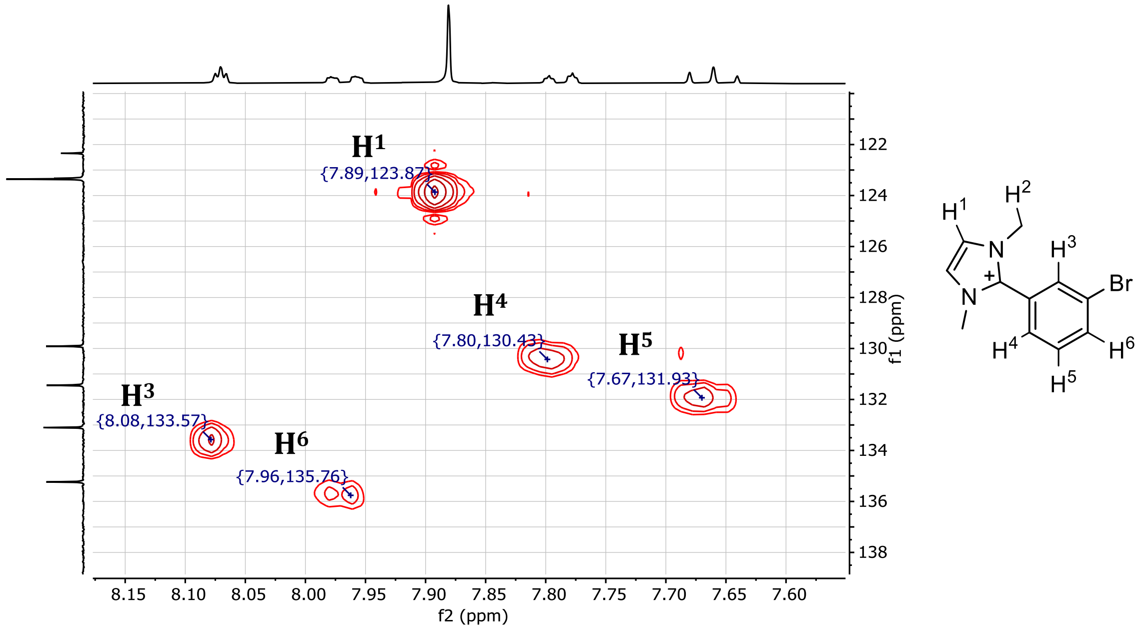

HMBC for compound **9** in DMSO-d_6 (peak shifting in x- and y-directions is an instrument artifact).

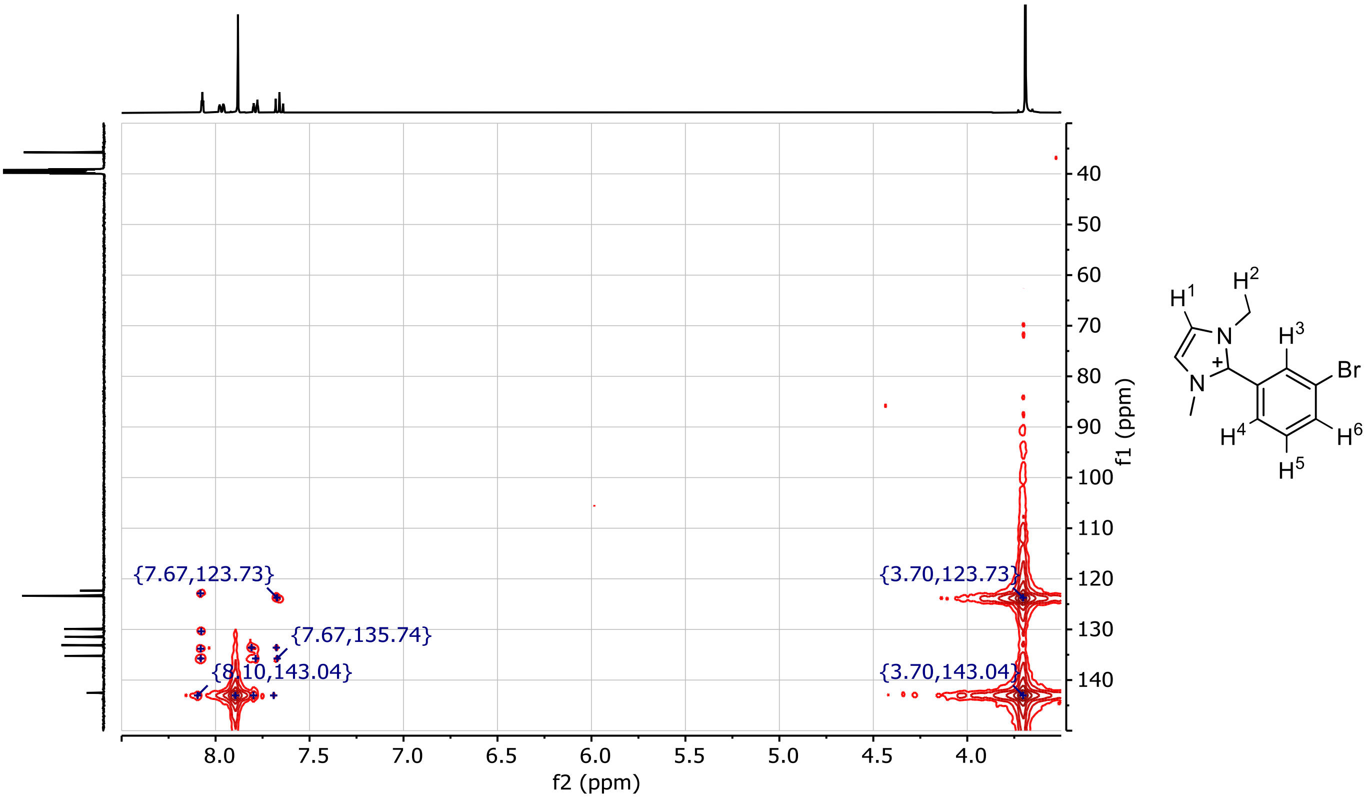

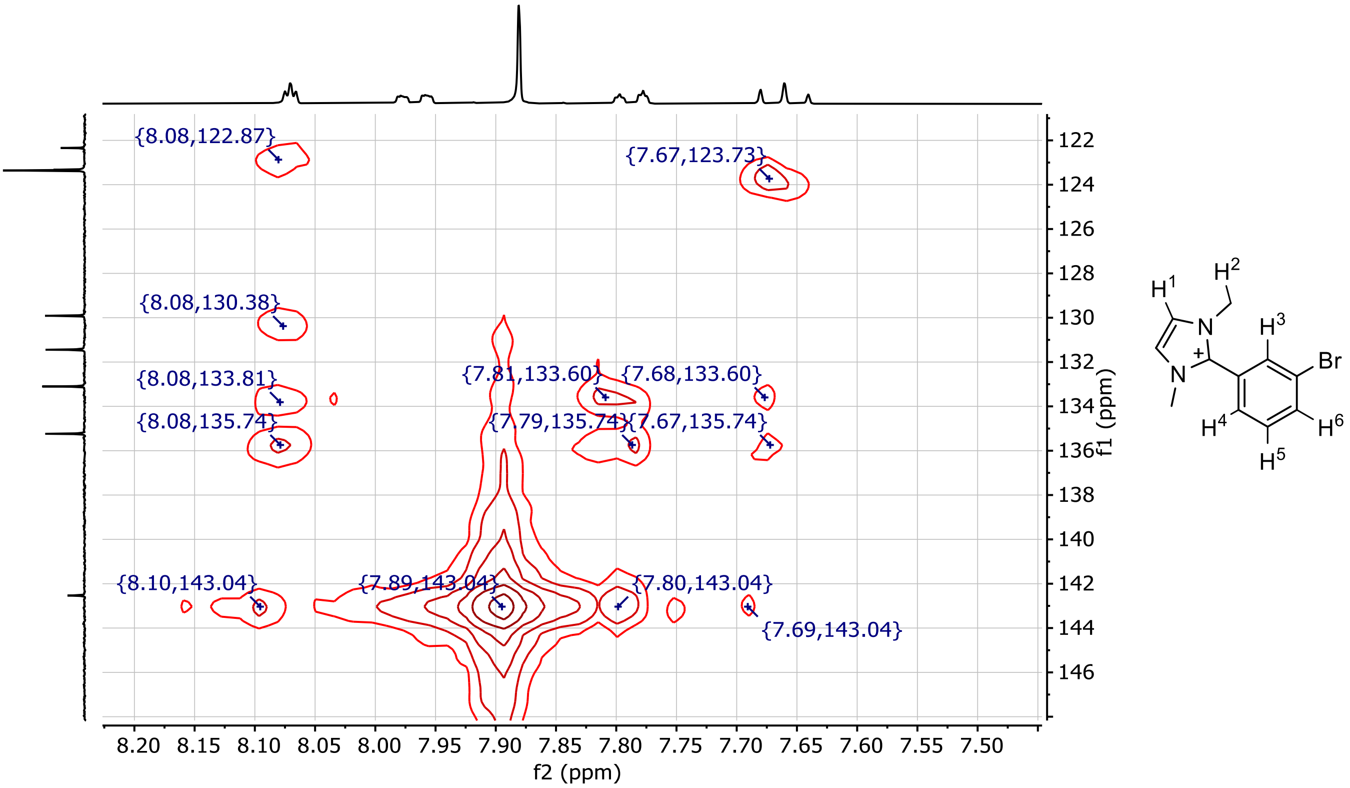

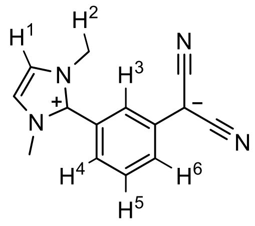

***m*-PB** Using the known positions of H^2 at 3.65 ppm and H^1 at 7.79 ppm, these protons correlate through HMBC to the imidazolium N-C-N at 145.2 ppm which had no attached protons. One dd H^4 at 6.75 ppm correlates through HMBC to the N-C-N carbon, while the other dd H^6 at 6.99 ppm does not correlate. The dd H^6 correlates through HMBC to the C-$(CN)_2$ carbon at 29.1 ppm, while H^4 does not. Therefore, H^4 is closer to the imidazolium group than H^6.

HMQC and expansion for compound ***m*-PB** in DMSO-d_6 (peak shifting in x- and y-directions is an instrument artifact).

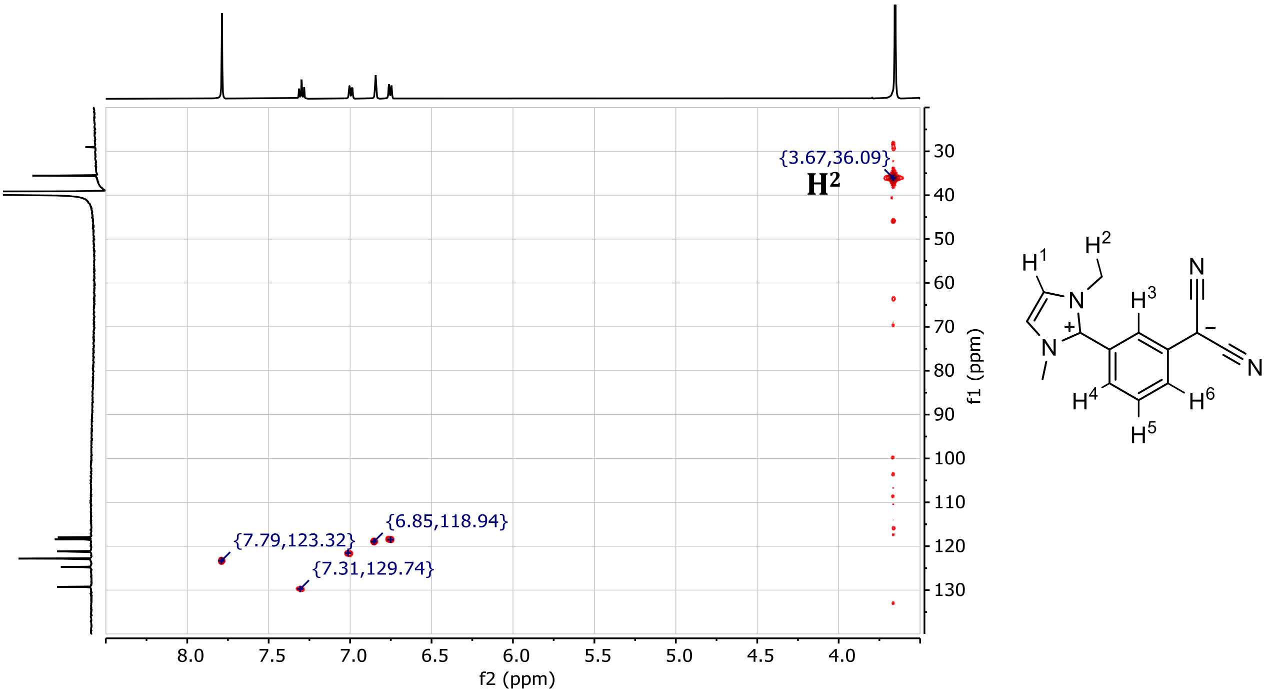

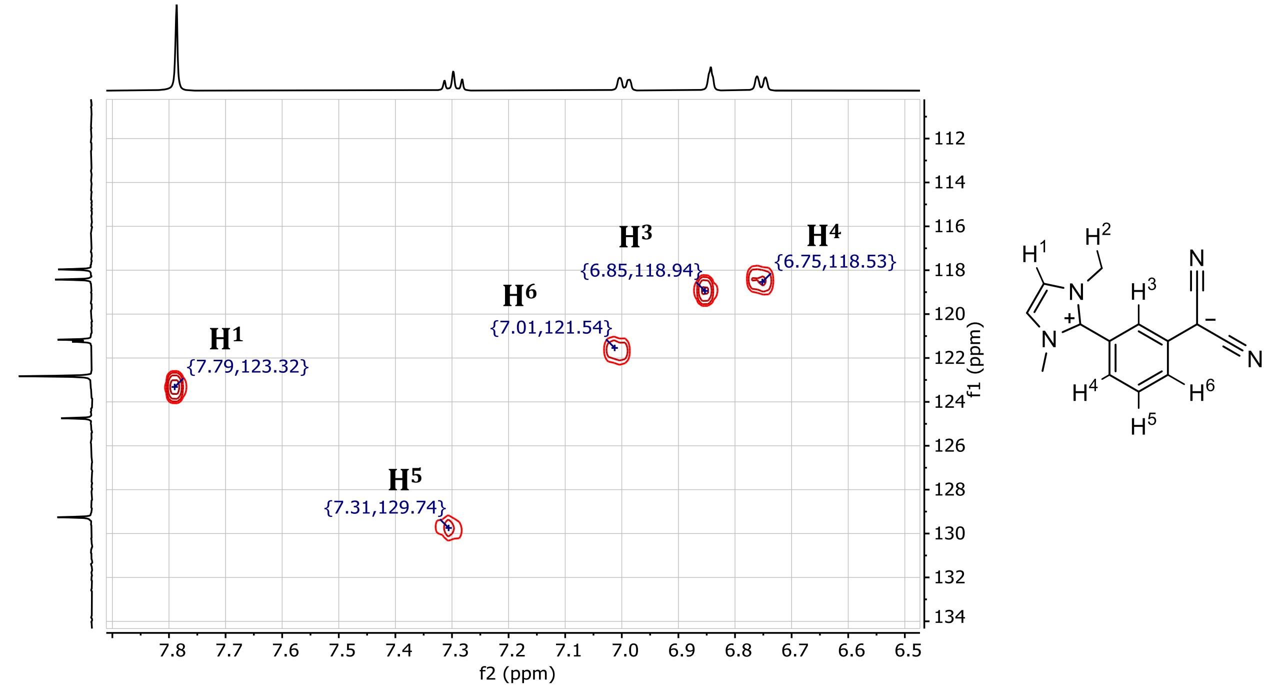

HMBC and expansion for compound ***m*****-PB** in DMSO-d_6 (peak shifting in x- and y-directions is an instrument artifact).

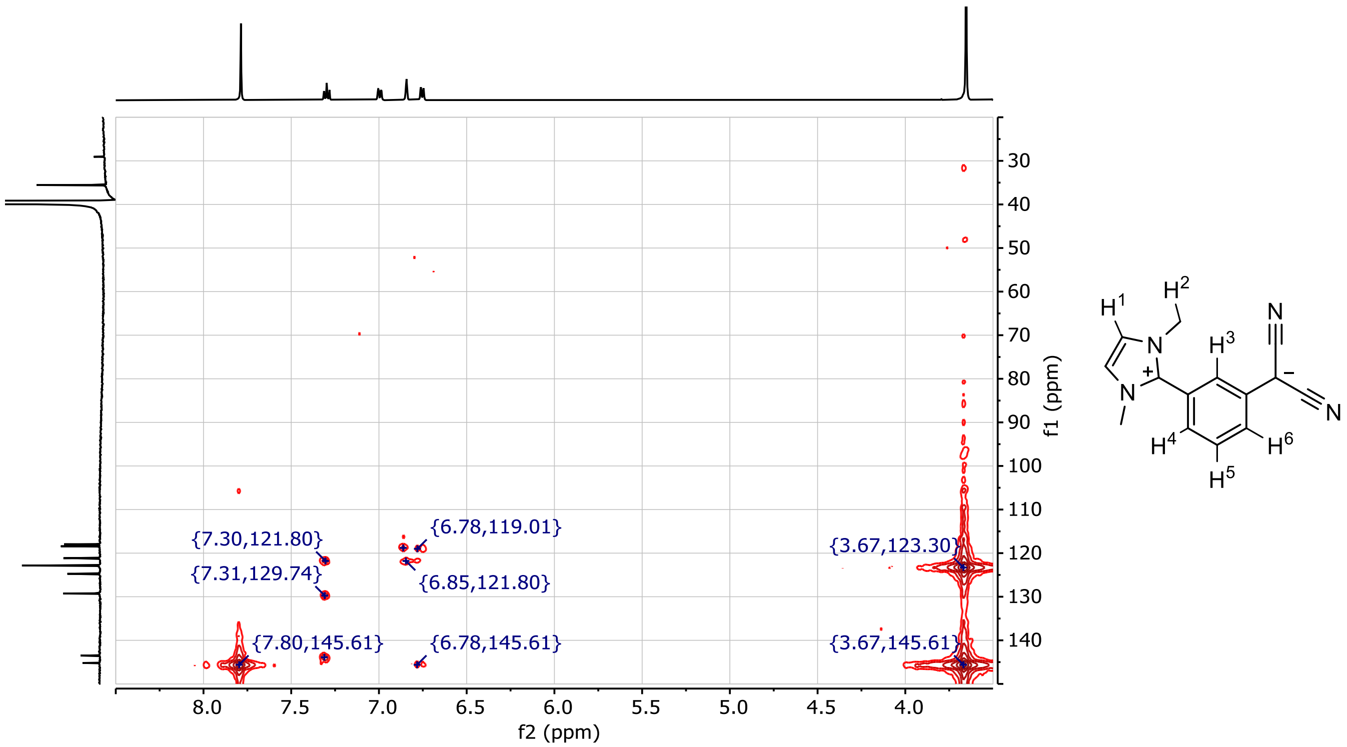

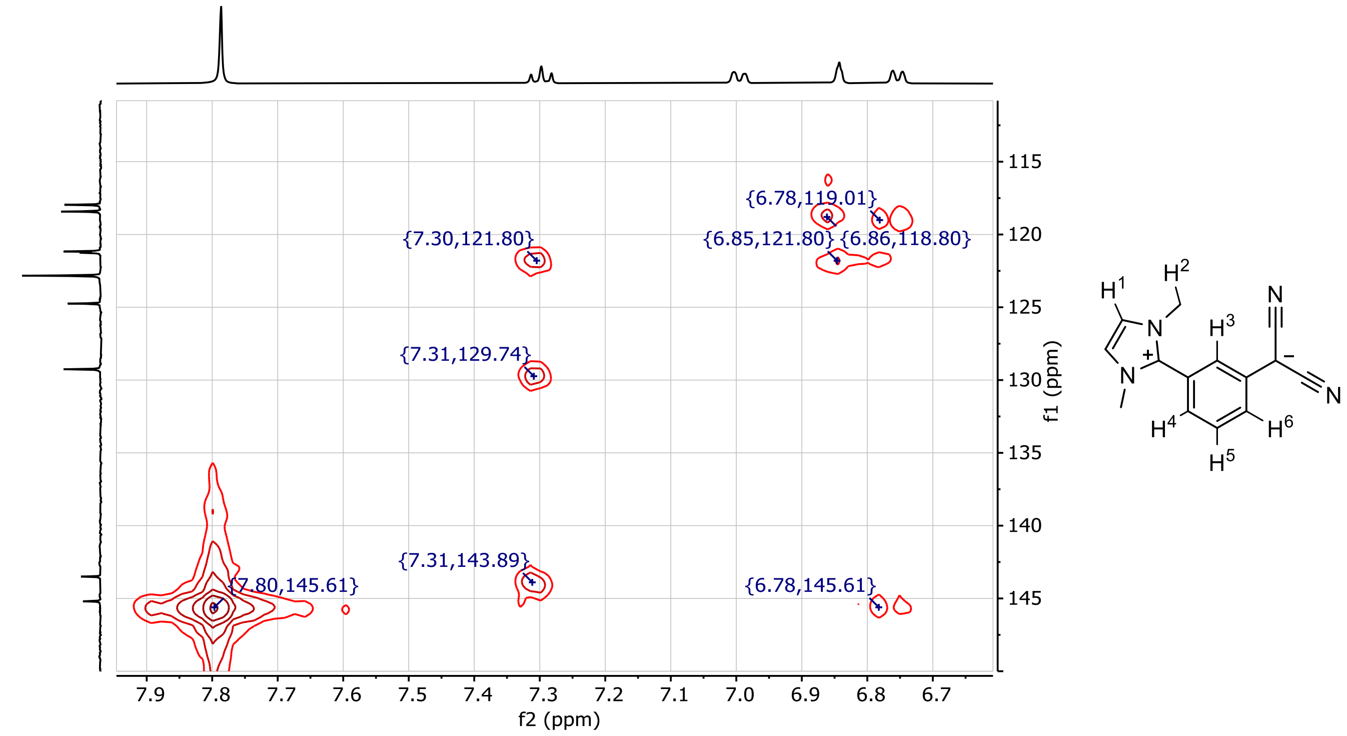

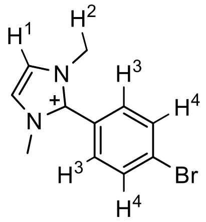

13 Using the known positions of H^2 at 3.69 ppm and H^1 at 7.88 ppm, these protons correlate through HMBC to the imidazolium N-C-N at 143.7 ppm which had no attached protons. The doublet H^3 at 7.72 ppm correlates through HMBC to that N-C-N carbon while the other doublet H^4 at 7.94 ppm did not. Therefore H^3 is closer to the imidazolium group than H^4.

HMQC and expansion for compound **13** in DMSO-d_6 (peak shifting in x- and y-directions is an instrument artifact).

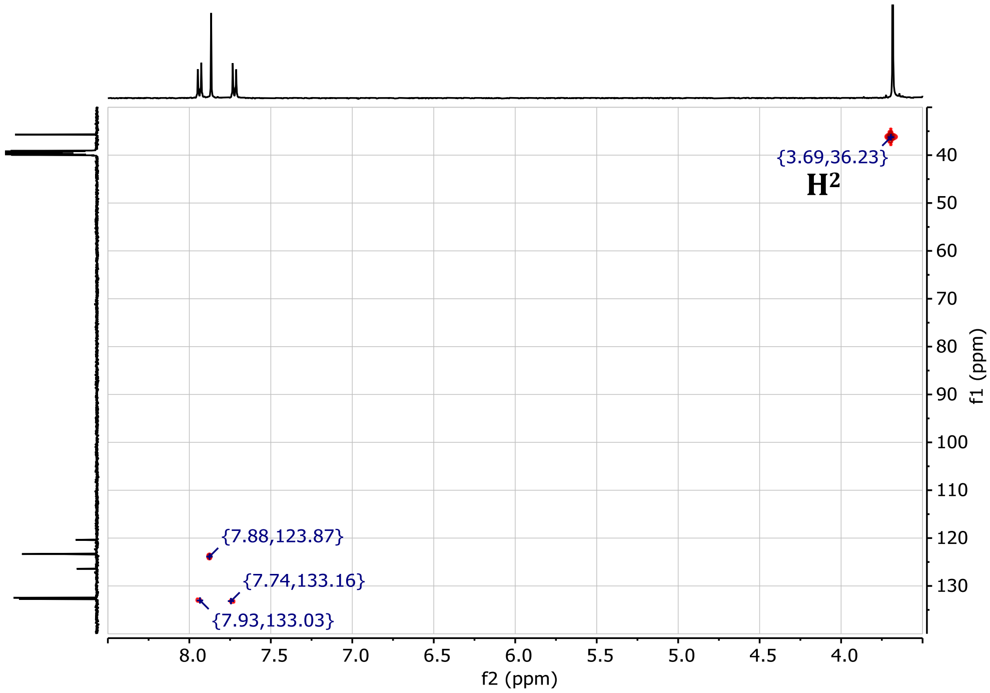

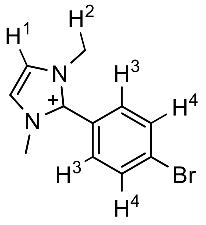

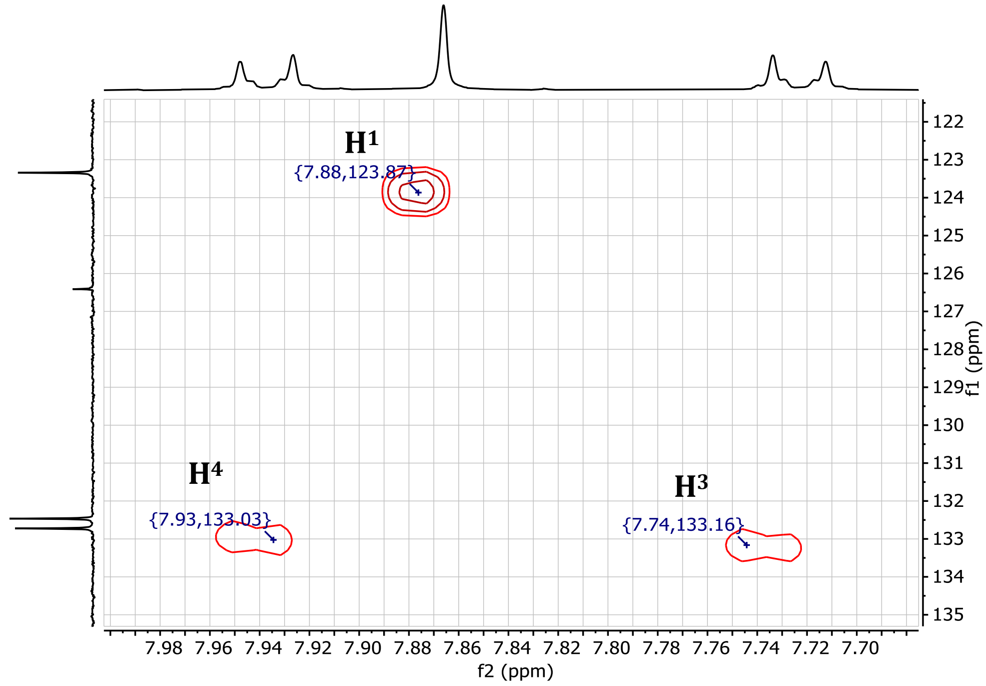

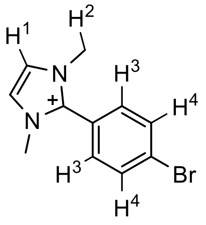

HMQC and expansion for compound **13** in DMSO-d_6 (peak shifting in x- and y-directions is an instrument artifact).

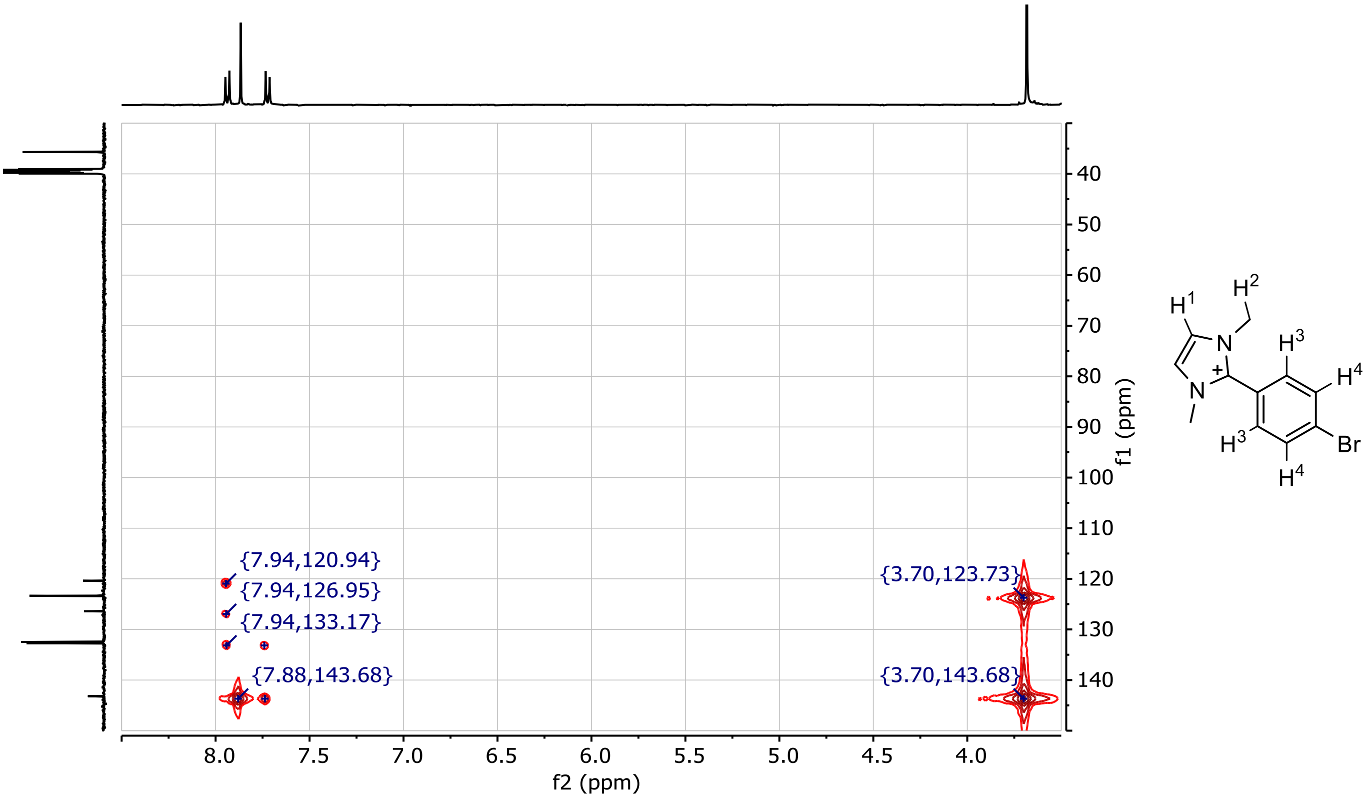

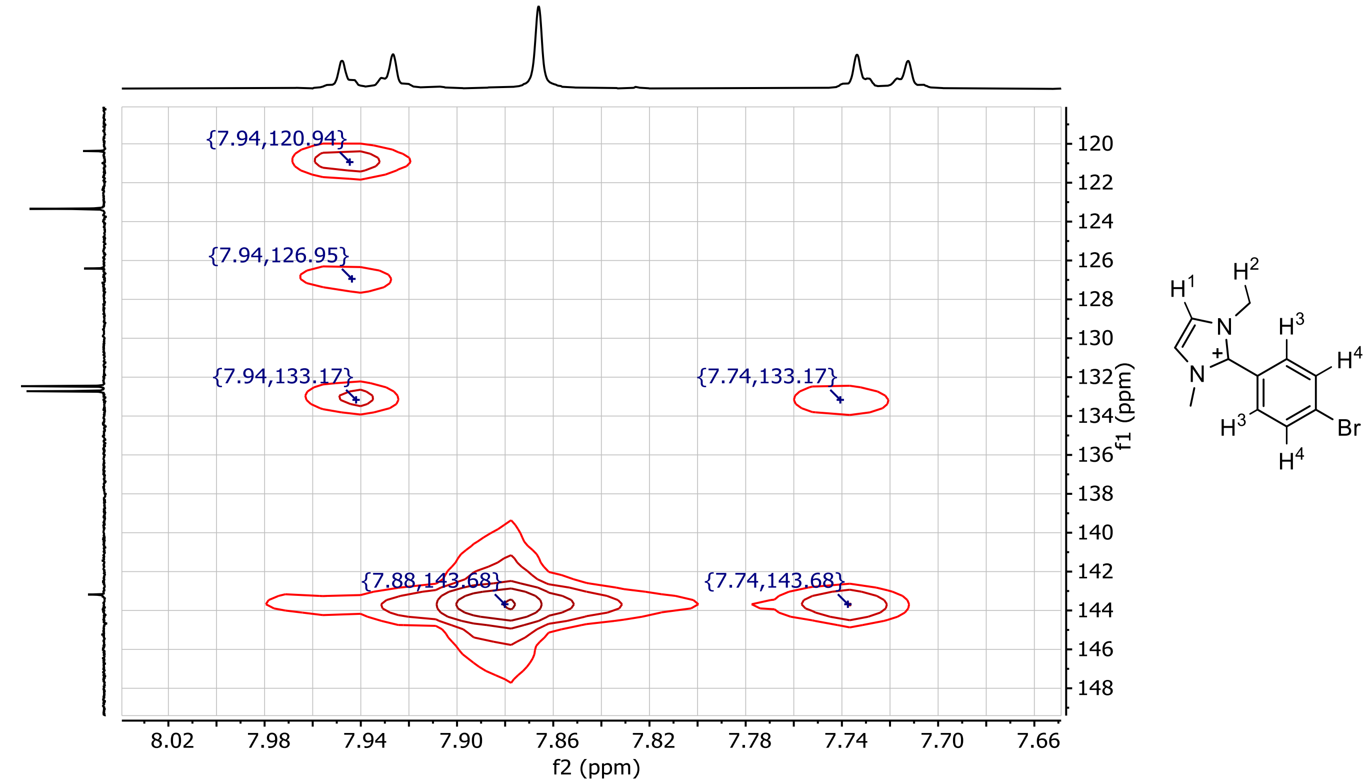

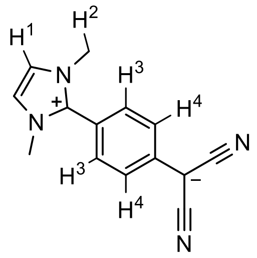

***p*-PB** Using the known positions of H^2 at 3.69 ppm and H^1 at 7.73 ppm, these protons correlate through HMBC to the imidazolium N-C-N at 145.5 ppm which had no attached protons. Assignment of H^3 and H^4 relies upon correlation to the *ipso* carbons which bear the imidazolium and the malonide groups, because the expected correlation to the N-C-N carbon was not observed. Using the change in ^{13}C chemical shift upon deprotonation (131 to 149 ppm)[65] of phenylmalononitrile in DMSO-d_6 and the assignment from previous literature,[66] the *ipso* C attached to imidazolium is 107.3 ppm, and the *ipso* C attached to malonide is 146.7 ppm. Then doublet H^3 at 6.90 ppm correlates through HMBC to the *ipso* C imidazolium at 107.3 ppm, while H^4 at 7.32 ppm does not. Also, H^4 correlates through HMBC to the C-$(CN)_2$ carbon at 31.5 ppm, while H^3 does not.

HMQC and expansion for compound ***p*-PB** in DMSO-d_6 (peak shifting in x- and y-directions is an instrument artifact).

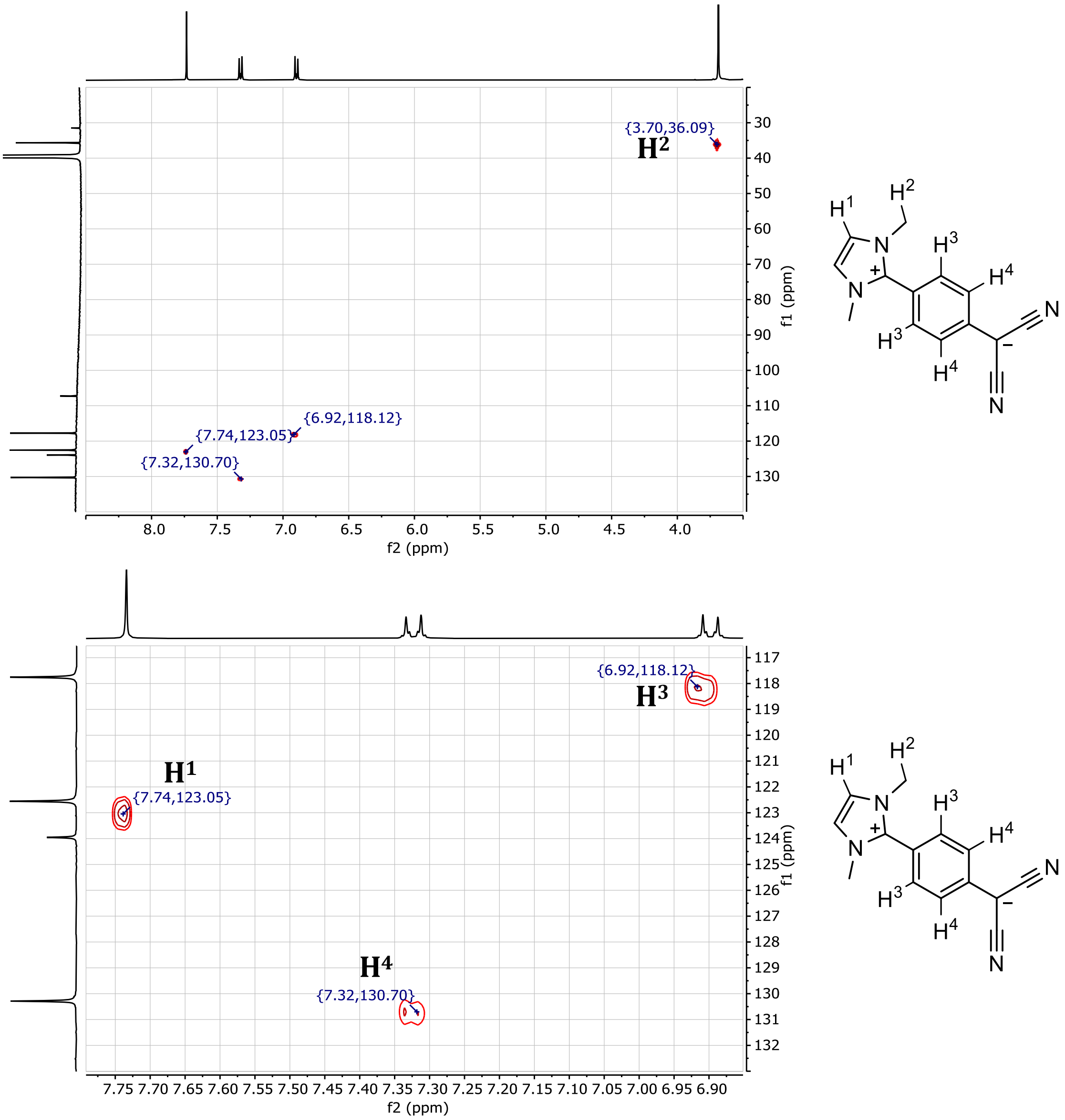

HMQC and expansion for compound ***p*-PB** in DMSO-d_6 (peak shifting in x- and y-directions is an instrument artifact).

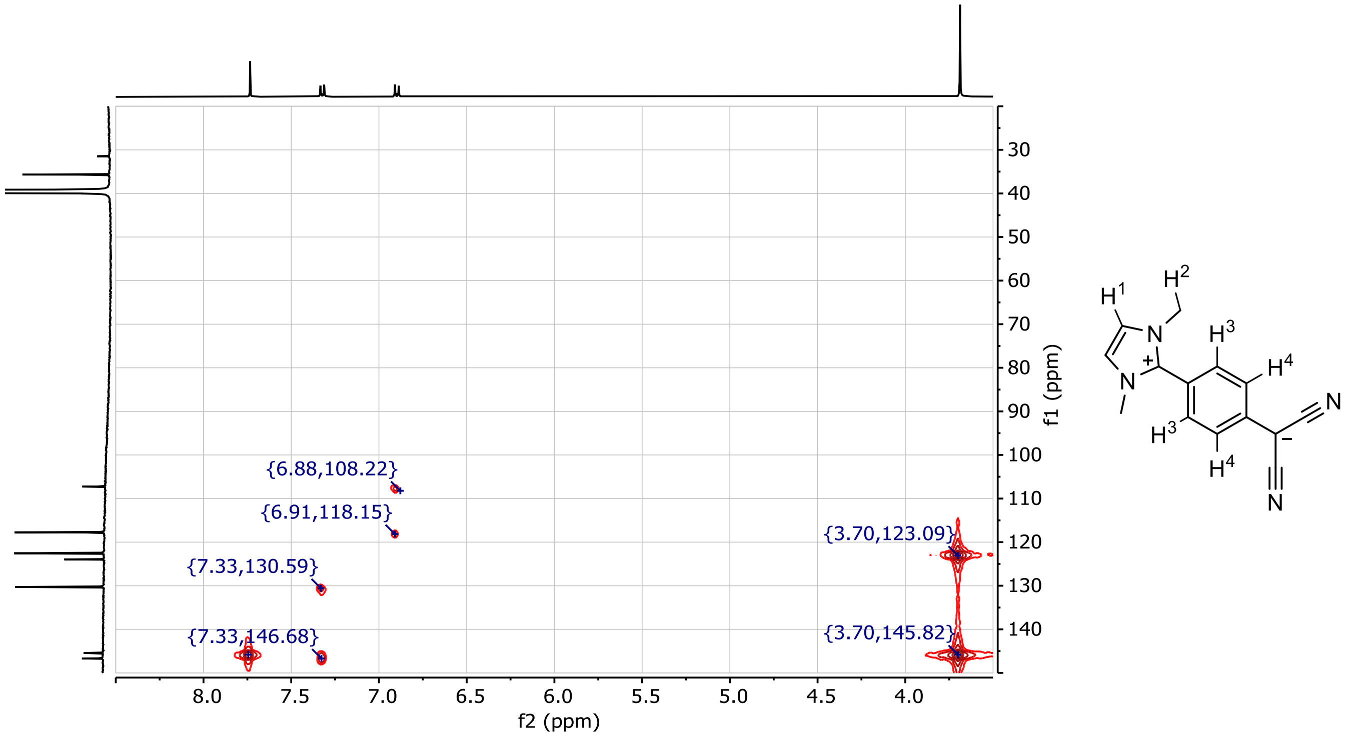

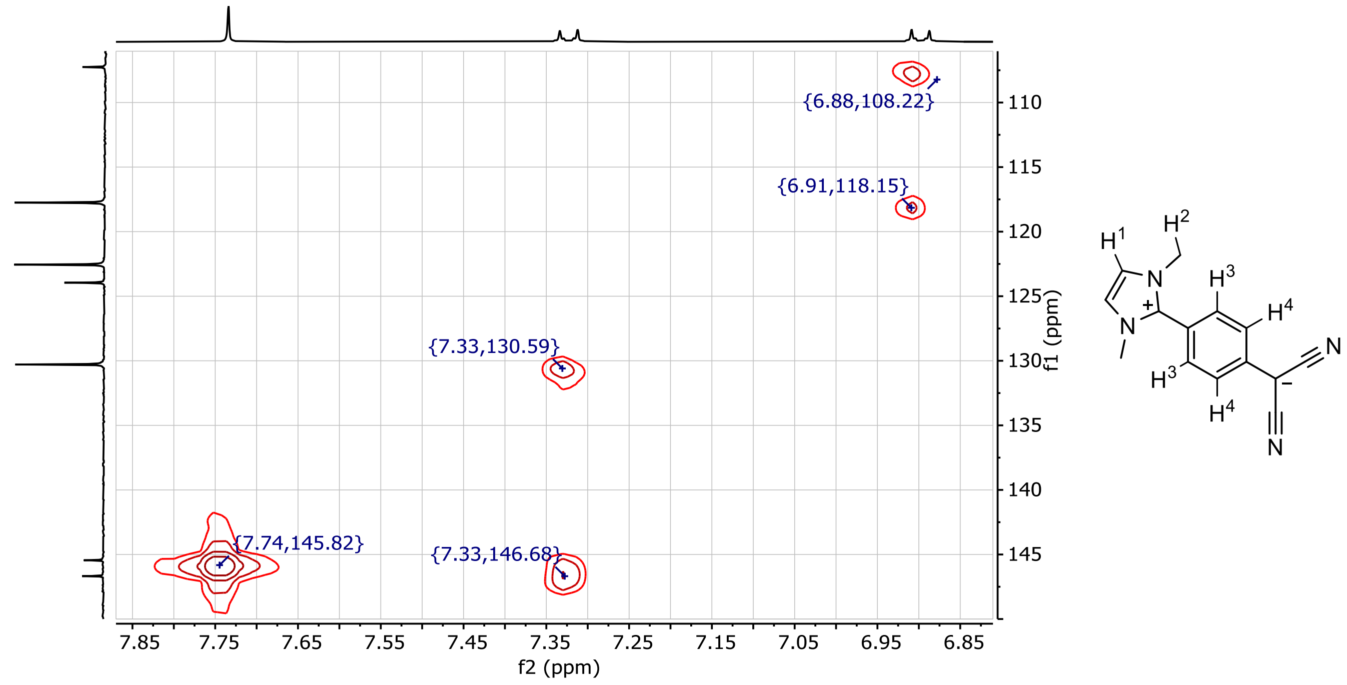

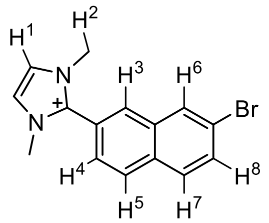

25 Using the known positions of H^2 at 3.76 ppm and H^1 at 7.93 ppm, these protons correlate through HMBC to the imidazolium N-C-N at 143.8 ppm which had no attached protons. Coupling constants group H^3, H^4, and H^5 separately from H^6, H^7 and H^8. Once grouped, an HMBC correlation between the N-C-N carbon and H^3 at 8.41 ppm and H^4 at 7.85 ppm assigns that group of protons to the ring with the imidazolium substituent.

HMQC and expansion for compound **25** in DMSO-d_6 (peak shifting in x- and y-directions is an instrument artifact).

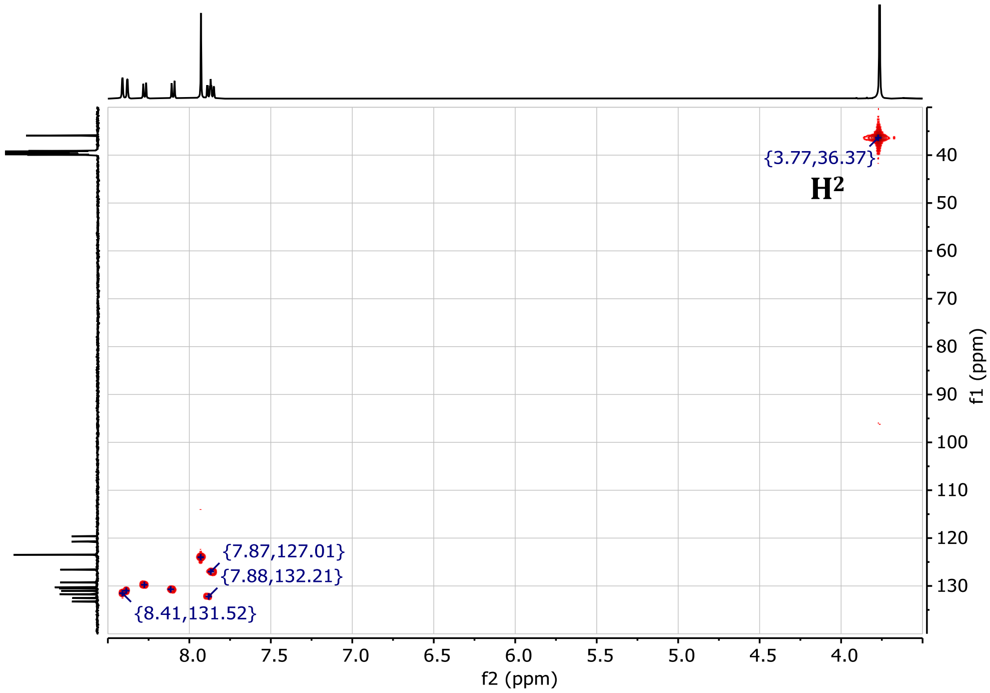

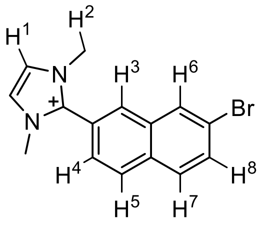

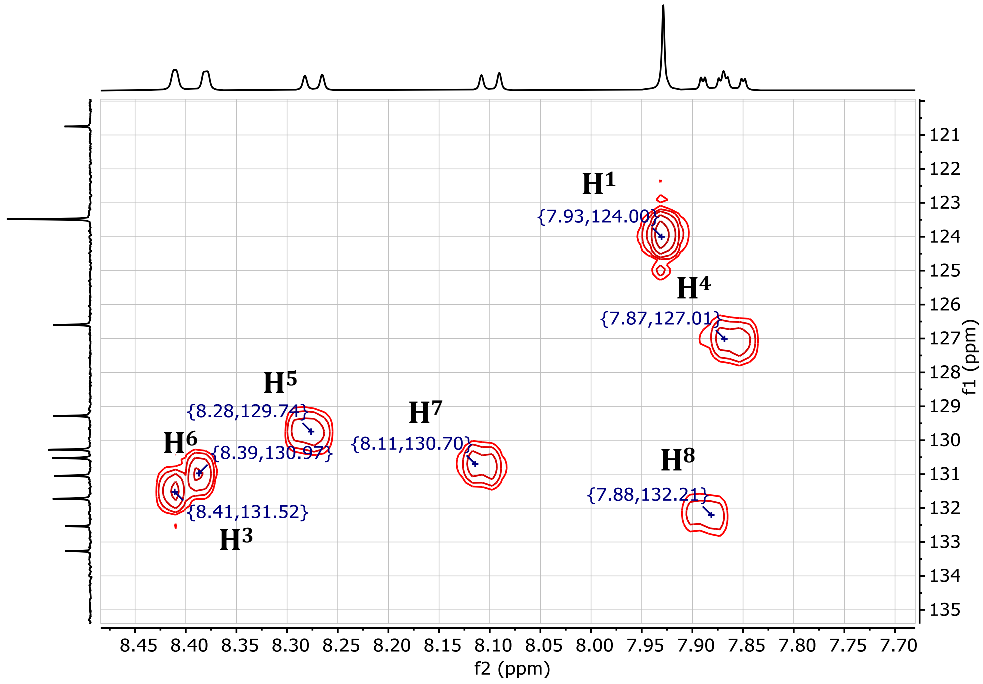

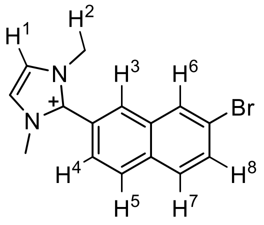

HMBC and expansion for compound **25** in DMSO-d_6 (peak shifting in x- and y-directions is an instrument artifact).

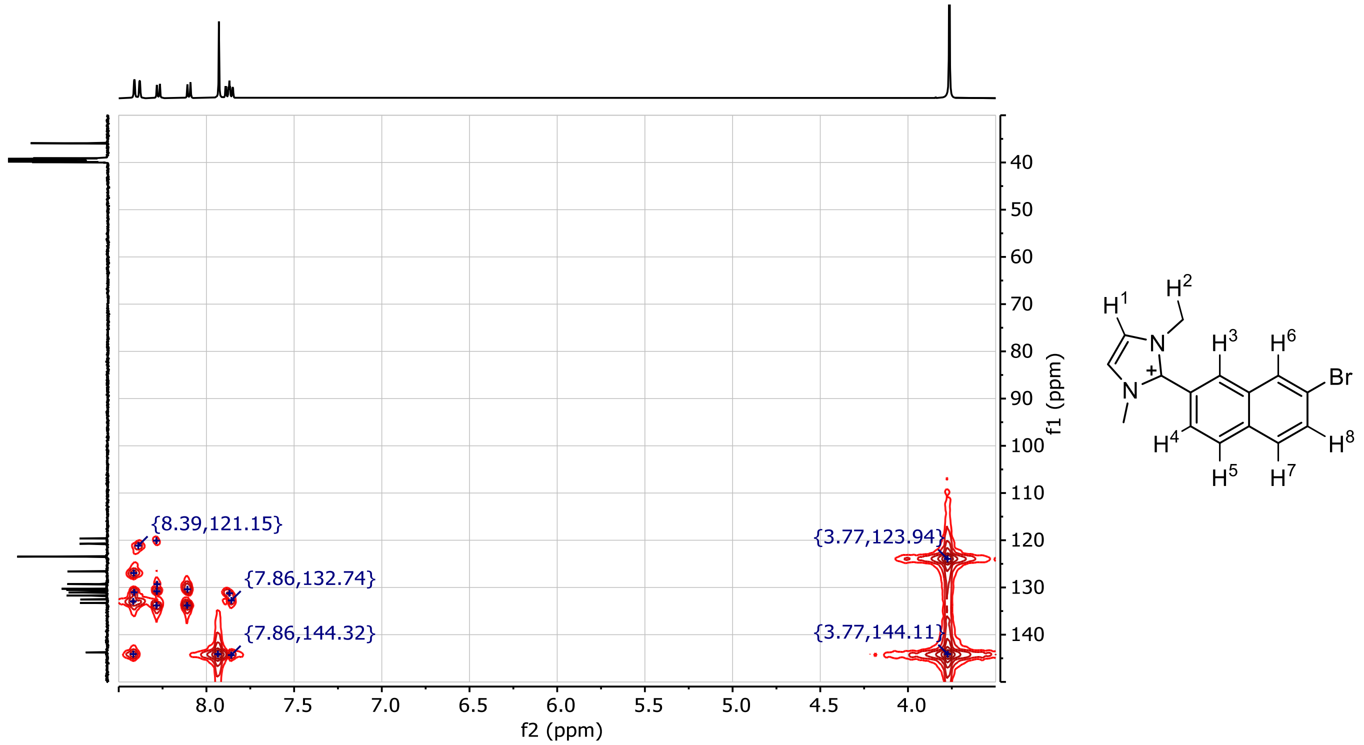

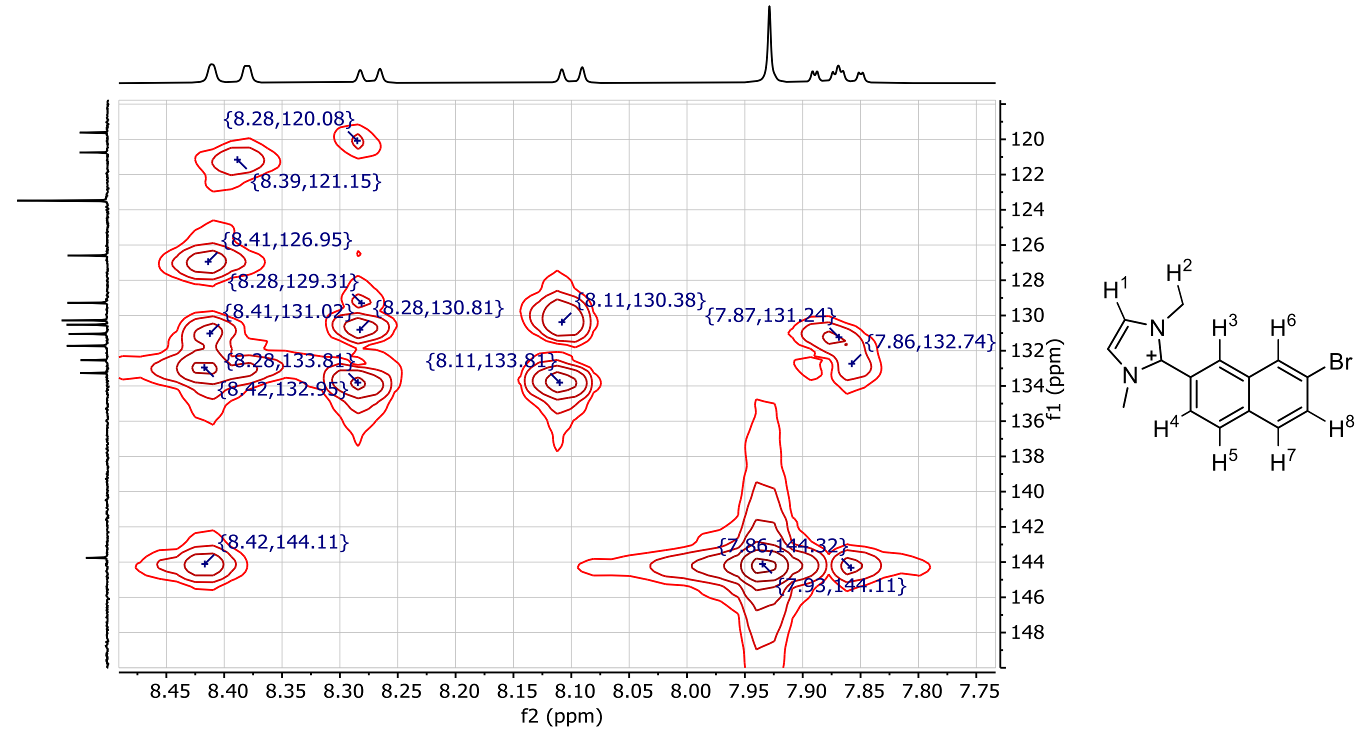

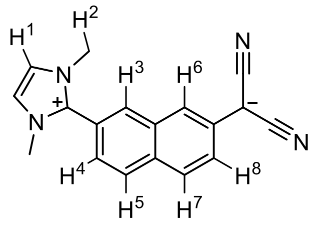

pseudo-*m*-NB Using the known positions of H[2] at 3.75 ppm and H[1] at 7.85 ppm, these protons correlate through HMBC to the imidazolium N-C-N at 144.9 ppm which had no attached protons. Coupling constants group H[3], H[4], and H[5] separately from H[6], H[7] and H[8]. Once grouped, an HMBC correlation between the N-C-N carbon and the *meta*-coupled doublet H[3] at 7.94 ppm assigned that group of protons to the to the ring with the imidazolium substituent. No correlations between protons and the malonide carbon are observed.

HMQC and expansion for **pseudo-*m*-NB** in DMSO-d_6 (peak shifting in x- and y-directions is an instrument artifact).

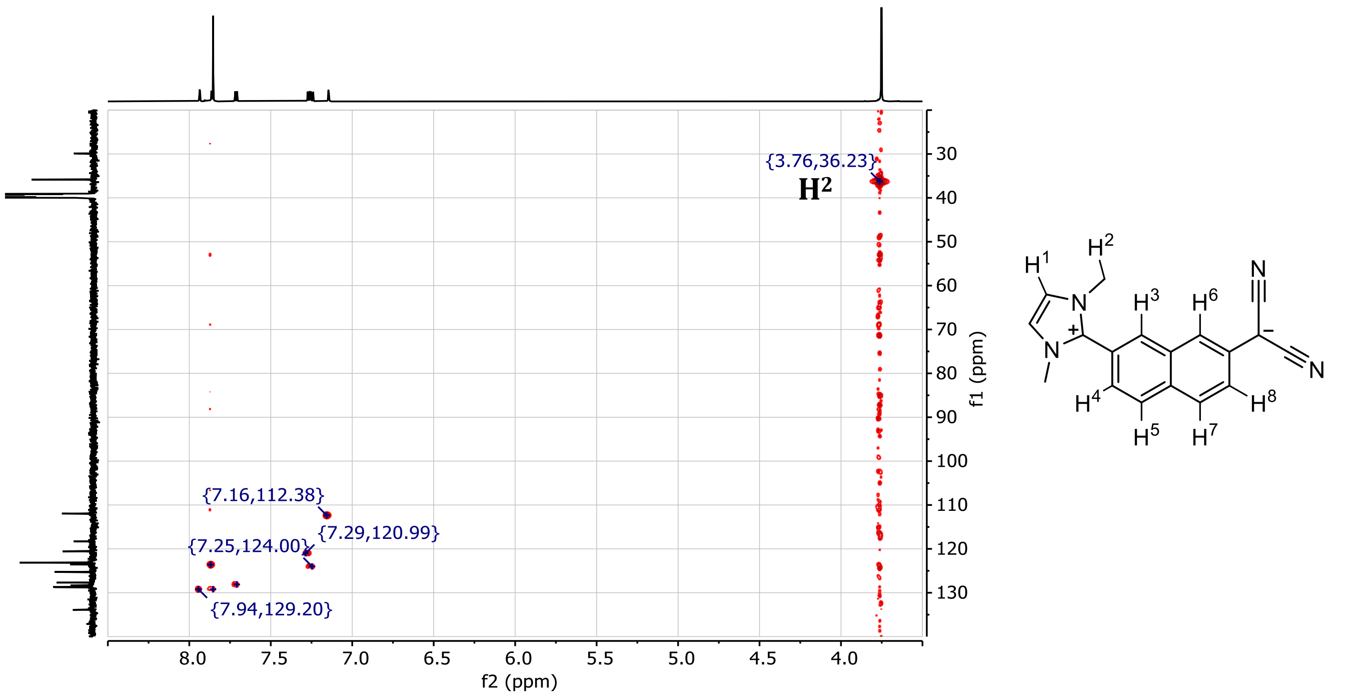

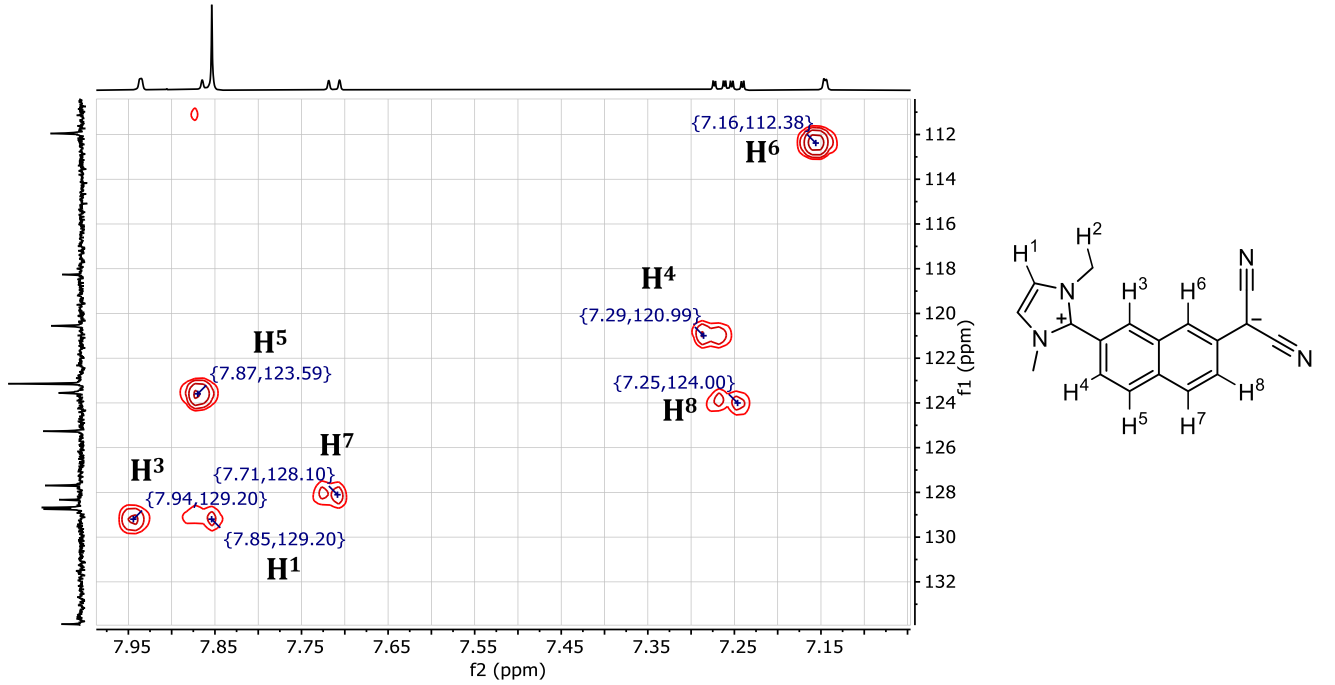

HMBC and expansion for **pseudo-*m*-NB** in DMSO-d_6 (peak shifting in x- and y-directions is an instrument artifact).

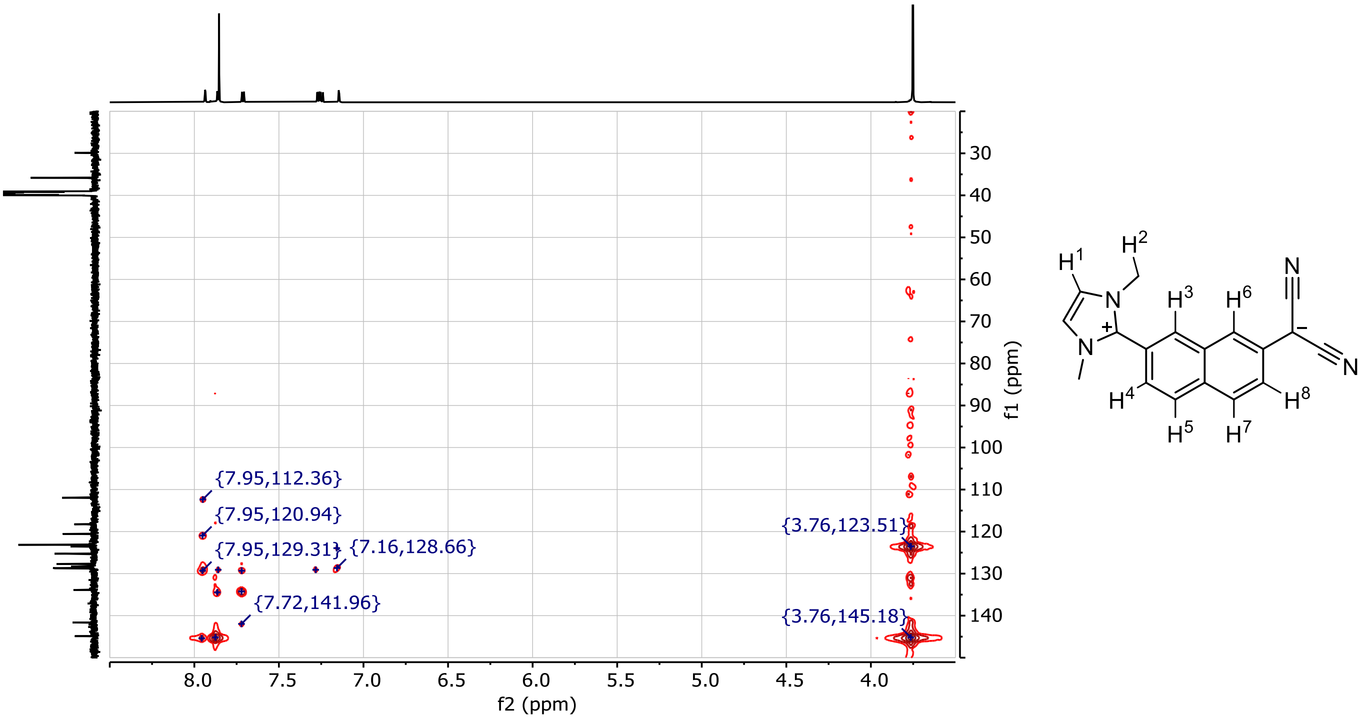

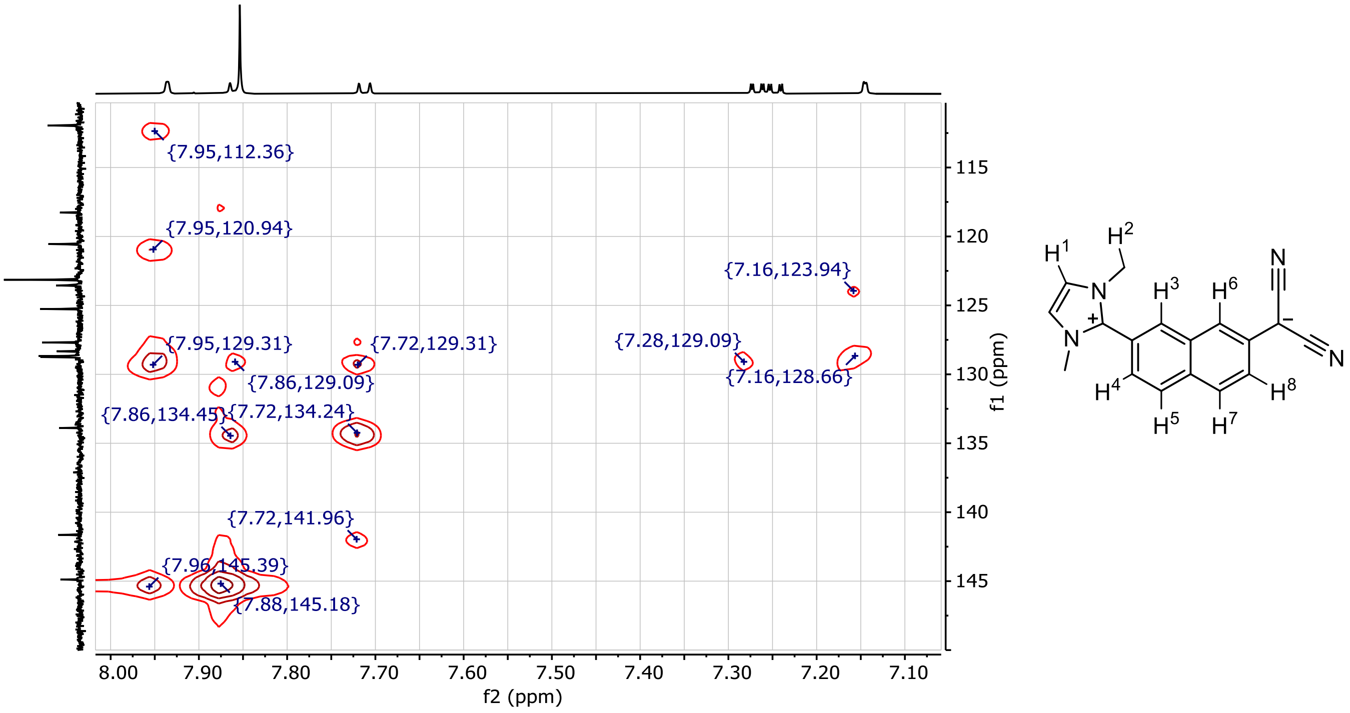

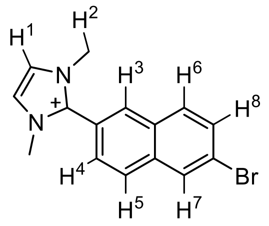

30 Using the known positions of H^2 at 3.75 ppm and H^1 at 7.91 ppm, these protons correlate through HMBC to the imidazolium N-C-N at 143.9 ppm which had no attached protons. Coupling constants group H^3, H^4, and H^5 separately from H^6, H^7 and H^8. Once grouped, an HMBC correlation between the N-C-N carbon and H^3 at 8.45 ppm and H^4 at 7.84 ppm assigns that group of protons to the ring with the imidazolium substituent.

HMQC and expansion for **30** in DMSO-d_6 (peak shifting in x- and y-directions is an instrument artifact).

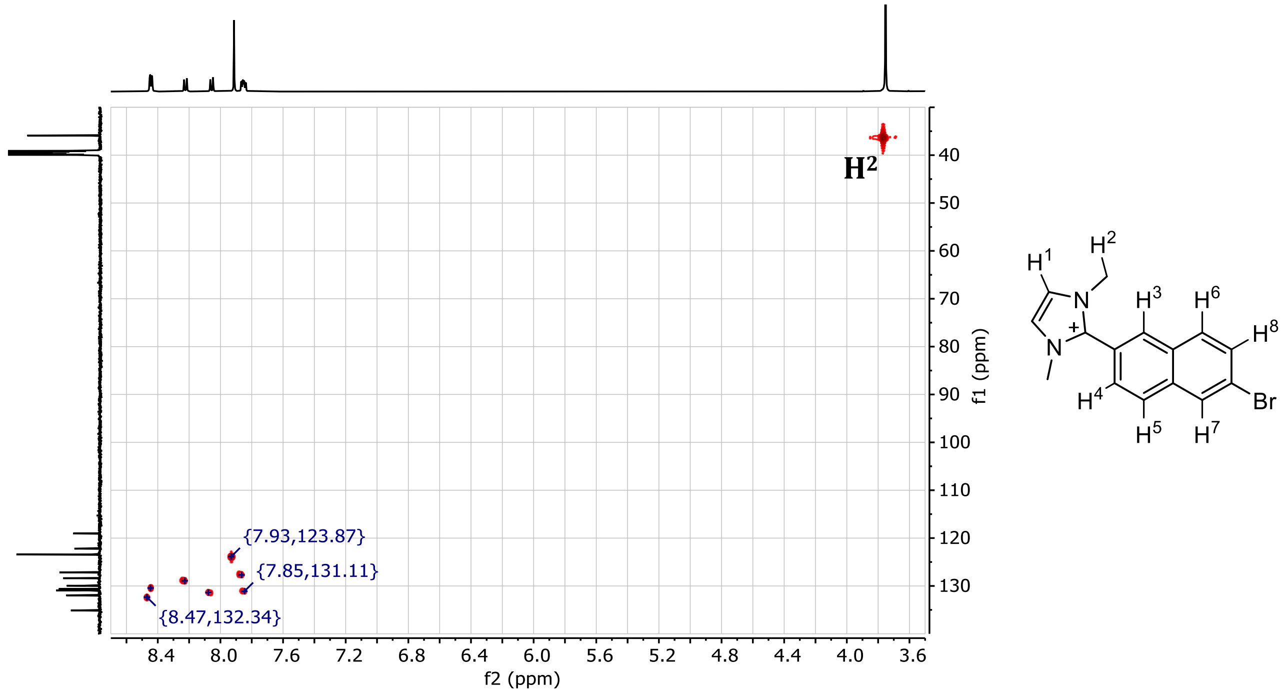

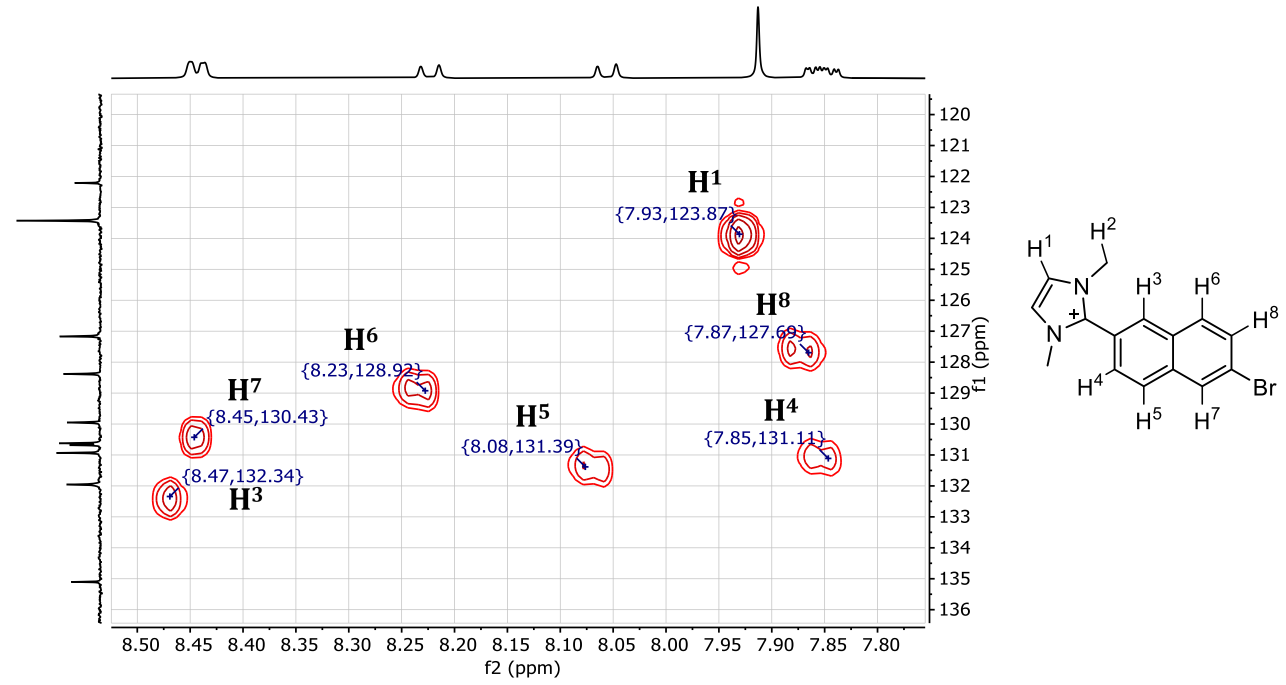

HMBC and expansion for **30** in DMSO-d_6 (peak shifting in x- and y-directions is an instrument artifact).

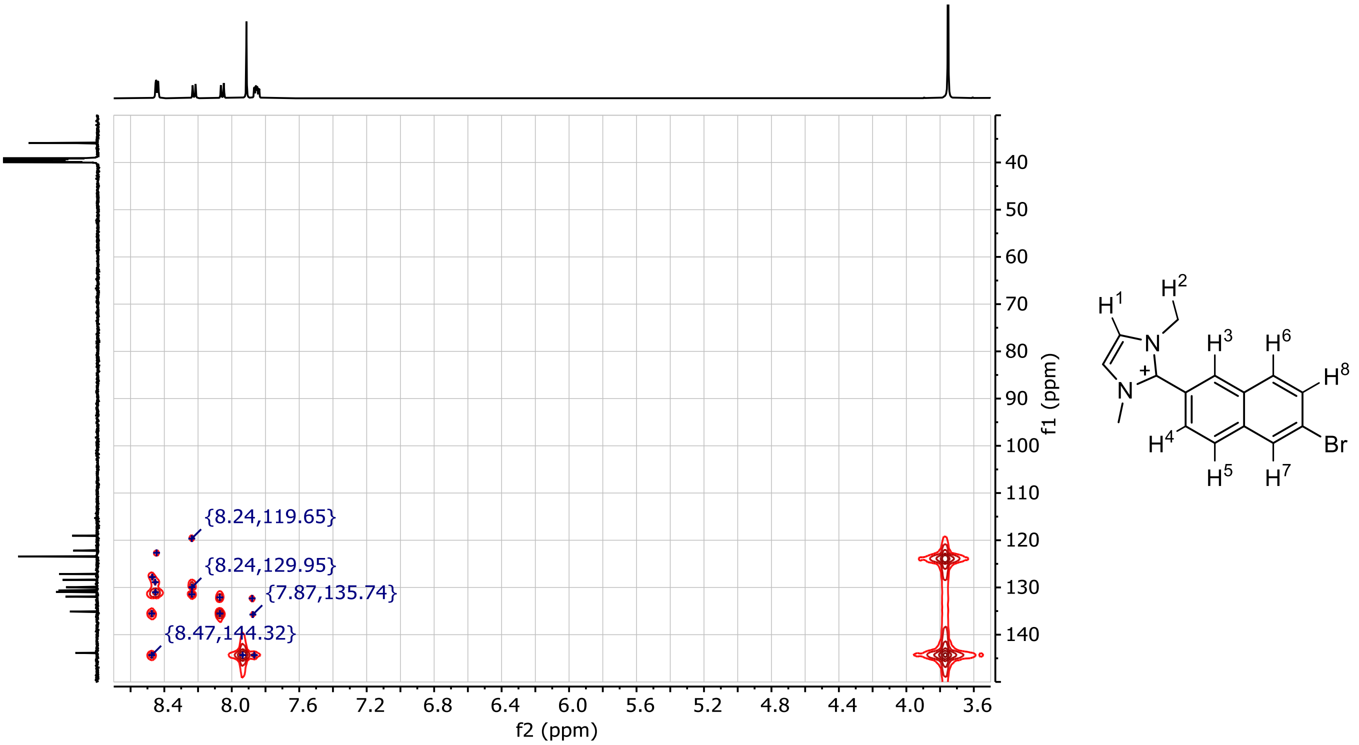

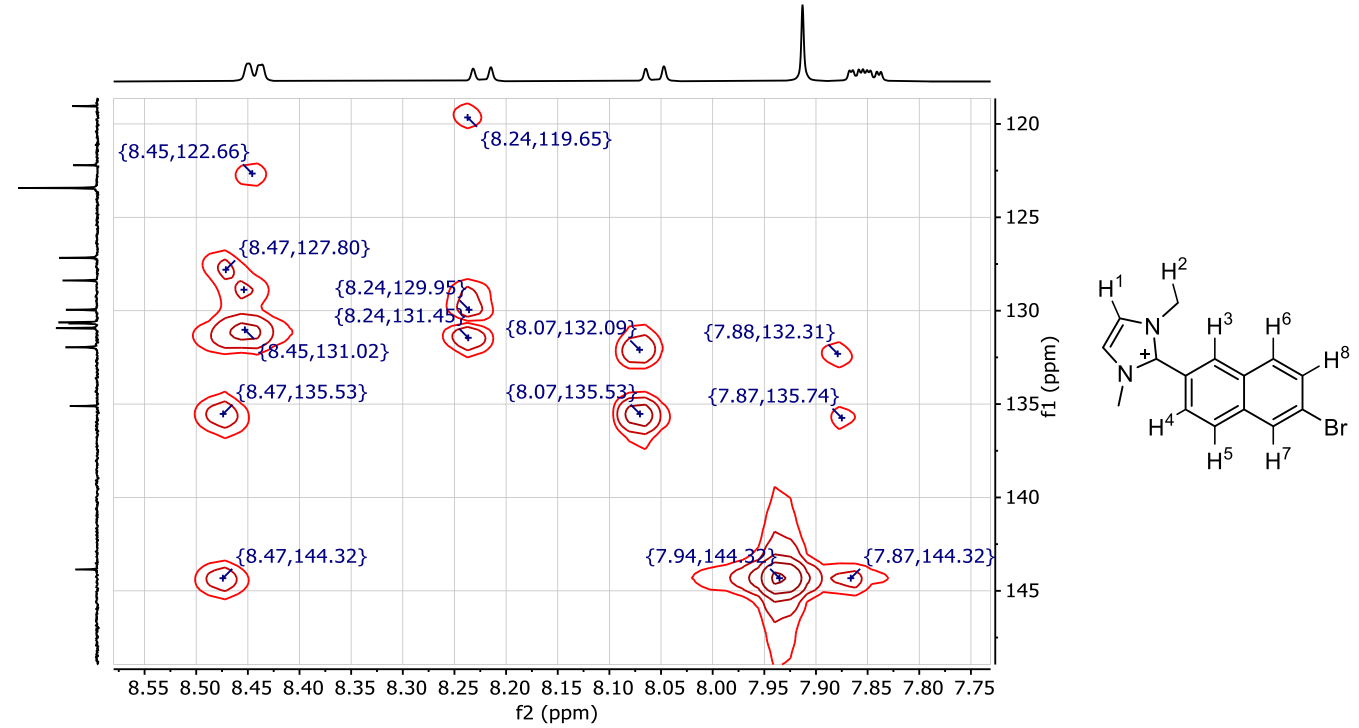

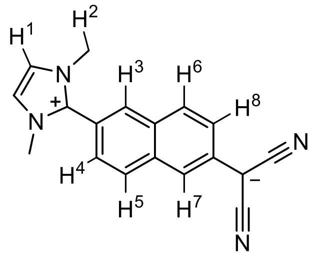

pseudo-*p*-NB Using the known positions of H2 at 3.75 ppm and H1 at 7.84 ppm, these protons correlate through HMBC to the imidazolium N-C-N at 145.0 ppm which had no attached protons. Coupling constants group H3, H4, and H5 separately from H6, H7 and H8. Once grouped, an HMBC correlation between the N-C-N carbon and the *meta*-coupled doublet H3 at 8.01 and H4 at 7.47 ppm ppm assigned that group of protons to the to the ring with the imidazolium substituent. No correlations between protons and the malonide carbon are observed.

HMQC and expansion for **pseudo-*p*-NB** in DMSO-d_6 (peak shifting in x- and y-directions is an instrument artifact).

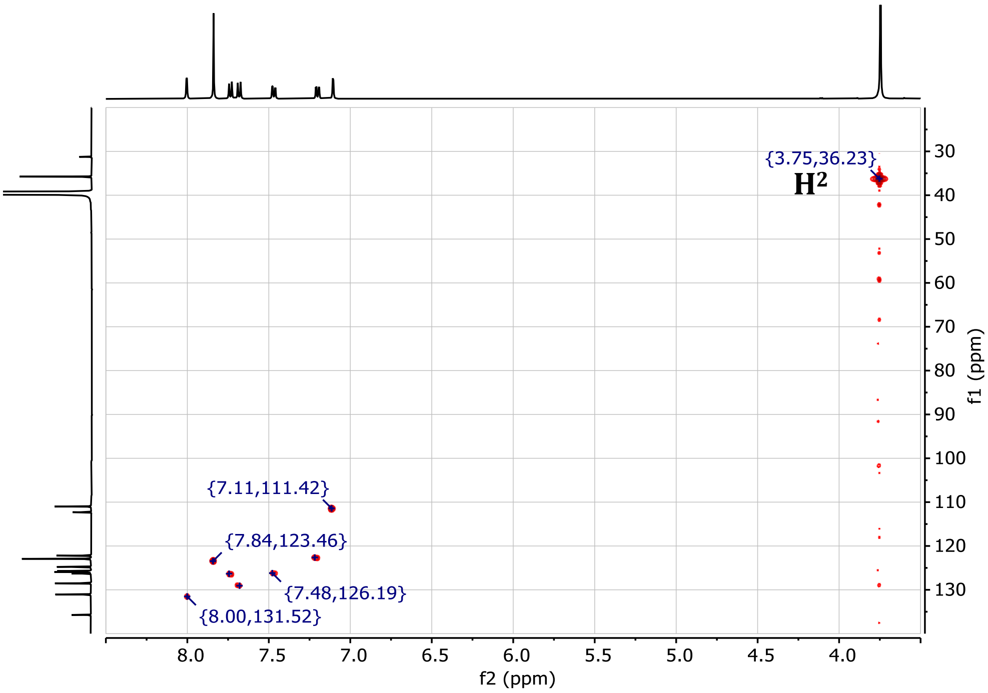

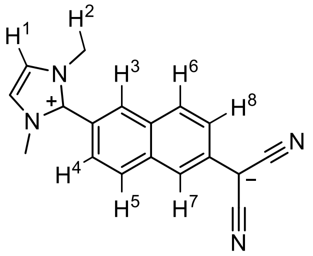

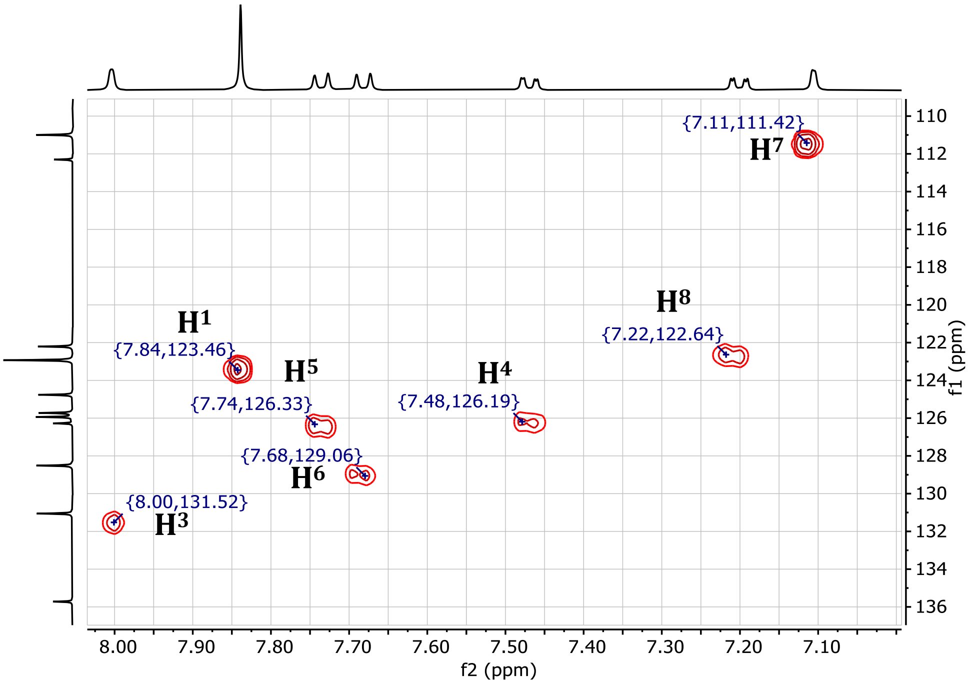

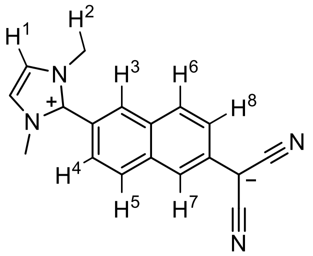

HMBC and expansion for **pseudo-*p*-NB** in DMSO-d_6 (peak shifting in x- and y-directions is an instrument artifact).

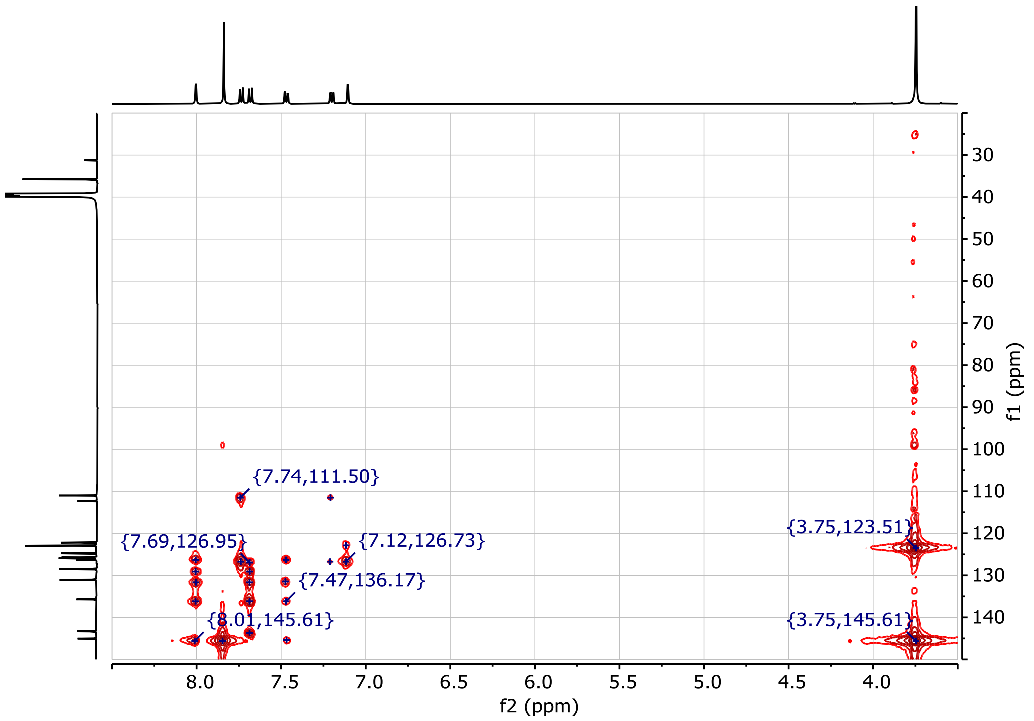

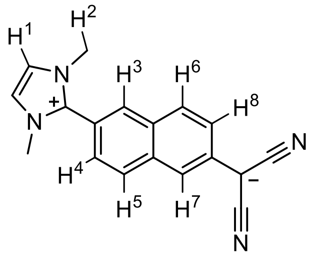

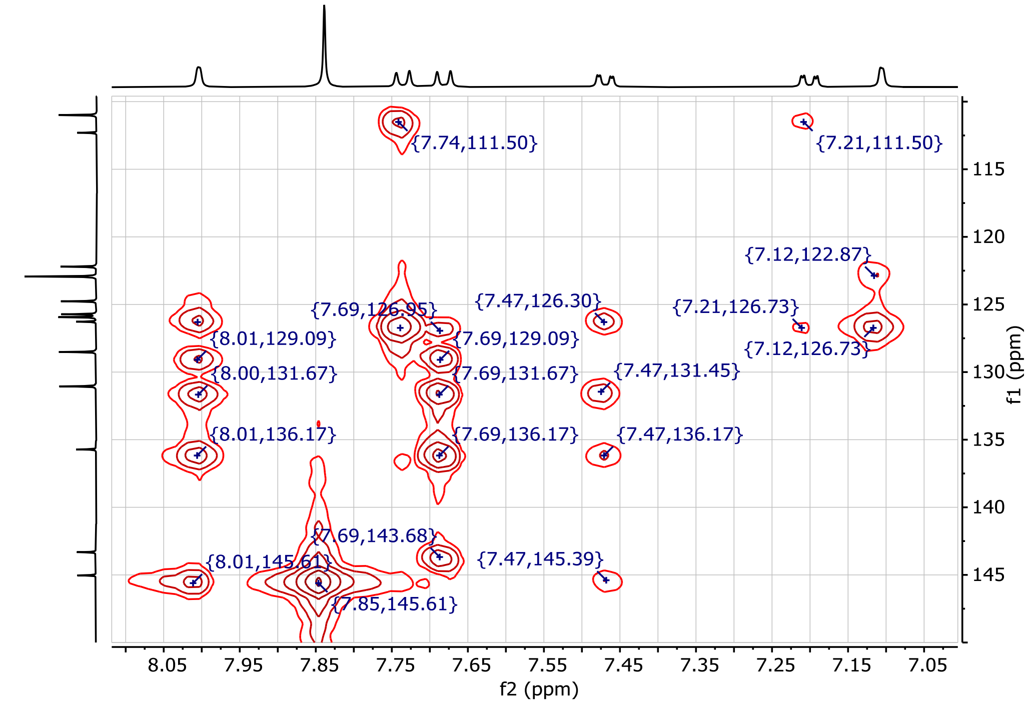

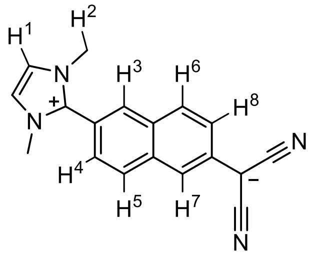

Section S10 Electrochemistry

Table S15. Formal reduction ($E^{0'}_{red}$) and oxidation ($E^{0'}_{ox}$) potentials of compounds measured by DPV in CH_3CN against the ferrocene/ferrocenium couple.

**Compound**	$E^{0'}_{red\,1}$**(V)**	$E^{0'}_{red\,2}$**(V)**	$E^{0'}_{ox\,1}$**(V)**	$E^{0'}_{ox\,2}$**(V)**	E^{elec}_{gap} **(eV)**
phenylimidazolium iodide **3**	-2.466	-	-0.010	+0.320	-
sodium phenylmalonide	-	-	-0.190	-	-
naphthylimidazolium iodide **17**	-3.007	-2.351	-0.199	0.325	-
sodium naphthylmalonide	-	-	-0.290	-	-
m-PB	-2.483	-	-0.075	-	2.558
***p*-PB**	-2.727	-	-0.055	-	2.782
**pseudo-*m*-NB**	-2.366	-	-0.045	-	2.411
**pseudo-*p*-NB**	-2.521	-	-0.095	-	2.616

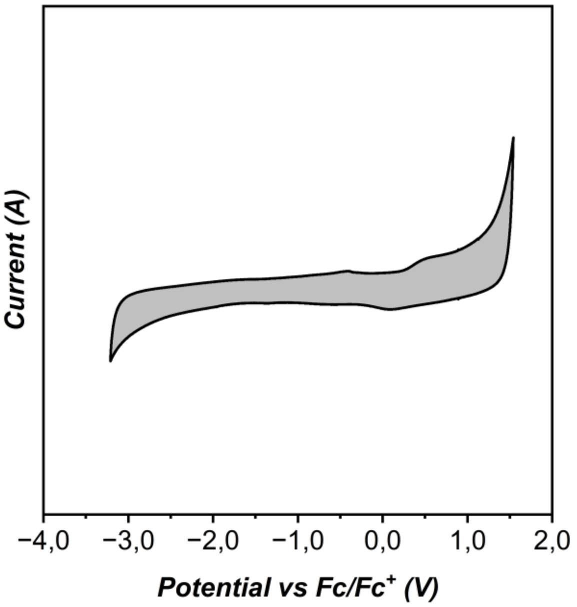

Figure S17. Cyclic voltammogram of blank $TBAPF_6$ in CH_3CN at 500 mV·s^{-1}.

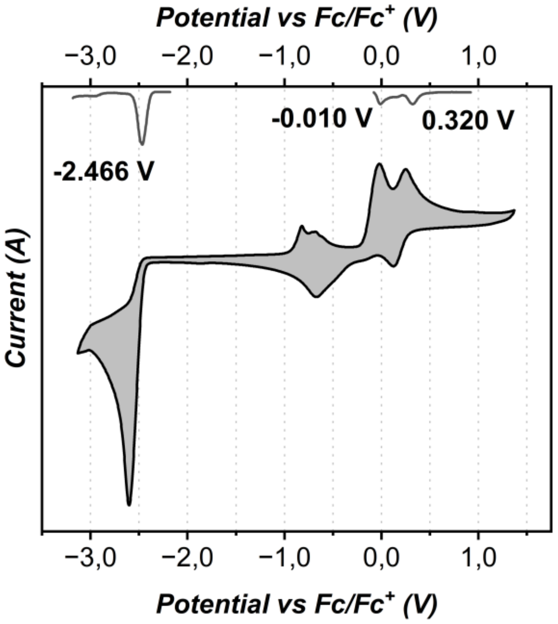

Figure S18. Cyclic voltammogram and inset DPV of full electrochemical window of phenylimidazolium iodide **3** in CH_3CN at 500 mV·s^{-1}.

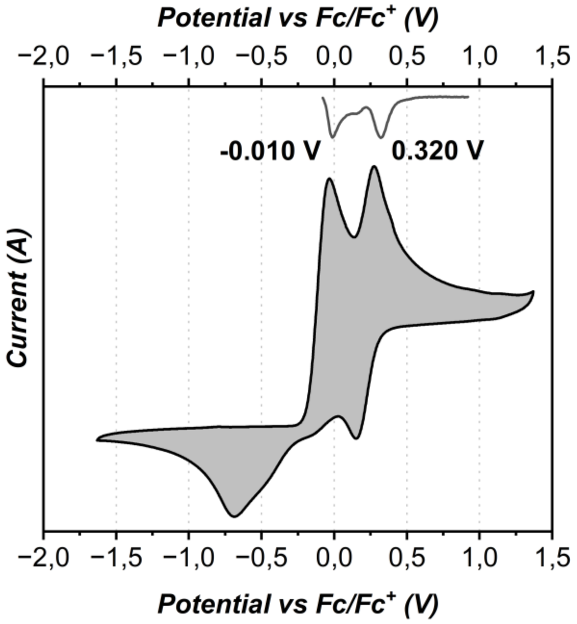

Figure S19. Cyclic voltammogram and inset DPV of anodic region of phenylimidazolium iodide **3** in CH_3CN at 500 mV·s^{-1}.

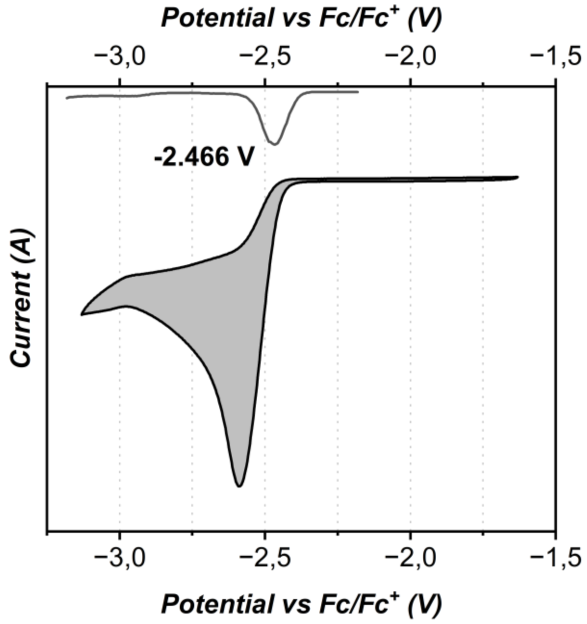

Figure S20. Cyclic voltammogram and inset DPV of cathodic window of phenylimidazolium iodide **3** in CH_3CN at 500 mV·s^{-1}.

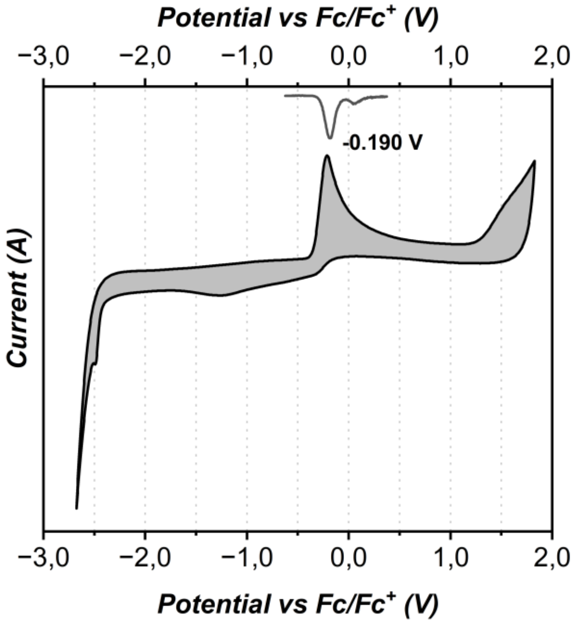

Figure S21. Cyclic voltammogram and inset DPV of full electrochemical window of sodium phenylmalonide in CH_3CN at 500 mV·s^{-1}.

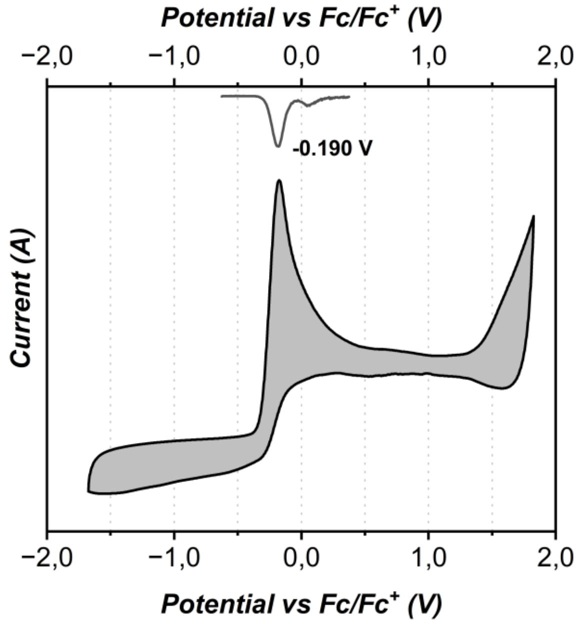

Figure S22. Cyclic voltammogram and inset DPV of anodic region of sodium phenylmalonide in CH_3CN at 500 mV·s^{-1}.

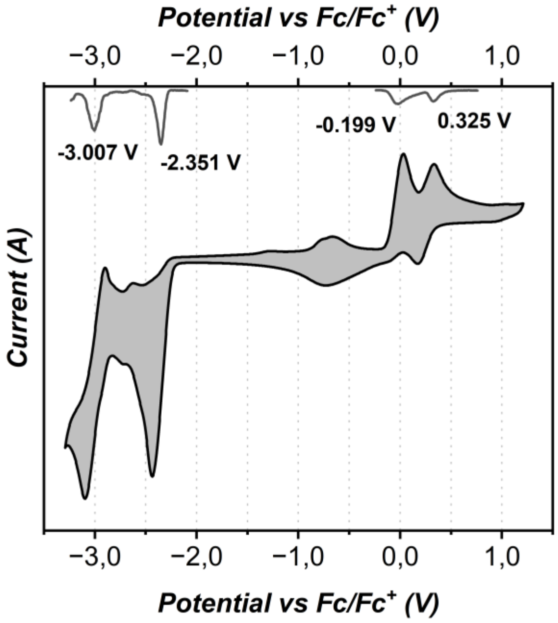

Figure S23. Cyclic voltammogram and inset DPV of full electrochemical window of naphthylimidazolium iodide **17** in CH_3CN at 500 mV·s^{-1}.

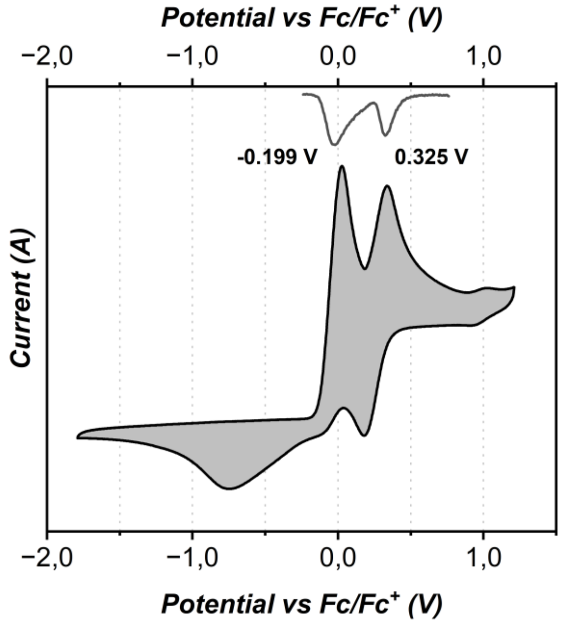

Figure S24. Cyclic voltammogram and inset DPV of anodic window of naphthylimidazolium iodide **17** in CH_3CN at 500 mV·s^{-1}.

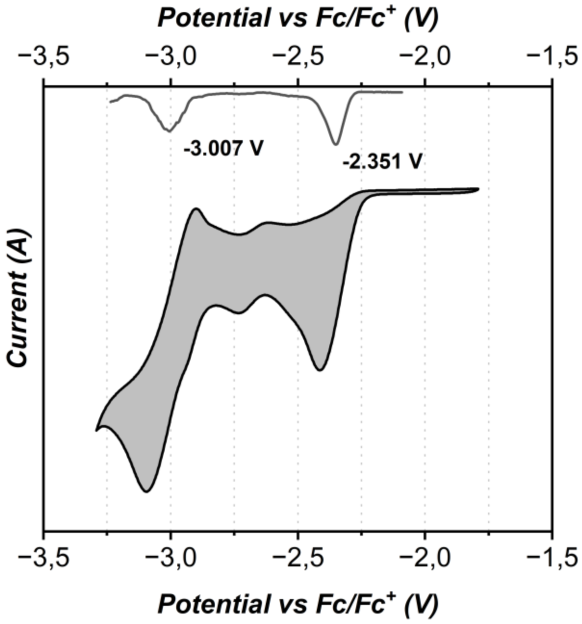

Figure S25. Cyclic voltammogram and inset DPV of cathodic region of naphthylimidazolium iodide **17** in CH_3CN at 500 mV·s^{-1}.

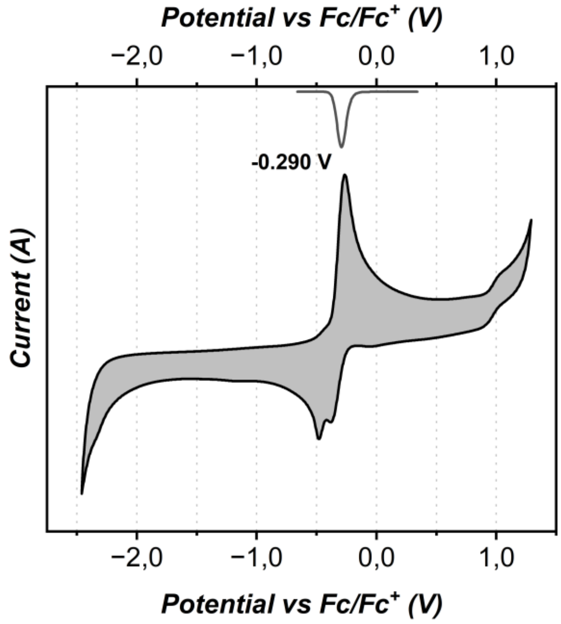

Figure S26. Cyclic voltammogram and inset DPV of full electrochemical window of sodium naphthylmalonide in CH_3CN at 500 mV·s^{-1}.

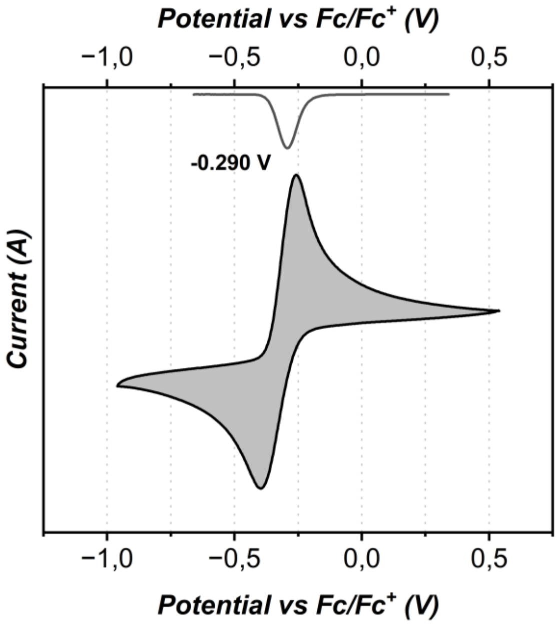

Figure S27. Cyclic voltammogram and inset DPV of anodic region of sodium naphthylmalonide in CH_3CN at 500 mV·s^{-1}.

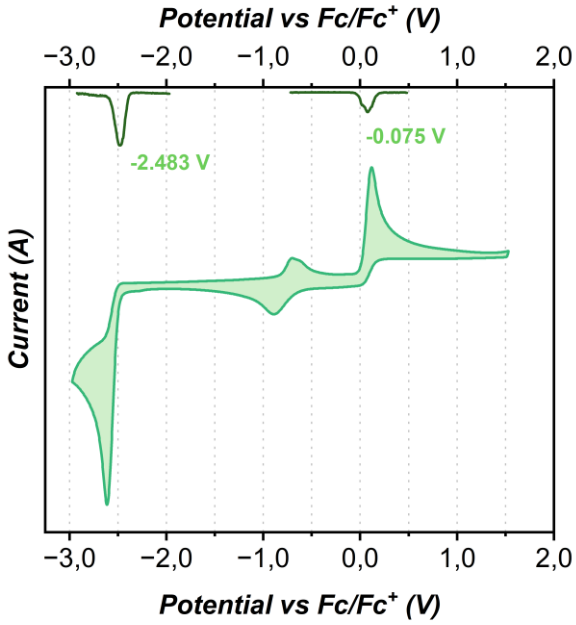

Figure S28. Cyclic voltammogram and inset DPV of full electrochemical window of ***m*-PB** in CH_3CN at 500 mV·s^{-1}.

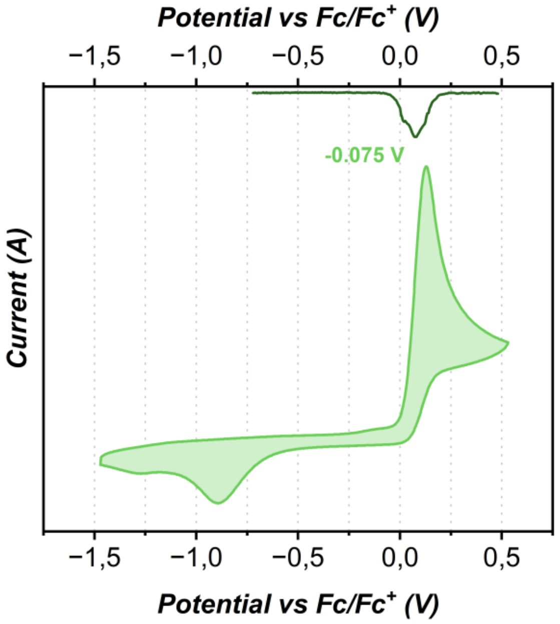

Figure S29. Cyclic voltammogram and inset DPV of anodic region of ***m*-PB** in CH_3CN at 500 mV·s^{-1}.

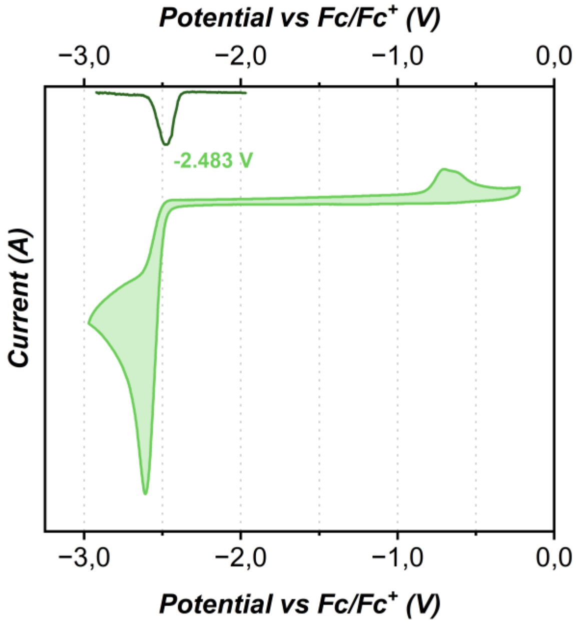

Figure S30. Cyclic voltammogram and inset DPV of cathodic region of ***m*-PB** in CH_3CN at 500 mV·s^{-1}.

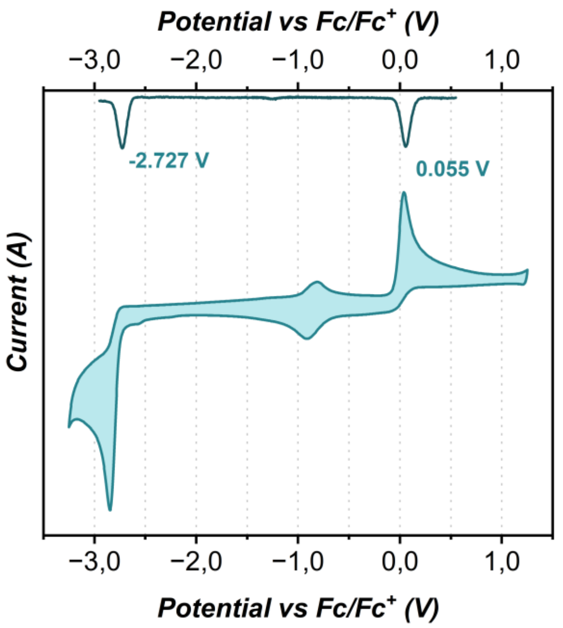

Figure S31. Cyclic voltammogram and inset DPV of full electrochemical window of ***p*-PB** in CH_3CN at 500 mV·s^{-1}.

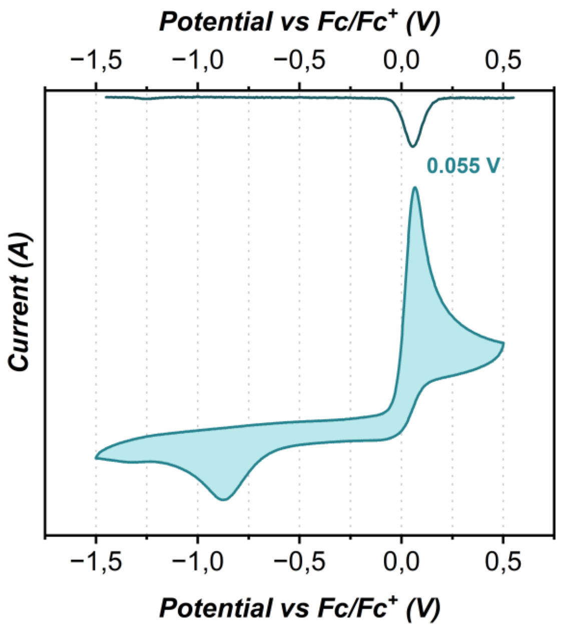

Figure S32. Cyclic voltammogram and inset DPV of anodic region of ***p*-PB** in CH_3CN at 500 mV·s^{-1}.

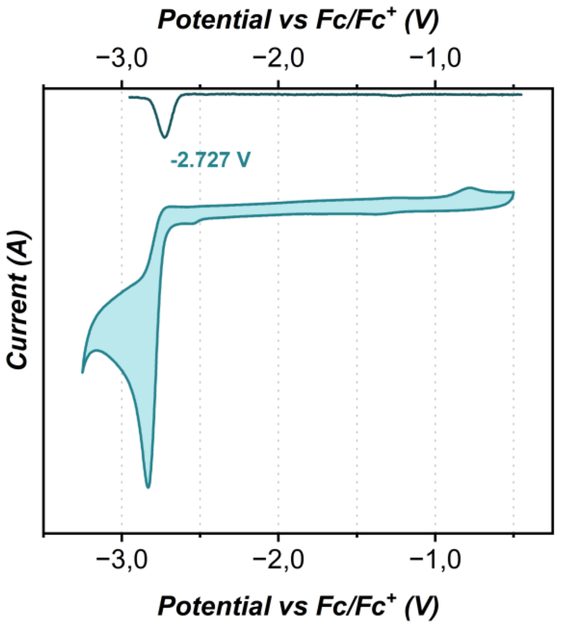

Figure S33. Cyclic voltammogram and inset DPV of cathodic region of ***p*-PB** in CH_3CN at 500 mV·s^{-1}.

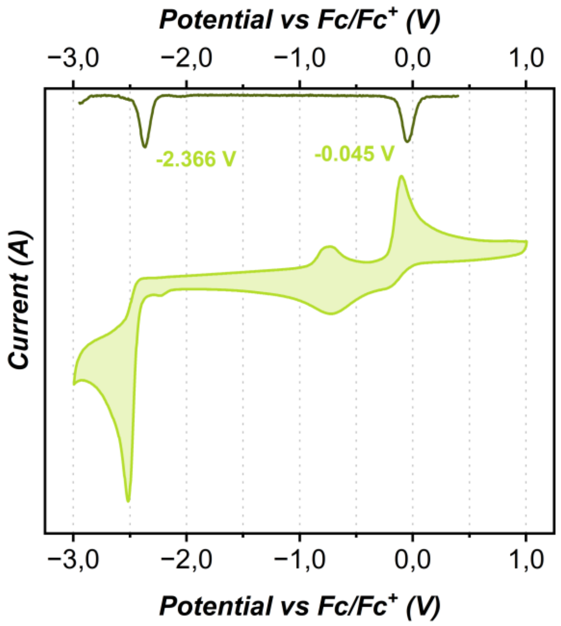

Figure S34. Cyclic voltammogram and inset DPV of full electrochemical window of **pseudo-*m*-NB** in CH_3CN at 500 mV·s^{-1}.

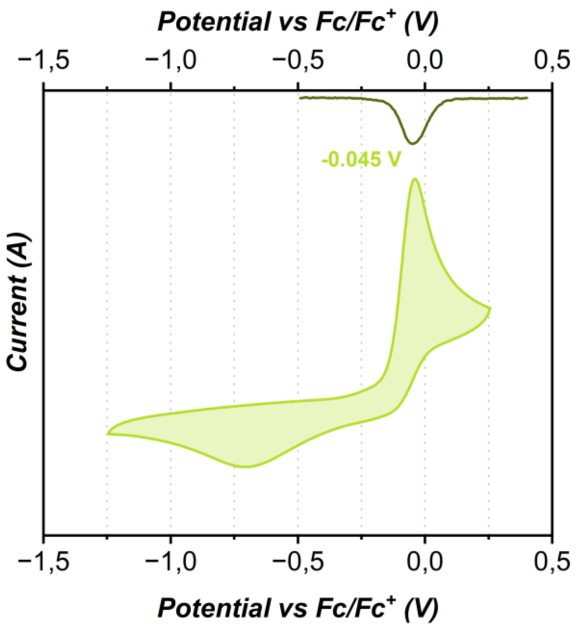

Figure S35. Cyclic voltammogram and inset DPV of anodic region of **pseudo-*m*-NB** in CH_3CN at 500 mV·s^{-1}.

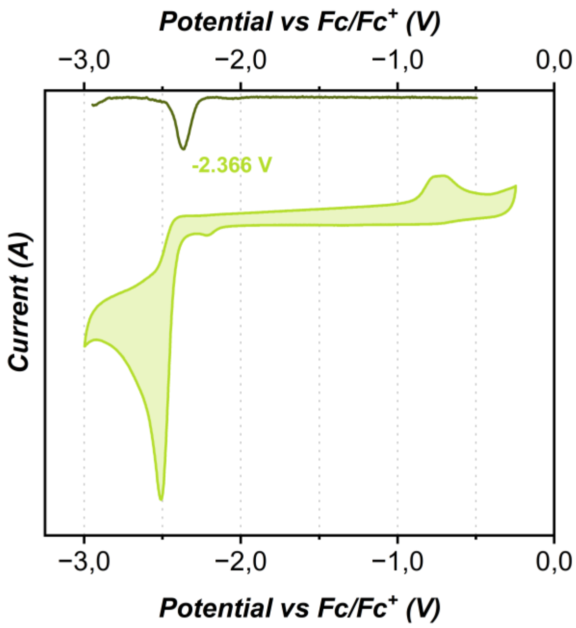

Figure S36. Cyclic voltammogram and inset DPV of cathodic region of **pseudo-*m*-NB** in CH_3CN at 500 mV·s^{-1}.

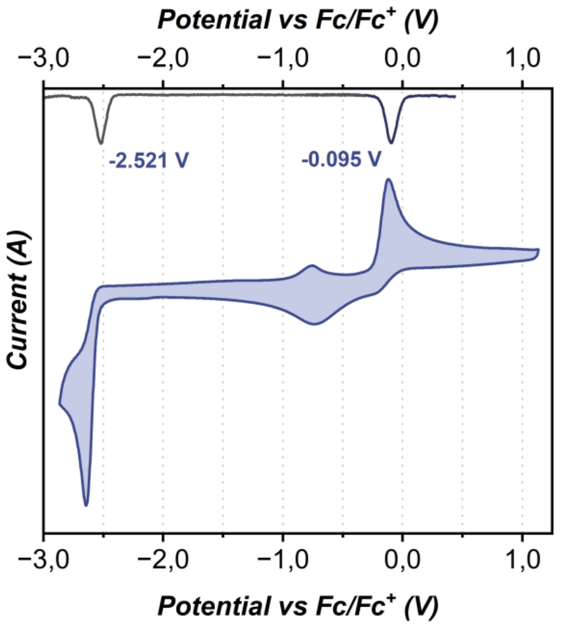

Figure S37. Cyclic voltammogram and inset DPV of full electrochemical window of **pseudo-*p*-NB** in CH_3CN at 500 mV·s^{-1}.

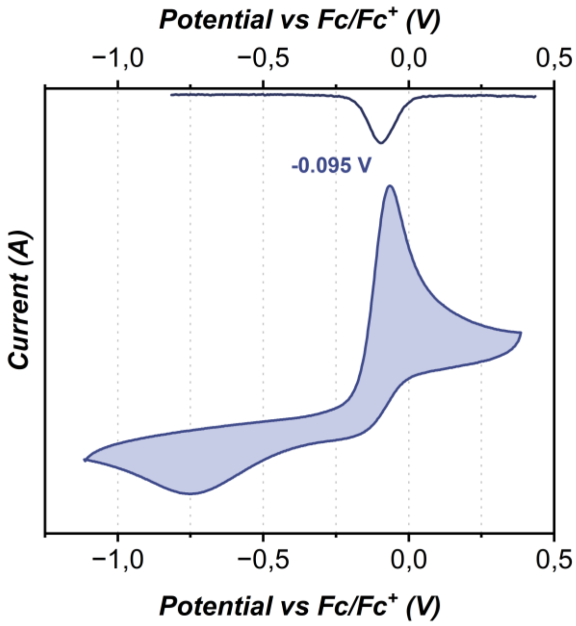

Figure S38. Cyclic voltammogram and inset DPV of anodic region of **pseudo-*p*-NB** in CH_3CN at 500 mV·s^{-1}.

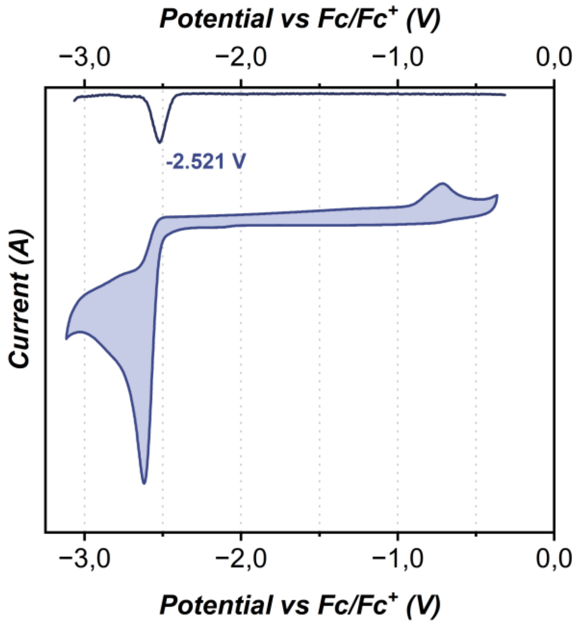

Figure S39. Cyclic voltammogram and inset DPV of cathodic region of **pseudo-*p*-NB** in CH_3CN at 500 mV·s^{-1}.

Section S11 Model Compounds Photophysics

Table S16. Absorbance maxima, molar absorption coefficients, and fluorescence data of model compounds in CH_3CN.

**Compound**	**Longest-wavelength Absorbance Maximum, λ_{abs} (nm)**	**Molar Absorption Coefficient at λ_{abs}, ε (L · mol^{-1} · cm^{-1})**	**Fluorescence Maximum, λ_{em} (nm)**	**Fluorescence Quantum Yield, Φ_F**
Benzene	259	120	not measured	not measured
Naphthalene	285	4 000	not measured	not measured
phenylimidazolium iodide **3**	244	22 000	315	0.02 ± 0.01
naphthylimidazolium iodide **17**	243	45 800	357	0.13 ± 0.01
sodium phenylmalonide	2 98	22 600	non fluorescent	non fluorescent
Sodium naphthylmalonide	398	2 200	non fluorescent	non fluorescent

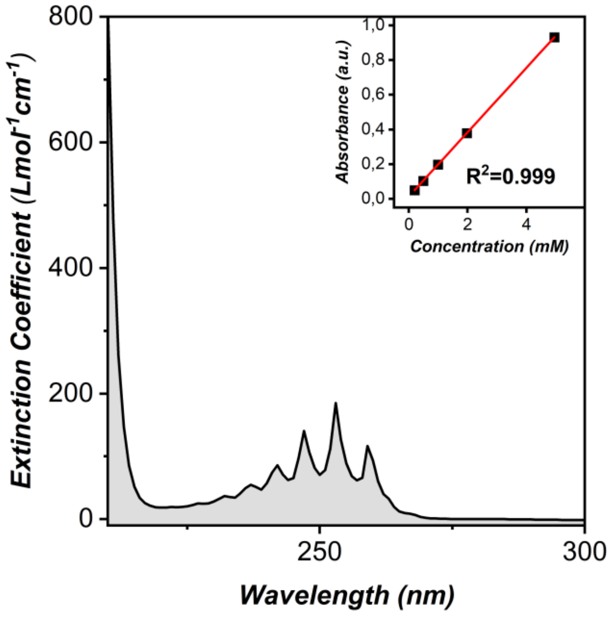

Figure S40. Absorption profile of benzene in CH_3CN with inset of absorbance at 253 nm at various concentrations.

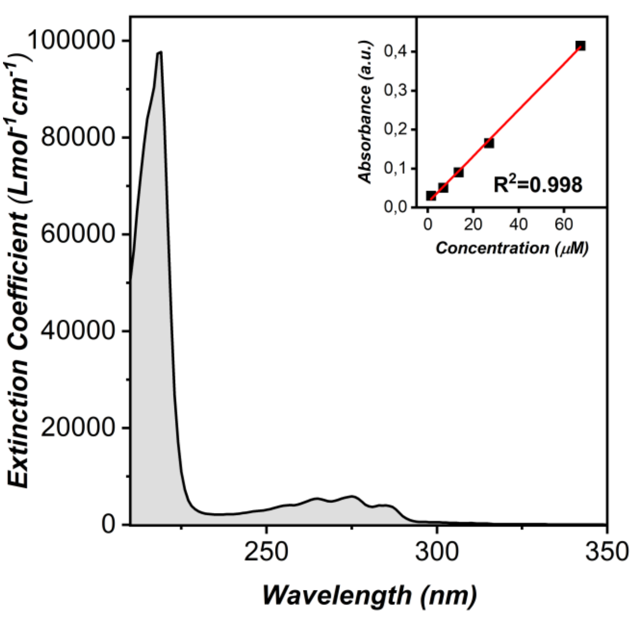

Figure S41. Absorption profile of naphthalene in CH_3CN with inset of absorbance at 275 nm at various concentrations.

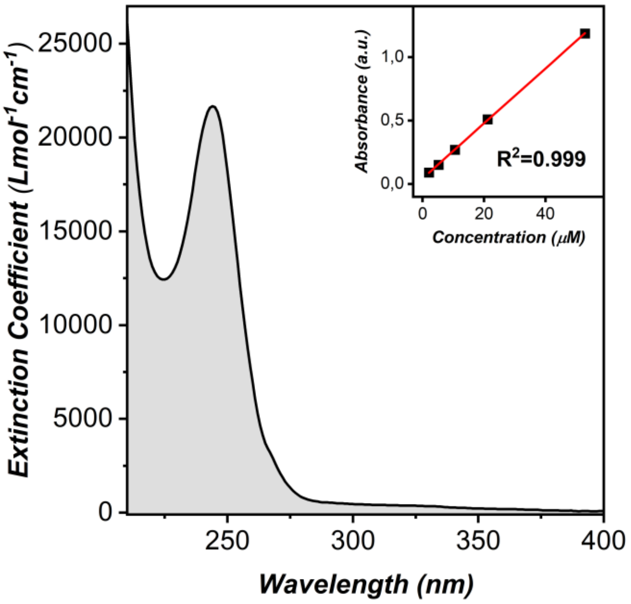

Figure S42. Absorption profile of phenylimidazolium iodide **3** in CH_3CN with inset of absorbance at 244 nm at various concentrations.

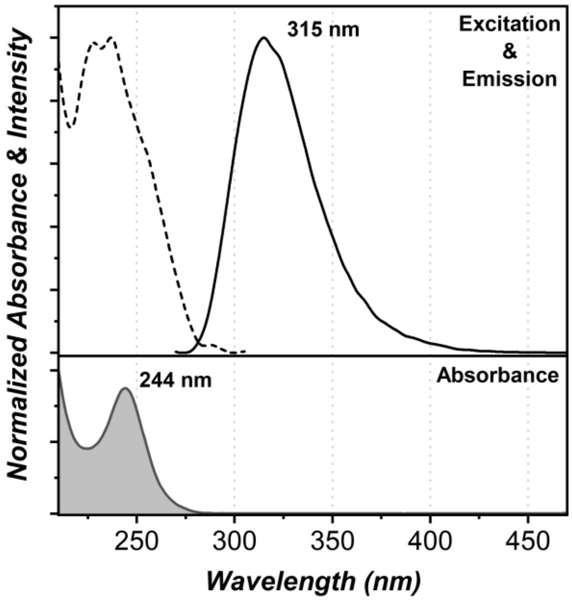

Figure S43. Excitation, emission (λ_{exc} 245 nm), and absorption spectra of phenylimidazolium iodide **3** in CH_3CN.

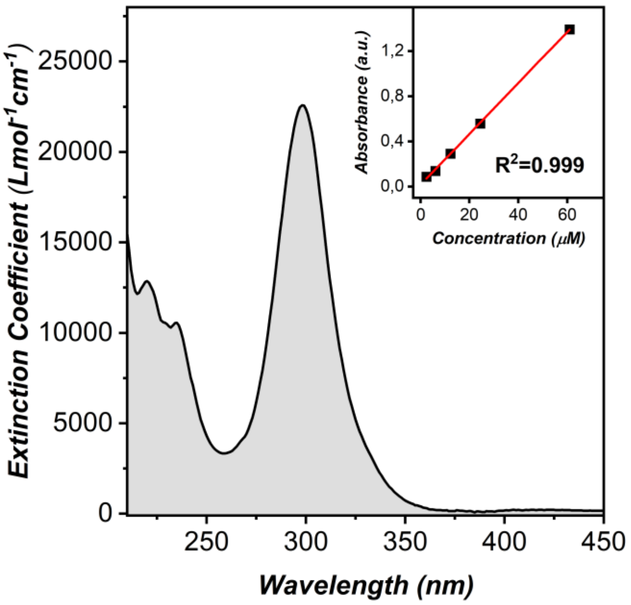

Figure S44. Absorption profile of sodium phenylmalonide in CH_3CN with a drop of 1 M NaOH/H_2O with inset of absorbance at 298 nm at various concentrations.

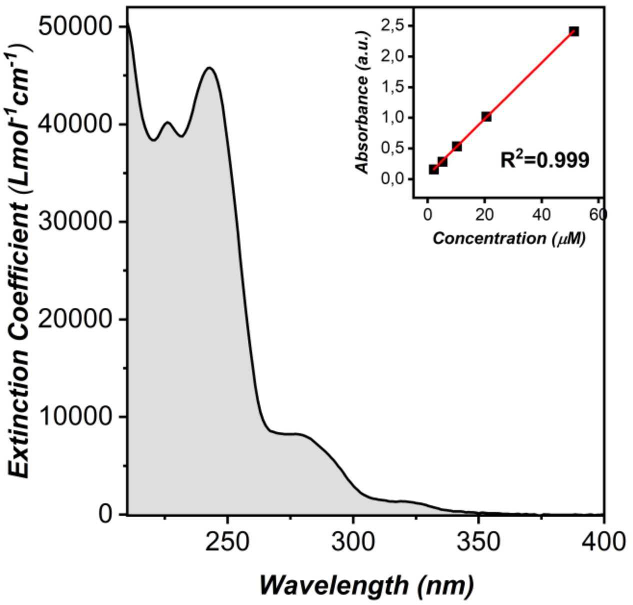

Figure S45. Absorption profile of naphthylimidazolium iodide **17** in CH_3CN with inset of absorbance at 243 nm at various concentrations.

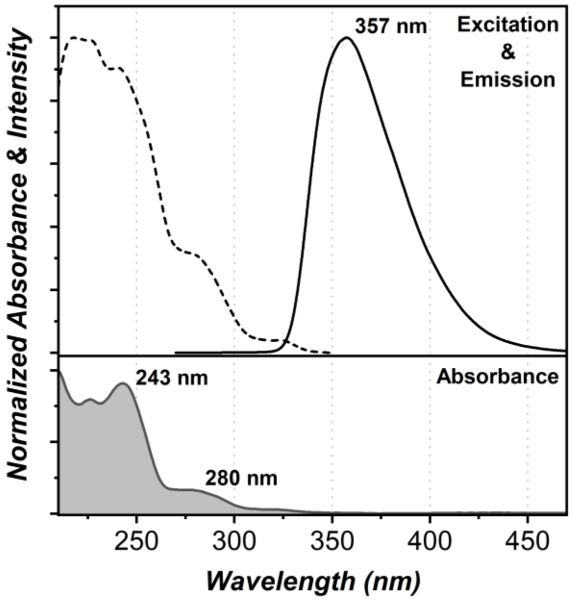

Figure S46. Excitation, emission (λ_{exc} 250 nm), and absorption spectra of naphthylimidazolium iodide **17** in CH_3CN.

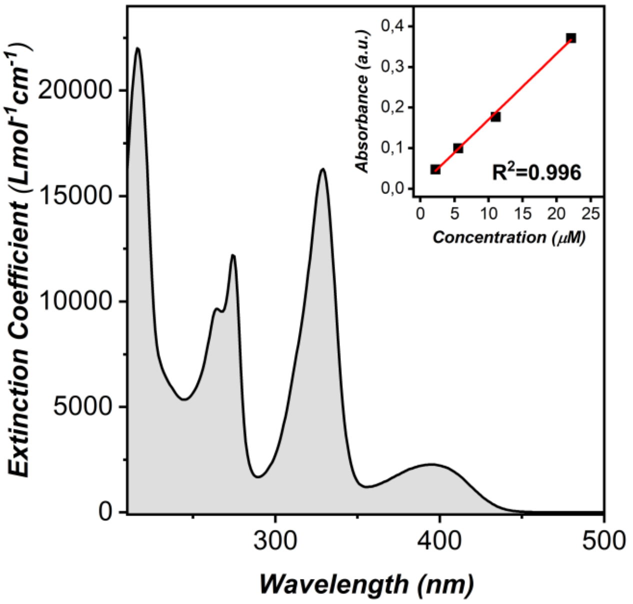

Figure S47. Absorption profile of sodium naphthylmalonide in CH_3CN with a drop of 1 M NaOH/H_2O with inset of absorbance at 307 nm at various concentrations.

Section S12 Concentration-dependent Photophysics

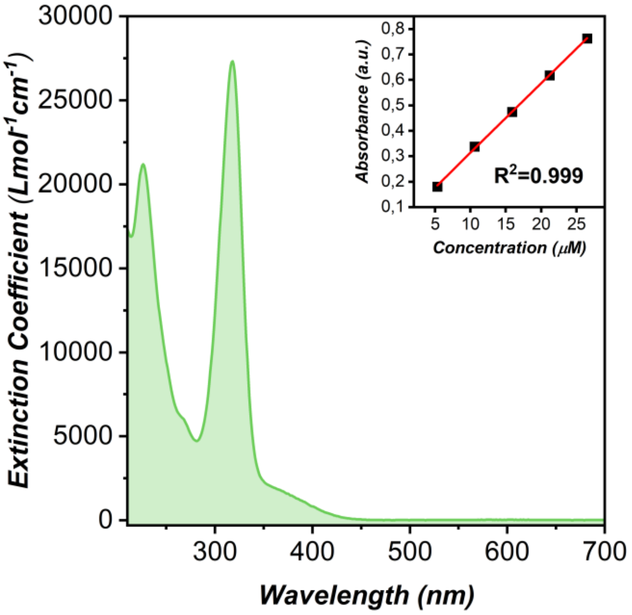

Figure S48. Absorption profile of ***m*-PB** in CH_3CN with inset of absorption at 318 nm versus concentration.

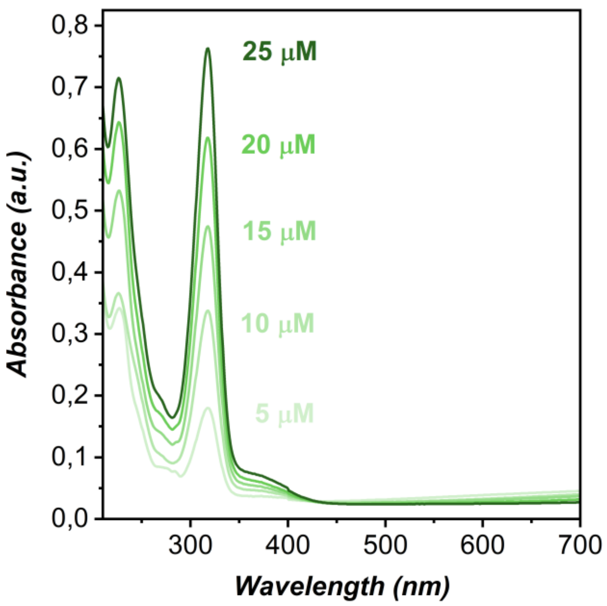

Figure S49. Absorption profiles of ***m*-PB** in CH_3CN at various concentrations.

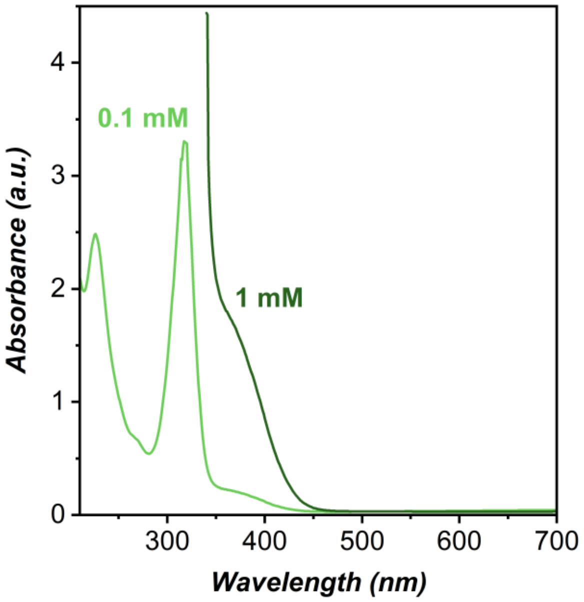

Figure S50. Absorption profiles of ***m*-PB** in CH_3CN at high concentrations.

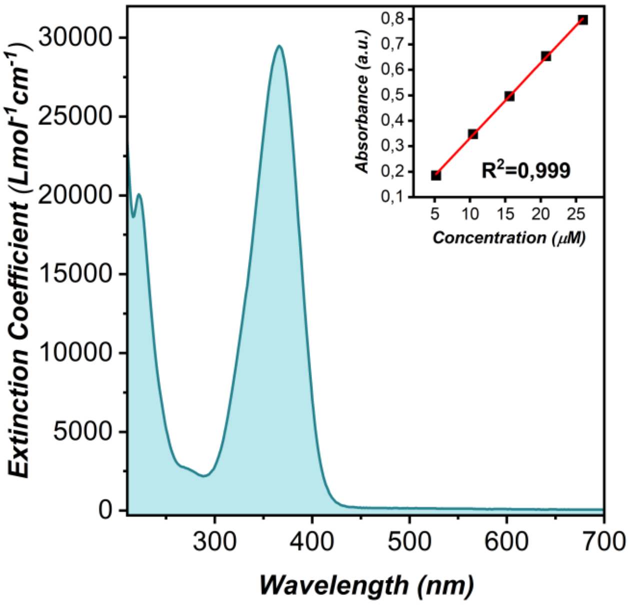

Figure S51. Absorption profile of ***p*-PB** in CH_3CN with inset of absorption at 365 nm versus concentration.

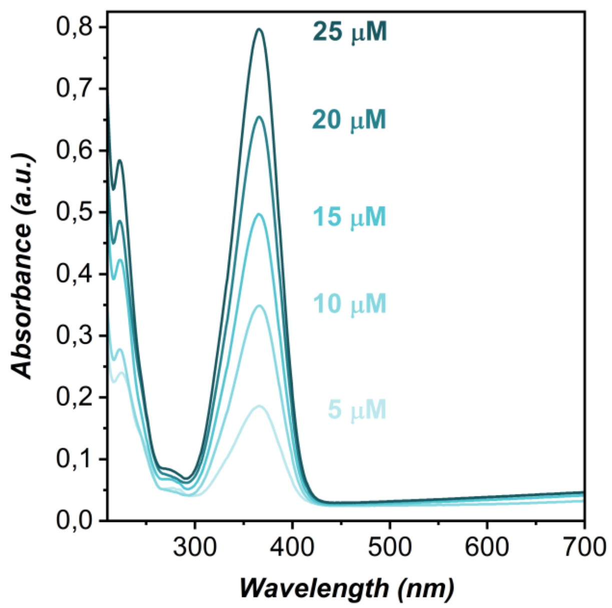

Figure S52. Absorption profiles of ***p*-PB** in CH_3CN at various concentrations.

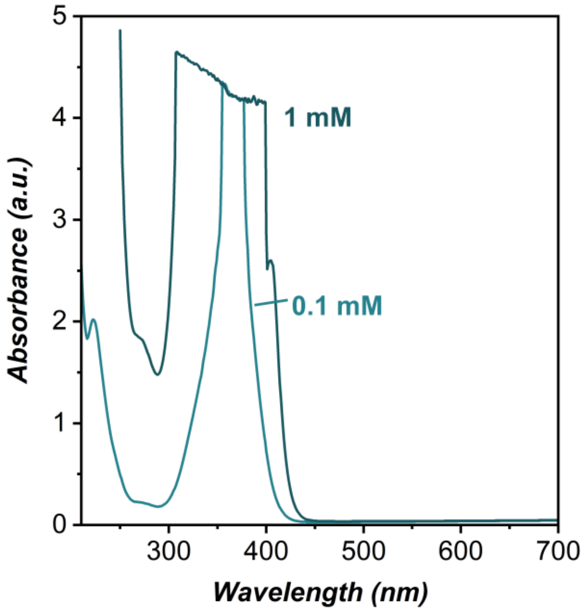

Figure S53. Absorption profiles of ***p*-PB** in CH_3CN at high concentrations. The artifact at 400 nm is instrumental in nature, originating from the vis-to-UV detector switch.

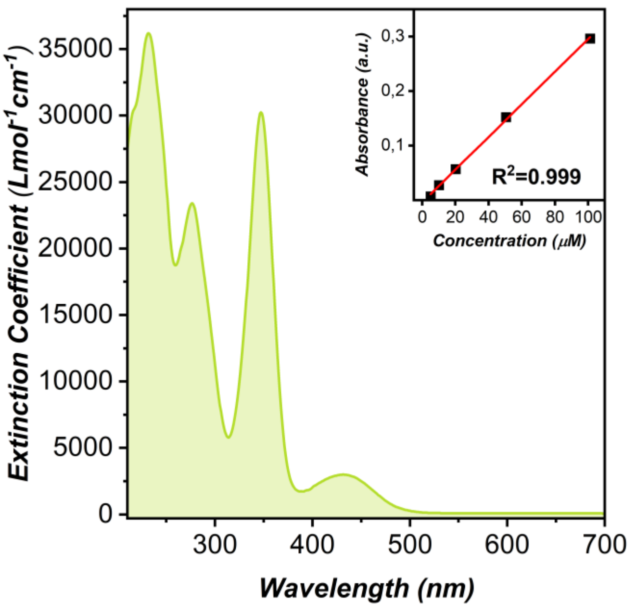

Figure S54. Absorption profile of **pseudo-*m*-NB** in CH_3CN with inset of absorption at 431 nm versus concentration.

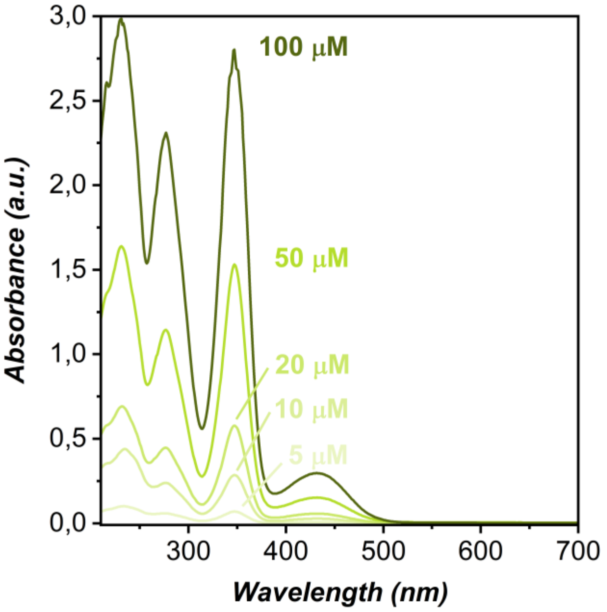

Figure S55. Absorption profiles of **pseudo-*m*-NB** in CH_3CN at various concentrations.

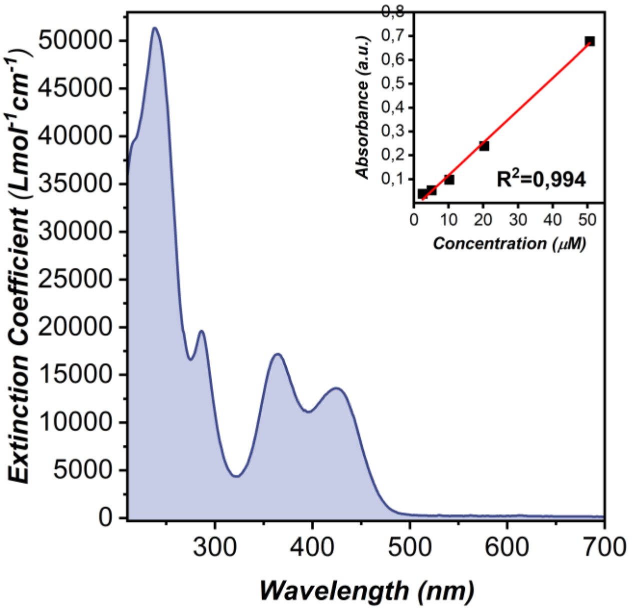

Figure S56. Absorption profile of **pseudo-*p*-NB** in CH_3CN with inset of absorption at 425 nm versus concentration.

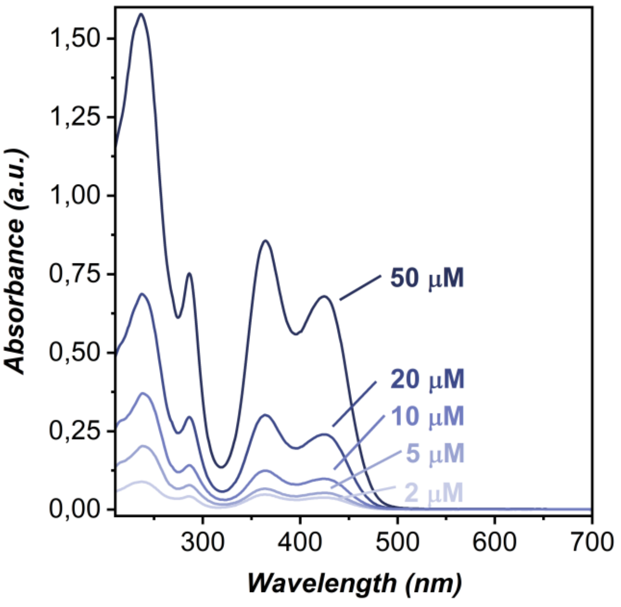

Figure S57. Absorption profiles of **pseudo-*p*-NB** in CH_3CN at various concentrations.

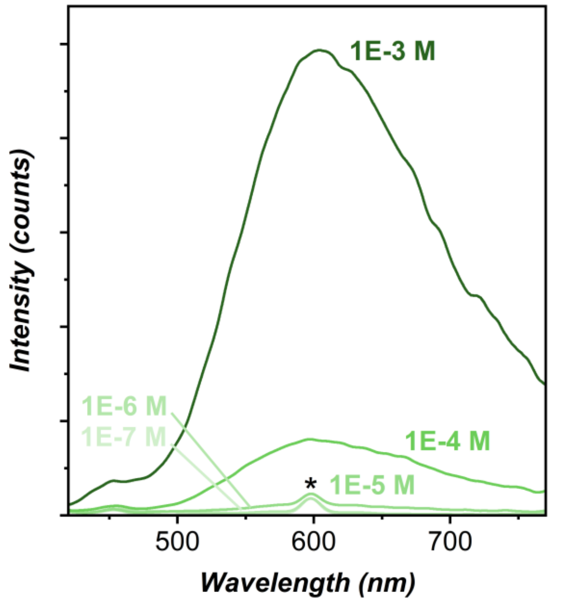

Figure S58. Fluorescence spectra of ***m*-PB** from excitation at 400 nm in CH_3CN at various concentrations. (* CH_3CN Raman peak)

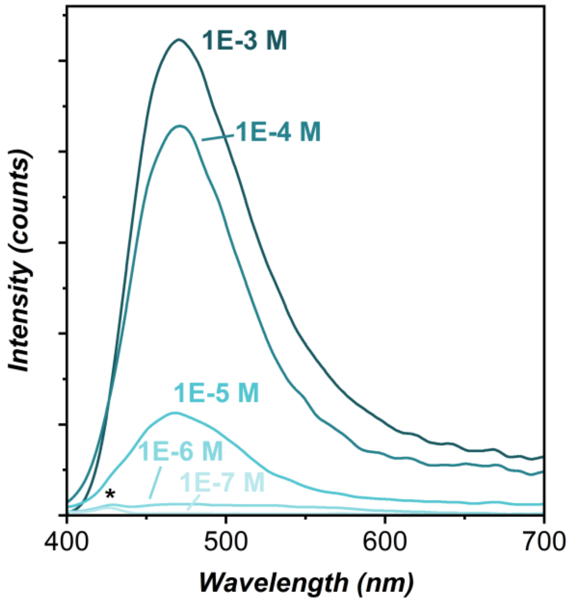

Figure S59. Fluorescence spectra of ***p*-PB** from excitation at 380 nm in CH_3CN at various concentrations. (* CH_3CN Raman peak)

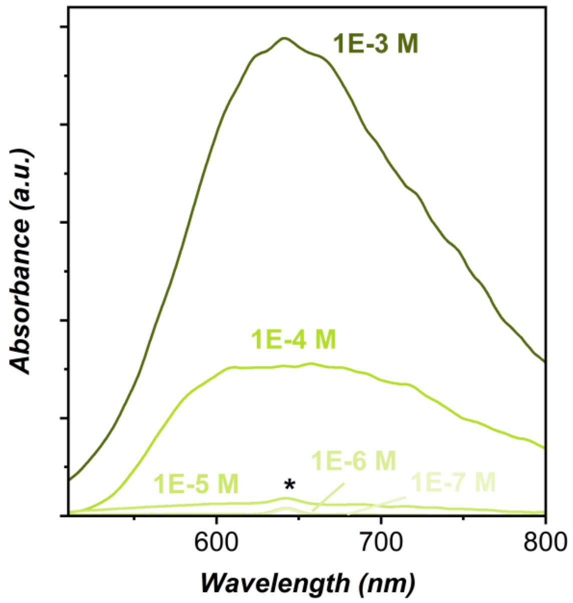

Figure S60. Fluorescence spectra of **pseudo-*m*-NB** from excitation at 430 nm in CH_3CN at various concentrations. (* CH_3CN Raman peak)

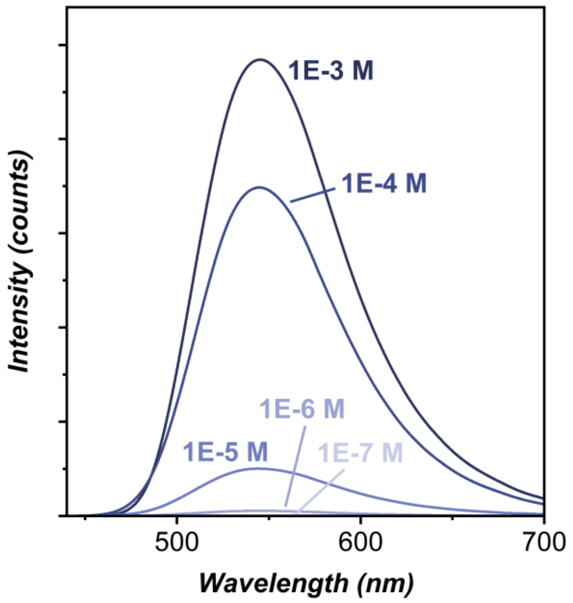

Figure S61. Fluorescence spectra of **pseudo-*p*-NB** from excitation at 420 nm in CH_3CN at various concentrations. (* CH_3CN Raman peak)

Section S13 Computational Modelling of Electronic Excitations

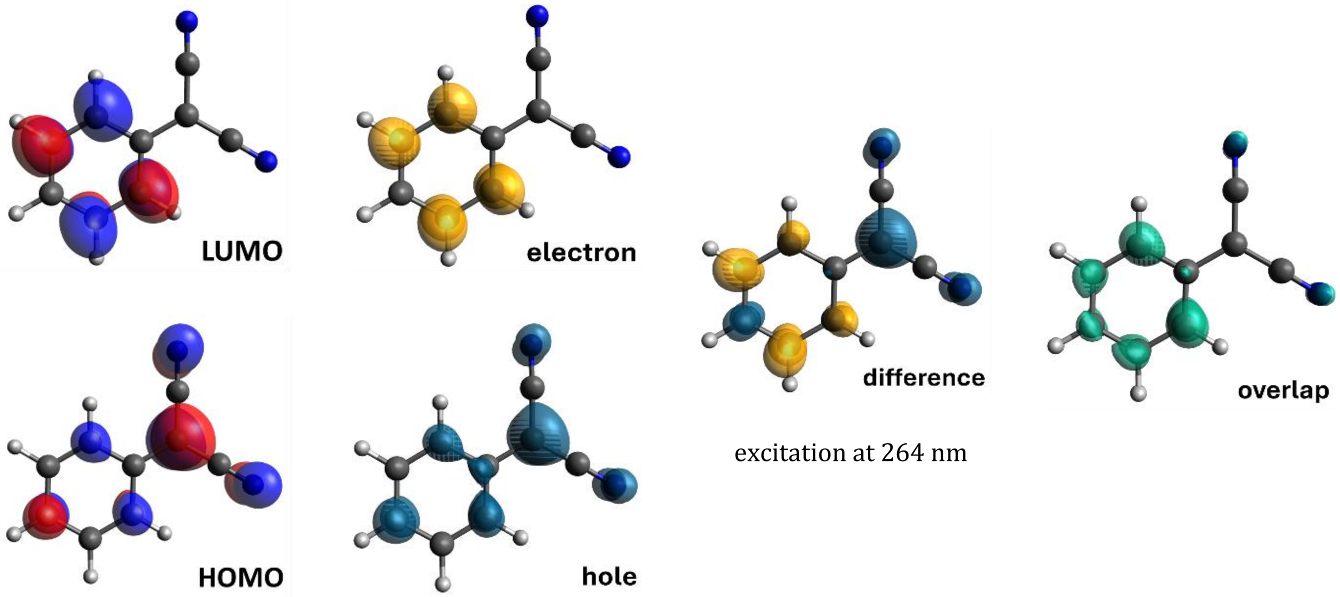

Figure S62. Kohn-Sham orbitals, hole & electron, charge density difference & overlap functions of HOMO-LUMO transition at 264 nm for phenylmalonide anion with isovalue 0.05.

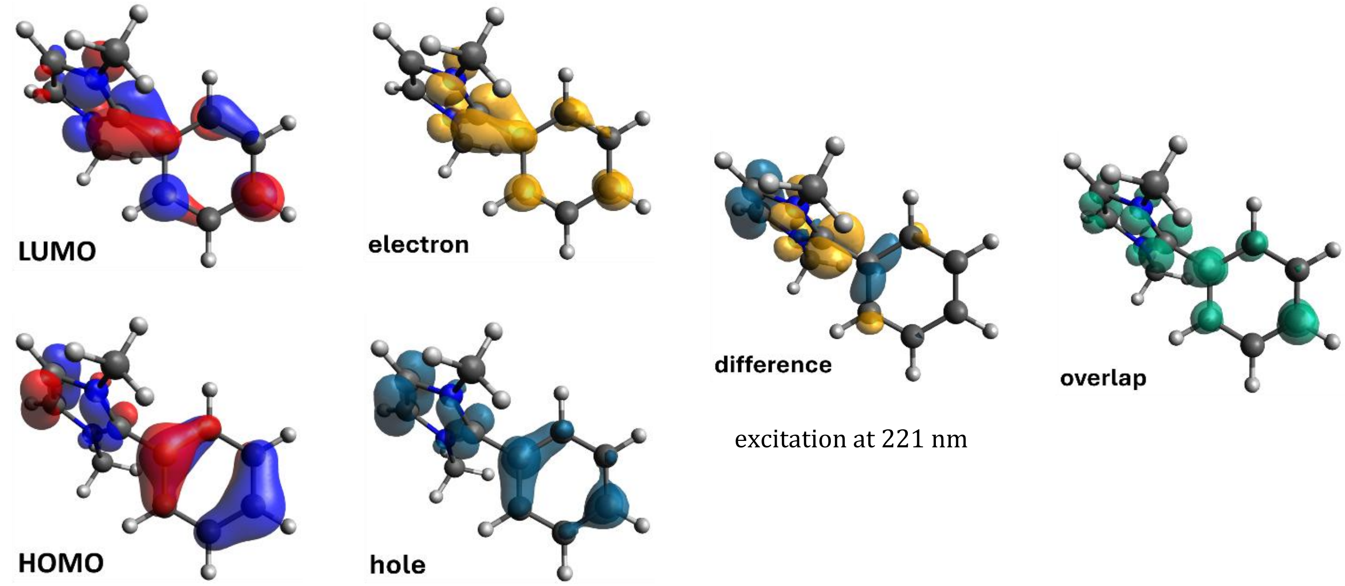

Figure S63. Kohn-Sham orbitals, hole & electron, charge density difference & overlap functions of HOMO-LUMO transition at 221 nm for phenylimidazolium cation with isovalue 0.05.

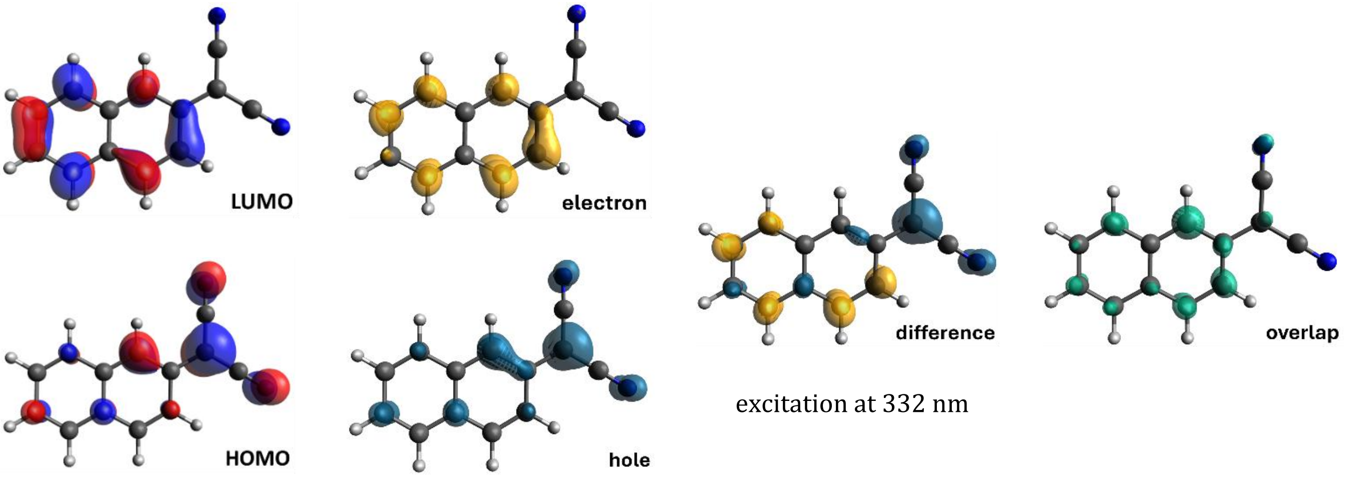

Figure S64. Kohn-Sham orbitals, hole & electron, charge density difference & overlap functions of HOMO-LUMO transition at 332 nm for naphthylmalonide anion with isovalue 0.05.

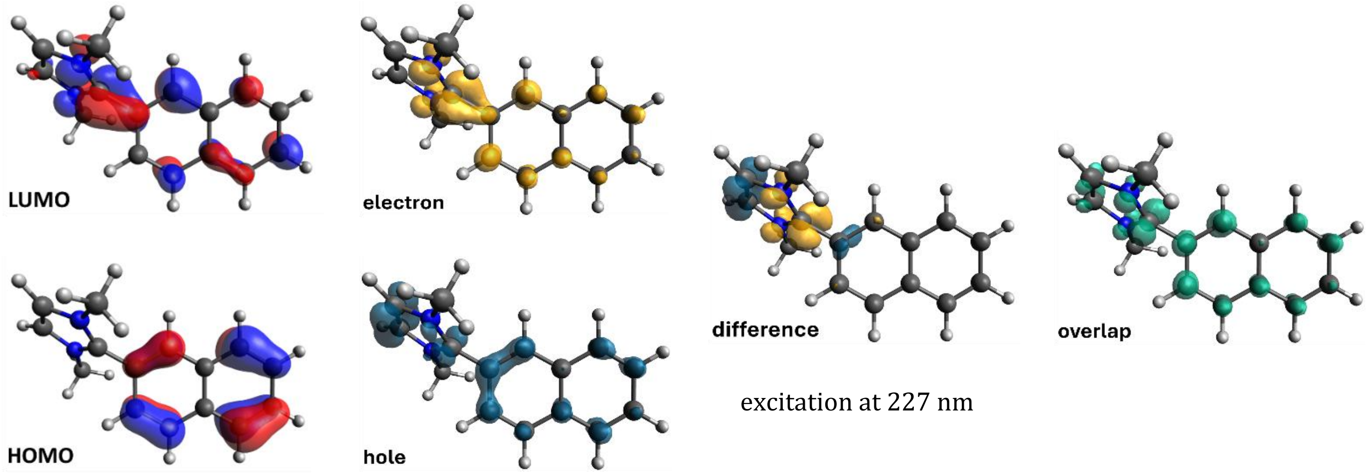

Figure S65. Kohn-Sham orbitals, hole & electron, charge density difference & overlap functions of HOMO-LUMO transition at 227 nm for naphthylimidazolium cation with isovalue 0.05.

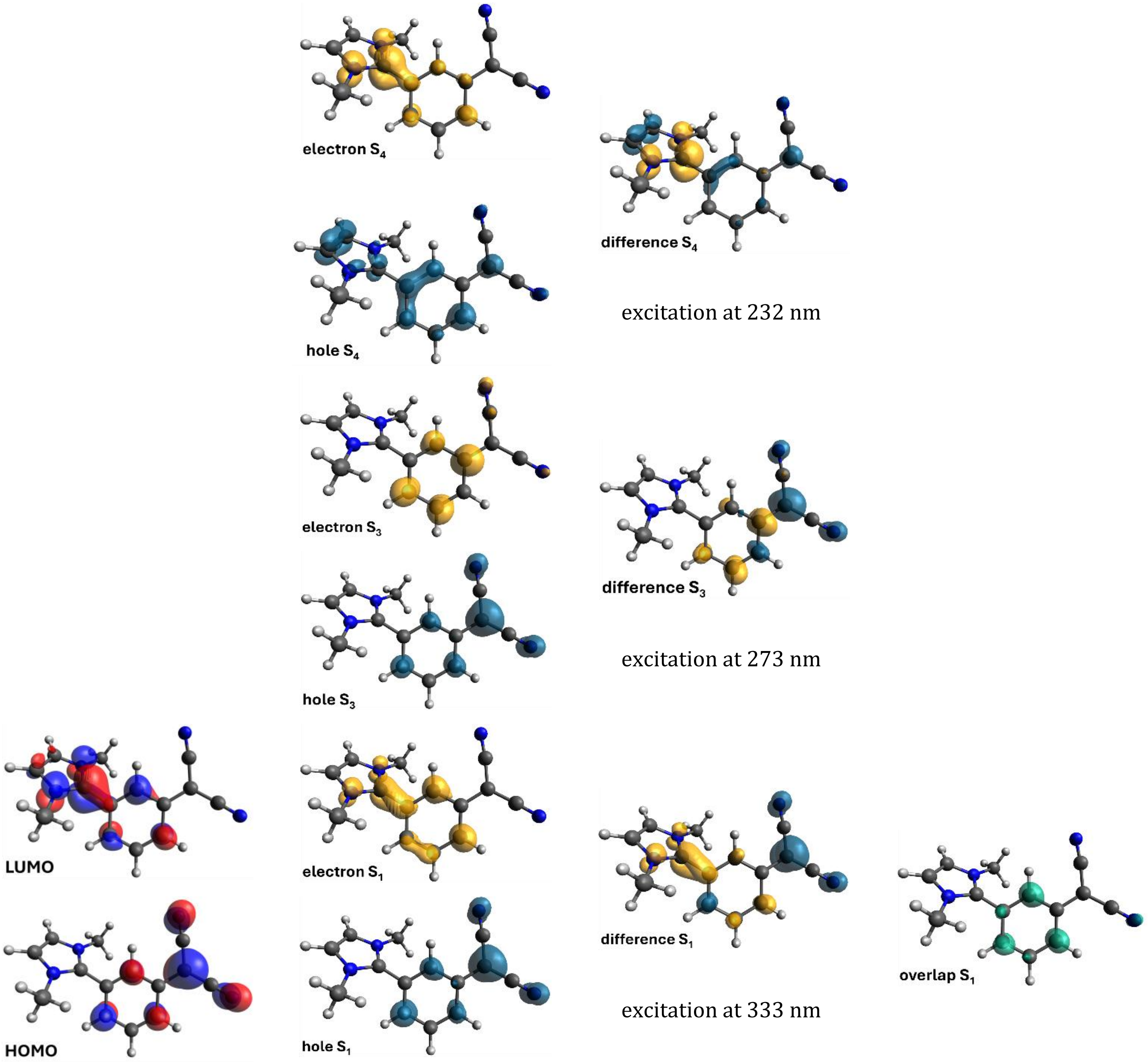

Figure S66. Kohn-Sham frontier orbitals, hole & electron, charge density difference & overlap functions for significant excitations of ***m*-PB** with isovalue 0.05.

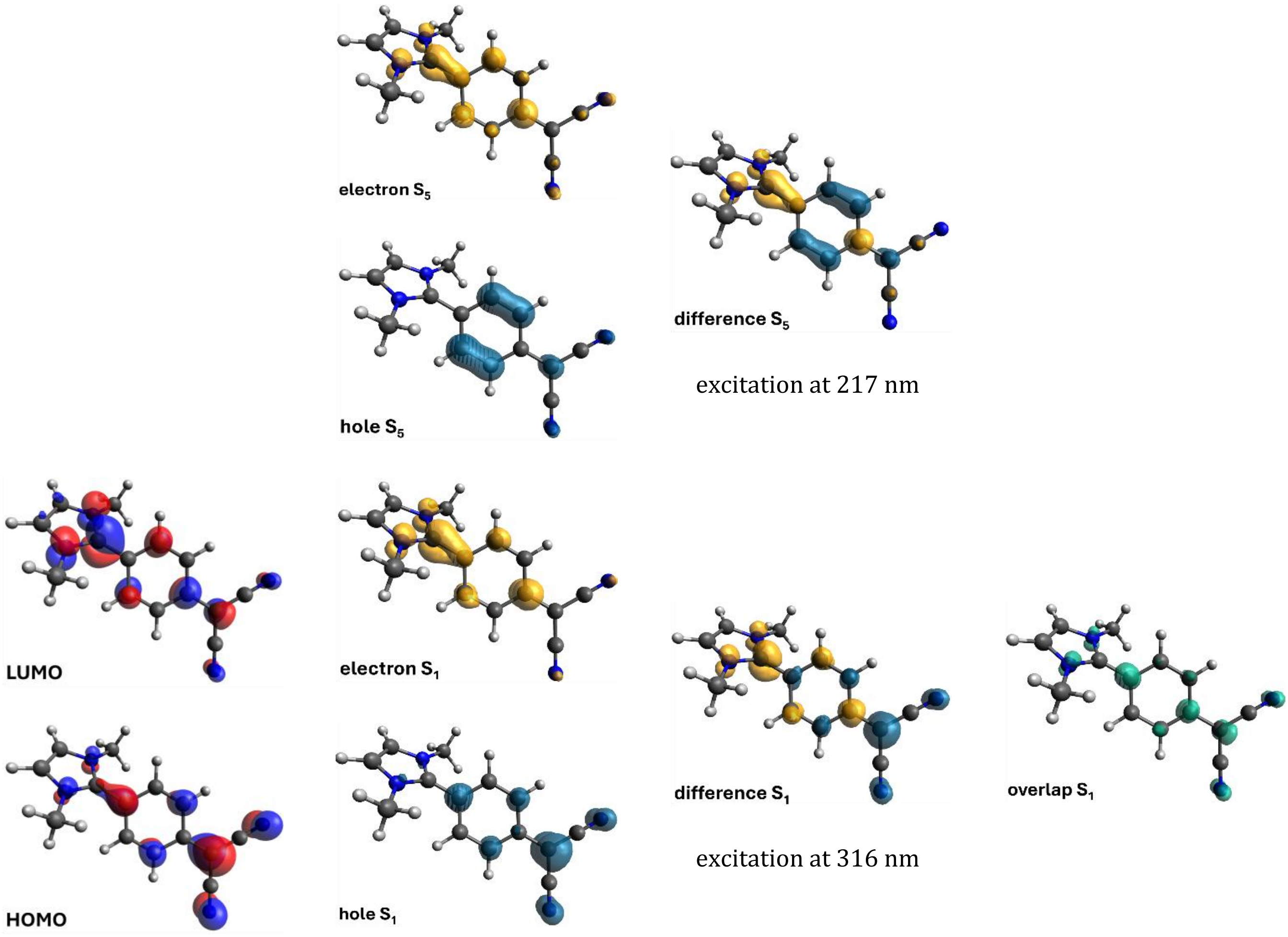

Figure S67. Kohn-Sham frontier orbitals, hole & electron, charge density difference & overlap functions for significant excitations of ***p*-PB** with isovalue 0.05.

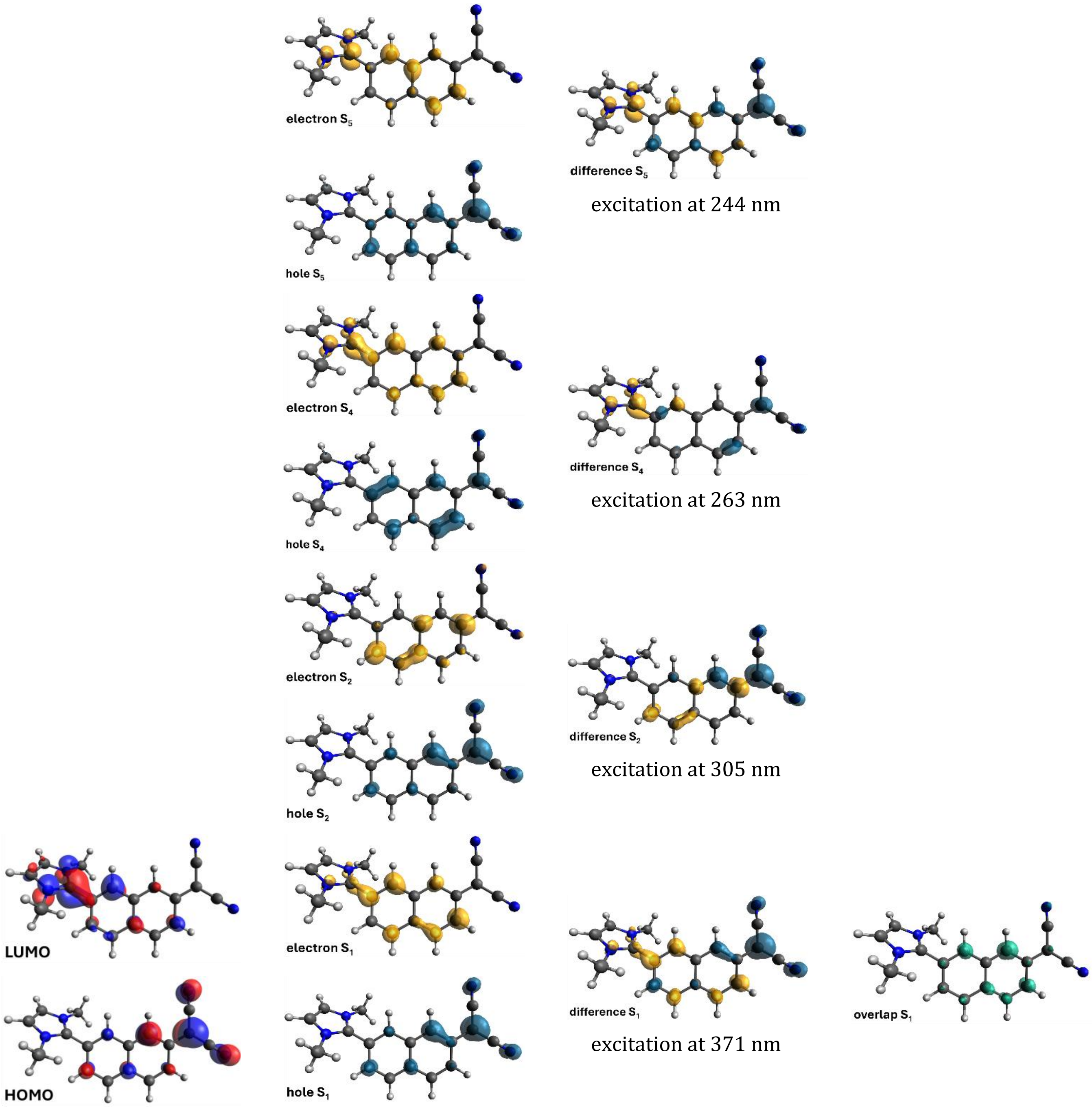

Figure S68. Kohn-Sham frontier orbitals, hole & electron, charge density difference & overlap functions for significant excitations of **pseudo-*m*-NB** with isovalue 0.05.

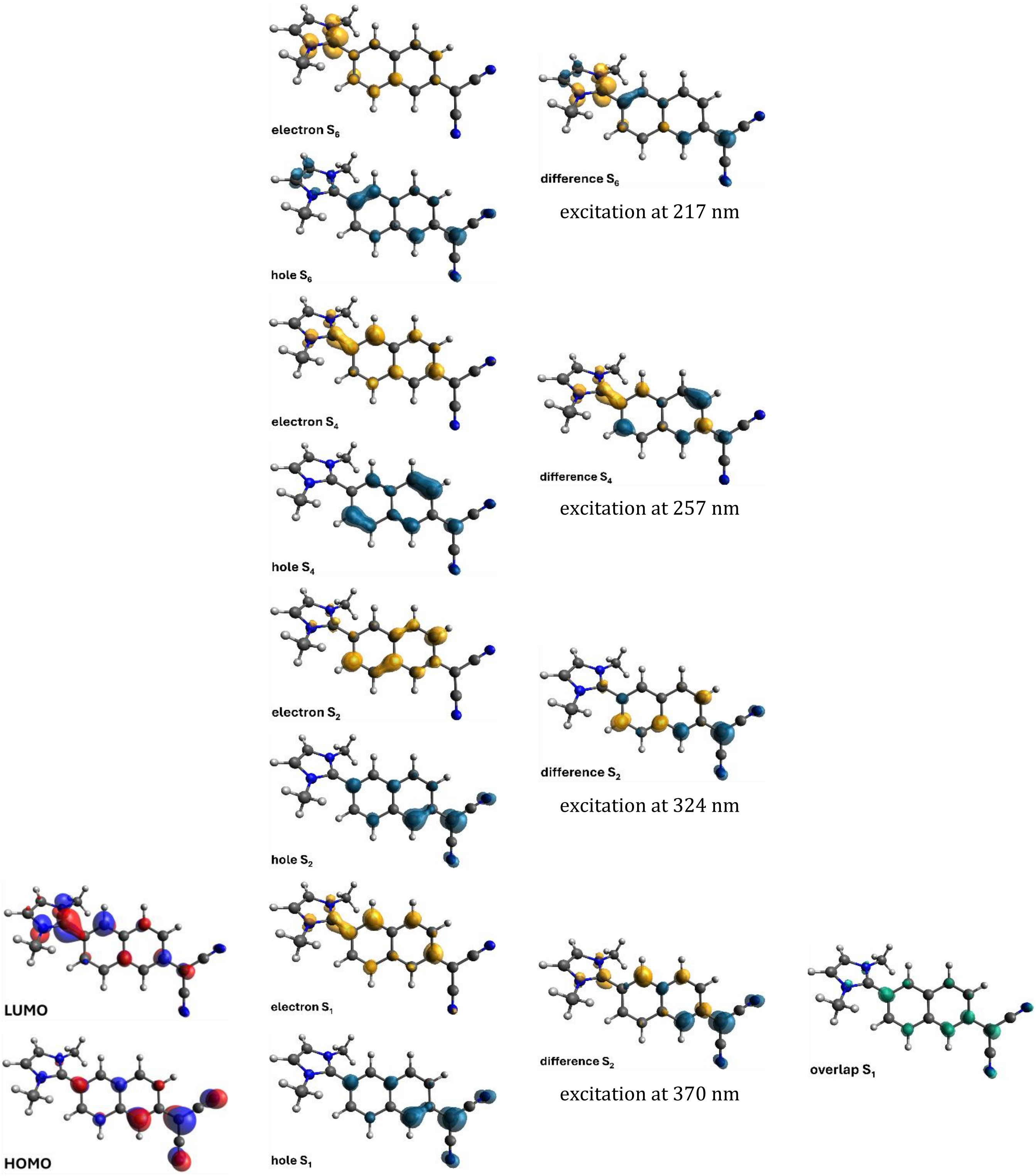

Figure S69. Kohn-Sham frontier orbitals, hole & electron, charge density difference & overlap functions for significant excitations of **pseudo-*p*-NB** with isovalue 0.05.

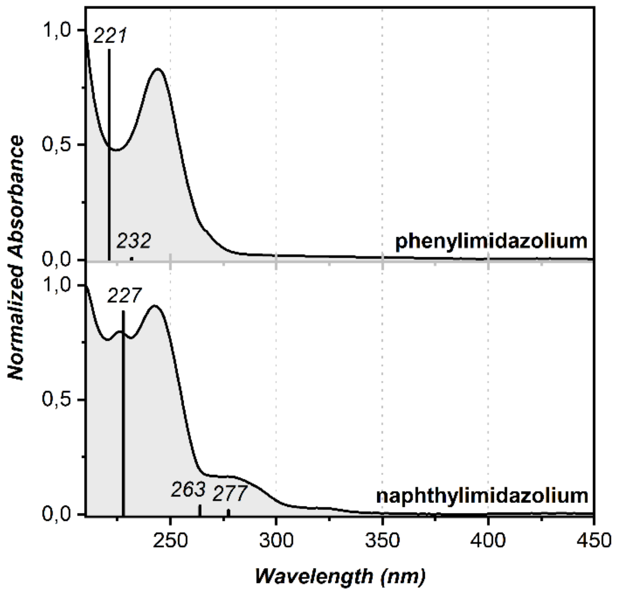

Figure S70. Calculated (CAM-B3LYP/def2-TZVP) and experimental absorbance spectra of phenylimidazolium and naphthylimidazolium cations in CH_3CN. Black bars indicate positions of vertical excitations from TD-DFT.

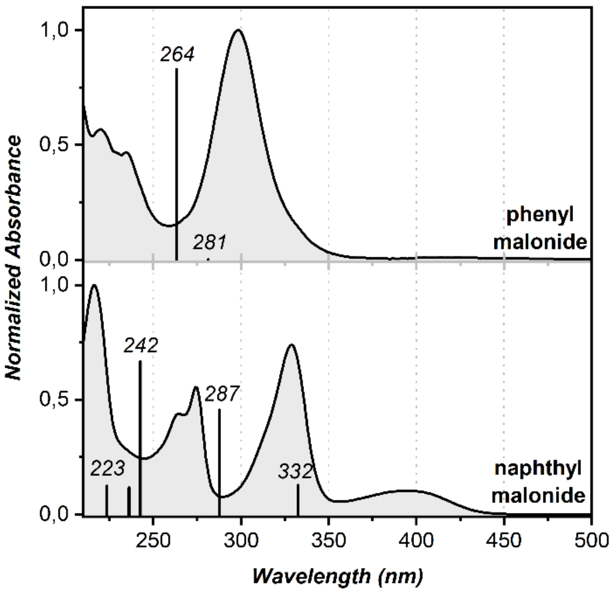

Figure S71. Calculated (CAM-B3LYP/def2-TZVP) and experimental absorbance spectra of phenylmalonide and naphthylmalonide anions in CH_3CN. Black bars indicate positions of vertical excitations from TD-DFT.

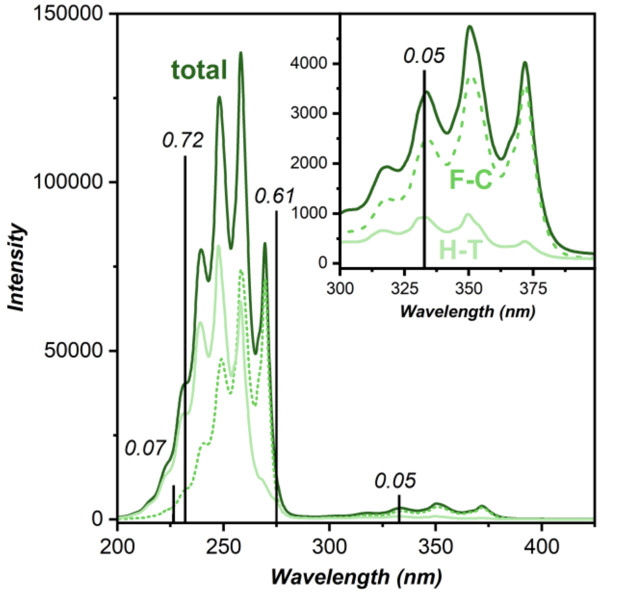

Figure S72. Calculated absorption spectra for ***m*-PB** (CAM-B3LYP/def2-TZVP) in CH_3CN. Lime lines show Frank-Condon and Herzberg-Teller components from excited-state dynamics calculations. Black bars show oscillator strengths of excitations from TD-DFT.

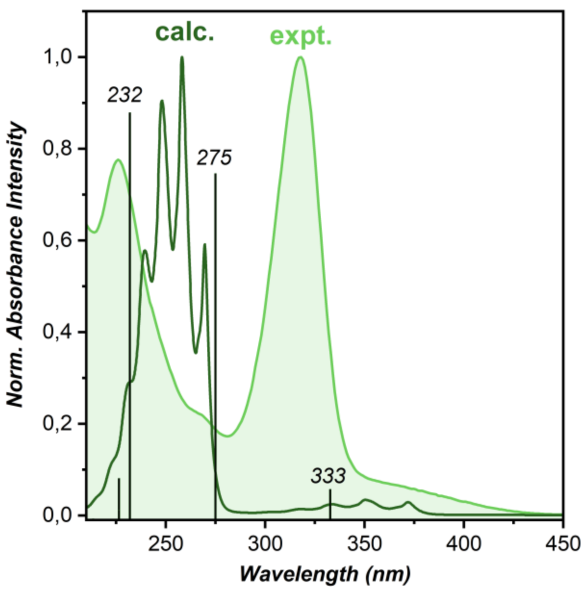

Figure S73. Calculated (excited state dynamics, CAM-B3LYP/def2-TZVP) and experimental absorbance spectra of ***m*-PB** in CH_3CN. Black bars indicate positions of vertical excitations from TD-DFT.

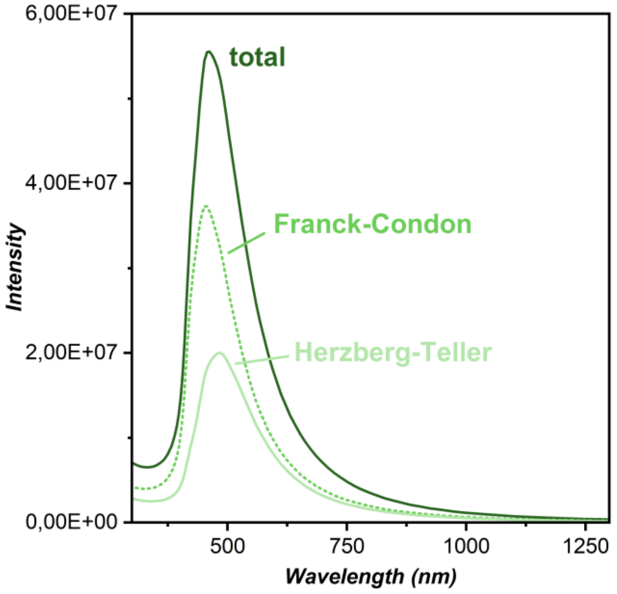

Figure S74. Calculated fluorescence spectra for ***m*-PB** at the CAM-B3LYP/def2-TZVP level in CH_3CN. Green lines show results from excited-state dynamics calculations, decomposed into Frank-Condon and Herzberg-Teller components.

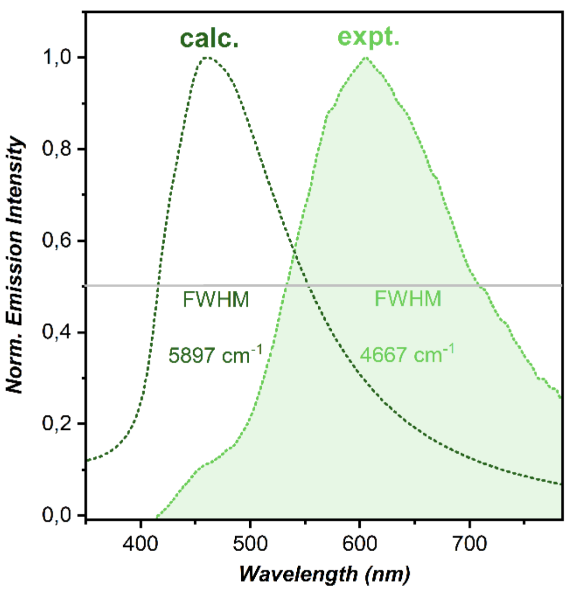

Figure S75. Calculated (excited state dynamics, CAM-B3LYP/def2-TZVP) and experimental fluorescence spectra of ***m*-PB** in CH_3CN.

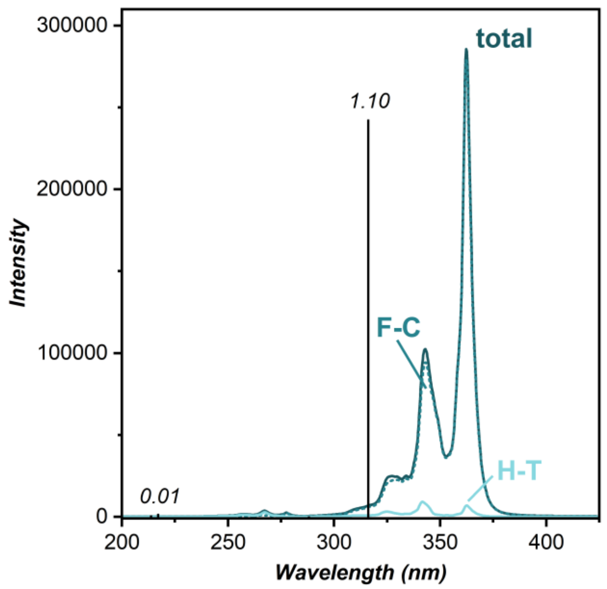

Figure S76. Calculated absorption spectra for ***p*-PB** (CAM-B3LYP/def2-TZVP) in CH_3CN. Teal lines show Frank-Condon and Herzberg-Teller components from excited-state dynamics calculations. Black bars show oscillator strengths of excitations from TD-DFT.

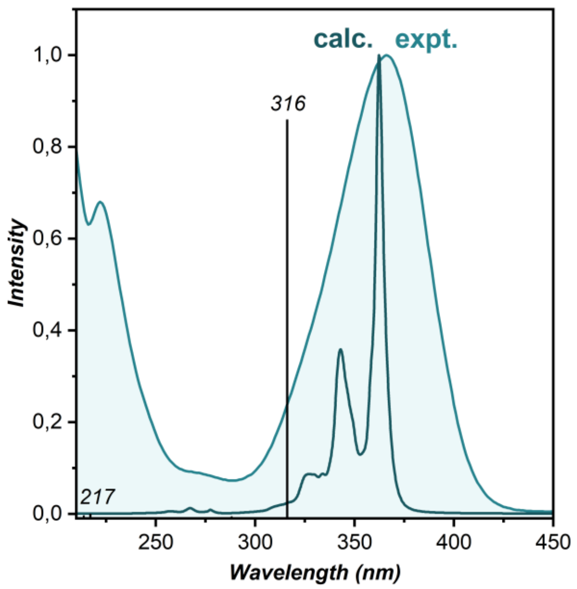

Figure S77. Calculated (excited state dynamics, CAM-B3LYP/def2-TZVP) and experimental absorbance spectra of ***p*-PB** in CH_3CN. Black bars indicate positions of vertical excitations from TD-DFT.

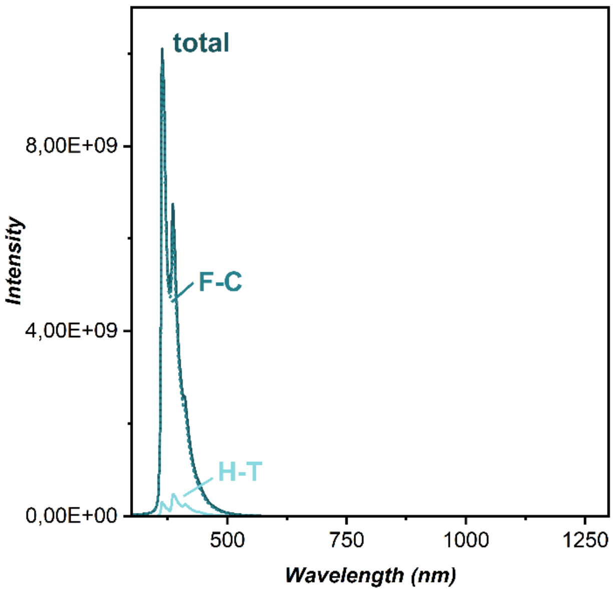

Figure S78. Calculated fluorescence spectra for ***p*-PB** at the CAM-B3LYP/def2-TZVP level in CH_3CN. Teal lines show results from excited-state dynamics calculations, decomposed into Frank-Condon and Herzberg-Teller components.

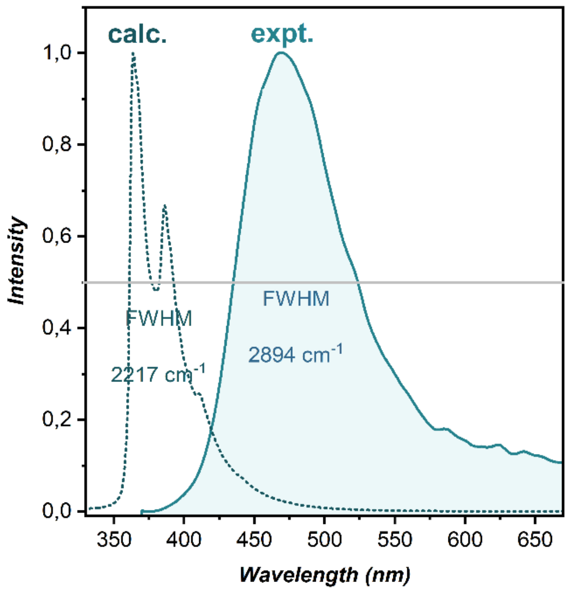

Figure S79. Calculated (excited state dynamics, CAM-B3LYP/def2-TZVP) and experimental fluorescence spectra of ***p*-PB** in CH_3CN.

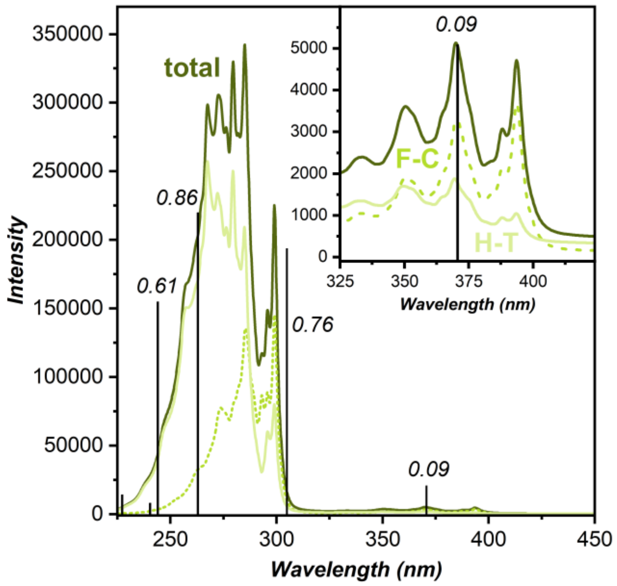

Figure S80. Calculated absorption spectra for **pseudo-*m*-NB** (CAM-B3LYP/def2-TZVP) in CH_3CN. Lime lines show Frank-Condon and Herzberg-Teller components from excited-state dynamics calculations. Black bars show oscillator strengths of excitations from TD-DFT.

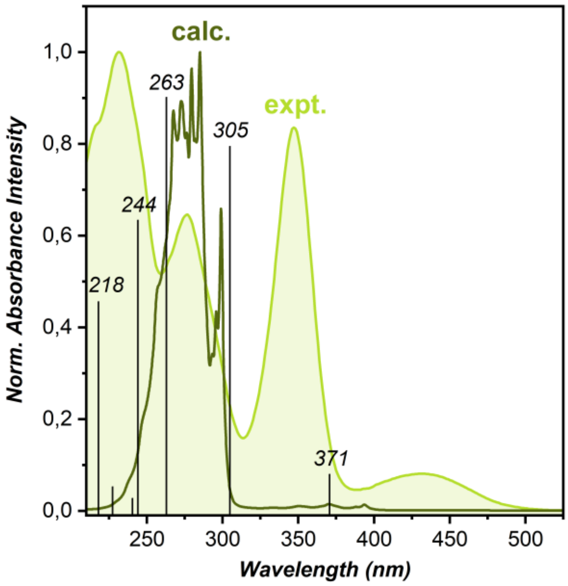

Figure S81. Calculated (excited state dynamics, CAM-B3LYP/def2-TZVP) and experimental absorbance spectra of **pseudo-*m*-NB** in CH_3CN. Black bars indicate positions of vertical excitations from TD-DFT.

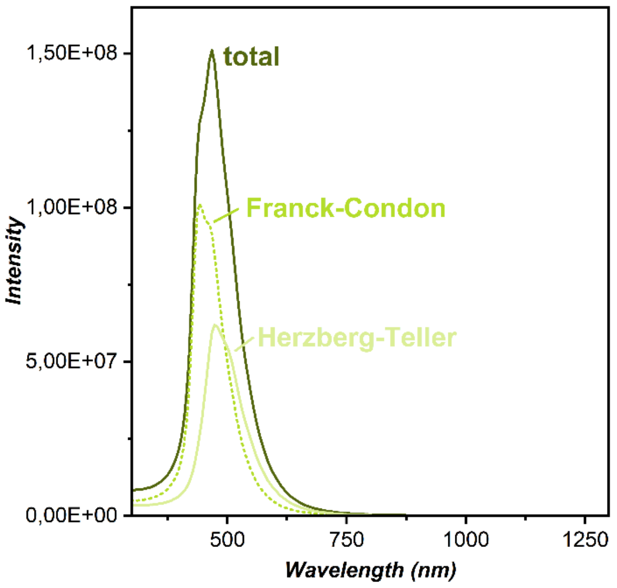

Figure S82. Calculated fluorescence spectra for **pseudo-*m*-NB** at the CAM-B3LYP/def2-TZVP level in CH_3CN. Lime lines show results from excited-state dynamics calculations, decomposed into Frank-Condon and Herzberg-Teller components.

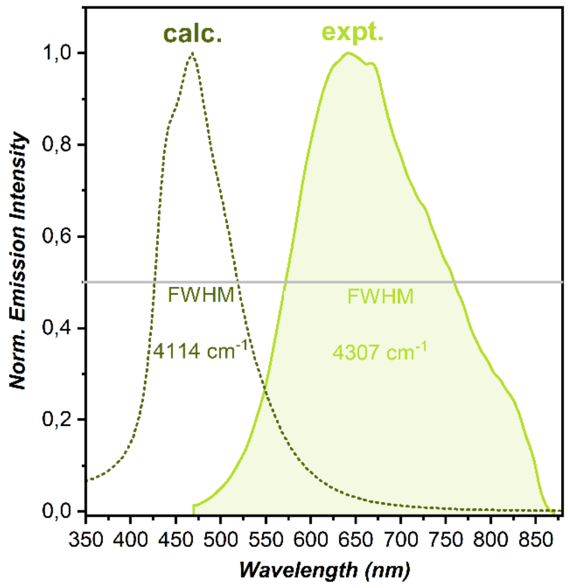

Figure S83. Calculated (excited state dynamics, CAM-B3LYP/def2-TZVP) and experimental fluorescence spectra of **pseudo-*m*-NB** in CH_3CN.

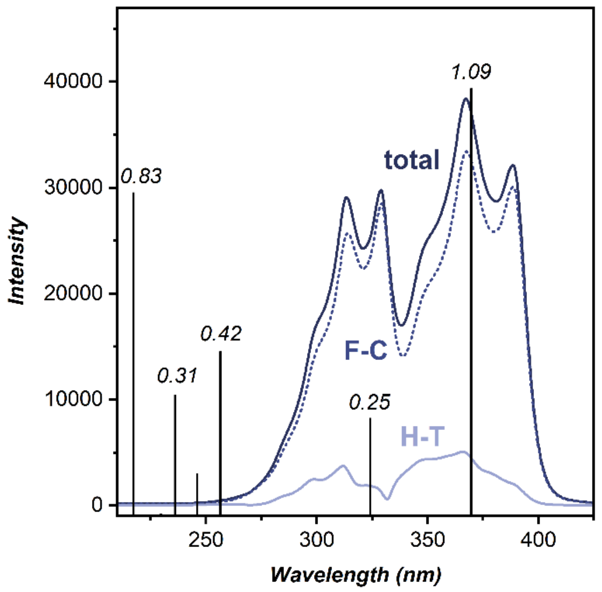

Figure S84. Calculated absorption spectra for **pseudo-*p*-NB** (CAM-B3LYP/def2-TZVP) in CH_3CN. Navy lines show Frank-Condon and Herzberg-Teller components from excited-state dynamics calculations. Black bars show oscillator strengths of excitations from TD-DFT.

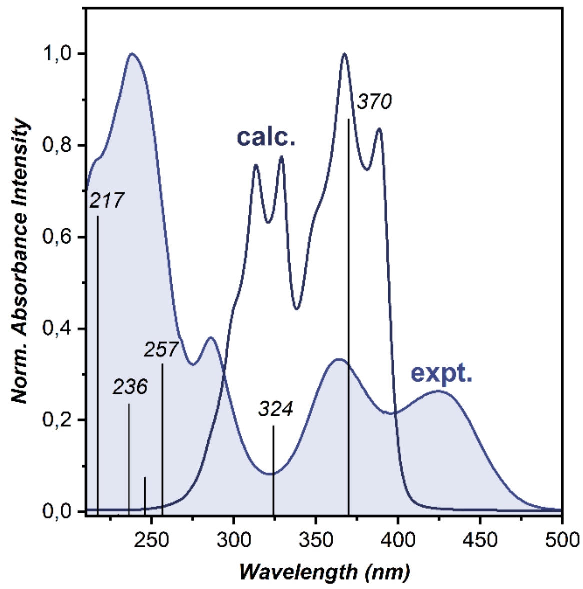

Figure S85. Calculated (excited state dynamics, CAM-B3LYP/def2-TZVP) and experimental absorbance spectra of **pseudo-*p*-NB** in CH_3CN. Black bars indicate positions of vertical excitations from TD-DFT.

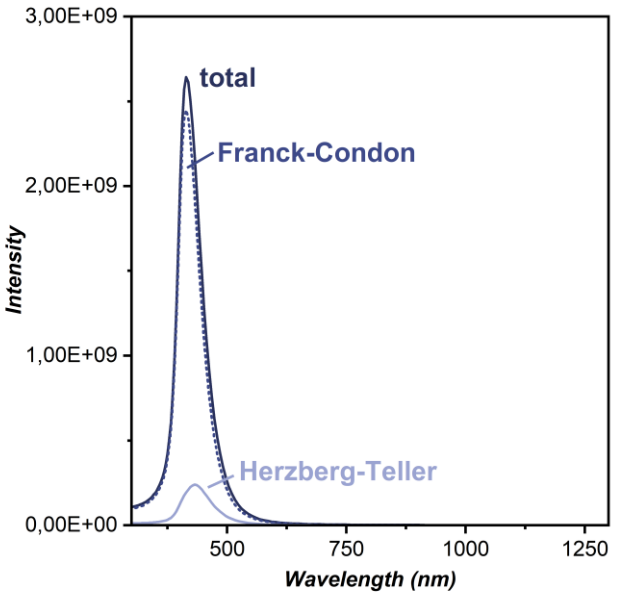

Figure S86. Calculated fluorescence spectra for **pseudo-*p*-NB** at the CAM-B3LYP/def2-TZVP level in CH_3CN. Navy lines show results from excited-state dynamics calculations, decomposed into Frank-Condon and Herzberg-Teller components.

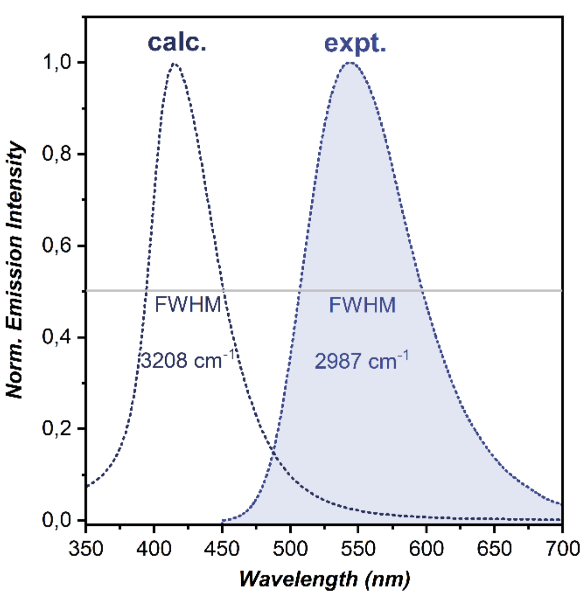

Figure S87. Calculated (excited state dynamics, CAM-B3LYP/def2-TZVP) and experimental fluorescence spectra of **pseudo-*p*-NB** in CH_3CN.

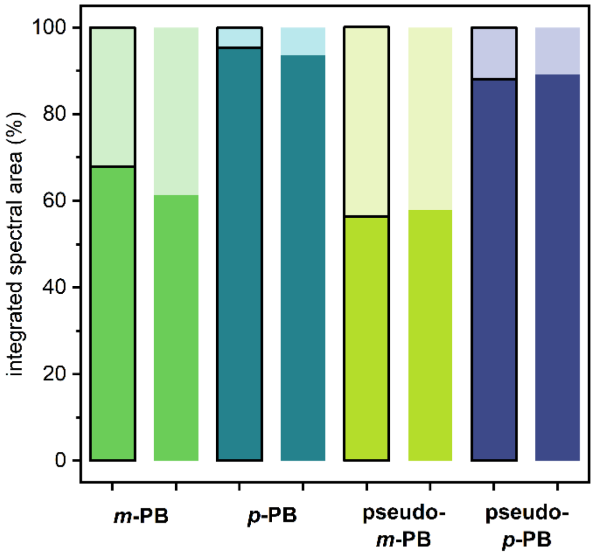

Figure S88 Integrated Franck-Condon (dark, below) and Herzberg-Teller (light, above) contributions to calculated lowest-energy absorption (black outline) and fluorescence (no outline).

Table S17. Predicted Franck-Condon and Herzberg-Teller components to lowest-energy absorption band, and fluorescence spectra.

compound	Spectral type	Franck-Condon (%)	Herzberg-Teller (%)
***m*-PB**	absorption	68.0	32.0
	fluorescence	61.3	38.7
***p*-PB**	absorption	95.3	4.7
	fluorescence	93.7	6.3
**pseudo-*m*-NB**	absorption	56.4	43.7
	fluorescence	57.9	42.1
**pseudo-*p*-NB**	absorption	88.0	12.0
	fluorescence	89.1	10.9

Table S18. Comparison of optical, electrochemical and theoretical HOMO-LUMO energy gaps in CH_3CN and differences between *meta*- and *para*-analogues.

compound	E_{gap}^{opt} (eV)[a]	ΔE_{m-p}^{opt} (eV)	E_{gap}^{elec} (eV)[b]	ΔE_{m-p}^{elec} (eV)	E_{gap}^{theo} (eV)[c]	ΔE_{m-p}^{theo} (eV)
***m*-PB**	2.961		2.558		3.725	
***p*-PB**	3.004	0.043	2.782	0.224	3.922	0.197
**pseudo-*m*-NB**	2.559		2.414		3.345	
**pseudo-*p*-NB**	2.583	0.024	2.616	0.202	3.354	0.009

[a] determined as the 0-0 transition from the overlap of normalized reduced UV-vis and fluorescence spectra.[67]

[b] determined from peak potentials of oxidation and reduction in DPV voltammograms.

[c] determined from vertical excitation energy of TD-DFT output.

The steps followed to determine the 0-0 transition are taken from literature.[67] Briefly:

1. Each datapoint of the wavelength ranges were converted from nanometers, to meters, then into photon energy using the formula $E = \frac{hc}{\lambda}$.
2. For absorbance, the photon energy was divided at each data point by the absorbance values. For fluorescence, the photon energy was cubed then divided at each data point by the fluorescence values.
3. The resulting data points were normalized and plotted on the same set of axes to determine the intersection (*ie.* the 0-0 transition).

Table S19. Various calculated properties of the lowest-energy excitation.

	***m*-PB**	***p*-PB**	**pseudo-*m*-NB**	**pseudo-*p*-NB**
**Oscillator Strength *f***	0.054	1.097	0.085	1.094
**$\Delta \mu_{molecule}$ from $S_0 \rightarrow S_1$ (D)**	-12.5	-9.3	-13.9	-11.0
**Trans. Elec. Dipole Moment μ_{elec}**	0.15	0.65	0.20	0.70
**Stokes Shift (cm^{-1})**	5284	886	4013	1552
**S_r index**	0.60	0.70	0.67	0.74
**D index (Å)**	2.34	2.16	2.47	2.21
***t* index (Å)**	0.54	0.09	0.47	0.17

$\Delta \mu_{molecule}$ – changes in molecular dipole from geometry-optimised S_0 and S_1 states; S_r – orbital overlap between the hole & electron, value from 0-1; D – distance between centroid of hole and electron in Angstrom (also called CT length); *t* – degree of separation between hole and electron in Angstrom (smaller numbers correspond to less H+/E- separation, and *vice versa*).

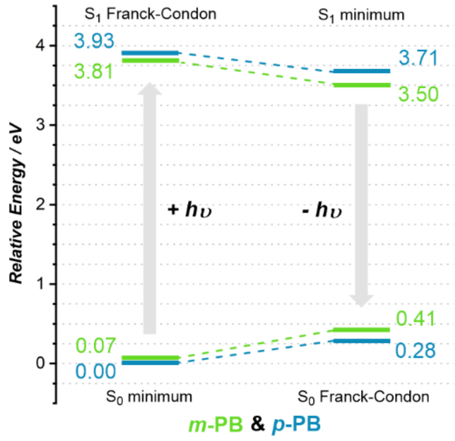

Figure S89. Calculated energies of ***m*-PB** and ***p*-PB** before and after excitation, and before and after relaxation using the DRACO solvation scheme. (De)excitation is represented by the grey arrow while geometric relaxation by the dashed line.

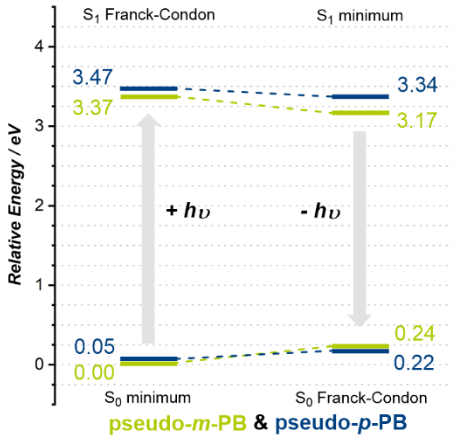

Figure S90. Calculated energies of **pseudo-*m*-PB** and **pseudo-*p*-PB** before and after excitation, and before and after relaxation using the DRACO solvation scheme. (De)excitation is represented by the grey arrow while geometric relaxation by the dashed line.

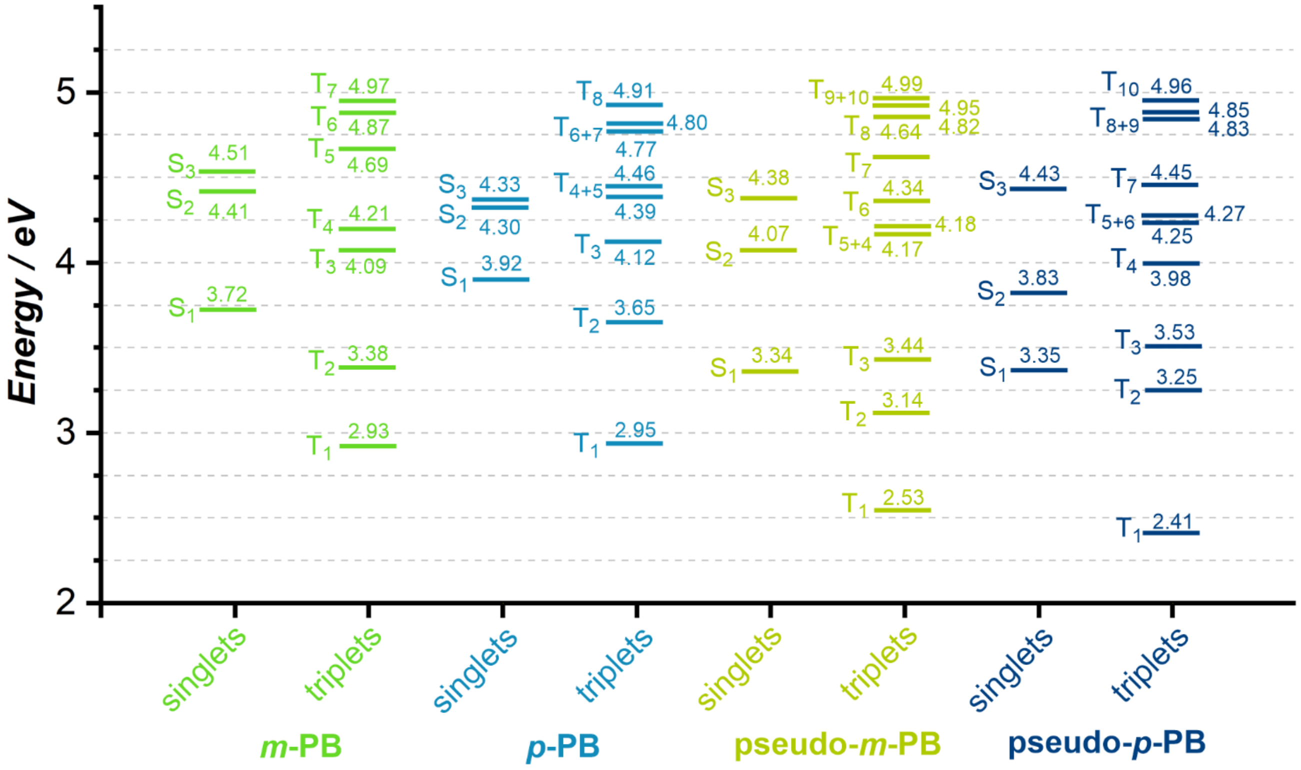

Figure S91. Calculated singlet (S_n) and triplet (T_n) states below 5 eV of betaines.

Table S20. Some calculated singlet-triplet spin-orbit coupling constants* of betaines.

***m*-PB**			***p*-PB**			**pseudo-*m*-NB**			**pseudo-*p*-NB**		
**T_n**	**S_n**	**ζ (cm^{-1}) ***	**T_n**	**S_n**	**ζ (cm^{-1}) ***	**T_n**	**S_n**	**ζ (cm^{-1}) ***	**T_n**	**S_n**	**ζ (cm^{-1}) ***
1	0	0,41	1	0	0,68	1	0	0,14	1	0	0,42
1	1	0,19	1	1	0,24	1	1	0,10	1	1	0,08
1	2	2,11	1	2	1,14	1	2	0,12	1	2	0,17
1	3	0,22	1	3	5,16	1	3	3,13	1	3	4,50
2	0	0,34	2	0	0,01	2	0	0,24	2	0	0,47
2	1	0,28	2	1	0,48	2	1	0,24	2	1	0,13
2	2	4,93	2	2	0,19	2	2	0,07	2	2	0,06
2	3	0,12	2	3	0,78	2	3	3,71	2	3	0,95
3	0	1,13	3	0	19,32	3	0	0,42	3	0	0,21
3	1	0,21	3	1	0,48	3	1	0,08	3	1	0,06
3	2	1,51	3	2	0,62	3	2	0,06	3	2	0,05
3	3	0,25	3	3	0,13	3	3	2,39	3	3	2,20
4	0	18,91	4	0	1,31	4	0	3,12	4	0	1,07
4	1	0,46	4	1	0,41	4	1	0,33	4	1	0,10
4	2	0,13	4	2	1,12	4	2	0,90	4	2	0,24
4	3	6,66	4	3	2,61	4	3	1,88	4	3	3,64
5	0	1,09	5	0	0,56	5	0	18,31	5	0	18,13
5	1	0,37	5	1	0,29	5	1	0,42	5	1	0,26
5	2	1,77	5	2	0,34	5	2	5,57	5	2	1,57
5	3	0,45	5	3	0,08	5	3	0,28	5	3	0,08
6	0	0,43	6	0	0,12	6	0	1,04	6	0	0,39
6	1	0,34	6	1	0,11	6	1	0,17	6	1	0,08
6	2	6,54	6	2	0,31	6	2	0,05	6	2	0,03
6	3	0,70	6	3	1,38	6	3	1,17	6	3	0,42
7	0	0,53	7	0	0,87	7	0	0,42	7	0	0,78
7	1	0,33	7	1	0,53	7	1	0,13	7	1	0,09
7	2	0,13	7	2	0,06	7	2	0,24	7	2	0,08
7	3	0,11	7	3	0,78	7	3	3,07	7	3	1,45

*The spin-orbit coupling constants ζ were calculated from the RMSD of the spin-orbit coupling matrix elements.

$$\zeta\ (RMSD, cm^{-1}) = \sqrt{X^2 + Y^2 + Z^2}$$

Table S21. Calculated electronic transitions for phenylimidazolium cation.

**Wavelength (nm)**	**Oscillator Strength**	**Transition Coefficient**	**MO Assignment**
231,7	0,01103	0,102030	HOMO-2→LUMO+1
		0,580214	HOMO-1→LUMO
		0,267442	HOMO→LUMO+1
221,2	0,58308	0,920507	HOMO→LUMO

Table S22. Calculated electronic transitions for phenylmalonide anion.

**Wavelength (nm)**	**Oscillator Strength**	**Transition Coefficient**	**MO Assignment**
291,5	2,42E-05	0,940206	HOMO→LUMO+2
281,3	0,009558	0,915360	HOMO→LUMO
263,5	0,681232	0,948851	HOMO→LUMO+1
219,9	6,89E-07	0,714619	HOMO→LUMO+6
		0,192246	HOMO→LUMO+7
218,6	0,00147	0,888073	HOMO→LUMO+3

Table S23. Calculated electronic transitions for naphthylimidazolium cation.

Wavelength (nm)	Oscillator Strength	Transition Coefficient	MO Assignment
276,8	0,057155	0.208033	HOMO-1→LUMO+1
		0.450519	HOMO→LUMO
		0.203117	HOMO→LUMO+1
263,4	0,095507	0.237928	HOMO-1→LUMO
		0.429503	HOMO→LUMO
		0.242840	HOMO→LUMO+1
227,2	1,698648	0.413246	HOMO-1→LUMO+1
		0.373878	HOMO→LUMO+2

Table S24. Calculated electronic transitions for naphthylmalonide anion.

Wavelength (nm)	Oscillator Strength	Transition Coefficient	MO Assignment
331,8	0,165678	0.883333	HOMO→LUMO
291,2	3,39E-05	0.900864	HOMO→LUMO+3
		0.109780	HOMO-1→LUMO
287,1	0,548557	0.783283	HOMO+→1
		0.586883	HOMO→LUMO+1
241,9	0,795215	0.260420	HOMO→LUMO+2
235,5	0,153025	0.162966	HOMO-2→LUMO
		0.118669	HOMO-1→LUMO
		0.239007	HOMO-1→LUMO+1
		0.333951	HOMO→LUMO+2
222,9	0,160795	0.179083	HOMO-2→LUMO
		0.291182	HOMO-1→LUMO+1
		0.329604	HOMO→LUMO+2
219,6	1,2E-06	0.327874	HOMO→LUMO+7
		0.523347	HOMO→LUMO+8
218,9	0,001665	0.905514	HOMO→LUMO+4

Table S25. Calculated electronic transitions for ***m*-PB**.

Wavelength (nm)	Oscillator Strength	Transition Coefficient	MO Assignment
332,8	0,05453	0.907516	HOMO→LUMO
281,1	3,8624E-5	0.205472	HOMO→LUMO+3
		0.675988	HOMO→LUMO+4
275	0,61331	0.903625	HOMO→LUMO+1
232	0,72103	0.615558	HOMO-1→LUMO
		0.219930	HOMO→LUMO+3
226,5	0,07377	0.212960	HOMO-1→LUMO
		0.235111	HOMO→LUMO+3
		0.120403	HOMO→LUMO+4
		0.127007	HOMO→LUMO+9
226,4	0,01737	0.250127	HOMO→LUMO+3
		0.155447	HOMO→LUMO+5
		0.127148	HOMO→LUMO+6
		0.143465	HOMO→LUMO+9
213,4	0,00218	0.259358	HOMO→LUMO+5
		0.338340	HOMO→LUMO+8
		0.164703	HOMO→LUMO+9

Table S26. Calculated electronic transitions for ***p*-PB**.

Wavelength (nm)	Oscillator Strength	Transition Coefficient	MO Assignment
316,1	1,09697	0.933276	HOMO→LUMO
288,1	0,00505	0.846227	HOMO→LUMO+1
286,1	3,28079E-4	0.863560	HOMO→LUMO+4
226,4	3,82855E-4	0.806348	HOMO→LUMO+3
217,1	0,01273	0.709193	HOMO-2→LUMO
213,8	0,00823	0.711721	HOMO→LUMO+9
211,3	0,00266	0.218527	HOMO→LUMO+5
		0.716890	HOMO→LUMO+7

Table S27. Calculated electronic transitions for **pseudo-*m*-NB**.

Wavelength (nm)	Oscillator Strength	Transition Coefficient	MO Assignment
370,7	0,08513	0.906277	HOMO→LUMO
304,9	0,76129	0.848838	HOMO→LUMO+1
283,2	8,6162E-5	0.899896	HOMO→LUMO+5
262,9	0,86304	0.701602	HOMO-1→LUMO
		0.104769	HOMO→LUMO+4
244,1	0,60956	0.136700	HOMO-1→LUMO
		0.476584	HOMO→LUMO+2
		0.192938	HOMO→LUMO+4
240,4	0,03579	0.220890	HOMO-2→LUMO
		0.527962	HOMO-1→LUMO+1
227,3	0,05981	0.236144	HOMO→LUMO+2
		0.113561	HOMO→LUMO+3
		0.488233	HOMO→LUMO+4
220,2	6,80627E-4	0.357283	HOMO→LUMO+4
		0.313321	HOMO→LUMO+3
218,1	0,4416	0.529326	HOMO-2→LUMO
		0.224756	HOMO-1→LUMO+1
214,9	0,00193	0.111806	HOMO→LUMO+7
		0.653088	HOMO→LUMO+8

Table S28. Calculated electronic transitions for **pseudo-*p*-NB**.

Wavelength (nm)	Oscillator Strength	Transition Coefficient	MO Assignment
369,7	1,09378	0.905925	HOMO→LUMO
324,2	0,25073	0.115430	HOMO-1→LUMO
		0.783600	HOMO→LUMO+1
279,6	4,6231E-5	0.879951	HOMO→LUMO+5
256,5	0,42096	0.119639	HOMO-2→LUMO
		0.653880	HOMO-1→LUMO
		0.120188	HOMO→LUMO+1
246,1	0,1082	0.467723	HOMO→LUMO+2
		0.273611	HOMO→LUMO+4
236,1	0,30929	0.184978	HOMO-2→LUMO
		0.217525	HOMO-1→LUMO+1
		0.151104	HOMO→LUMO+2
		0.279706	HOMO→LUMO+4
229,7	0,0067	0.435046	HOMO-2→LUMO
		0.189151	HOMO-1→LUMO+1
		0.190158	HOMO→LUMO+2
217,3	0,82705	0.342160	HOMO-1→LUMO+1
		0.261404	HOMO→LUMO+4
215,5	1,28159E-4	0.468910	HOMO→LUMO+11
		0.112046	HOMO→LUMO+13
		0.145434	HOMO→LUMO+14
212,8	0,00204	0.864855	HOMO→LUMO+8

Section S14 Acidochromism

The pK_a of the conjugate acids of the betaines in CH_3CN was determined by titrating a TFA/CH_3CN solution into a solution of the respective betaines in a quartz cuvette. The concentration of titrant was high enough such that less than 50 µL were added for full transformation. Following the titrations, the volume of titrant was plotted against the change in absorbance and fluorescence of the betaines. This was then fitted to a sigmoidal curve in Origin and the inflection point (*i.e.* the equivalence point) of that fitted sigmoidal curve was determined from its second derivative. The half-equivalence points were then taken (the point at which [conjugate base] = [acid] and $\log_{10}([A^-]/[HA]) = 0$) and inputted into the Henderson-Haselbalch equation, substituting pH for $-\log_{10}([TFA])$. The approximated acidities are comparable to Bordwell's phenylmalononitrile (pK_a = 4.2) and a related xylylene betaine[68] (pK_a = 3.4).

Table S29. pK_a values for conjugate acids of betaines in acetonitrile.

compound	pK_a
***m*-PB**	4.8
***p*-PB**	4.6
**pseudo-*m*-NB**	5.2
**pseudo-*p*-NB**	5.1

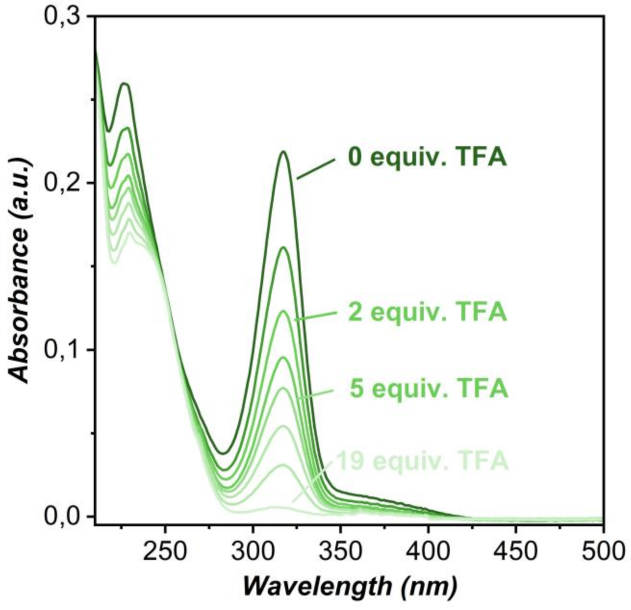

Figure S92. Absorption spectra of ***m*-PB** in CH_3CN as increasing amounts of TFA are titrated. Isosbestic points: 254 nm.

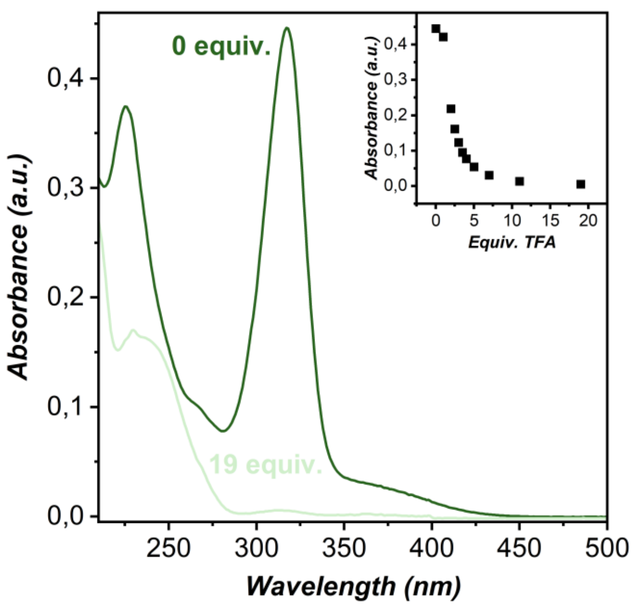

Figure S93. Absorption spectra of ***m*-PB** in CH_3CN before and after saturation with TFA, with inset of absorbance at 318 nm as a function of TFA molar equivalents.

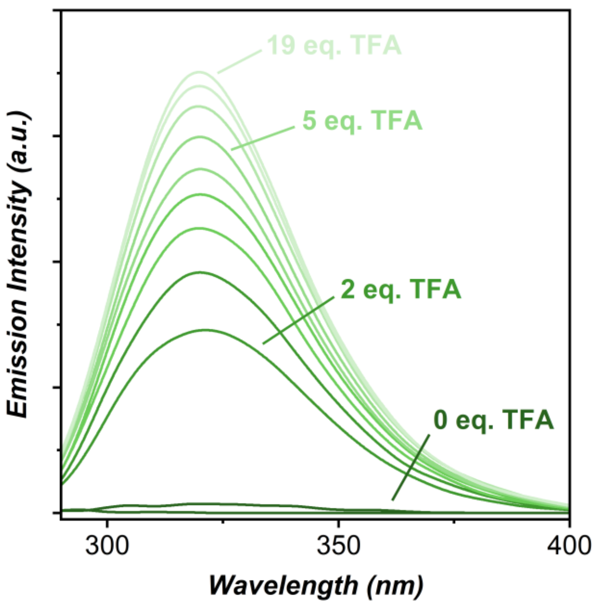

Figure S94. Fluorescence spectra of ***m*-PB** in CH_3CN excited at 270 nm as increasing amounts of TFA are titrated. (emission from 400-700 nm is dominated by 2nd order diffraction)

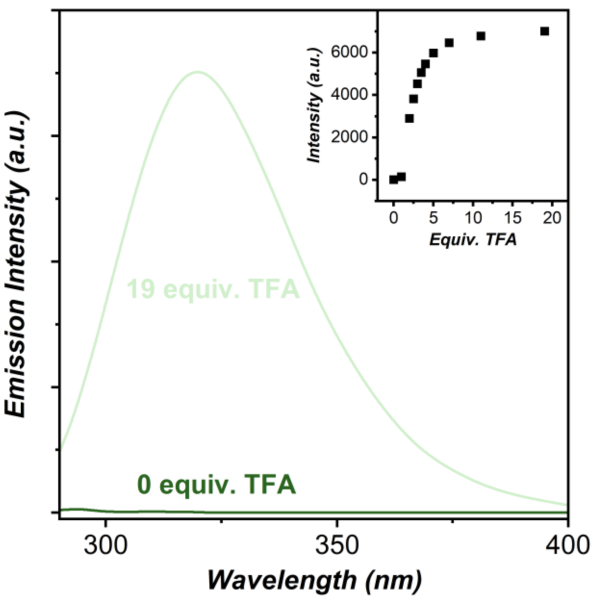

Figure S95. Fluorescence spectra of ***m*-PB** in CH_3CN excited at 270 nm before and after saturation with TFA, with inset of emission at 320 nm as a function of TFA molar equivalents.

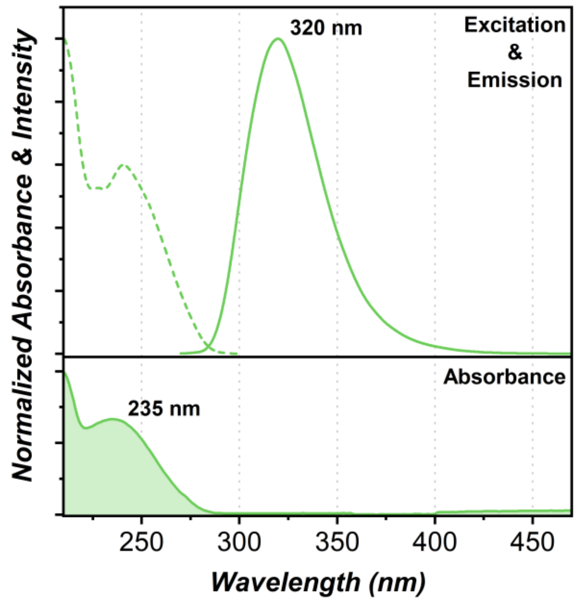

Figure S96. Excitation, emission (λ_{exc} 250 nm), and absorption spectra of ***m*-PB** in CH_3CN with sufficient TFA for full protonation.

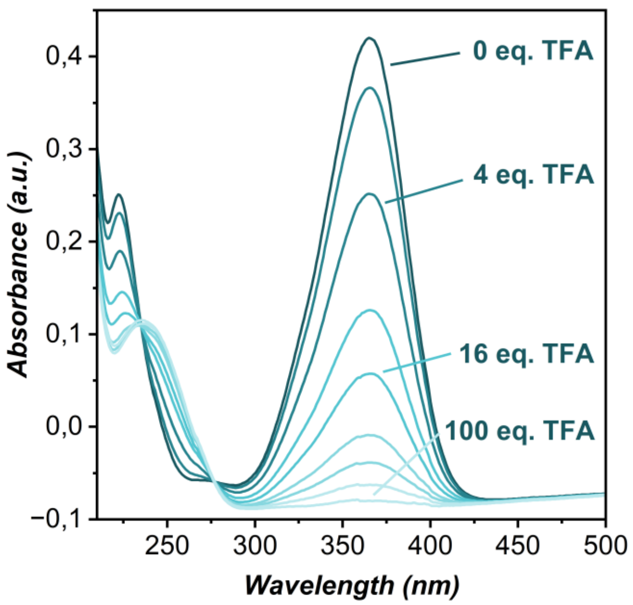

Figure S97. Absorption spectra of ***p*-PB** in CH_3CN as increasing amounts of TFA are titrated. Isosbestic points: 276 and 236 nm.

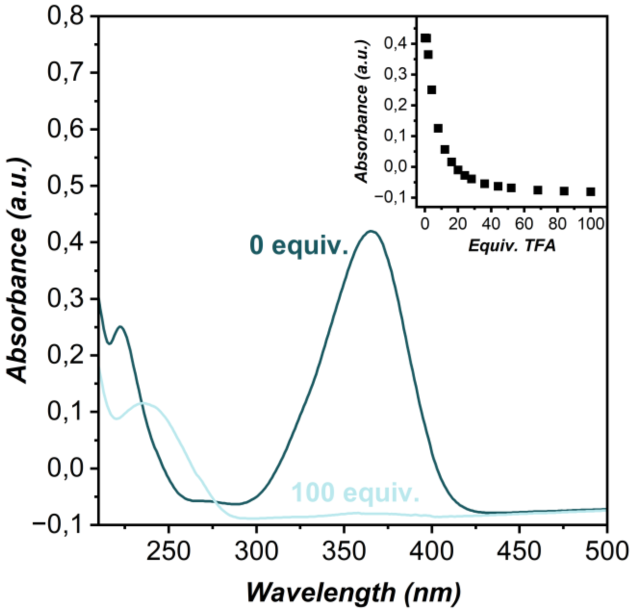

Figure S98. Absorption spectra of ***p*-PB** in CH_3CN before and after saturation with TFA, with inset of absorbance at 363 nm as a function of TFA molar equivalents.

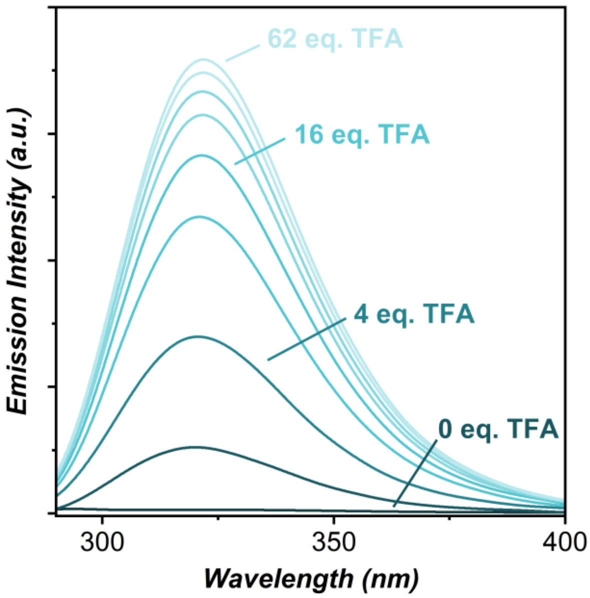

Figure S99. Fluorescence spectra of ***p*-PB** in CH_3CN excited at 270 nm as increasing amounts of TFA are titrated.

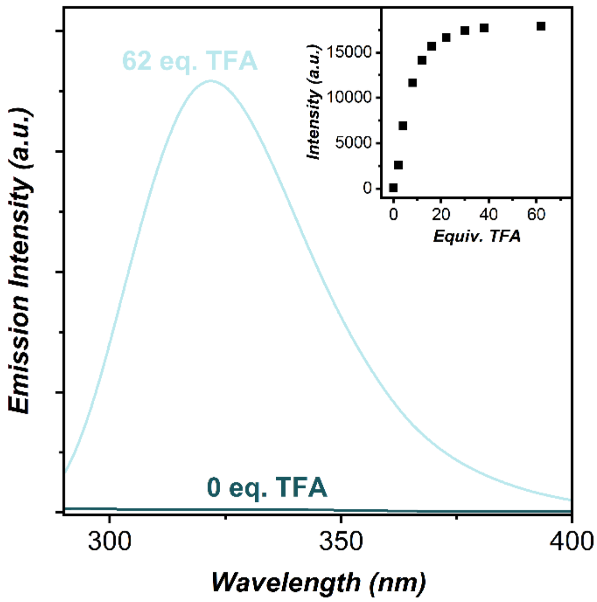

Figure S100. Fluorescence spectra of ***p*-PB** in CH_3CN excited at 270 nm before and after saturation with TFA, with inset of emission at 321 nm as a function of TFA molar equivalents.

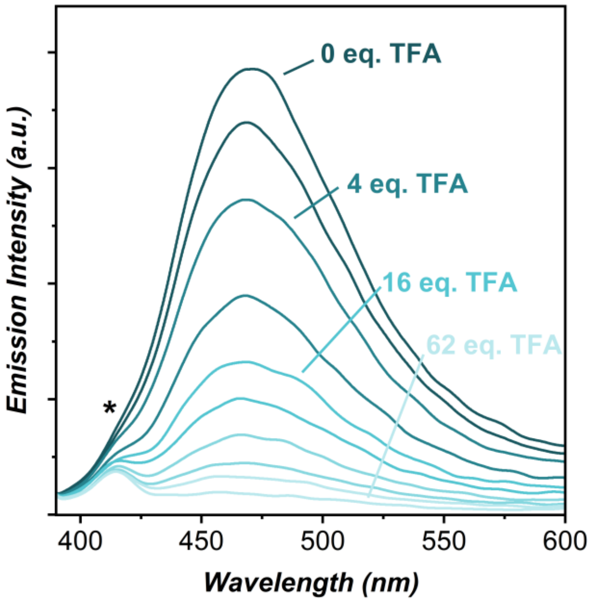

Figure S101. Fluorescence spectra of ***p*-PB** in CH_3CN excited at 370 nm as increasing amounts of TFA are titrated. (* CH_3CN Raman peak)

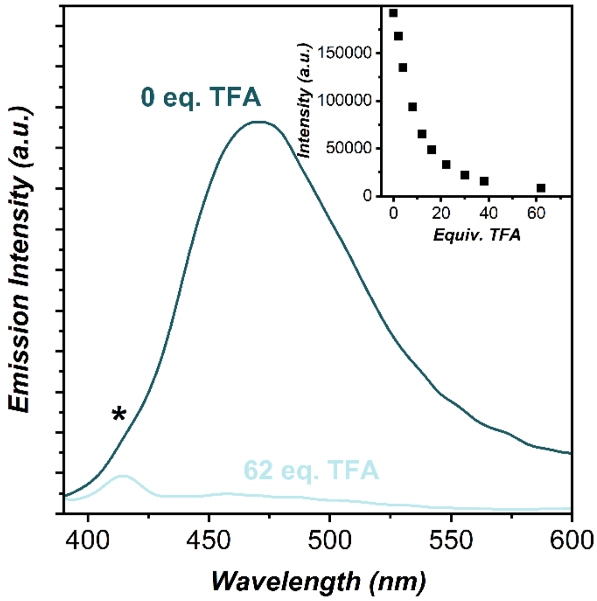

Figure S102. Fluorescence spectra of ***p*-PB** in CH_3CN excited at 370 nm before and after saturation with TFA, with inset of emission at 450 nm as a function of TFA molar equivalents. (* CH_3CN Raman peak)

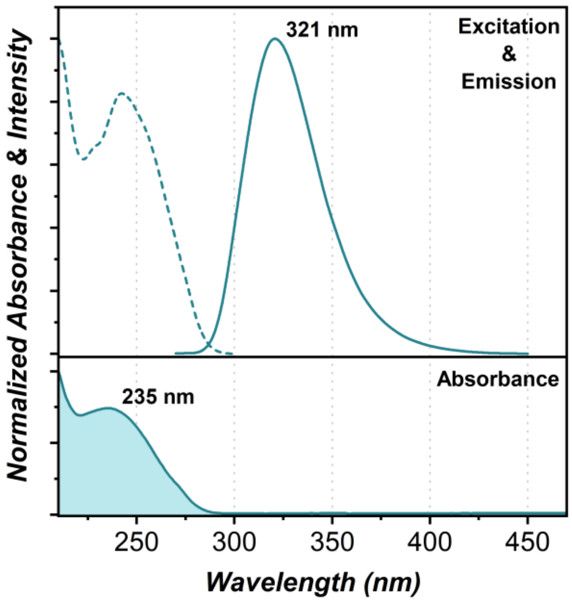

Figure S103. Excitation, emission (λ_{exc} 250 nm), and absorption spectra of ***p*-PB** in CH_3CN with sufficient TFA for full protonation.

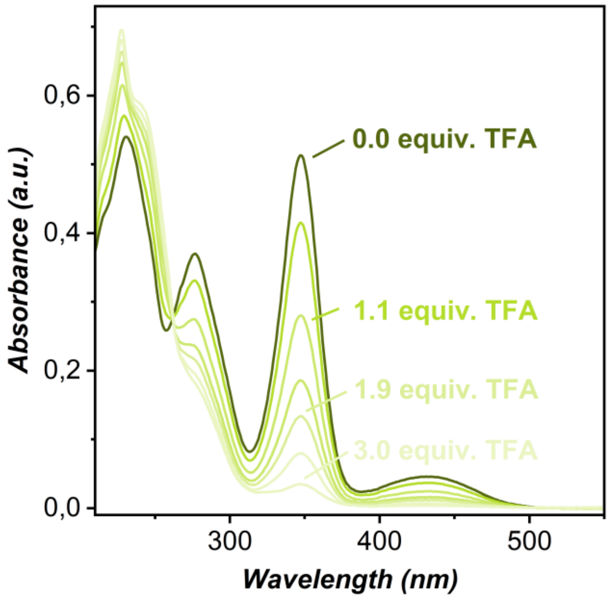

Figure S104. Absorption spectra of **pseudo-*m*-NB** in CH_3CN as increasing amounts of TFA are titrated. Isosbestic points: 262 nm.

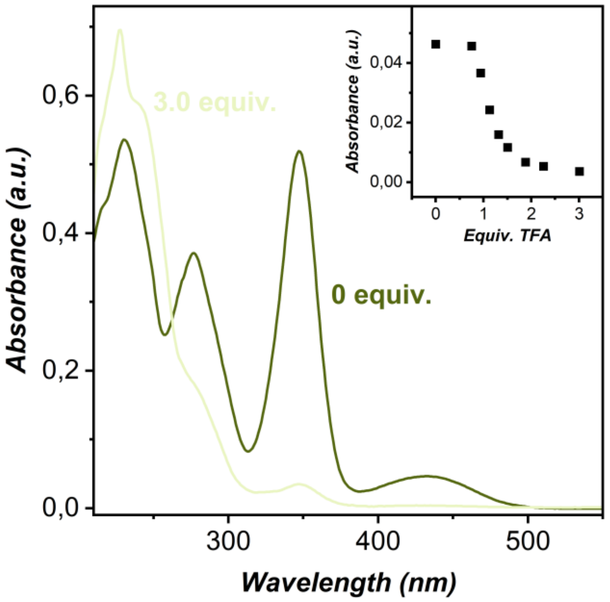

Figure S105. Absorption spectra of **pseudo-*m*-NB** in CH_3CN before and after saturation with TFA, with inset of absorbance at 431 nm as a function of TFA molar equivalents.

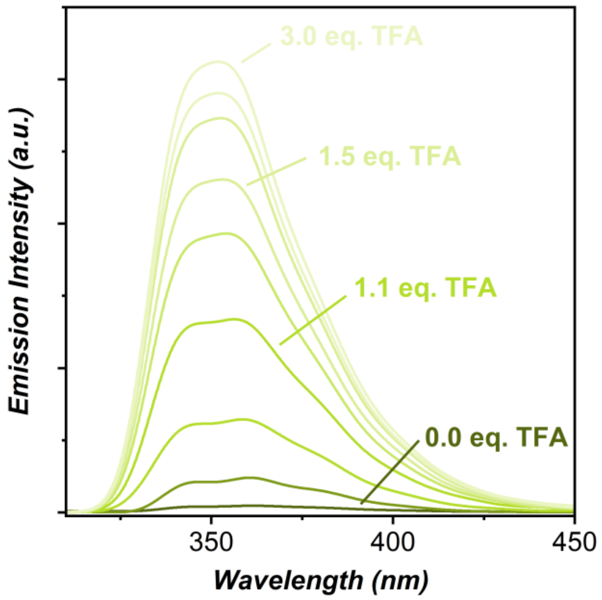

Figure S106. Fluorescence spectra of **pseudo-*m*-NB** in CH_3CN excited at 290 nm as increasing amounts of TFA are titrated. (emission from 400-800 nm is dominated by 2nd order diffraction)

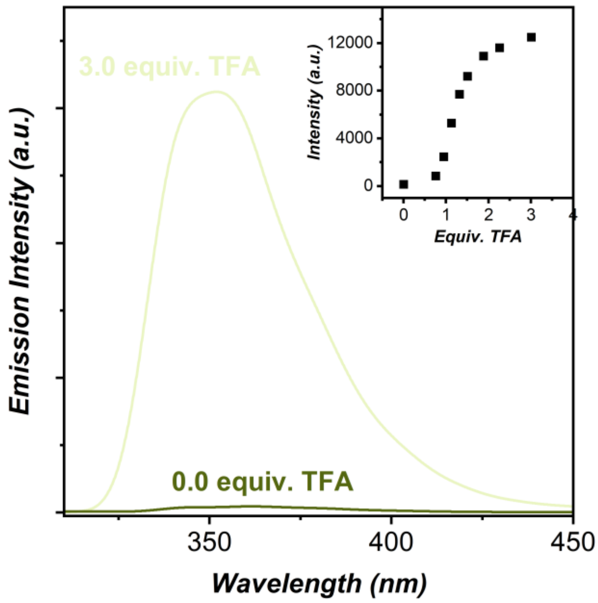

Figure S107. Fluorescence spectra of **pseudo-*m*-NB** in CH_3CN excited at 290 nm before and after saturation with TFA, with inset of emission at 353 nm as a function of TFA molar equivalents.

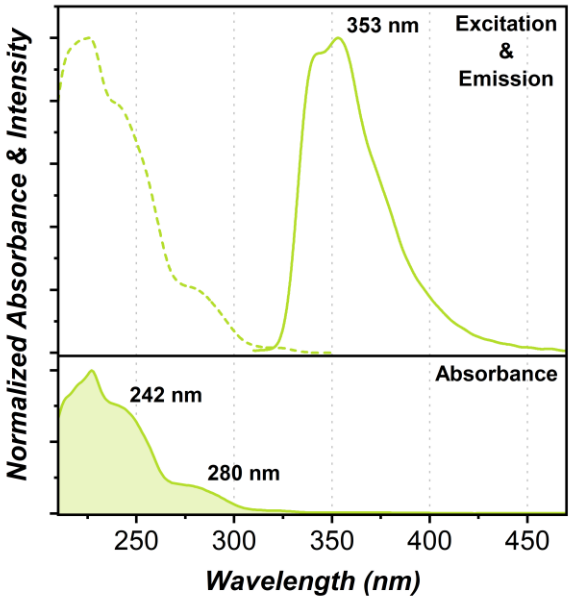

Figure S108. Excitation, emission (λ_{exc} 300 nm), and absorption spectra of **pseudo-*m*-NB** in CH_3CN with sufficient TFA for full protonation.

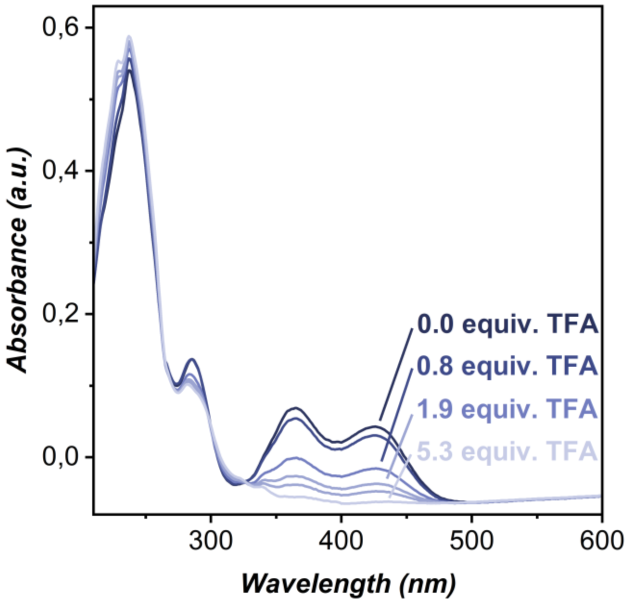

Figure S109. Absorption spectra of **pseudo-*p*-NB** in CH_3CN as increasing amounts of TFA are titrated. Isosbestic points: 327, 300, 263 nm.

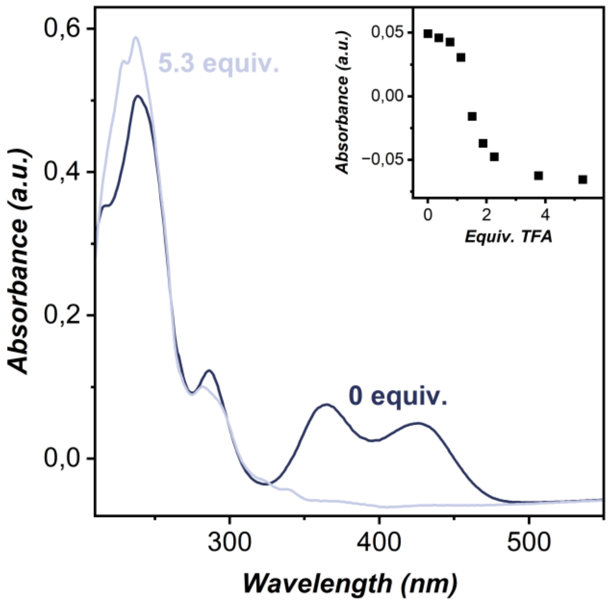

Figure S110. Absorption spectra of ***m*-NB** in CH_3CN before and after saturation with TFA, with inset of absorbance at 425 nm as a function of TFA molar equivalents.

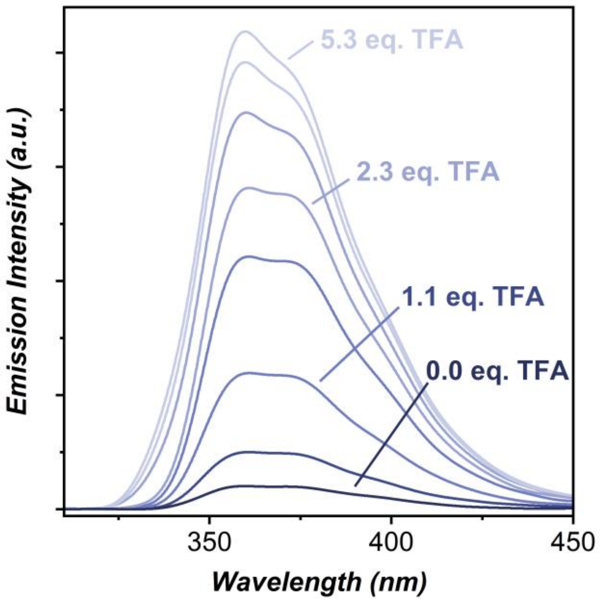

Figure S111. Fluorescence spectra of **pseudo-*p*-NB** in CH_3CN excited at 290 nm as increasing amounts of TFA are titrated.

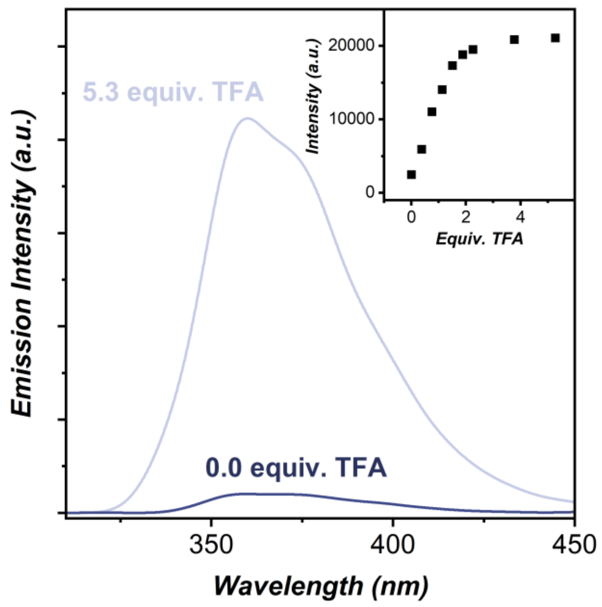

Figure S112. Fluorescence spectra of **pseudo-*p*-NB** in CH_3CN excited at 290 nm before and after saturation with TFA, with inset of emission at 355 nm as a function of TFA molar equivalents.

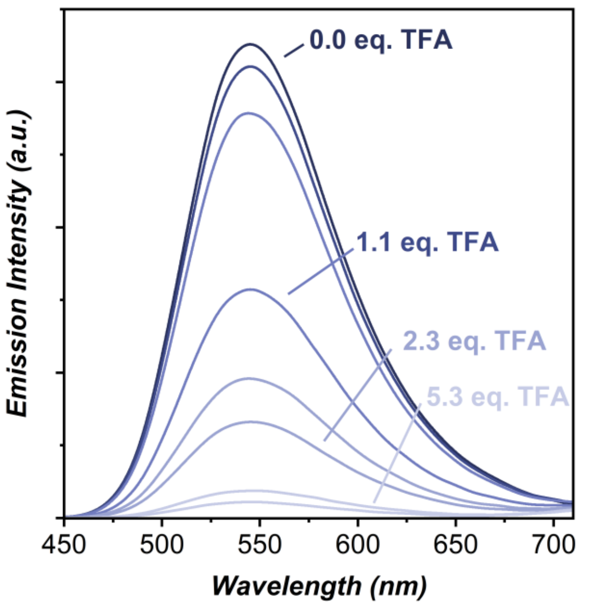

Figure S113. Fluorescence spectra of **pseudo-*p*-NB** in CH_3CN excited at 420 nm as increasing amounts of TFA are titrated.

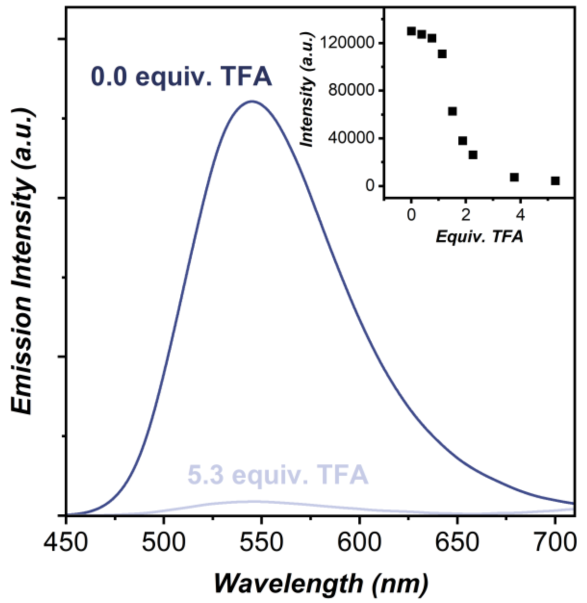

Figure S114. Fluorescence spectra of **pseudo-*p*-NB** in CH_3CN excited at 420 nm before and after saturation with TFA, with inset of emission at 549 nm as a function of TFA molar equivalents.

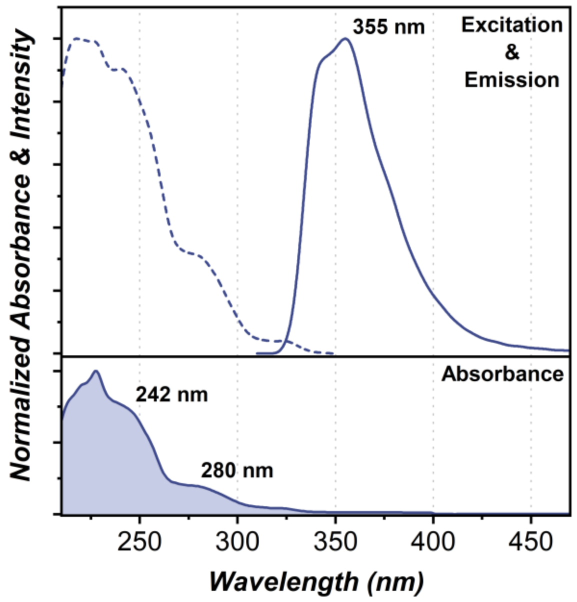

Figure S115. Excitation, emission (λ_{exc} 300 nm), and absorption spectra of **pseudo-*m*-NB** in CH_3CN with sufficient TFA for full protonation.

Section S15 Solvatochromism

To compare the prepared betaines with known zero-overlap fluorophores with large stokes shifts,[69] the overlap of absorbance and emission spectra was calculated. The intensities at the point where the normalised absorption and emission cross are greater than 0.034, therefore the betaines do not qualify as zero-overlap fluorophores. However, it is clear that ***m*-PB** and **pseudo-*m*-NB** have lesser spectral overlap than their *para*-analogues.

Table S30. Calculated spectral overlap parameters of prepared betaines.

	***m*-PB**	***p*-PB**	**pseudo-*m*-NB**	**pseudo-*p*-NB**
**Spectral Overlap (%)***	~0.1	~1.4	~0.3	~0.8
**Normalised Intensity at Cross-over Point**	0.0113	0.0816	0.0176	0.0439

*calculated using the normalised absorbance and emission spectra with the 'overlap area' function in Origin.

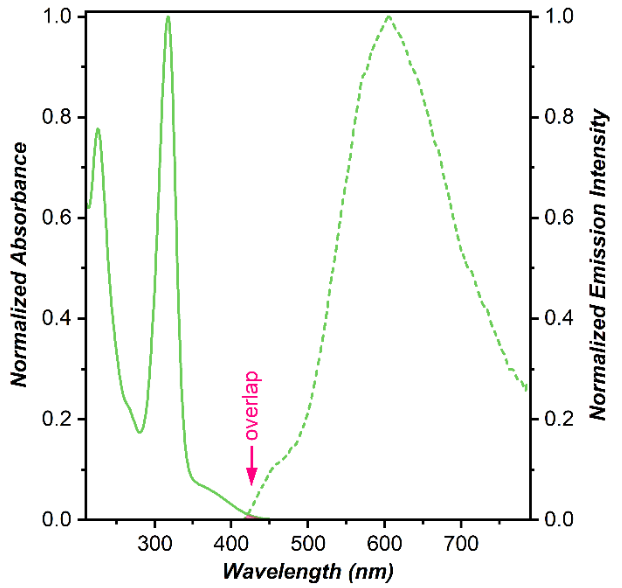

Figure S116. Normalised absorbance (solid) and emission (dashed, λ_{exc} 400 nm) of acetonitrile solutions of ***m*-PB**. Overlap shown in pink.

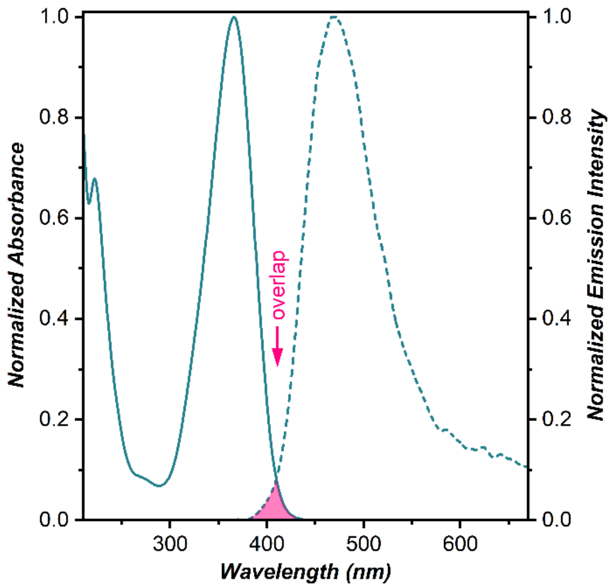

Figure S117. Normalised absorbance (solid) and emission (dashed, λ_{exc} 370 nm) of acetonitrile solutions of ***p*-PB**. Overlap shown in pink.

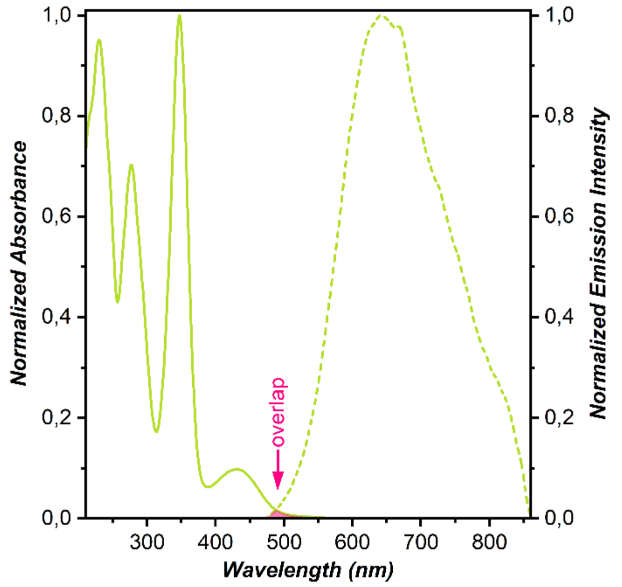

Figure S118. Normalised absorbance (solid) and emission (dashed, λ_{exc} 430 nm) of acetonitrile solutions of **pseudo-*m*-NB**. Overlap shown in pink.

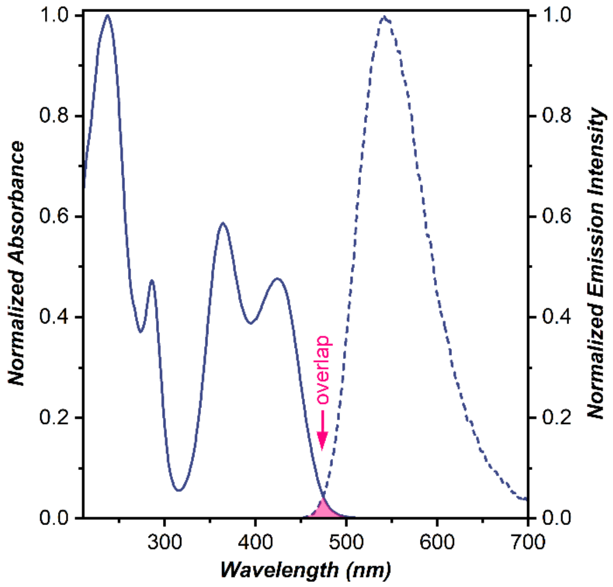

Figure S1119. Normalised absorbance (solid) and emission (dashed, λ_{exc} 420 nm) of acetonitrile solutions of **pseudo-*p*-NB**. Overlap shown in pink.

Table S31. Absorbance maxima and molar absorption coefficients of compounds in various solvents.

Compound	Solvent	Longest-wavelength Absorbance Maximum, λ_{abs} (nm)	Absorption Coefficient at λ_{abs}, ε (L · mol^{-1} · cm^{-1})	E_{gap}^{opt} (eV)$^{+}$
***m*-PB**	acetone	360*	-	-
	acetonitrile	375*	1 600	2.986
	dimethylformamide	377*	-	-
	dimethylsulfoxide	377*	-	-
	formamide	360*	-	-
	benzonitrile	390*	-	-
***p*-PB**	acetone	377	-	-
	acetonitrile	366	29 500	3.004
	dimethylformamide	372	-	-
	dimethylsulfoxide	370	-	-
	formamide	356	-	-
	benzonitrile	384	-	-
**pseudo-*m*-NB**	acetone	444	-	-
	acetonitrile	431	2 800	2.559
	dimethylformamide	438	-	-
	dimethylsulfoxide	434	-	-
	formamide	412	-	-
	benzonitrile	449	-	-
**pseudo-*p*-NB**	acetone	439	-	-
	acetonitrile	424	13 600	2.583
	dimethylformamide	433	-	-
	dimethylsulfoxide	428	-	-
	formamide	406	-	-
	benzonitrile	446	-	-

*determined using a curve-fitting approach.

+determined using the 0-0 transition (detailed in Table S18).

Relative quantum yields of fluorescence were determined using the relative method outlined by Fery-Forgues and Lavabre[5] according to the following formula:

$$\Phi_F^{unk} = \frac{A_{std}}{A_{unk}} \times \frac{F_{unk}}{F_{std}} \times \left(\frac{n_{unk}}{n_{std}}\right)^2 \times \Phi_F^{std}$$

Table S32. Fluorescence maxima and quantum yields of compounds in various solvents.

Compound	Solvent	Fluorescence Maximum, λ_{em} (nm)	Fluorescence Quantum Yield, Φ_F	Stokes Shift (cm^{-1})
***m*-PB**	acetone	602	0.01(5) ± 0.01	10127
	acetonitrile	605	0.01(4) ± 0.01	10138
	dimethylformamide	595	0.01(8) ± 0.01	9789
	dimethylsulfoxide	591	0.03(2) ± 0.01	9605
	formamide	568	0.01(7) ± 0.01	10250
	benzonitrile	579	0.01(6) ± 0.01	8370
***p*-PB**	acetone	472	< 0.01 (0.0042)	5339
	acetonitrile	470	< 0.01 (0.00059)	6046
	dimethylformamide	465	< 0.01 (0.0036)	5376
	dimethylsulfoxide	464	< 0.01 (0.0077)	5475
	formamide	462	< 0.01 (0.0062)	6445
	benzonitrile	476	< 0.01 (0.0042)	5033
**pseudo-*m*-NB**	acetone	638	0.03(6) ± 0.01	6849
	acetonitrile	641	0.02(3) ± 0.01	7601
	dimethylformamide	625	0.04(4) ± 0.01	6831
	dimethylsulfoxide	614	0.07(6) ± 0.01	6755
	formamide	622	0.05(1) ± 0.01	8195
	benzonitrile	660	0.04(5) ± 0.01	7120
**pseudo-*p*-NB**	acetone	547	0.436 ± 0.022*	5303
	acetonitrile	544	0.469 ± 0.023*	4397
	dimethylformamide	537	0.606 ± 0.030*	4473
	dimethylsulfoxide	534	0.750 ± 0.037*	4638
	formamide	534	0.760 ± 0.038*	5904
	benzonitrile	558	0.729 ± 0.036*	4500

*determined using an integrating sphere.

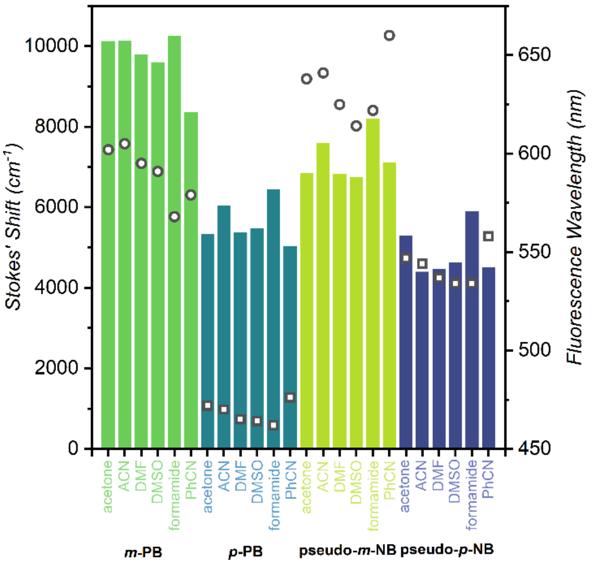

Figure S120. Stokes shifts of betaines in different solvents by colour: ***m*-PB**, ***p*-PB**, **pseudo-*m*-NB**, **pseudo-*p*-NB** and associated fluorescence peak wavelengths (circles and squares).

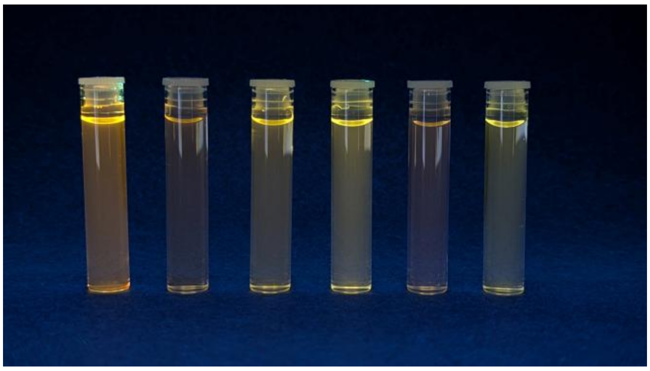

Figure S121. Photograph of ~1 mM ***m*-PB** solutions in (L-R): PhCN, acetone, DMF, DMSO, CH_3CN, formamide taken with night-mode on a Google Pixel 7.

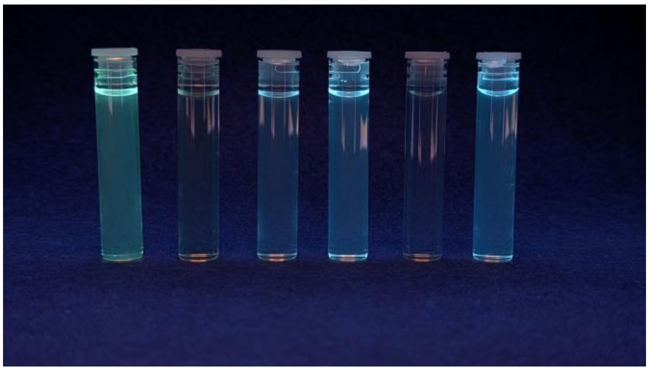

Figure S122. Photograph of ~1 mM ***p*-PB** solutions in (L-R): PhCN, acetone, DMF, DMSO, CH_3CN, formamide taken with night-mode on a Google Pixel 7.

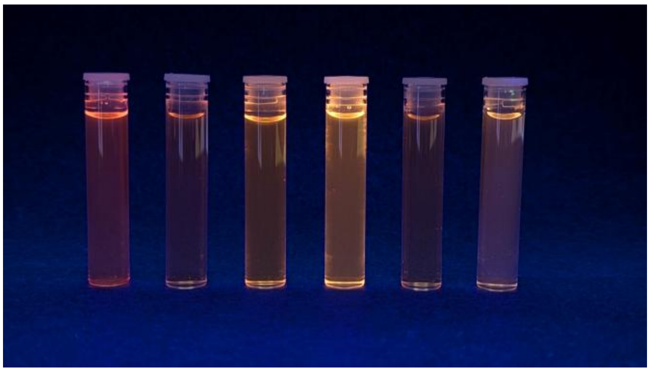

Figure S123. Photograph of ~1 mM **pseudo-*m*-NB** solutions in (L-R): PhCN, acetone, DMF, DMSO, CH_3CN, formamide taken with night-mode on a Google Pixel 7.

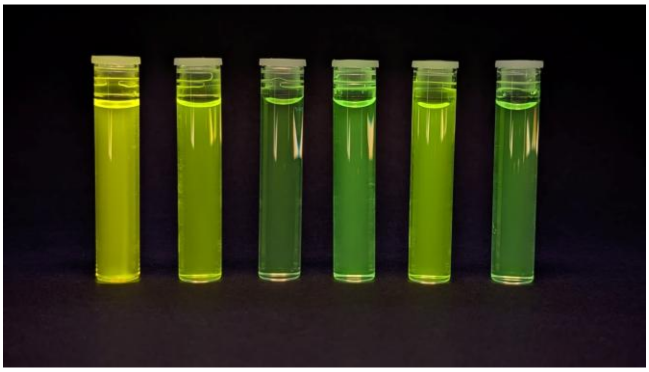

Figure S124. Photograph of ~1 µM **pseudo-*p*-NB** solutions in (L-R): PhCN, acetone, DMF, DMSO, CH_3CN, formamide taken with night-mode on a Google Pixel 7.

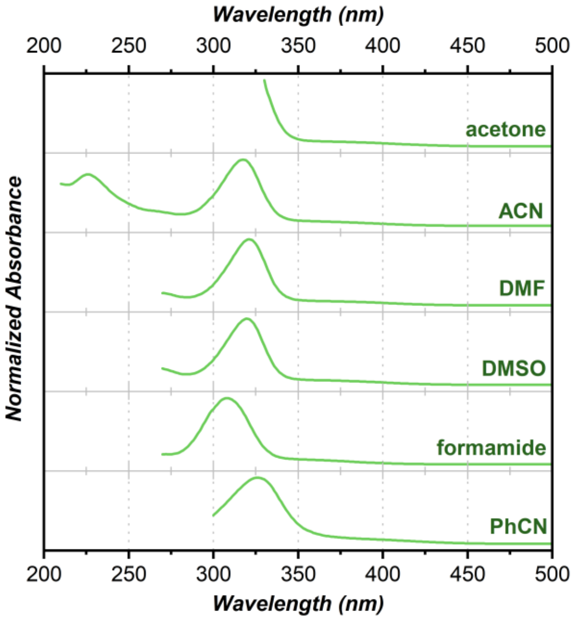

Figure S125. Absorbance spectra of ***m*-PB** in various solvents.

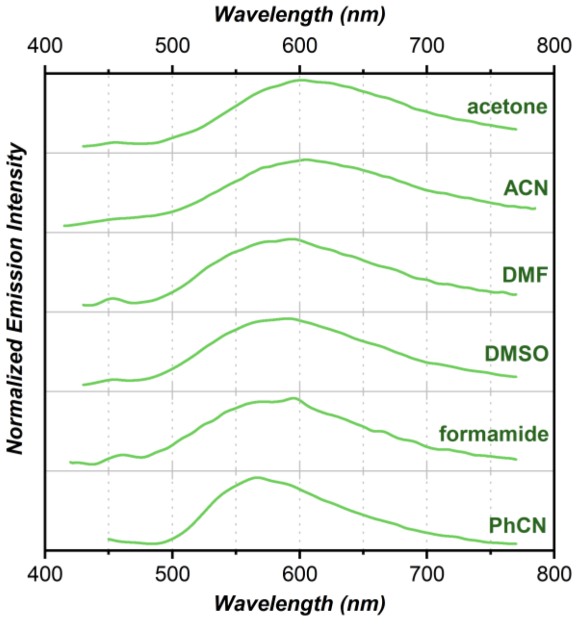

Figure S126. Fluorescence spectra of ***m*-PB** excited at 400 nm, in various solvents.

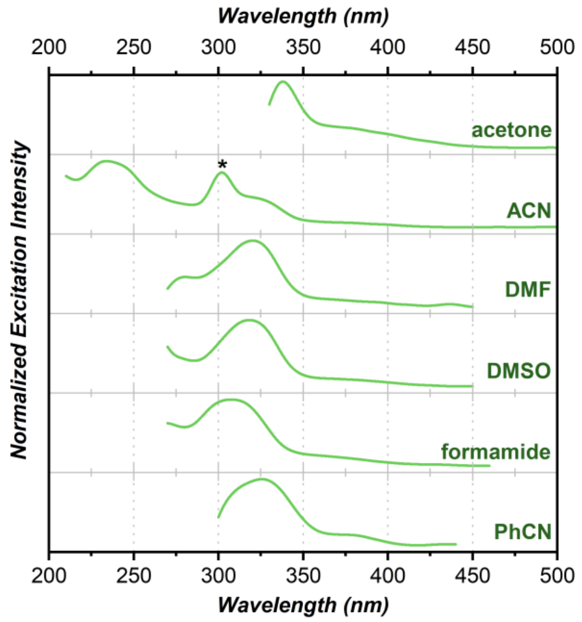

Figure S127. Excitation spectra of ***m*-PB** from 600 nm emission, in various solvents. (*second order diffraction peak)

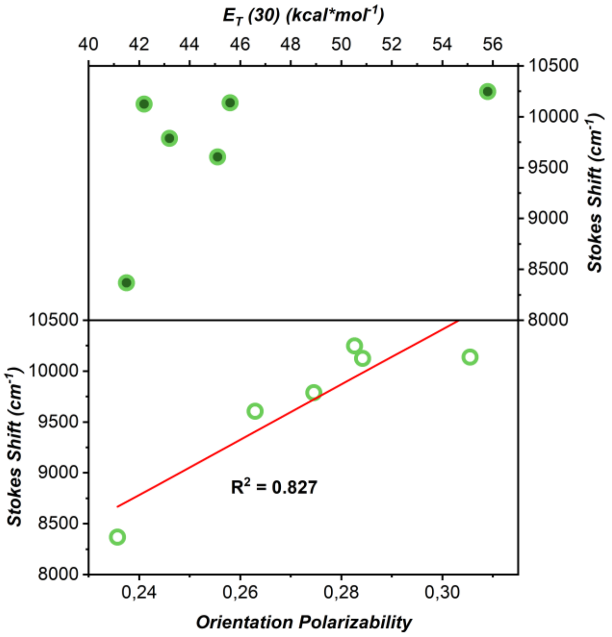

Figure S128. Plot of $E_T(30)$ parameters[70] and orientation polarisability[71,72] versus Stokes shift for ***m*-PB** in various solvents. The red line shows the linear regression best fit with R^2.

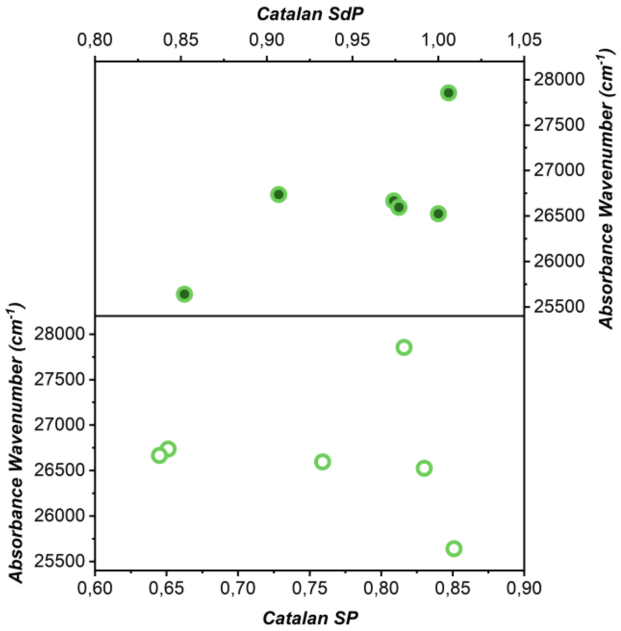

Figure S129. Plot of Catalán’s SdP and SP parameters[73] versus absorbance peak wavenumber for ***m*-PB** in various solvents.

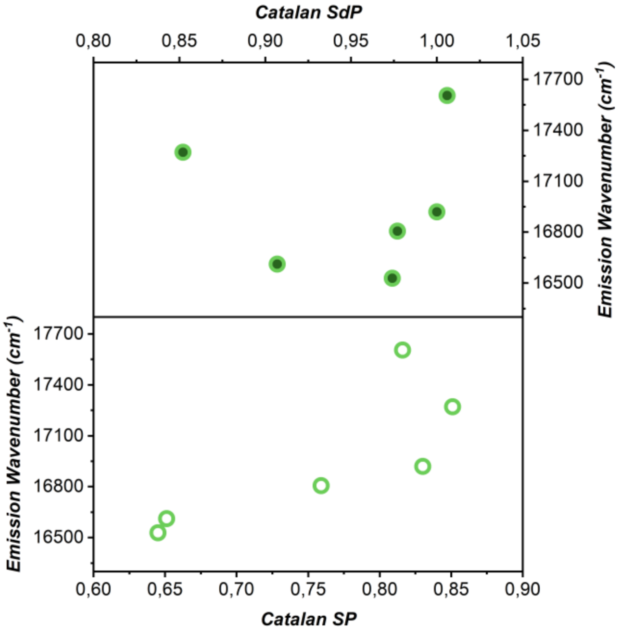

Figure S130. Plot of Catalán’s SdP and SP parameters[73] versus emission peak wavenumber for ***m*-PB** in various solvents.

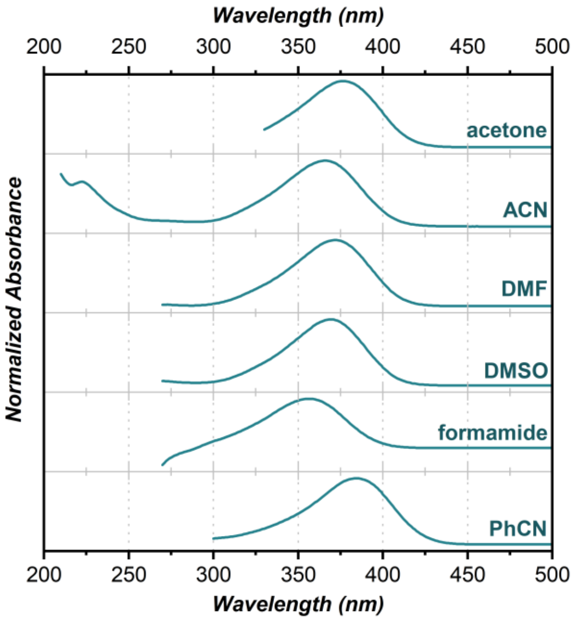

Figure S131. Absorbance spectra of ***p*-PB** in various solvents.

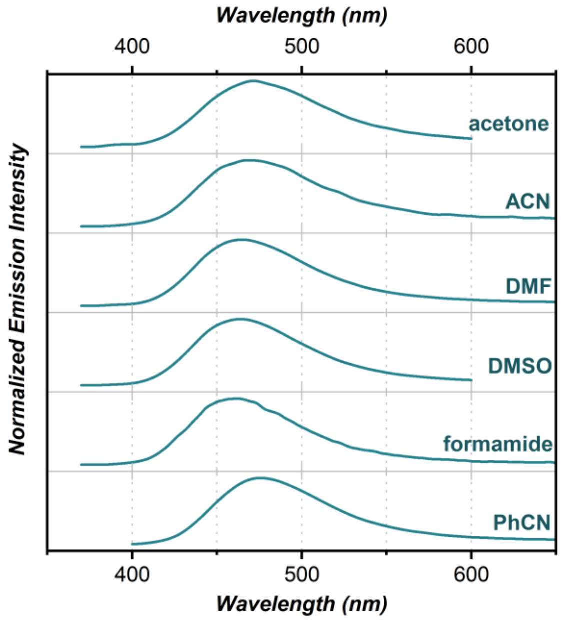

Figure S132. Fluorescence spectra of ***p*-PB** excited at 360 nm, in various solvents.

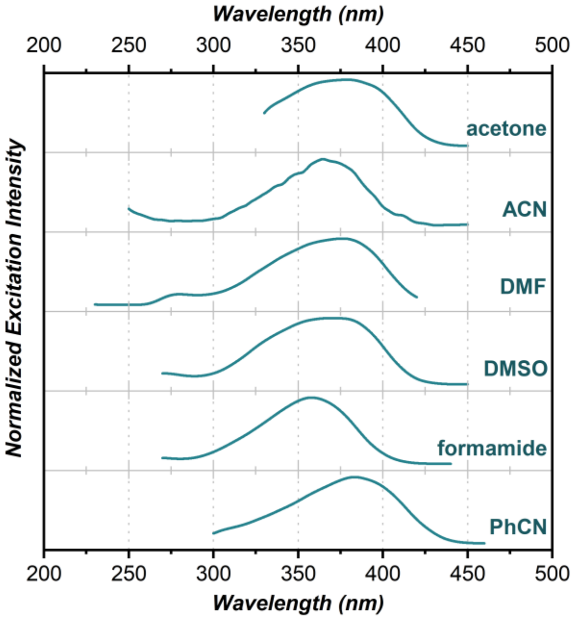

Figure S133. Excitation spectra of ***p*-PB** from 460 nm emission, in various solvents.

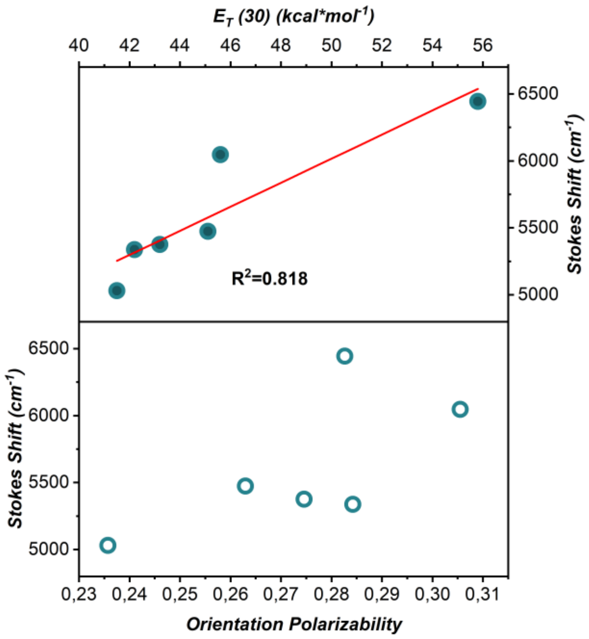

Figure S134. Plot of $E_T(30)$ parameters[70] and orientation polarisability[71,72] versus Stokes shift for ***p*-PB** in various solvents. The red line shows the linear regression best fit with R^2.

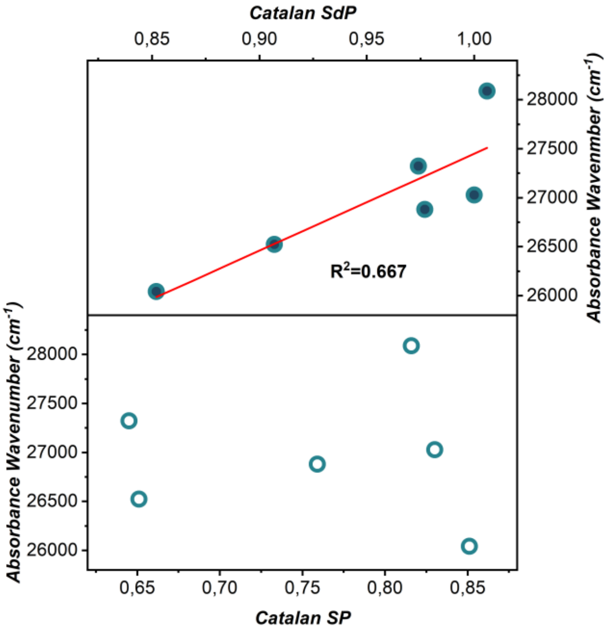

Figure S135. Plot of Catalán's SdP and SP parameters[73] versus absorbance peak wavenumber for ***p*-PB** in various solvents. The red line shows the linear regression best fit with R^2.

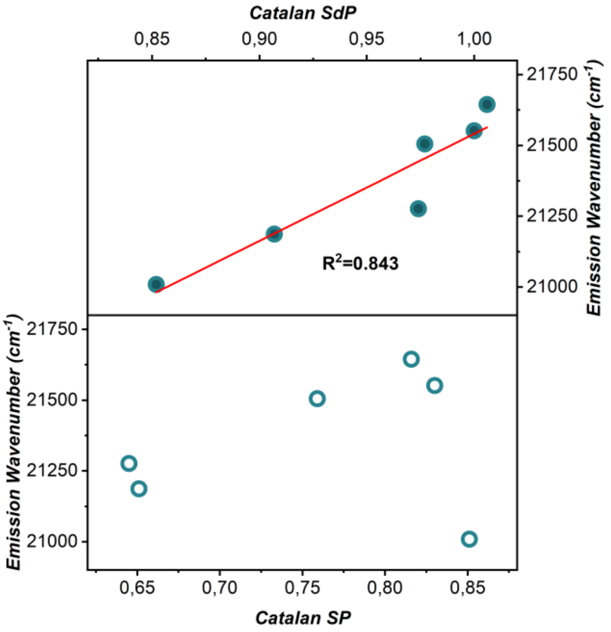

Figure S136. Plot of Catalán's SdP and SP parameters[73] versus emission peak wavenumber for ***p*-PB** in various solvents. The red line shows the linear regression best fit with R^2.

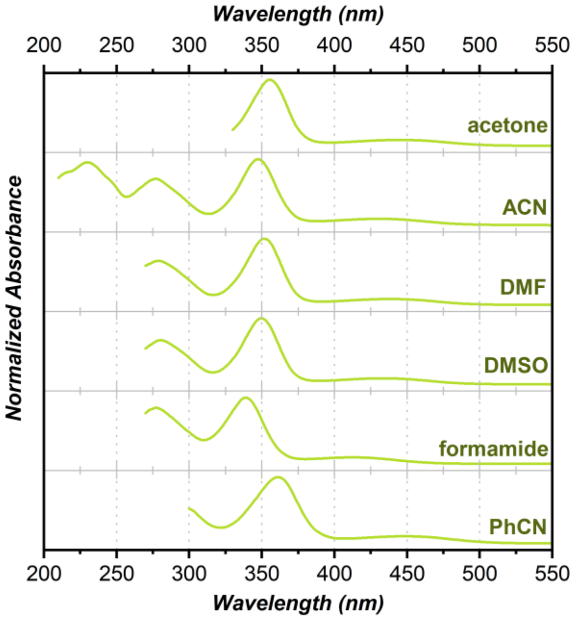

Figure S137. Absorbance spectra of **pseudo-*m*-NB** in various solvents.

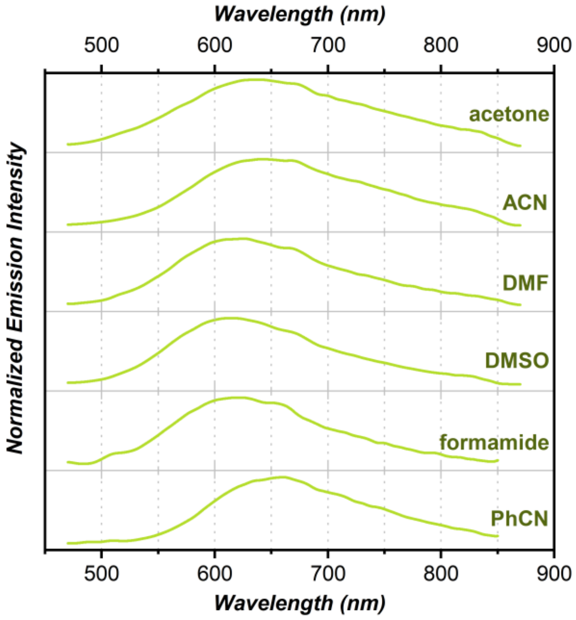

Figure S138. Fluorescence spectra of **pseudo-*m*-NB** excited at 430 nm, in various solvents.

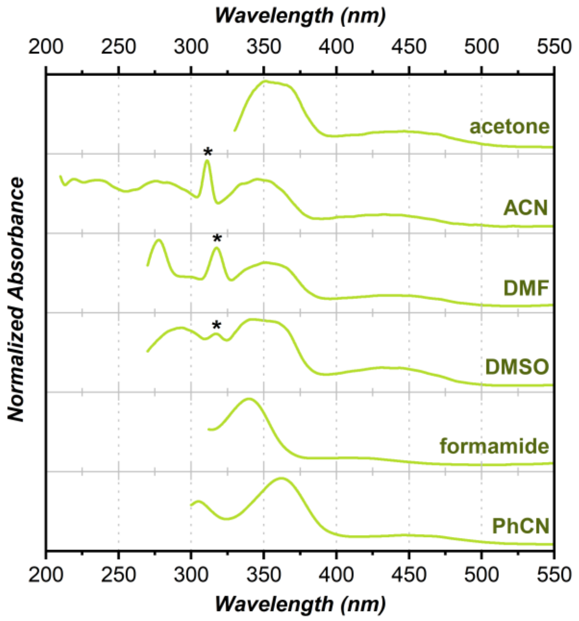

Figure S139. Excitation spectra of **pseudo-*m*-NB** from 650 nm emission, in various solvents. (*second order diffraction peak)

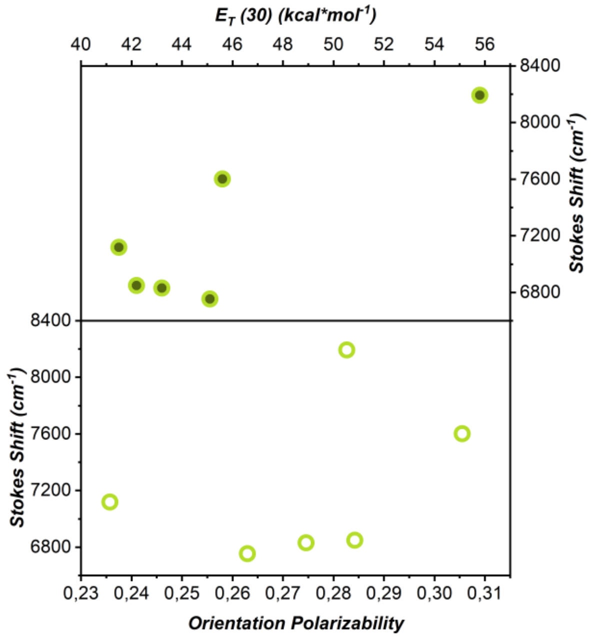

Figure S140. Plot of $E_T(30)$ parameters[70] and orientation polarisability[71,72] versus Stokes shift for **pseudo-*m*-NB** in various solvents.

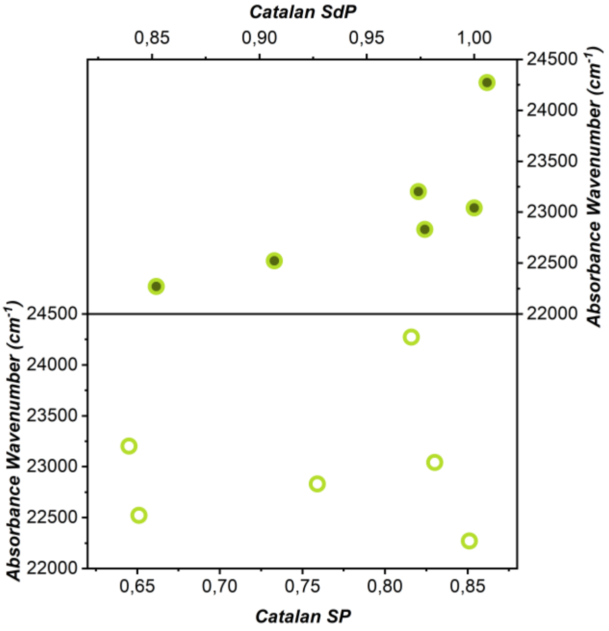

Figure S141. Plot of Catalán's SdP and SP parameters[73] versus absorbance peak wavenumber for **pseudo-*m*-NB** in various solvents.

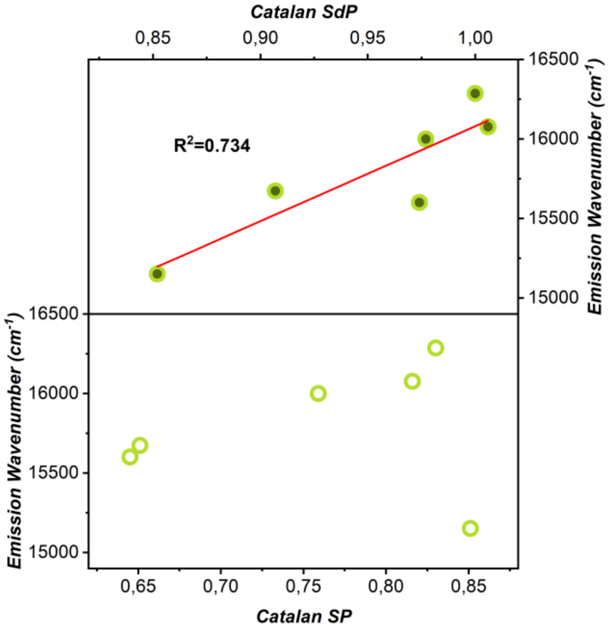

Figure S142. Plot of Catalán's SdP and SP parameters[73] versus emission peak wavenumber for **pseudo-*m*-NB** in various solvents. The red line shows the linear regression best fit with R^2.

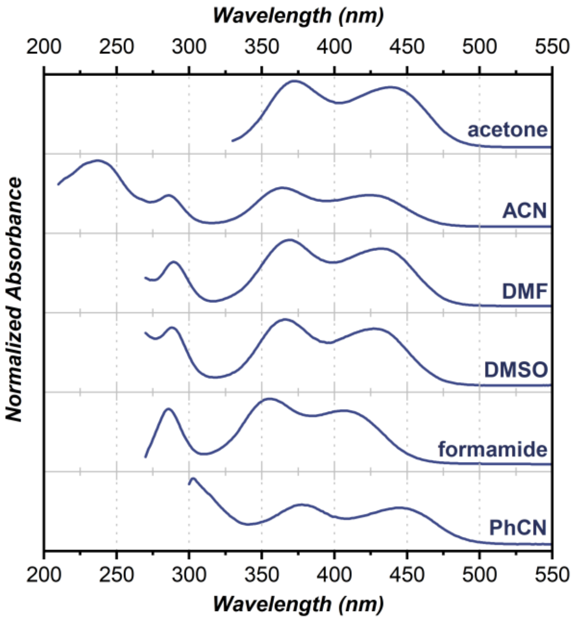

Figure S143. Absorbance spectra of **pseudo-*p*-NB** in various solvents.

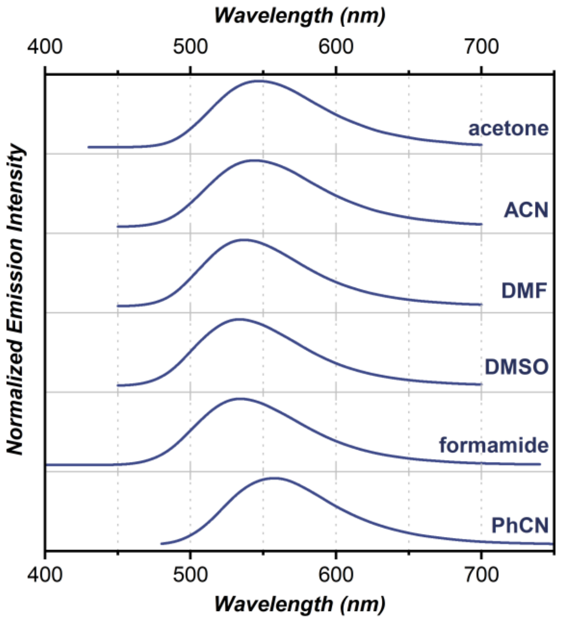

Figure S144. Fluorescence spectra of **pseudo-*p*-NB** excited at 430 nm, in various solvents.

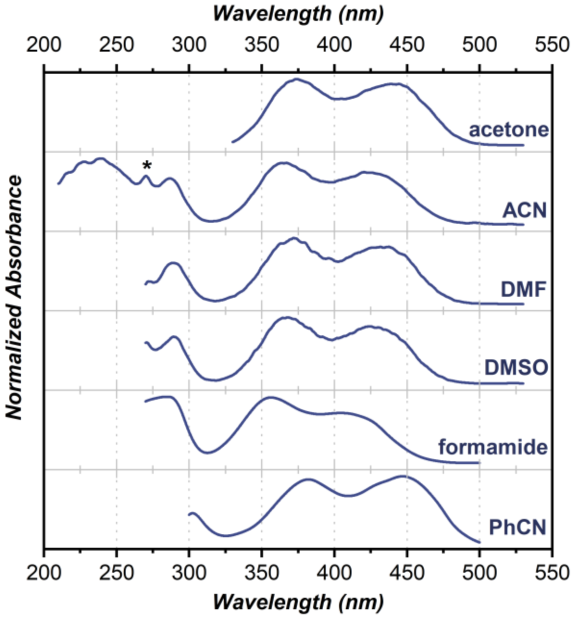

Figure S145. Excitation spectra of **pseudo-*p*-NB** from 550 nm emission, in various solvents. (*second order diffraction peak)

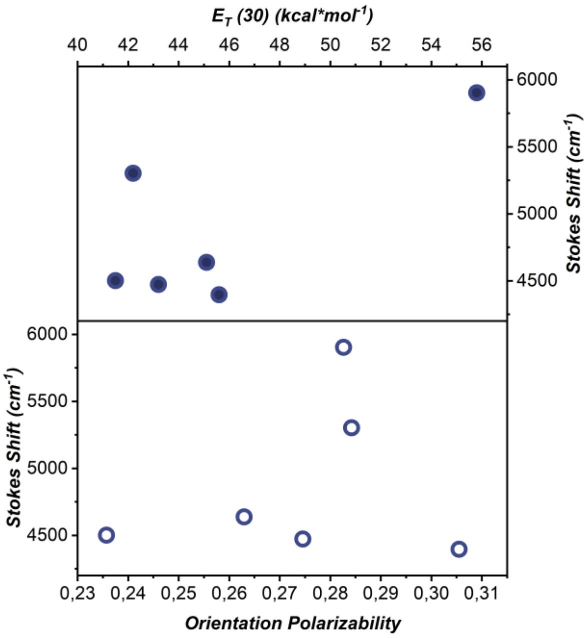

Figure S146. Plot of $E_T(30)$ parameters[70] and orientation polarisability[71,72] versus Stokes shift for **pseudo-*p*-NB** in various solvents.

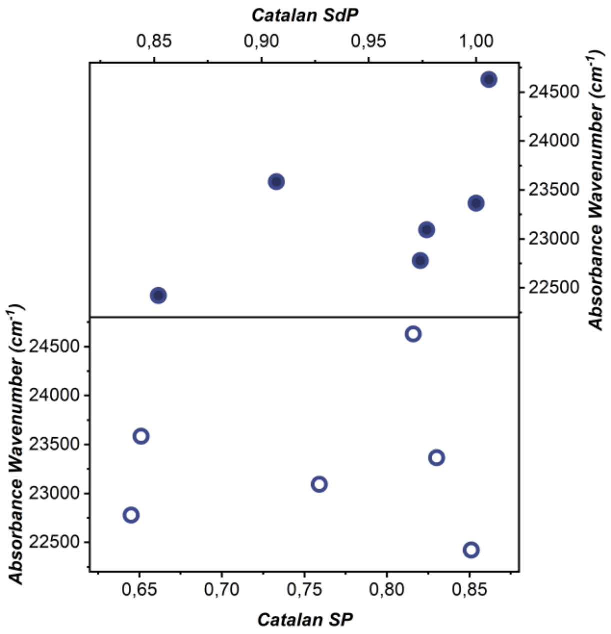

Figure S147. Plot of Catalán's SdP and SP parameters[73] versus absorbance peak wavenumber for **pseudo-*p*-NB** in various solvents.

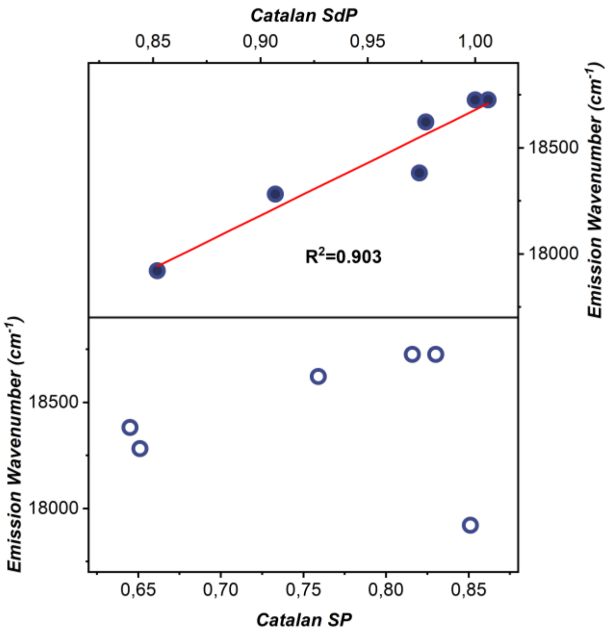

Figure S148. Plot of Catalán's SdP and SP parameters[73] versus emission peak wavenumber for **pseudo-*p*-NB** in various solvents. The red line shows the linear regression best fit with R^2.

Section S16 Computational Modelling of Rotational Energy Barriers

Previously optimized structures at the CAM-B3LYP/def2-SVP level in CH_3CN (CPCM) for all betaines in the 1) singlet ground state, 2) lowest singlet excited state, and 3) lowest triplet excited state, were used as input structures for relaxed surface scans around the aryl-imidazolium (alpha, D_α) and aryl-malonide (beta, D_β) dihedral angles. The labelling is based on work from literature.[66] Calculations were done at the CAM-B3LYP/def2-SVP-D4 level in CH_3CN (CPCM) including empirical dispersion D4.[28,74]

Table S33. Calculated rotational energy barriers for overcoming the aryl-imidazolium (α) and aryl-malonide (β) bonds in various electronic states.

Dihedral	State	*m*-PB (kJ/mol)	*p*-PB (kJ/mol)	pseudo-*m*-NB (kJ/mol)	pseudo-*p*-NB (kJ/mol)
D_α	**S_0**	33.38	29.53	34.37	33.17
	**S_1**	52.66	50.30	21.42	23.95
	**T_1**	52.74	47.60	21.36	18.42
D_β	**S_0**	41.49	49.27	42.66	45.75
	**S_1**	34.78	14.11	51.48	34.54
	**T_1**	60.44	30.33	63.57	45.98

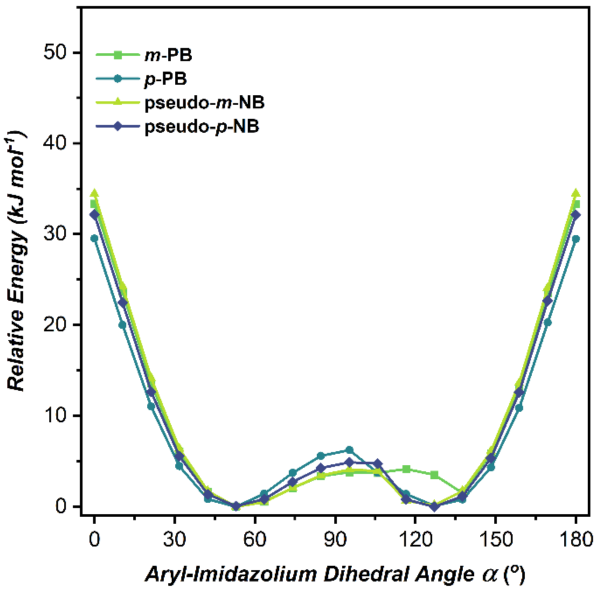

Figure S149. Relaxed surface scans of the dihedral angle D_α of all betaines in S_0.

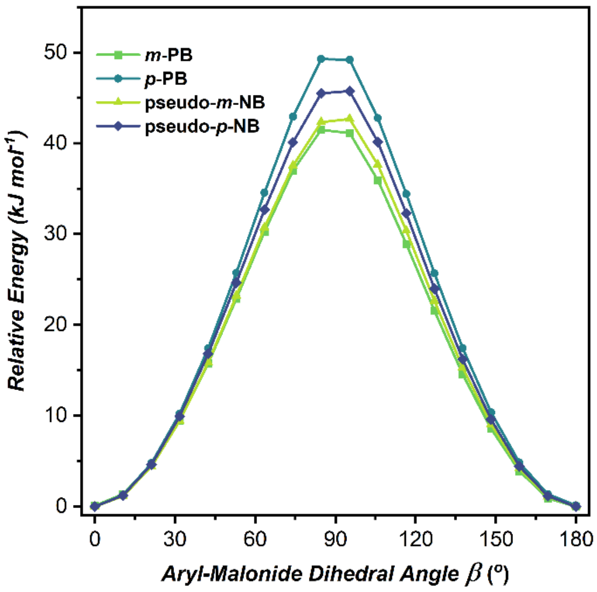

Figure S150. Relaxed surface scans of the dihedral angle D_β of all betaines in S_0.

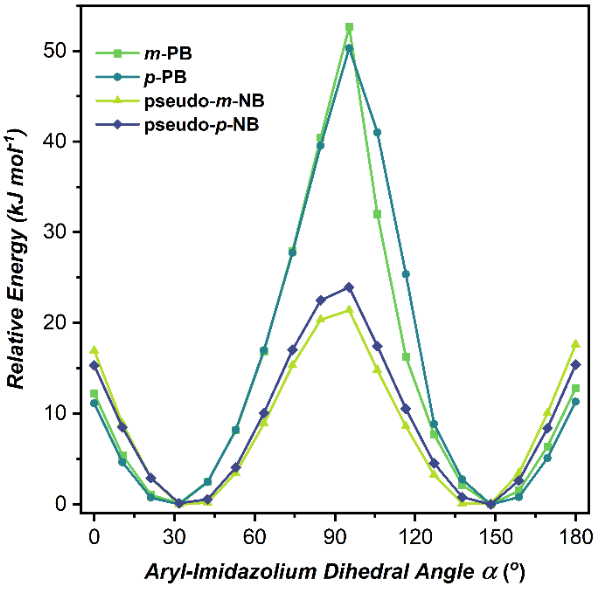

Figure S151. Relaxed surface scans of the dihedral angle D_α of all betaines in S_1.

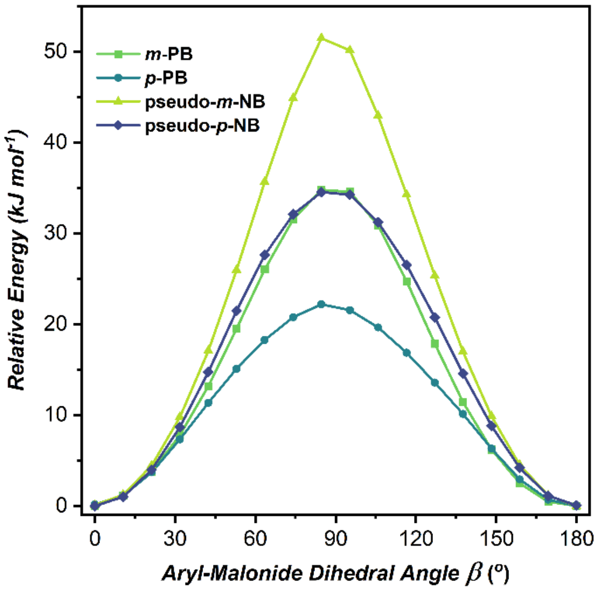

Figure S152. Relaxed surface scans of the dihedral angle D_β of all betaines in S_1.

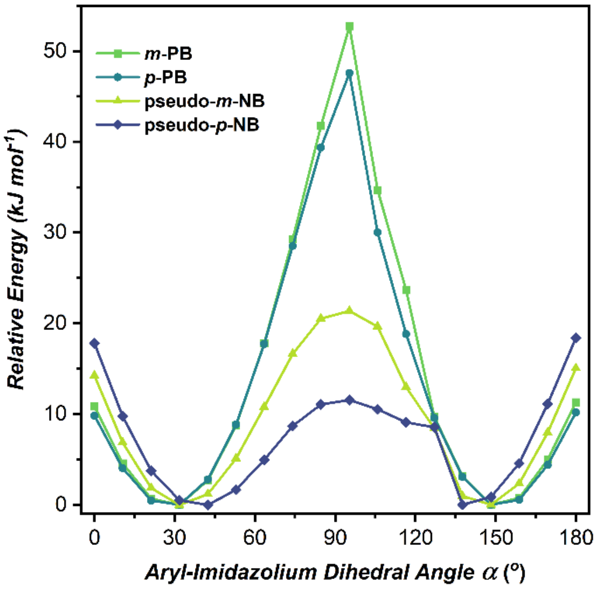

Figure S153. Relaxed surface scans of the dihedral angle D_α of all betaines in T_1.

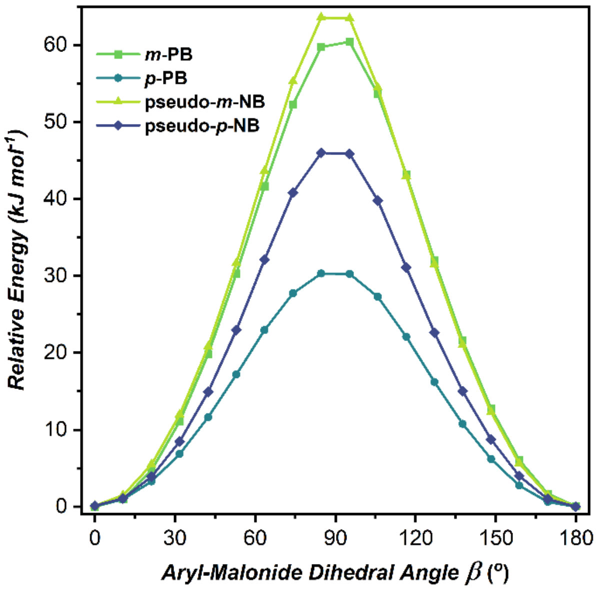

Figure S154. Relaxed surface scans of the dihedral angle D_β of all betaines in T_1.

The rotational energy barriers obtained from calculation were compared to changes in the aryl – imidazolium and aryl – malonide bond lengths, as well as the Mayer, Fuzzy, and Laplacian bond orders obtained from Multiwfn analysis on the same structures. The only index which gave reasonable agreement was the Laplacian bond order, plotted below.[47] The rotational barrier around dihedral Dβ agrees quite poorly, indicating that changes in that barrier do not originate (solely) from bond strength.

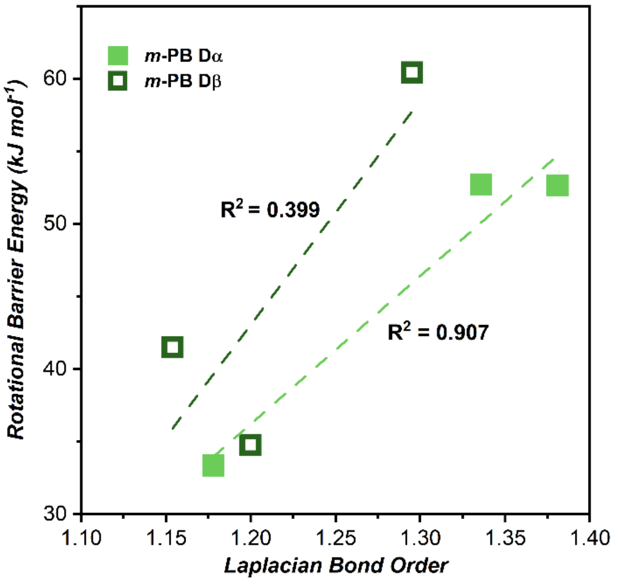

Figure S155. Calculated rotational barrier around Dα and Dβ versus Laplacian bond order for ***m*-PB** in S_0, S_1, and T_1.

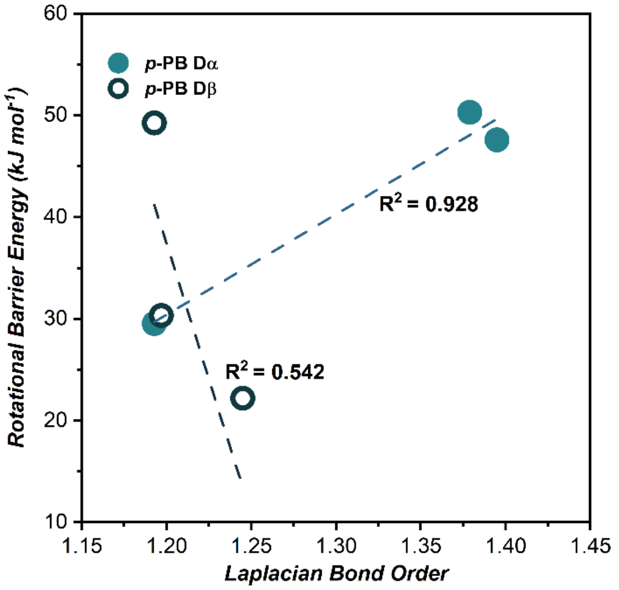

Figure S156. Calculated rotational barrier around Dα and Dβ versus Laplacian bond order for ***p*-PB** in S_0, S_1, and T_1.

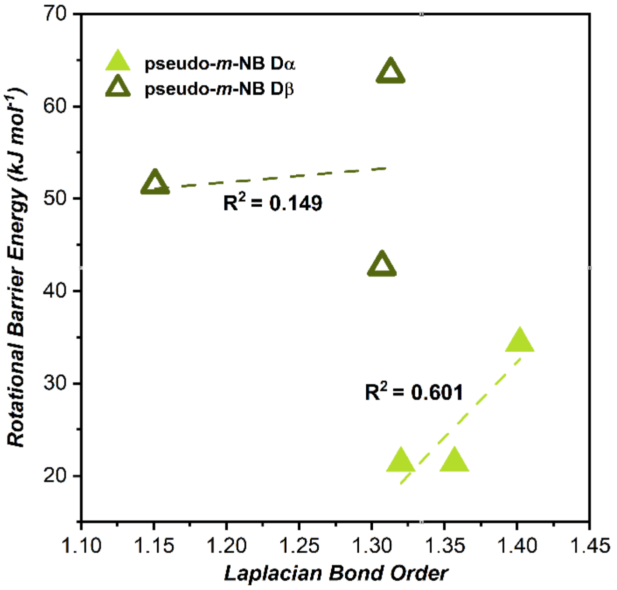

Figure S157. Calculated rotational barrier around Dα and Dβ versus Laplacian bond order for **pseudo-*m*-NB** in S_0, S_1, and T_1.

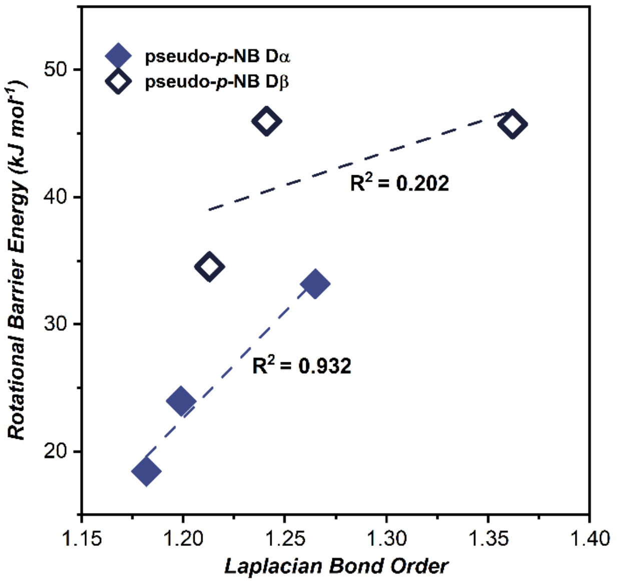

Figure S158. Calculated rotational barrier around Dα and Dβ versus Laplacian bond order for **pseudo-*p*-NB** in S_0, S_1, and T_1.

To explore possible non-radiative de-excitation pathways available to the zwitterions, the potential energy surfaces of some low-lying excited states were calculated as a function of rotation around D_α and D_β. Calculations were done at the CAM-B3LYP/def2-SVP-D4 level in CH_3CN (CPCM) including empirical dispersion D4.

Along the D_α potential energy surfaces, intersystem crossing from S_1 to either T_3 or T_4 can be achieved between a dihedral angle of 20°-60°. Considering that these angles are the local minima on both the S_0 and S_1 surfaces, it is quite likely for intersystem crossing to occur through rotation of the aryl-imidazolium bond, therefore quenching the fluorescence.

Along the D_β potential energy surfaces of ***m*-PB** and ***p*-PB**, the S_1 intersects with a triplet state at 80°-90°, which is an energy maximum on the S_0 and S_1 surface. Population of these conformers is less likely, and therefore rotation of the aryl-malonide bond does not contribute to intersystem crossing. The singlet and triplet surfaces of **pseudo-*m*-NB** and **pseudo-*p*-NB** do not intersect.

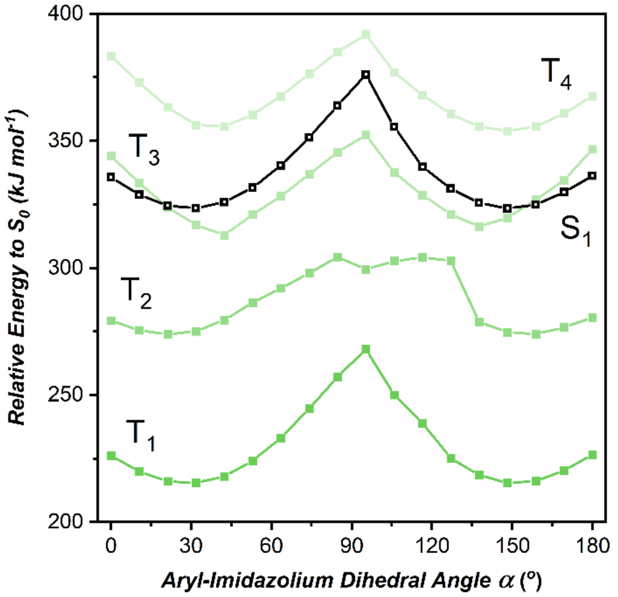

Figure S159. Relaxed surface scans of the dihedral angle D_α of ***m*-PB** in S_1 and T_1-T_4 relative to S_0.

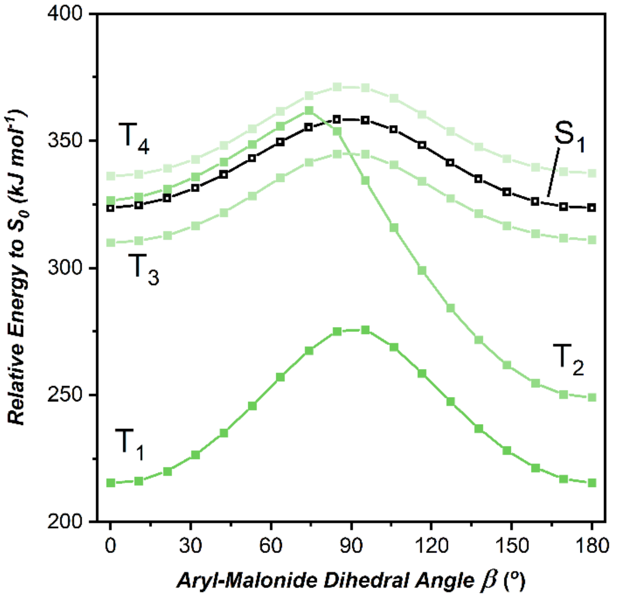

Figure S160. Relaxed surface scans of the dihedral angle D_β of ***m*-PB** in S_1 and T_1-T_4 relative to S_0.

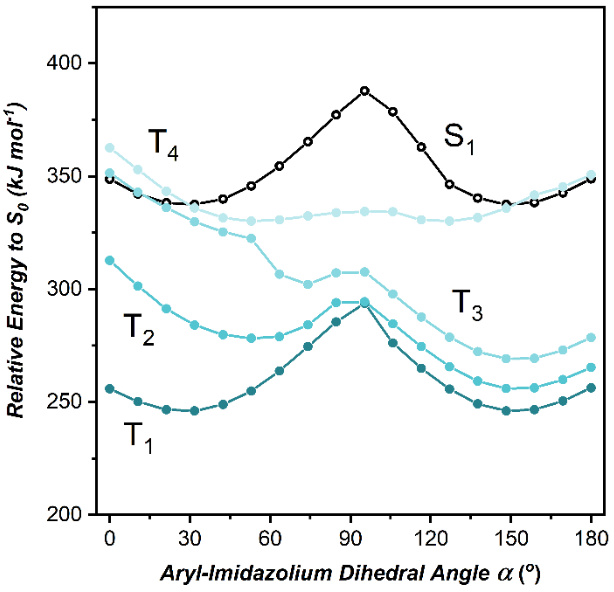

Figure S161. Relaxed surface scans of the dihedral angle D_α of ***p*-PB** in S_1 and T_1-T_4 relative to S_0.

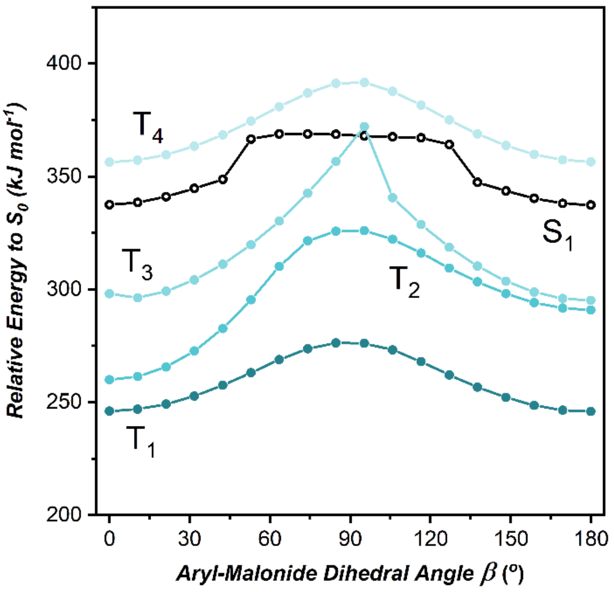

Figure S162. Relaxed surface scans of the dihedral angle D_β of ***p*-PB** in S_1 and T_1-T_4 relative to S_0.

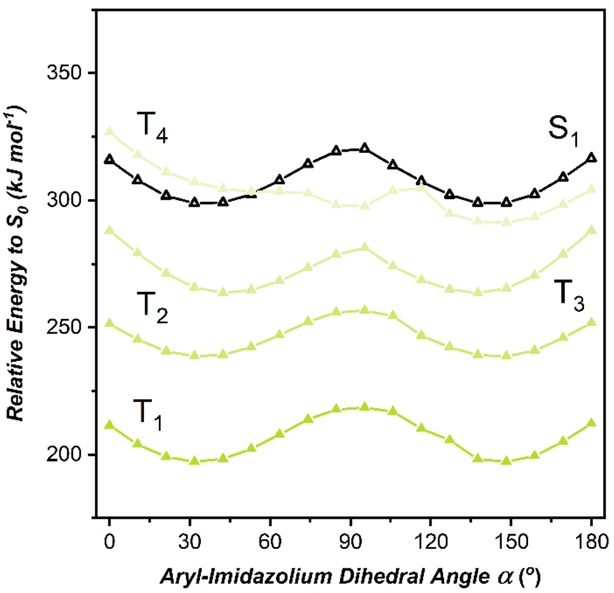

Figure S163. Relaxed surface scans of the dihedral angle D_α of **pseudo-*m*-NB** in S_1 and T_1-T_4 relative to S_0.

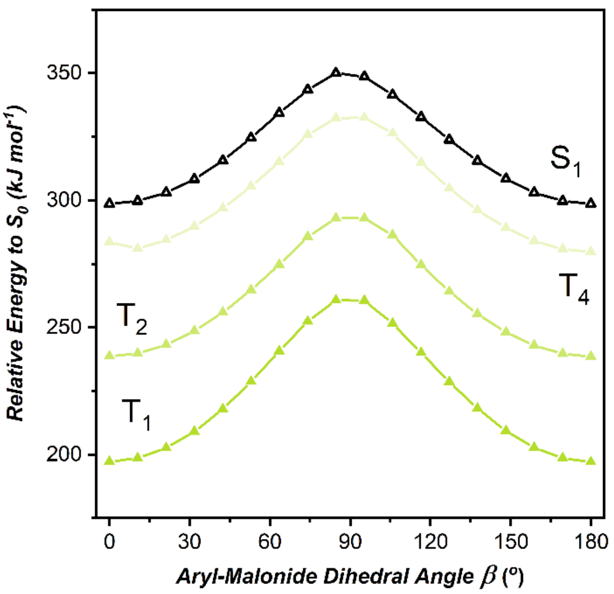

Figure S164. Relaxed surface scans of the dihedral angle D_β of **pseudo-*m*-NB** in S_1 and T_1-T_4 relative to S_0. T_3 did not converge.

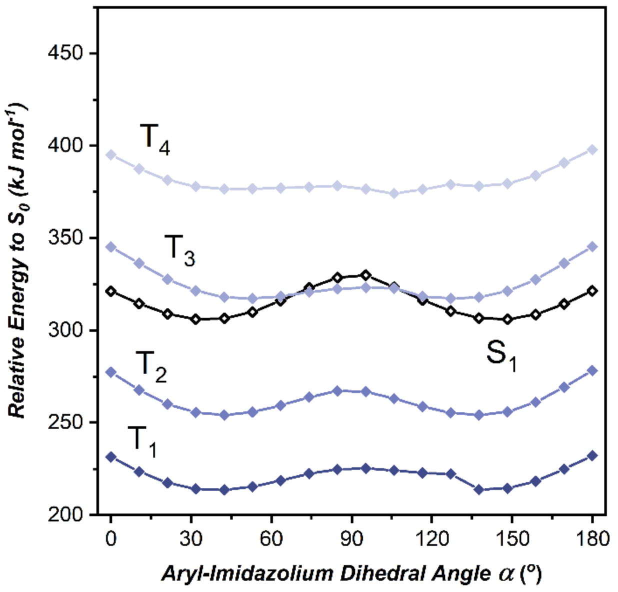

Figure S165. Relaxed surface scans of the dihedral angle D_α of **pseudo-*p*-NB** in S_1 and T_1-T_4 relative to S_0.

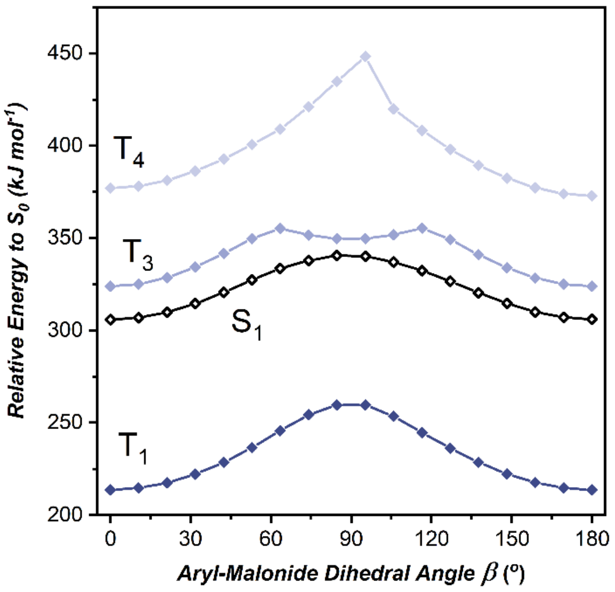

Figure S166. Relaxed surface scans of the dihedral angle D_β of **pseudo-*p*-NB** in S_1 and T_1-T_4 relative to S_0. T_2 did not converge.

Section S17 Fluorescence Lifetimes Fitting

Intensity-weighted lifetimes were calculated using the individual intensities (B_i) and the lifetimes (τ_i) of the components obtained from reconvolution fitting:

$$\tau_{avg} = \frac{\sum(B_i \times \tau_i^2)}{\sum(B_i \times \tau_i)}$$

Radiative rate constants (in seconds) were calculated using the intensity-weighted lifetimes:

$$k_r(s) = \frac{1\ 000\ 000\ 000}{\tau_{avg}}$$

Non-radiative rate constants (in seconds) were calculated using the fluorescence quantum yields and the radiative rate constants:

$$\Phi_F = \frac{k_r}{k_r + k_{nr}}$$

Table S34. Lifetimes and rate constants of compounds in various solvents.

Compound	Solvent	Intensity-weighted Lifetime, τ_{avg} (ns)	Radiative Rate Constant, k_r (s^{-1})	Non-radiative Rate Constant, k_{nr} (s^{-1})
***m*-PB**	acetone	1.31	7.66 E+08	5.11 E+10
	acetonitrile	1.36	7.36 E+08	5.24 E+10
	dimethylformamide	1.81	5.53 E+08	3.06 E+10
	dimethylsulfoxide	2.31	4.34 E+08	1.32 E+10
	formamide	1.77	5.66 E+08	3.26 E+10
	benzonitrile	3.21	3.12 E+08	1.87 E+10
***p*-PB**	acetone	0.01*	2.34 E+11	3.35 E+14
	acetonitrile	0.03*	3.16 E+10	5.32 E+13
	dimethylformamide	0.02*	5.84 E+10	1.64 E+13
	dimethylsulfoxide	0.04*	2.69 E+10	3.45 E+12
	formamide	0.06*	1.56 E+10	2.49 E+12
	benzonitrile	0.08*	1.33 E+10	3.15 E+12
**pseudo-*m*-NB**	acetone	2.06	4.86 E+08	1.30 E+10
	acetonitrile	2.06	4.78 E+08	2.02 E+10
	dimethylformamide	3.09	3.24 E+08	7.11 E+09
	dimethylsulfoxide	4.35	2.30 E+08	2.80 E+09
	formamide	2.40	4.17 E+08	7.68 E+09
	benzonitrile	3.55	2.82 E+08	6.03 E+09
**pseudo-*p*-NB**	acetone	2.51	3.98 E+08	5.15 E+08
	acetonitrile	2.83	3.54 E+08	4.00 E+08
	dimethylformamide	3.31	3.02 E+08	1.96 E+08
	dimethylsulfoxide	3.91	2.56 E+08	8.54 E+07
	formamide	4.17	2.40 E+08	7.57 E+07
	benzonitrile	3.76	2.66 E+08	9.88 E+07

*lifetimes of ***p*-PB** emission were too short to accurately measure with our instrumentation.

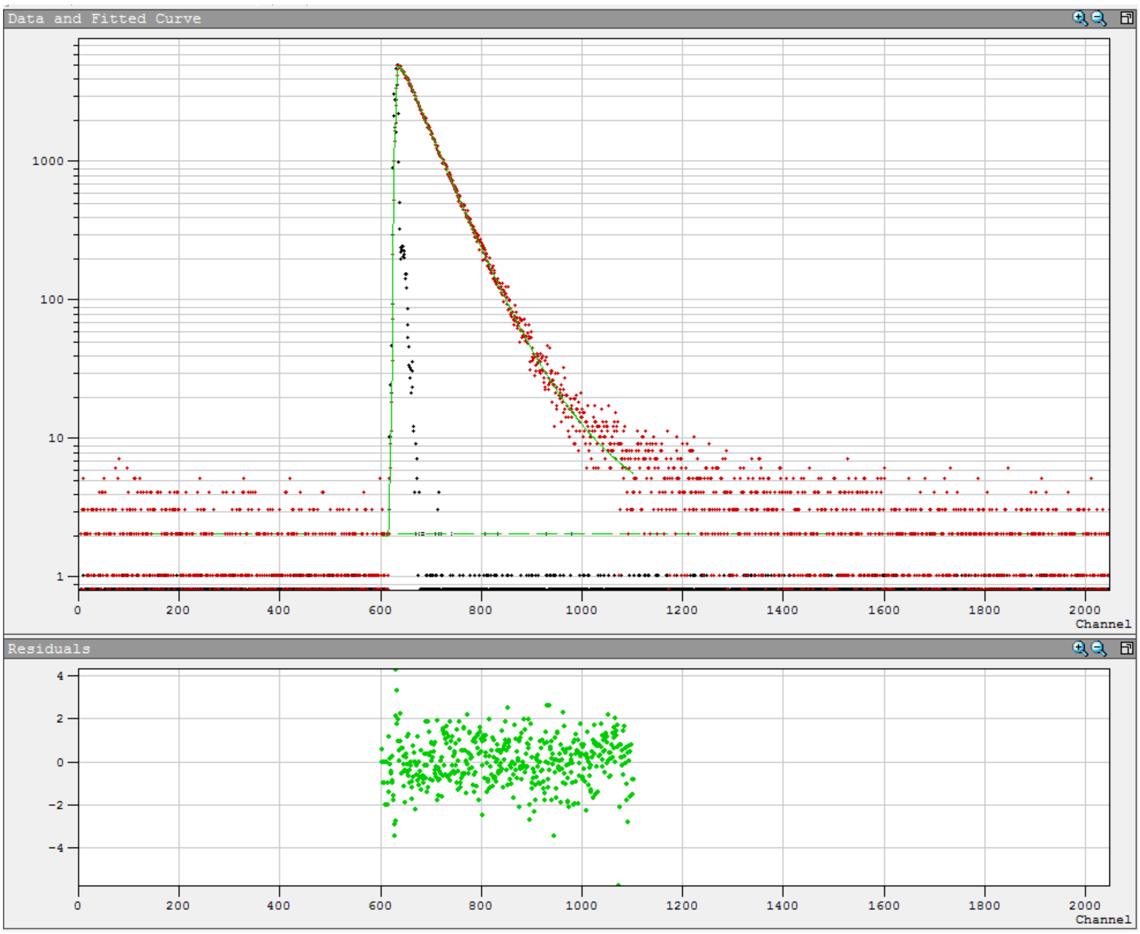

Fluorescence decay trace and reconvolution best fit of ***m*-PB** in acetone.

Reconvolution parameters of ***m*-PB** in acetone.

PhenZwit Ace Decay 50ns.FL

❖ **Exponential Components Analysis (Reconvolution)**

Fitting range [600; 1100] channels

χ^2 1,247

	B_i	ΔB_i	f_i (%)	Δf_i (%)	τ_i (ns)	$\Delta\tau_i$ (ns)
**intensity-weighted lifetime**					**1,31**	
1	0,1681	0,001	94,306	1,054	1,202	0,006
2	0,0041	0,0012	5,694	1,755	3,005	0,034

		Probability 60%		Probability 90%	
	τ_i (ns)	$Conf_{low}$	$Conf_{high}$	$Conf_{low}$	$Conf_{high}$
1	1,202	1,166	1,225	1,147	1,232
2	3,005	2,284	4,358	2,069	5,237

Shift 0,002 (± 0,026 ns)

Decay Background 2.000 fixed (± 0)

IRF background 0,1

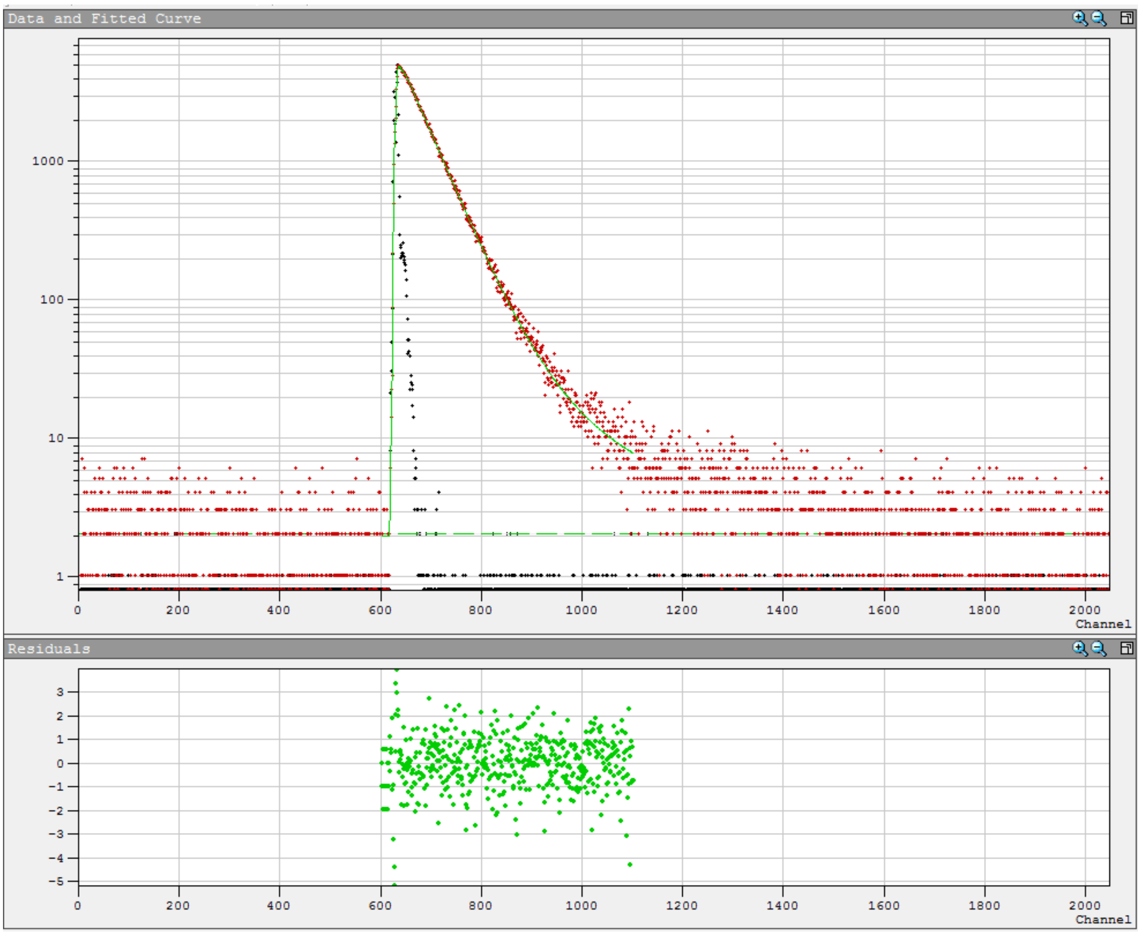

Fluorescence decay trace and reconvolution best fit of ***m*-PB** in acetonitrile.

Reconvolution parameters of ***m*-PB** in acetonitrile.

PhenZwit ACN Decay 50ns.FL

❖ **Exponential Components Analysis (Reconvolution)**

Fitting range [600; 1100] channels

χ^2 1,284

	B_i	ΔB_i	f_i (%)	Δf_i (%)	τ_i (ns)	$\Delta\tau_i$ (ns)
**intensity-weighted lifetime**					**1,36**	
1	0,1721	0,0006	95,954	0,673	1,23	0,005
2	0,002	0,0005	4,046	1,125	4,441	0,03

		Probability 60%		Probability 90%	
	τ_i (ns)	Conf $_{low}$	Conf $_{high}$	Conf $_{low}$	Conf $_{high}$
1	1,23	1,21	1,248	1,2	1,255
2	4,441	3,321	6,848	3,01	+inf.

Shift	0,0004	(±	0,029	ns)
Decay Background	2.000 fixed	(±	0	)
IRF background	0,1			

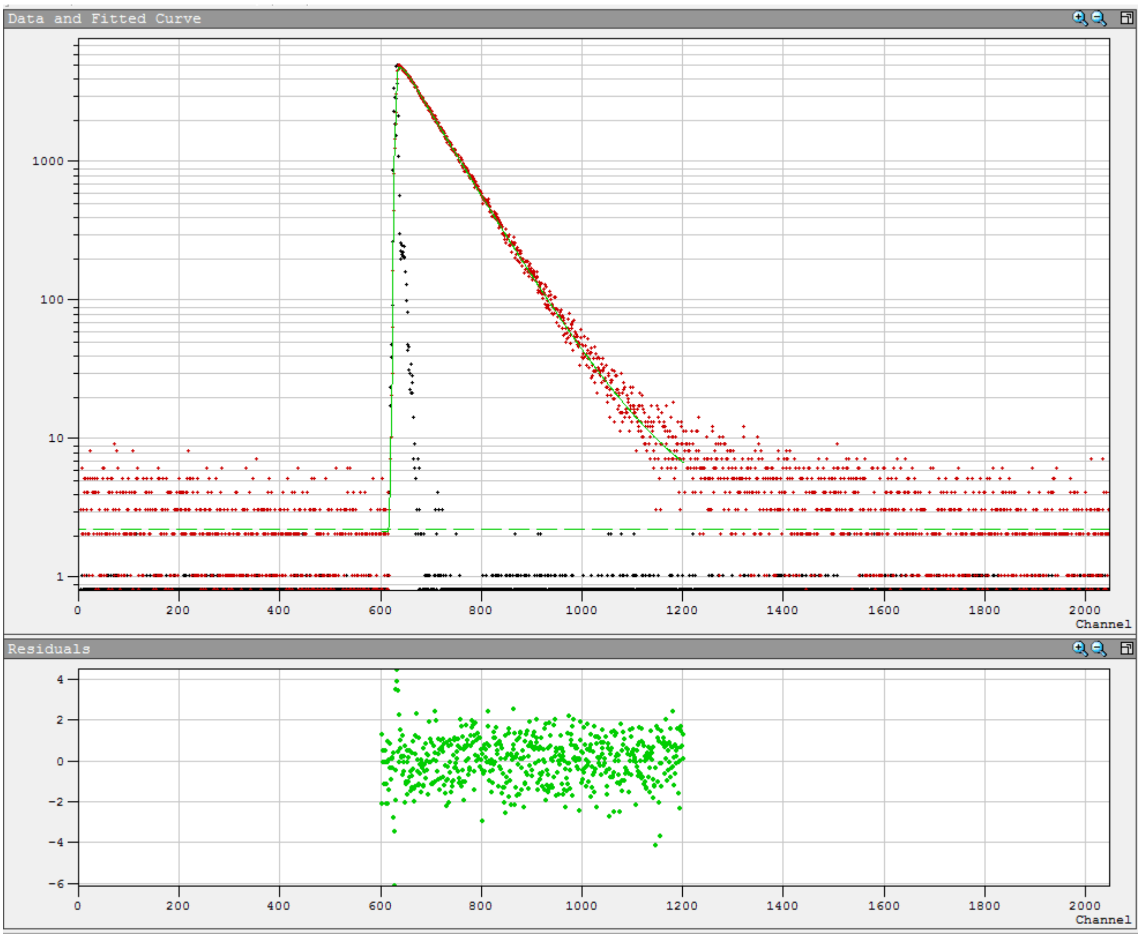

Fluorescence decay trace and reconvolution best fit of ***m*-PB** in dimethylformamide.

Reconvolution parameters of ***m*-PB** in dimethylformamide.

PhenZwit DMF Decay 50ns.FL

❖ Exponential Components Analysis (Reconvolution)

Fitting range [600; 1200] channels

χ^2 1,358

	B_i	ΔB_i	f_i (%)	Δf_i (%)	τ_i (ns)	$\Delta\tau_i$ (ns)
**intensity-weighted lifeitme**					1,81	
1	0,1542	0,0044	93,934	3,169	1,708	0,009
2	0,0051	0,0046	6,066	5,694	3,36	0,074

		Probability 60%		Probability 90%	
	τ_i (ns)	Conf $_{low}$	Conf $_{high}$	Conf $_{low}$	Conf $_{high}$
1	1,708	-inf.	1,75	-inf.	1,761
2	3,36	-inf.	+inf.	-inf.	+inf.

Shift	0,009	(±	0,03 ns)
Decay Background	2,136	(±	0,882)
IRF background	0,2		

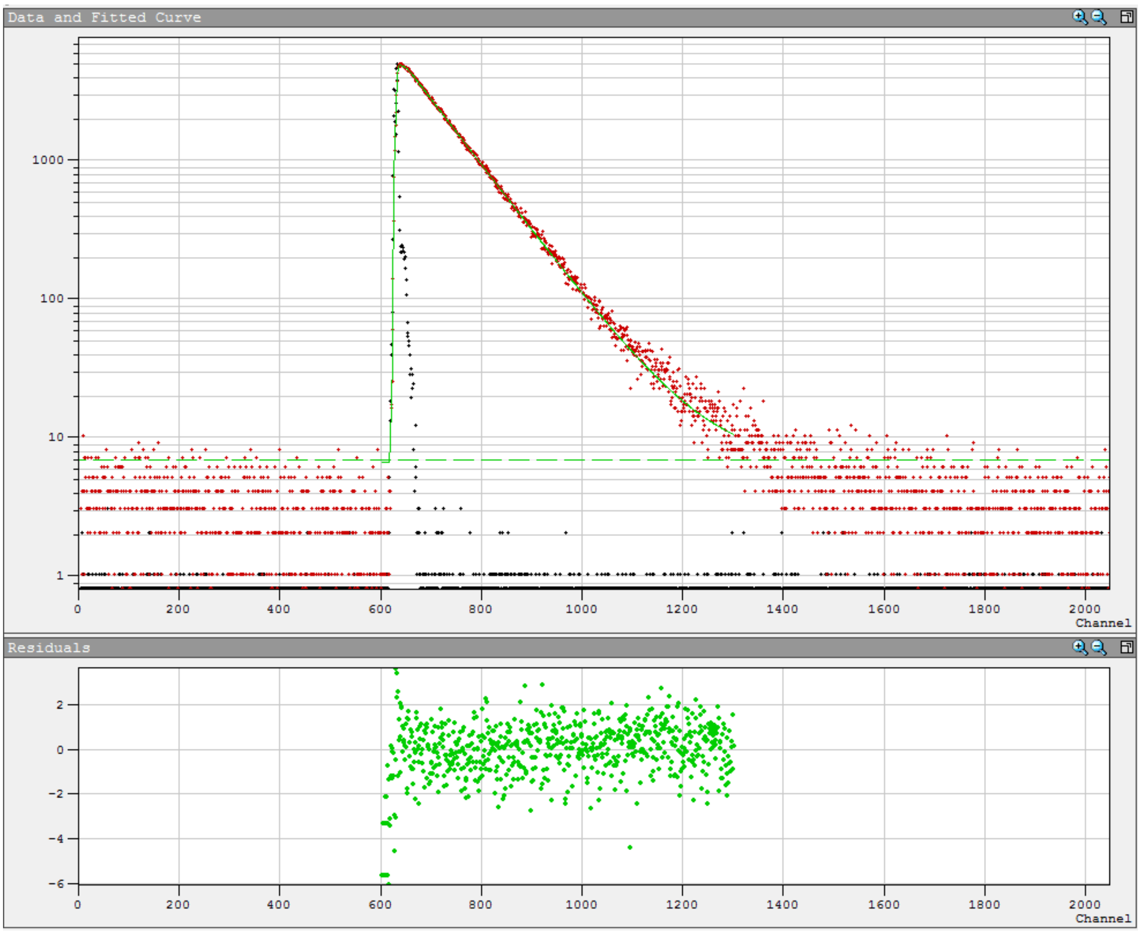

Fluorescence decay trace and reconvolution best fit of ***m*-PB** in dimethylsulfoxide.

Reconvolution parameters of ***m*-PB** in dimethylsulfoxide.

PhenZwit DMSO Decay 50ns.FL

❖ Exponential Components Analysis (Reconvolution)

Fitting range [600; 1300] channels

χ^2 1,172

	B_i	ΔB_i	f_i (%)	Δf_i (%)	τ_i (ns)	$\Delta\tau_i$ (ns)
**intensity-weighted lifetime**					**2,31**	
1	0,159	0,0007	97,547	0,583	2,2	0,003
2	0,0014	0,0009	2,453	1,604	6,428	0,042

		Probability 60%		Probability 90%	
	τ_i (ns)	Conf $_{low}$	Conf $_{high}$	Conf $_{low}$	Conf $_{high}$
1	2,2	2,14	2,226	2,09	2,233
2	6,428	3,677	+inf.	-inf.	+inf.

Shift	0,012	(±	0,025	ns)
Decay Background	3.000 fixe(	(±	0	)
IRF background	0,2			

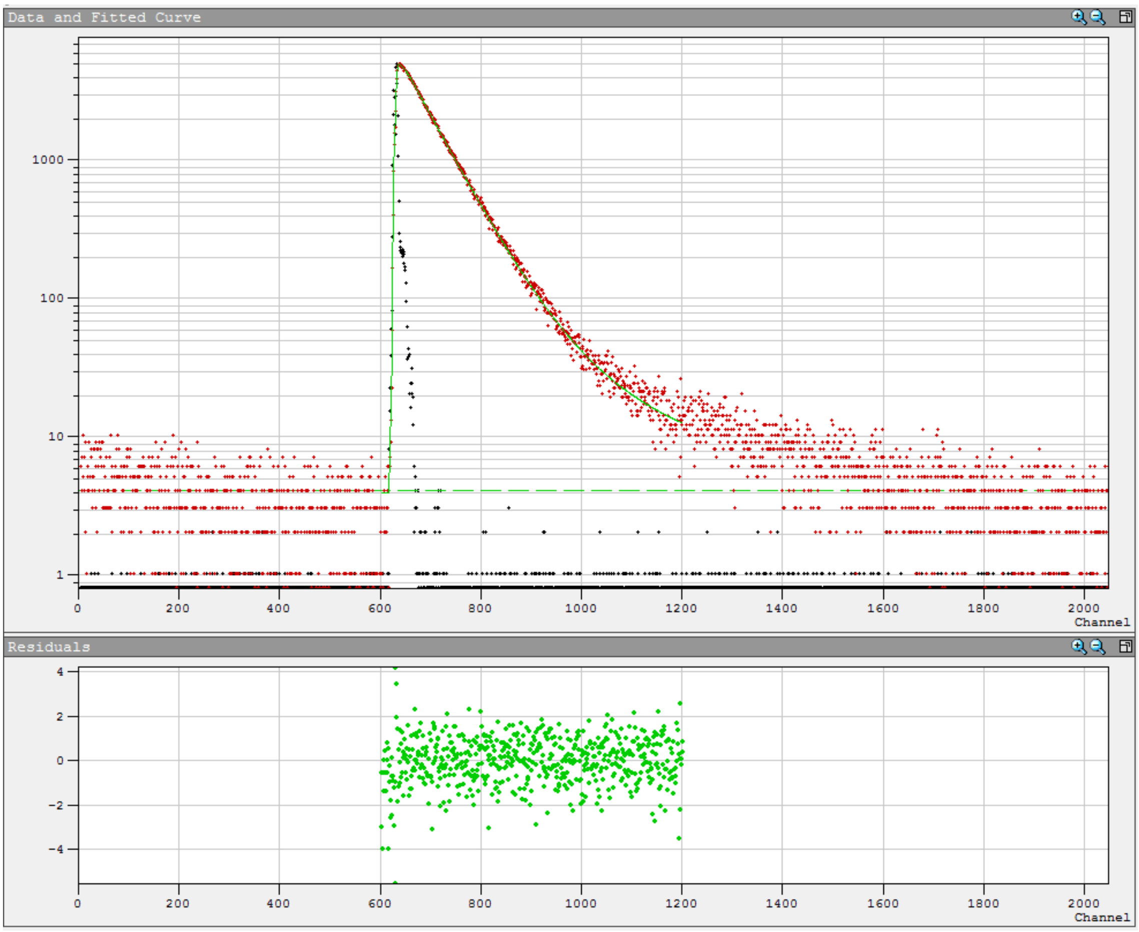

Fluorescence decay trace and reconvolution best fit of ***m*-PB** in formamide.

Reconvolution parameters of ***m*-PB** in formamide.

PhenZwit formamide Decay 50ns.FL

❖ **Exponential Components Analysis (Reconvolution)**

Fitting range [600; 1200] channels

χ^2 1,216

	B_i	ΔB_i	f_i (%)	Δf_i (%)	τ_i (ns)	$\Delta\tau_i$ (ns)
**intensity-weighted lifetime**					**1,77**	
1	0,1657	0,0005	93,944	0,508	1,545	0,003
2	0,0032	0,0006	6,056	1,117	5,191	0,016

		Probability 60%		Probability 90%	
	τ_i (ns)	Conf $_{low}$	Conf $_{high}$	Conf $_{low}$	Conf $_{high}$
1	1,545	1,521	1,564	1,511	1,572
2	5,191	4,302	6,747	3,989	7,608

Shift	0,012	(±	0,025	ns)
Decay Background	4.000 fixe(	(±	0	)
IRF background	0,1			

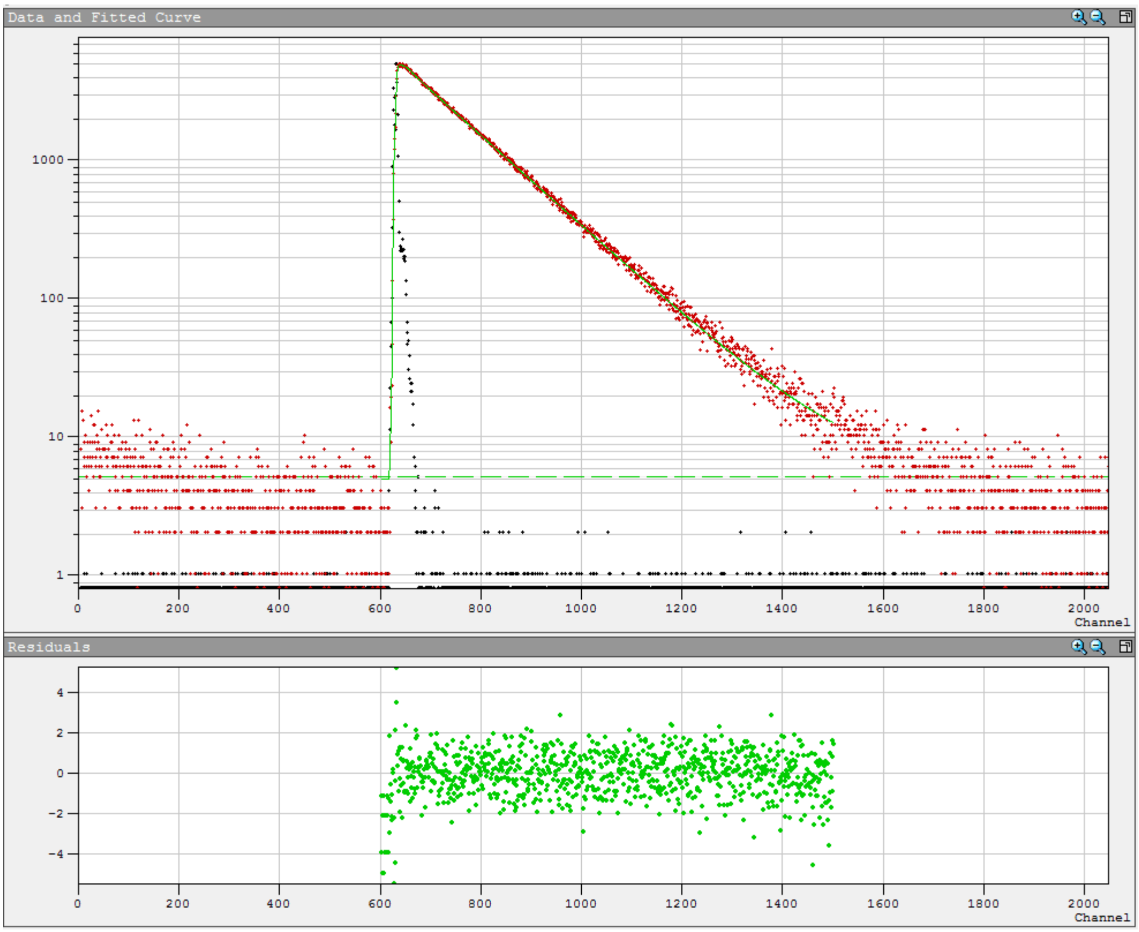

Fluorescence decay trace and reconvolution best fit of ***m*-PB** in benzonitrile.

Reconvolution parameters of ***m*-PB** in benzonitrile.

PhenZwit PhCN Decay 50ns.FL

❖ Exponential Components Analysis (Reconvolution)

Fitting range [600; 1500] channels

χ^2 1,305

	B_i	ΔB_i	f_i (%)	Δf_i (%)	τ_i (ns)	$\Delta\tau_i$ (ns)
**intensity-weighted lifetime**					**3,21**	
1	0,0157	0,0015	1,301	1,267	0,393	0,346
2	0,1437	0,0004	98,699	0,313	3,247	0,0005

		Probability 60%		Probability 90%	
	τ_i (ns)	Conf $_{low}$	Conf $_{high}$	Conf $_{low}$	Conf $_{high}$
1	0,393	0,27	0,567	0,233	0,651
2	3,247	3,241	3,256	3,235	3,263

Shift	0,015	(±	0,032	ns)
Decay Background	5.000 fixe(	(±	0	)
IRF background	0,1			

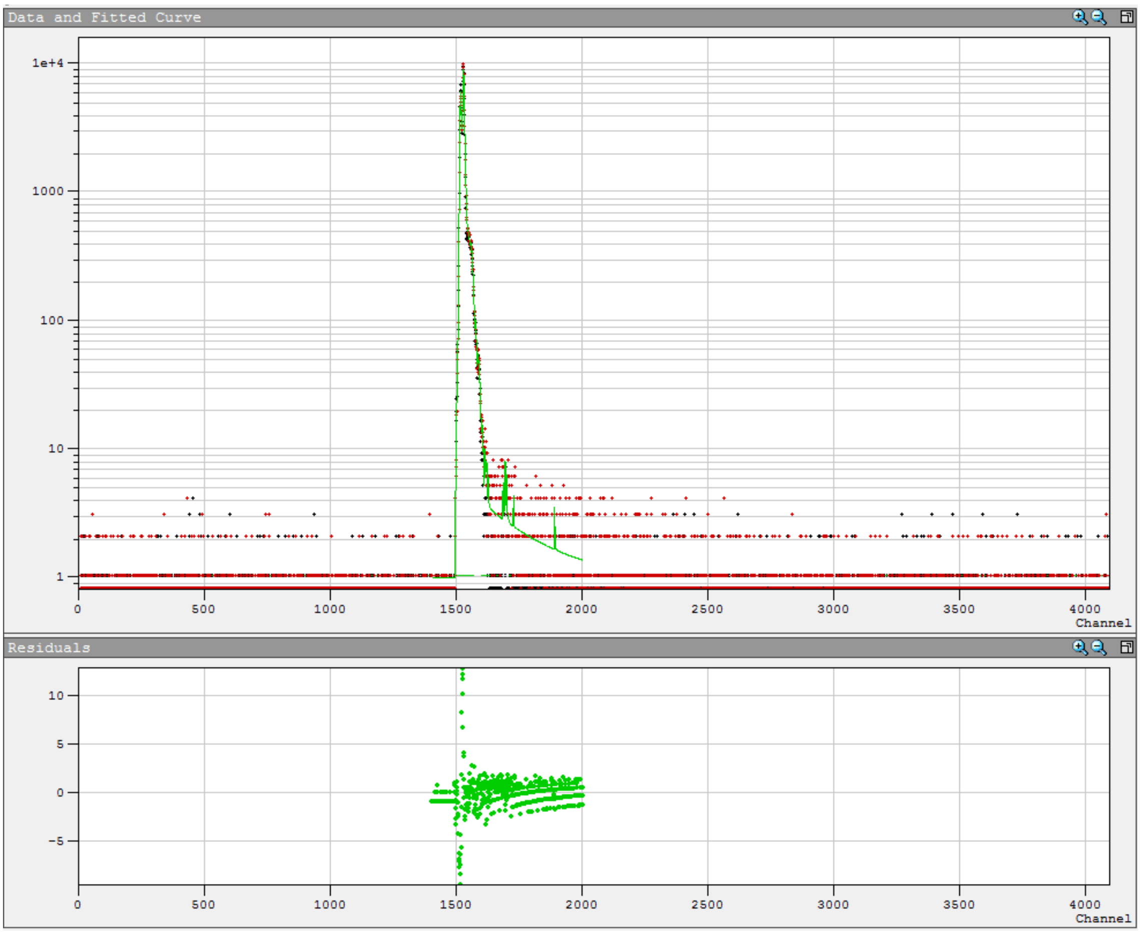

Fluorescence decay trace and reconvolution best fit of ***p*-PB** in acetone.

Reconvolution parameters of ***p*-PB** in acetone.

PhenQuin Ace Decay 50ns.FL

❖ **Exponential Components Analysis (Reconvolution)**

Fitting range [1400; 2000] channels

χ^2 3,131

	B_i	ΔB_i	f_i (%)	Δf_i (%)	τ_i (ns)	$\Delta\tau_i$ (ns)
**intensity-weighted lifetime**					**0,01**	
1	83,3526	393,6478	98,438	1,50E+07	0,003	384,129
2	0,0664	0,0135	1,529	54,817	0,05	1,795
3	2,90E-05	2,20E-05	0,032	0,029	2,401	0,372

		Probability 60%		Probability 90%	
	τ_i (ns)	Conf $_{low}$	Conf $_{high}$	Conf $_{low}$	Conf $_{high}$
1	0,003	-inf.	+inf.	-inf.	+inf.
2	0,05	0,045	0,056	0,042	0,06
3	2,401	-inf.	+inf.	-inf.	+inf.

Shift -0,002 (± 0,046 ns)

Decay Background 1.000 fixed (± 0)

IRF background 0,3

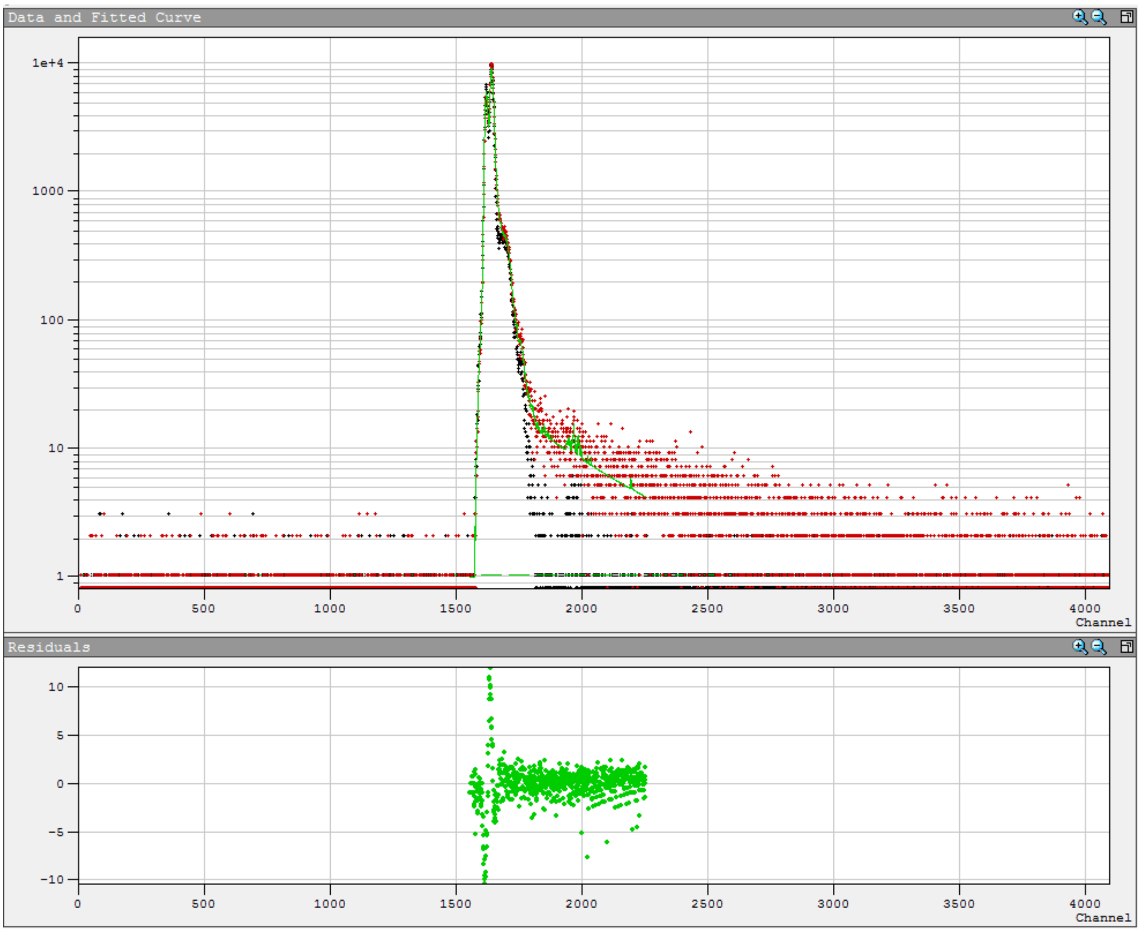

Fluorescence decay trace and reconvolution best fit of ***p*-PB** in acetonitrile.

Reconvolution parameters of ***p*-PB** in acetonitrile.

PhenQuin ACN Decay 20ns.FL

❖ Exponential Components Analysis (Reconvolution)

Fitting range [1550; 2250] channels

χ^2 4,729

	B_i	ΔB_i	f_i (%)	Δf_i (%)	τ_i (ns)	$\Delta\tau_i$ (ns)
**intensity-weighted lifetime**					**0,03**	
1	2,3706	3,6071	82,459	5,90E+06	0,003	241,248
2	0,0391	0,0088	16,233	1056,464	0,04	2,602
3	8,30E-05	2,10E-05	1,308	0,478	1,531	0,175

		Probability 60%		Probability 90%	
	τ_i (ns)	Conf $_{low}$	Conf $_{high}$	Conf $_{low}$	Conf $_{high}$
1	0,003	-inf.	+inf.	-inf.	+inf.
2	0,04	0,036	0,047	0,034	0,052
3	1,531	1,057	2,527	0,965	+inf.

Shift	-0,003	(±	0,369	ns)
Decay Background	1.000 fixed	(±	0	)
IRF background	0,2			

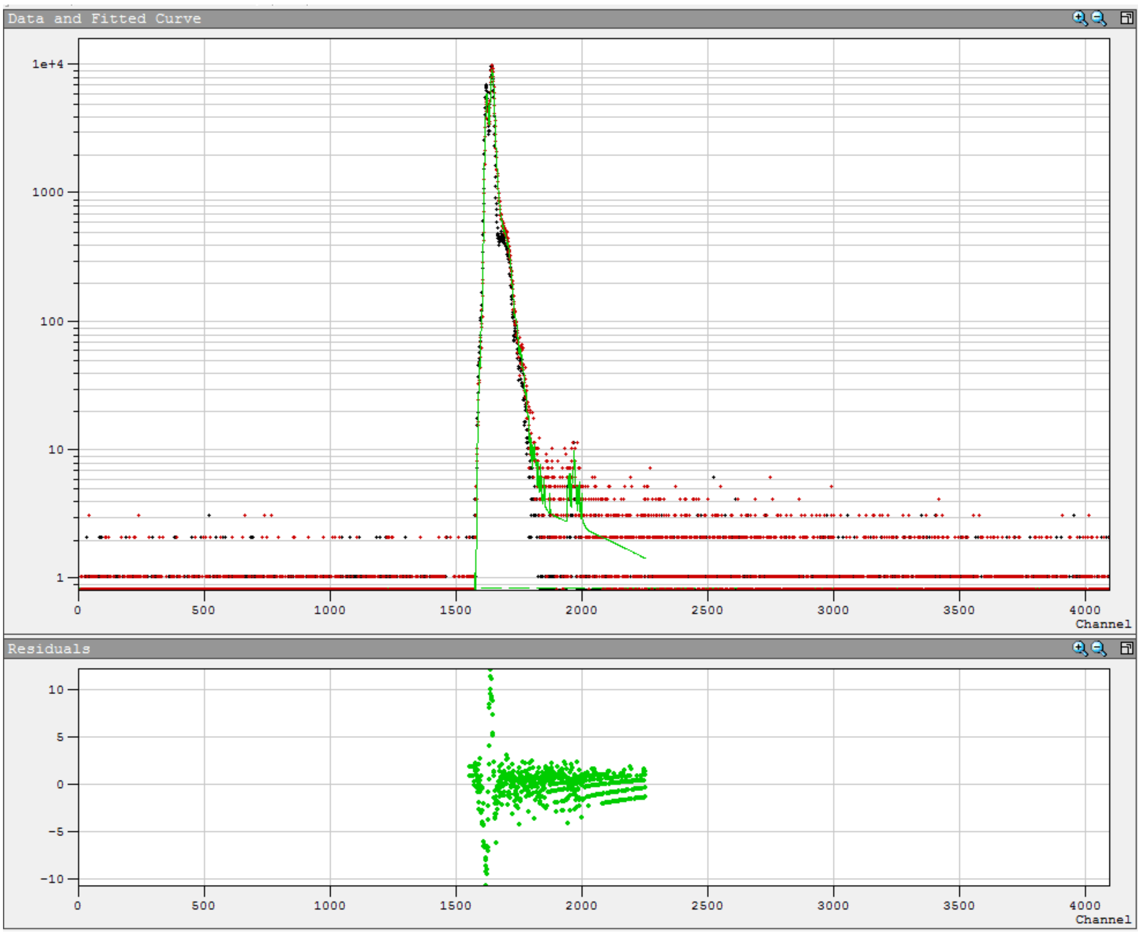

Fluorescence decay trace and reconvolution best fit of ***p*-PB** in dimethylformamide.

Reconvolution parameters of ***p*-PB** in dimethylformamide.

PhenQuin DMF Decay 20ns.FL

❖ Exponential Components Analysis (Reconvolution)

Fitting range [1550; 2250] channels

χ^2 4,84

	B_i	ΔB_i	f_i (%)	Δf_i (%)	τ_i (ns)	$\Delta\tau_i$ (ns)
**intensity-weighted lifetime**					**0,02**	
1	1,9137	1,3297	76,563	2,30E+06	0,004	108,813
2	0,0441	0,0062	23,165	719,794	0,047	1,444
3	1,50E-05	1,80E-05	0,272	0,466	1,61	0,823

		Probability 60%		Probability 90%	
	τ_i (ns)	Conf $_{low}$	Conf $_{high}$	Conf $_{low}$	Conf $_{high}$
1	0,004	-inf.	0,006	-inf.	+inf.
2	0,047	0,045	0,052	0,043	0,053
3	1,61	-inf.	+inf.	-inf.	+inf.

Shift 0,004 (± 0,182 ns)

Decay Background 1.000 fixed (± 0)

IRF background 0,2

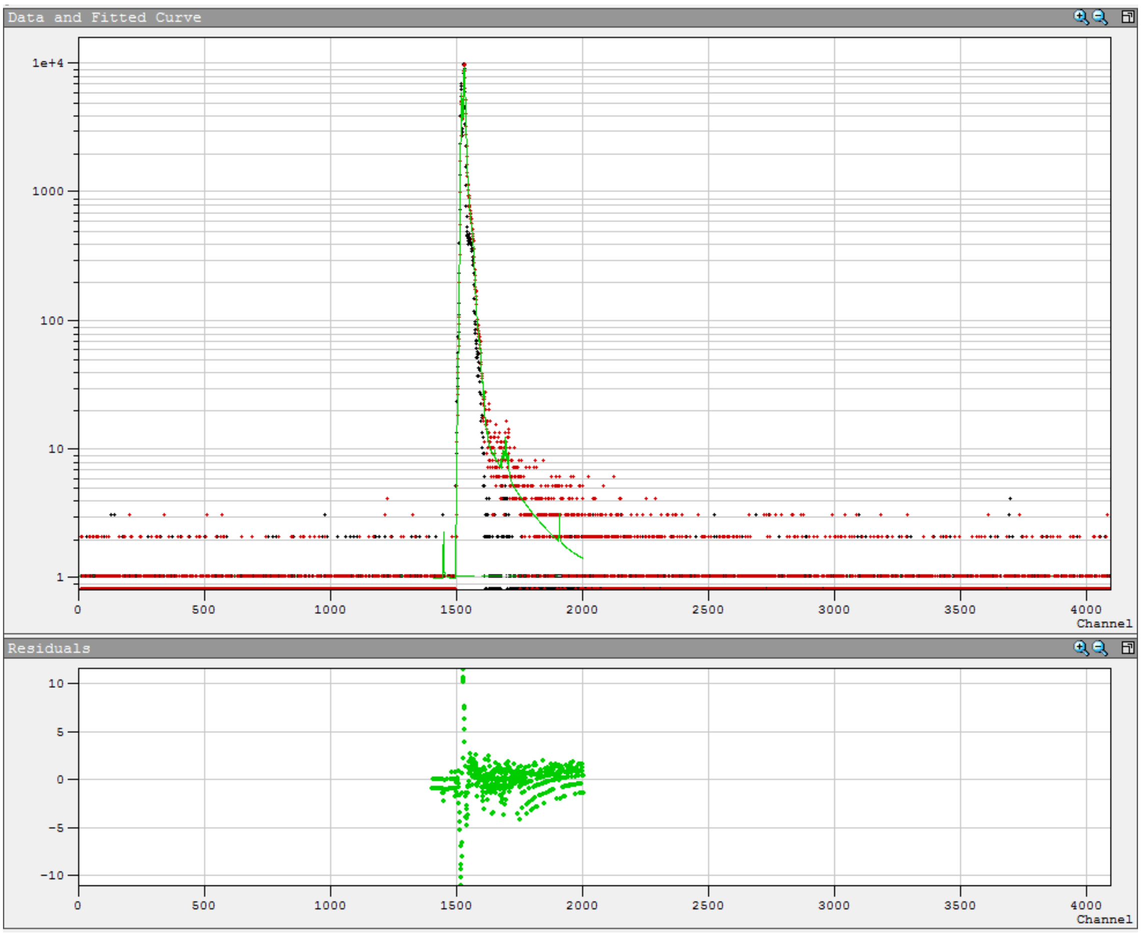

Fluorescence decay trace and reconvolution best fit of ***p*-PB** in dimethylsulfoxide.

Reconvolution parameters of ***p*-PB** in dimethylsulfoxide.

PhenQuin DMSO Decay 50ns.FL

❖ **Exponential Components Analysis (Reconvolution)**

Fitting range [1400; 2000] channels

χ^2 3,548

	B_i	ΔB_i	f_i (%)	Δf_i (%)	τ_i (ns)	$\Delta\tau_i$ (ns)
**intensity-weighted lifetime**					**0,04**	
1	2,1023	1,3458	71,252	3,70E+05	0,008	43,135
2	0,0878	0,0105	27,855	267,688	0,078	0,737
3	0,0001	4,70E-05	0,893	0,39	1,474	0,174

		Probability 60%		Probability 90%	
	τ_i (ns)	Conf $_{low}$	Conf $_{high}$	Conf $_{low}$	Conf $_{high}$
1	0,008	-inf.	+inf.	-inf.	+inf.
2	0,078	0,073	0,093	0,071	0,098
3	1,474	0,838	2,69	-inf.	+inf.

Shift	0,009	(±	0,117	ns)
Decay Background	1.000 fixed	(±	0	)
IRF background	0,2			

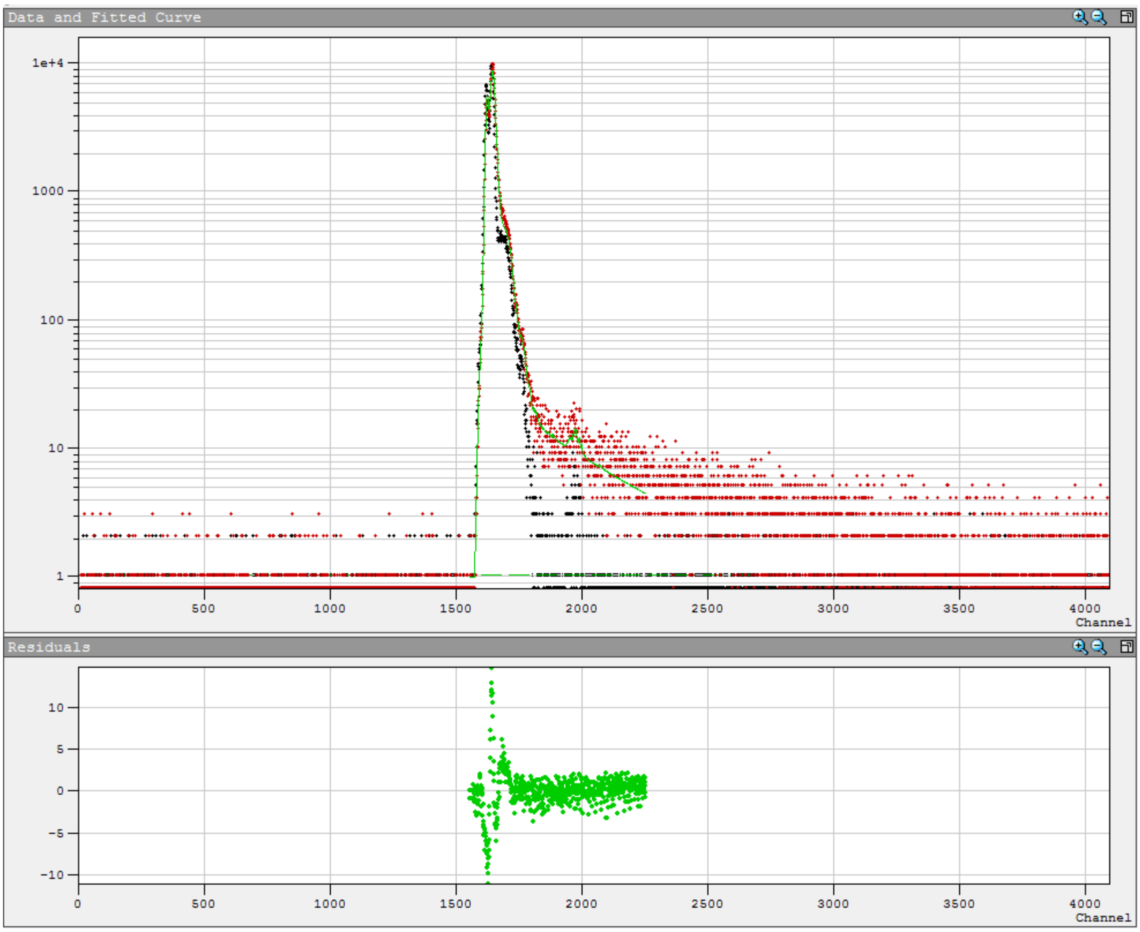

Fluorescence decay trace and reconvolution best fit of ***p*-PB** in formamide.

Reconvolution parameters of ***p*-PB** in formamide.

PhenQuin PhCN Decay 50ns.FL

❖ **Exponential Components Analysis (Reconvolution)**

Fitting range [200; 1000] channels

χ^2 2,513

	B_i	ΔB_i	f_i (%)	Δf_i (%)	τ_i (ns)	$\Delta\tau_i$ (ns)
**intensity-weighted lifetime**					**0,08**	
1	13,3473	13,904	78,665	1,70E+06	0,004	82,34
2	0,0645	0,0035	11,059	73,034	0,109	0,714
3	0,0121	0,0022	7,212	6,87	0,379	0,292
4	0,0014	0,0003	3,063	0,773	1,35	0,059

		Probability 60%		Probability 90%	
	τ_i (ns)	Conf $_{low}$	Conf $_{high}$	Conf $_{low}$	Conf $_{high}$
1	0,004	-inf.	+inf.	-inf.	+inf.
2	0,109	0,083	0,126	0,075	0,133
3	0,379	0,274	0,53	0,256	0,574
4	1,35	1,103	1,842	1,06	2,055

Shift 0,02 (± 0,048 ns)

Decay Background 1.000 fixed (± 0)

IRF background 0,3

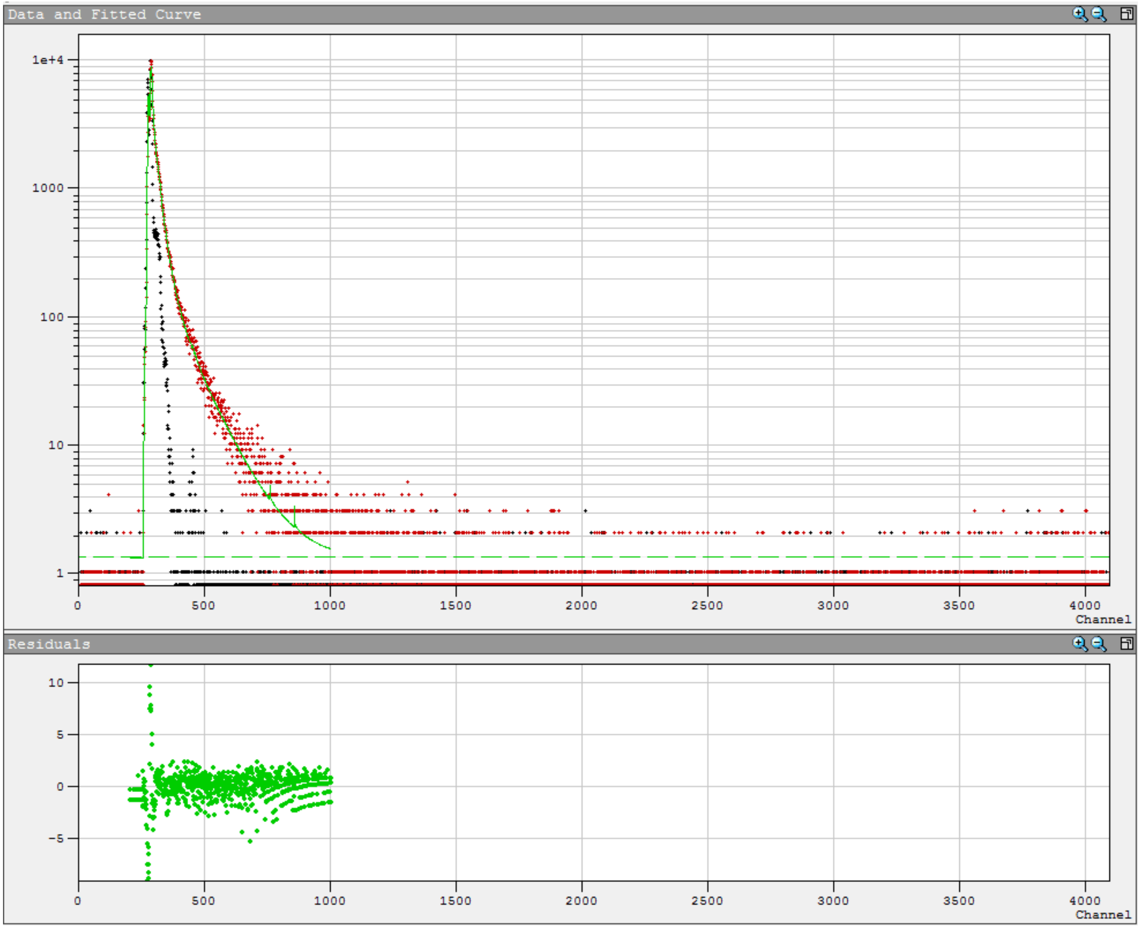

Fluorescence decay trace and reconvolution best fit of ***p*-PB** in benzonitrile.

Reconvolution parameters of ***p*-PB** in benzonitrile.

PhenQuin formamide Decay 20ns.FL

❖ Exponential Components Analysis (Reconvolution)

Fitting range [1550; 2250] channels

χ^2 5,483

	B_i	ΔB_i	f_i (%)	Δf_i (%)	τ_i (ns)	$\Delta\tau_i$ (ns)
**intensity-weighted lifetime**					**0,06**	
1	0,0067	0,7223	3,421	2,00E+05	0,033	2254,043
2	0,1858	0,7225	94,535	2,30E+05	0,033	80,476
3	8,60E-05	3,10E-05	2,044	1,055	1,544	0,243

		Probability 60%		Probability 90%	
	τ_i (ns)	Conf $_{low}$	Conf $_{high}$	Conf $_{low}$	Conf $_{high}$
1	0,033	-inf.	+inf.	-inf.	+inf.
2	0,033	-inf.	0,037	-inf.	0,038
3	1,544	1,004	2,874	0,888	+inf.

Shift 0,0008 (± 0,162 ns)

Decay Background 1.000 fixed (± 0)

IRF background 0,2

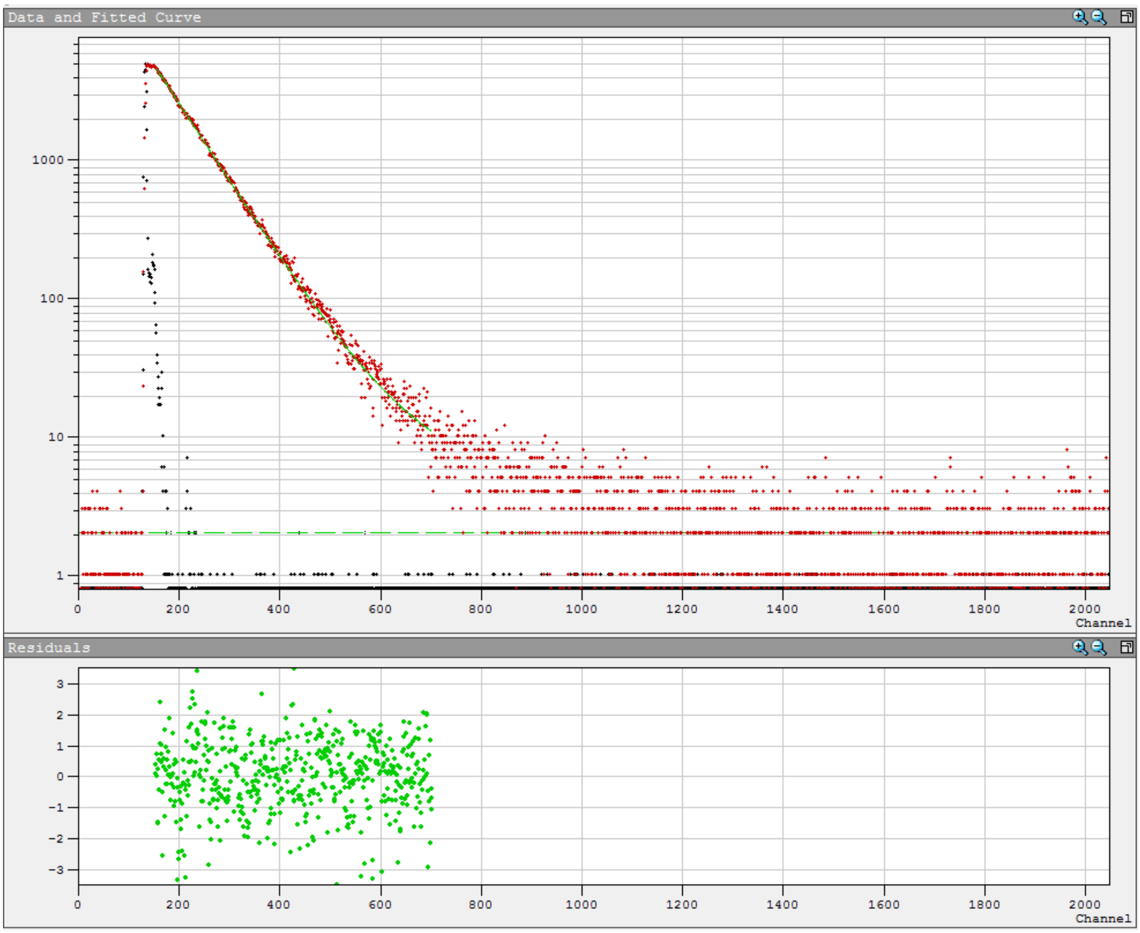

Fluorescence decay trace and reconvolution best fit of **pseudo-*m*-NB** in acetone.

Reconvolution parameters of **pseudo-*m*-NB** in acetone.

NaphZwit Ace Decay 50ns.FL

❖ **Exponential Components Analysis (Reconvolution)**

Fitting range [100; 700] channels

χ^2 1,322

	B_i	ΔB_i	f_i (%)	Δf_i (%)	τ_i (ns)	$\Delta\tau_i$ (ns)
					**2,06**	
1	0,2349	0,0006	98,165	0,38	1,888	0,003
2	0,0008	0,0005	1,835	1,196	10,752	0,052

		Probability 60%		Probability 90%	
	τ_i (ns)	Conf $_{low}$	Conf $_{high}$	Conf $_{low}$	Conf $_{high}$
1	1,888	1,859	1,906	1,842	1,909
2	10,752	-inf.	+inf.	-inf.	+inf.

Shift	0,008	(±	0,023	ns)
Decay Background	2.000 fixed	(±	0	)
IRF background	0			

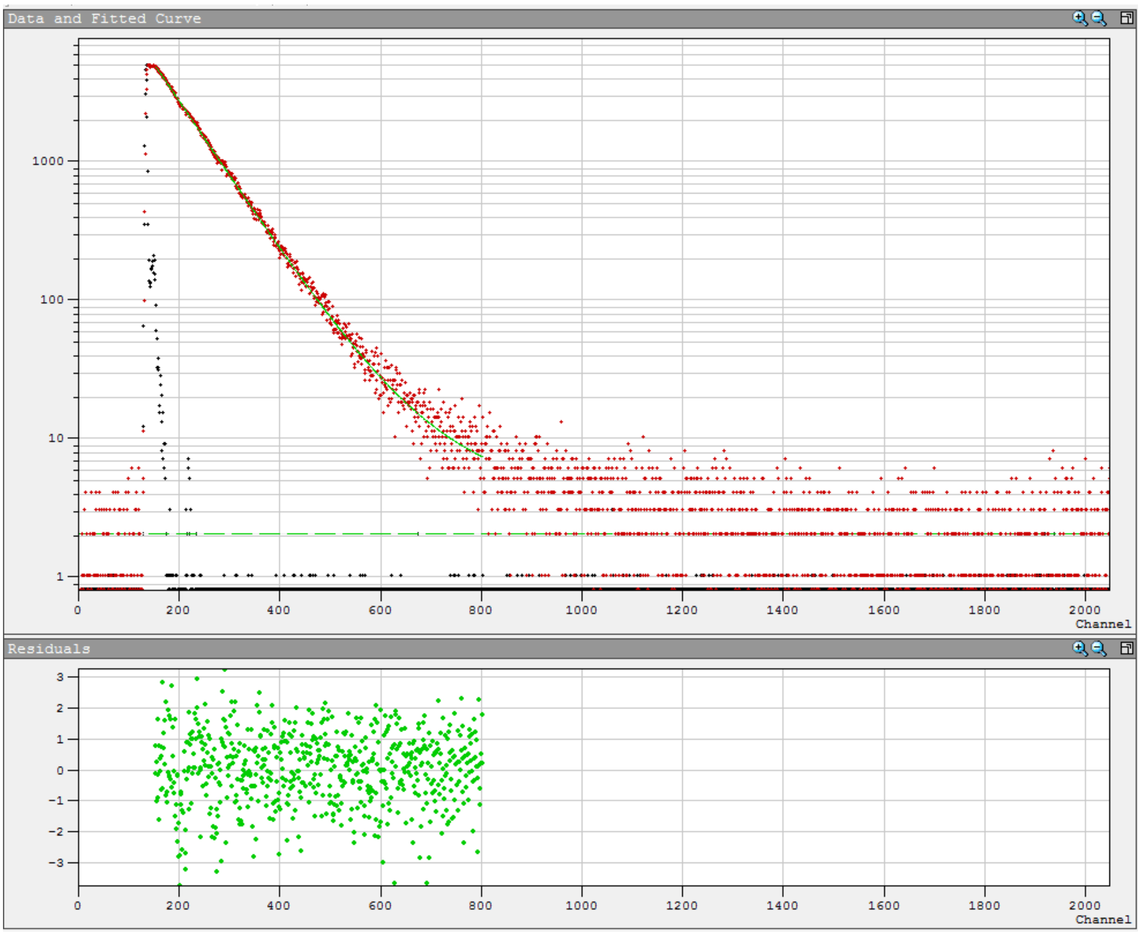

Fluorescence decay trace and reconvolution best fit of **pseudo-*m*-NB** in acetonitrile.

Reconvolution parameters of **pseudo-*m*-NB** in acetonitrile.

NaphZwit ACN Decay 50ns.FL

❖ **Exponential Components Analysis (Reconvolution)**

Fitting range [100; 800] channels

χ^2 1,424

	B_i	ΔB_i	f_i (%)	Δf_i (%)	τ_i (ns)	$\Delta\tau_i$ (ns)
					**2,09**	
1	0,2497	0,0006	98,19	0,367	1,961	0,002
2	0,0009	0,0005	1,81	0,959	9,569	0,038

		Probability 60%		Probability 90%	
	τ_i (ns)	Conf $_{low}$	Conf $_{high}$	Conf $_{low}$	Conf $_{high}$
1	1,961	1,935	1,98	1,92	1,984
2	9,569	5,073	+inf.	-inf.	+inf.

Shift	0,003	(±	0,025	ns)
Decay Background	2.000 fixed	(±	0	)
IRF background	0,1			

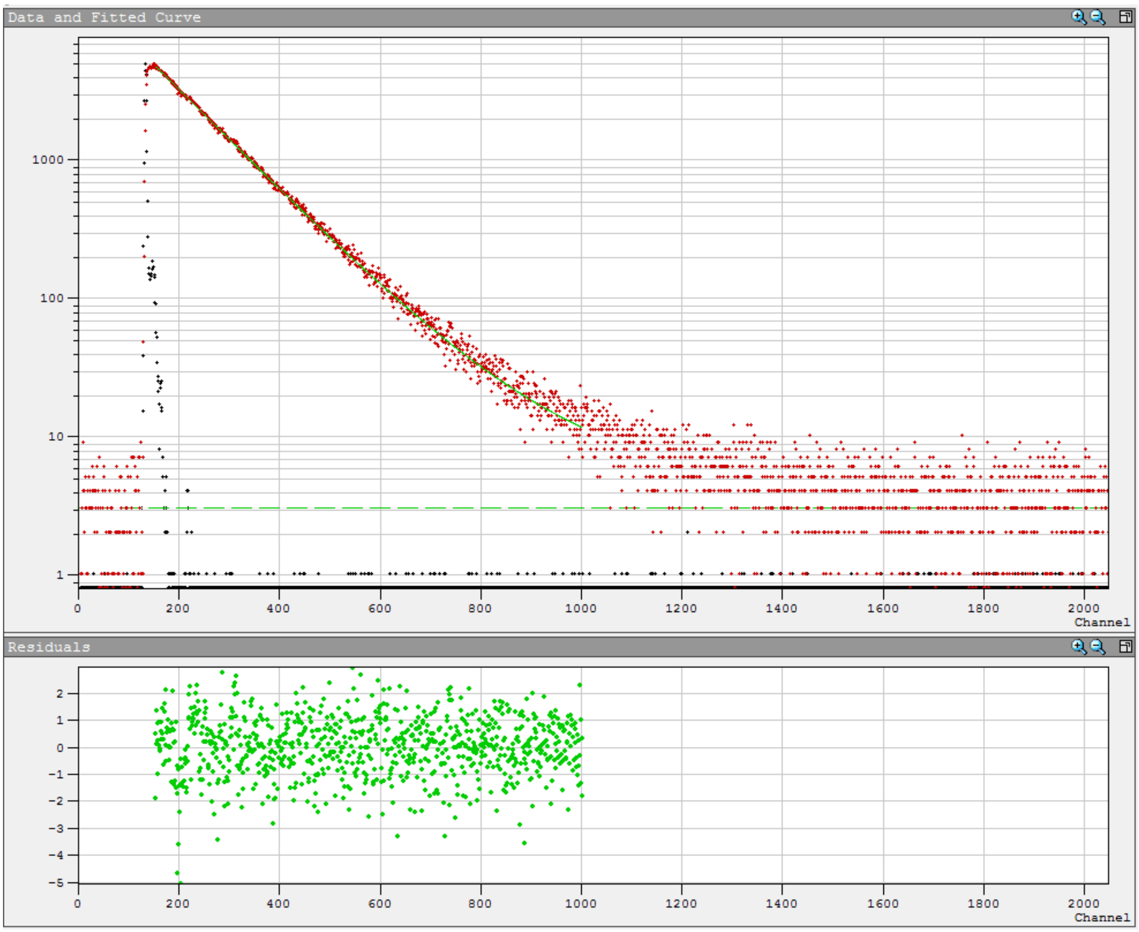

Fluorescence decay trace and reconvolution best fit of **pseudo-*m*-NB** in dimethylformamide.

Reconvolution parameters of **pseudo-*m*-NB** in dimethylformamide.

NaphZwit DMF Decay 50ns.FL

❖ Exponential Components Analysis (Reconvolution)

Fitting range [100; 1000] channels

χ^2 1,381

	B_i	ΔB_i	f_i (%)	Δf_i (%)	τ_i (ns)	$\Delta\tau_i$ (ns)
					**3,09**	
1	0,2257	0,0009	97,322	0,459	2,907	0,002
2	0,0018	0,0011	2,678	1,549	9,783	0,029

		Probability 60%		Probability 90%	
	τ_i (ns)	$Conf_{low}$	$Conf_{high}$	$Conf_{low}$	$Conf_{high}$
1	2,907	2,852	2,939	2,82	2,947
2	9,783	5,867	+inf.	5,158	+inf.

Shift 0,008 (± 0,025 ns)

Decay Background 3.000 fixed (± 0)

IRF background 0,1

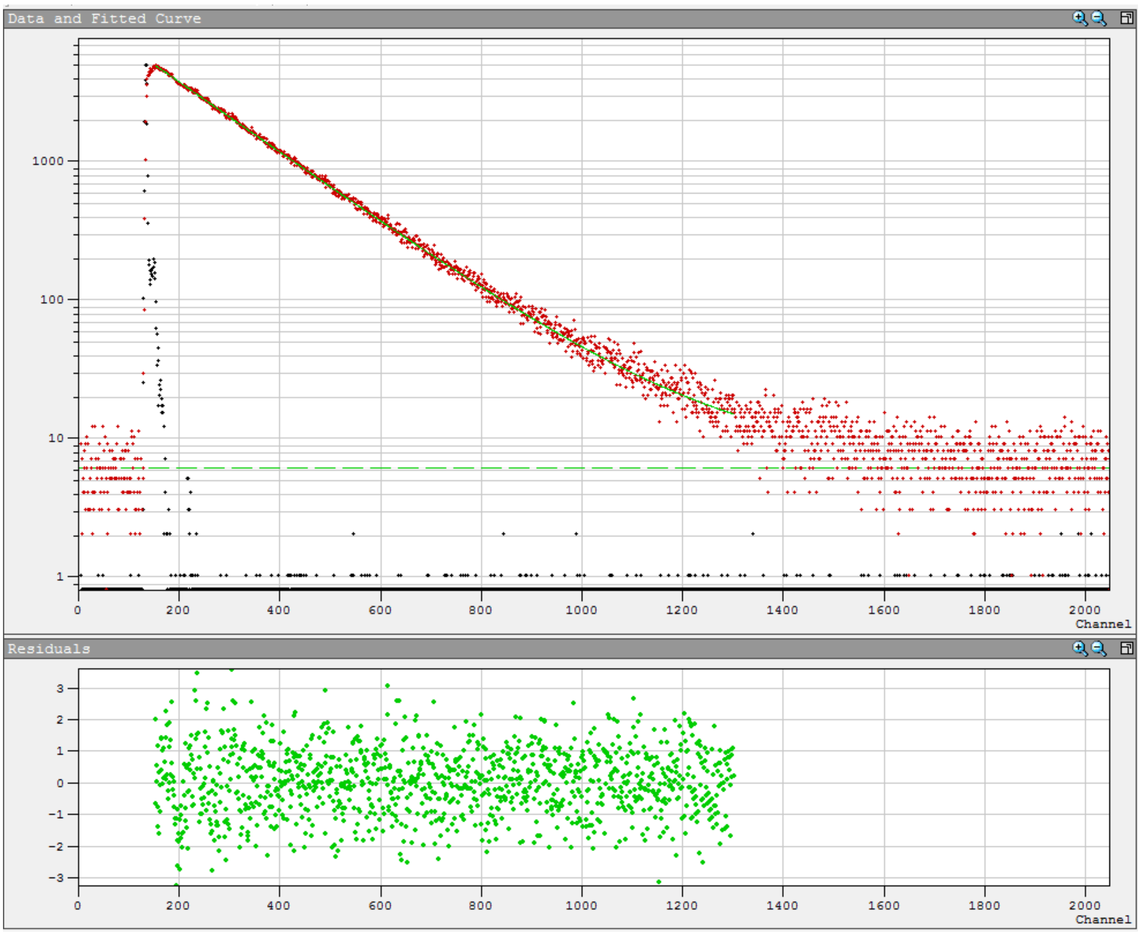

Fluorescence decay trace and reconvolution best fit of **pseudo-*m*-NB** in dimethylsulfoxide.

Reconvolution parameters of **pseudo-*m*-NB** in dimethylsulfoxide.

NaphZwit DMSO Decay 50ns.FL

❖ **Exponential Components Analysis (Reconvolution)**

Fitting range [100; 1400] channels

χ^2 1,413

	B_i	ΔB_i	f_i (%)	Δf_i (%)	τ_i (ns)	$\Delta\tau_i$ (ns)
					**4,35**	
1	0,2085	0,0005	98,986	0,267	4,206	0,001
2	0,0005	0,0005	1,014	1,116	18,049	0,038

		Probability 60%		Probability 90%	
	τ_i (ns)	$Conf_{low}$	$Conf_{high}$	$Conf_{low}$	$Conf_{high}$
1	4,206	4,155	4,222	4,118	4,237
2	18,049	-inf.	+inf.	-inf.	+inf.

Shift 0,012 (± 0,026 ns)

Decay Background 6.000 fixed (± 0)

IRF background 0,1

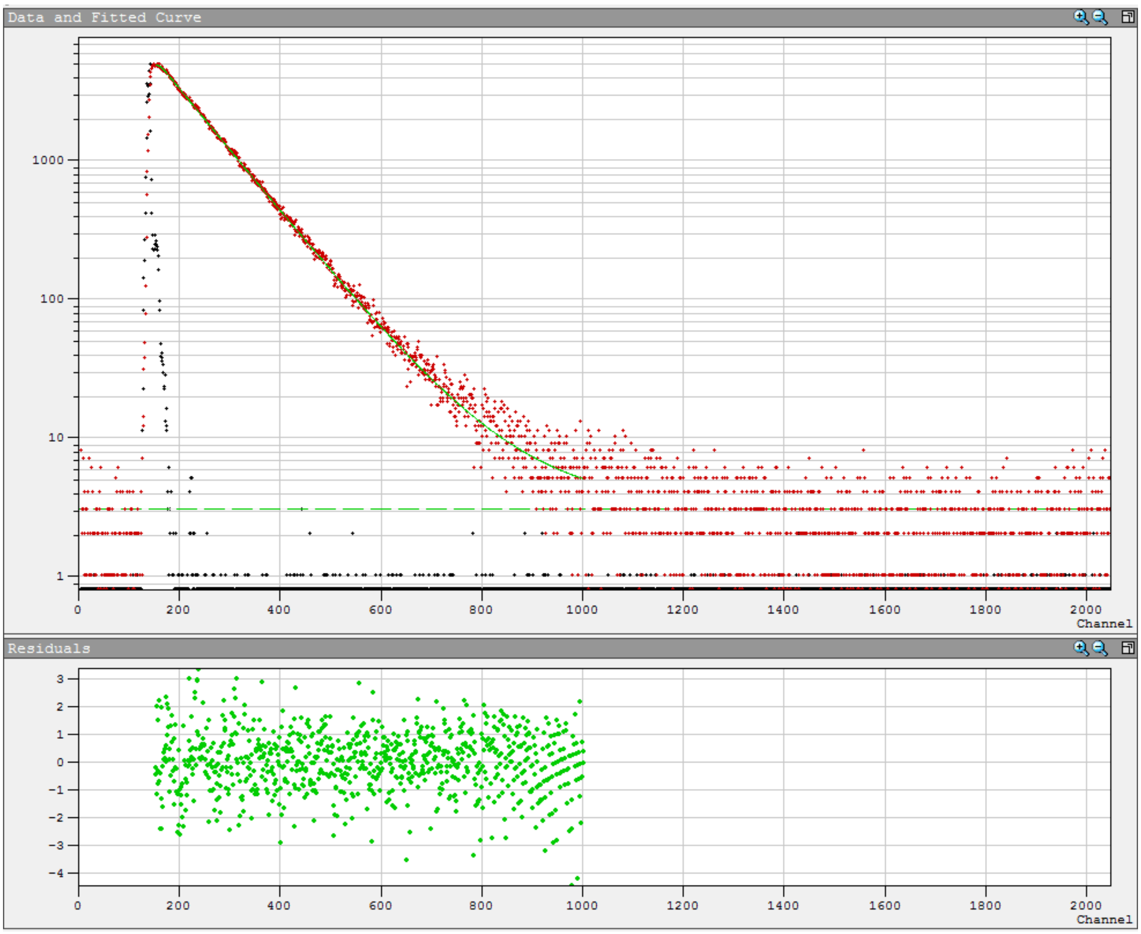

Fluorescence decay trace and reconvolution best fit of **pseudo-*m*-NB** in formamide.

Reconvolution parameters of **pseudo-*m*-NB** in formamide.

NaphZwit formamide Decay 50ns.FL

❖ **Exponential Components Analysis (Reconvolution)**

Fitting range [100; 1000] channels

χ^2 1,501

	B_i	ΔB_i	f_i (%)	Δf_i (%)	τ_i (ns)	$\Delta\tau_i$ (ns)
					**2,4**	
1	0,1546	0,0004	99,305	0,292	2,414	0,002
2	0,0002	0,0003	0,695	0,762	11,016	0,064

		Probability 60%		Probability 90%	
	τ_i (ns)	Conf $_{low}$	Conf $_{high}$	Conf $_{low}$	Conf $_{high}$
1	2,414	2,386	2,424	2,364	2,433
2	11,016	-inf.	+inf.	-inf.	+inf.

Shift	0,035	(±	0,032	ns)
Decay Background	3.000 fixed	(±	0	)
IRF background	0			

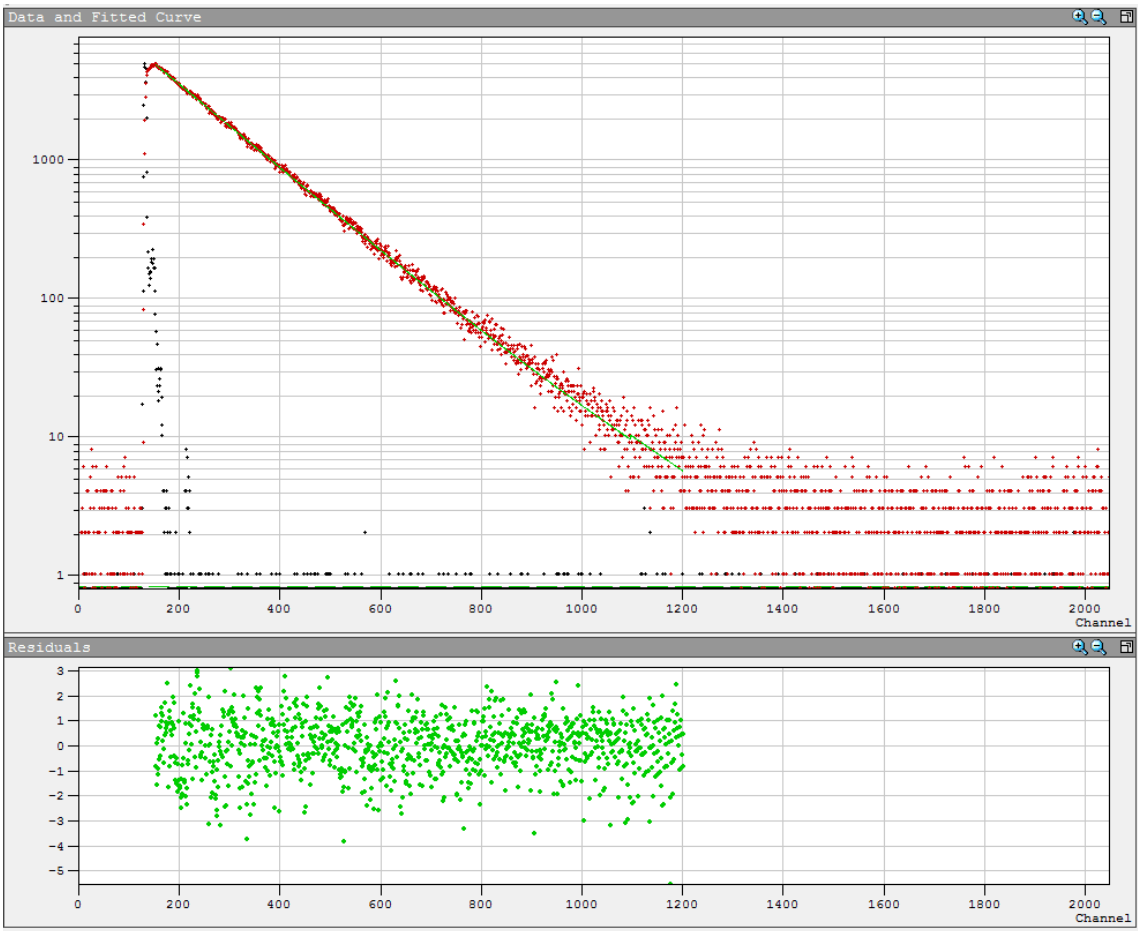

Fluorescence decay trace and reconvolution best fit of **pseudo-*m*-NB** in benzonitrile.

Reconvolution parameters of **pseudo-*m*-NB** in benzonitrile.

NaphZwit PhCN Decay 50ns.FL

❖ **Exponential Components Analysis (Reconvolution)**

Fitting range [100; 1200] channels

χ^2 1,458

	B_i	ΔB_i	f_i (%)	Δf_i (%)	τ_i (ns)	$\Delta\tau_i$ (ns)
					**3,55**	
1	0,2018	0,0004	99,955	0,244	3,541	0,001
2	2,40E-05	0,0004	0,045	0,694	13,214	0,973

		Probability 60%		Probability 90%	
	τ_i (ns)	Conf $_{low}$	Conf $_{high}$	Conf $_{low}$	Conf $_{high}$
1	3,541	3,469	3,577	3,469	3,579
2	13,214	-inf.	+inf.	-inf.	+inf.

Shift	0,016	(±	0,023	ns)
Decay Background	2.583 fixed	(±	0	)
IRF background	0			

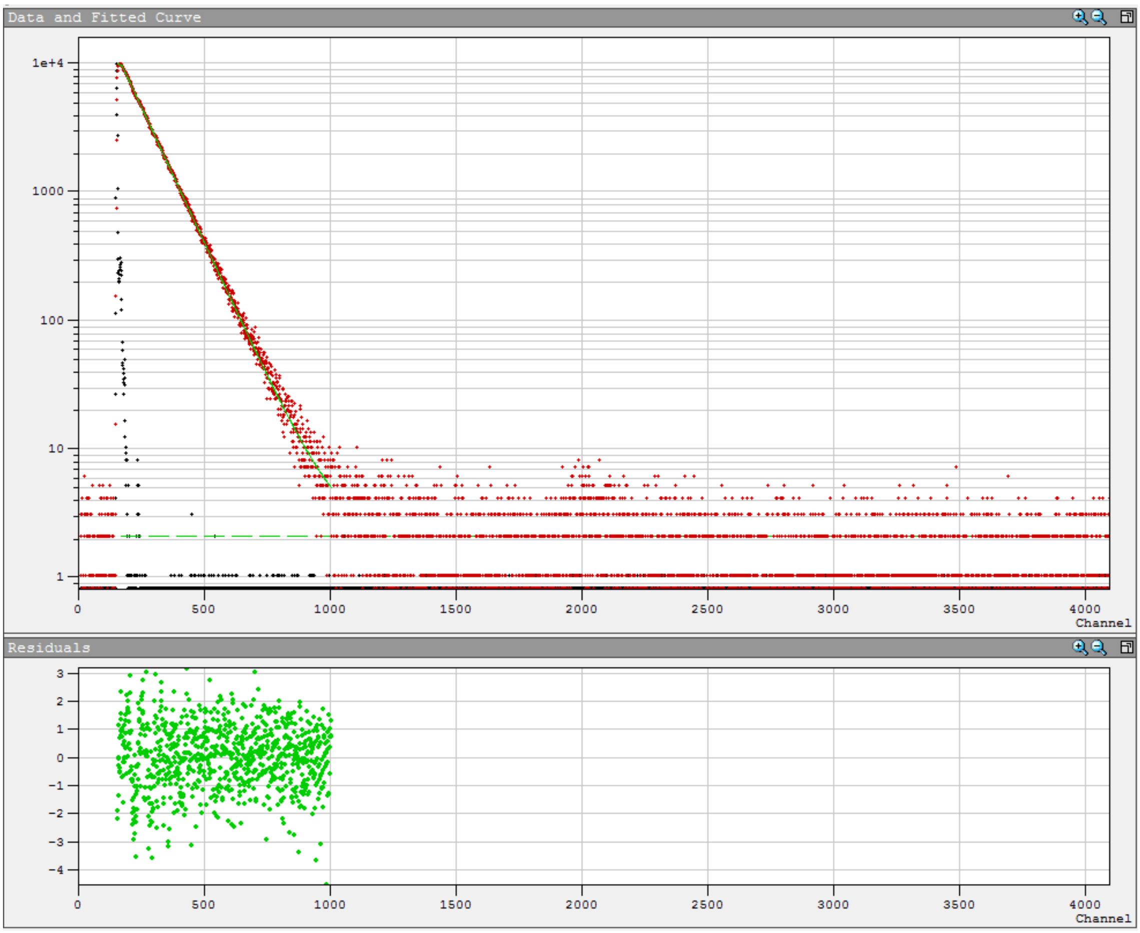

Fluorescence decay trace and reconvolution best fit of **pseudo-*p*-NB** in acetone.

Reconvolution parameters of **pseudo-*p*-NB** in acetone.

NaphQuin Ace Decay 100ns.FL

❖ **Exponential Components Analysis (Reconvolution)**

Fitting range [150; 750] channels

χ^2 1,459

	B_i	ΔB_i	f_i (%)	Δf_i (%)	τ_i (ns)	$\Delta\tau_i$ (ns)
1	0,3054	0,0015	100	0,526	2,514	0,0006

		Probability 60%		Probability 90%	
	τ_i (ns)	Conf $_{low}$	Conf $_{high}$	Conf $_{low}$	Conf $_{high}$
1	2,514	2,51	2,518	2,506	2,523

Shift	-0,011	(±	0,612	ns)
Decay Background	2.000 fixed	(±	0	)
IRF background	0			

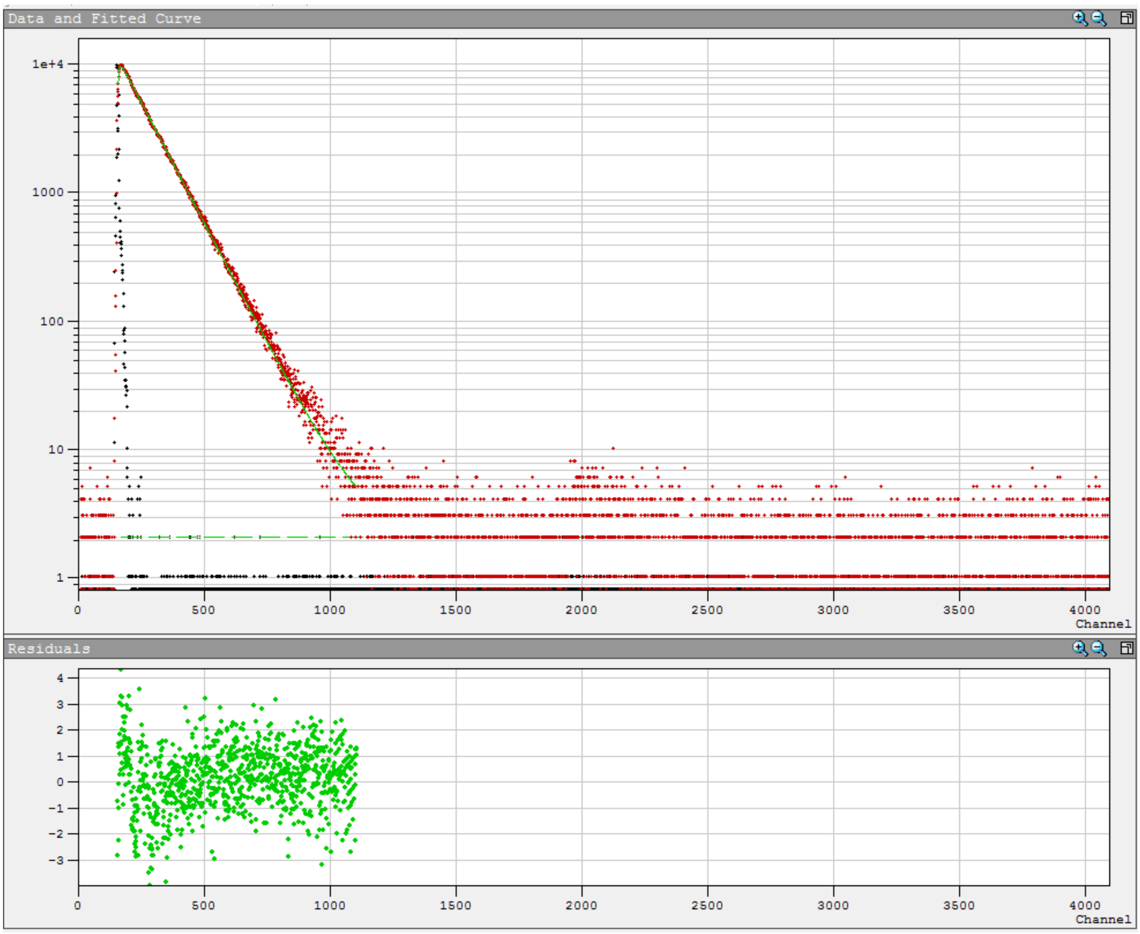

Fluorescence decay trace and reconvolution best fit of **pseudo-*p*-NB** in acetonitrile.

Reconvolution parameters of **pseudo-*p*-NB** in acetonitrile.

NaphQuin ACN Decay 100ns.FL

❖ **Exponential Components Analysis (Reconvolution)**

Fitting range [150; 1000] channels

χ^2 1,512

	B_i	ΔB_i	f_i (%)	Δf_i (%)	τ_i (ns)	$\Delta\tau_i$ (ns)
1	0,1644	0,0003	100	0,192	2,827	0,0005

		Probability 60%		Probability 90%	
	τ_i (ns)	$Conf_{low}$	$Conf_{high}$	$Conf_{low}$	$Conf_{high}$
1	2,827	2,824	2,831	2,82	2,834

Shift	0,018	(±	0,081	ns)
Decay Background	2.000 fixed	(±	0	)
IRF background	0,1			

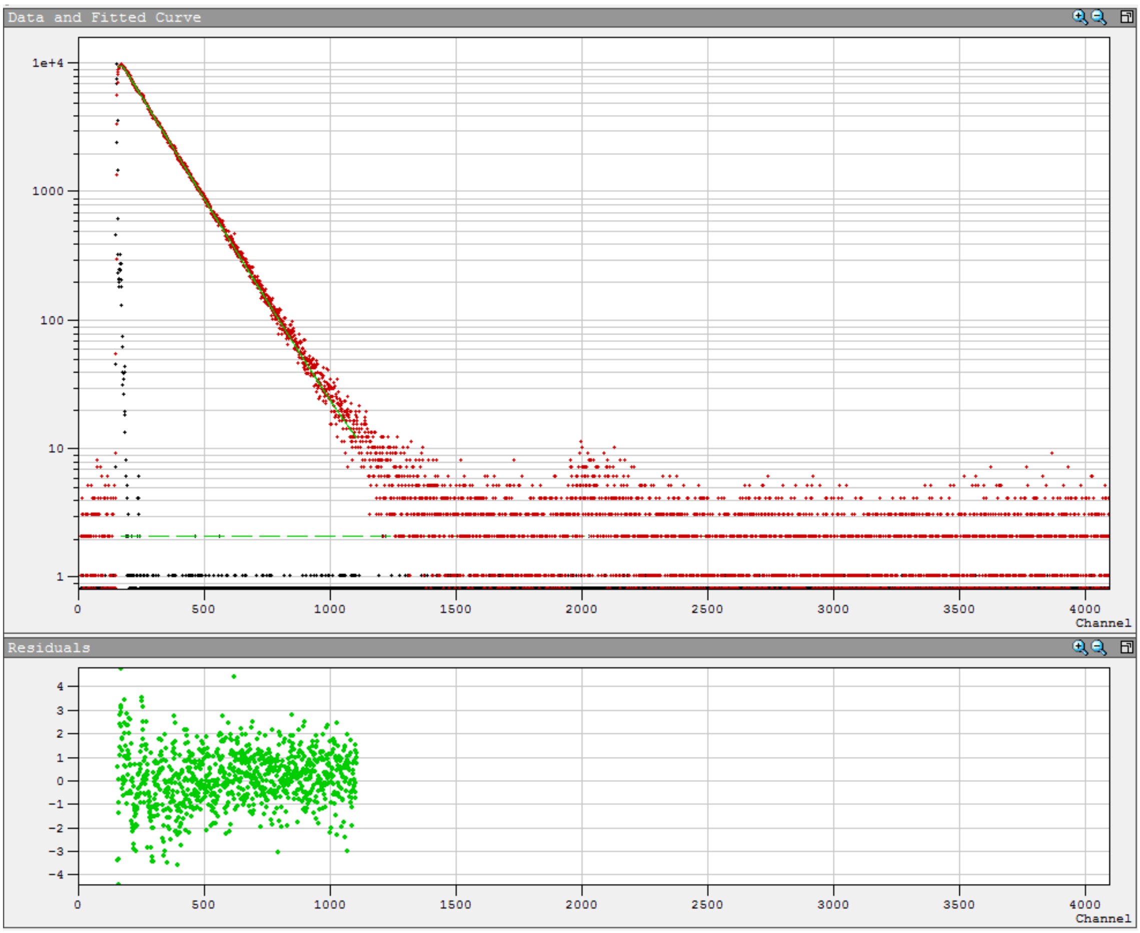

Fluorescence decay trace and reconvolution best fit of **pseudo-*p*-NB** in dimethylformamide.

Reconvolution parameters of **pseudo-*p*-NB** in dimethylformamide.

NaphQuin DMF Decay 100ns.FL

❖ **Exponential Components Analysis (Reconvolution)**

Fitting range [150; 1000] channels

χ^2 1,483

	B_i	ΔB_i	f_i (%)	Δf_i (%)	τ_i (ns)	$\Delta\tau_i$ (ns)
1	0,2972	0,0005	100	0,185	3,311	0,0004

		Probability 60%		Probability 90%	
	τ_i (ns)	$Conf_{low}$	$Conf_{high}$	$Conf_{low}$	$Conf_{high}$
1	3,311	3,307	3,315	3,304	3,318

Shift	0,078	(±	0,115	ns)
Decay Background	2.000 fixed	(±	0	)
IRF background	0			

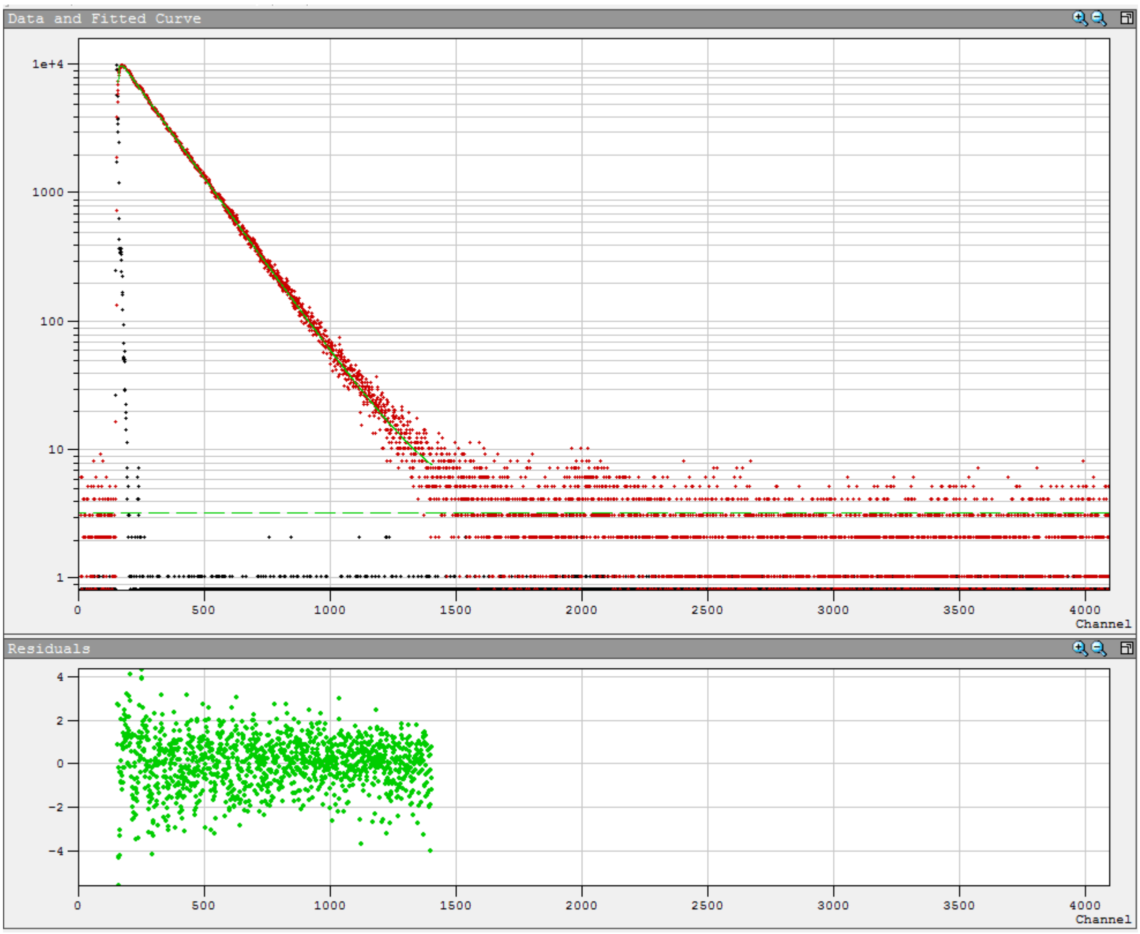

Fluorescence decay trace and reconvolution best fit of **pseudo-*p*-NB** in dimethylsulfoxide.

Reconvolution parameters of **pseudo-*p*-NB** in dimethylsulfoxide.

NaphQuin DMSO Decay 100ns.FL

❖ **Exponential Components Analysis (Reconvolution)**

Fitting range [150; 1250] channels

χ^2 1,536

	B_i	ΔB_i	f_i (%)	Δf_i (%)	τ_i (ns)	$\Delta\tau_i$ (ns)
1	0,2	0,0003	100	0,164	3,905	0,0003

		Probability 60%		Probability 90%	
	τ_i (ns)	Conf $_{low}$	Conf $_{high}$	Conf $_{low}$	Conf $_{high}$
1	3,905	3,902	3,909	3,898	3,912

Shift	0,061	(±	0,088	ns)
Decay Background	2.000 fixed	(±	0	)
IRF background	0			

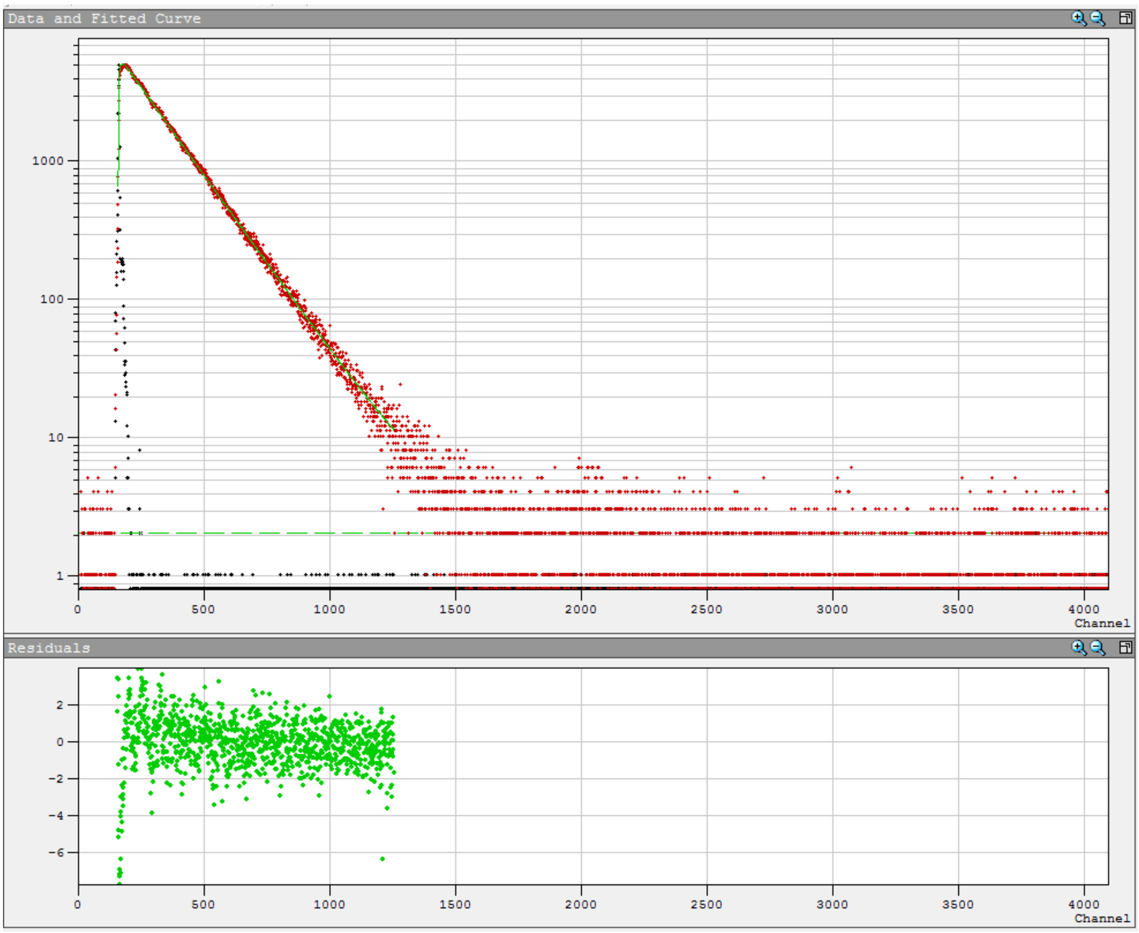

Fluorescence decay trace and reconvolution best fit of **pseudo-*p*-NB** in formamide.

Reconvolution parameters of **pseudo-*p*-NB** in formamide.

NaphQuin formamide Decay 100ns.FL

❖ **Exponential Components Analysis (Reconvolution)**

Fitting range	[150; 1250] channels					
χ^2	1,836					
	B_i	ΔB_i	f_i (%)	Δf_i (%)	τ_i (ns)	$\Delta\tau_i$ (ns)
1	0,1936	0,0005	100	0,256	4,172	0,0004

		Probability 60%		Probability 90%	
	τ_i (ns)	Conf $_{low}$	Conf $_{high}$	Conf $_{low}$	Conf $_{high}$
1	4,172	4,164	4,18	4,156	4,188
Shift	0,023	(±	0,041	ns)	
Decay Background	2.000 fixed	(±	0	)	
IRF background	0,1				

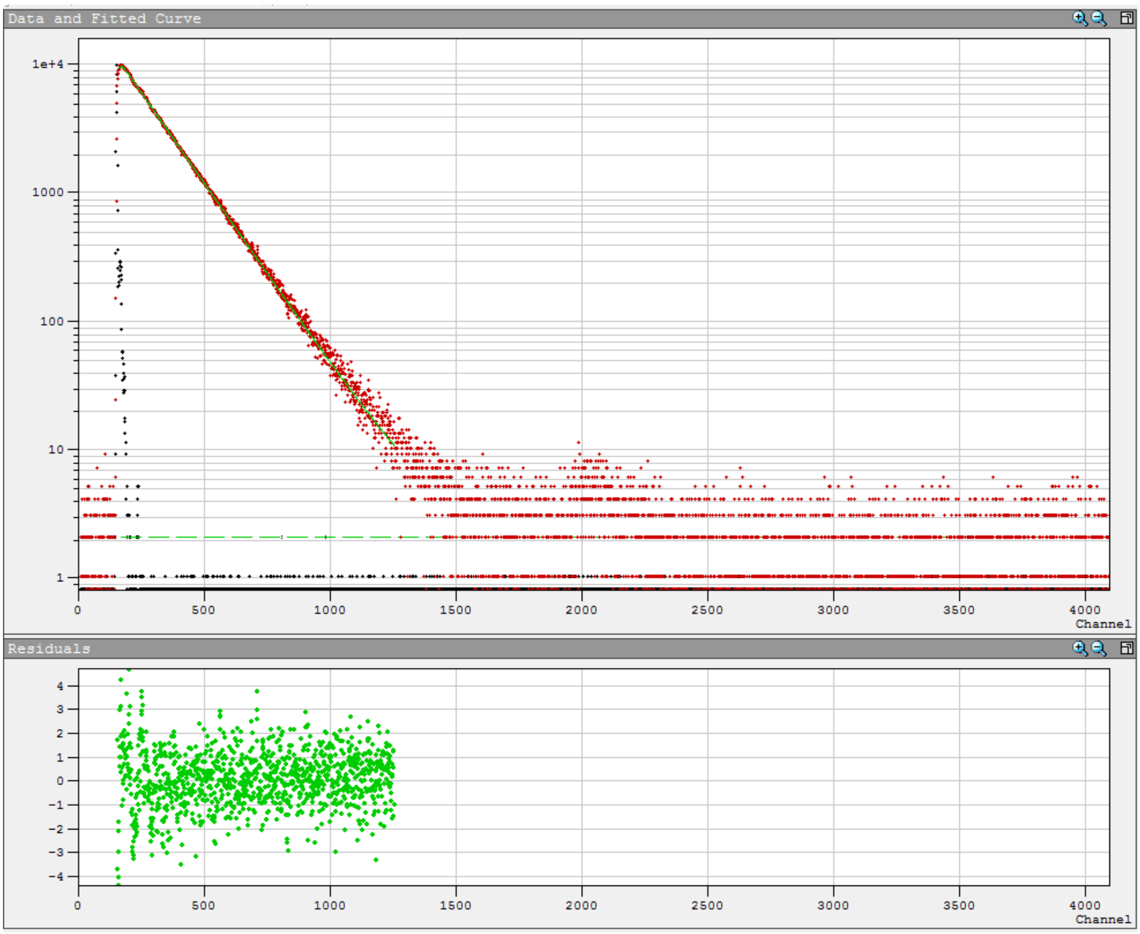

Fluorescence decay trace and reconvolution best fit of **pseudo-*p*-NB** in benzonitrile.

Reconvolution parameters of **pseudo-*p*-NB** in benzonitrile.

NaphQuin PhCN Decay 100ns.FL

❖ **Exponential Components Analysis (Reconvolution)**

Fitting range [150; 1250] channels

χ^2 1,42

	B_i	ΔB_i	f_i (%)	Δf_i (%)	τ_i (ns)	$\Delta\tau_i$ (ns)
1	0,292	0,0004	100	0,154	3,764	0,0003

		Probability 60%		Probability 90%	
	τ_i (ns)	Conf $_{low}$	Conf $_{high}$	Conf $_{low}$	Conf $_{high}$
1	3,764	3,761	3,767	3,757	3,77

Shift	0,101	(±	0,093	ns)
Decay Background	2.000 fixed	(±	0	)
IRF background	0			

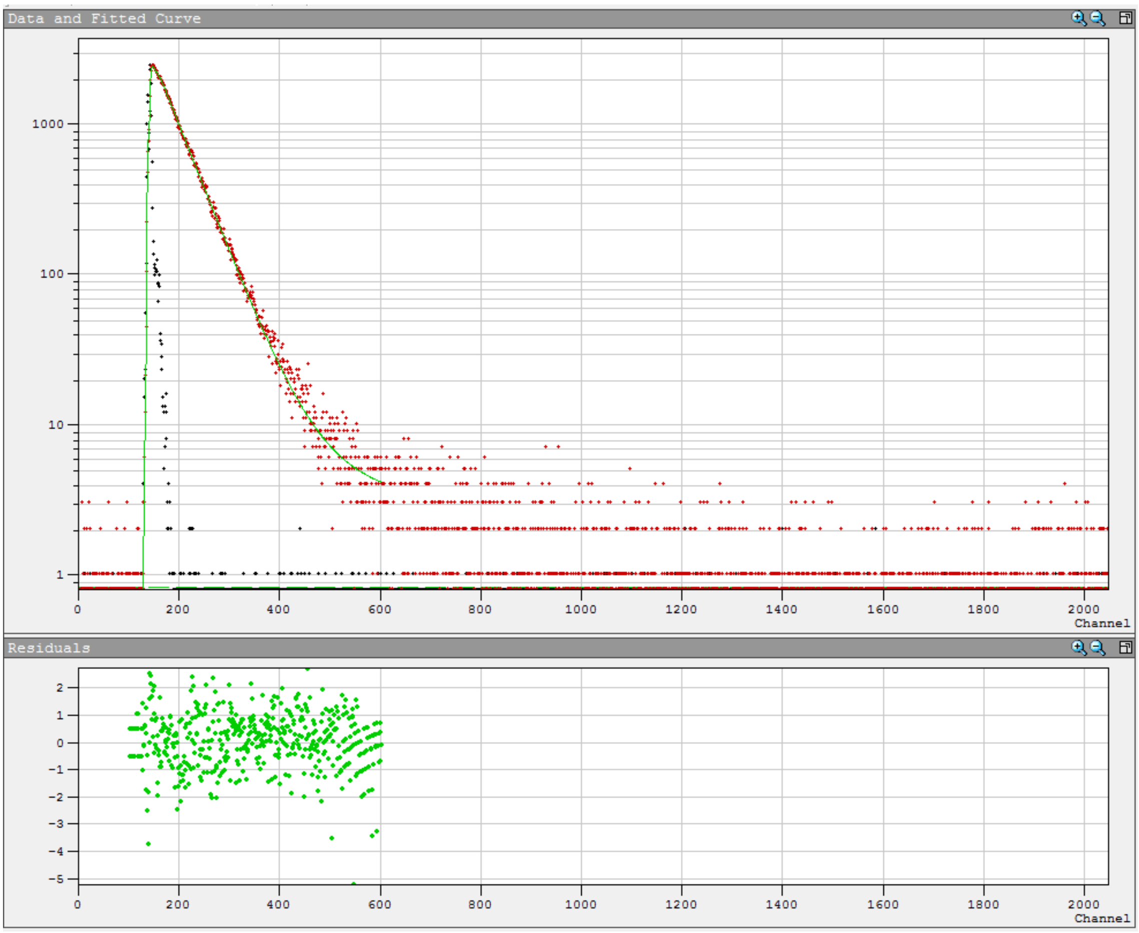

Fluorescence decay trace and reconvolution best fit of ***m*-PB** in 1.63% PEG-400/CH_3CN.

Reconvolution parameters of ***m*-PB** in 1.63% PEG-400/CH_3CN.

PhenZwit ACN 1_63 PEG Decay 50ns.FL

❖ **Exponential Components Analysis (Reconvolution)**

Fitting range [100; 600] channels

χ^2 1,025

	B_i	ΔB_i	f_i (%)	Δf_i (%)	τ_i (ns)	$\Delta\tau_i$ (ns)
**intensity-weighted lifetime**					**1,32**	
1	0,1707	0,0009	95,324	0,96	1,214	0,006
2	0,0029	0,001	4,676	1,684	3,482	0,04

		Probability 60%		Probability 90%	
	τ_i (ns)	$Conf_{low}$	$Conf_{high}$	$Conf_{low}$	$Conf_{high}$
1	1,214	1,175	1,239	1,152	1,246
2	3,482	2,372	6,399	2,083	+inf.

Shift	0,005	(±	0,028	ns)
Decay Background	1.000 fixed	(±	0	)
IRF background	0,1			

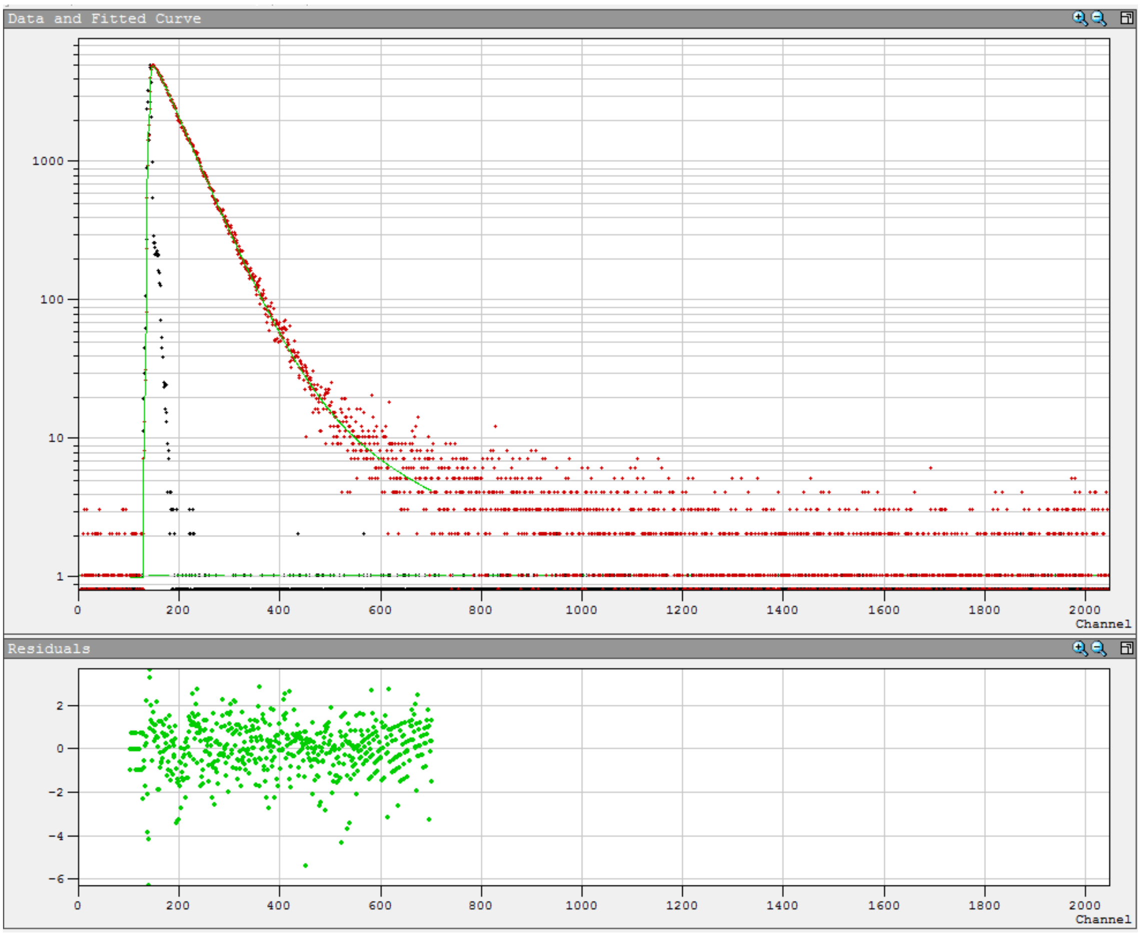

Fluorescence decay trace and reconvolution best fit of ***m*-PB** in 3.60% PEG-400/CH_3CN.

Reconvolution parameters of ***m*-PB** in 3.60% PEG-400/CH_3CN.

PhenZwit ACN 3_60 PEG Decay 50ns.FL

❖ **Exponential Components Analysis (Reconvolution)**

Fitting range [100; 700] channels

χ^2 1,424

	B_i	ΔB_i	f_i (%)	Δf_i (%)	τ_i (ns)	$\Delta\tau_i$ (ns)
**intensity-weighted lifetime**					**1,37**	
1	0,1714	0,0005	96,659	0,588	1,259	0,004
2	0,0016	0,0004	3,341	0,862	4,666	0,025

		Probability 60%		Probability 90%	
	τ_i (ns)	Conf $_{low}$	Conf $_{high}$	Conf $_{low}$	Conf $_{high}$
1	1,259	1,24	1,273	1,23	1,278
2	4,666	3,484	7,035	3,148	8,666

Shift 0,007 (± 0,028 ns)

Decay Background 1.000 fixed (± 0)

IRF background 0,1

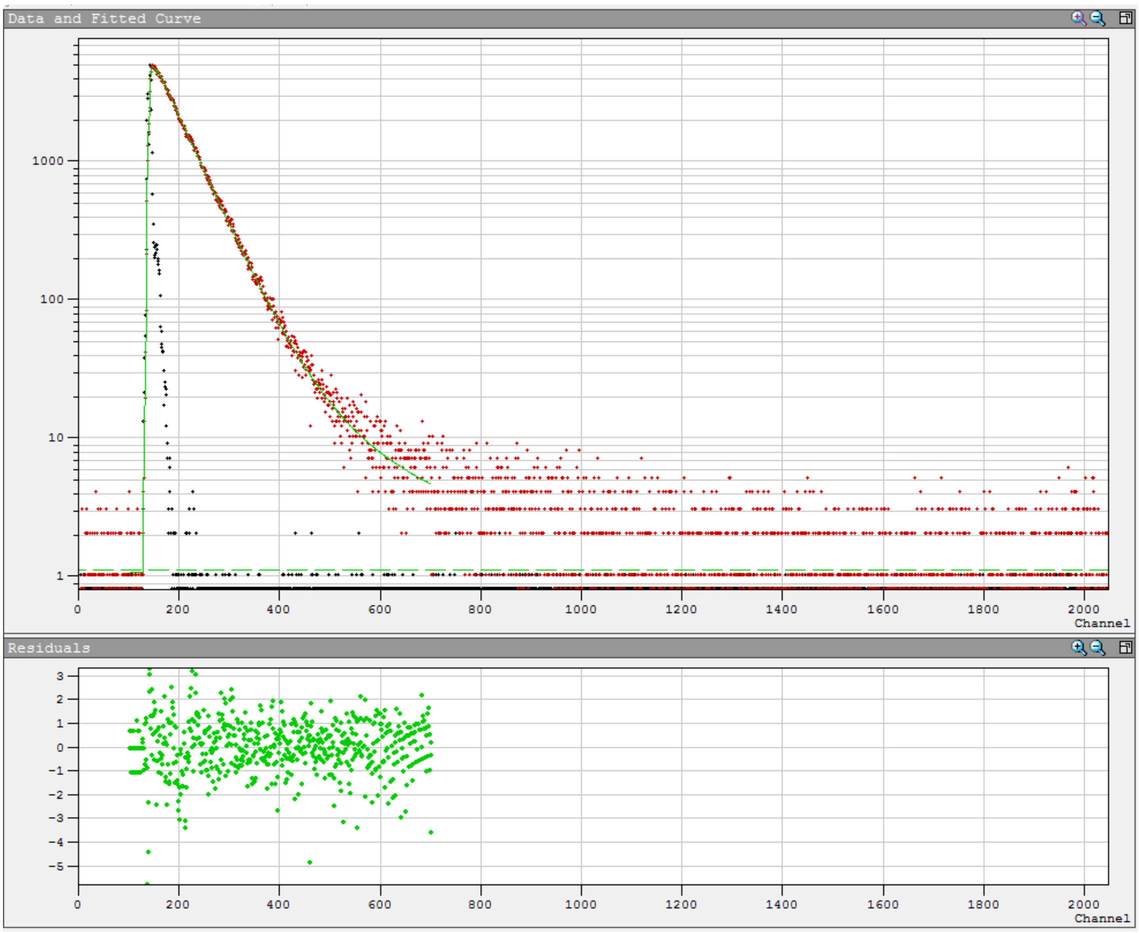

Fluorescence decay trace and reconvolution best fit of ***m*-PB** in 9.05% PEG-400/CH_3CN.

Reconvolution parameters of ***m*-PB** in 9.05% PEG-400/CH_3CN.

PhenZwit ACN 9_05 PEG Decay 50ns.FL

❖ Exponential Components Analysis (Reconvolution)

Fitting range [100; 700] channels

χ^2 1,294

	B_i	ΔB_i	f_i (%)	Δf_i (%)	τ_i (ns)	$\Delta\tau_i$ (ns)
**intensity-weighted lifetime**					**1,43**	
1	0,1735	0,0005	96,767	0,543	1,317	0,004
2	0,0015	0,0004	3,233	0,892	4,932	0,031

		Probability 60%		Probability 90%	
	τ_i (ns)	Conf $_{low}$	Conf $_{high}$	Conf $_{low}$	Conf $_{high}$
1	1,317	1,294	1,332	1,278	1,337
2	4,932	3,113	8,075	2,557	+inf.

Shift 0,0008 (± 0,028 ns)

Decay Background 1,073 (± 0,622)

IRF background 0,2

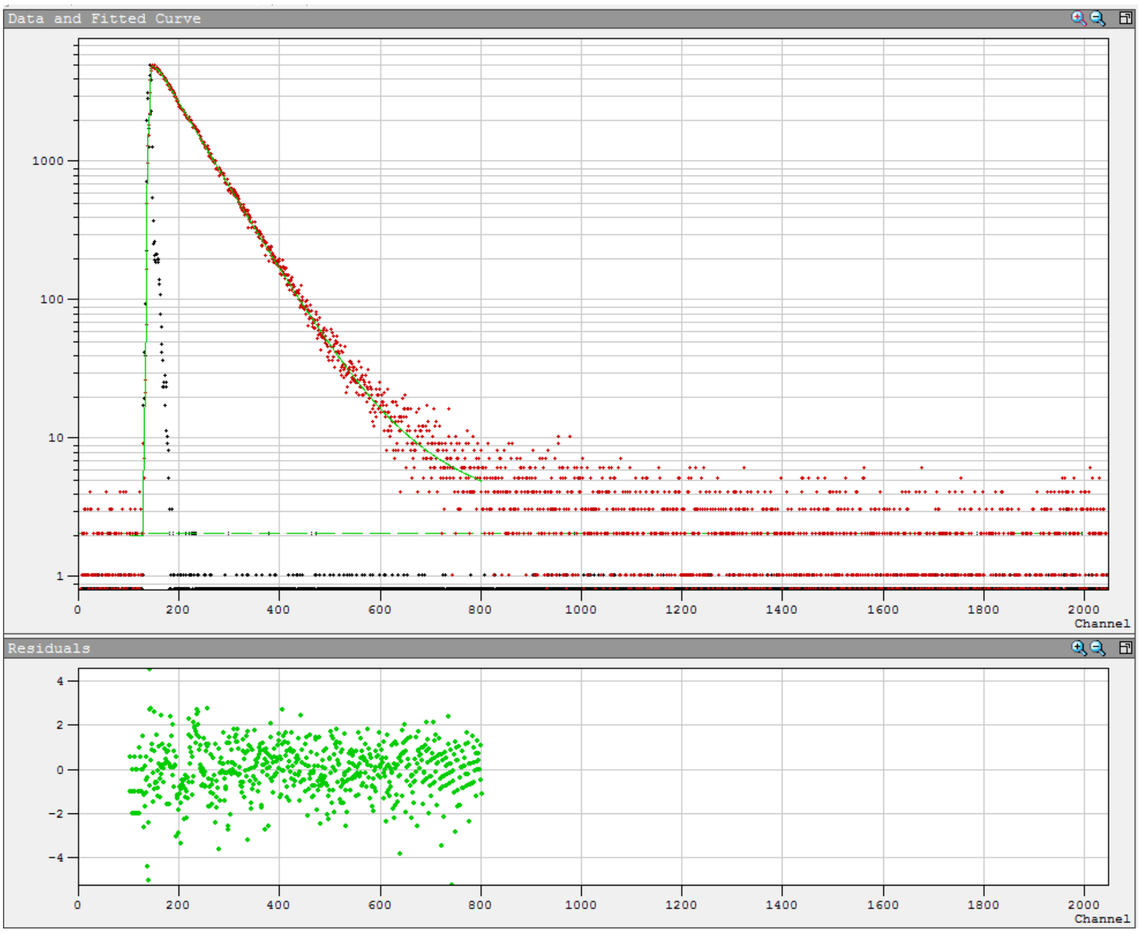

Fluorescence decay trace and reconvolution best fit of ***m*-PB** in 37.39% PEG-400/CH_3CN.

Reconvolution parameters of ***m*-PB** in 37.39% PEG-400/CH_3CN.

PhenZwit ACN 37_39 PEG Decay 50ns.FL

❖ **Exponential Components Analysis (Reconvolution)**

Fitting range [100; 800] channels

χ^2 1,358

	B_i	ΔB_i	f_i (%)	Δf_i (%)	τ_i (ns)	$\Delta\tau_i$ (ns)
**intensity-weighted lifetime**					**1,82**	
1	0,17	0,0005	98,022	0,422	1,728	0,003
2	0,001	0,0005	1,978	0,931	6,075	0,036

		Probability 60%		Probability 90%	
	τ_i (ns)	$Conf_{low}$	$Conf_{high}$	$Conf_{low}$	$Conf_{high}$
1	1,728	1,699	1,746	1,682	1,75
2	6,075	3,718	+inf.	3,222	+inf.

Shift	0,003	(±	0,028	ns)
Decay Background	2.000 fixed	(±	0	)
IRF background	0,1			

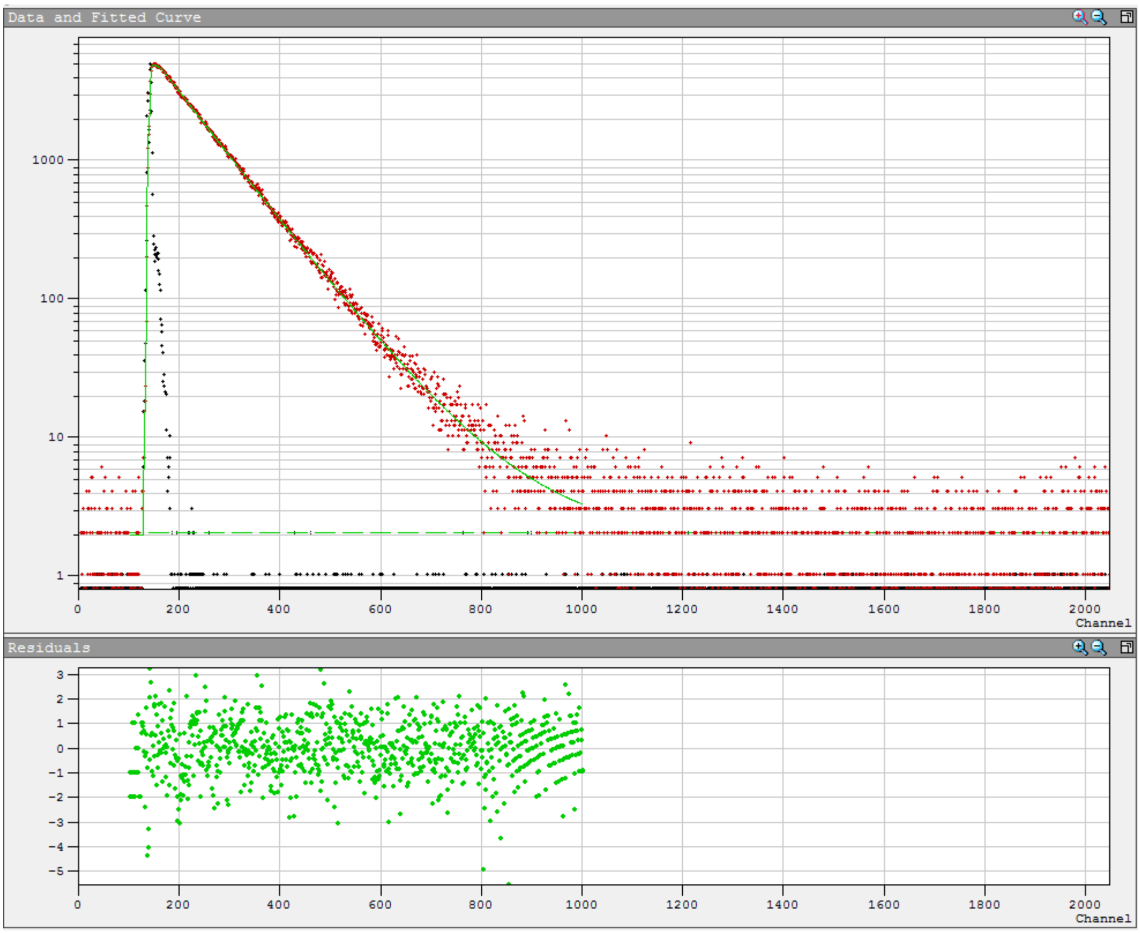

Fluorescence decay trace and reconvolution best fit of ***m*-PB** in 57.33% PEG-400/CH_3CN.

Reconvolution parameters of ***m*-PB** in 57.33% PEG-400/CH_3CN.

PhenZwit ACN 57_33 PEG Decay 50ns.FL

❖ Exponential Components Analysis (Reconvolution)

Fitting range [100; 1000] channels

χ^2 1,264

	B_i	ΔB_i	f_i (%)	Δf_i (%)	τ_i (ns)	$\Delta\tau_i$ (ns)
**intensity-weighted lifetime**					**2,32**	
1	0,1594	0,0054	91,59	3,379	2,2	0,006
2	0,0089	0,0057	8,41	5,405	3,605	0,036

		Probability 60%		Probability 90%	
	τ_i (ns)	$Conf_{low}$	$Conf_{high}$	$Conf_{low}$	$Conf_{high}$
1	2,2	1,945	2,256	1,727	2,269
2	3,605	2,662	5,808	-inf.	+inf.

Shift	0,008	(±	0,025	ns)
Decay Background	2.000 fixed	(±	0	)
IRF background	0,1			

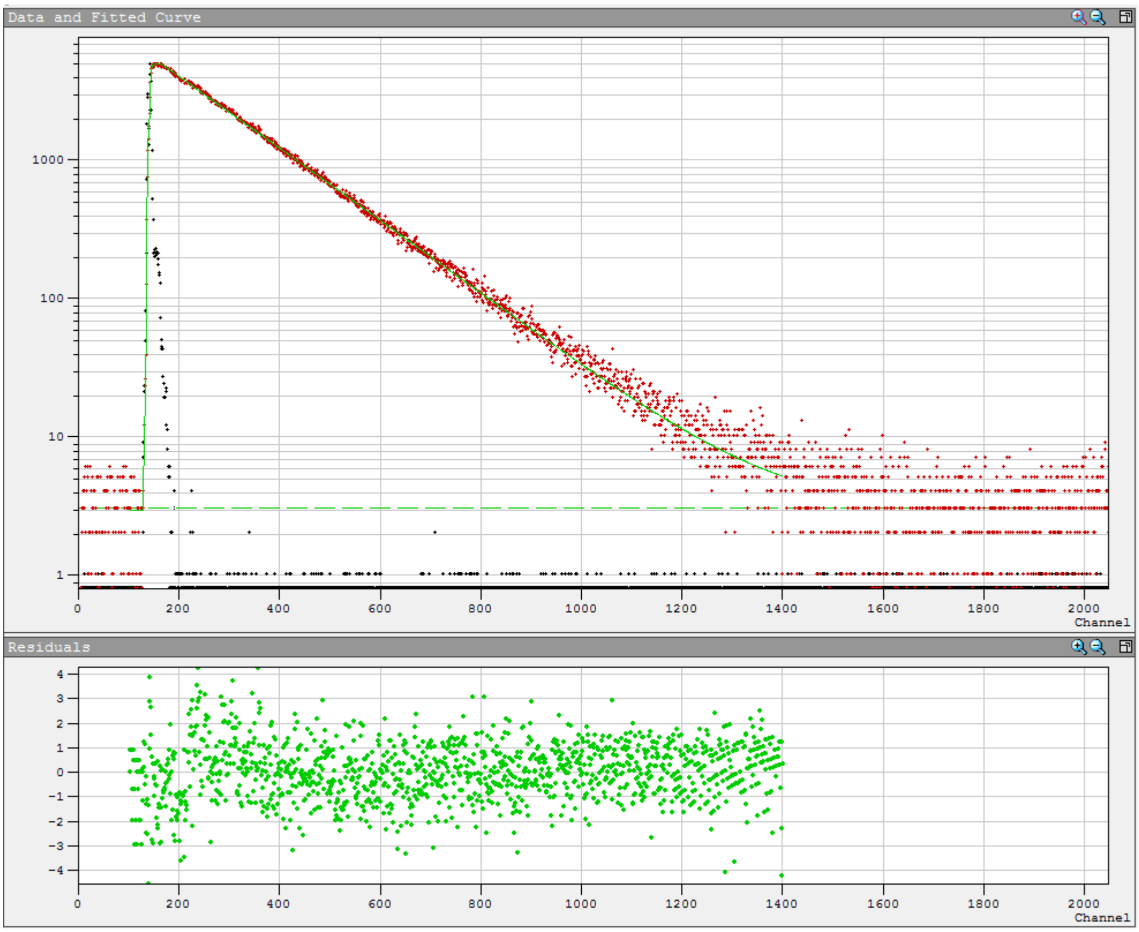

Fluorescence decay trace and reconvolution best fit of ***m*-PB** in 100% PEG-400.

Reconvolution parameters of ***m*-PB** in 100% PEG-400.

PhenZwit ACN 100 PEG Decay 50ns.FL

❖ Exponential Components Analysis (Reconvolution)

Fitting range [100; 1400] channels

χ^2 1,414

	B_i	ΔB_i	f_i (%)	Δf_i (%)	τ_i (ns)	$\Delta\tau_i$ (ns)
**intensity-weighted lifetime**					**4,5**	
1	0,3288	0,849	65,203	168,691	4,394	0,022
2	0,1641	0,8491	34,797	179,728	4,696	0,037

		Probability 60%		Probability 90%	
	τ_i (ns)	Conf $_{low}$	Conf $_{high}$	Conf $_{low}$	Conf $_{high}$
1	4,394	4,2	4,484	4,15	4,485
2	4,696	3,771	5,563	-inf.	6,386

Shift	0,009	(±	0,031	ns)
Decay Background	3.000 fixed	(±	0	)
IRF background	0,1			

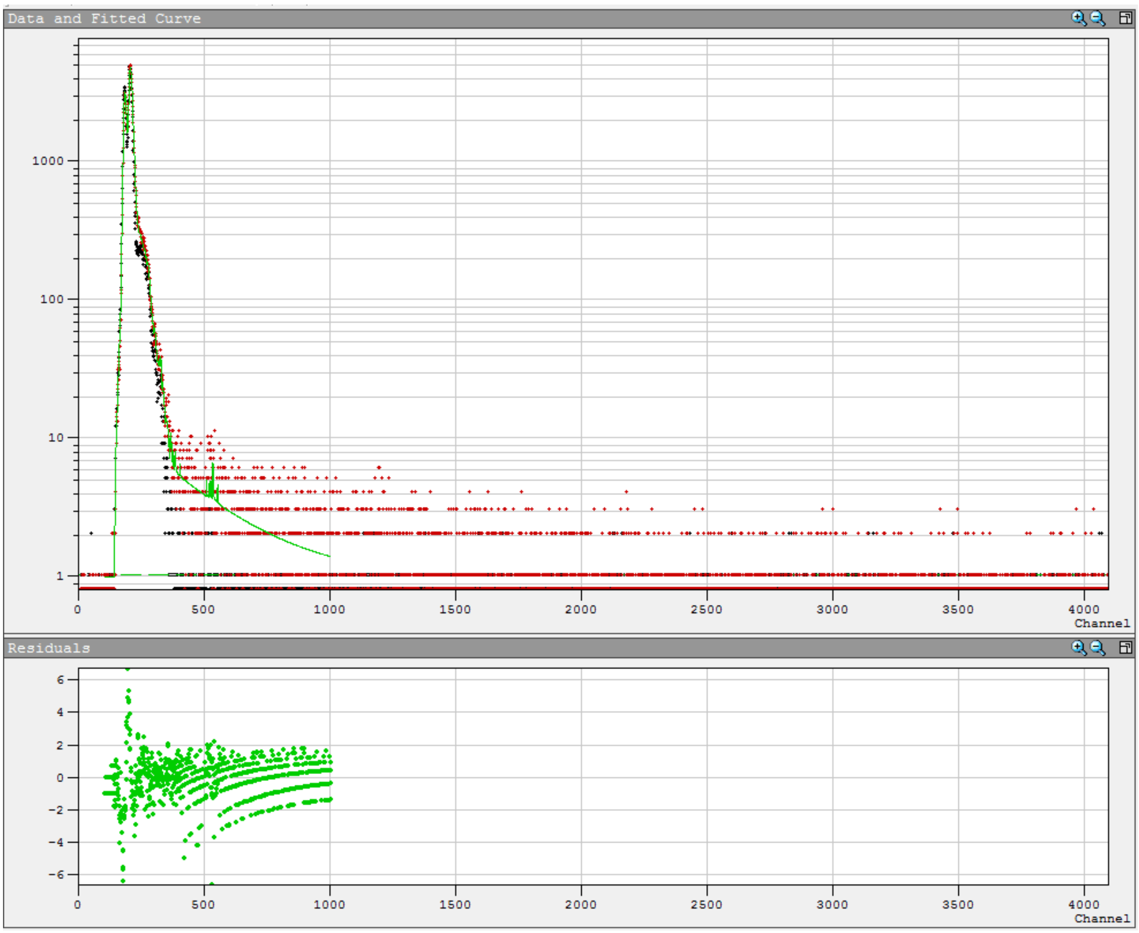

Fluorescence decay trace and reconvolution best fit of ***p*-PB** in 1.63% PEG-400/CH_3CN.

Reconvolution parameters of ***p*-PB** in 1.63% PEG-400/CH_3CN.

PhenQuin ACN 1_63 PEG Decay 20ns.FL

❖ **Exponential Components Analysis (Reconvolution)**

Fitting range [100; 1000] channels

χ^2 1,745

	B_i	ΔB_i	f_i (%)	Δf_i (%)	τ_i (ns)	$\Delta\tau_i$ (ns)
**intensity-weighted lifetime**					**0,02**	
1	1,9508	0,4659	82,389	7,60E+05	0,004	36,218
2	0,0367	0,0029	16,715	335,416	0,042	0,844
3	6,70E-05	8,70E-06	0,896	0,186	1,24	0,096

		Probability 60%		Probability 90%	
	τ_i (ns)	Conf $_{low}$	Conf $_{high}$	Conf $_{low}$	Conf $_{high}$
1	0,004	0,002	0,007	-inf.	+inf.
2	0,042	0,04	0,048	0,038	0,051
3	1,24	0,858	1,889	0,773	2,19

Shift 0,003 (± 0,073 ns)

Decay Background 1.000 fixed (± 0)

IRF background 0,1

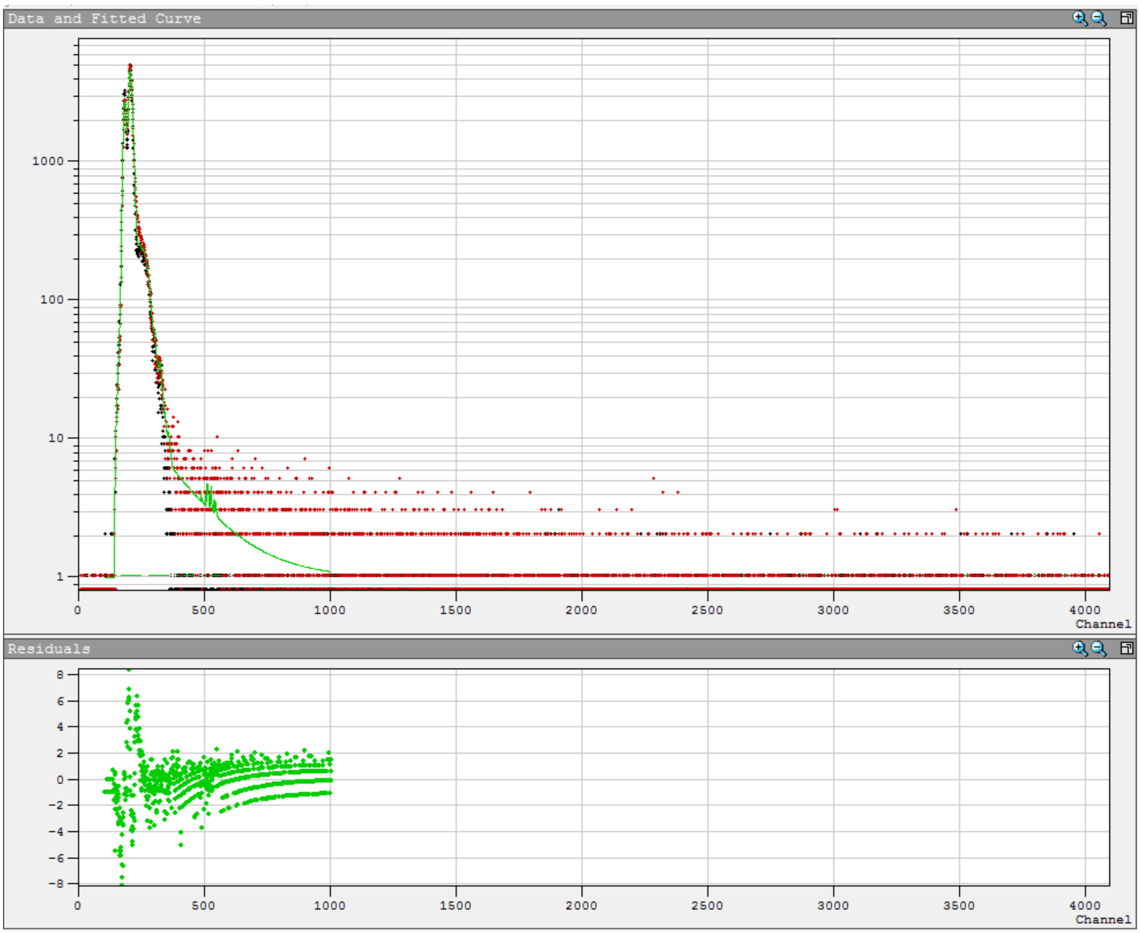

Fluorescence decay trace and reconvolution best fit of ***p*-PB** in 3.60% PEG-400/CH_3CN.

Reconvolution parameters of ***p*-PB** in 3.60% PEG-400/CH_3CN.

PhenQuin ACN 3_60 PEG Decay 20ns.FL

❖ Exponential Components Analysis (Reconvolution)

Fitting range [100; 1000] channels

χ^2 2,66

	B_i	ΔB_i	f_i (%)	Δf_i (%)	τ_i (ns)	$\Delta\tau_i$ (ns)
**intensity-weighted lifetime**					**0,03**	
1	0,0079	25,9692	2,406	-2,00E+06	0,019	16945,73
2	0,3139	25,9693	96,242	2,20E+06	0,019	423,747
3	0,0001	3,00E-05	1,351	0,85	0,785	0,271

		Probability 60%		Probability 90%	
	τ_i (ns)	Conf $_{low}$	Conf $_{high}$	Conf $_{low}$	Conf $_{high}$
1	0,019	-inf.	+inf.	-inf.	+inf.
2	0,019	-inf.	0,02	-inf.	0,021
3	0,785	-inf.	1,446	-inf.	+inf.

Shift -0,01 (± 0,103 ns)

Decay Background 1.000 fixed (± 0)

IRF background 0,1

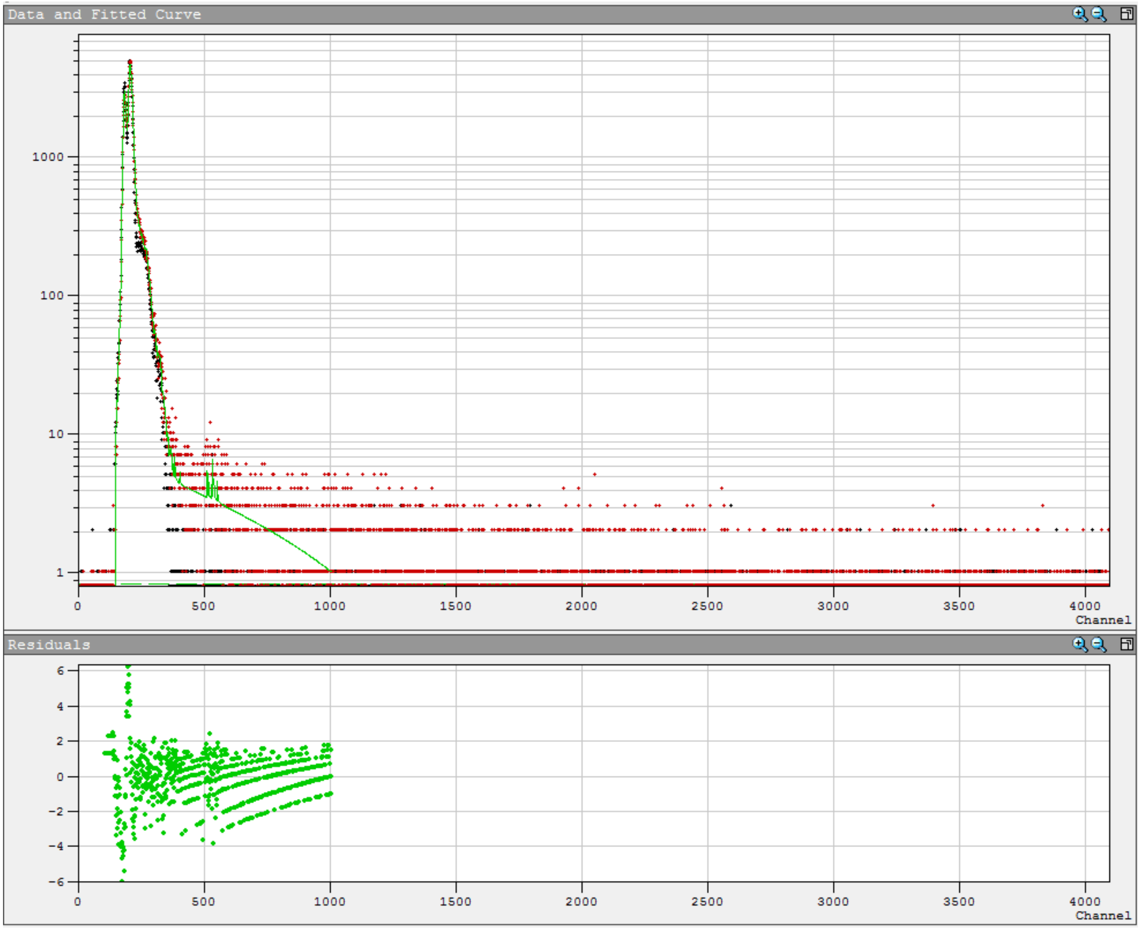

Fluorescence decay trace and reconvolution best fit of ***p*-PB** in 9.05% PEG-400/CH_3CN.

Reconvolution parameters of ***p*-PB** in 9.05% PEG-400/CH_3CN.

PhenQuin ACN 9_05 PEG Decay 20ns.FL

❖ Exponential Components Analysis (Reconvolution)

Fitting range [100; 1000] channels

χ^2 2,034

	B_i	ΔB_i	f_i (%)	Δf_i (%)	τ_i (ns)	$\Delta\tau_i$ (ns)
**intensity-weighted lifetime**					**0,09**	
1	1,1318	0,2983	79,157	4,90E+05	0,005	33,817
2	0,0312	0,0035	18,608	430,839	0,046	1,07
3	5,30E-05	5,80E-06	2,234	0,288	3,264	0,064

		Probability 60%		Probability 90%	
	τ_i (ns)	$Conf_{low}$	$Conf_{high}$	$Conf_{low}$	$Conf_{high}$
1	0,005	0,003	0,009	-inf.	0,009
2	0,046	0,043	0,054	0,041	0,056
3	3,264	1,649	+inf.	-inf.	+inf.

Shift	-0,003	(± 0,131	ns)
Decay Background	-1,266	(± 0,57	)
IRF background	0,2		

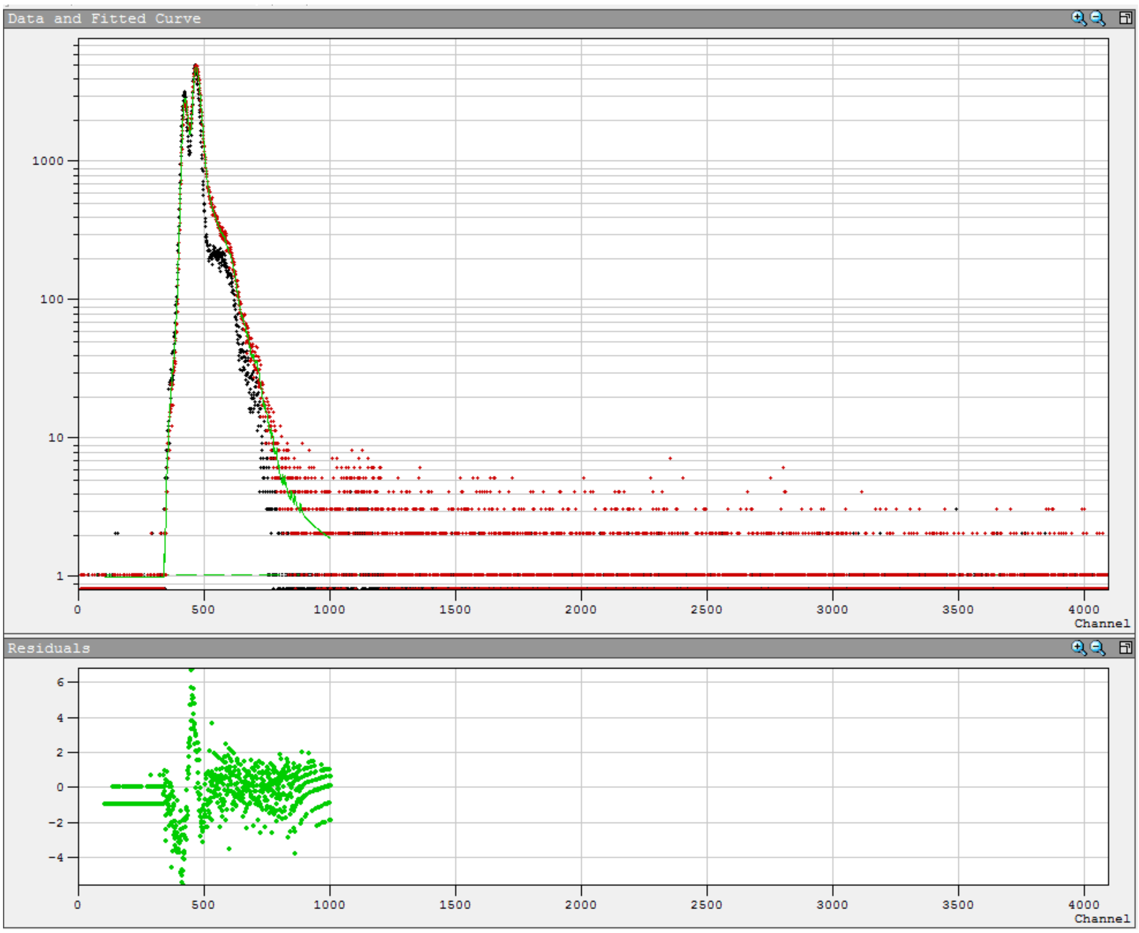

Fluorescence decay trace and reconvolution best fit of ***p*-PB** in 37.39% PEG-400/CH_3CN.

Reconvolution parameters of ***p*-PB** in 37.39% PEG-400/CH_3CN.

PhenQuin ACN 37_39 PEG Decay 10ns.FL

❖ **Exponential Components Analysis (Reconvolution)**

Fitting range [100; 1000] channels

χ^2 2,217

	B_i	ΔB_i	f_i (%)	Δf_i (%)	τ_i (ns)	$\Delta\tau_i$ (ns)
**intensity-weighted lifetime**					**0,03**	
1	0,1922	0,0135	73,42	40712,82	0,012	6,652
2	0,0112	0,0013	25,544	316,408	0,072	0,88
3	7,90E-05	6,10E-05	1,036	3,117	0,414	0,925

		Probability 60%		Probability 90%	
	τ_i (ns)	Conf $_{low}$	Conf $_{high}$	Conf $_{low}$	Conf $_{high}$
1	0,012	0,009	0,013	0,009	0,014
2	0,072	0,055	0,079	0,04	0,084
3	0,414	-inf.	+inf.	-inf.	+inf.

Shift	-0,002	(±	0,21	ns)
Decay Background	1.000 fixed	(±	0	)
IRF background	0,1			

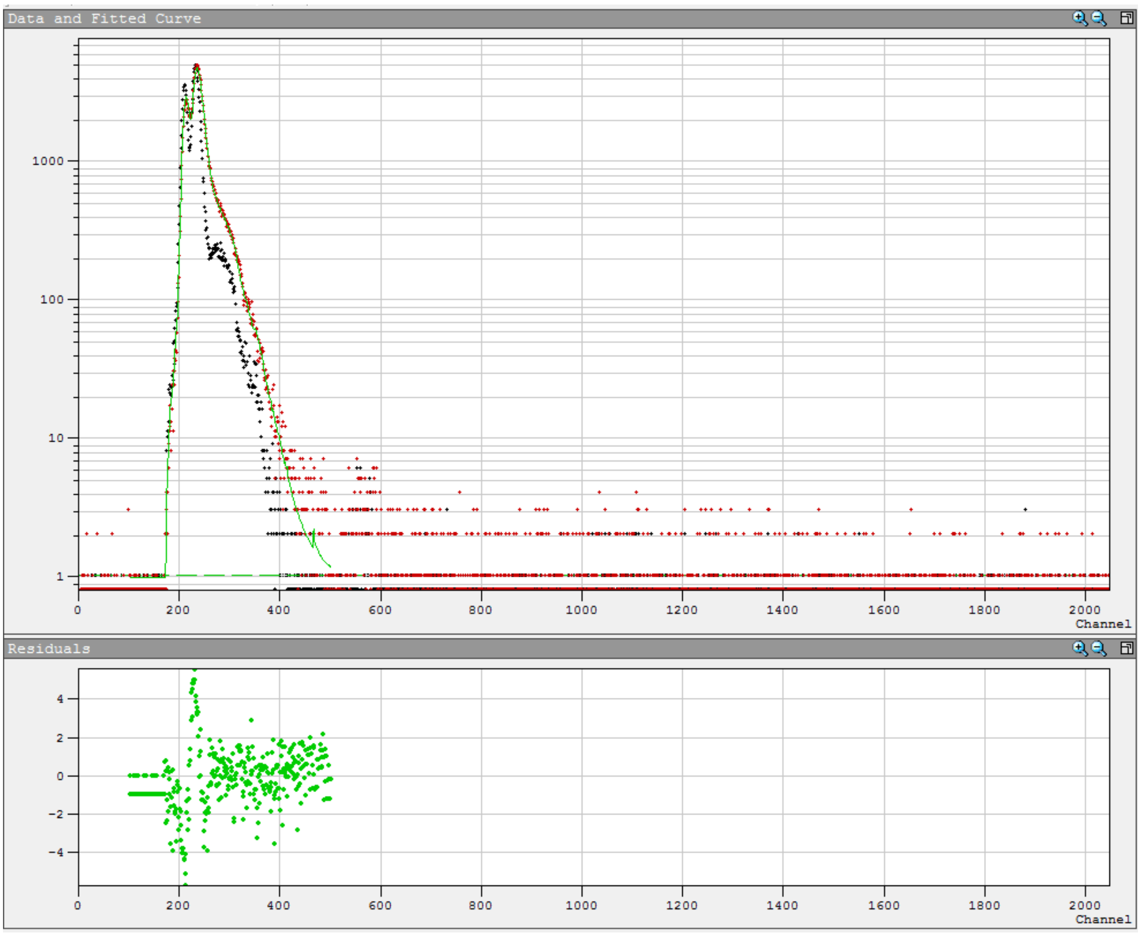

Fluorescence decay trace and reconvolution best fit of ***p*-PB** in 57.33% PEG-400/CH_3CN.

Reconvolution parameters of ***p*-PB** in 57.33% PEG-400/CH_3CN.

PhenQuin ACN 57_33 PEG Decay 10ns.FL

❖ **Exponential Components Analysis (Reconvolution)**

Fitting range [100; 500] channels

χ^2 2,499

	B_i	ΔB_i	f_i (%)	Δf_i (%)	τ_i (ns)	$\Delta\tau_i$ (ns)
**intensity-weighted lifetime**					**0,04**	
1	0,6606	2,0088	32,283	3,80E+06	0,004	455,701
2	0,12	0,0169	51,83	4564,667	0,034	3,01
3	0,0092	0,0018	15,886	59,395	0,137	0,487

		Probability 60%		Probability 90%	
	τ_i (ns)	Conf $_{low}$	Conf $_{high}$	Conf $_{low}$	Conf $_{high}$
1	0,004	-inf.	+inf.	-inf.	+inf.
2	0,034	0,03	+inf.	0,027	+inf.
3	0,137	0,127	0,162	0,121	+inf.

Shift	0,002	(±	0,581	ns)
Decay Background	1.000 fixed	(±	0	)
IRF background	0,1			

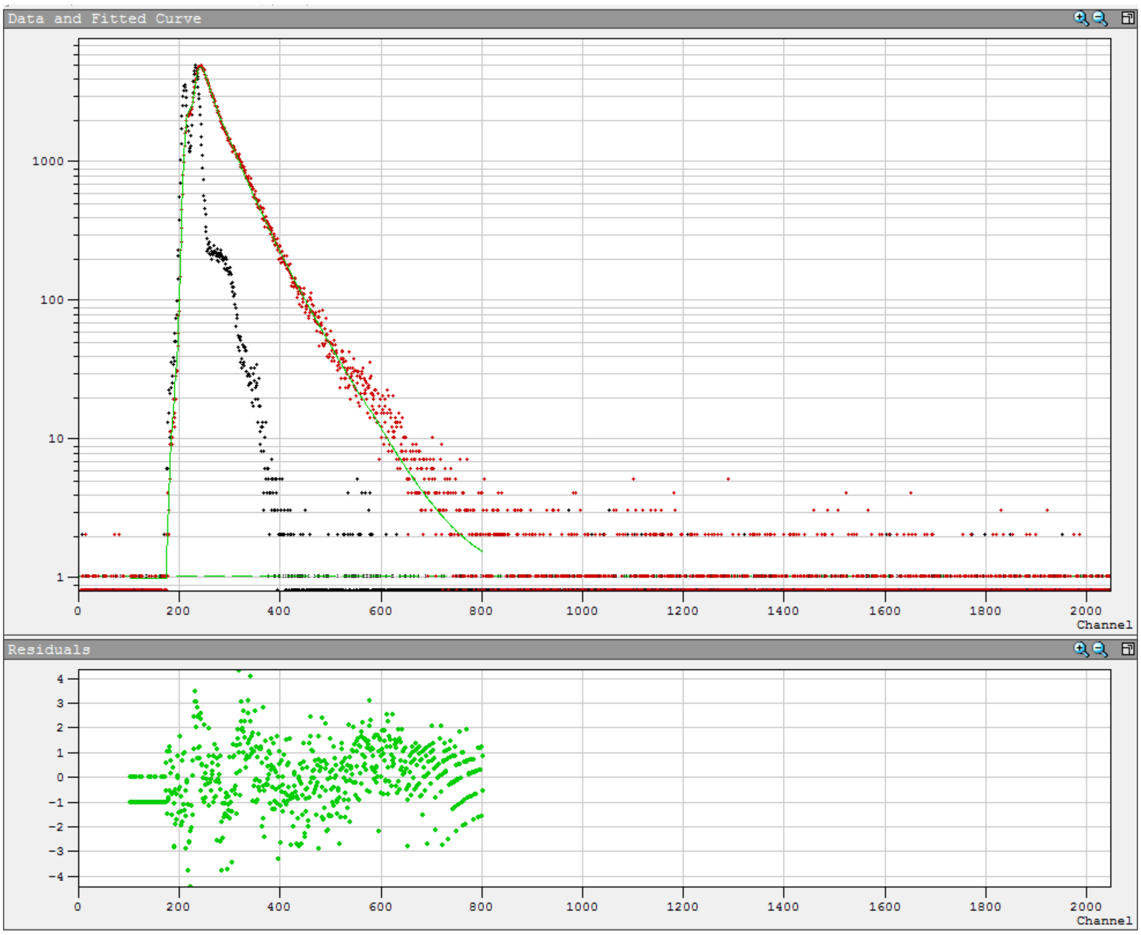

Fluorescence decay trace and reconvolution best fit of ***p*-PB** in 100% PEG-400.

Reconvolution parameters of ***p*-PB** in 100% PEG-400.

PhenQuin ACN 100 PEG Decay 10ns.FL

❖ **Exponential Components Analysis (Reconvolution)**

Fitting range [100; 800] channels

χ^2 1,6

	B_i	ΔB_i	f_i (%)	Δf_i (%)	τ_i (ns)	$\Delta\tau_i$ (ns)
**intensity-weighted lifetime**					**0,19**	
1	0,0251	0,0053	5,844	2107,365	0,033	11,883
2	0,0677	0,0044	55,402	199,724	0,116	0,41
3	0,0165	0,001	38,753	8,449	0,332	0,053

		Probability 60%		Probability 90%	
	τ_i (ns)	$Conf_{low}$	$Conf_{high}$	$Conf_{low}$	$Conf_{high}$
1	0,033	0,018	+inf.	0,013	+inf.
2	0,116	0,101	0,128	0,092	0,131
3	0,332	0,315	0,351	0,31	0,358

Shift 0,002 (± 0,184 ns)

Decay Background 1.000 fixed (± 0)

IRF background 0,1

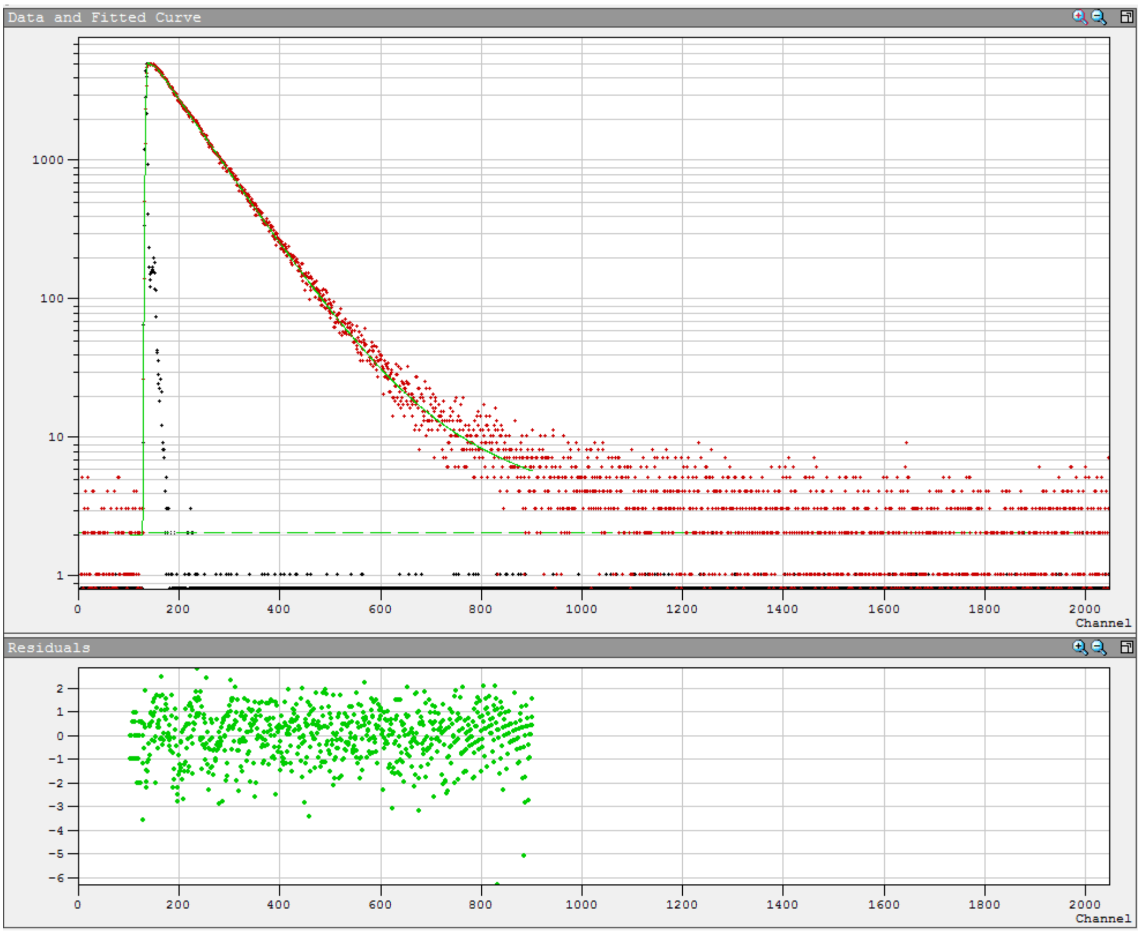

Fluorescence decay trace and reconvolution best fit of **pseudo-*m*-NB** in 1.63% PEG-400/CH_3CN.

Reconvolution parameters of **pseudo-*m*-NB** in 1.63% PEG-400/CH_3CN.

NaphZwit ACN 1_63 PEG Decay 50ns.FL

❖ Exponential Components Analysis (Reconvolution)

Fitting range [100; 900] channels

χ^2 1,19

	B_i	ΔB_i	f_i (%)	Δf_i (%)	τ_i (ns)	$\Delta\tau_i$ (ns)
**intensity-weighted lifetime**					**2,13**	
1	0,2489	0,0005	97,233	0,296	1,994	0,002
2	0,002	0,0005	2,767	0,72	6,902	0,018

		Probability 60%		Probability 90%	
	τ_i (ns)	Conf $_{low}$	Conf $_{high}$	Conf $_{low}$	Conf $_{high}$
1	1,994	1,97	2,012	1,959	2,017
2	6,902	5,103	10,782	4,621	+inf.

Shift	-0,003	(±	0,014	ns)
Decay Background	2.000 fixed	(±	0	)
IRF background	0			

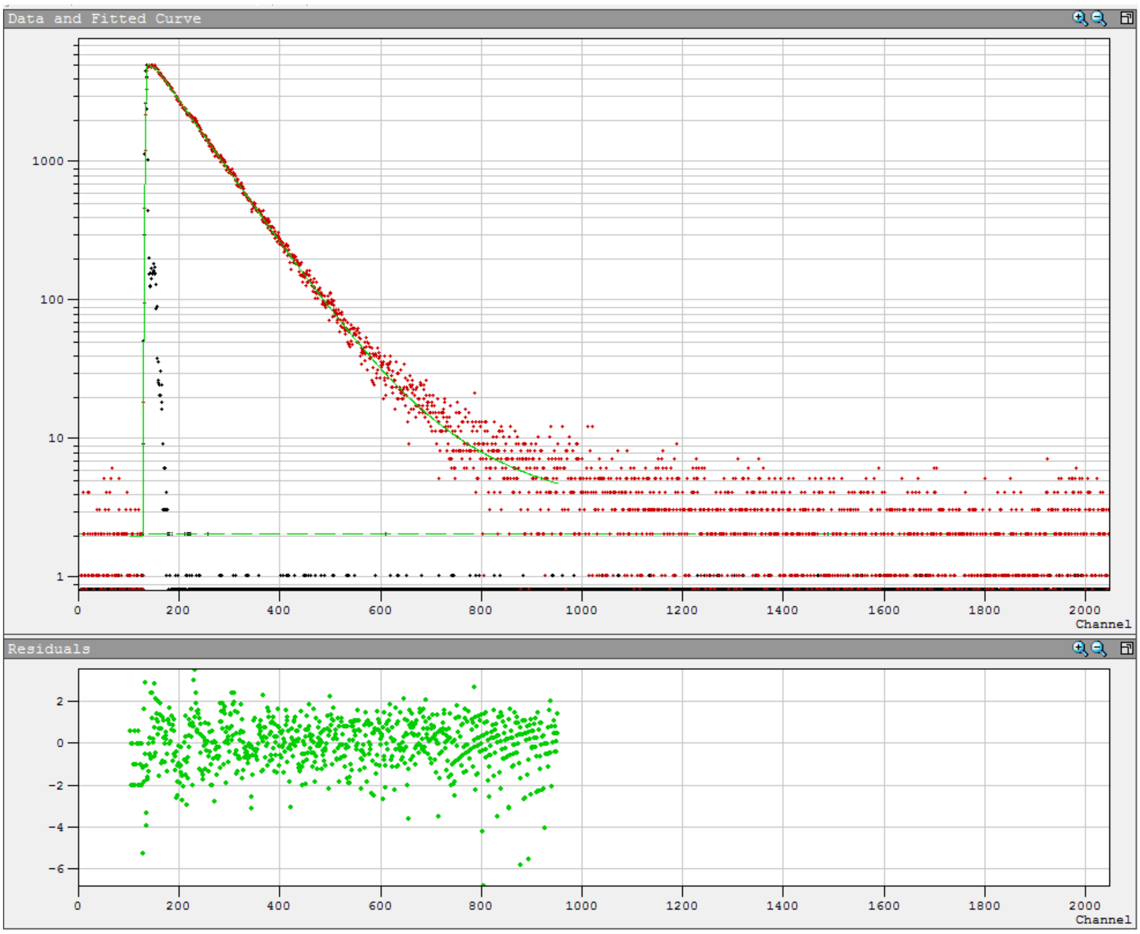

Fluorescence decay trace and reconvolution best fit of **pseudo-*m*-NB** in 3.60% PEG-400/CH_3CN.

Reconvolution parameters of **pseudo-*m*-NB** in 3.60% PEG-400/CH_3CN.

NaphZwit ACN 3_60 PEG Decay 50ns.FL

❖ Exponential Components Analysis (Reconvolution)

Fitting range [100; 950] channels

χ^2 1,474

	B_i	ΔB_i	f_i (%)	Δf_i (%)	τ_i (ns)	$\Delta\tau_i$ (ns)
**intensity-weighted lifetime**					**2,16**	
1	0,2438	0,0005	97,839	0,301	2,038	0,002
2	0,0015	0,0005	2,161	0,702	7,396	0,021

		Probability 60%		Probability 90%	
	τ_i (ns)	$Conf_{low}$	$Conf_{high}$	$Conf_{low}$	$Conf_{high}$
1	2,038	2,015	2,053	2,004	2,06
2	7,396	5,169	12,713	4,661	+inf.

Shift -0,002 (± 0,015 ns)

Decay Background 2.000 fixed (± 0)

IRF background 0

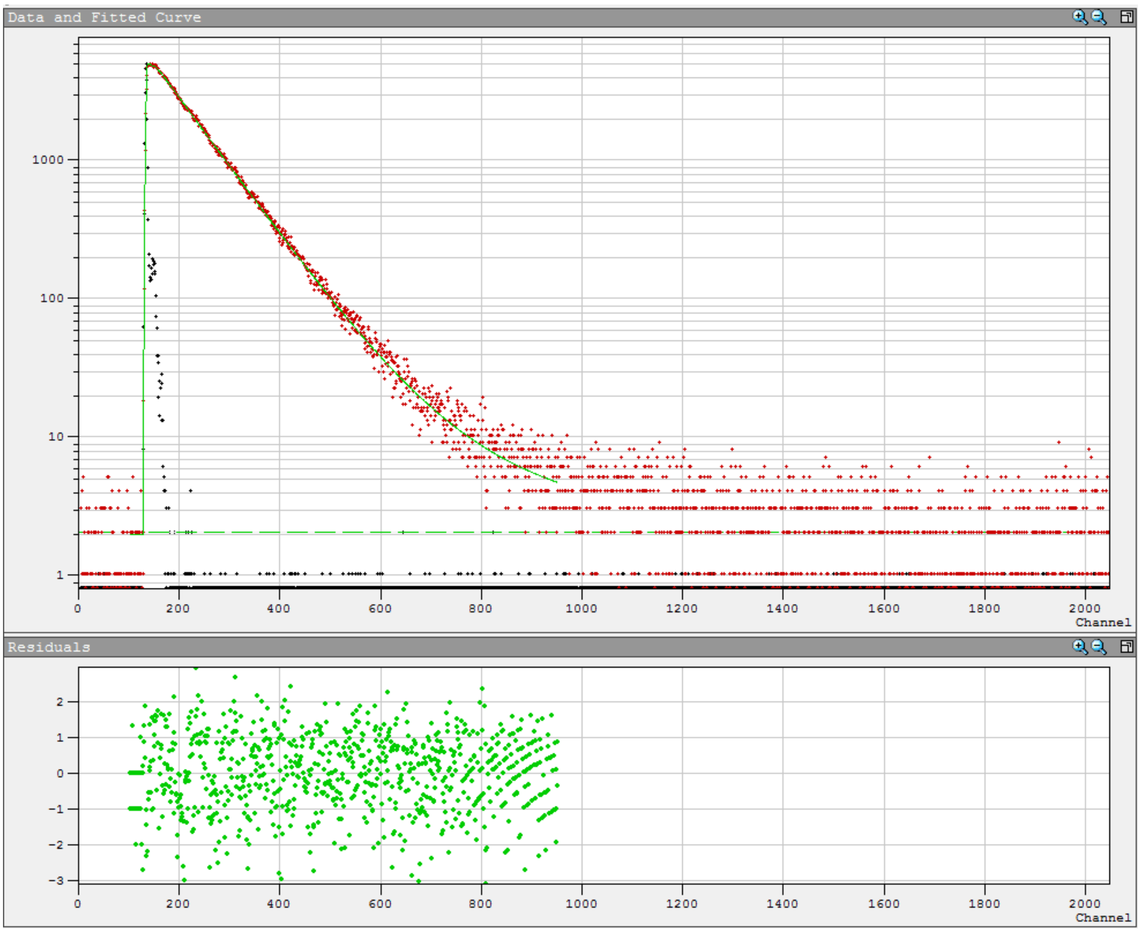

Fluorescence decay trace and reconvolution best fit of **pseudo-*m*-NB** in 9.05% PEG-400/CH_3CN.

Reconvolution parameters of **pseudo-*m*-NB** in 9.05% PEG-400/CH_3CN.

NaphZwit ACN 9_05 PEG Decay 50ns.FL

❖ Exponential Components Analysis (Reconvolution)

Fitting range [100; 950] channels

χ^2 1,117

	B_i	ΔB_i	f_i (%)	Δf_i (%)	τ_i (ns)	$\Delta\tau_i$ (ns)
**intensity-weighted lifetime**					**2,24**	
1	0,2438	0,0006	97,558	0,322	2,119	0,002
2	0,0019	0,0006	2,442	0,824	6,954	0,026

		Probability 60%		Probability 90%	
	τ_i (ns)	$Conf_{low}$	$Conf_{high}$	$Conf_{low}$	$Conf_{high}$
1	2,119	2,082	2,138	2,047	2,144
2	6,954	3,995	12,625	-inf.	+inf.

Shift	0,003	(±	0,019 ns)
Decay Background	1,647	(±	0,575)
IRF background	0,1		

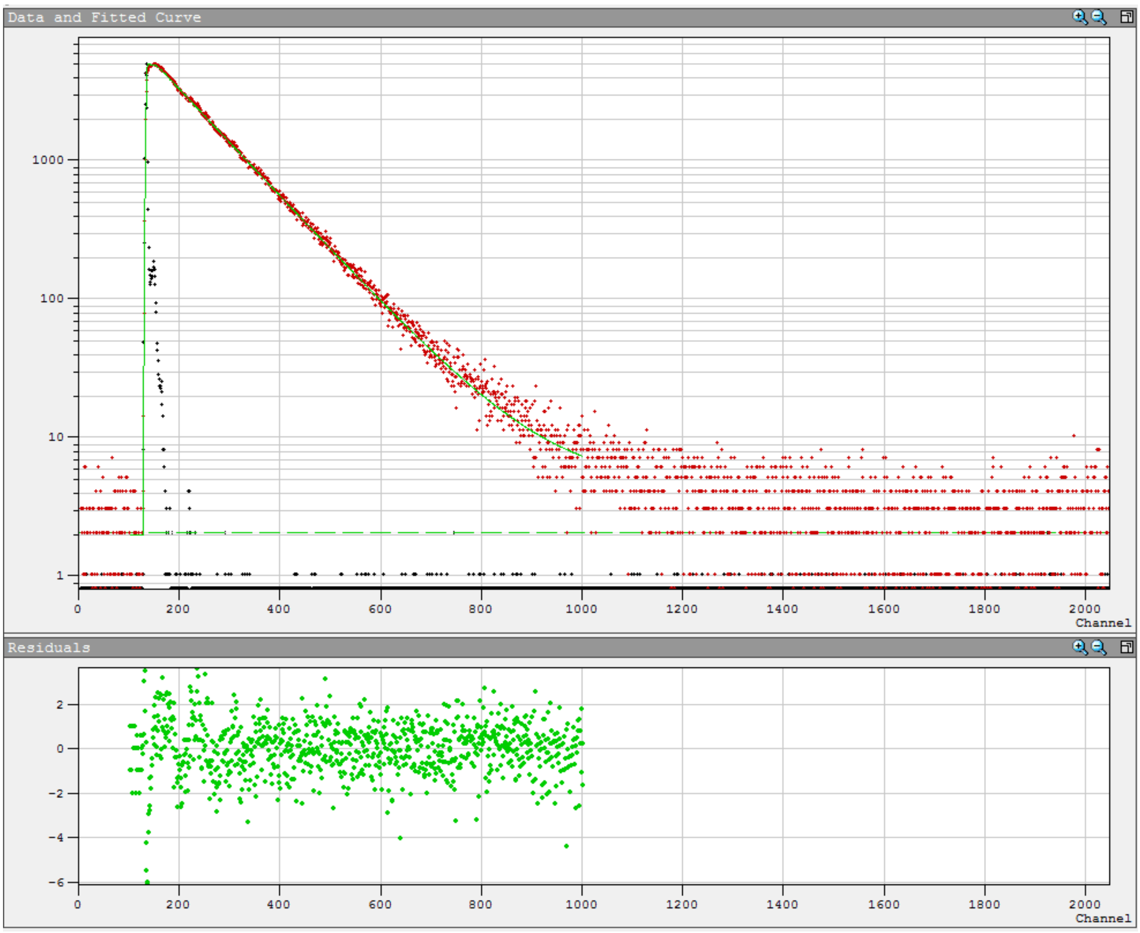

Fluorescence decay trace and reconvolution best fit of **pseudo-*m*-NB** in 37.39% PEG-400/CH_3CN.

Reconvolution parameters of **pseudo-*m*-NB** in 37.39% PEG-400/CH_3CN.

NaphZwit ACN 37_39 PEG Decay 50ns.FL

❖ **Exponential Components Analysis (Reconvolution)**

Fitting range [100; 1200] channels

χ^2 1,472

	B_i	ΔB_i	f_i (%)	Δf_i (%)	τ_i (ns)	$\Delta\tau_i$ (ns)
**intensity-weighted lifetime**					**2,95**	
1	0,2452	0,0005	99,036	0,232	2,743	0,0009
2	0,0002	0,0001	0,964	0,554	27,587	0,029

		Probability 60%		Probability 90%	
	τ_i (ns)	$Conf_{low}$	$Conf_{high}$	$Conf_{low}$	$Conf_{high}$
1	2,743	-inf.	2,75	-inf.	2,756
2	27,587	-inf.	+inf.	-inf.	+inf.

Shift -0,0003 (± 0,017 ns)

Decay Background 1,741 (± 0,762)

IRF background 0

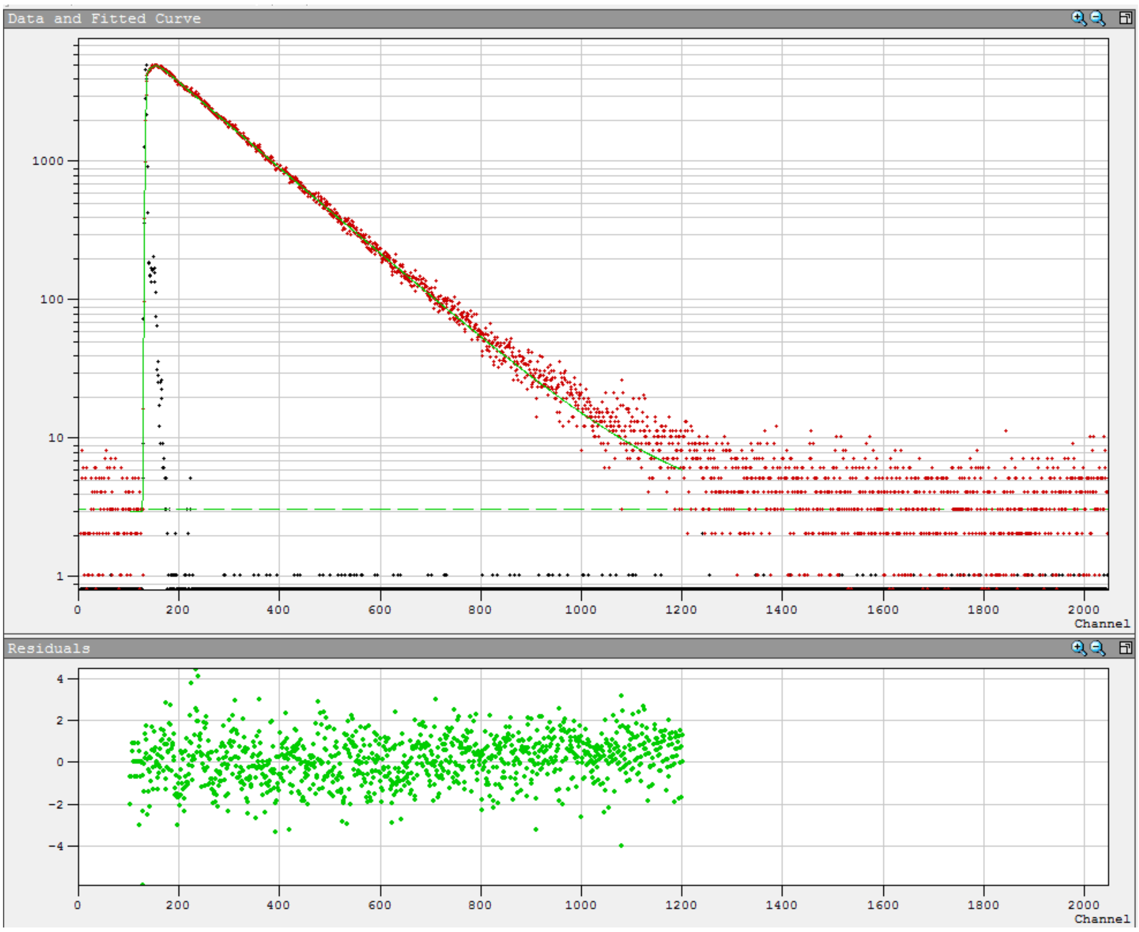

Fluorescence decay trace and reconvolution best fit of **pseudo-*m*-NB** in 57.33% PEG-400/CH_3CN.

Reconvolution parameters of **pseudo-*m*-NB** in 57.33% PEG-400/CH_3CN.

NaphZwit ACN 57_33 PEG Decay 50ns.FL

❖ Exponential Components Analysis (Reconvolution)

Fitting range [100; 1200] channels

χ^2 1,291

	B_i	ΔB_i	f_i (%)	Δf_i (%)	τ_i (ns)	$\Delta\tau_i$ (ns)
**intensity-weighted lifetime**					**3,38**	
1	0,0481	0,0032	1,288	-2,127	0,227	0,389
2	0,2442	0,0006	98,712	0,236	3,422	0,0004

		Probability 60%		Probability 90%	
	τ_i (ns)	Conf $_{low}$	Conf $_{high}$	Conf $_{low}$	Conf $_{high}$
1	0,227	0,19	0,269	0,173	0,299
2	3,422	3,417	3,427	3,413	3,431

Shift -0,002 (± 0,023 ns)

Decay Background 3.000 fixed (± 0)

IRF background 0

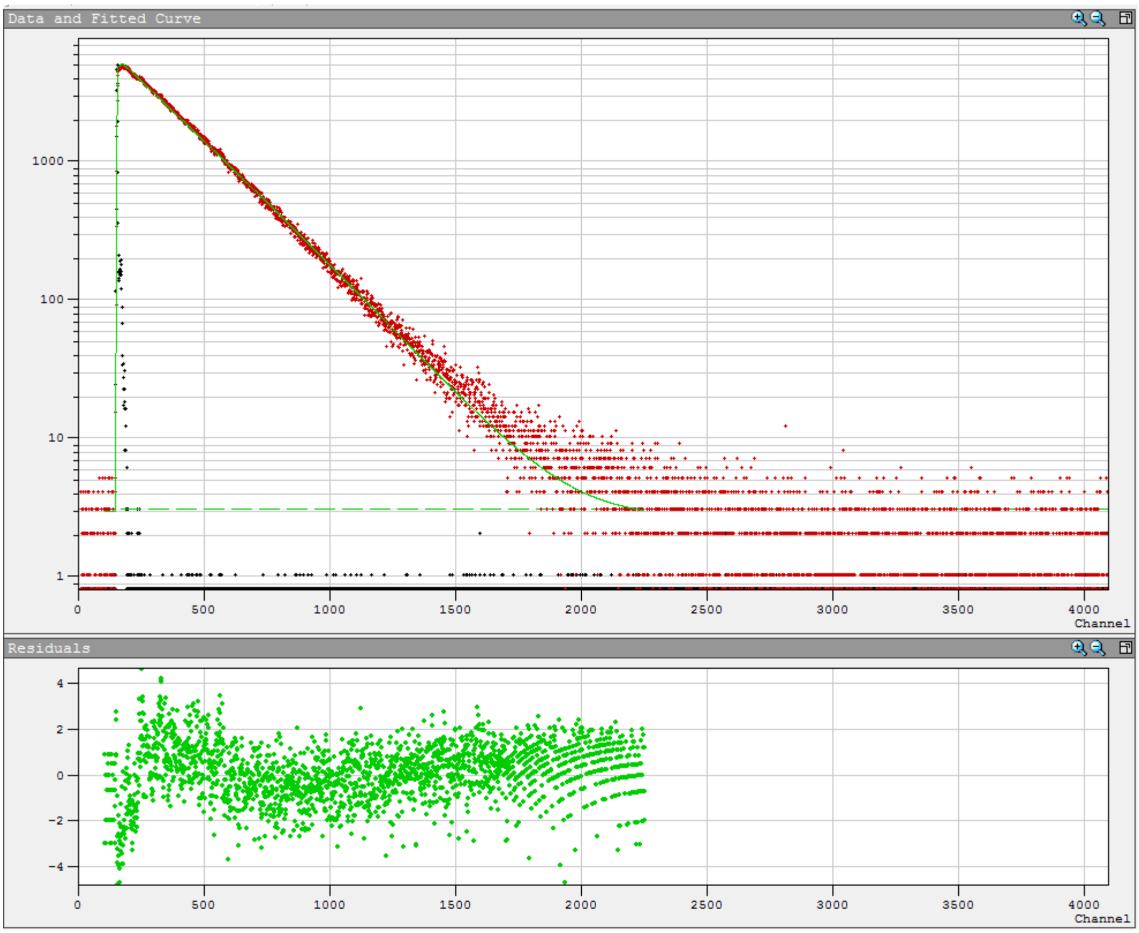

Fluorescence decay trace and reconvolution best fit of **pseudo-*m*-NB** in 100% PEG-400.

Reconvolution parameters of **pseudo-*m*-NB** in 100% PEG-400.

NaphZwit ACN 100 PEG Decay 50ns.FL

❖ Exponential Components Analysis (Reconvolution)

Fitting range [100; 2250] channels

χ^2 1,592

	B_i	ΔB_i	f_i (%)	Δf_i (%)	τ_i (ns)	$\Delta\tau_i$ (ns)
**intensity-weighted lifetime**					**7,02**	
1	0,7779	1,0448	57,418	77,162	6,875	0,005
2	0,5503	1,0449	42,582	80,818	7,207	0,006

		Probability 60%		Probability 90%	
	τ_i (ns)	Conf $_{low}$	Conf $_{high}$	Conf $_{low}$	Conf $_{high}$
1	6,875	6,7	7,066	6,618	+inf.
2	7,207	-inf.	7,502	-inf.	7,569

Shift 0,013 (± 0,025 ns)

Decay Background 3.000 fixed (± 0)

IRF background 0

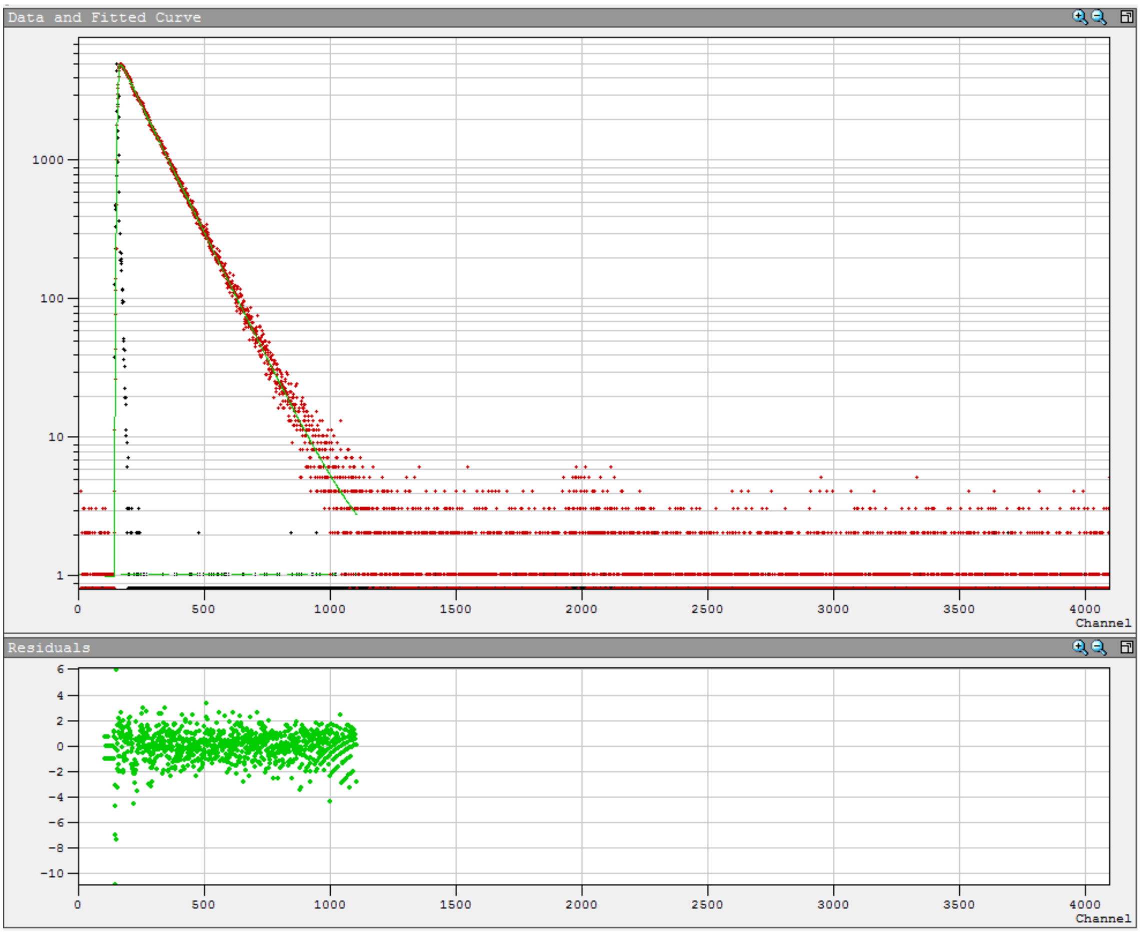

Fluorescence decay trace and reconvolution best fit of **pseudo-*p*-NB** in 1.63% PEG-400/CH_3CN.

Reconvolution parameters of **pseudo-*p*-NB** in 1.63% PEG-400/CH_3CN.

NaphQuin ACN 1_63 PEG Decay 100ns.FL

❖ **Exponential Components Analysis (Reconvolution)**

Fitting range [100; 1100] channels

χ^2 1,534

	B_i	ΔB_i	f_i (%)	Δf_i (%)	τ_i (ns)	$\Delta\tau_i$ (ns)
**intensity-weighted lifetime**					**2,86**	
1	0,0083	0,0018	1,774	0,897	1,009	0,295
2	0,1607	0,002	98,226	1,262	2,889	0,002

		Probability 60%		Probability 90%	
	τ_i (ns)	Conf $_{low}$	Conf $_{high}$	Conf $_{low}$	Conf $_{high}$
1	1,009	0,557	+inf.	-inf.	+inf.
2	2,889	2,873	2,944	2,86	3,103

Shift 0,026 (± 0,041 ns)

Decay Background 1.000 fixed (± 0)

IRF background 0,1

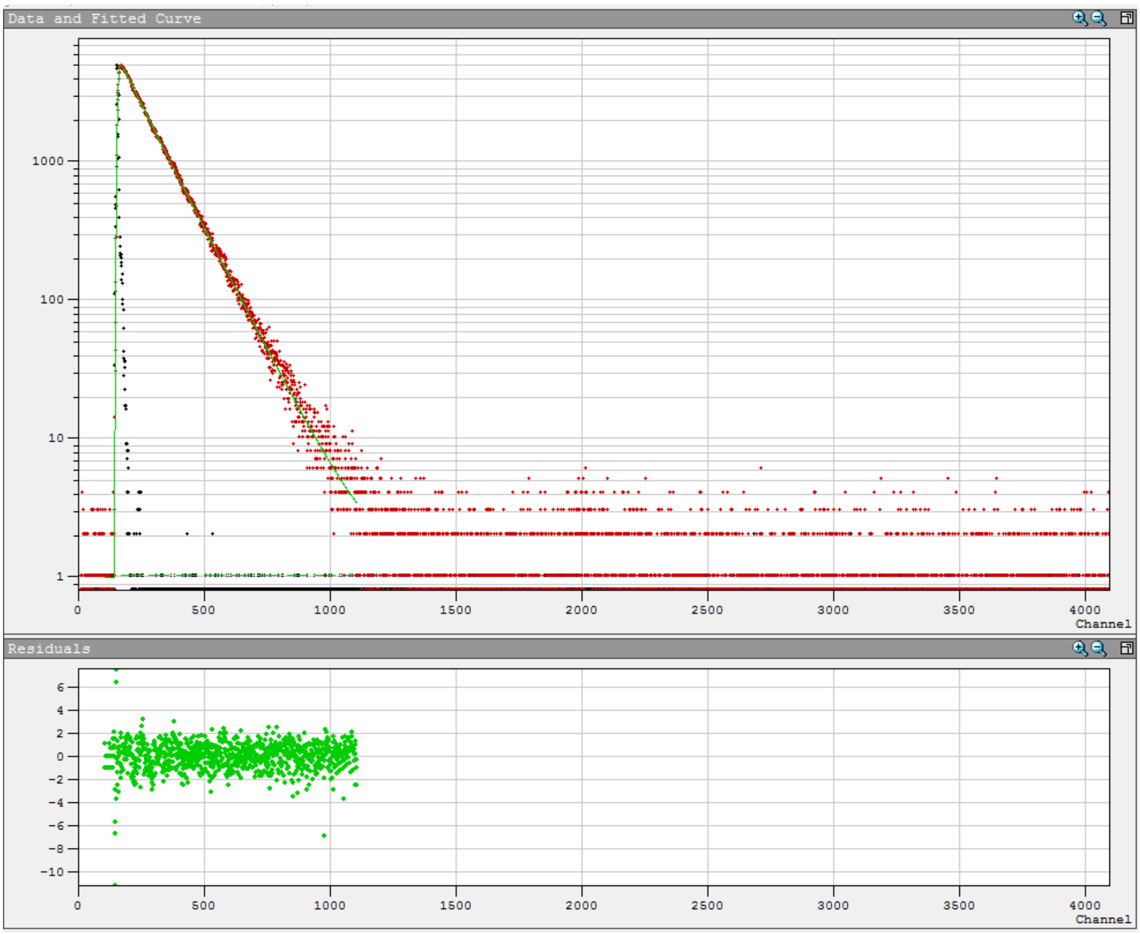

Fluorescence decay trace and reconvolution best fit of **pseudo-*p*-NB** in 3.60% PEG-400/CH_3CN.

Reconvolution parameters of **pseudo-*p*-NB** in 3.60% PEG-400/CH_3CN.

NaphQuin ACN 3_60 PEG Decay 100ns.FL

❖ Exponential Components Analysis (Reconvolution)

Fitting range [100; 1100] channels

χ^2 1,503

	B_i	ΔB_i	f_i (%)	Δf_i (%)	τ_i (ns)	$\Delta\tau_i$ (ns)
**intensity-weighted lifetime**					**2,96**	
1	0,0115	0,0035	3,39	1,418	1,394	0,165
2	0,1517	0,0038	96,61	2,473	3,014	0,002

		Probability 60%		Probability 90%	
	τ_i (ns)	Conf $_{low}$	Conf $_{high}$	Conf $_{low}$	Conf $_{high}$
1	1,394	0,834	+inf.	-inf.	+inf.
2	3,014	2,982	3,139	2,976	3,497

Shift 0,028 (± 0,038 ns)

Decay Background 1.000 fixed (± 0)

IRF background 0,1

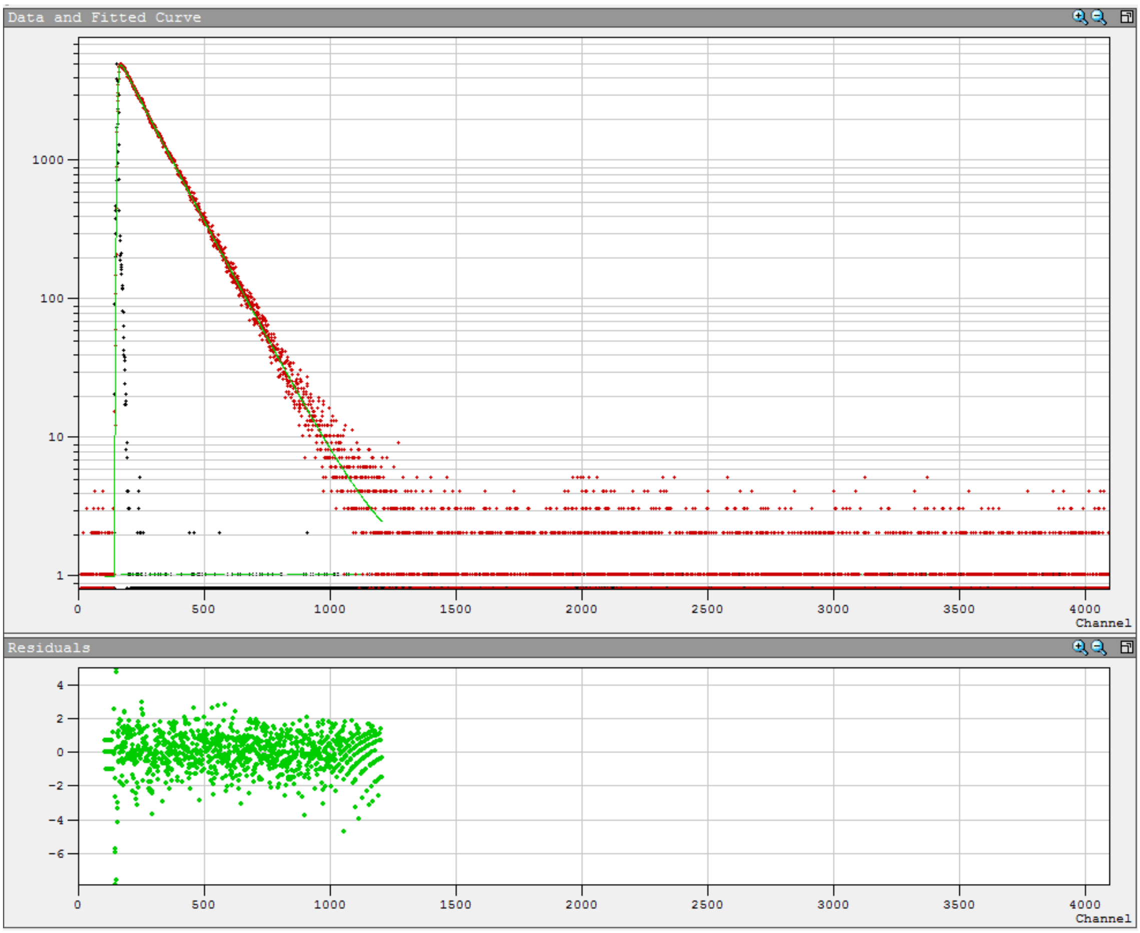

Fluorescence decay trace and reconvolution best fit of **pseudo-*p*-NB** in 9.05% PEG-400/CH_3CN.

Reconvolution parameters of **pseudo-*p*-NB** in 9.05% PEG-400/CH_3CN.

NaphQuin ACN 9_05 PEG Decay 100ns.FL

❖ Exponential Components Analysis (Reconvolution)

Fitting range [100; 1200] channels

χ^2 1,377

	B_i	ΔB_i	f_i (%)	Δf_i (%)	τ_i (ns)	$\Delta\tau_i$ (ns)
**intensity-weighted lifetime**					**3,07**	
1	0,0123	0,0036	3,549	1,342	1,491	0,131
2	0,1584	0,0038	96,451	2,397	3,132	0,002

		Probability 60%		Probability 90%	
	τ_i (ns)	Conf $_{low}$	Conf $_{high}$	Conf $_{low}$	Conf $_{high}$
1	1,491	0,998	+inf.	0,847	+inf.
2	3,132	3,101	3,215	3,095	3,352

Shift 0,023 (± 0,027 ns)

Decay Background 1.000 fixed (± 0)

IRF background 0

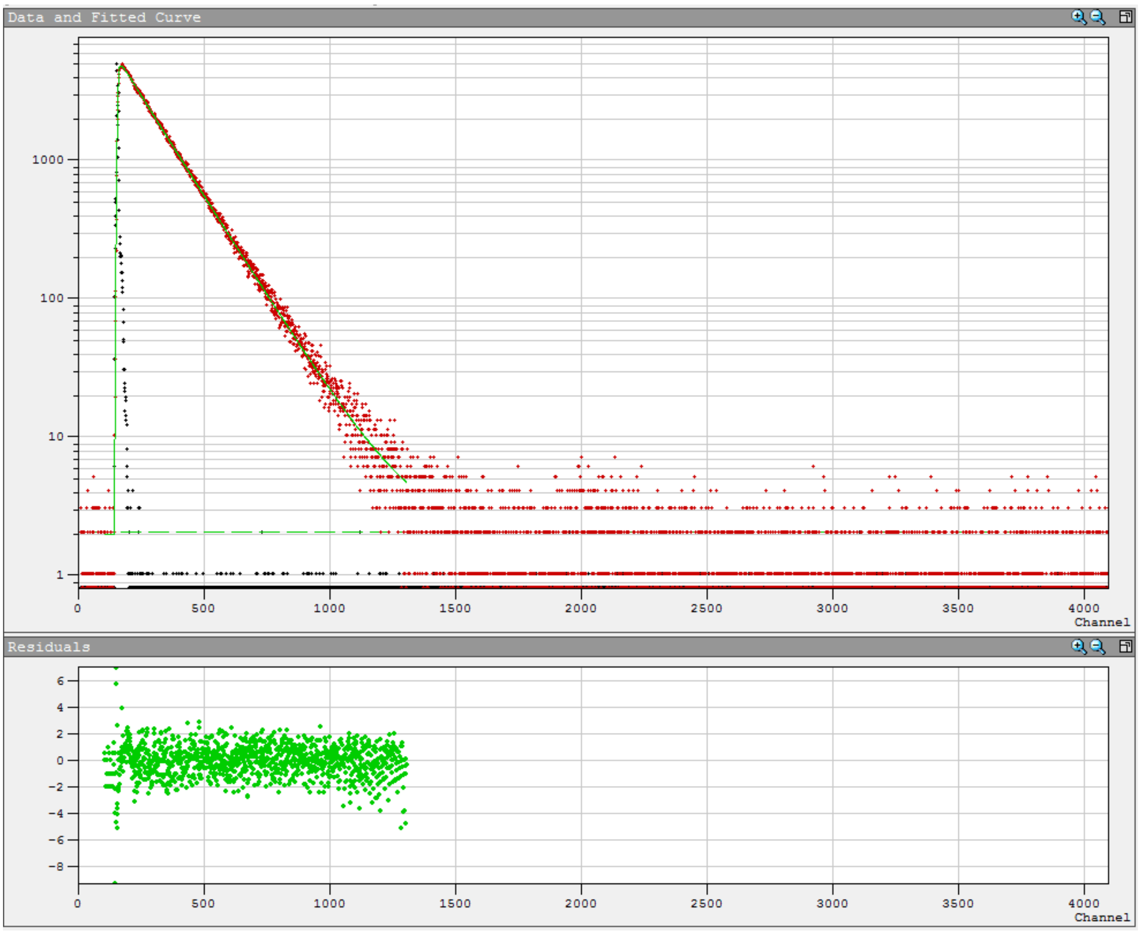

Fluorescence decay trace and reconvolution best fit of **pseudo-*p*-NB** in 37.39% PEG-400/CH_3CN.

Reconvolution parameters of **pseudo-*p*-NB** in 37.39% PEG-400/CH_3CN.

NaphQuin ACN 37_39 PEG Decay 100ns.FL

❖ **Exponential Components Analysis (Reconvolution)**

Fitting range [100; 1300] channels

χ^2 1,541

	B_i	ΔB_i	f_i (%)	Δf_i (%)	τ_i (ns)	$\Delta\tau_i$ (ns)
**intensity-weighted lifetime**					**3,66**	
1	0,0089	0,0163	3,782	7,221	2,417	0,198
2	0,1477	0,0166	96,218	10,929	3,712	0,005

		Probability 60%		Probability 90%	
	τ_i (ns)	Conf $_{low}$	Conf $_{high}$	Conf $_{low}$	Conf $_{high}$
1	2,417	-inf.	+inf.	-inf.	+inf.
2	3,712	-inf.	6,595	-inf.	+inf.

Shift 0,036 (± 0,034 ns)

Decay Background 2.000 fixed (± 0)

IRF background 0,1

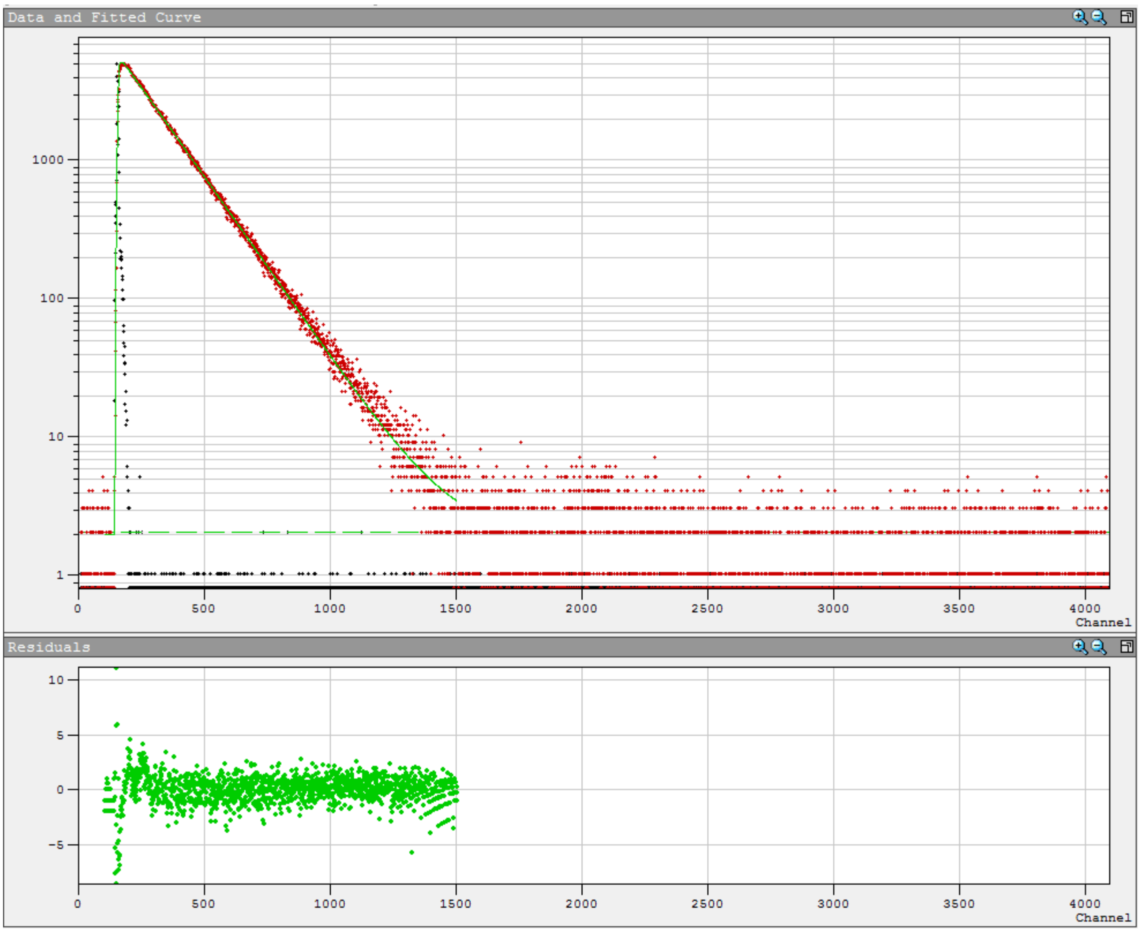

Fluorescence decay trace and reconvolution best fit of **pseudo-*p*-NB** in 57.33% PEG-400/CH_3CN.

Reconvolution parameters of **pseudo-*p*-NB** in 57.33% PEG-400/CH_3CN.

NaphQuin ACN 57_33 PEG Decay 100ns.FL

❖ Exponential Components Analysis (Reconvolution)

Fitting range [100; 1500] channels

χ^2 1,898

	B_i	ΔB_i	f_i (%)	Δf_i (%)	τ_i (ns)	$\Delta\tau_i$ (ns)
**intensity-weighted lifetime**					**4,49**	
1	0,2775	1,1022	69,225	275,573	4,384	0,039
2	0,114	1,1024	30,775	297,059	4,744	0,081

		Probability 60%		Probability 90%	
	τ_i (ns)	$Conf_{low}$	$Conf_{high}$	$Conf_{low}$	$Conf_{high}$
1	4,384	4,18	4,475	4,156	4,477
2	4,744	-inf.	6,549	-inf.	+inf.

Shift 0,041 (± 0,042 ns)

Decay Background 2.000 fixed (± 0)

IRF background 0,1

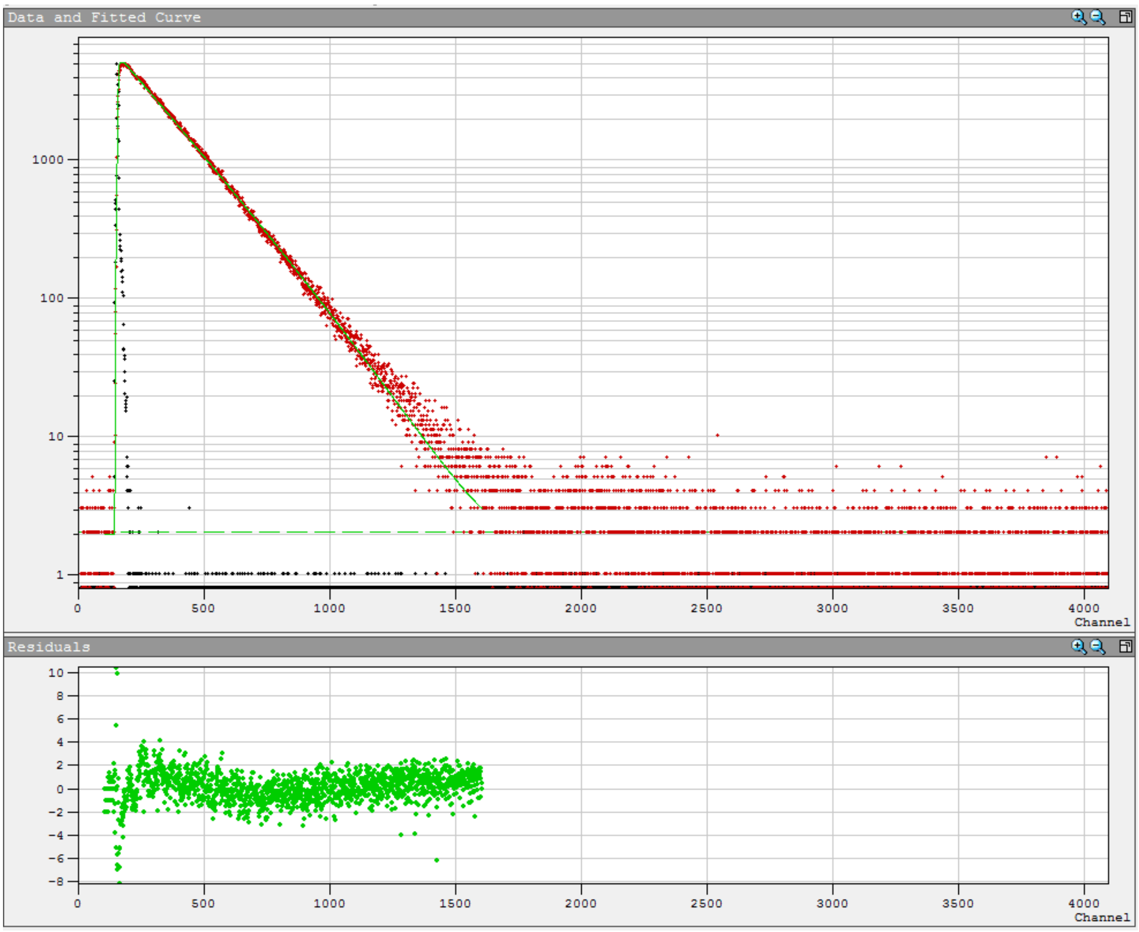

Fluorescence decay trace and reconvolution best fit of **pseudo-*p*-NB** in 100% PEG-400.

Reconvolution parameters of **pseudo-*p*-NB** in 100% PEG-400.

NaphQuin ACN 100 PEG Decay 100ns.FL

❖ **Exponential Components Analysis (Reconvolution)**

Fitting range	[100; 1600] channels
χ^2	1,868

	B_i	ΔB_i	f_i (%)	Δf_i (%)	τ_i (ns)	$\Delta\tau_i$ (ns)
**intensity-weighted lifetime**					**5,78**	
1	0,5684	1,3531	57,058	135,931	5,653	0,011
2	0,4068	1,3531	42,942	142,729	5,945	0,014

		Probability 60%		Probability 90%	
	τ_i (ns)	Conf $_{low}$	Conf $_{high}$	Conf $_{low}$	Conf $_{high}$
1	5,653	5,462	5,768	5,378	5,77
2	5,945	-inf.	6,243	-inf.	6,245

Shift	0,054	(±	0,047	ns)
Decay Background	2.000 fixed	(±	0	)
IRF background	0			

Section S18 Oxygen-dependent Photophysics

Emission spectra were recorded both in ambient oxygenated acetonitrile and solvent which had been degassed by freeze-pump-thaw cycles prior to use.

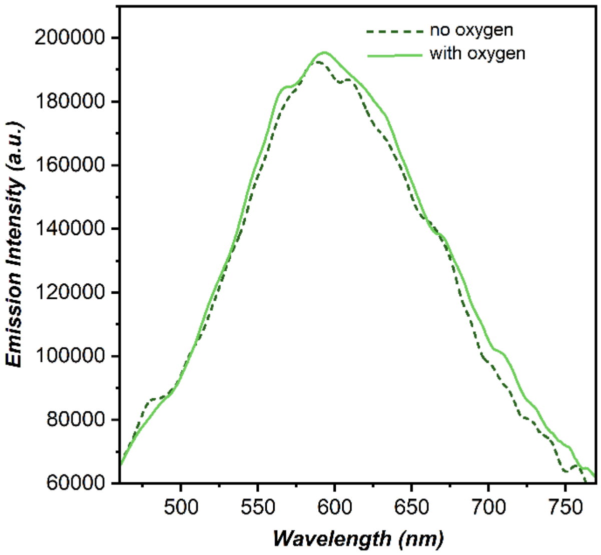

Figure S167. Emission spectra of oxygenated (solid) and deoxygenated (dash) acetonitrile solutions of ***m*-PB**, λ_{exc} 400 nm.

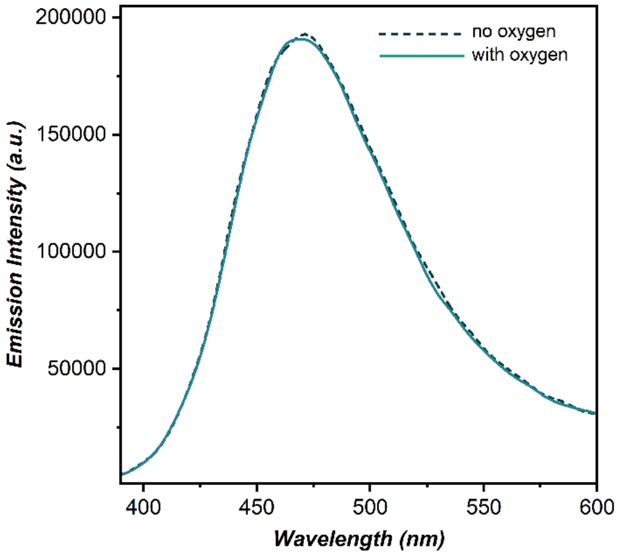

Figure S168. Emission spectra of oxygenated (solid) and deoxygenated (dash) acetonitrile solutions of ***p*-PB**, λ_{exc} 370 nm.

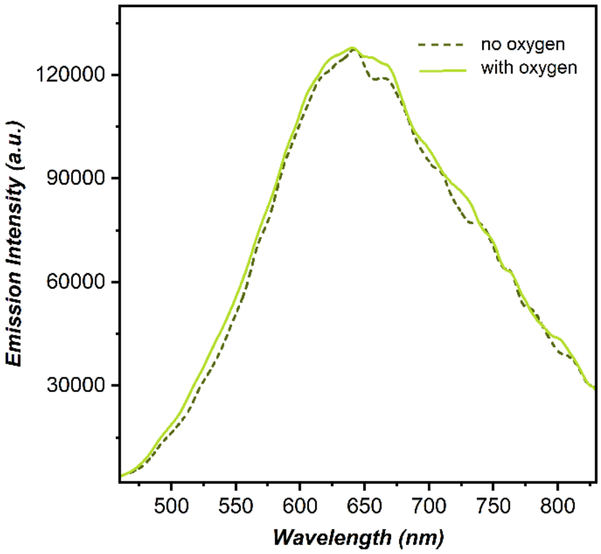

Figure S169. Emission spectra of oxygenated (solid) and deoxygenated (dash) acetonitrile solutions of **pseudo-*m*-NB**, λ_{exc} 430 nm.

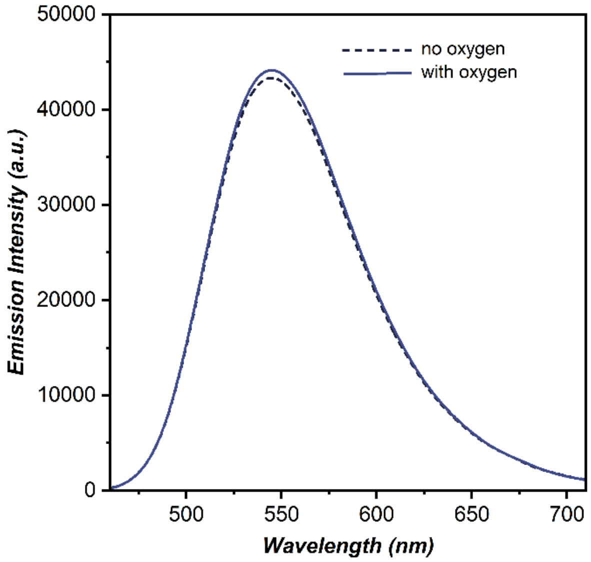

Figure S170. Emission spectra of oxygenated (solid) and deoxygenated (dash) acetonitrile solutions of **pseudo-*p*-NB**, λ_{exc} 420 nm.

Section S19 Viscosity-dependent Photophysics

Table S35. Collected properties of pure and mixed solvent systems.

solvent	Dynamic viscosity, η (kg · m^{-1} · s^{-1})[75]	mol fraction PEG-400	Measured refractive index, n_D^{20}
acetone	0.30	-	1.359
acetonitrile	0.34	-	1.344
dimethylformamide	0.81	-	1.430
dimethylsulfoxide	1.99	-	1.479
formamide	3.30	-	1.446
benzonitrile	1.16	-	1.528
1.63% PEG-400/CH_3CN	1.14	0.00243	1.345
3.60% PEG-400/CH_3CN	2.17	0.00546	1.348
9.05% PEG-400/CH_3CN	5.87	0.0144	1.356
37.39% PEG-400/CH_3CN	22.52	0.0808	1.394
57.33% PEG-400/CH_3CN	33.63	0.165	1.421
100% PEG-400	55.84	100	1.464

Table S36. Fluorescence quantum yield of compounds in mixtures of varying viscosity.

Compound	Percentage PEG-400 in CH_3CN (%)	Fluorescence Quantum Yield, Φ_F
***m*-PB**	0.00	0.01(4) ± 0.01
	1.63	0.01(3) ± 0.01
	3.60	0.01(2) ± 0.01
	9.05	0.01(4) ± 0.01
	37.39	0.01(9) ± 0.01
	57.33	0.02(9) ± 0.01
	100.0	0.06(4) ± 0.01
***p*-PB**	0.00	< 0.01 (0.00059)
	1.63	< 0.01 (0.0006)
	3.60	< 0.01 (0.0007)
	9.05	< 0.01 (0.0009)
	37.39	< 0.01 (0.0019)
	57.33	< 0.01 (0.0038)
	100.0	0.04(0) ± 0.01
**pseudo-*m*-NB**	0.00	0.02(3) ± 0.01
	1.63	0.02(3) ± 0.01
	3.60	0.02(4) ± 0.01
	9.05	0.02(7) ± 0.01
	37.39	0.03(7) ± 0.01
	57.33	0.04(8) ± 0.01
	100.0	0.09(0) ± 0.01
**pseudo-*p*-NB** *determined using an integrating sphere	0.00	0.469 ± 0.023*
	1.63	0.498 ± 0.025*
	3.60	0.520 ± 0.026*
	9.05	0.550 ±0.028*
	37.39	0.737 ± 0.037*
	57.33	0.816 ± 0.041*
	100.0	0.942 ± 0.047*

Intensity-weighted lifetimes were calculated using the individual intensities (B_i) and the lifetimes (τ_i) of the components obtained from reconvolution fitting:

$$\tau_{avg} = \frac{\sum(B_i \times \tau_i^2)}{\sum(B_i \times \tau_i)}$$

Radiative rate constants (in seconds) were calculated using the intensity-weighted lifetimes:

$$k_r(s) = \frac{1\,000\,000\,000}{\tau_{avg}}$$

Non-radiative rate constants (in seconds) were calculated using the fluorescence quantum yields and the radiative rate constants:

$$\Phi_F = \frac{k_r}{k_r + k_{nr}}$$

Table S37. Lifetimes and rate constants of compounds in solvents of varying viscosity.

Compound	Percentage PEG-400 in CH_3CN (%)	Intensity-weighted Lifetime, τ (ns)	Radiative Rate Constant, k_R (s^{-1})	Non-radiative Rate Constant, k_{NR} (s^{-1})
	1.63	1.32	7.58 E+08	5.86 E+10
	3.60	1.37	7.28 E+08	5.79 E+10
***m*-PB**	9.05	1.43	6.99 E+08	4.97 E+10
	37.39	1.82	5.51 E+08	2.81 E+10
	57.33	2.32	4.31 E+08	1.43 E+10
	100.0	4.50	2.22 E+08	3.23 E+09
	1.63	0.02	4.74 E+10	7.31 E+13
	3.60	0.03	3.48 E+10	4.97 E+13
***p*-PB**	9.05	0.09	1.10 E+10	1.20 E+13
	37.39	0.03	3.17 E+10	1.62 E+13
	57.33	004	2.48 E+10	6.46 E+12
	100.0	0.19	5.14 E+09	1.24 E+11
	1.63	2.13	4.70 E+08	1.98 E+10
	3.60	2.16	4.64 E+08	1.87 E+10
**pseudo-*m*-NB**	9.05	2.24	4.47 E+08	1.61 E+10
	37.39	2.95	3.40 E+08	8.86 E+09
	57.33	3.38	2.96 E+08	5.86 E+09
	100.0	7.02	1.43 E+08	1.44 E+09
	1.63	2.86	3.50 E+08	3.54 E+08
	3.60	2.96	3.38 E+08	3.11 E+08
**pseudo-*p*-NB**	9.05	3.07	3.25 E+08	2.66 E+08
	37.39	3.66	2.73 E+08	9.76 E+07
	57.33	4.49	2.22 E+08	5.03 E+07
	100.0	5.78	1.73 E+08	1.06 E+07

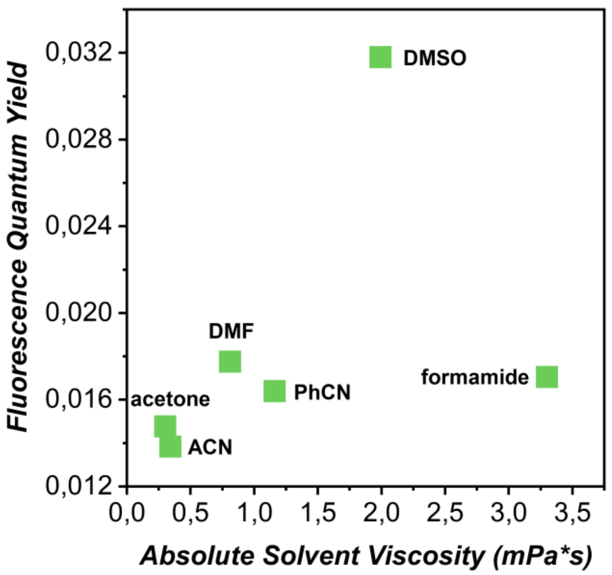

Figure S171. Plot of solvent viscosity versus fluorescence quantum yields for solutions of ***m*-PB**.

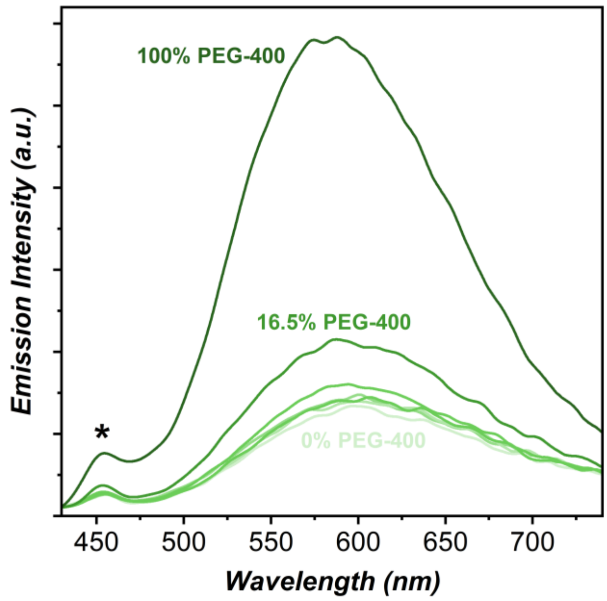

Figure S172. Fluorescence spectra of ***m*-PB** in CH_3CN from 400 nm excitation with increasing mol fraction of PEG-400. (* CH_3CN Raman peak)

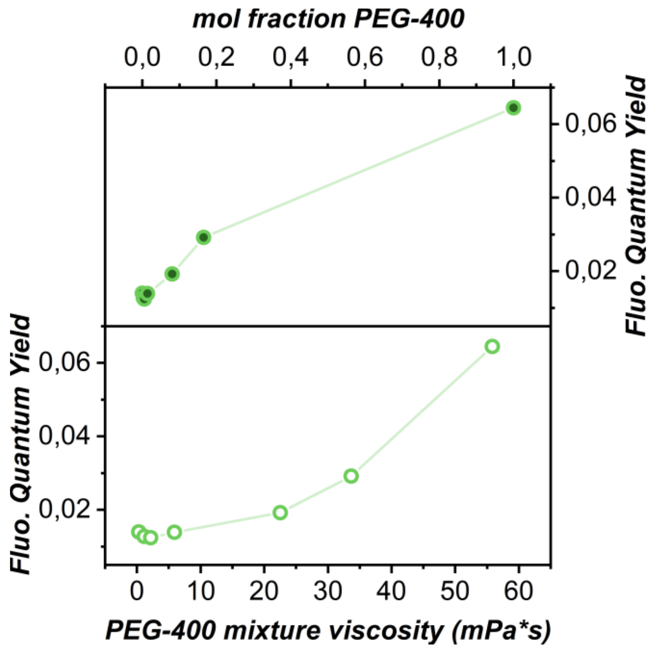

Figure S173. Plots of PEG-400 mol fraction and mixture viscosity versus fluorescence quantum yield for solutions of ***m*-NB** in CH_3CN.

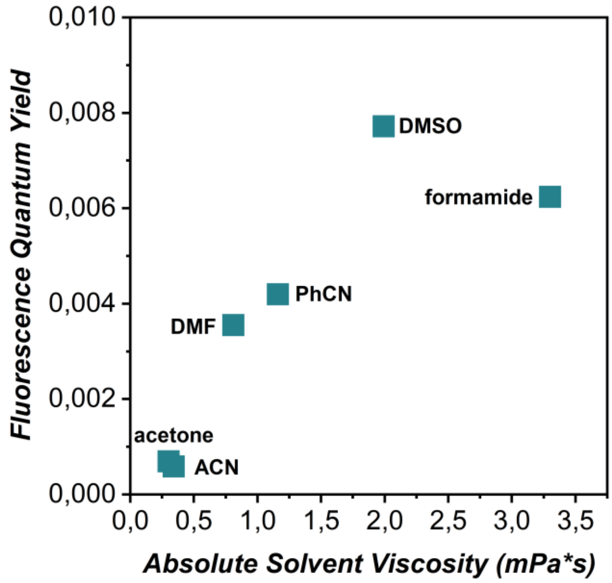

Figure S174. Plot of solvent viscosity versus fluorescence quantum yields for solutions of ***p*-PB**.

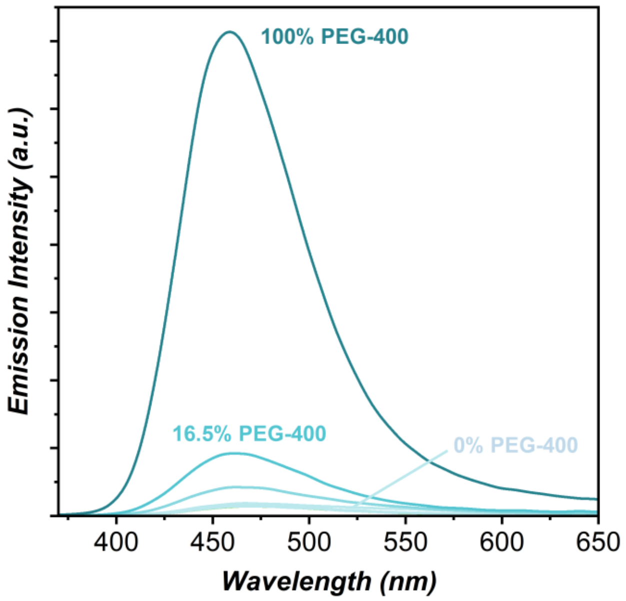

Figure S175. Fluorescence spectra of ***p*-PB** in CH_3CN from 350 nm excitation with increasing mol fraction of PEG-400.

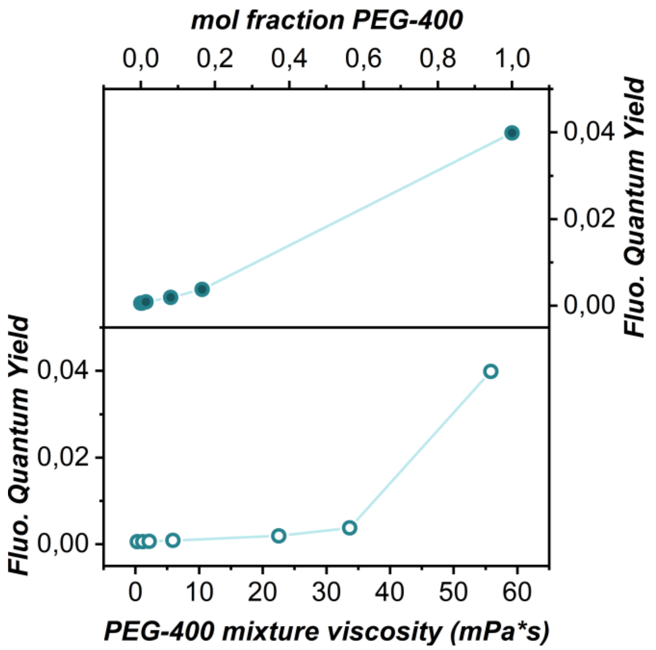

Figure S176. Plots of PEG-400 mol fraction and mixture viscosity versus fluorescence quantum yield for solutions of ***p*-NB** in CH_3CN.

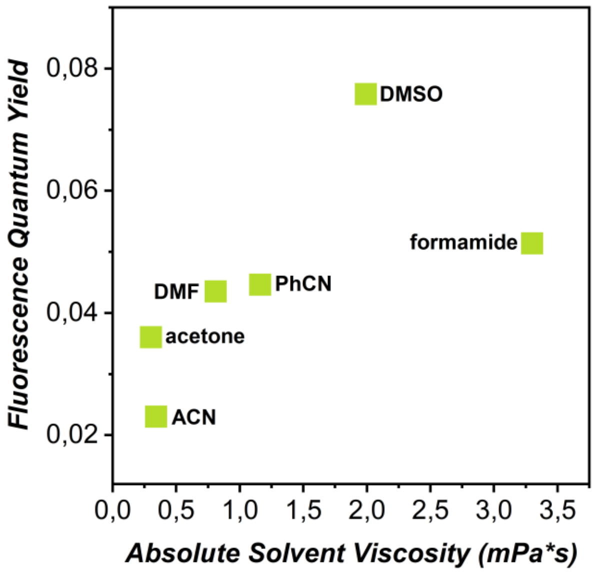

Figure S177. Plot of solvent viscosity versus fluorescence quantum yields for solutions of **pseudo-*m*-NB**.

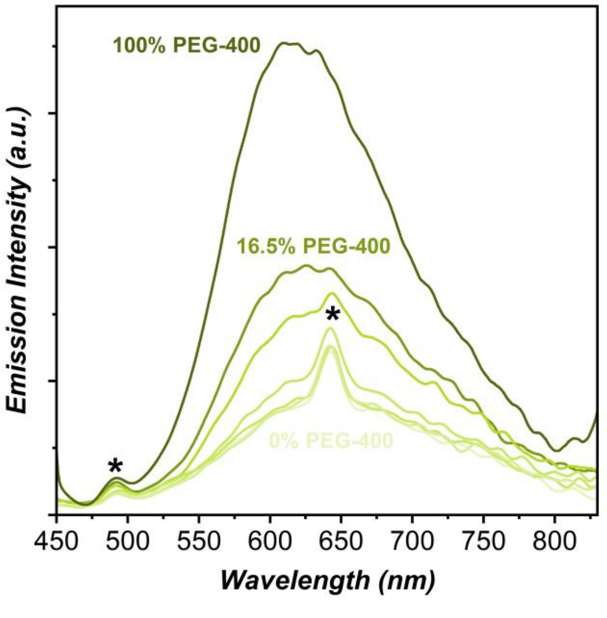

Figure S178. Fluorescence spectra of **pseudo-*m*-NB** in CH_3CN from 430 nm excitation with increasing mol fraction of PEG-400. (* CH_3CN Raman peak)

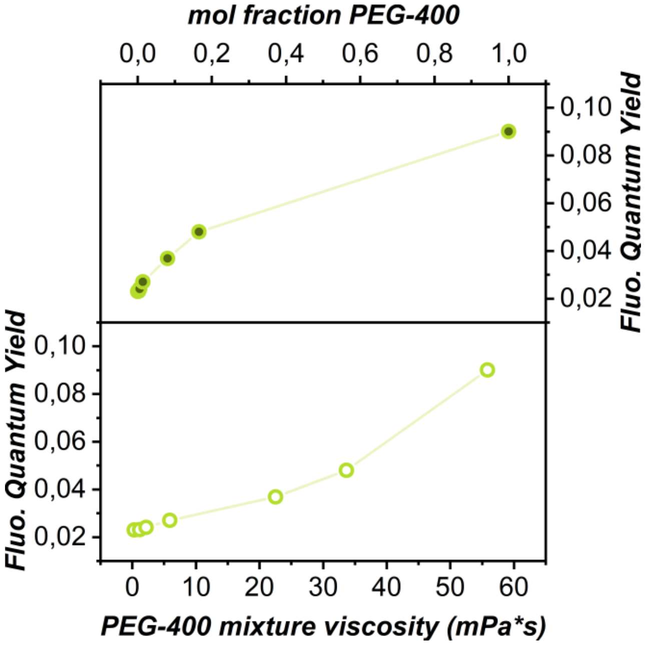

Figure S179. Plots of PEG-400 mol fraction and mixture viscosity versus fluorescence quantum yield for solutions of **pseudo-*m*-NB** in CH_3CN.

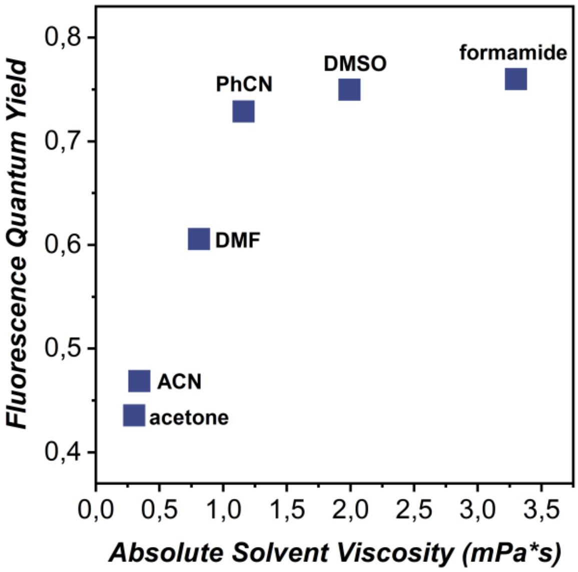

Figure S180. Plot of solvent viscosity versus fluorescence quantum yields for solutions of **pseudo-*p*-NB**.

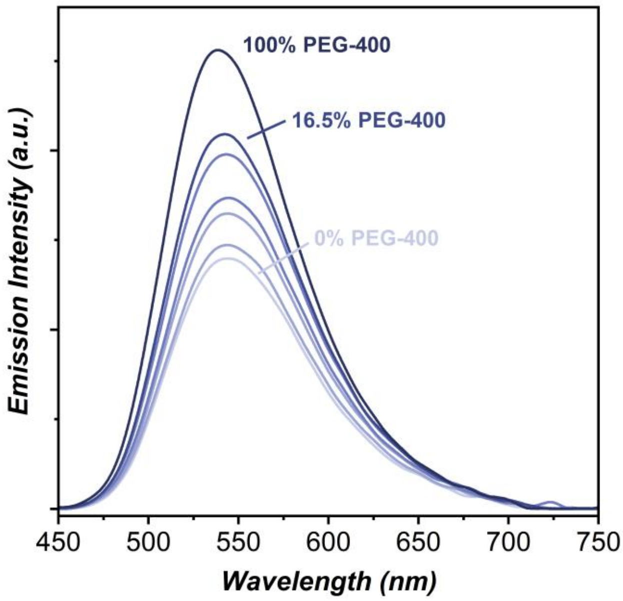

Figure S181. Fluorescence spectra of **pseudo-*p*-NB** in CH_3CN from 430 nm excitation with increasing mol fraction of PEG-400.

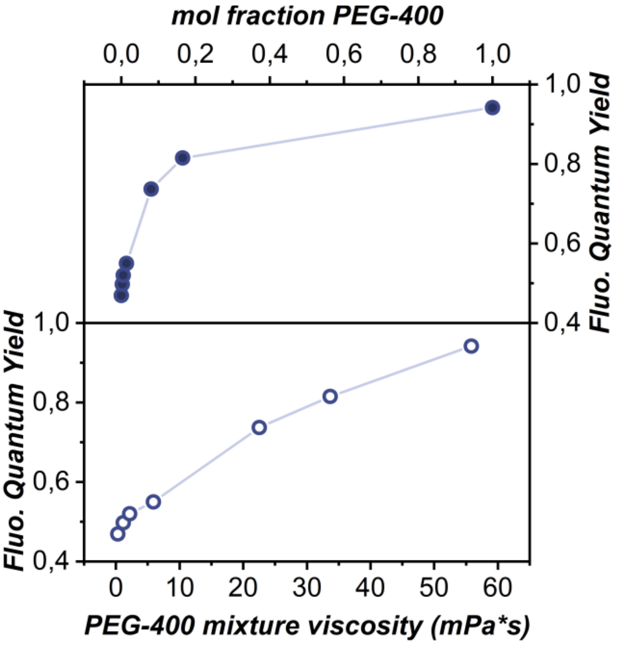

Figure S182. Plots of PEG-400 mol fraction and mixture viscosity versus fluorescence quantum yield for solutions of **pseudo-*p*-NB** in CH_3CN.

Section S20 Electron Paramagnetic Resonance Spectroscopy

Betaines ***m*-PB** and **pseudo-*m*-NB** contain the same *meta*-linkage as the archetypical triplet diradical *meta*-xylylene. As a cautionary investigation, X-band electron paramagnetic resonance (EPR) spectroscopy was conducted but detected no signal from unpaired electrons in CH_2Cl_2 solutions (Section S20). A weak isotropic singlet in the powder EPR was identified as trace paramagnetic impurity by analysing the behaviour of the magnetic moment between 5 – 400 K by superconducting quantum interference device (SQUID) magnetometry (details in Section S21). Compounds ***m*-PB**, **pseudo-*m*-NB**, ***p*-PB**, and **pseudo-*p*-NB** therefore exhibit closed-shell electronic structures with singlet ground states.

Dilution of samples with solvent causes the small signal (visible in the powder EPR spectra) to vanish.

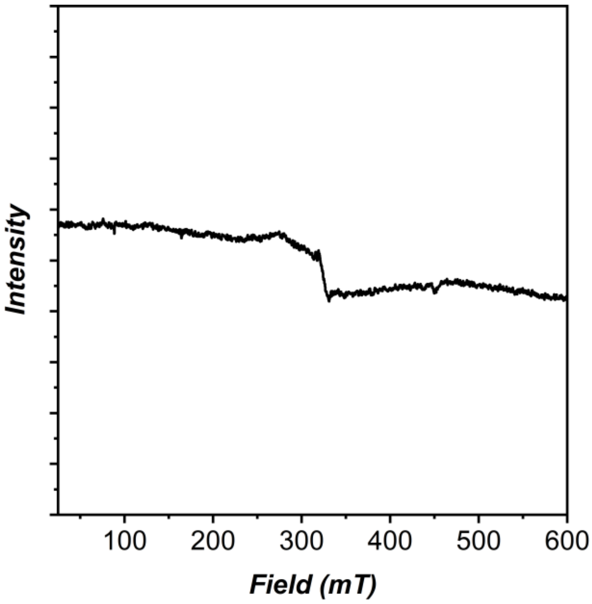

Figure S183. X-band EPR spectrum of blank CH_2Cl_2 recorded at 298 K with sweep time 120 sec, modulation amplitude 1.0 mT, and power attenuation 10 dB.

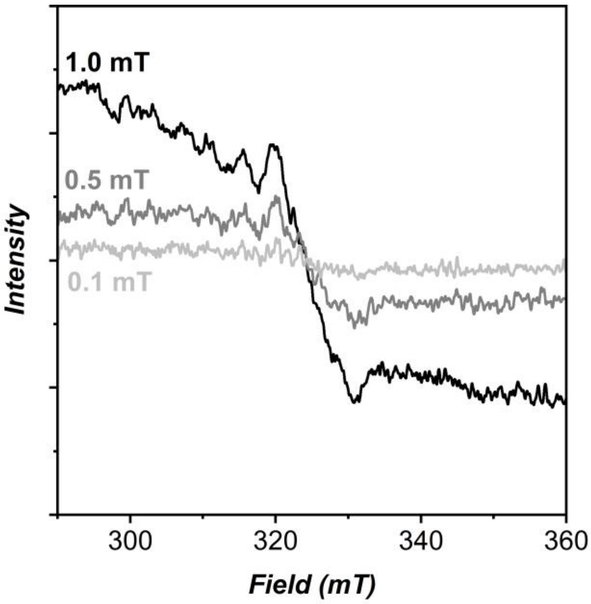

Figure S184. X-band EPR spectrum of blank CH_2Cl_2 recorded at 298 K with various modulation amplitudes.

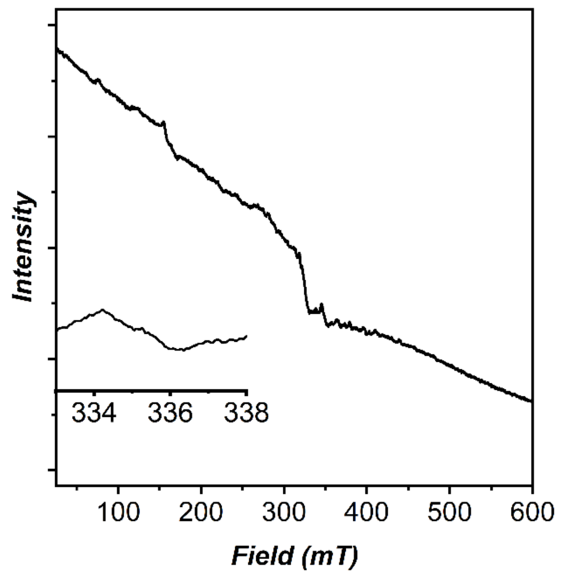

Figure S185. X-band EPR spectrum of blank quartz capillary recorded at 298 K with sweep time 120 sec, modulation amplitude 1.0 mT, and power attenuation 10 dB.

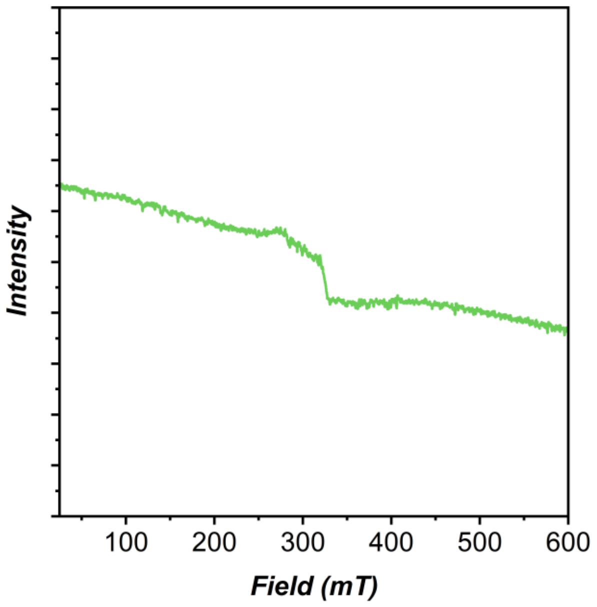

Figure S186. X-band EPR spectrum of 1 mM ***m*-PB** in CH_2Cl_2 recorded at 298 K with sweep time 120 sec, modulation amplitude 1.0 mT, and power attenuation 10 dB.

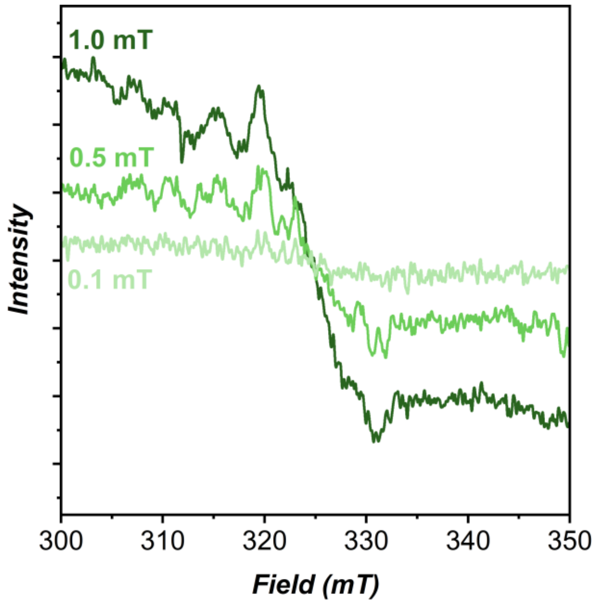

Figure S187. X-band EPR spectrum of 1 mM ***m*-PB** in CH_2Cl_2 recorded at 298 K with sweep time 120 sec, power attenuation 10 dB and various modulation amplitudes.

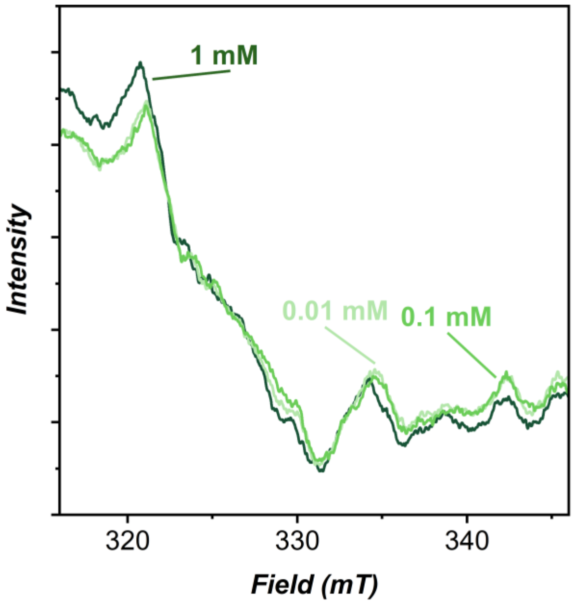

Figure S188. X-band EPR spectrum of ***m*-PB** in CH_2Cl_2 at various concentrations recorded at 298 K with sweep time 120 sec, power attenuation 10 dB, and modulation amplitude 1.0 mT.

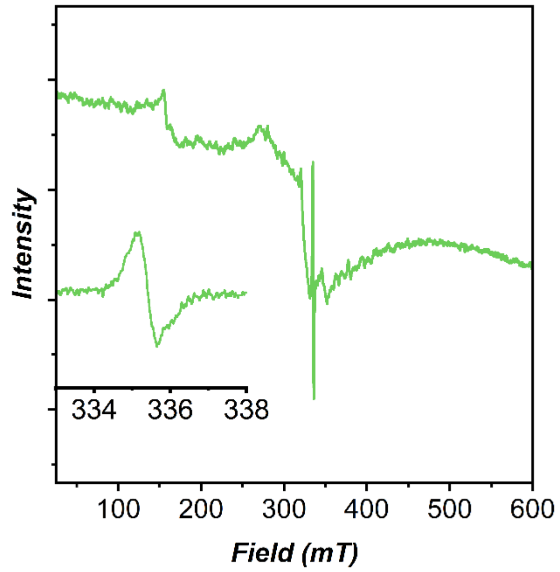

Figure S189. X-band EPR spectrum of ***m*-PB** powder recorded at 298 K with sweep time 120 sec, power attenuation 30 dB, and modulation amplitude 0.1 mT.

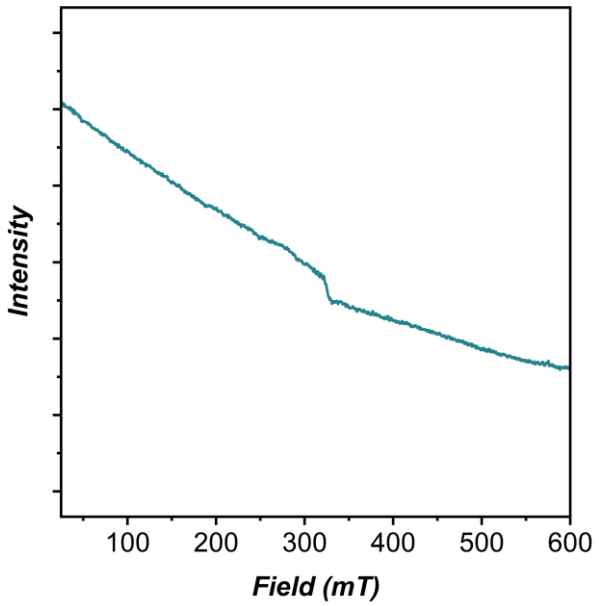

Figure S190. X-band EPR spectrum of 1 mM ***p*-PB** in CH_2Cl_2 recorded at 298 K with sweep time 120 sec, modulation amplitude 1.0 mT, and power attenuation 10 dB.

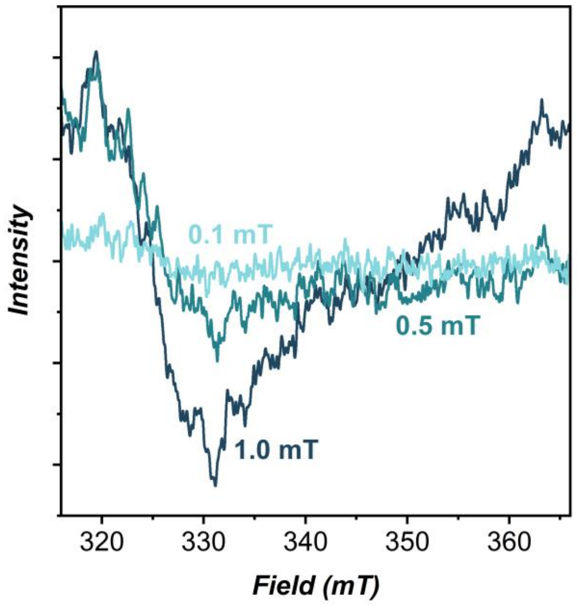

Figure S191. X-band EPR spectrum of 1 mM ***p*-PB** in CH_2Cl_2 recorded at 298 K with sweep time 120 sec, power attenuation 10 dB and various modulation amplitudes.

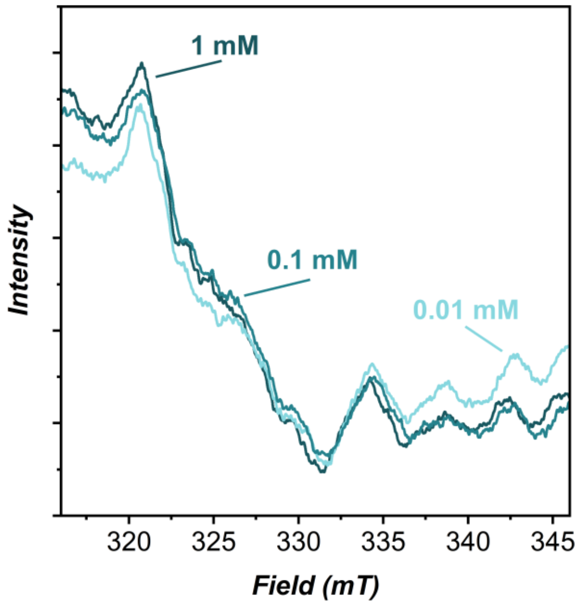

Figure S192. X-band EPR spectrum of ***p*-PB** in CH_2Cl_2 at various concentrations recorded at 298 K with sweep time 120 sec, power attenuation 10 dB, and modulation amplitude 1.0 mT.

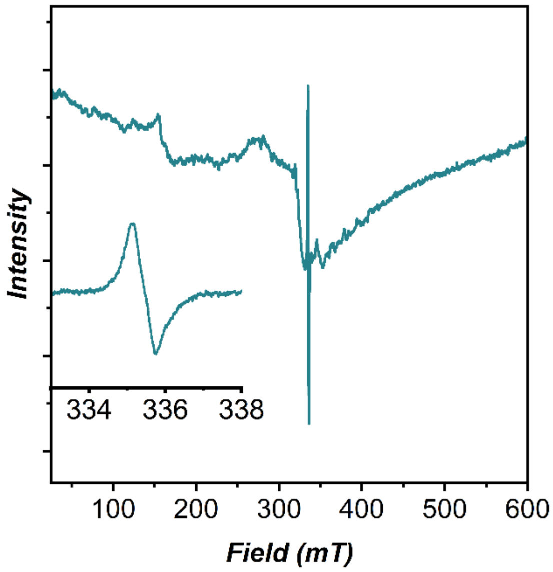

Figure S193. X-band EPR spectrum of ***p*-PB** powder recorded at 298 K with sweep time 120 sec, power attenuation 20 dB, and modulation amplitude 0.1 mT.

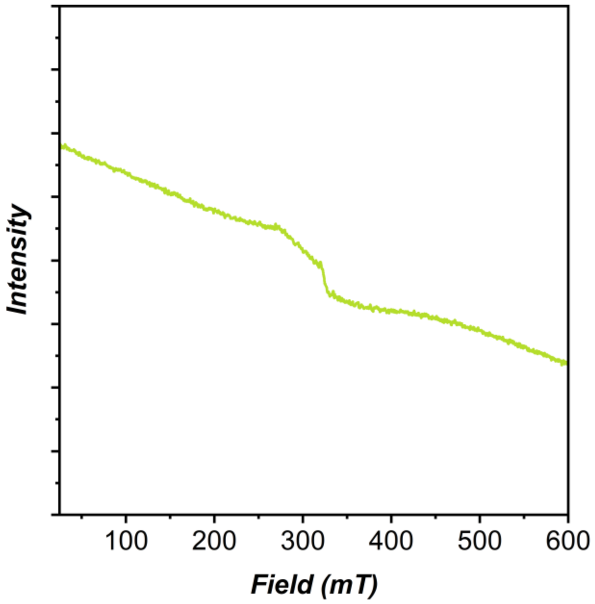

Figure S194. X-band EPR spectrum of 1 mM **pseudo-*m*-NB** in CH_2Cl_2 recorded at 298 K with sweep time 120 sec, modulation amplitude 1.0 mT, and power attenuation 10 dB.

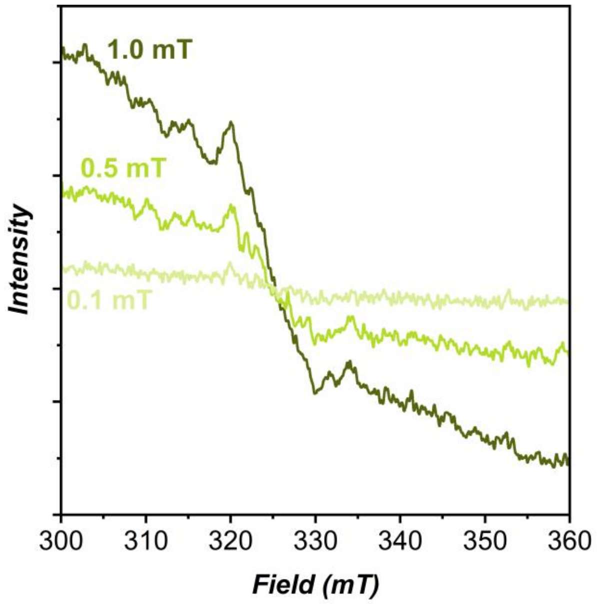

Figure S195. X-band EPR spectrum of 1 mM **pseudo-*m*-NB** in CH_2Cl_2 recorded at 298 K with sweep time 120 sec, power attenuation 10 dB and various modulation amplitudes.

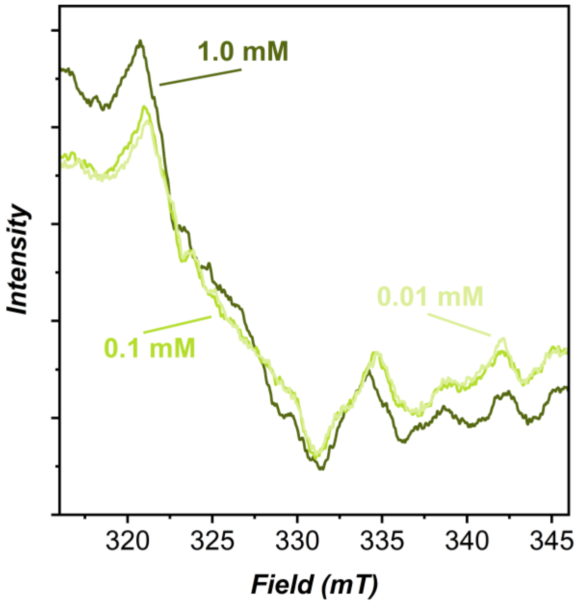

Figure S196. X-band EPR spectrum of ***m*-PB** in CH_2Cl_2 at various concentrations recorded at 298 K with sweep time 120 sec, power attenuation 10 dB, and modulation amplitude 1.0 mT.

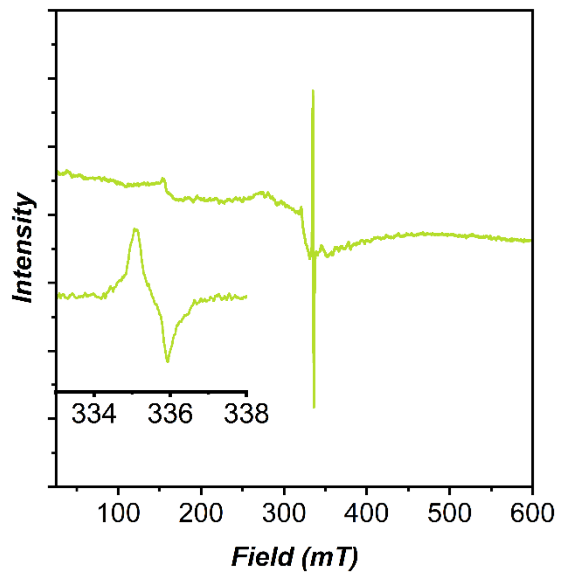

Figure S197. X-band EPR spectrum of **pseudo-*m*-PB** powder recorded at 298 K with sweep time 120 sec, power attenuation 30 dB, and modulation amplitude 0.05 mT.

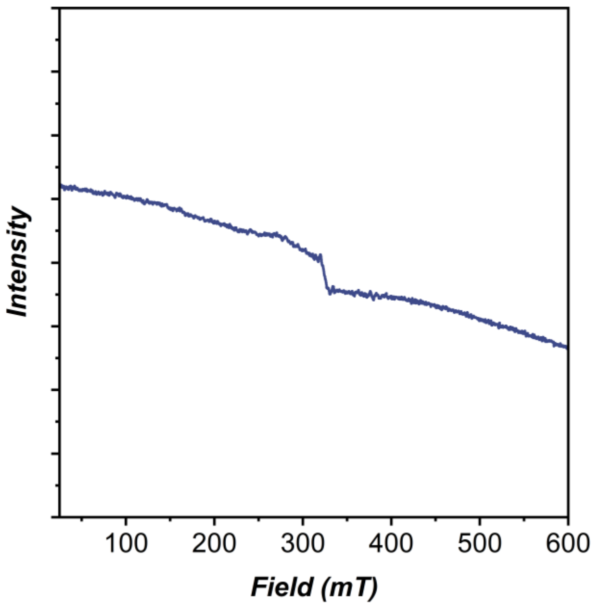

Figure S198. X-band EPR spectrum of 1 mM **pseudo-*p*-NB** in CH_2Cl_2 recorded at 298 K with sweep time 120 sec, modulation amplitude 1.0 mT, and power attenuation 10 dB.

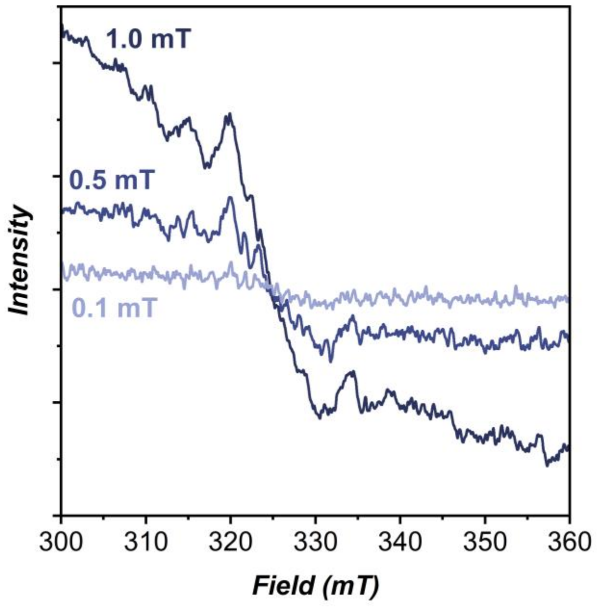

Figure S199. X-band EPR spectrum of 1 mM **pseudo-*p*-NB** in CH_2Cl_2 recorded at 298 K with sweep time 120 sec, power attenuation 10 dB and various modulation amplitudes.

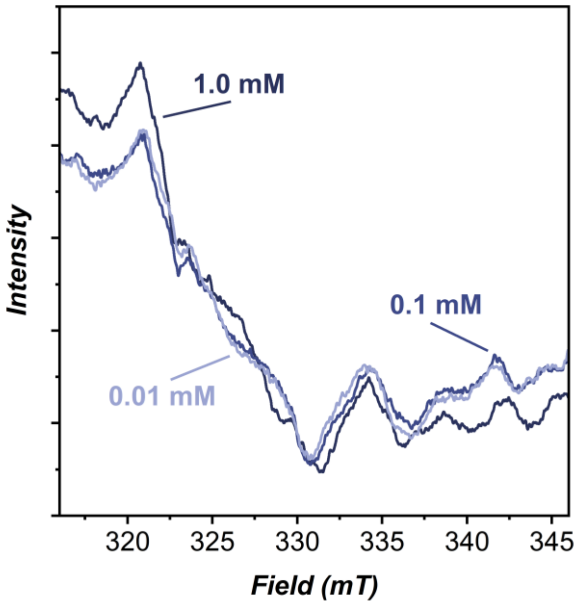

Figure S200. X-band EPR spectrum of **pseudo-*p*-NB** in CH_2Cl_2 at various concentrations recorded at 298 K with sweep time 120 sec, power attenuation 10 dB, and modulation amplitude 1.0 mT.

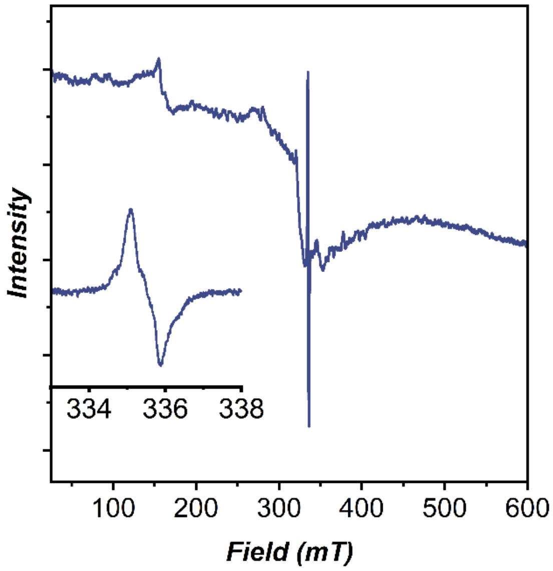

Figure S201. X-band EPR spectrum of **pseudo-*p*-PB** powder recorded at 298 K with sweep time 120 sec, power attenuation 30 dB, and modulation amplitude 0.1 mT.

Section S21 SQUID Magnetometry

The sharp increase in the molar magnetic moments at low temperatures is indicative of a paramagnetic impurity in samples ***m*-PB**, ***p*-PB** and **pseudo-*m*-NB**. The signal for **pseudo-*p*-NB** was not distinguishable from an empty sample capsule.

The diamagnetic core (orbital) susceptibility was calculated by Pascal's method using collected data tables.[76] The diamagnetic susceptibilities found this way were -118.58×10^{-6} emu·mol^{-1} for ***m*-PB** and ***p*-PB**, and -138.40×10^{-6} emu·mol^{-1} for **pseudo-*m*-NB** and **pseudo-*p*-NB**.

The behaviour of the magnetic moment response to temperature was analysed by plotting the product of temperature and molar magnetic susceptibility (χ_mT) versus the temperature and attempting to fit the data to the Bleaney-Bowers equation. This fitting attempt failed, however. The specific form of the Bleaney-Bowers equation is given below, [77] where N is Avogadro's number, μ_B is the Bohr magneton, g is the g-factor, k_B is Boltzmann's constant, e is Euler's number, and $-2J$ is the singlet triplet gap $\Delta E_{S\text{-}T}$.

$$\chi_m T = \frac{2N\mu_B^2 g^2}{k_B\left[3 + e^{\left(\frac{-2J}{k_B T}\right)}\right]}$$

The plotted χ_mT versus T follows neither the expected trend for diradicaloid singlets with a thermally accessible triplet state[77] nor for diradicaloid triplets with a thermally accessible singlet state.[78] Instead,

the linear plot indicates that there is little change in the magnetic moment as the temperature is linearly swept from 5 to 400 K. A constant signal, multiplied by T in this way, would also should a straight line with slope = 1. This is evidence that the electrons are much more strongly coupled than in literature examples. The similarity between *meta*-betaines and the model compound ***p*-PB** with an unequivocal closed shell indicates that all betaines have a closed shell ground state and the moment does not appreciably change in the temperature window.

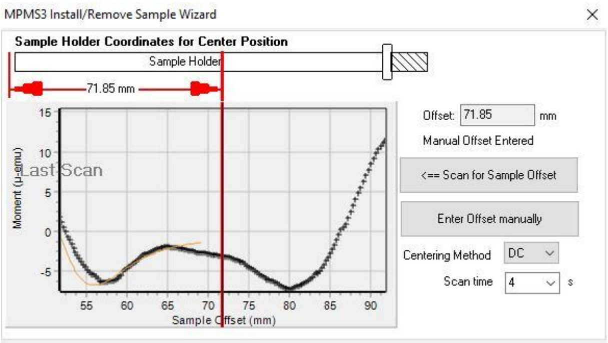

Figure S202. Raw output of moment vs. position at 298 K for a blank sample capsule.

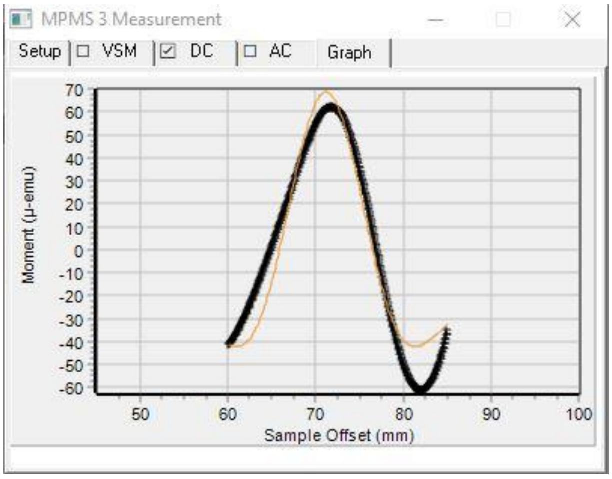

Figure S203. Raw output of moment vs. position at 298 K for ***m*-PB**.

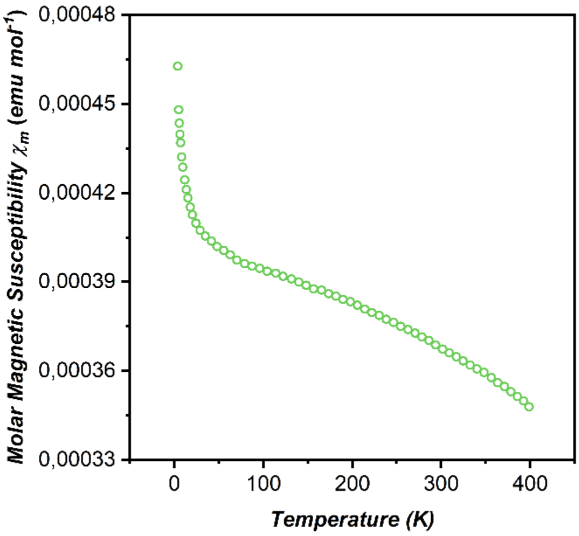

Figure S204. χ_m vs T plot for ***m*-PB** from SQUID data. The sharp increase below 50 K is characteristic of paramagnetism from impurity.

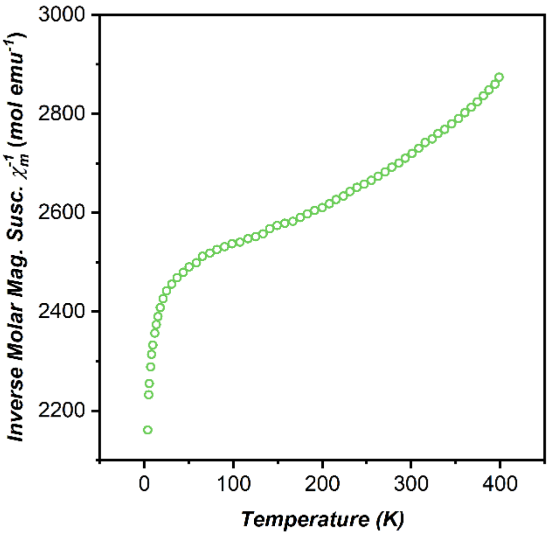

Figure S205. χ_m^{-1} vs T plot for ***m*-PB** from SQUID data. The negative curvature between 100-400 K indicates the presence of temperature-independent contributions to a diamagnetic susceptibility.

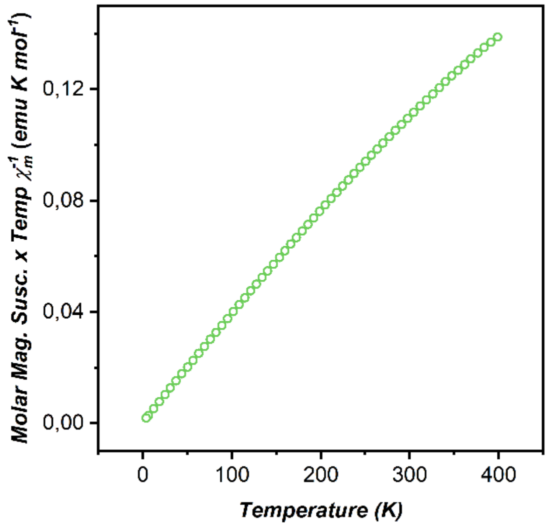

Figure S206. χ_mT vs T plot for ***m*-PB** from SQUID data. Thermally accessible triplets yield signal which increases sigmoidally with T, thermally accessible singlets yield signal which decreases asymptotically with T. Near-linearity indicates very little change in magnetic moment with temperature.

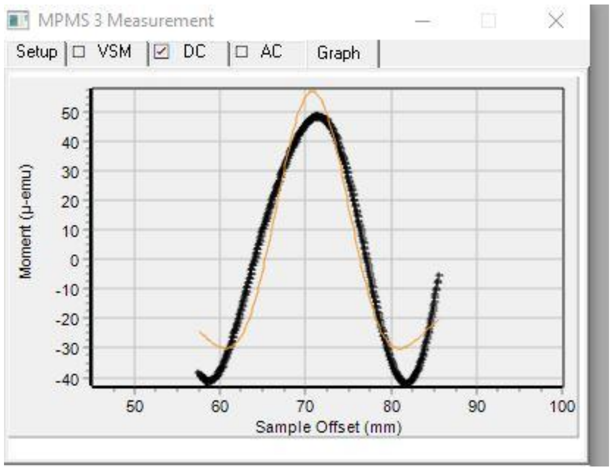

Figure S207. Raw output of moment vs. position at 298 K for ***p*-PB**.

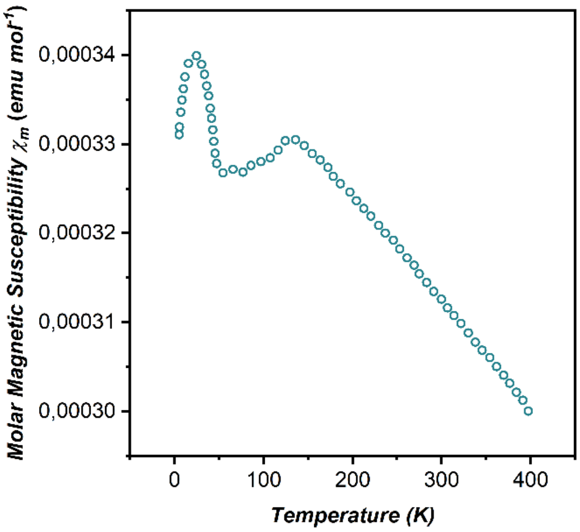

Figure S208. χ_m vs T plot for ***p*-PB** from SQUID data. The peaks around 150 and 50 K are characteristic of magnetic phase transitions of an impurity.

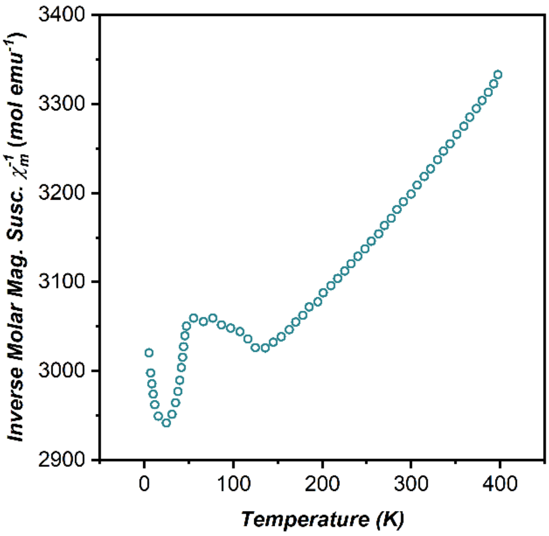

Figure S209. χ_m^{-1} vs T plot for ***p*-PB** from SQUID data. The negative curvature between 100-400 K indicates the presence of temperature-independent contributions to a diamagnetic susceptibility.

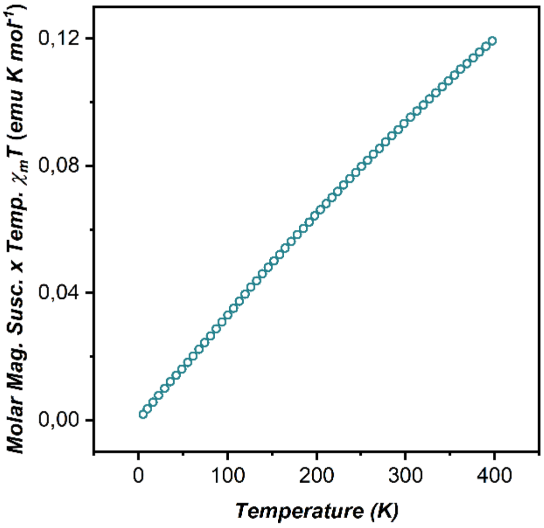

Figure S210. χ_mT vs T plot for ***p*-PB** from SQUID data. Thermally accessible triplets yield signal which increases sigmoidally with T, thermally accessible singlets yield signal which decreases asymptotically with T. Near-linearity indicates very little change in magnetic moment with temperature.

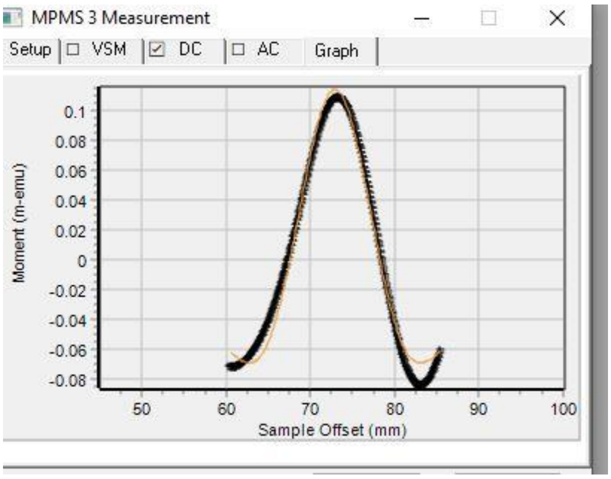

Figure S211. Raw output of moment vs. position at 298 K for **pseudo-*m*-PB**.

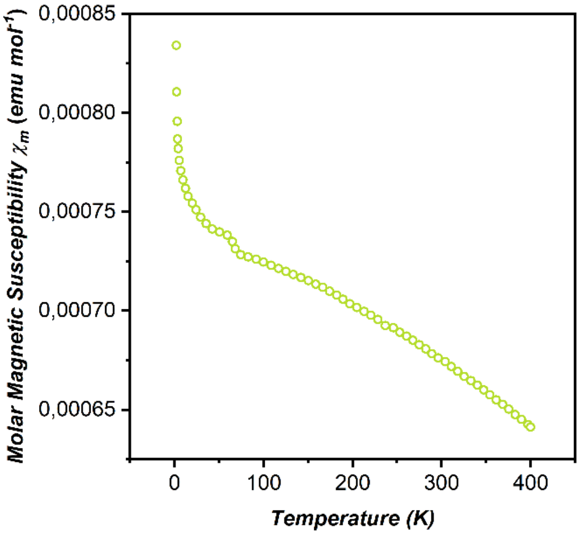

Figure S212. χ_m vs T plot for **pseudo-*m*-NB** from SQUID data. The sharp increase below 50 K is characteristic of paramagnetism from impurity.

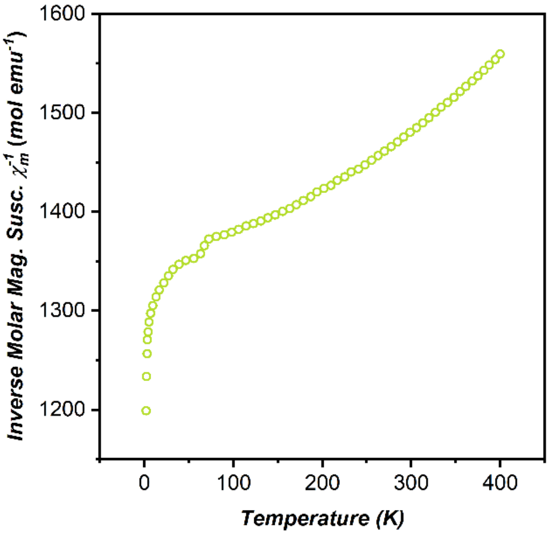

Figure S213. χ_m^{-1} vs T plot for **pseudo-*m*-NB** from SQUID data. The negative curvature between 100-400 K indicates the presence of temperature-independent contributions to a diamagnetic susceptibility.

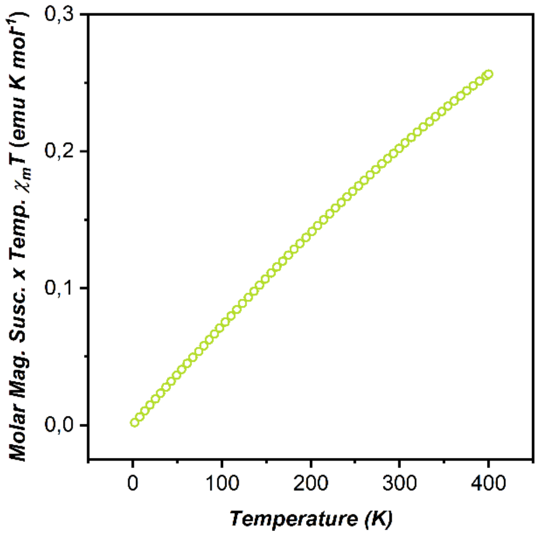

Figure S214. χ_mT vs T plot for **pseudo-*m*-NB** from SQUID data. Thermally accessible triplets yield signal which increases sigmoidally with T, thermally accessible singlets yield signal which decreases asymptotically with T. Near-linearity indicates very little change in magnetic moment with temperature.

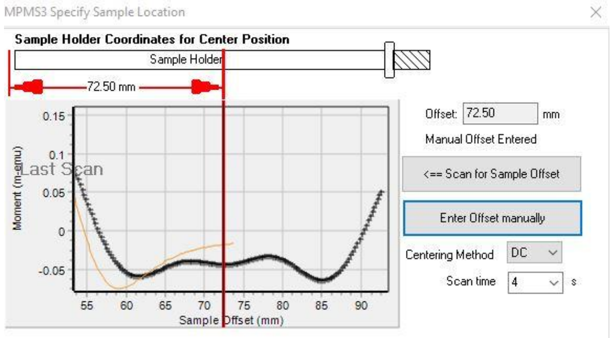

Figure S215. Raw output of moment vs. position at 298 K for **pseudo-*p*-PB**.

Section S22 Predicted Stability of Singlet Betaines versus Triplet Diradicals

Calculation of derivatives with π-donor strengths ranging from weak (-C(CH3)2-) to strong (-N(CH3)-) in the figure below validates our theory that $\Delta E_{S\text{-}T}$ could be modified from −35 to +5 kcal·mol^{-1}. The same substituents on *para*-xylylene have a significantly weaker effect.

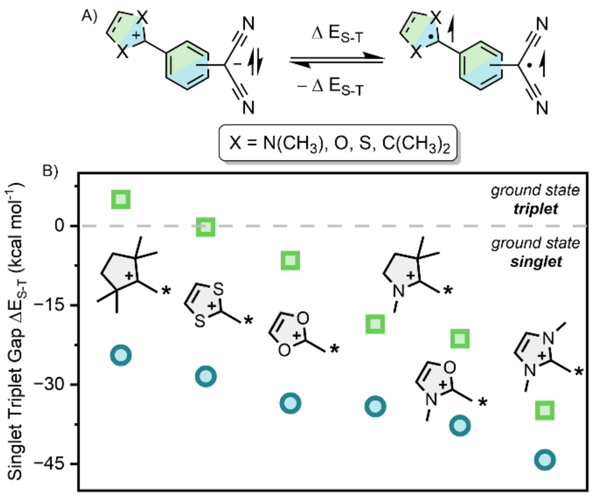

Figure S216. A) Equilibrium between singlet betaines and triplet diradicals based upon ***m*-PB** and ***p*-PB** and B) singlet-triplet gaps for derivatives of ***m*-PB** (green squares) and ***p*-PB** (teal circles) at the M06-2X-D3/6-311++G(3df,3pd) level in gas phase.

Following the rating of various exchange-correlation functionals[79] we employed a few of the better-performers from a variety of 'computational families'[13,80–82] to determine the adiabatic singlet-triplet gap of ***m*-PB** as a model. The reliability of M06-2X/6-311++G(3df,3pd) was shown by Mañeru *et al.* to be valid with respect to the experimental singlet-triplet energy gap, such that it produced only a 7% error in the adiabatic singlet-triplet energy gap.[83]

Calculations for ***m*-PB** in Table S38 are done both in the gas-phase and in CH_3CN (CPCM) with the 6-311++G(d,p) basis set, while those for all betaines in Table S39 are done with the 6-311++G(3df,3pd) basis set. Electronic energies with zero-point energy correction from Gaussian were used for calculation.

Table S38. Relative zpe-corrected electronic energies of singlet versus triplet state of ***m*-PB** in gas and acetonitrile from many functionals.

**Theory**	**State**	**Singlet Energy (kcal·mol^{-1})**	**Triplet Energy (kcal·mol^{-1})**	**Triplet State <S^2> before annihil.**
CAM-B3LYP-D3[84]	CH_3CN	-49.62	0	2.0655
	gas	-29.49	0	2.0785
M05-2X-D3[82]	CH_3CN	-53.08	0	2.0551
	gas	-33.62	0	2.0651
PW6B95-D3[80]	CH_3CN	-49.69	0	2.0320
	gas	-31.05	0	2.0389
ωB97-XD2[81]	CH_3CN	-51.63	0	2.0585
	gas	-31.33	0	2.0696
M06-2X-D3* [85]	CH_3CN	-53.26	0	2.0405
	gas	-34.52	0	2.0464

*calculated with the 6-311++G(3df,3pd) basis set for comparison with literature.[83]

Table S39. Relative zpe-corrected electronic energies of singlet versus triplet states of all compounds.*

compound	State	Betaine Energy (kcal·mol^{-1})	Diradical Energy (kcal·mol^{-1})	Triplet State <S^2> before annihil.
***m*-PB**	CH_3CN	-53.26	0	2.0405
	gas	-34.52	0	2.0464
***p*-PB**	CH_3CN	-59.48	0	2.0128
	gas	-44.04	0	2.0170
**pseudo-*m*-NB**	CH_3CN	-48.38	0	2.0416
	gas	-26.11	0	2.0557
**pseudo-*p*-NB**	CH_3CN	-51.67	0	2.0285
	gas	-33.51	0	2.0224
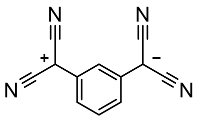	CH_3CN	+19.45	0	2.0639
	gas	+23.49	0	2.0655
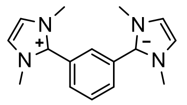	CH_3CN	+3.96	0	2.0319
	gas	+4.78	0	2.0356

*calculated at the M06-2X-D3/6-311++G(3df,3pd) level of theory.

Various common broken-symmetry methods were attempted to model the open-shell singlet state of ***m*-PB**, but each of these only converged to the closed-shell singlet. The closed-shell nature was checked by inspecting the occupancies of the natural orbitals for numbers lesser than 2 and greater than 0. Starting geometries of both singlet or triplet (optimised previously at the M06-2X-D3/6-311++G(3df,3pd) level) were used as input for the single-point calculations. M06-2X, CAM-B3LYP, ωB97X-D, PW6B95 functionals with the 6-31G, 6-311+G(d,p), 6-311++G(3df,3pd) basis sets were tried.

1) the spin-flip procedure from ORCA, flipping one methylene carbon atom, both methylene carbon atoms, then all phenylene ring carbon atoms.
2) the *guess=mix* keyword in Gaussian.
3) restricted-open DFT.

The only method which was capable of achieving an open-shell singlet was UHF with *guess=mix*, which was used for diradical character indices (see below), but energies were not comparable due to lack of electron correlation in HF theory.

The energy of the open-shell singlet state relative to the closed-shell singlet, and the triplet, was indirectly modelled using the state-averaged complete active space SA-CASSCF(2,2) method provided by ORCA. Optimised singlet and triplet geometries at the M06-2X-D3/6-311++G(3df,3pd) level were used to generate wavefunctions at the HF/6-311++G(3df,3pd) level, which were used as input for the SA-CASSCF(2,2) calculations. Singlet and triplet multiplicities were selected.

Table S40. Relative energies of three lowest-lying electronic configurations of all compounds by CASSCF(2,2) calculations.

**compound**	**Starting Geometry**	**Closed-shell Singlet (kcal·mol^{-1})**	**Triplet (kcal·mol^{-1})**	**Open-shell Singlet (kcal·mol^{-1})**
***m*-PB**	singlet	-69.53	0	+23.98
	triplet	-65.97	0	+16.65
***p*-PB**	singlet	-74.26	0	+11.64
	triplet	-75.39	0	+14.80
**pseudo-*m*-NB**	singlet	-31.11	0	+27.85
	triplet	-9.17	0	+26.80
**pseudo-*p*-NB**	singlet	-43.87	0	+44.44
	triplet	-33.71	0	+45.35

The diradical character y_0 was calculated as suggested from literature[86] using a broken-symmetry (*guess=mix* keyword) single-point energy calculation in the gas phase of the open-shell singlet state at the UHF/6-311++G(3df,3pd) level based on a geometry optimized at with the M06-2X-D3 level. DFT methods were avoided because they are reported to overestimate the diradical character,[86] while CASSCF(2,2) calculations using the HOMO/LUMO orbitals yielded similar results to UHF. The natural orbital populations were inputted to the following equation to find the diradical character for each molecule. The highest-occupied and lowest-unoccupied natural orbitals (HONO & LUNO) were determined by finding the adjacent orbitals with 1) the lowest occupancy (n) which was still >1 (HONO) and 2) the orbital with the highest occupancy (n) which was still <1 (LUNO). For reference, the diradical character for naphthalene calculated by the original authors in this way (and replicated by us) is 0.05. These were calculated with Gaussian 16 Rev C 02.

$$y_o = 1 - \frac{2T_0}{1+T_0^2}, \text{ where } T_0 = \frac{n_{HONO} - n_{LUNO}}{2}$$

Table S41. Diradical characters of open-shell singlet states.

	N_{HONO}	n_{LUNO}	Diradical character y_0
***m*-PB**	1.94487	0.05513	0.001
***p*-PB**	1.97891	0.02109	<0.001
**pseudo-*m*-NB**	1.96239	0.03761	0.001
**pseudo-*p*-NB**	1.96997	0.03003	<0.001

To probe the effect of donor-acceptor group substitution on the electronic ground state, the singlet-triplet gap of a series of structures was modelled with ORCA without the rijcosx approximation. The following are thermal & zero-point corrected electronic energies in the gas phase using the M06-2X-D3/6-311++G(3df,3pd) level of theory.

Table S42. Relative zpe-corrected electronic energies of singlet versus triplet states of *meta*-compounds in gas phase.

Structure	Singlet Energy (kcal·mol^{-1})	Triplet Energy (kcal·mol^{-1})	Triplet State $\langle S^2 \rangle$ before annihil.
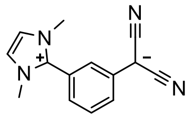	-34.90	0	2.049728
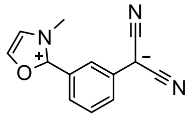	-21.31	0	2.055171
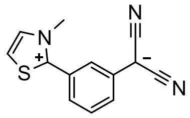	-18.62	0	2.049846
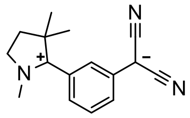	-18.54	0	2.054463
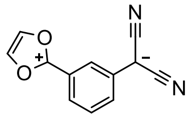	-6.53	0	2.056973
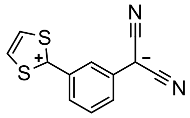	-0.24	0	2.056125
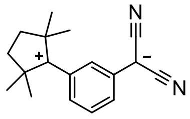	5.03	0	2.043567

Structural data and output files are located in the data repository

The singlet-triplet gap of *para*-analogues of the corresponding compounds from the above Table 43 were determined using the same level of theory. In all cases, the S-T gap for *meta*-substituted compounds is lower and a triplet state may be thermally accessible in a few cases, while all *para*-substituted compounds have a large S-T gap, indicating a thermally inaccessible triplet state.

Table S43. Relative zpe-corrected electronic energies of singlet versus triplet states of *para*-compounds in gas phase.

Structure	Singlet Energy (kcal·mol^{-1})	Triplet Energy (kcal·mol^{-1})	$\Delta E_{meta\text{-}para}$ (kcal·mol^{-1})
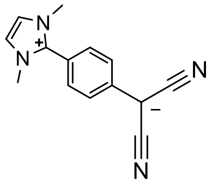	-44.19	0	+9.22
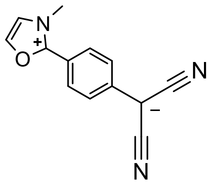	-37.72	0	+16.41
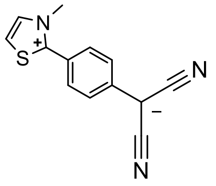	-33.41	0	+14.79
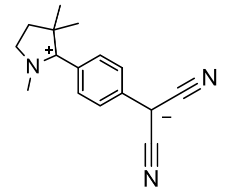	-34.10	0	+15.56
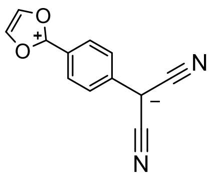	-33.52	0	+26.99
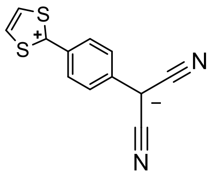	-28.37	0	+28.61
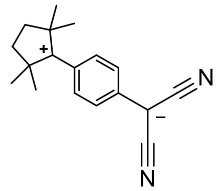	-24.42	0	+29.45

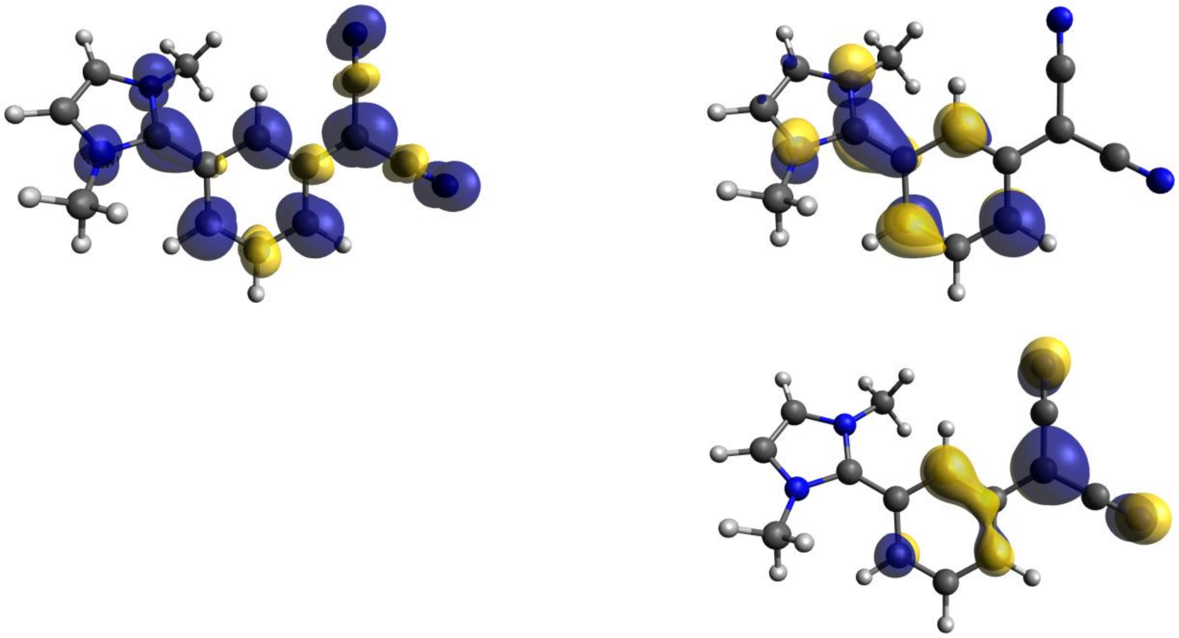

Figure S217. Spin-density surface of triplet diradical form of ***m*-PB** with isovalue=0.005 (left), and two highest-lying biorthogonalised SOMOs with isovalue=0.05 (right).

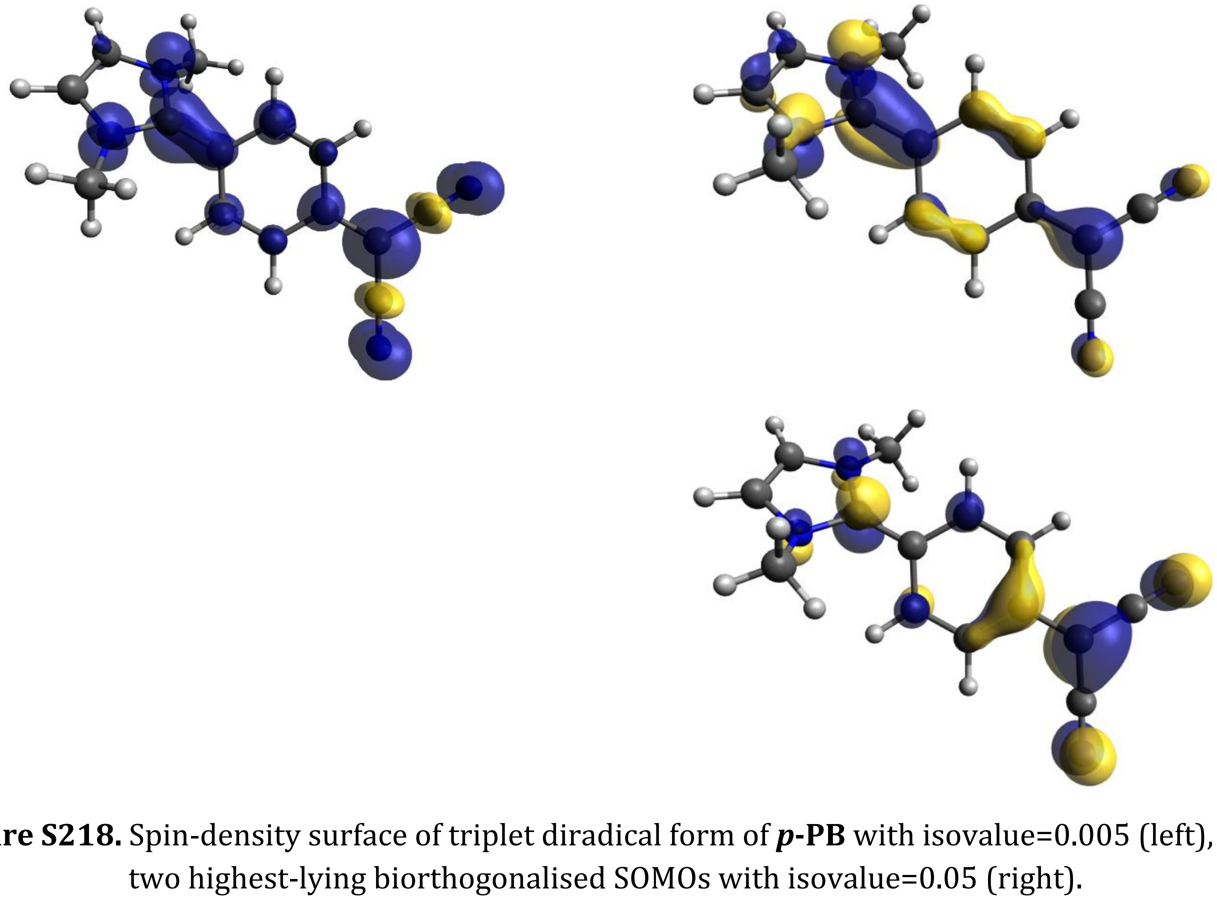

Figure S218. Spin-density surface of triplet diradical form of ***p*-PB** with isovalue=0.005 (left), and two highest-lying biorthogonalised SOMOs with isovalue=0.05 (right).

Section S23 Computational Modelling of Ground States with Point-Charges

When $\Delta E_{S\text{-}T}$ of the simplified *meta*-xylylene core was monitored in the presence of increasingly large point charges using ORCA, the initial preference for the triplet ground state was replaced by the singlet ground state (Section S23). We can infer that the charge-separated character of the zwitterionic singlet state is preferentially stabilised by a strongly polarising environment. The point charges are computational proxies for electronegative atoms which stabilise positive and negative charges, suggesting that *meta*-xylylene derivatives could be designed with variable ground state multiplicity depending on the substituent.

Point charges were added to model the electrostatic effects of donor and acceptor groups. Structures of the *meta*-xylylene and *meta*-naphthylene closed-shell singlet and open-shell triplet optimised in gas phase at M06-2X/6-311++G(3df,3pd) using ORCA (without rijcosx approximation) were aligned with the XY-plane using subfunction 11, subfunction 7 of main function 300 in Multiwfn. Then a positive point charge was added 1 Å above and below one methylene carbon atom, while a corresponding negative point charge was added 1 Å above and below the other methylene carbon atom. This was chosen to dominantly affect the methylene *p*-orbitals and π-system, instead of the σ-system. The singlet-triplet gap was calculated using the energy difference from single-point calculations at M06-2X/6-311++G(3df,3pd). The S-T gap for *meta*-xylylene at zero-charge was satisfactorily similar to literature (36 kcal·mol^{-1} vs 33 kcal·mol^{-1}), even without a zpe-correction.[83] The S-T gap decreases and eventually switches sign at higher point charge magnitude, indicating that the ground state could be selectively switched if sufficiently polarising substituents are appended to the terminal carbon atoms.

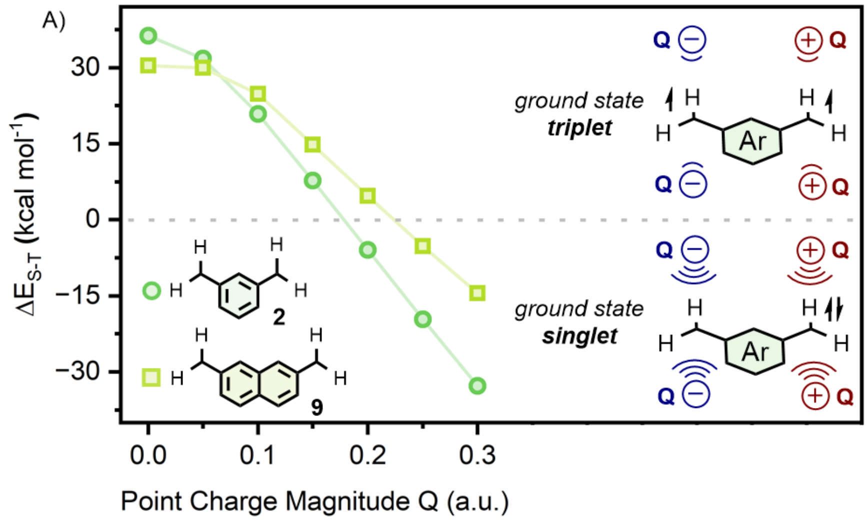

Figure S219. Calculated singlet-triplet gap as a function of point charge magnitude for *meta*-xylylene (green circles) and 2,7-di(methylene)naphthylene (lime squares).

meta-xylylene triplet in gas phase M06-2X

Atomic Type	X	Y	Z
C	0,027154	0,036461	0,00003
C	-1,175366	0,771626	0,000028
C	-2,437404	0,145195	0,000021
C	-2,470373	-1,278139	0,000017
C	-1,297822	-2,013143	0,000018
C	-0,064935	-1,384235	0,000024
H	0,846599	-1,966469	0,000025
H	-1,345448	-3,093525	0,000015
H	-3,429396	-1,778218	0,000011
C	-3,619886	0,893225	0,000018
H	-4,583108	0,406301	-0,000001
H	-3,59356	1,972238	0,000008
H	-1,127242	1,853574	0,000031
C	1,270502	0,677826	0,000033
H	2,187032	0,107682	-0,000018
H	1,339862	1,75493	-0,000003

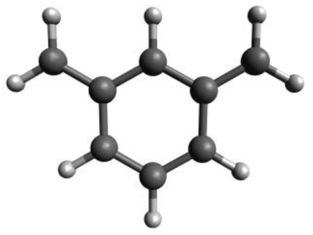

E(M06-2X) -309.532997919158 a.u.
Lowest Vibration 183.95 cm^{-1}
Imaginary Frequencies 0

meta-xylylene singlet in gas phase M06-2X

Atomic Type	X	Y	Z
C	0,038471	-0,044394	-0,000105
C	-1,179234	0,68432	-0,000114
C	-2,455825	0,065926	-0,000103
C	-2,495478	-1,351275	-0,000023
C	-1,299344	-2,05258	0,000013
C	-0,046172	-1,459388	-0,000027
H	0,855742	-2,051735	-0,000007
H	-1,347133	-3,134708	0,000062
H	-3,445673	-1,862529	-0,000001
C	-3,515989	0,964218	-0,000136
H	-4,543559	0,629078	0,000401
H	-3,333065	2,028061	0,000084
H	-1,130838	1,767317	-0,00015
C	1,173132	0,757182	-0,000134
H	2,167255	0,332861	0,000389
H	1,084321	1,832975	0,000108

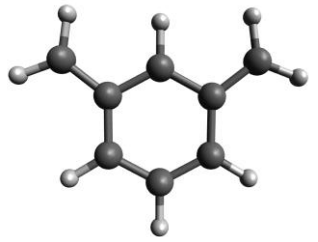

E(M06-2X) -309.475198406251 a.u.
Lowest Vibration 104.79 cm^{-1}
Imaginary Frequencies 0

2,7-di(methylene)naphthylene triplet in gas phase M06-2X

Atomic Type	X	Y	Z
C	-0,006614	0,10623	0,133024
C	-1,229082	0,772779	0,095134
C	-1,281662	2,206518	0,004355
C	-2,469874	2,867442	-0,033028
C	-3,703011	2,171493	0,017125
C	-4,950908	2,841037	-0,017769
C	-6,124246	2,15498	0,034575
C	-6,14527	0,720621	0,126634
C	-7,352977	0,028858	0,181112
H	-7,375022	-1,048208	0,249875
H	-8,295535	0,554407	0,154432
C	-4,909829	0,052529	0,159439
C	-3,687193	0,746057	0,107681
C	-2,449618	0,078753	0,143377
H	-2,438889	-1,002039	0,212029
H	-4,896214	-1,028178	0,22852
H	-7,068143	2,683168	0,007432
H	-4,954737	3,921416	-0,087378
H	-2,490401	3,94766	-0,101956
H	-0,349316	2,754208	-0,03354
H	0,03712	-0,970029	0,203838
H	0,924082	0,651968	0,098503

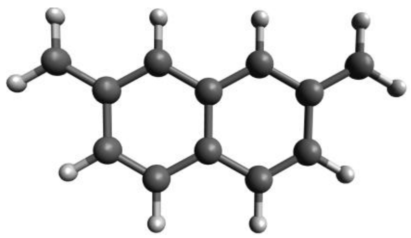

**E(M06-2X)**	-463.16369897 a.u.
**Lowest Vibration**	90.34 cm^{-1}
**Imaginary Frequencies**	0

2,7-di(methylene)naphthylene singlet in gas phase M06-2X

Atomic Type	X	Y	Z
C	0,036518	0,198849	0,125683
C	-1,225176	0,741163	0,097292
C	-1,294782	2,182062	0,013359
C	-2,487017	2,824471	-0,020721
C	-3,7021	2,088566	0,027695
C	-4,933047	2,798161	-0,006869
C	-6,110536	2,130178	0,0399
C	-6,148497	0,687965	0,123904
C	-7,398321	0,119689	0,165554
H	-7,528099	-0,951441	0,229588
H	-8,285318	0,735004	0,136383
C	-4,923773	-0,008894	0,155854
C	-3,686146	0,647967	0,111834
C	-2,434329	0,018171	0,142666
H	-2,392584	-1,060714	0,20583
H	-4,941422	-1,088442	0,218884
H	-7,050422	2,666717	0,015239
H	-4,905542	3,877669	-0,07039
H	-2,538392	3,903093	-0,084243
H	-0,366985	2,738719	-0,02104
H	0,188248	-0,869393	0,189713
H	0,910384	0,832113	0,0873

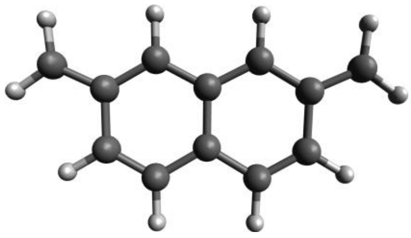

**E(M06-2X)**	-463.116082353 a.u.
**Lowest Vibration**	78.49 cm^{-1}
**Imaginary Frequencies**	0

Section S24 References

1 G. R. Fulmer, A. J. M. Miller, N. H. Sherden, H. E. Gottlieb, A. Nudelman, B. M. Stoltz, J. E. Bercaw and K. I. Goldberg, *Organometallics*, 2010, **29**, 2176–2179.
2 O. V. Dolomanov, L. J. Bourhis, R. J. Gildea, J. A. K. Howard and H. Puschmann, *Journal of Applied Crystallography*, 2009, **42**, 339–341.
3 G. M. Sheldrick, *Acta Crystallogr A Found Adv*, 2015, **71**, 3–8.
4 G. M. Sheldrick, *Acta Crystallogr C Struct Chem*, 2015, **71**, 3–8.
5 S. Fery-Forgues and D. Lavabre, *J. Chem. Educ.*, 1999, **76**, 1260.
6 K. Suzuki, A. Kobayashi, S. Kaneko, K. Takehira, T. Yoshihara, H. Ishida, Y. Shiina, S. Oishi and S. Tobita, *Phys. Chem. Chem. Phys.*, 2009, **11**, 9850.
7 R. F. Chen, *Analytical Letters*, 1967, **1**, 35–42.
8 R. Sens and K. H. Drexhage, *Journal of Luminescence*, 1981, **24–25**, 709–712.
9 Frisch, M. J.; Trucks, G. W.; Schlegel, H. B.; Scuseria, G. E.; Robb, M. A.; Cheeseman, J. R.; Scalmani, G.; Barone, V.; Petersson, G. A.; Nakatsuji, H.; Li, X.; Caricato, M.; Marenich, A. V.; Bloino, J.; Janesko, B. G.; Gomperts, R.;, Mennucci, B.; Hratchian, H. P.; Ortiz, J. V.; Izmaylov, A. F.; Sonnenberg, J. L.; Williams-Young, D.; Ding, F.; Lipparini, F.; Egidi, F.; Goings, J.; Peng, B.; Petrone, A.; Henderson, T.; Ranasinghe, D.; Zakrzewski, V. G.; Gao, J.; Rega, N.; Zheng, G.; Li, Ehara, M.; Toyota, K.; Fukuda, R.; Hasegawa, J.; Ishida, M.; Nakajima, T.; Honda, Y.; Kitao, O.; Nakai, H.; Vreven, T.; Throssell, K.; Montgomery, J. A., Jr.;, Peralta, J. E.; Ogliaro, F.; Bearpark, M. J.; Heyd, J. J.; Brothers, E. N.; Kudin, K. N.; Staroverov, V. N.; Keith, T. A.; Kobayashi, R.; Normand, J.; Raghavachari, K.; Rendell, A. P.; and Burant, J. C.; Iyengar, S. S.; Tomasi, J.; Cossi, M.; Millam, J. M.; Klene, M.; Adamo, C.; Cammi, R.; Ochterski, J. W.; Martin, R. L.; Morokuma, K.; Farkas, O.; Foresman, J. B.; Fox, D. J., Gaussian 16 (version Revision C.02) Gaussian Inc., Wallingford, CT 2016.
10 J. Andzelm, C. Kölmel and A. Klamt, *The Journal of Chemical Physics*, 1995, **103**, 9312–9320.
11 D. M. York and M. Karplus, *J. Phys. Chem. A*, 1999, **103**, 11060–11079.
12 M. Cossi, N. Rega, G. Scalmani and V. Barone, *J Comput Chem*, 2003, **24**, 669–681.
13 S. Grimme, J. Antony, S. Ehrlich and H. Krieg, *The Journal of Chemical Physics*, 2010, **132**, 154104.
14 L. Bennett, B. Melchers and B. Proppe, *Freie Universität Berlin*, 2020, preprint, DOI: 10.17169/REFUBIUM-26754.
15 M. Garcia-Ratés and F. Neese, *J Comput Chem*, 2020, **41**, 922–939.
16 F. Neese, *J Comput Chem*, 2003, **24**, 1740–1747.
17 F. Neese, F. Wennmohs, A. Hansen and U. Becker, *Chemical Physics*, 2009, **356**, 98–109.
18 F. Neese, *J Comput Chem*, 2023, **44**, 381–396.
19 F. Neese, *WIREs Comput Mol Sci*, 2012, **2**, 73–78.
20 D. Bykov, T. Petrenko, R. Izsák, S. Kossmann, U. Becker, E. Valeev and F. Neese, *Molecular Physics*, 2015, **113**, 1961–1977.
21 M. Garcia-Ratés and F. Neese, *J Comput Chem*, 2019, **40**, 1816–1828.
22 F. Neese, F. Wennmohs, U. Becker and C. Riplinger, *The Journal of Chemical Physics*, 2020, **152**, 224108.
23 B. De Souza, F. Neese and R. Izsák, *The Journal of Chemical Physics*, 2018, **148**, 034104.
24 F. Weigend, *Phys. Chem. Chem. Phys.*, 2006, **8**, 1057.
25 F. Weigend and R. Ahlrichs, *Phys. Chem. Chem. Phys.*, 2005, **7**, 3297.
26 W. F. Polik and J. R. Schmidt, *WIREs Comput Mol Sci*, 2022, **12**, e1554.
27 A. V. Marenich, C. J. Cramer and D. G. Truhlar, *J. Phys. Chem. B*, 2009, **113**, 6378–6396.
28 L. Wittmann, I. Gordiy, M. Friede, B. Helmich-Paris, S. Grimme, A. Hansen and M. Bursch, *Phys. Chem. Chem. Phys.*, 2024, **26**, 21379–21394.
29 C. Plett, M. Stahn, M. Bursch, J.-M. Mewes and S. Grimme, *J. Phys. Chem. Lett.*, 2024, **15**, 2462–2469.
30 B. Helmich-Paris, B. De Souza, F. Neese and R. Izsák, *The Journal of Chemical Physics*, 2021, **155**, 104109.
31 R. Izsák, F. Neese and W. Klopper, *The Journal of Chemical Physics*, 2013, **139**, 094111.
32 F. Neese and G. Olbrich, *Chemical Physics Letters*, 2002, **362**, 170–178.
33 F. Jensen, *J. Chem. Theory Comput.*, 2015, **11**, 132–138.
34 R. Izsák, A. Hansen and F. Neese, *Molecular Physics*, 2012, **110**, 2413–2417.
35 K. Wolinski, J. F. Hinton and P. Pulay, *J. Am. Chem. Soc.*, 1990, **112**, 8251–8260.
36 C. McMichael Rohlfing, L. C. Allen and R. Ditchfield, *Chemical Physics*, 1984, **87**, 9–15.
37 R. Ditchfield, *Molecular Physics*, 1974, **27**, 789–807.
38 R. Ditchfield, *The Journal of Chemical Physics*, 1972, **56**, 5688–5691.
39 G. L. Stoychev, A. A. Auer, R. Izsák and F. Neese, *J. Chem. Theory Comput.*, 2018, **14**, 619–637.
40 T. Lu, *The Journal of Chemical Physics*, 2024, **161**, 082503.
41 T. Lu and F. Chen, *J Comput Chem*, 2012, **33**, 580–592.
42 T. M. Krygowski, *J. Chem. Inf. Comput. Sci.*, 1993, **33**, 70–78.
43 J. Kruszewski and T. M. Krygowski, *Tetrahedron Letters*, 1972, **13**, 3839–3842.
44 E. M. Arpa, S. Stafström and B. Durbeej, *J. Org. Chem.*, 2025, **90**, 1297–1308.
45 E. M. Arpa and B. Durbeej, *Phys. Chem. Chem. Phys.*, 2023, **25**, 16763–16771.

46 E. Matito, M. Duran and M. Solà, *The Journal of Chemical Physics*, 2005, **122**, 014109.
47 T. Lu and F. Chen, *J. Phys. Chem. A*, 2013, **117**, 3100–3108.
48 Z. Liu, T. Lu and Q. Chen, *Carbon*, 2020, **165**, 461–467.
49 M. D. Hanwell, D. E. Curtis, D. C. Lonie, T. Vandermeersch, E. Zurek and G. R. Hutchison, *J Cheminform*, 2012, **4**, 17.
50 S. Haneda, A. Okui, C. Ueba and M. Hayashi, *Tetrahedron*, 2007, **63**, 2414–2417.
51 A. Prades, M. Viciano, M. Sanaú and E. Peris, *Organometallics*, 2008, **27**, 4254–4259.
52 T. Sakamoto, E. Katoh, Y. Kondo and H. Yamanaka, *Chem. Pharm. Bull.*, 1988, **36**, 1664–1668.
53 D. Zhang, M. Lian, J. Liu, S. Tang, G. Liu, C. Ma, Q. Meng, H. Peng and D. Zhu, *Org. Lett.*, 2019, **21**, 2597–2601.
54 G. L. P. Aydos, G. Marin, G. Ebeling, F. P. Dos Santos, B. C. Leal, R. D. Zink, B. A. Vargas, P. Migowski, R. Stieler, B. B. De Araújo, P. Gonçalves, H. K. Stassen, L. Dos Santos Pereira, A. P. Musse and J. Dupont, *ChemSusChem*, 2023, **16**, e202300971.
55 N. Todorovic, E. Awuah, S. Albu, C. Ozimok and A. Capretta, *Org. Lett.*, 2011, **13**, 6180–6183.
56 T. X. Neenan and G. M. Whitesides, *J. Org. Chem.*, 1988, **53**, 2489–2496.
57 G. M. Paternò, Q. Chen, X. Wang, J. Liu, S. G. Motti, A. Petrozza, X. Feng, G. Lanzani, K. Müllen, A. Narita and F. Scotognella, *Angew Chem Int Ed*, 2017, **56**, 6753–6757.
58 K. J. Schwarz, C. Yang, J. W. B. Fyfe and T. N. Snaddon, *Angew Chem Int Ed*, 2018, **57**, 12102–12105.
59 W. D. Ollis, S. P. Stanforth and C. A. Ramsden, *Tetrahedron*, 1985, **41**, 2239–2329.
60 L.-P. Zhang, J.-F. Ma and G.-J. Ping, 2007.
61 P. Molina, C. López-Leonardo, J. Llamas-Botía, C. Foces-Foces and C. Fernandez-Castaño, *J. Chem. Soc., Chem. Commun.*, 1995, 1387–1389.
62 *Proc. R. Soc. Lond. A*, 1951, **207**, 306–320.
63 A. J. Arduengo, H. V. R. Dias, R. L. Harlow and M. Kline, *J. Am. Chem. Soc.*, 1992, **114**, 5530–5534.
64 C.-H. Wu, L. J. Karas, H. Ottosson and J. I.-C. Wu, *Proc. Natl. Acad. Sci. U.S.A.*, 2019, **116**, 20303–20308.
65 A. Abbotto, S. Bradamante and G. A. Pagani, *J. Org. Chem.*, 1993, **58**, 449–455.
66 P. Rietsch, F. Witte, S. Sobottka, G. Germer, A. Krappe, A. Güttler, B. Sarkar, B. Paulus, U. Resch-Genger and S. Eigler, *Angew Chem Int Ed*, 2019, **58**, 8235–8239.
67 K. Vandewal, J. Benduhn and V. C. Nikolis, *Sustainable Energy Fuels*, 2018, **2**, 538–544.
68 T. Kawase, T. Iwata and M. Oda, *Chemistry Letters*, 2003, **32**, 322–323.
69 A. Dhara, T. Sadhukhan, E. G. Sheetz, A. H. Olsson, K. Raghavachari and A. H. Flood, *J. Am. Chem. Soc.*, 2020, **142**, 12167–12180.
70 C. Reichardt, *Chem. Rev.*, 1994, **94**, 2319–2358.
71 E. Lippert, *Zeitschrift für Elektrochemie, Berichte der Bunsengesellschaft für physikalische Chemie*, 1957, **61**, 962–975.
72 N. Mataga, Y. Kaifu and M. Koizumi, *Bulletin of the Chemical Society of Japan*, 1956, **29**, 465–470.
73 J. Catalán, *J. Phys. Chem. B*, 2009, **113**, 5951–5960.
74 E. Caldeweyher, J.-M. Mewes, S. Ehlert and S. Grimme, *Phys. Chem. Chem. Phys.*, 2020, **22**, 8499–8512.
75 S. Bharathi and M. C. S. Subha, *Russ. J. Phys. Chem.*, 2021, **95**, S201–S208.
76 G. A. Bain and J. F. Berry, *J. Chem. Educ.*, 2008, **85**, 532.
77 J. Shen, Y. Han, S. Dong, H. Phan, T. S. Herng, T. Xu, J. Ding and C. Chi, *Angew Chem Int Ed*, 2021, **60**, 4464–4469.
78 Z. Zhu, D. Zhang, T. Xiao, Y. Fang, X. Xiao, X. Wang, S. Jiang and D. Zhao, *Angew Chem Int Ed*, 2023, **62**, e202314900.
79 L. Goerigk, A. Hansen, C. Bauer, S. Ehrlich, A. Najibi and S. Grimme, *Phys. Chem. Chem. Phys.*, 2017, **19**, 32184–32215.
80 Y. Zhao and D. G. Truhlar, *J. Phys. Chem. A*, 2005, **109**, 5656–5667.
81 J.-D. Chai and M. Head-Gordon, *Phys. Chem. Chem. Phys.*, 2008, **10**, 6615.
82 Y. Zhao, N. E. Schultz and D. G. Truhlar, *J. Chem. Theory Comput.*, 2006, **2**, 364–382.
83 D. Reta Mañeru, A. K. Pal, I. D. P. R. Moreira, S. N. Datta and F. Illas, *J. Chem. Theory Comput.*, 2014, **10**, 335–345.
84 T. Yanai, D. P. Tew and N. C. Handy, *Chemical Physics Letters*, 2004, **393**, 51–57.
85 Y. Zhao and D. G. Truhlar, *Theor Chem Account*, 2008, **120**, 215–241.
86 K. Kamada, K. Ohta, A. Shimizu, T. Kubo, R. Kishi, H. Takahashi, E. Botek, B. Champagne and M. Nakano, *J. Phys. Chem. Lett.*, 2010, **1**, 937–940.
